# Temporal changes in sex‐ and age‐specific incidence profiles of mental disorders—A nationwide study from 1970 to 2016

**DOI:** 10.1111/acps.13410

**Published:** 2022-02-18

**Authors:** Oleguer Plana‐Ripoll, Natalie C. Momen, John J. McGrath, Theresa Wimberley, Isabell Brikell, Diana Schendel, Malene Thygesen, Nanna Weye, Carsten B. Pedersen, Ole Mors, Preben B. Mortensen, Søren Dalsgaard

**Affiliations:** ^1^ National Centre for Register‐based Research Aarhus University Aarhus Denmark; ^2^ Department of Clinical Epidemiology Aarhus University and Aarhus University Hospital Aarhus Denmark; ^3^ Queensland Centre for Mental Health Research Wacol Qld Australia; ^4^ Queensland Brain Institute University of Queensland St. Lucia Qld Australia; ^5^ iPSYCH – The Lundbeck Foundation Initiative for Integrative Psychiatric Research Copenhagen Denmark; ^6^ iPSYCH – The Lundbeck Foundation Initiative for Integrative Psychiatric Research Aarhus Denmark; ^7^ CIRRAU – Centre for Integrated Register‐based Research Aarhus University Aarhus Denmark; ^8^ Department of Medical Epidemiology and Biostatistics Karolinska Institute Stockholm Sweden; ^9^ AJ Drexel Autism Institute Drexel University Philadelphia Pennsylvania USA; ^10^ Big Data Centre for Environment and Health BERTHA, Aarhus University Aarhus Denmark; ^11^ Department of Clinical Medicine Aarhus University Aarhus Denmark

**Keywords:** incidence, mental disorders, register‐based epidemiology, time trends

## Abstract

**Objective:**

Information on mental disorders over time is critical for documenting changes in population burden, and aiding understanding of potential causal and non‐causal factors. The aim of this study was to provide temporal changes in the sex‐ and age‐specific incidence rates (IR) of mental disorders diagnosed in Danish hospitals during five decades and investigate whether such changes may be attributable to changes in administrative reporting practice.

**Methods:**

This population‐based cohort study included all people living in Denmark between 1970 and 2016. Mental disorders diagnoses were obtained from the Danish Psychiatric Central Research Register. We estimated the IR of each mental disorder (all persons, and sex‐ and age‐specific IRs) and examined the impact of two administrative changes.

**Results:**

Our study included 9 107 157 people, followed for 233.0 million person‐years. During follow‐up, 9.5% were diagnosed with at least one mental disorder. The IR for any mental disorder was 39.0 per 10,000 person‐years. Despite fluctuations, this increased between 1970–84 and 2005–2016, from 28.9 to 63.0 per 10,000 person‐years. Increases were most pronounced for younger age groups. Administrative changes did appear to influence incidence rates.

**Conclusion:**

Mental disorder IRs have increased in Denmark since 1970, with age of diagnosis shifting downwards. Both trends were likely impacted by administrative changes, while the latter is likely to be (partly) attributable to earlier detection and increased reporting of child‐onset conditions. Our findings may provide valuable context of the epidemiology of mental disorders across age groups for comparison with other studies and populations.

**Significant outcomes**

- Incidence rates of mental disorders varied across time period: for most mental disorders, they decreased from 1970–84 to 1985–1994, but they increased from 1995 onwards.
- The majority of first mental disorder diagnoses in the 1970s and 1980s were between 25 and 50 years old; however, from the mid‐1990s onwards, an increasing number of individuals were diagnosed between age 15 and 25 years.
- Some of the changes in incidence rates coincided with known changes in diagnostic and reporting practices.

**Limitations**

- Data in the registers are only available from 1969, so prevalent cases may be identified in the first time period of 1970 to 1984, which could have biased our findings toward overestimating incidence rates and have shifted the age distribution upwards.
- Not all of the diagnostic categories included in this study have been validated within the Danish registers (although several have).
- Under‐detection of mental disorder is likely as we only hold information on diagnoses made during hospital visits.

## 1 INTRODUCTION

Over a century ago studies were already estimating the incidence of mental disorders diagnosed in hospitals.^1^ Studies have highlighted apparent fluctuations in incidence over time^2^, ^3^, ^4^, ^5^; periods of increased incidence of different mental disorders have often been accompanied by both public and scientific concern.^6^ Some of these concerns have been driven by fear of unrecognized causal risk factors causing the increase, although it is now acknowledged that a combination of many diverse factors (including for example risk factors, factors related to detection, or factors related to health seeking) contribute to temporal changes in the reported cases of mental disorders over time.

While some changes in mental disorder incidence can be attributed to changes in risk factors, there are also several well‐known administrative factors influencing such time trends, including temporal changes in the number of hospital beds, treatment availability, registration of hospital admissions, and implementation of new clinical guidelines and diagnostic classifications. In Denmark, as in many other countries, there have been several such administrative changes in the last 50 years, which have influenced the incidence of diagnosed mental disorders.^4^, ^7^, ^8^ In addition, the incidence of mental disorders is also affected by non‐administrative factors, such as population demographics, cultural, and societal changes in how psychiatry and mental disorders are perceived, both by the general public and by healthcare professionals. Scientific discoveries made by some of the founders of modern psychiatry and pioneers in the classification of mental disorders (including Morel, Kraepelin, Bleuler, and Strømgren) have changed the concept of mental disorders, for example, the diagnostic concepts of schizophrenia have evolved over time,^9^ as have those for autism,^10^ and this also strongly influenced time trends in the incidence of diagnosed mental disorders.^11^, ^12^

A study that used nationwide administrative data on the number of persons treated for psychoses in a mental health hospital in Norway showed a stable cumulative incidence between 1926 and 1955, after which it increased until 1965.^2^ The author highlighted a range of factors that could have influenced the increase, including better access to psychiatric hospital beds and availability of new psychotropic drugs, but also stated that “public attitude changed, and hospitalization became more generally acceptable or even desirable.”^2^ More recent studies have described trends over time for selected mental disorders, including for schizophrenia,^3^ mood disorders,^5^ autism spectrum disorder,^8^ and other childhood psychiatric disorders.^4^, ^13^ However, long term, comprehensive studies of mental disorders as currently classified, which can improve our understanding of the reasons behind mental disorder diagnosis patterns, are lacking. Here, we aim to provide a broad overview of the temporal changes in the sex‐ and age‐specific incidence rates (IRs) of mental disorders diagnosed in Danish hospitals from 1970 until 2016. Using methods adapted from Hansen et al.,^8^ we also estimate whether such changes in incidence of mental disorders through five decades may be attributable to changes in administrative reporting practices. Specifically, we hypothesized that the addition of diagnoses made in outpatient appointments to the registers would increase the incidence of all disorders, while the impact of the shift from the use of the International Classification of Disease Eighth Revision (ICD‐8) to the Tenth Revision (ICD‐10) would differ depending on the disorder of interest. Additionally, we discuss other potential demographic, scientific, and cultural changes influencing the incidence profiles of mental disorders. The Danish national health registries offer exceptional possibilities to examine time trends in the nationwide incidence of the full spectrum of mental disorders diagnosed at hospitals, at the individual level, and classified according to the ICD. We expected IRs to have risen overall between 1970 and 2016, especially in younger age groups, considering an expansion of child and adolescent mental health services (CAMHS) has been observed in Denmark in recent years as well as the introduction in ICD‐10 of specific diagnostic criteria for select childhood‐onset conditions.^14^

### 1.1 Aims of the study

The aims of this study are to provide temporal changes in the sex‐ and age‐specific incidence rates of mental disorders diagnosed in Danish hospitals from 1970 until 2016 and investigate whether such changes may be attributable to changes in administrative reporting practice.

## 2 MATERIAL AND METHODS

### 2.1 Population

The study population included all persons aged 1–100 years living in Denmark at any point between January 1, 1970 and December 31, 2016. Each individual in the study was followed from their 1st birthday, immigration to Denmark, or January 1, 1970 (whichever happened last) until their 100th birthday, death, emigration from Denmark, or December 31, 2016 (whichever happened first). All demographic dates were obtained from the Danish Civil Registration System,^15^ which has maintained information on all residents since 1968, including sex, date of birth, continuously updated information on vital status, and a unique personal identification number that can be used to link information from various national registries.

### 2.2 Identification of mental disorders

Information on mental disorders was obtained from the Danish Psychiatric Central Research Register,^16^ which contains data on all admissions to psychiatric inpatient facilities since 1969 and in addition visits to outpatient psychiatric departments and emergency departments since 1995. The diagnostic system used was the Danish modification of the ICD‐8 from 1969 to 1993, and ICD‐10 from 1994 onwards. Specific disorders were defined using the ICD‐10 codes and corresponding ICD‐8 codes described in Table S1. For each individual in the study, the date of onset for each disorder was defined as the date of first contact with the psychiatric care system (inpatient, outpatient, or emergency visit) leading to a diagnosis of the disorder.

### 2.3 Statistical analysis

We estimated the IR of each mental disorder as the number of new cases diagnosed in hospitals divided by the total person‐years of follow‐up. IRs were estimated for males and females combined and separately, for all ages and for each specific 5‐year age group (1–4 years, 5–9 years, …, 90–94 years, 95–100 years), and for the entire period (1970–2016) and four specific calendar periods (1970–1984, 1985–1994, 1995–2004, and 2005–2016). These calendar periods were chosen to capture the two administrative changes in 1994/1995 (shift from ICD‐8 to ICD‐10 and inclusion of diagnoses made in outpatient clinics) and to allow adequate power in each calendar year group before and after the administrative changes.

Using the same methodology as Hansen et al.,^8^ we estimated the effect of the two administrative changes on the IR of mental disorders: the change in diagnostic criteria (i.e., shift from ICD‐8 to ICD‐10) in 1994 and the change to include diagnoses made in outpatient clinics in the register data in 1995 (only inpatient diagnoses were reported before 1995). For this, the two changes were modeled as time‐dependent covariates and we fitted a Cox proportional hazards regression model stratified on birth year, that is, assuming different baseline rates across birth years. Furthermore, the distribution of age‐at‐diagnosis was estimated for 2‐year periods from 1970 to 2016 and for males and females separately, including individuals with a first registered diagnosis of a specific disorder in the given period. Finally, IRs and age‐at‐diagnosis distributions for each mental disorder were also estimated using inpatient data only, to assess how the IR would have differed without the addition of outpatient data.

## 3 RESULTS

The study population included 9,107,157 persons (4,592,827 males and 4 514 330 females), who were followed up for a total of 233.0 million person‐years. During follow‐up, 43.7% were censored before the end of the study, due to death (*n* = 2,547,861) or emigration (*n* = 1,431,110). In the cohort, 9.5% (*n* = 866,524; 397,296 males and 469,228 females) were diagnosed with at least one mental disorder in a hospital during follow‐up. Across the total observation period, the IR for any mental disorder was 39.0 per 10,000 person‐years (36.0 in males and 41.9 in females). The disorder with the highest IR was anxiety disorder (15.8 per 10,000 person‐years), for both males (12.6) and females (18.9) (see Table S1).

The figures and text below present results for “any mental disorder,” however, figures showing results for specific types of mental disorders included in this analysis can be found in the Appendix S1 (Figures S1–S3).

### 3.1 Time trends in incidence

**FIGURE 1:**
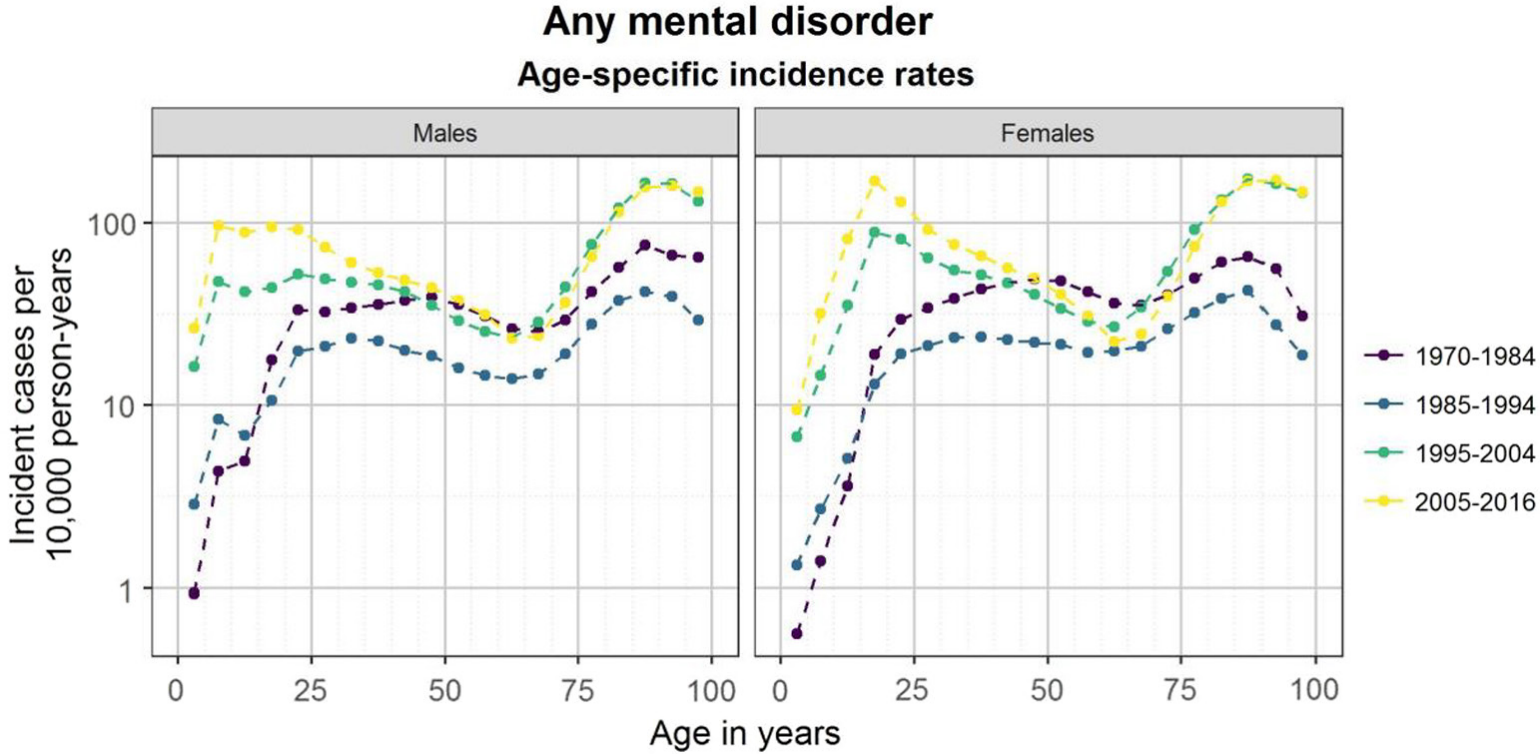
Age‐specific incidence rates of mental disorders from 1970 to 2016.

The IR of any mental disorder in the total study population decreased between 1970–84 (28.9 per 10,000) and 1985–94 (18.0 per 10,000) (Table 1). This trend was observed in both males (from 26.2 to 16.8 per 10,000; Table S2) and females (from 31.6 to 19.3 per 10,000; Table S3). This was also observed for most age groups, except those below age 15 years, for which there were small increases (Figure 1 and Table S4). In 1995–2004, the IR of any mental disorder was 47.2 for males and females combined, which increased to 63.0 in 2005–2016. IRs did not increase between these two periods for age groups between 60–64 and 90–95 years. Age‐specific IRs by calendar period can be found in Figure S1 for types of mental disorder.

**TABLE 1:** Number of cases and incidence rate per 10,000 person‐years of each mental disorder in Denmark including all contacts, in four time periods

Mental disorder	Number of cases				Incidence rate per 10,000 person‐years			
	1970–1984	1985–1994	1995–2004	2005–2016	1970–1984	1985–1994	1995–2004	2005–2016
Any mental disorder	209,128	85,212	221,400	350,784	28·88 (28·87–28·88)	18·04 (18·03–18·04)	47·17 (47·16–47·18)	63·02 (63·00–63·03)
Organic disorders	47,575	18,964	48,123	60,045	13.18 (13.17–13.18)	7.26 (7.26–7.27)	17.64 (17.64–17.65)	17.46 (17.45–17.47)
Alzheimer's disease	22,069	9456	14,889	25,969	6.09 (6.09–6.09)	3.60 (3.60–3.61)	5.42 (5.42–5.43)	7.50 (7.50–7.50)
Vascular dementia	9492	2922	9547	9387	2.62 (2.61–2.62)	1.11 (1.11–1.11)	3.47 (3.47–3.47)	2.70 (2.70–2.71)
Substance use disorder	61,079	25,857	39,829	51,117	9.55 (9.55–9.55)	5.96 (5.96–5.96)	9.31 (9.31–9.31)	9.71 (9.71–9.72)
Alcohol use disorder	47,026	21,144	30,100	31,526	7.34 (7.34–7.34)	4.86 (4.86–4.86)	7.01 (7.01–7.01)	5.96 (5.96–5.96)
Cannabis use disorder	2784	1994	5934	16,197	0.43 (0.43–0.43)	0.45 (0.45–0.45)	1.37 (1.37–1.37)	3.03 (3.03–3.03)
Schizophrenia spectrum disorder	36,462	21,354	24,856	34,450	5.69 (5.69–5.69)	4.91 (4.91–4.91)	5.79 (5.79–5.79)	6.51 (6.51–6.52)
Schizophrenia	12,840	6953	12,535	15,729	2.00 (2.00–2.00)	1.59 (1.59–1.59)	2.90 (2.90–2.90)	2.95 (2.95–2.96)
Schizoaffective disorders	3435	1983	2675	2,037	0.53 (0.53–0.53)	0.45 (0.45–0.45)	0.62 (0.62–0.62)	0.38 (0.38–0.38)
Mood disorders	67,657	22,995	70,142	121,267	10.60 (10.59–10.60)	5.31 (5.31–5.31)	16.44 (16.44–16.45)	23.32 (23.32–23.33)
Bipolar disorder	9874	5218	8677	15,464	1.54 (1.53–1.54)	1.19 (1.19–1.19)	2.00 (2.00–2.01)	2.90 (2.90–2.90)
Recurrent depression	17,781	8168	27,463	53,996	2.77 (2.77–2.77)	1.87 (1.87–1.87)	6.36 (6.36–6.36)	10.19 (10.19–10.19)
Single and recurrent depression	58,221	19,180	63,803	111,441	9.11 (9.11–9.11)	4.42 (4.42–4.42)	14.92 (14.91–14.92)	21.36 (21.36–21.37)
Anxiety disorder	56,351	23,394	89,169	173,501	8.10 (8.10–8.11)	5.07 (5.07–5.07)	19.47 (19.46–19.47)	31.38 (31.38–31.39)
Obsessive‐compulsive disorder	949	371	4012	14,525	0.14 (0.14–0.14)	0.08 (0.08–0.08)	0.86 (0.86–0.86)	2.53 (2.53–2.53)
Eating disorders	819	1061	7209	14,708	0.11 (0.11–0.11)	0.22 (0.22–0.22)	1.46 (1.46–1.46)	2.44 (2.44–2.44)
Anorexia nervosa	708	654	2042	4,387	0.10 (0.10–0.10)	0.13 (0.13–0.13)	0.41 (0.41–0.41)	0.73 (0.73–0.73)
Personality disorders	63,108	20,468	32,731	40,582	9.88 (9.87–9.88)	4.73 (4.73–4.73)	7.66 (7.66–7.66)	7.72 (7.72–7.72)
Personality disorders (borderline‐type)	1406	2470	6521	11,830	0.22 (0.22–0.22)	0.56 (0.56–0.56)	1.50 (1.50–1.50)	2.22 (2.21–2.22)
Antisocial personality disorder	11,984	1326	2191	2,019	1.86 (1.86–1.86)	0.30 (0.30–0.30)	0.51 (0.51–0.51)	0.38 (0.38–0.38)
Intellectual disability	1766	1410	7485	14,697	0.24 (0.24–0.24)	0.29 (0.29–0.29)	1.52 (1.52–1.52)	2.44 (2.43–2.44)
Developmental disorders	528	695	6081	27,364	0.07 (0.07–0.07)	0.14 (0.14–0.14)	1.23 (1.23–1.23)	4.54 (4.54–4.54)
Childhood autism	134	209	1605	8,056	0.02 (0.02–0.02)	0.04 (0.04–0.04)	0.32 (0.32–0.32)	1.33 (1.33–1.33)
Behavioral disorders	4792	3733	15,742	65,973	0.65 (0.65–0.65)	0.76 (0.76–0.76)	3.20 (3.19–3.20)	11.00 (11.00–11.01)
ADHD	769	571	4492	45,955	0.10 (0.10–0.10)	0.12 (0.12–0.12)	0.91 (0.91–0.91)	7.63 (7.63–7.63)

### 3.2 Time trends in age distribution

**FIGURE 2:**
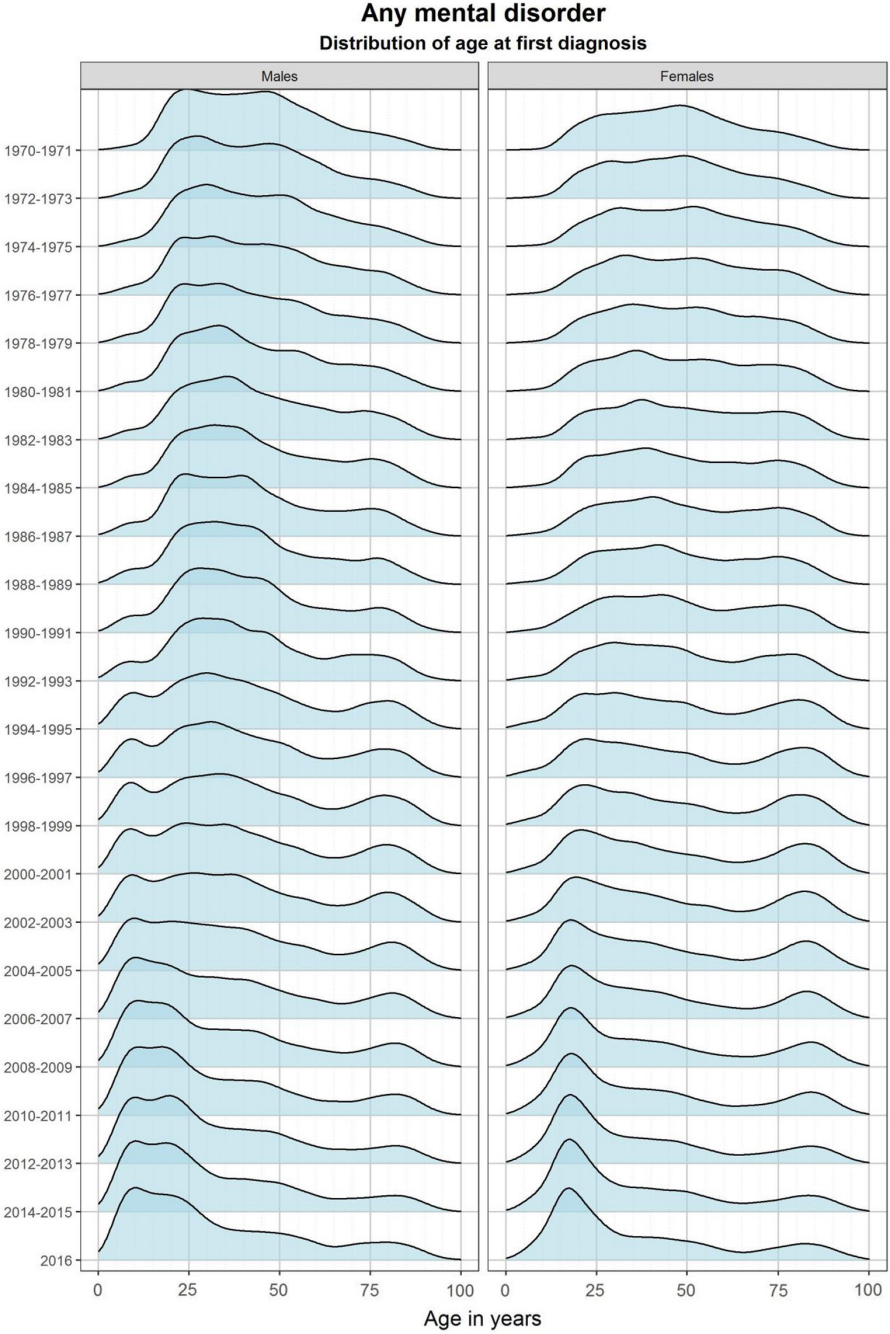
Changes in the distribution of age at first diagnosis of mental disorders from 1970 to 2016.

**FIGURE 3:**
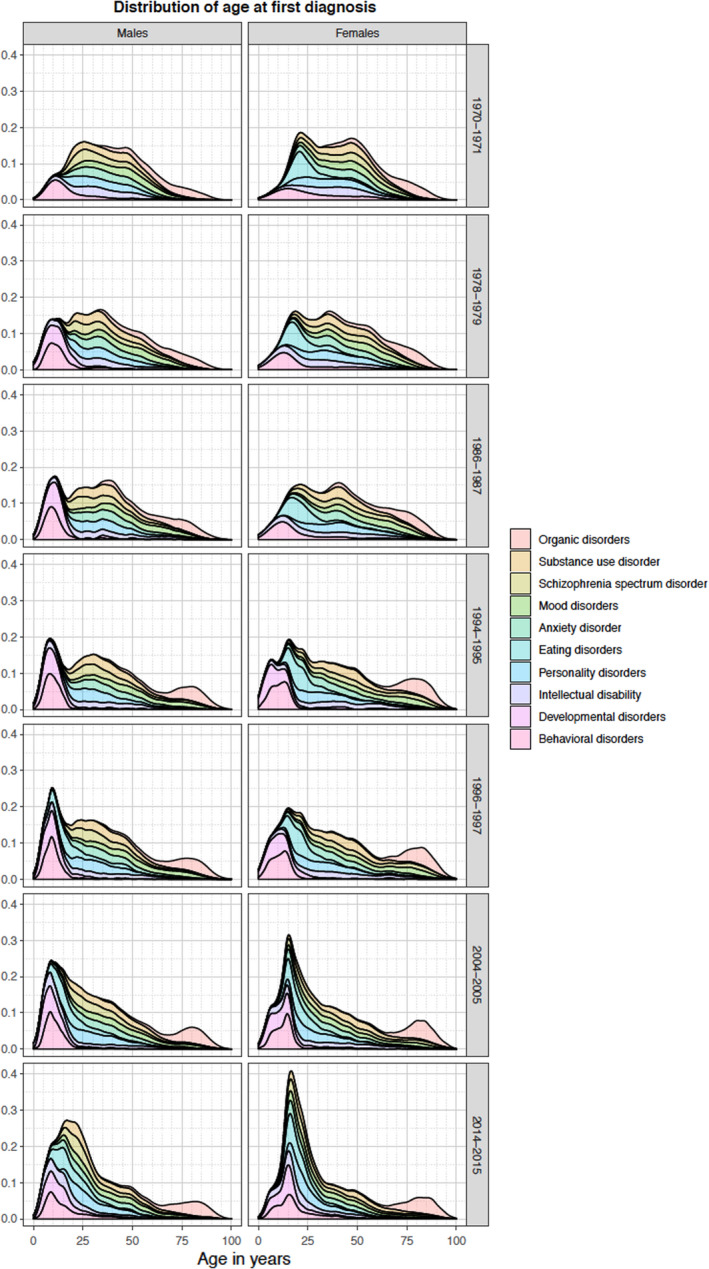
Time trends in the proportional age distribution of incident cases of groups of mental disorders, by sex and in seven periods of calendar years between 1970 and 2016.

In the 1970s and 1980s, the majority of persons diagnosed with any mental disorder were aged between 25 and 50 years at their first diagnosis (Figure 2). From the mid‐1990s, IRs for diagnosis between age 15 and 25 years increased, and from 2004 this became the most common age group to receive a first diagnosis of any mental disorder in both sexes. Throughout the study period, the IR also increased over time for those receiving their first diagnosis of a mental disorder above 75 years of age. Changes in the distribution of age at first diagnosis from 1970 to 2016 can be found in Figure S2 for types of mental disorder. The proportional age distribution of incident cases of specific mental disorders, by sex and in seven periods of calendar years between 1970 and 2016, are shown in Figure 3.

### 3.3 Time trends in inpatient admissions

The IR for inpatient admissions for any mental disorder decreased from 1970–84 to 1985–94 (Figure S3A). However, in 1995–2016, IRs increased slightly in both sexes in those under 25 years old; in those over 25 years, it remained largely unchanged in males, and decreased slightly in females. The IRs for inpatient admissions for the specific mental disorders are shown in Figure S3B–Z. All results for inpatients only can be found in Table S5.

### 3.4 The effect of administrative changes on the incidence of mental disorders

For any mental disorder and most of the specific mental disorders, the inclusion of outpatient data in the registers in 1995 explained part of the observed increase in IR (Table S6). There appeared to be less of an impact due to the change from ICD‐8 to ICD‐10 in 1994 (HRs less elevated in general), although for intellectual disability the ICD‐10 shift resulted in a fourfold increase in incidence rates. For some disorders, this had no apparent influence (e.g., schizophrenia and personality disorders). For some disorders (e.g., ADHD and personality disorders [borderline‐type]), the observed differences in the influence of the two changes in reporting practices differed somewhat between males and females (Table S6).

## 4 DISCUSSION

In this nationwide cohort study of the incidence of mental disorders diagnosed in hospitals covering almost five decades, several important time trends emerged. First, the IRs of most mental disorders decreased from 1970–84 to 1985–1994 (except eating disorders and NDDs). Second, compared to pre‐1995 levels, the IRs of all mental disorders increased from 1995 onwards; for some disorders, this was observed across most age groups (for example, bipolar disorder, depression, neurotic disorders, eating disorders, and NDDs), whereas for others it was restricted to particular age groups (SUD, schizophrenia, and personality disorders). Third, the majority diagnosed with a mental disorder in the 1970s and 1980s were between 25 and 50 years old at their first diagnosis in the registers. From the mid‐1990s onwards, an increasing number of individuals were diagnosed between age 15 and 25 years, and from 2004 the first diagnosis peaked in this age group for both sexes for most disorders. We would like to emphasize the utility of comparing estimates on age‐specific IRs and distribution of age‐at‐diagnosis simultaneously. While the former compared new cases among the general population, the latter is only based on those diagnosed. Consequently, demographic changes in the source population might also impact the distribution of age‐at‐diagnosis.

We found that some of the changes in IR coincided with known changes in diagnostic and reporting practices, which are likely to have contributed to these time trends. First, the decrease in IRs of mental disorder diagnoses in hospitals in 1970–1994 could reflect political reform in 1970 in Denmark^17^ that resulted in a dramatic decrease in psychiatric inpatient beds,^3^, ^18^ which continued until the 1990s.^7^, ^14^ This may also partly explain the decrease in IR observed for some specific disorders, for example, schizophrenia spectrum disorder, SUD, and bipolar disorder. Similar scenarios occurred in several other countries at this time.^19^

Second, the inclusion of data on outpatient and emergency ward visits in the Danish registers from 1995 could have contributed to the increased IR observed in the registers after 1995. However, such increased IRs were also observed in other countries during the 1990s, suggesting that other factors were also important.^20^, ^21^ Inclusion of outpatient contacts is also likely to explain some of increased IRs for specific disorders reported in the Appendix S1, like schizophrenia spectrum disorder; however, the post‐1994 increase was mainly in those below 25 years old, and this shift toward younger diagnosis started decades earlier (with the IR increasing between 1957 and 1972 in that age group, despite an overall decrease). NDD IRs increased across all age groups after 1994; this was also seen for development and behavioral disorders, and ADHD. Although the inclusion of outpatient data appears to explain part of the increase, the IR for NDDs pre‐1995 was almost negligible and the increase occurred from around 2000; so although outpatient clinics resources continued to increase,^7^ this cannot be the not the only explanation for the increase in NDDs.

Third, CAMHS have expanded over the last two decades in Denmark. In 2001, 0.4% of individuals under 18 years of age were in contact with a child and adolescent psychiatric department, but by 2018 it was 3.3%.^14^ This expansion has been driven largely by developments in evidence‐based medicine, the publication of clinical guidelines specific to children and adolescents, and the 2008 introduction of a two‐month maximum waiting time for psychiatric evaluation for this age group.^7^ Additionally, more standardized screening and diagnostic tools for childhood conditions have become available. This CAMHS expansion is not unique to Denmark^22^ and coincided with a large increase in incidence of diagnosed NDDs worldwide.^4^, ^8^, ^22^, ^23^, ^24^ The expansion may also explain the downward shift in age at first diagnosis observed after 2000 for bipolar disorder, depression, anxiety disorders, personality disorders (in females), schizophrenia spectrum disorders, and SUD, in agreement with findings from previous studies.^25^

Fourth, parallel to administrative changes, there have been cultural and societal changes in how psychiatry in general is perceived and mental disorders in children and adolescents in particular. These have likely contributed to some of the observed time trends. For instance, the late increase observed for the IRs of NDDs in 2005 for females (compared with that seen already in 1995 for males) may reflect a movement away from a stereotypical perception of childhood autism and ADHD as being disorders found *only* in boys, as also seen in other countries.^24^ Similarly, we observed broadening of the age range at first diagnosis for these two disorders. In 1970–1994, they were typically only diagnosed in preschool and school‐age children; however, in later years, these diagnoses were also made for young adults (1995–2004) and older adults (2005–2016). There has been slow acknowledgment that adults can be newly diagnosed as having NDDs, with development of adult‐specific criteria for them, although these disorders by definition have childhood onset.

### 4.1 Comparisons to prior studies

Pedersen et al. have previously described IRs of mental disorders in Denmark. While our IRs are not directly comparable (i.e., as overall IRs were considered) some similar patterns emerge.^26^ We observed an decreased IR for inpatient admissions for schizophrenia between 1970 and 1987 and this concurs with a previous study.^3^ An earlier Danish study reported increased incidence of borderline personality disorder since 1970, especially in younger age groups, and mostly in females, attributing to the change in diagnostic classification from ICD‐8 to ICD‐10 in 1994, and temporal variations in etiology and/or changes in diagnostic habits.^27^ Our findings support that a large part of the increase in IR for borderline personality disorder from 1970 to 2016 was indeed explained by the 70% increase in IR from ICD‐8 to ICD‐10 in 1994 (HR 1.7 [95% CI 1.4–2.1]).

The incidence of NDDs has also been studied extensively using Danish data, with similar findings to ours.^4^, ^8^, ^13^, ^23^ However, our study offers additional knowledge regarding time trends in the incidence of NDDs, by covering a longer time period, estimating sex‐specific rates and providing details on the temporal changes in the age distribution at first diagnosis.

### 4.2 Strengths and limitations

Our study uses nationwide individual‐level data on incident clinical diagnoses based on the entire population; selection bias is thus unlikely. It provides a comprehensive overview of IR trends for all mental disorders, by sex and age. Furthermore, our study has a long observation period, spanning almost five decades, and we had accurate data on censoring due to death and migration. Additionally, we have been able to apply a quantitative approach to assess impact of selected administrative factors on reported incidence.

However, our study has several important limitations. First, as data in the registers are only available from 1969, the first time period from 1970 to 1984 may not include only true incident cases, but also prevalent cases with prior (unregistered) admissions due to mental disorders. This will be relevant for the older age groups, as these individuals have had more time at risk of a first diagnosis before 1969. This will have biased our findings toward overestimating IRs and have shifted the age distribution upwards in those patient groups (i.e., older age groups during 1970–1984). Considering data only from 2005 onwards minimizes the potential bias introduced.^26^ Second, although some diagnoses have been validated in Danish registers (e.g., mood disorder, ADHD, and childhood autism),^28^, ^29^, ^30^ not all of the diagnostic categories included in this study have been validated. Third, under‐detection of mental disorder is likely. We only hold information on diagnoses made during hospital visits; prior to 1995, the data only relate to inpatient visits. Some mental disorders may be managed in primary care (and a small proportion in private clinics in Denmark), and some people will not seek medical advice for conditions. We are unable to investigate changes in the true underlying incidence of the disorders, but merely in the diagnoses of the disorder, and thus cannot comment on whether part of the observed increase maybe due to actual increases in mental illnesses. Finally, our evaluation of potential explanations for time trends is not exhaustive, and hence, caution in the interpretation of these associations should be exerted.

To conclude, our nationwide study of mental disorder incidence since 1970 yielded evidence for an overall increase in mental disorder IRs and downward shift in age of diagnosis over this period. The study results are consistent with previous findings and provide further details of factors underlying incidence patterns. Specifically, administrative changes in the Danish healthcare system over the study period appeared to influence some of the trends observed; however, they may have also been driven by demographic changes in the population (e.g., shift in age distribution), and public and professional perception of psychiatry and mental disorders. Many previous studies have contributed with evidence; however, the present study is, to our knowledge, the first to describe time trends in IRs for a broad spectrum of mental disorders and to estimate the influence of administrative changes in the healthcare system. The study results may both provide valuable baseline scientific and historical context of the epidemiology of mental disorders for comparison with other studies and populations, and fuel hypothesis‐driven investigations of the epidemiologic architecture of mental disorders.

## CONFLICT OF INTEREST

The authors declare no conflicts of interest.

## AUTHOR CONTRIBUTIONS

OP‐R and SD conceptualized the study. OP‐R designed the study and did the data analysis. OP‐R, NCM, and SD wrote the first draft of the manuscript. All co‐authors interpreted the results, revised the manuscript critically, and accepted the final version for publication.

### PEER REVIEW

The peer review history for this article is available at https://publons.com/publon/10.1111/acps.13410.

## Supporting information

Appendix S1.

## Data Availability

Access to individual‐level Denmark data is governed by Danish authorities. These include the Danish Data Protection Agency, the Danish Health Data Authority, the Ethical Committee, and Statistics Denmark. Each scientific project must be approved before initiation, and approval is granted to a specific Danish research institution. Researchers at Danish research institutions may obtain the relevant approval and data. International researchers may gain data access if governed by a Danish research institution having needed approval and data access.
