# Supplementary material for: Temporal changes in sex‐ and age‐specific incidence profiles of mental disorders—A nationwide study from 1970 to 2016
Source: Acta Psychiatr Scand. 2022 Feb 18;145(6):604–14. doi: 10.1111/acps.13410 (PMC9305516; doi:10.1111/acps.13410)
Supplement: Supplementary file 1 — Appendix S1. [file ACPS-145-604-s001.pdf]

Supplementary File 1

Temporal changes in sex- and age-specific incidence profiles of mental disorders – a nationwide study from 1970 to 2016

eFigures 1A-1Y. Age-specific incidence rates, by calendar period

Note that the vertical scale is logarithmic and scaled differently for each disorder, depending on the number of cases.

eFigure 1A.

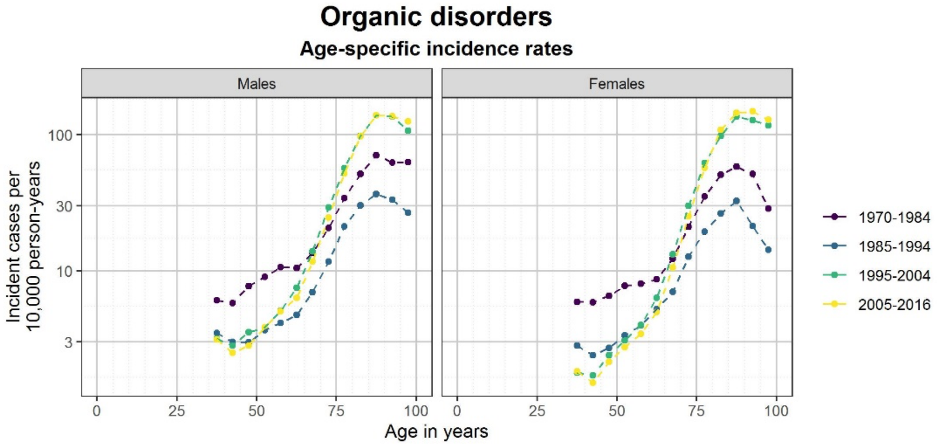

eFigure 1B.

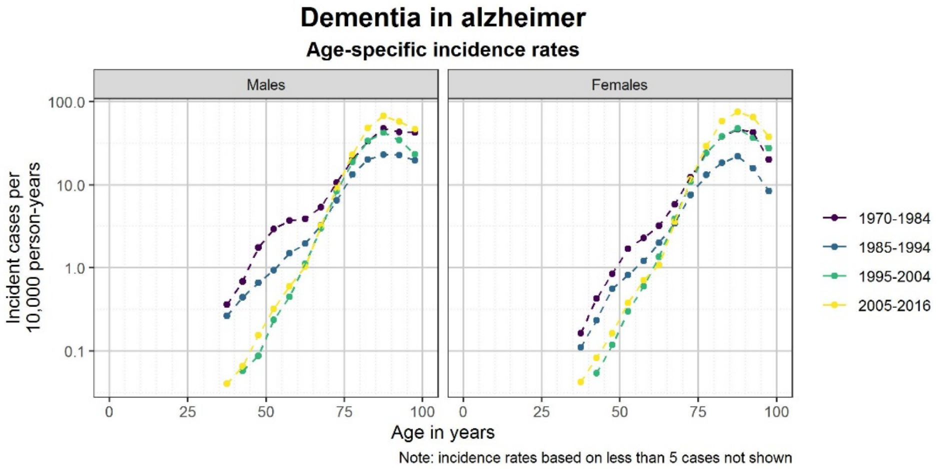

eFigure 1C.

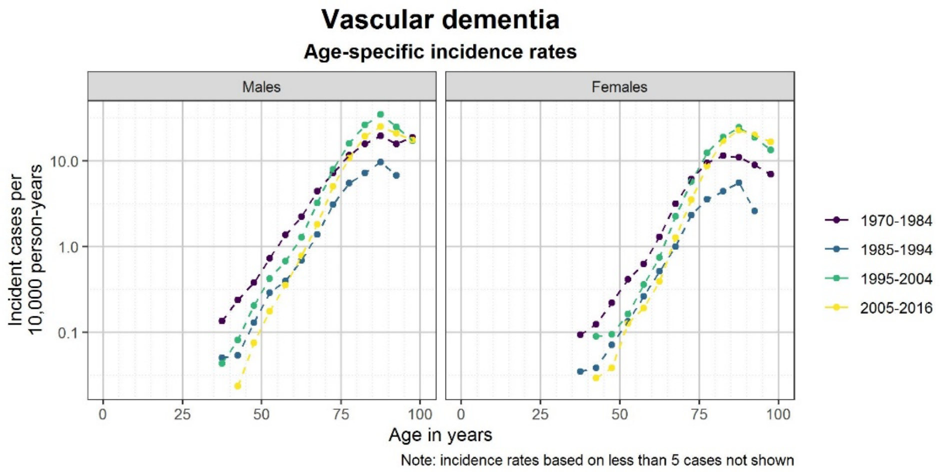

eFigure 1D.

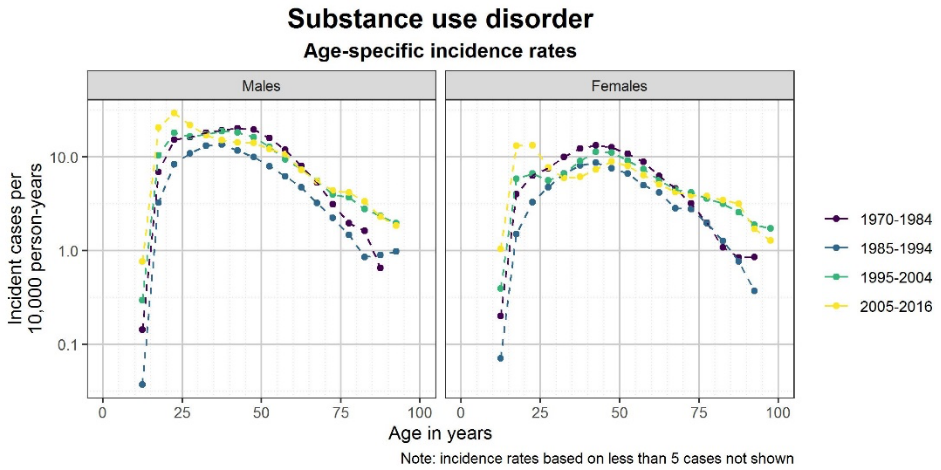

eFigure 1E.

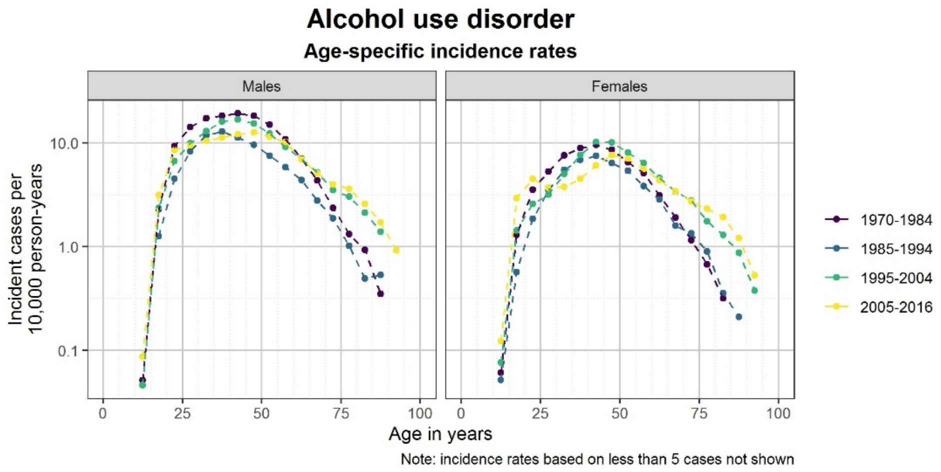

eFigure 1F.

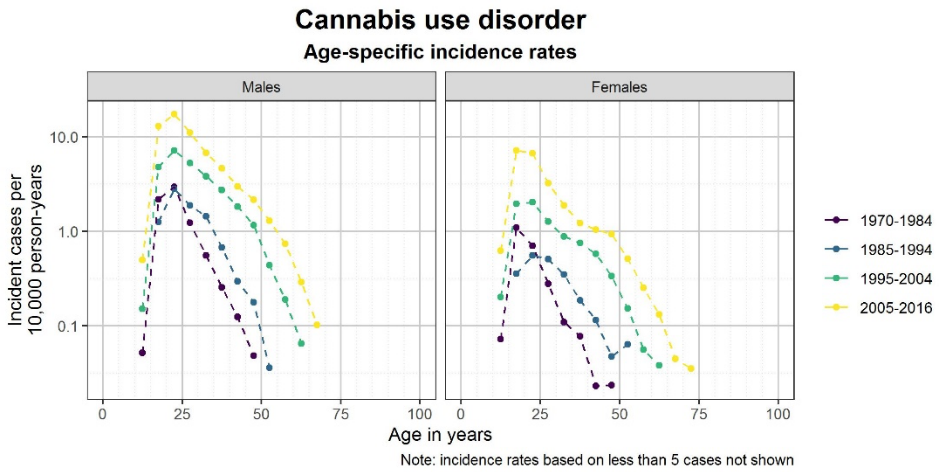

eFigure 1G.

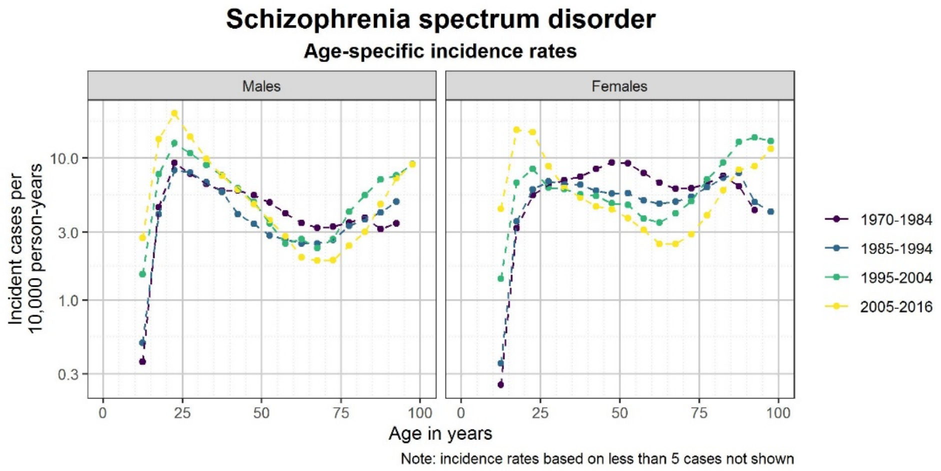

eFigure 1H.

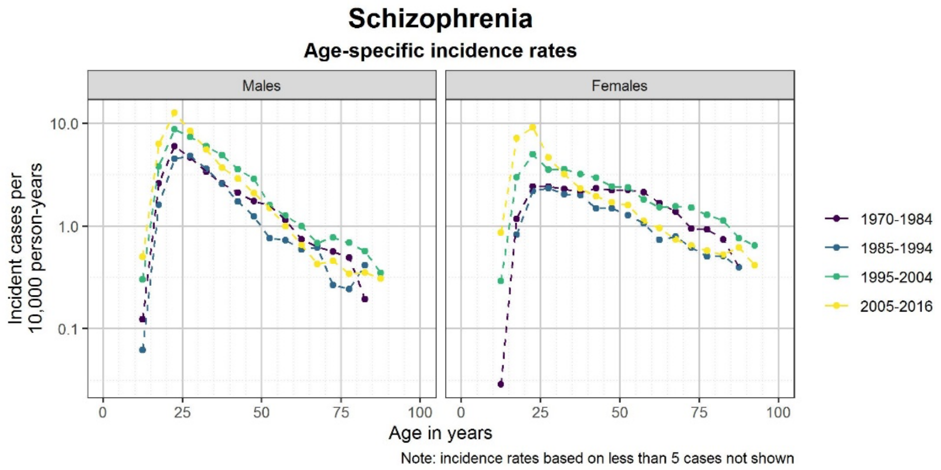

eFigure 1I.

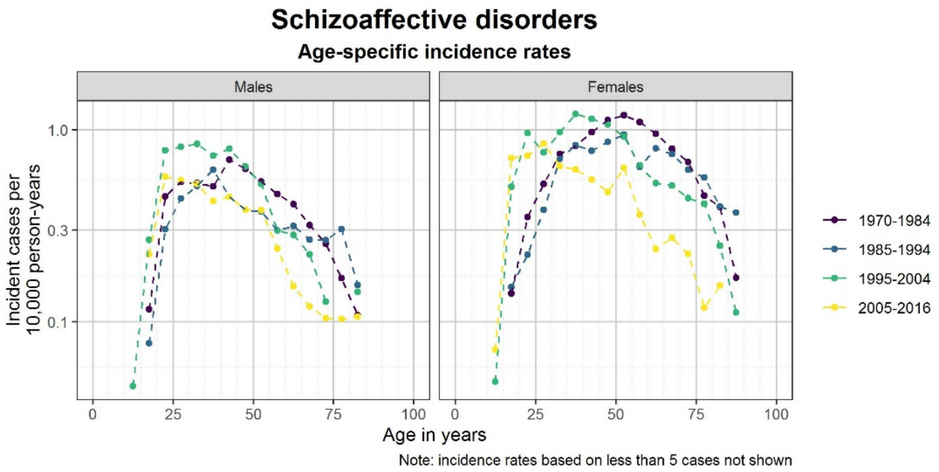

eFigure 1J.

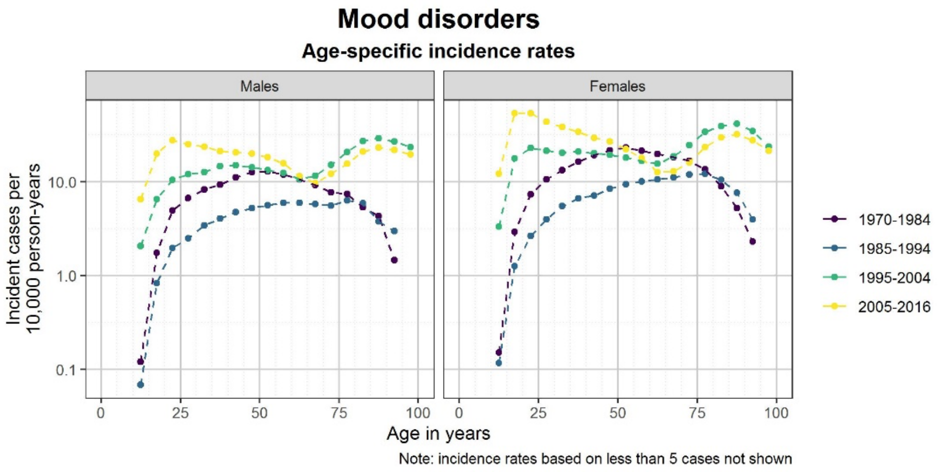

eFigure 1K.

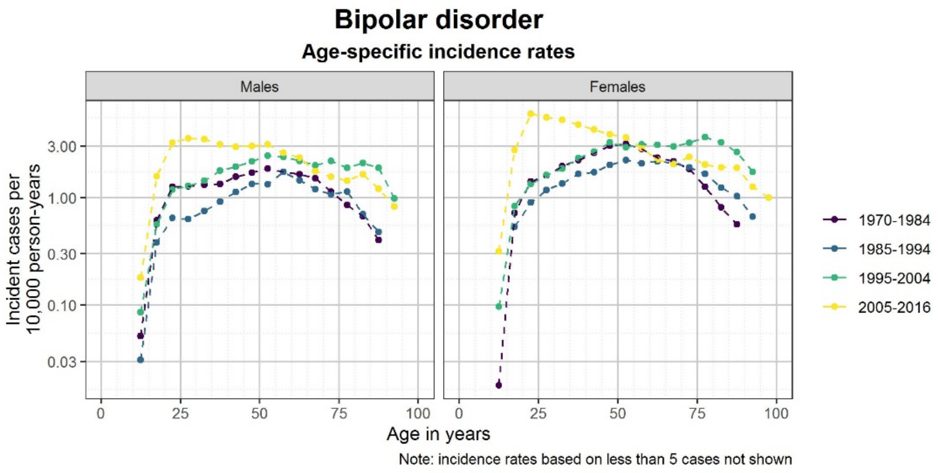

eFigure 1L.

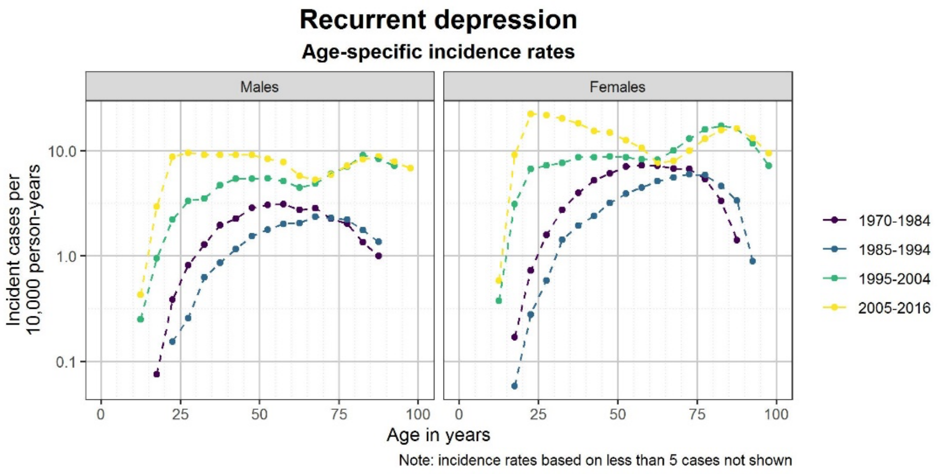

eFigure 1M.

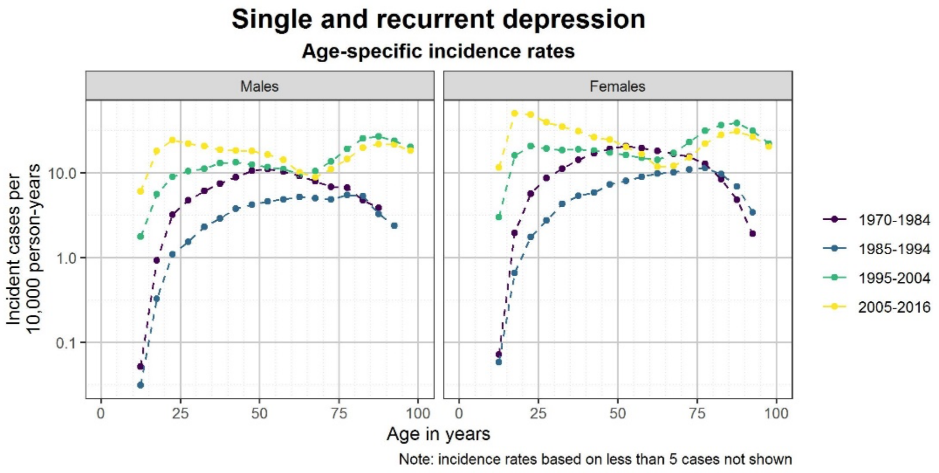

eFigure 1N.

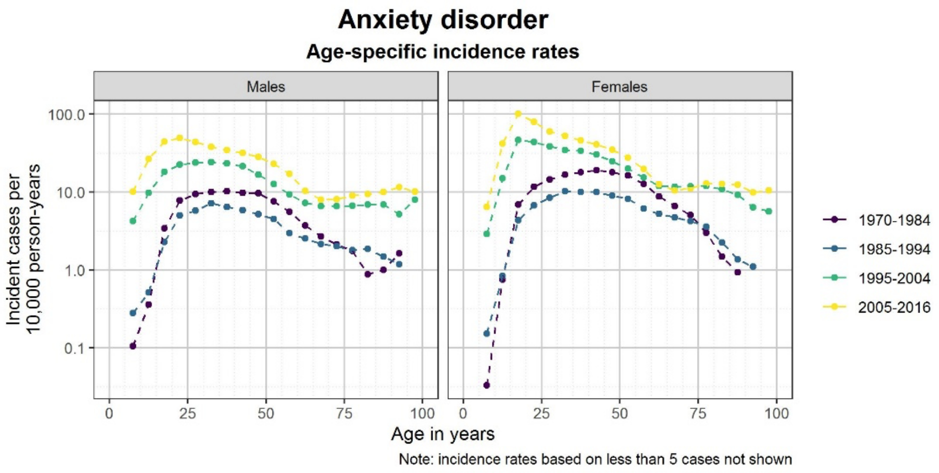

eFigure 1O.

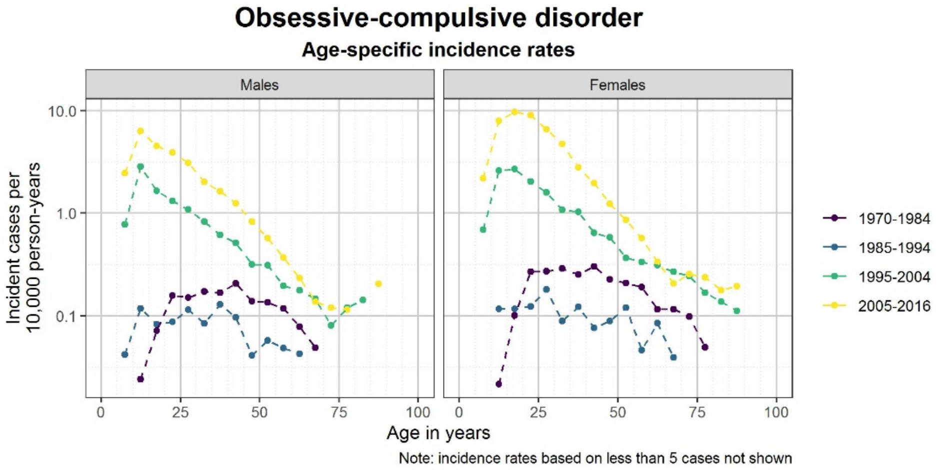

eFigure 1P.

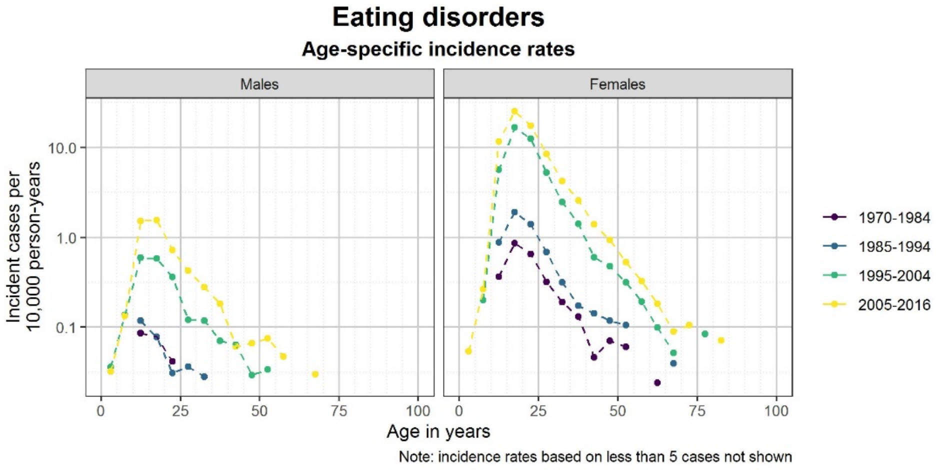

eFigure 1Q.

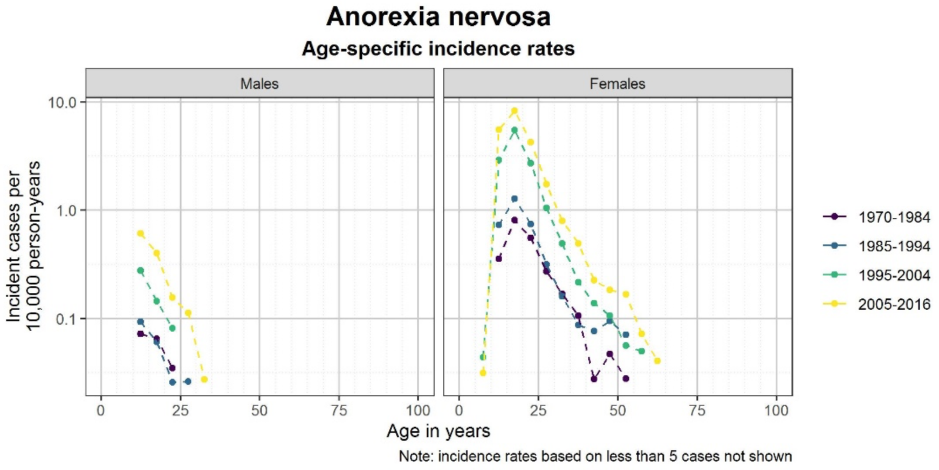

eFigure 1R.

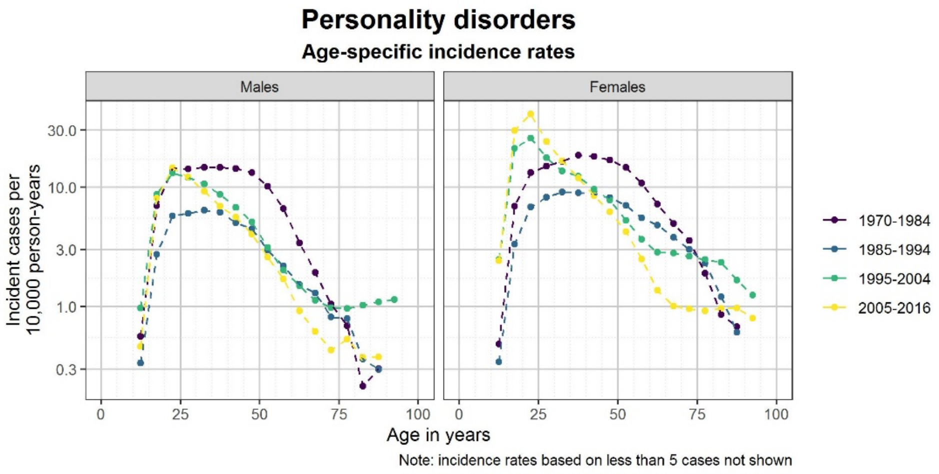

eFigure 1S.

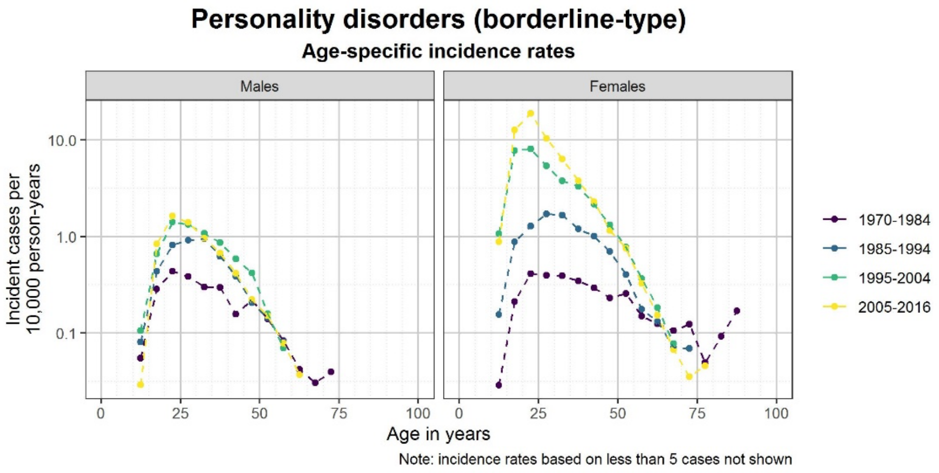

eFigure 1T.

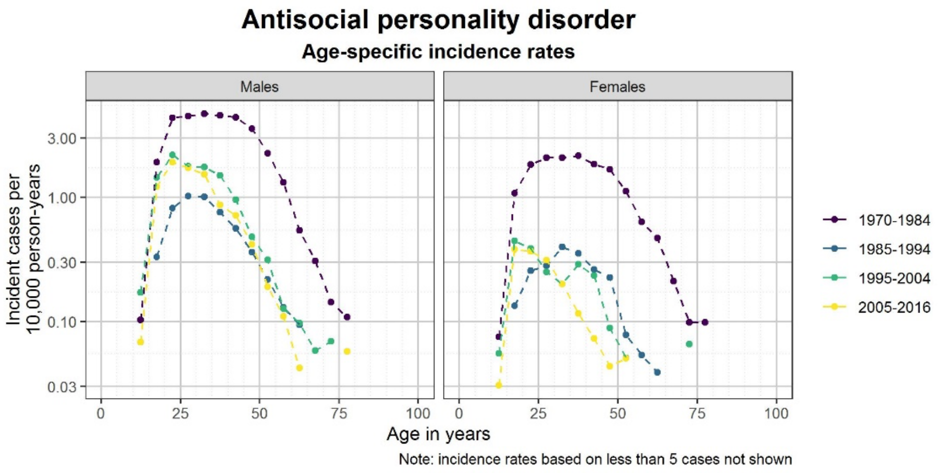

eFigure 1U.

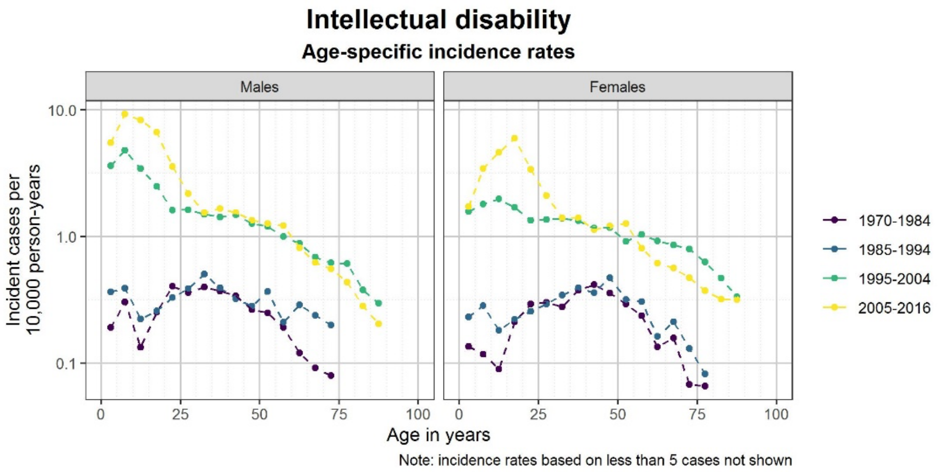

eFigure 1V.

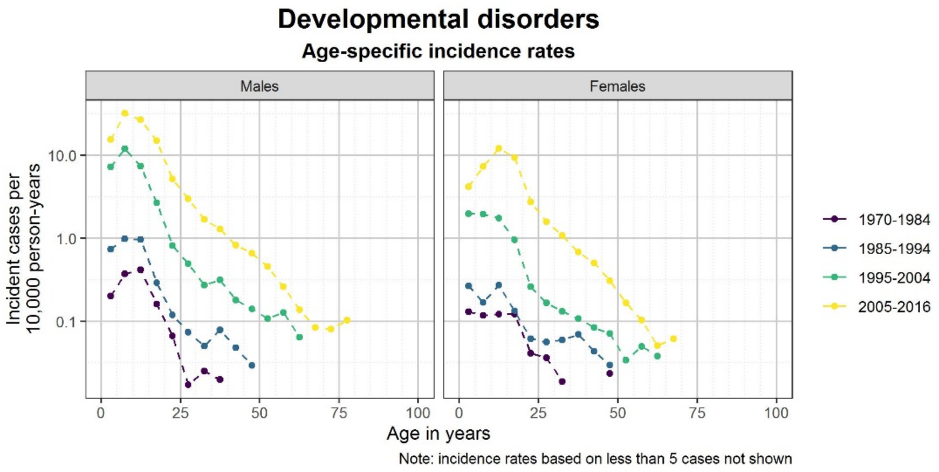

eFigure 1W.

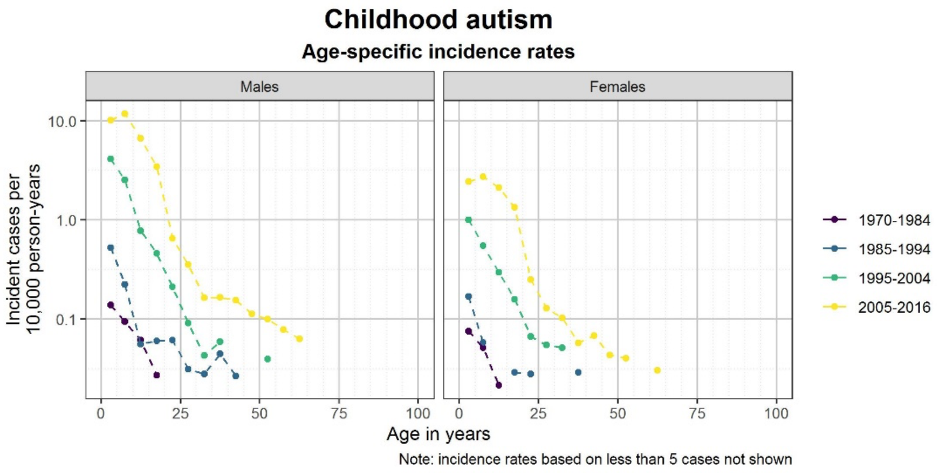

eFigure 1X.

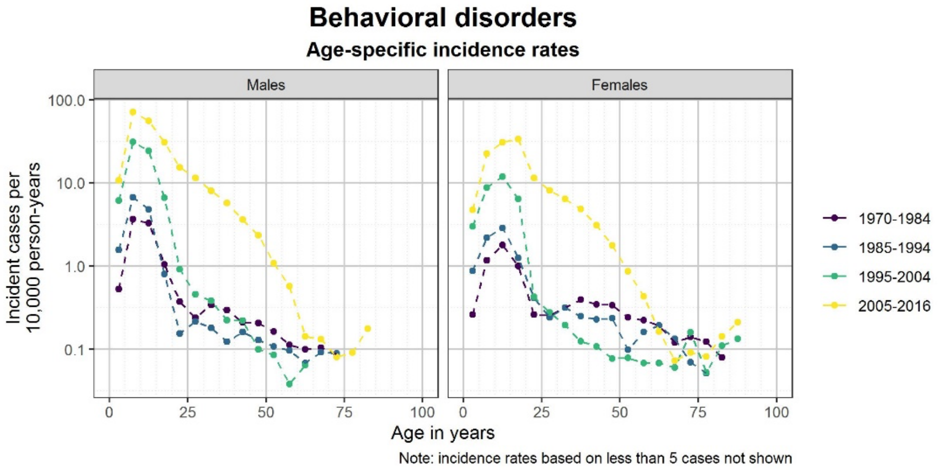

eFigure 1Y.

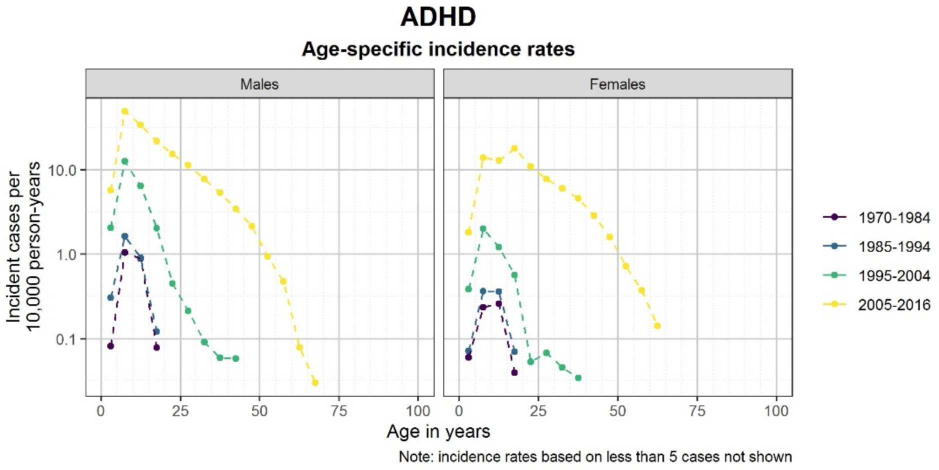

eFigures 2A-2Y. Changes in the distribution of age at first diagnosis of mental disorders from 1970 to 2016

Each curve represents the proportion of individuals receiving their first diagnosis of the mental disorder in question at different age, during specific time periods (e.g. 1970-1971). Note, that the vertical scale for the curves is percentage and that the area under each curve sums to 100%. Importantly, changes in incidence rates from one time period to the next are not shown in this figure.

eFigure 2A.

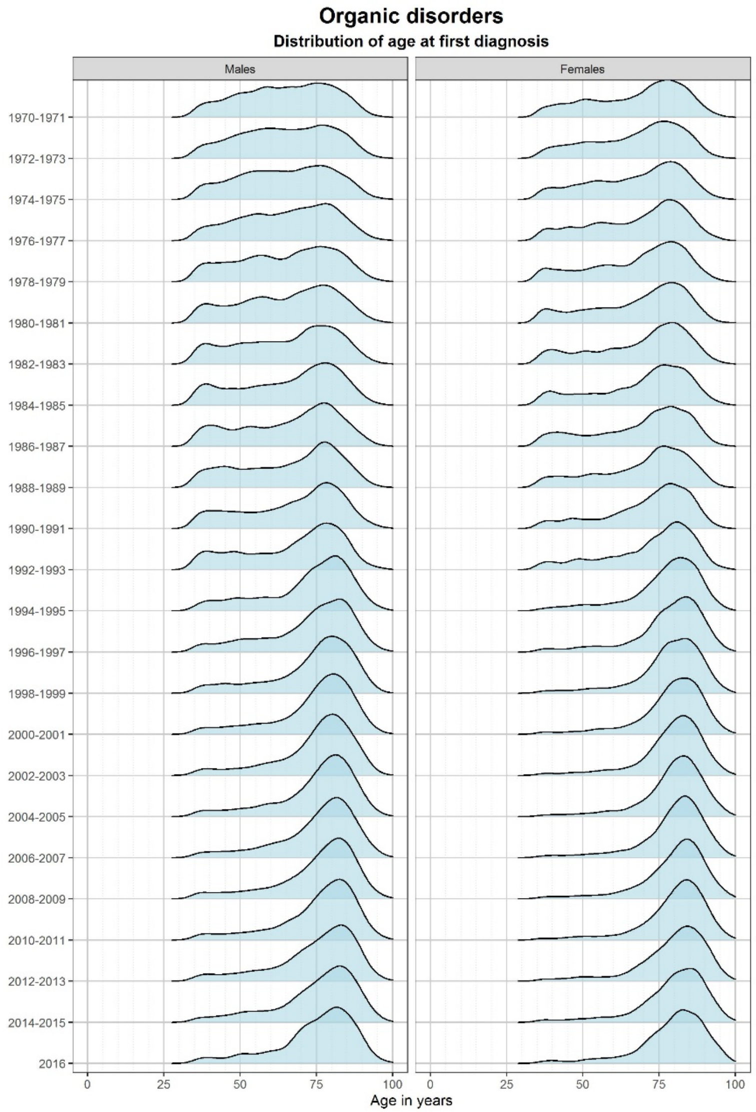

eFigure 2B.

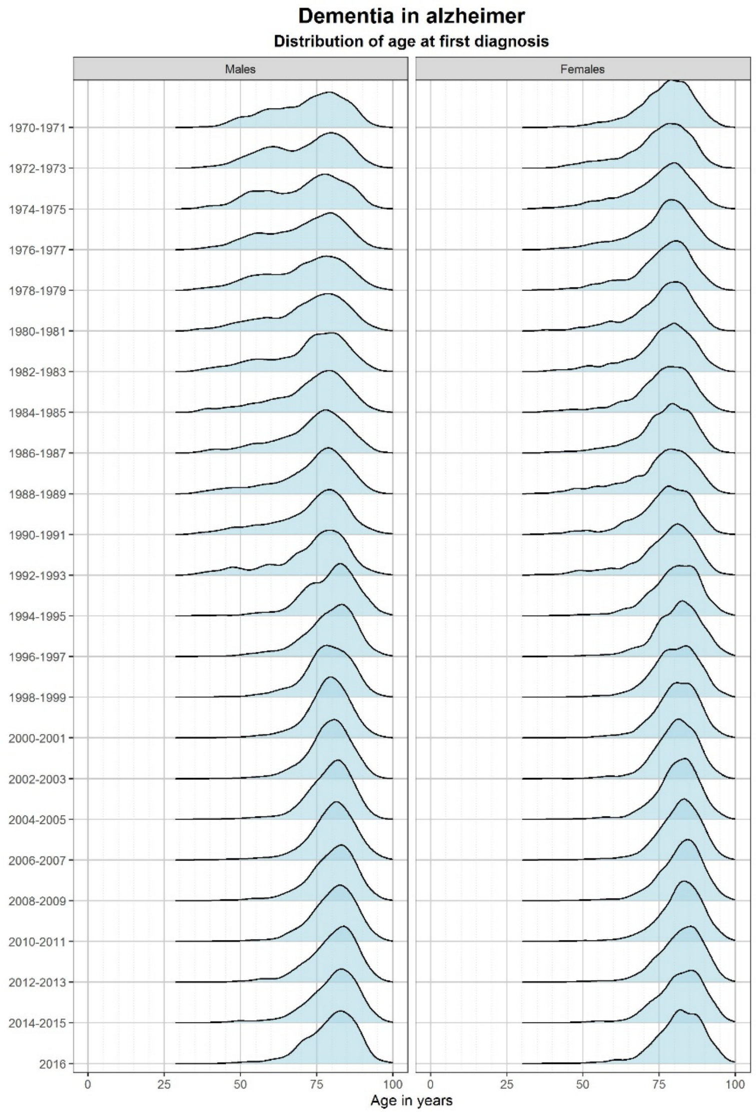

eFigure 2C.

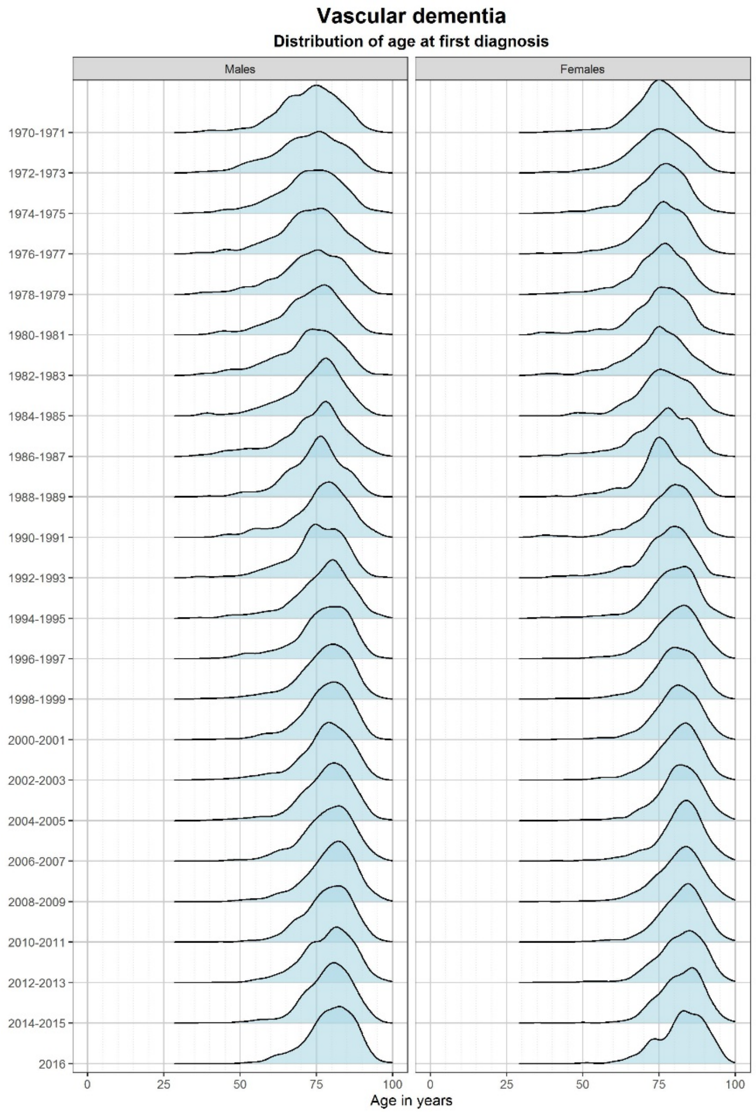

eFigure 2D.

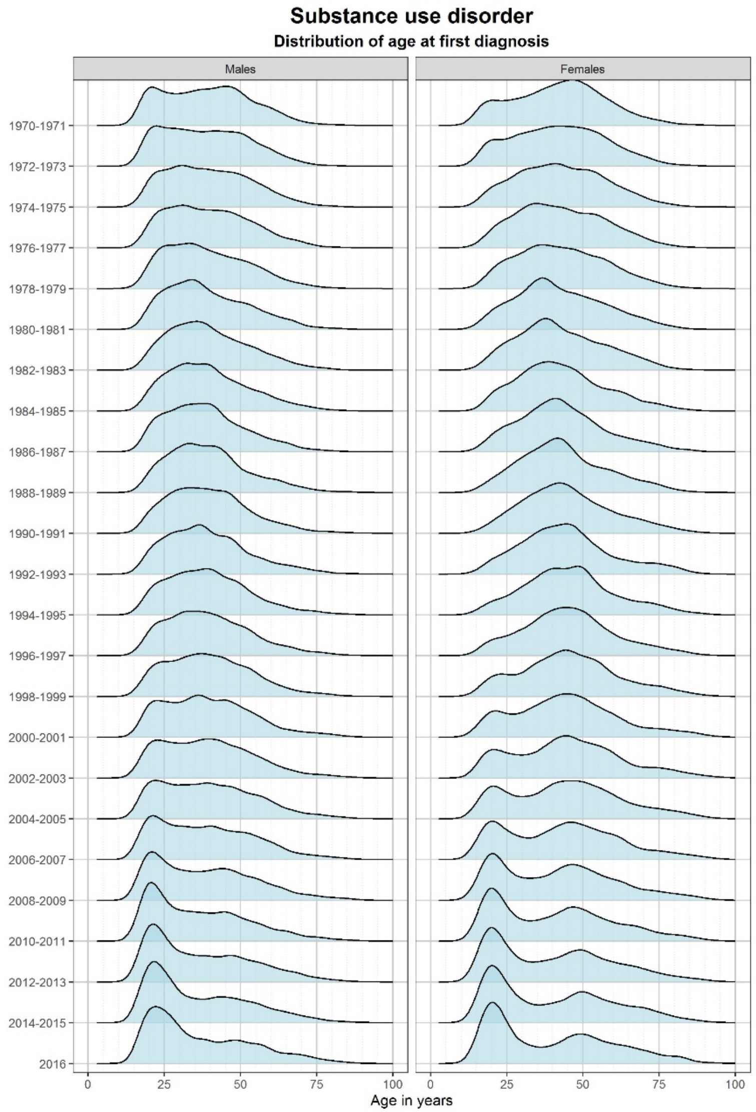

eFigure 2E.

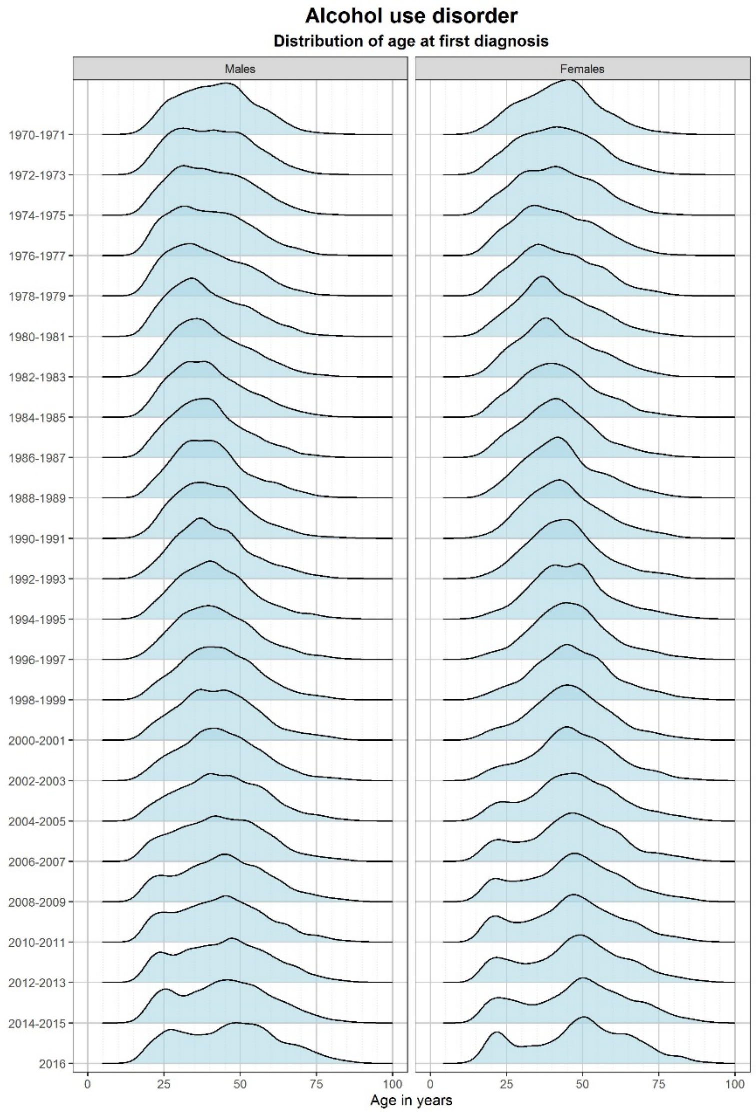

eFigure 2F.

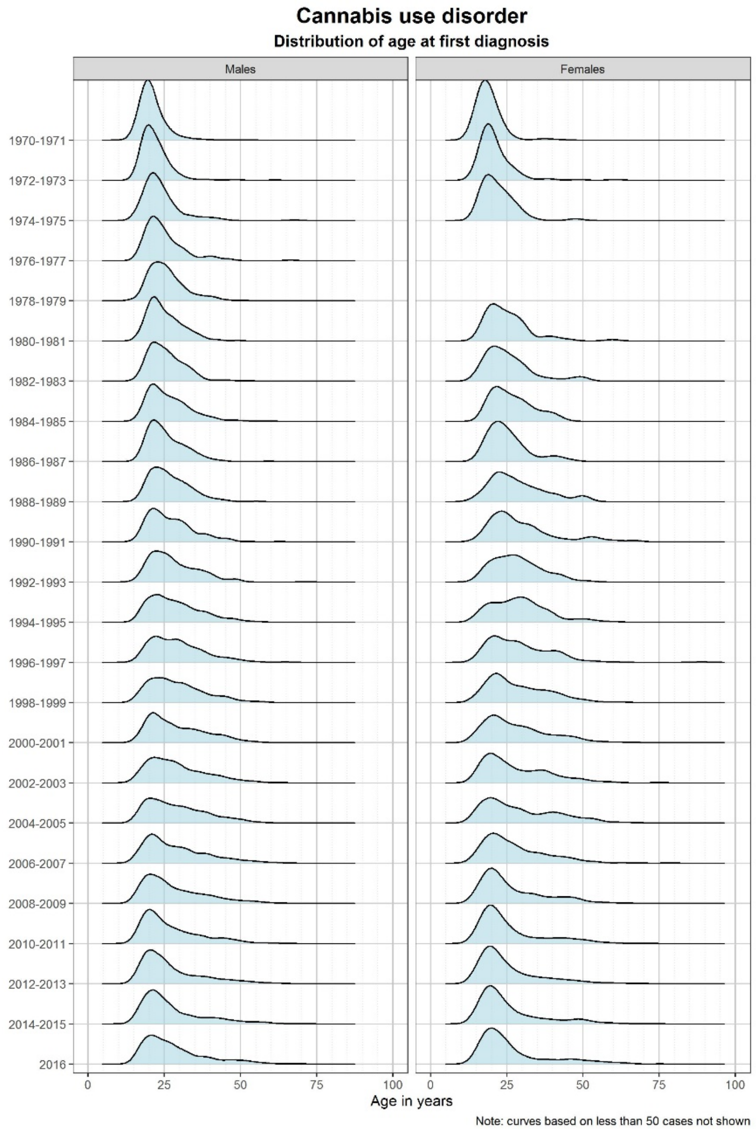

eFigure 2G.

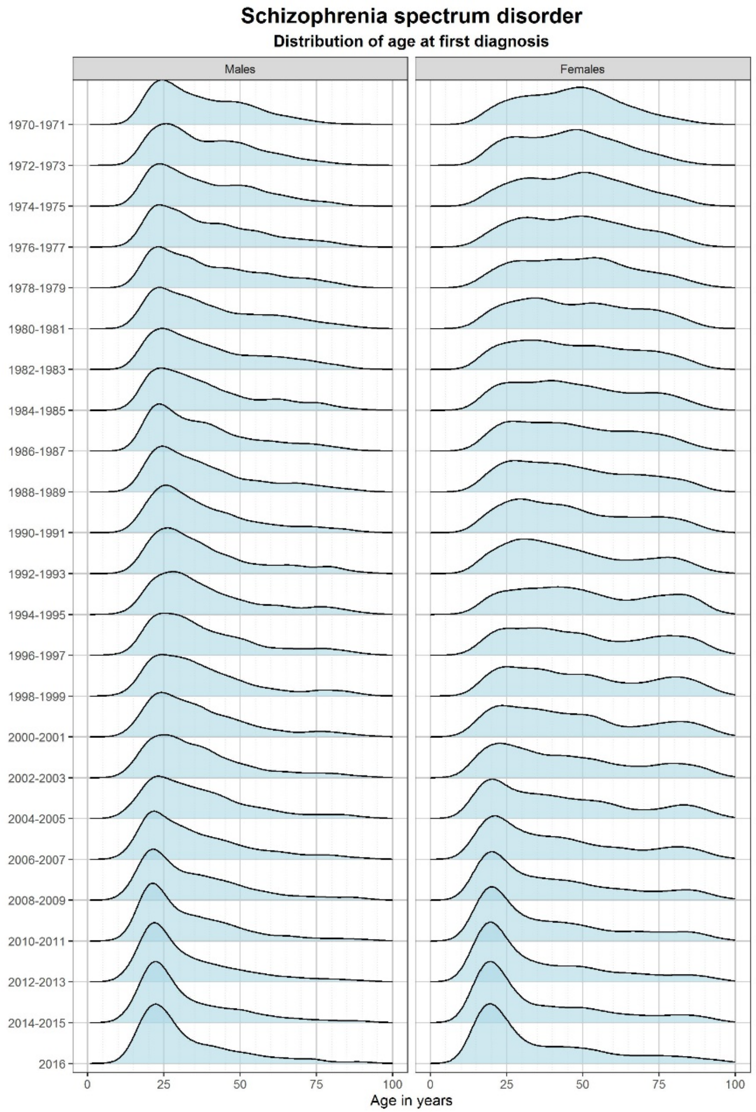

eFigure 2H.

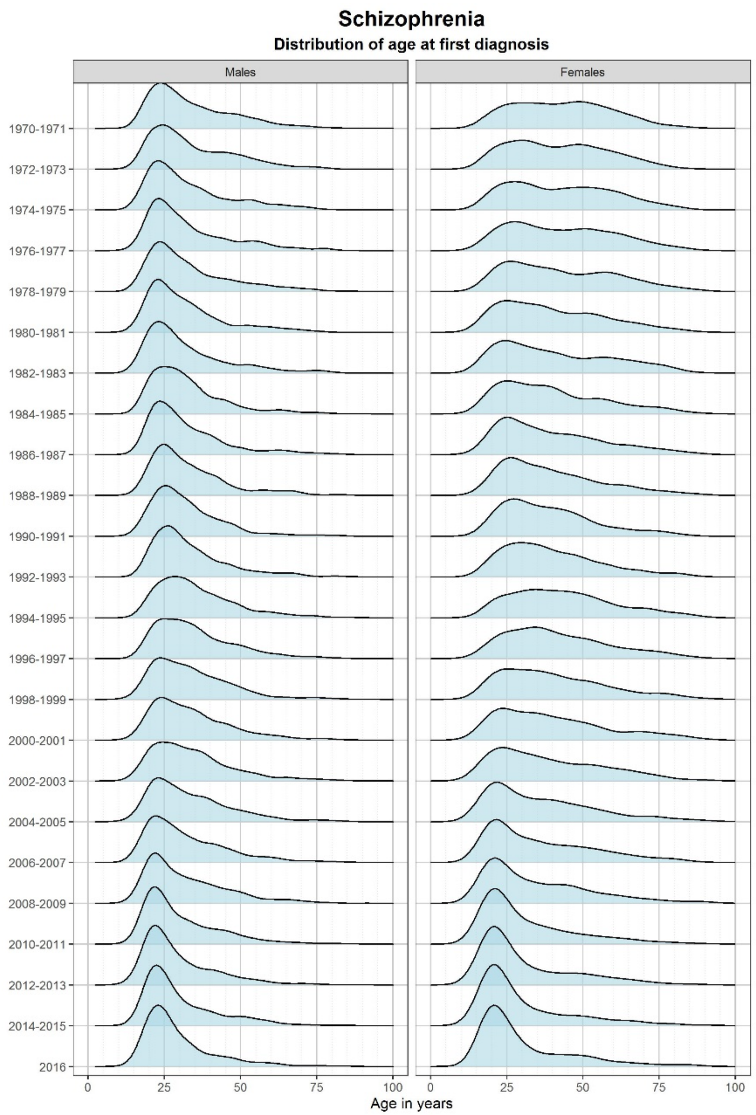

eFigure 2I.

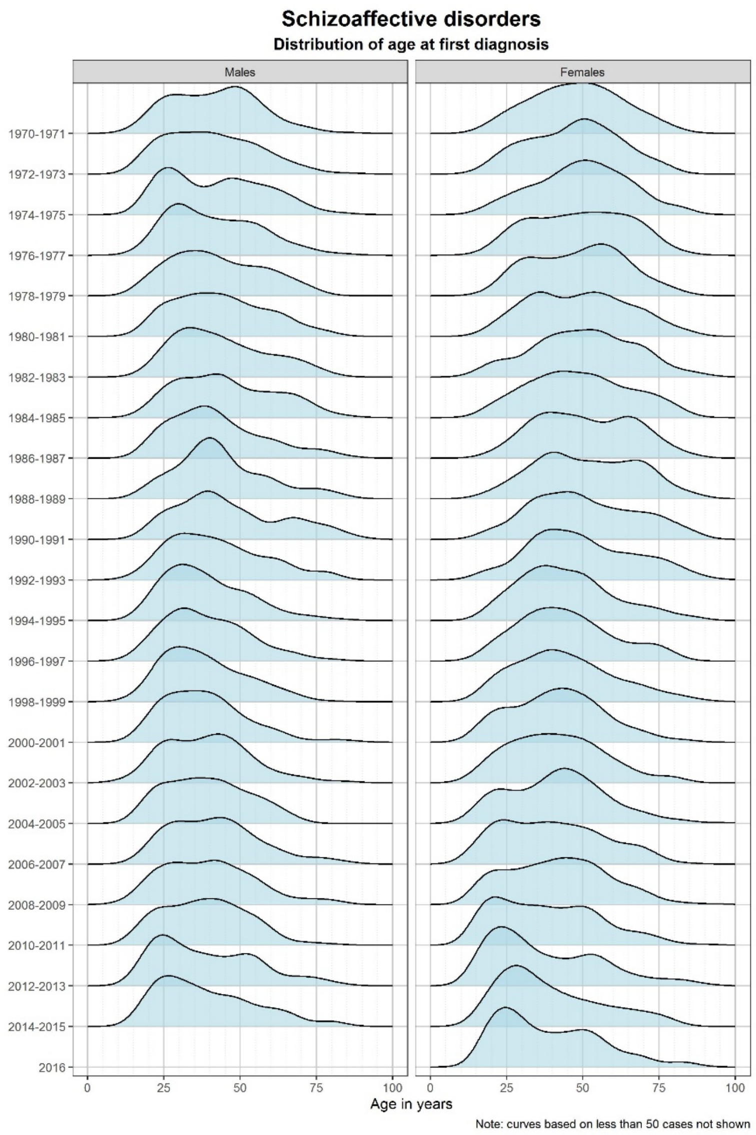

eFigure 2J.

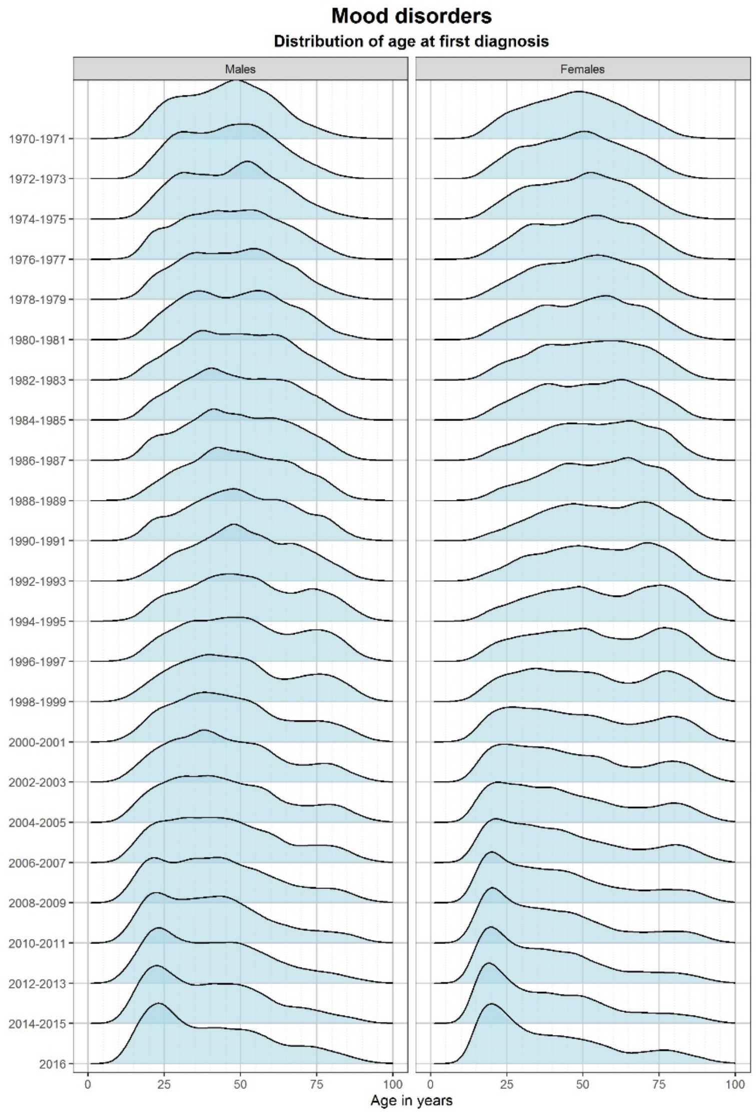

eFigure 2K.

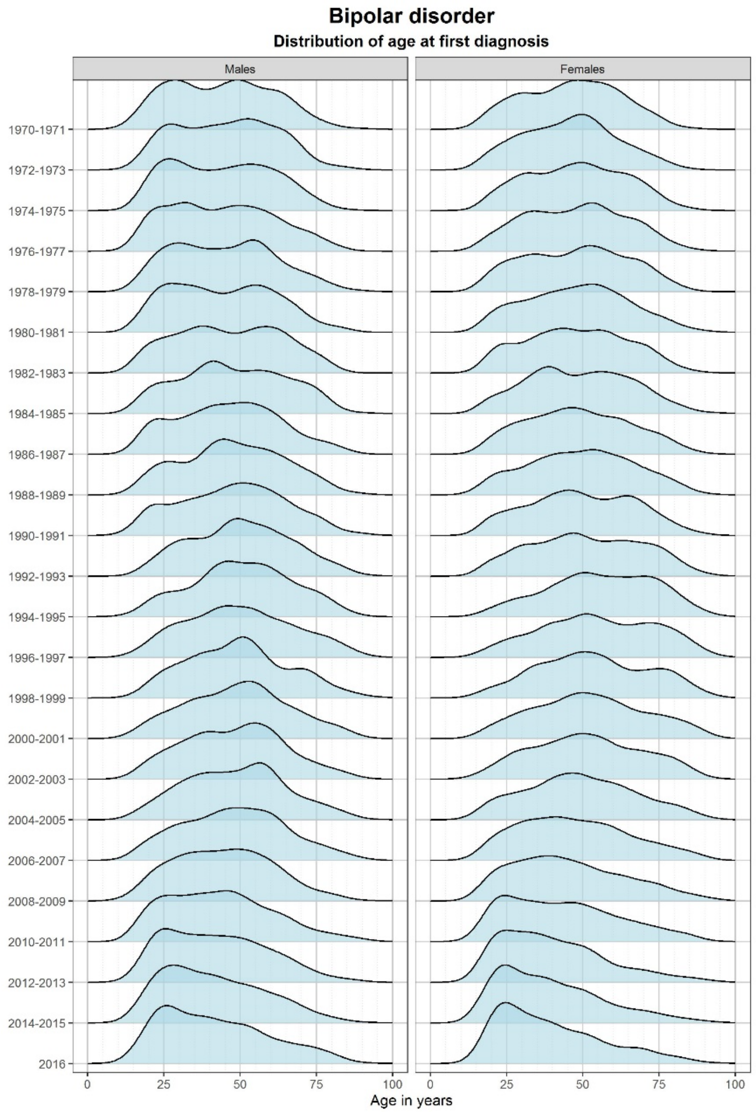

eFigure 2L.

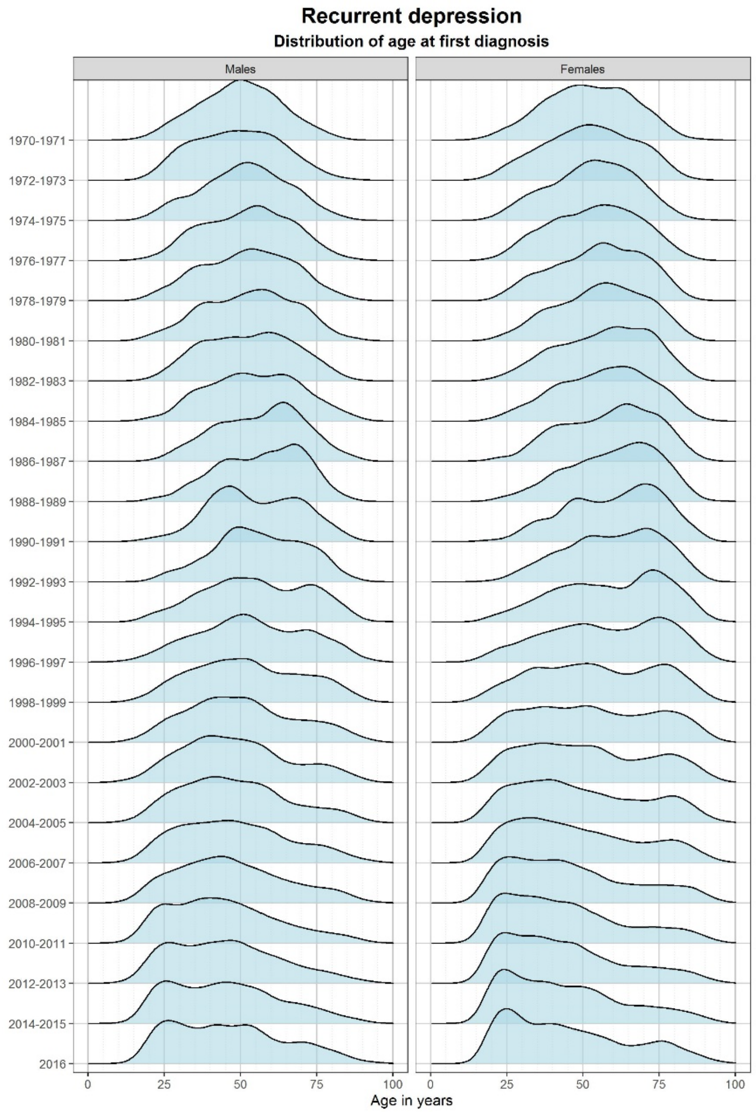

eFigure 2M.

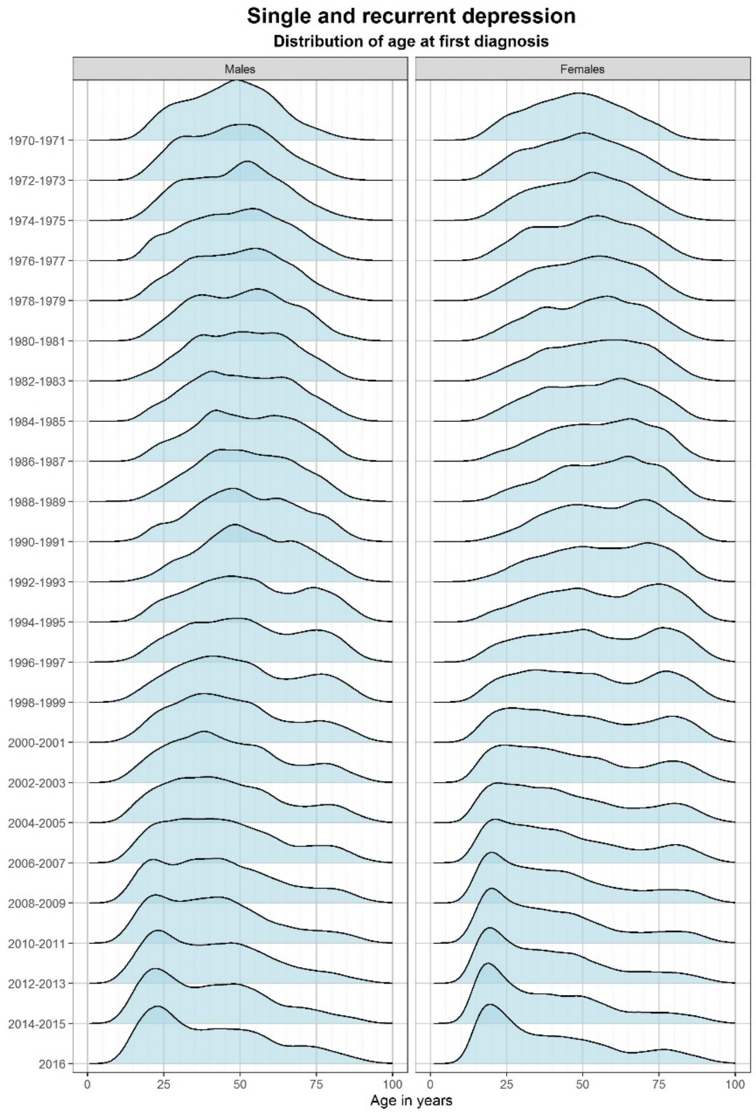

eFigure 2N.

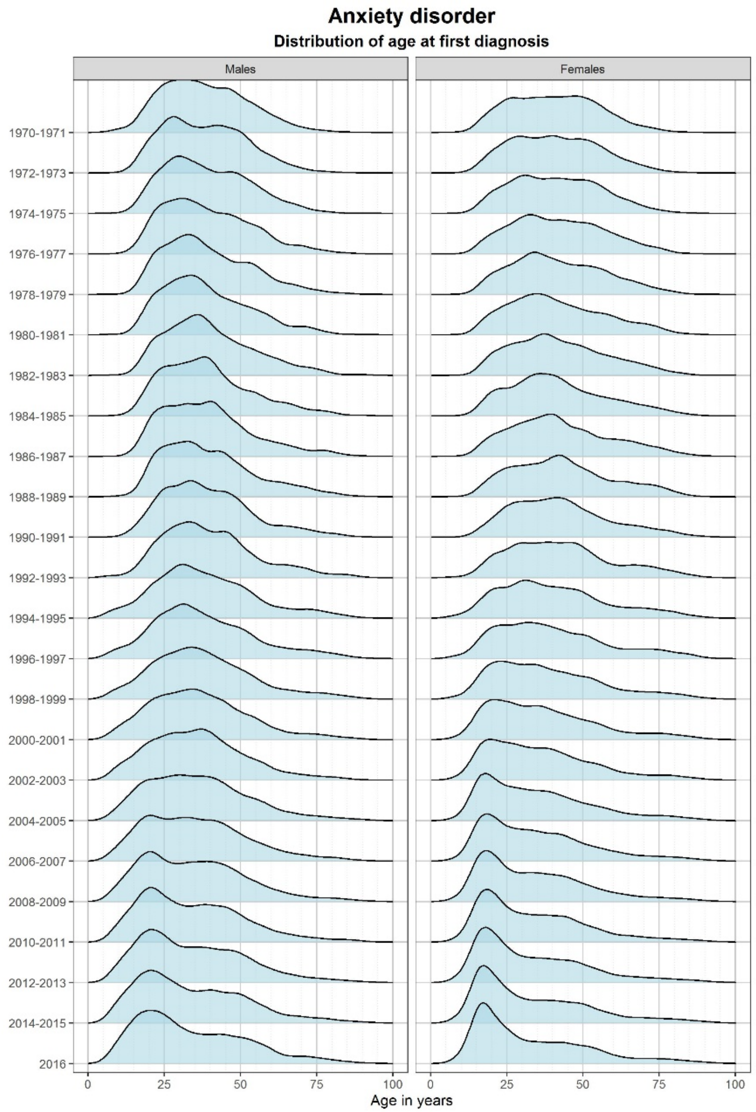

eFigure 2O.

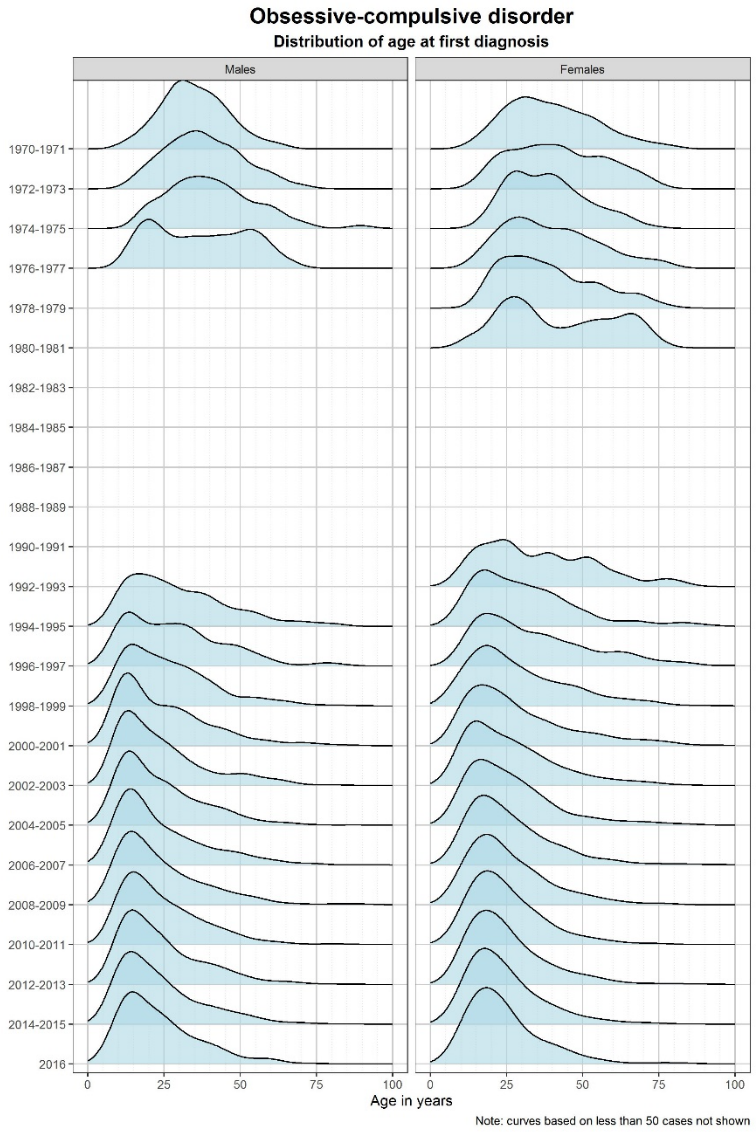

eFigure 2P.

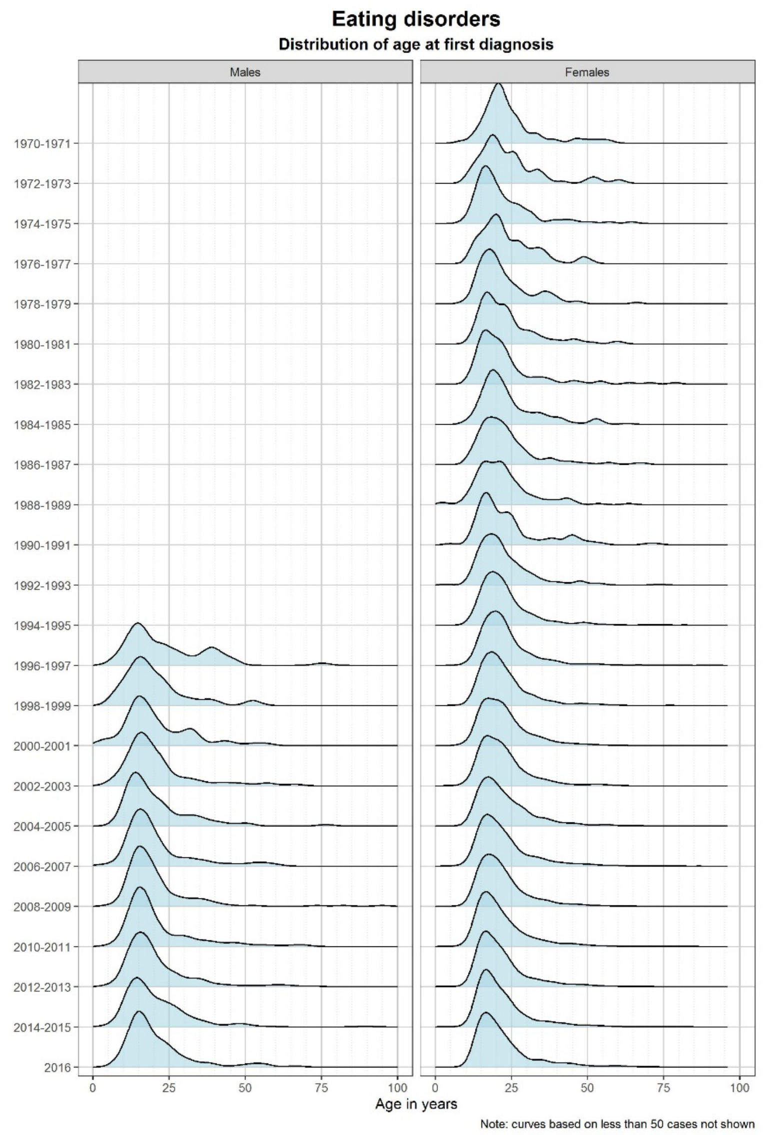

eFigure 2Q.

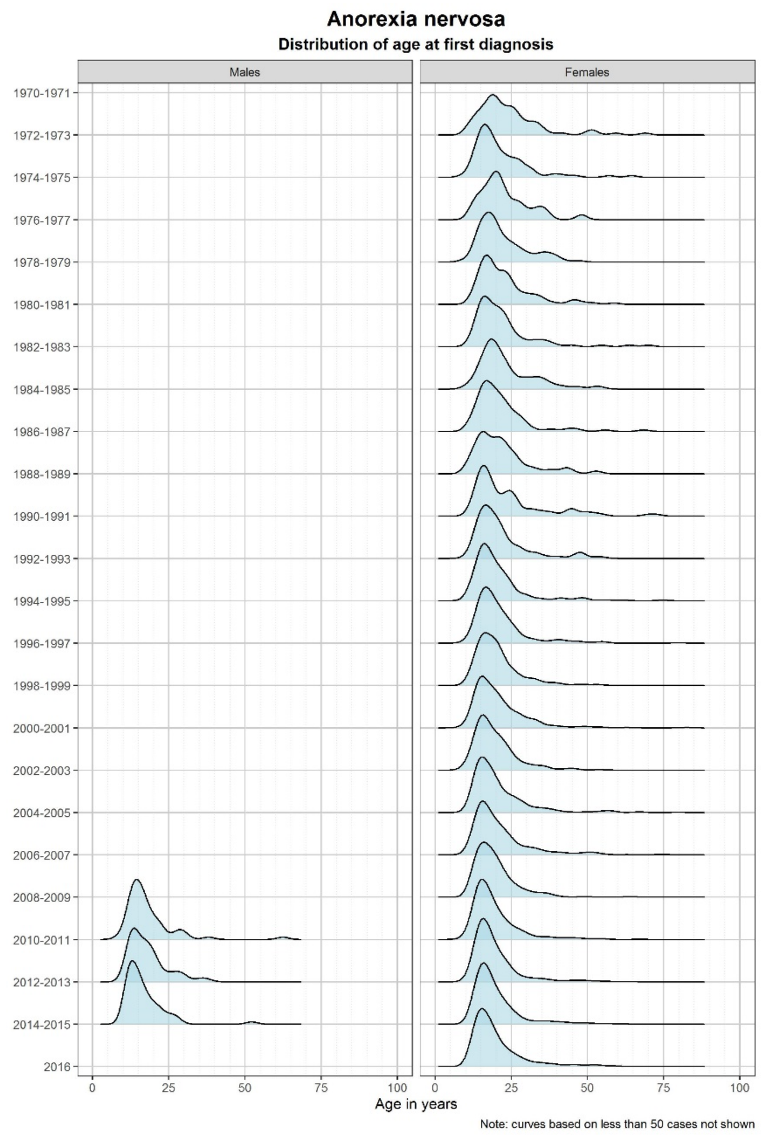

eFigure 2R.

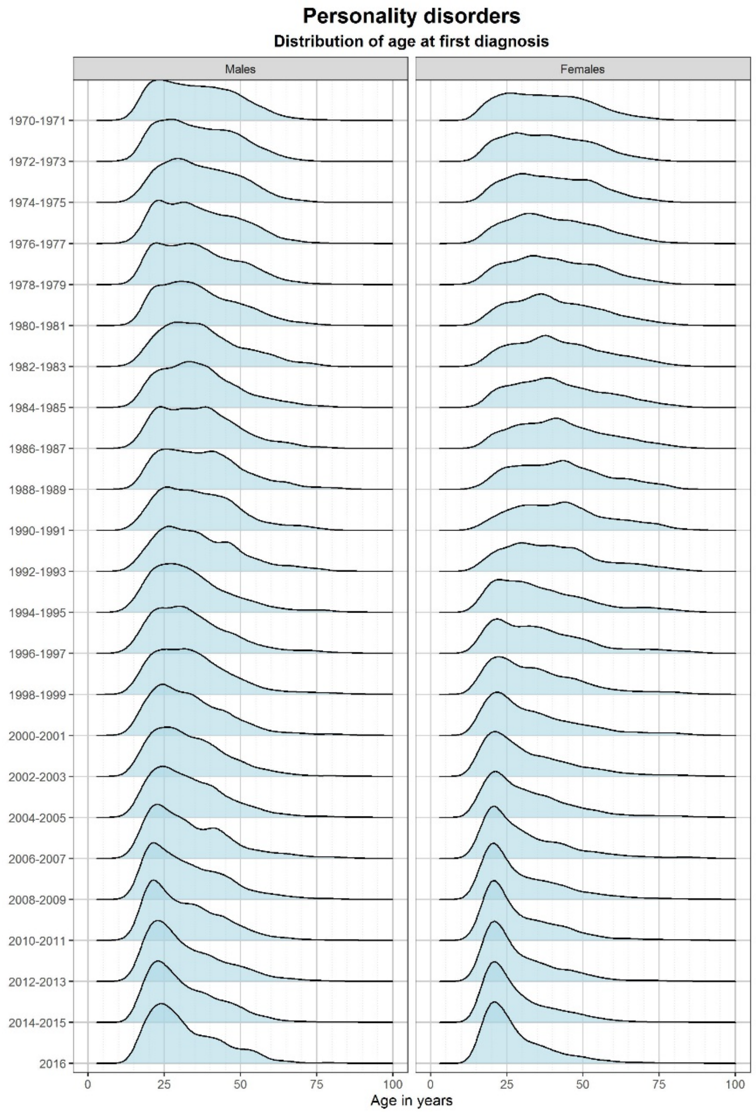

eFigure 2S.

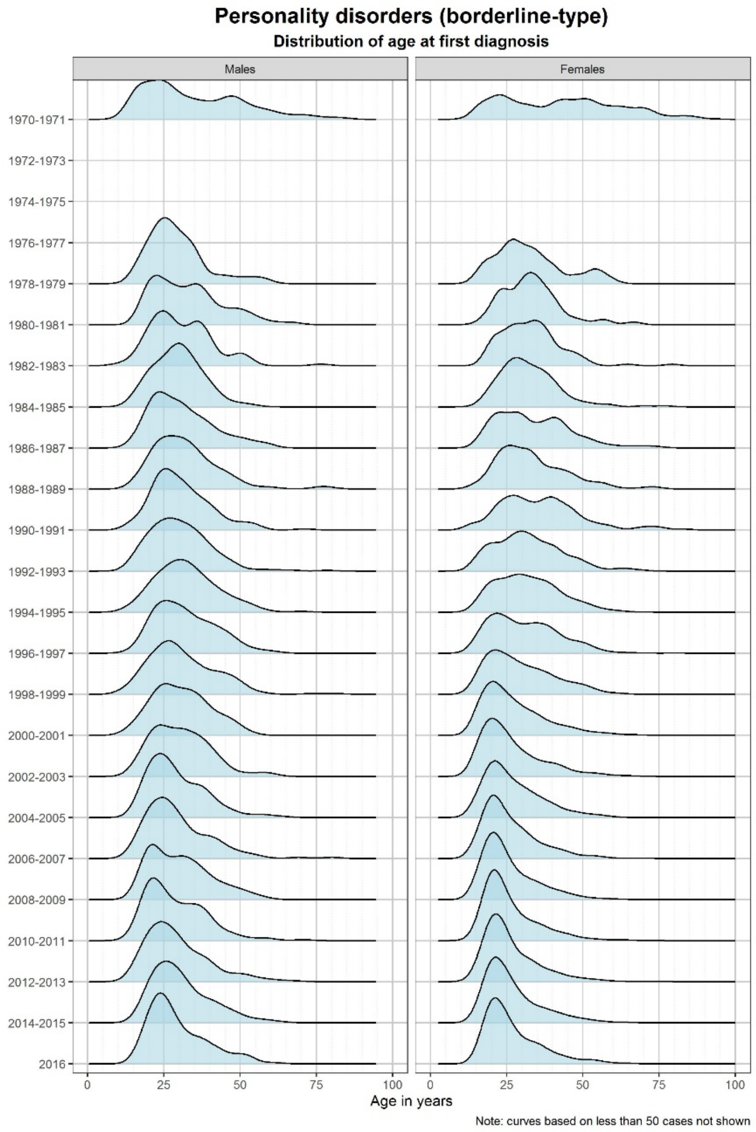

eFigure 2T.

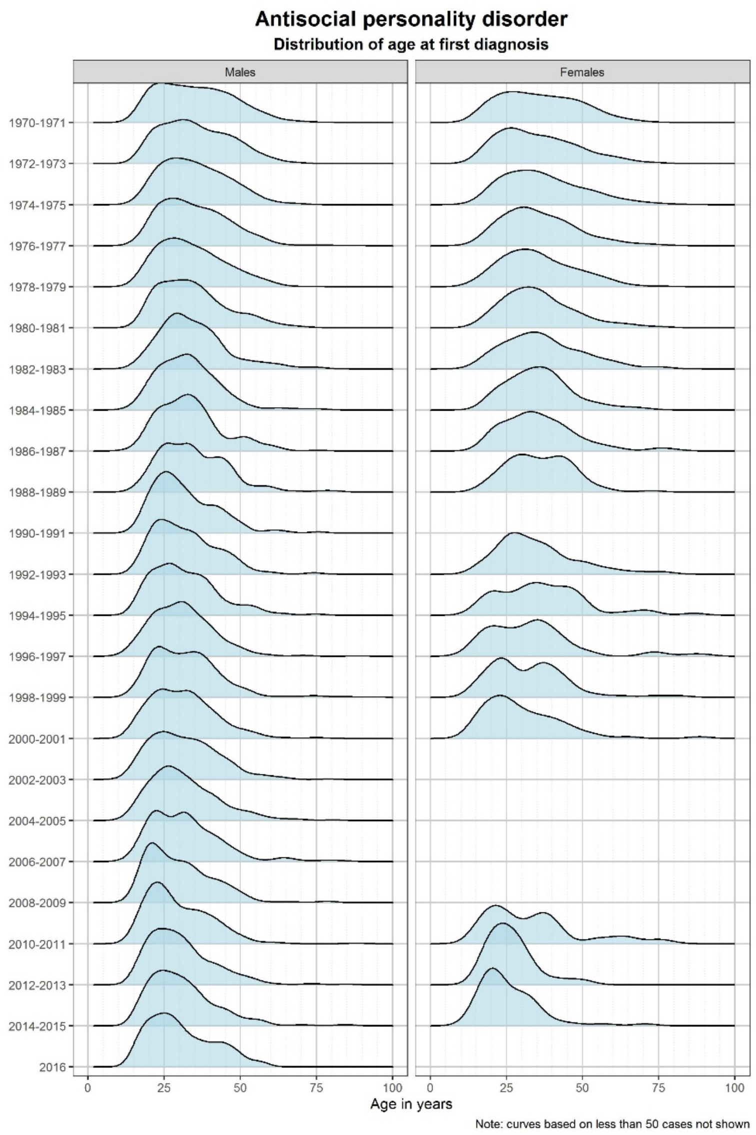

eFigure 2U.

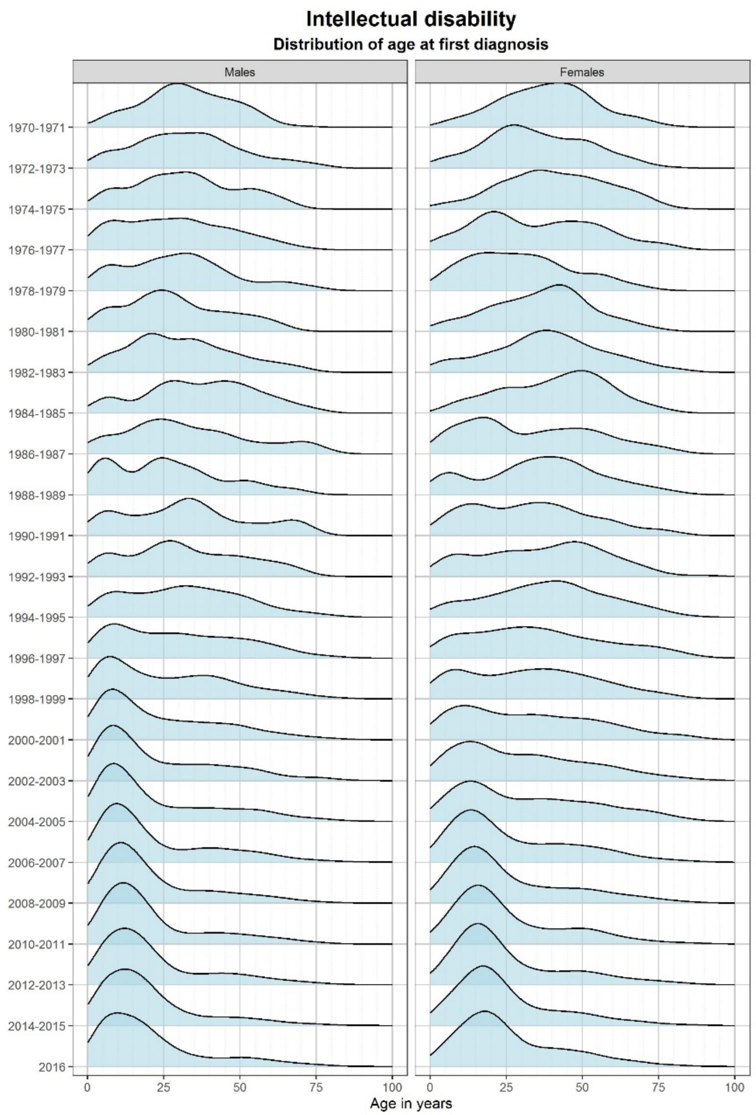

eFigure 2V.

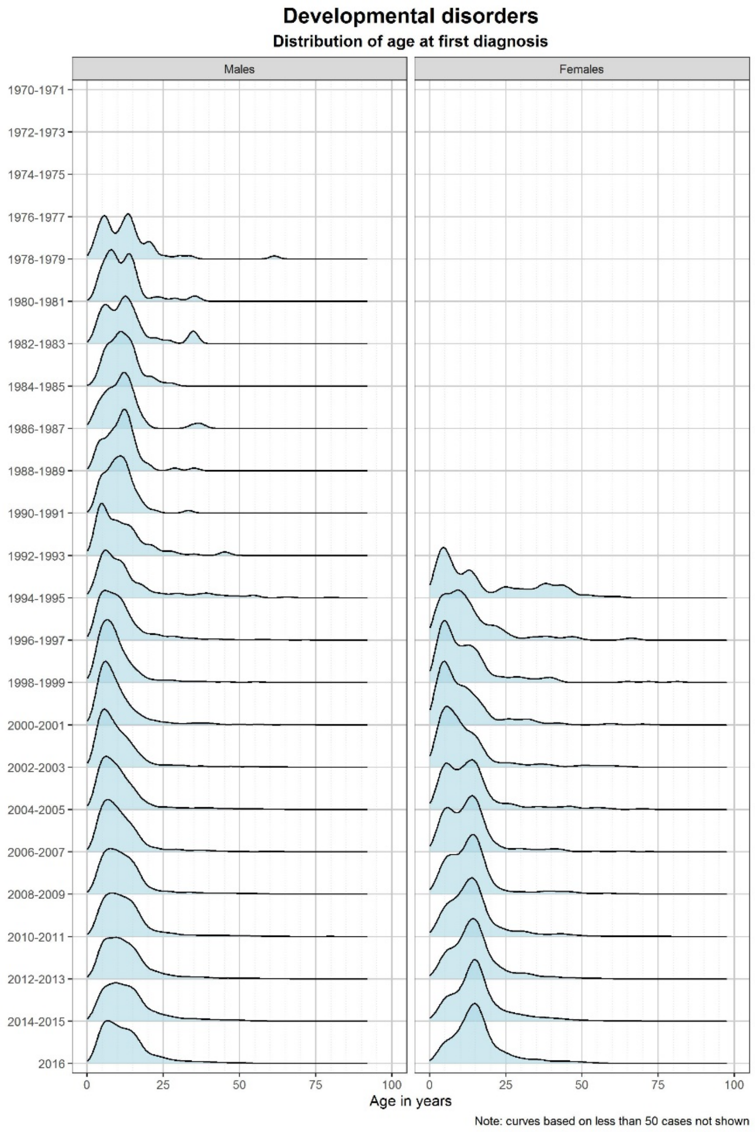

eFigure 2W.

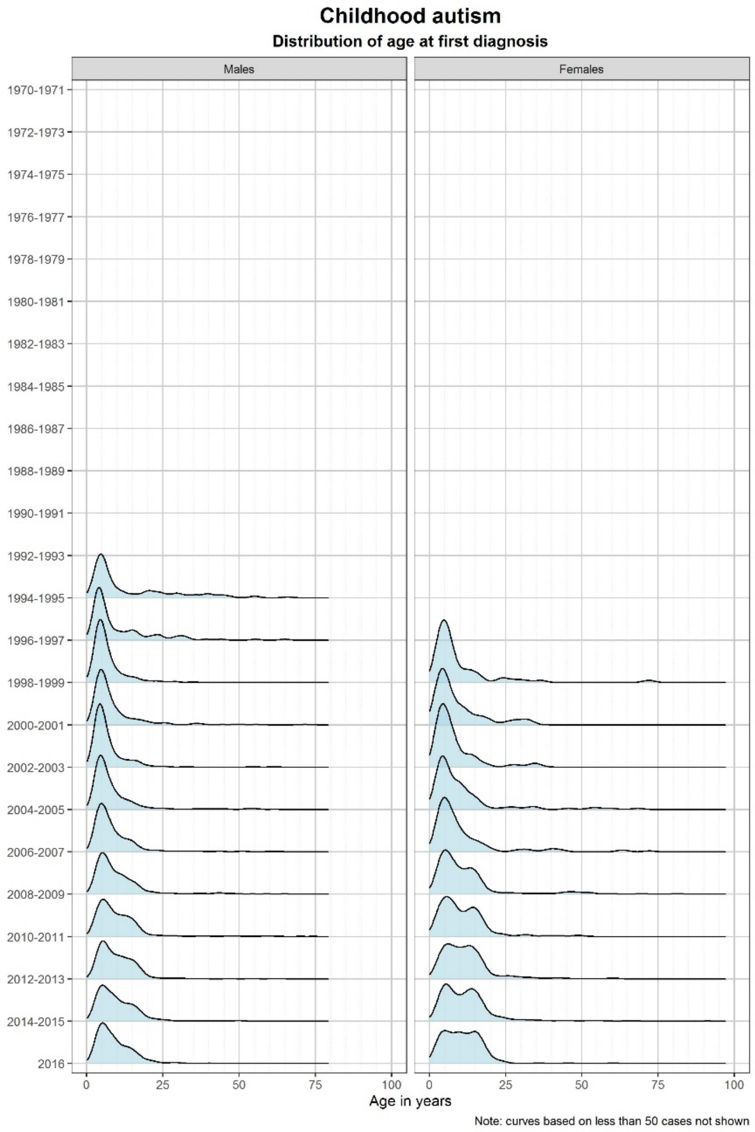

eFigure 2X.

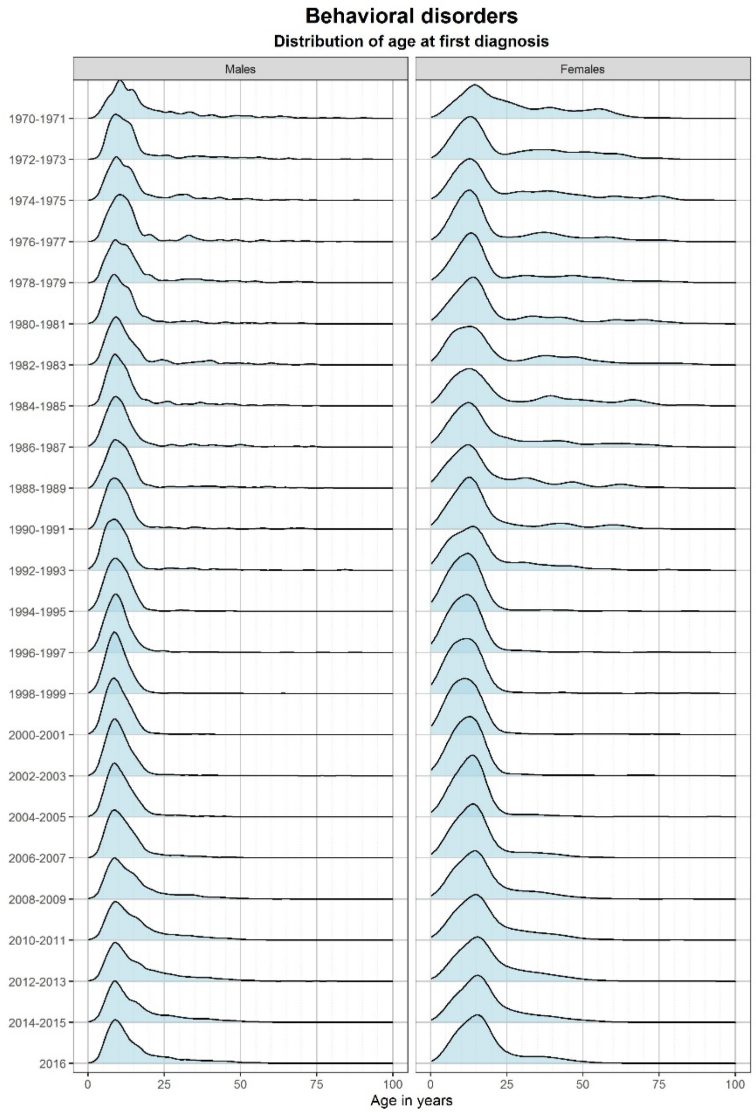

eFigure 2Y.

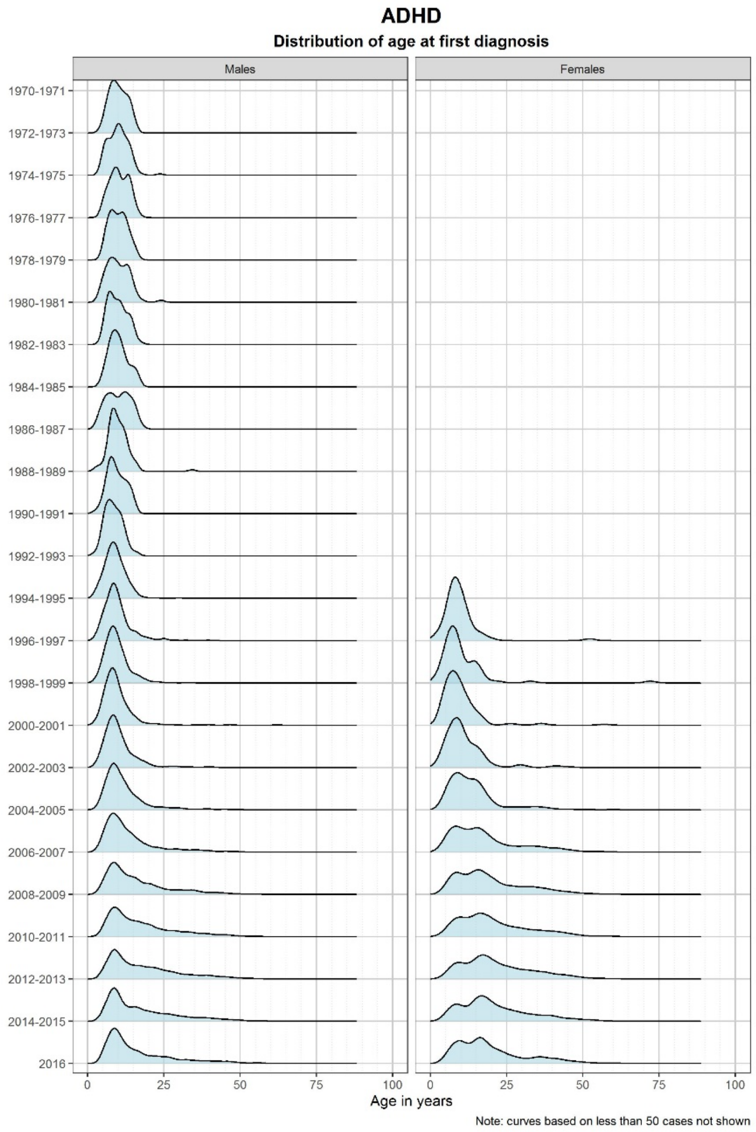

eFigures 3A-3Z. Age-specific incidence rates (for inpatient cases only), by calendar period

Note that the vertical scale is logarithmic and scaled differently for each disorder, depending on the number of cases.

eFigure 3A.

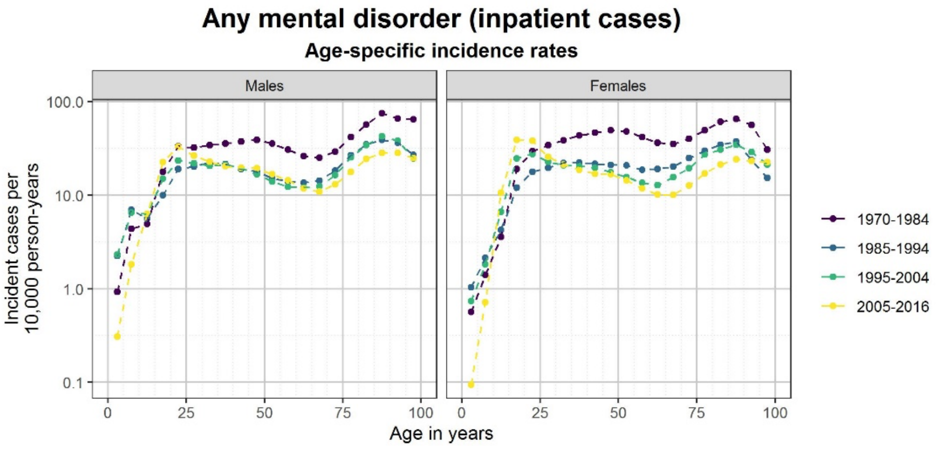

eFigure 3B.

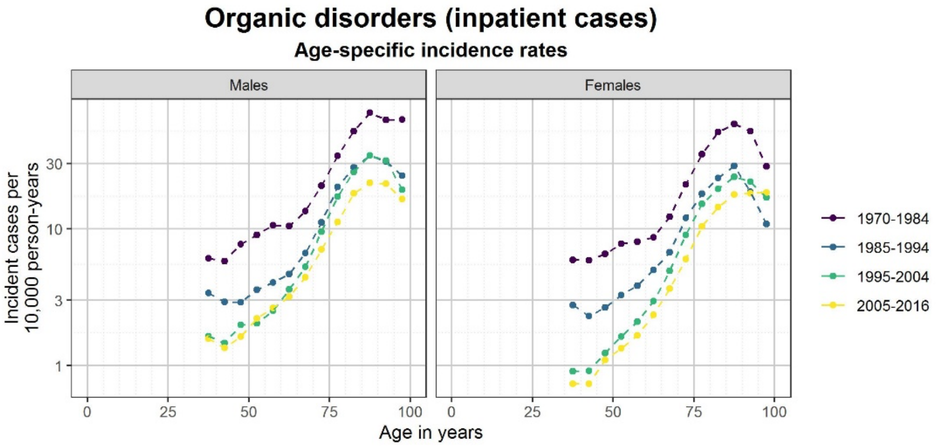

eFigure 3C.

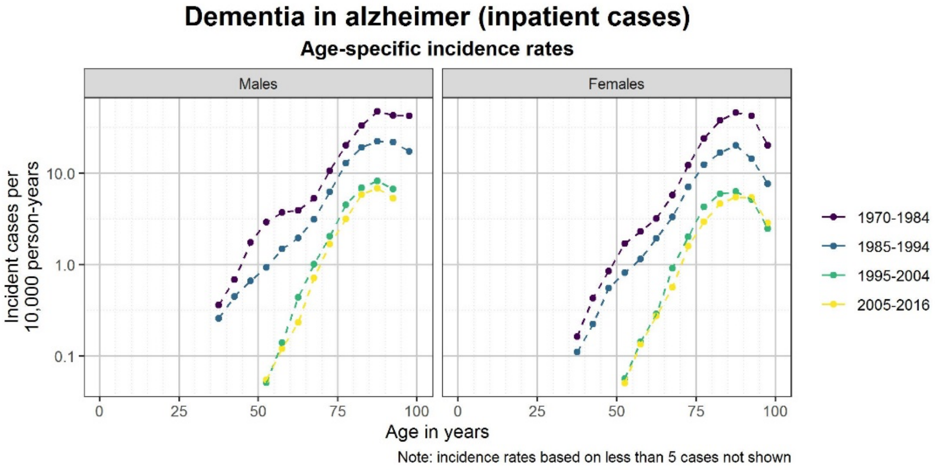

eFigure 3D.

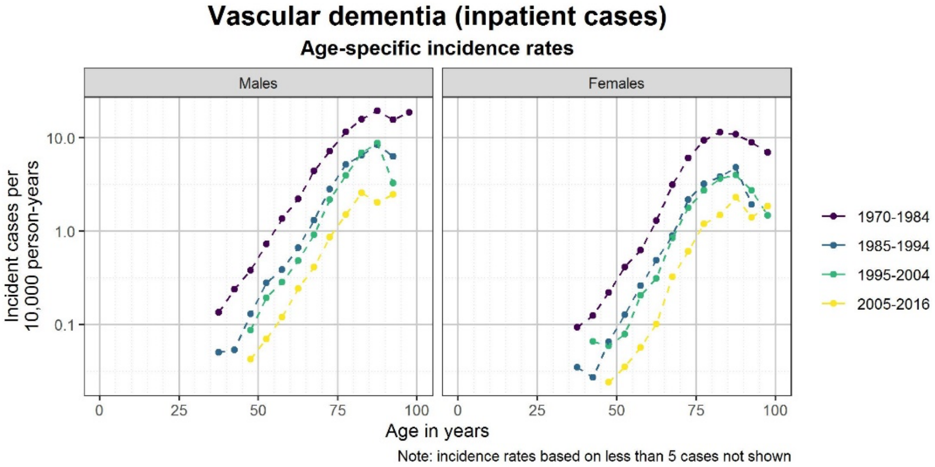

eFigure 3E.

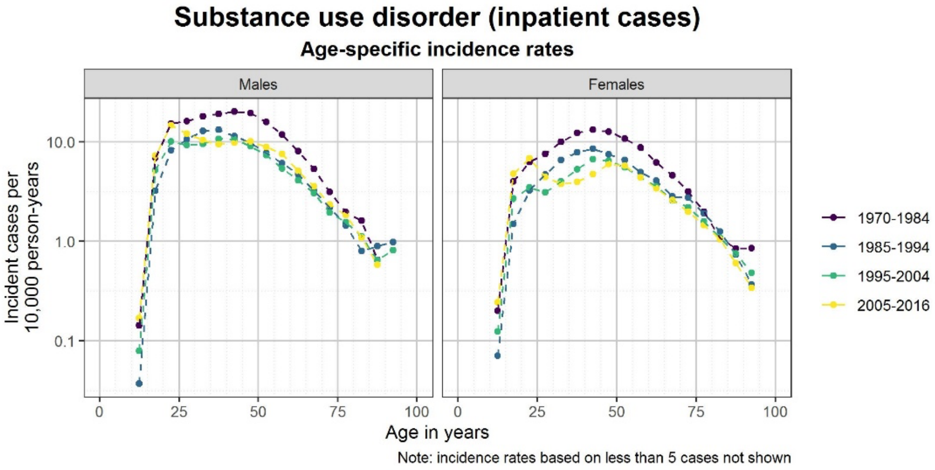

eFigure 3F.

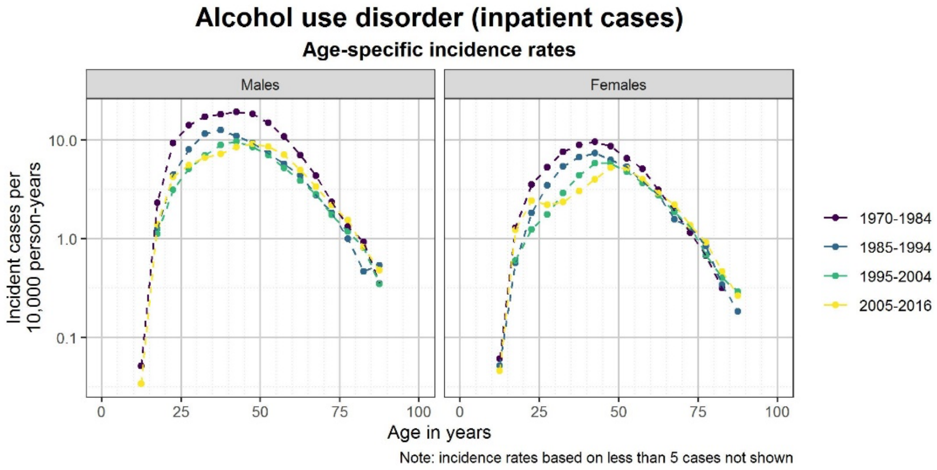

eFigure 3G.

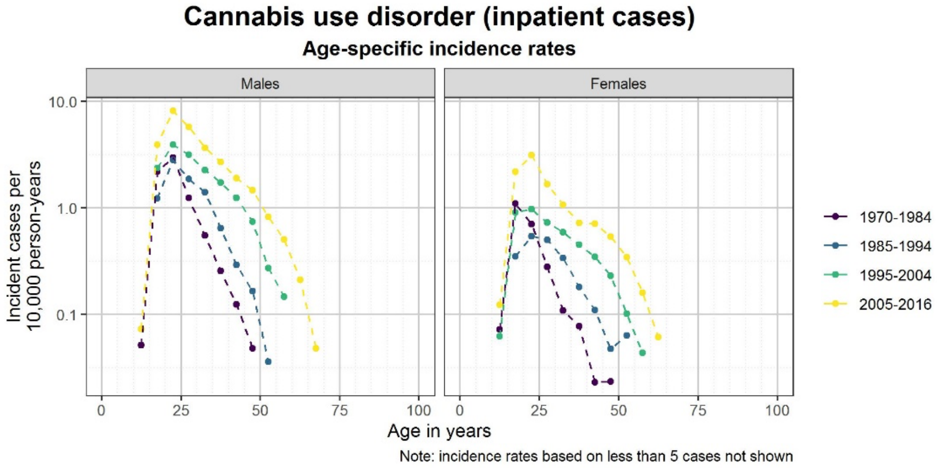

eFigure 3H.

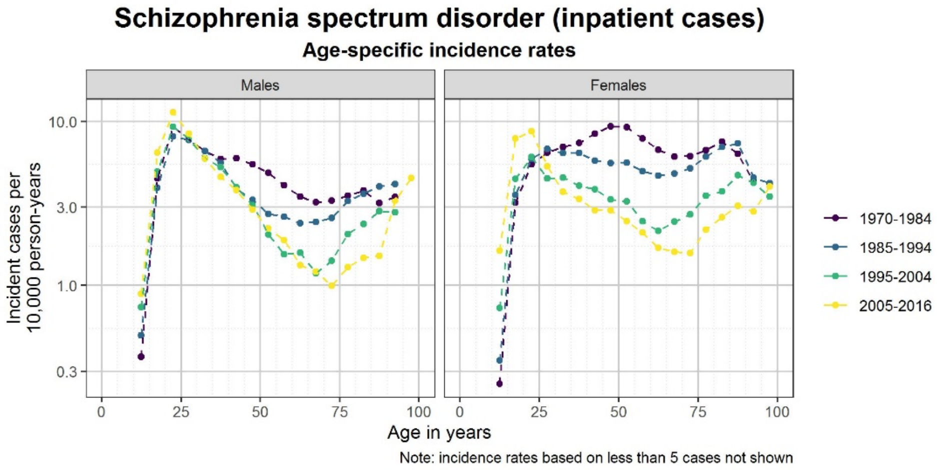

eFigure 3I.

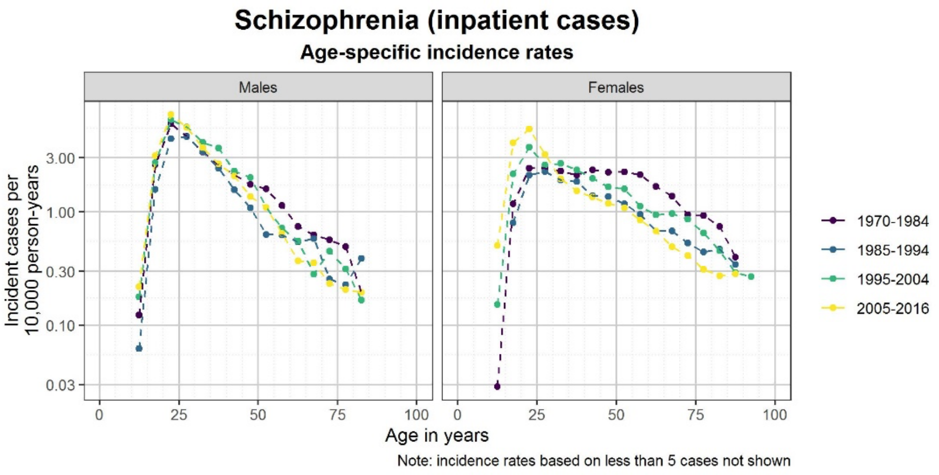

eFigure 3J.

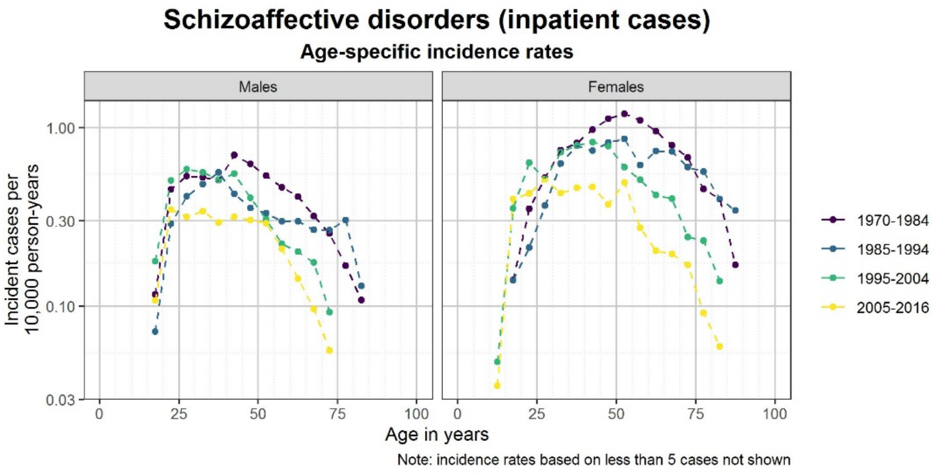

eFigure 3K.

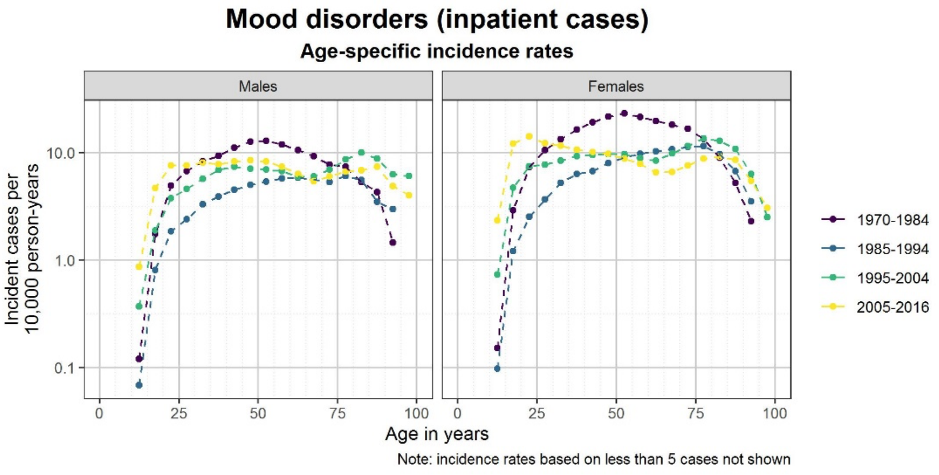

eFigure 3L.

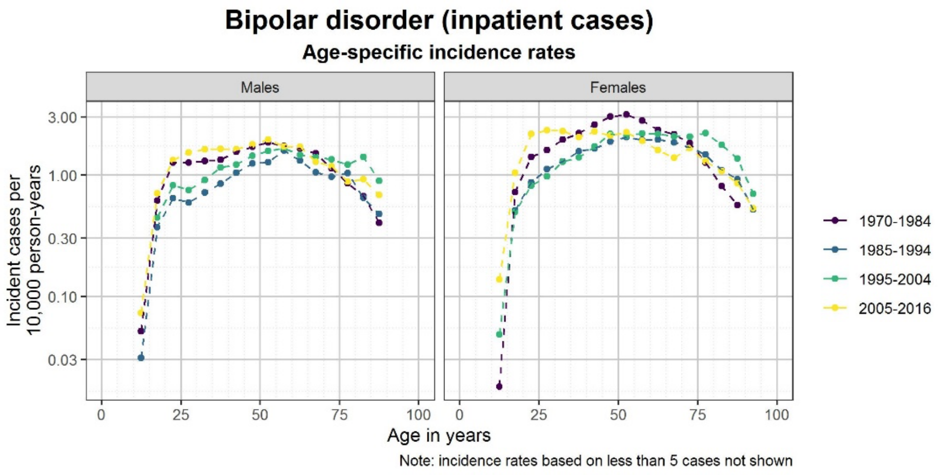

eFigure 3M.

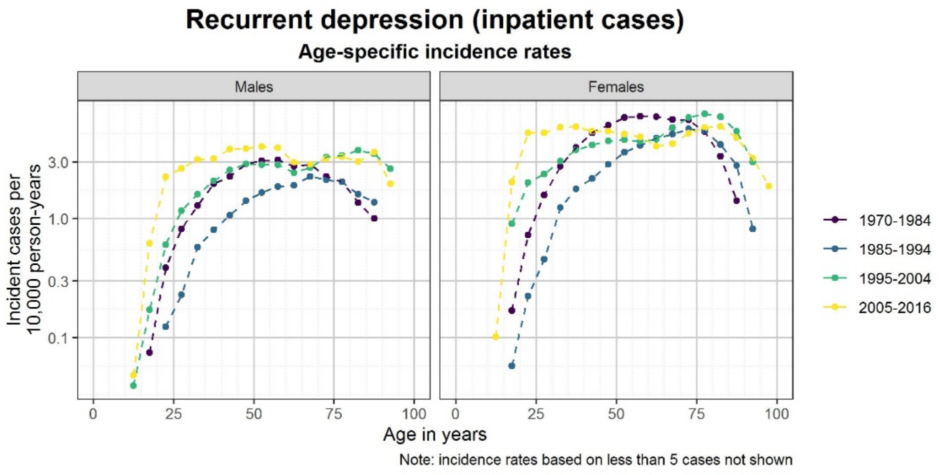

eFigure 3N.

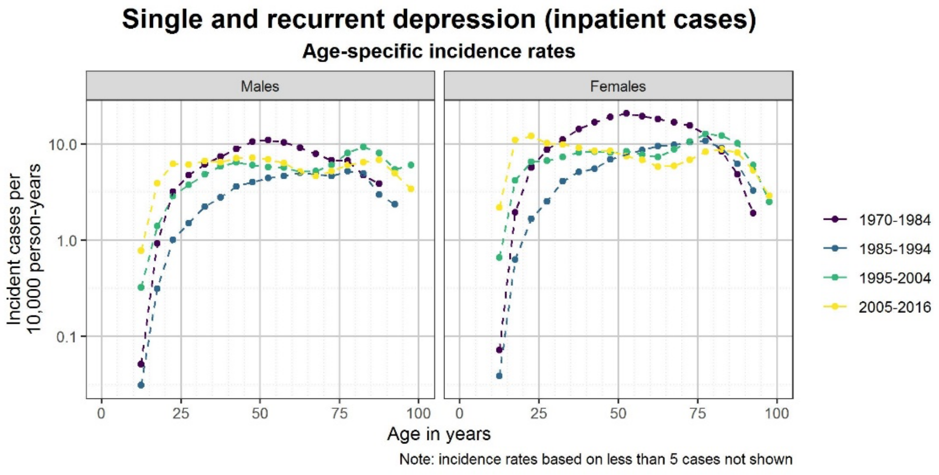

eFigure 3O.

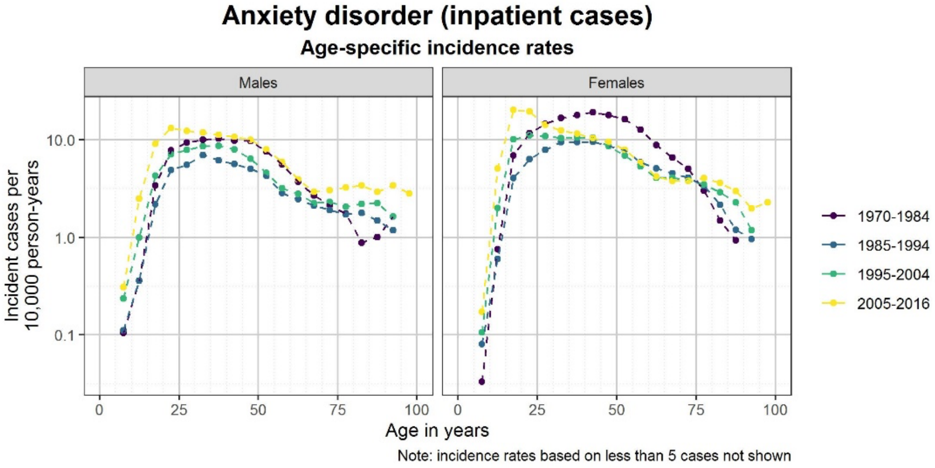

eFigure 3P.

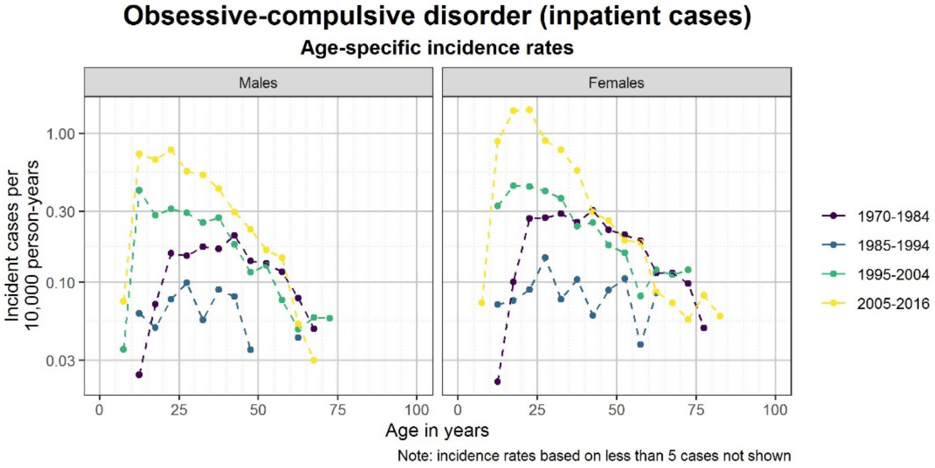

eFigure 3Q.

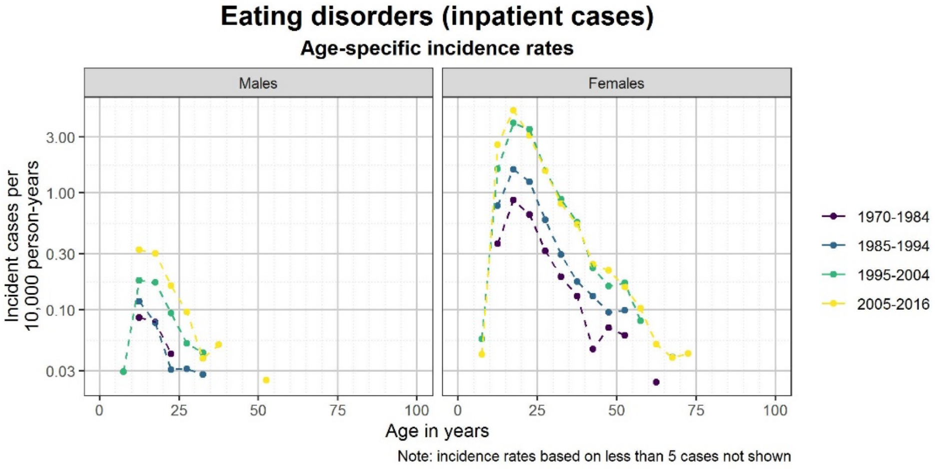

eFigure 3R.

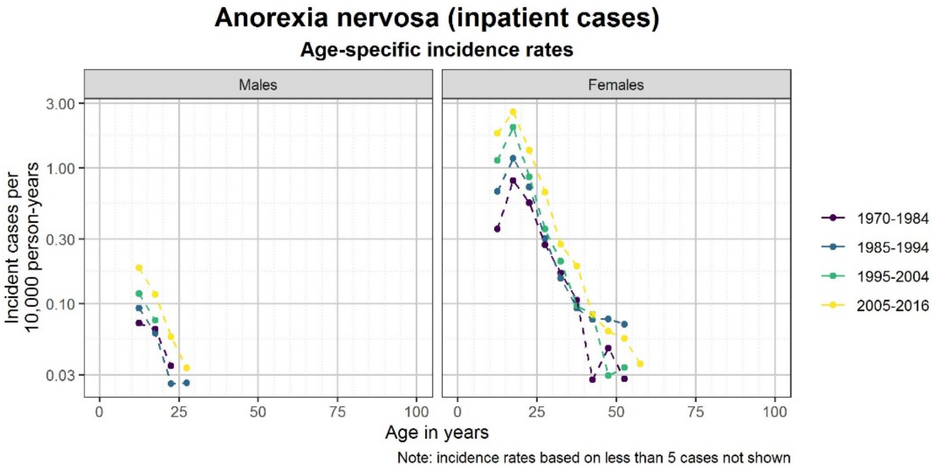

eFigure 3S.

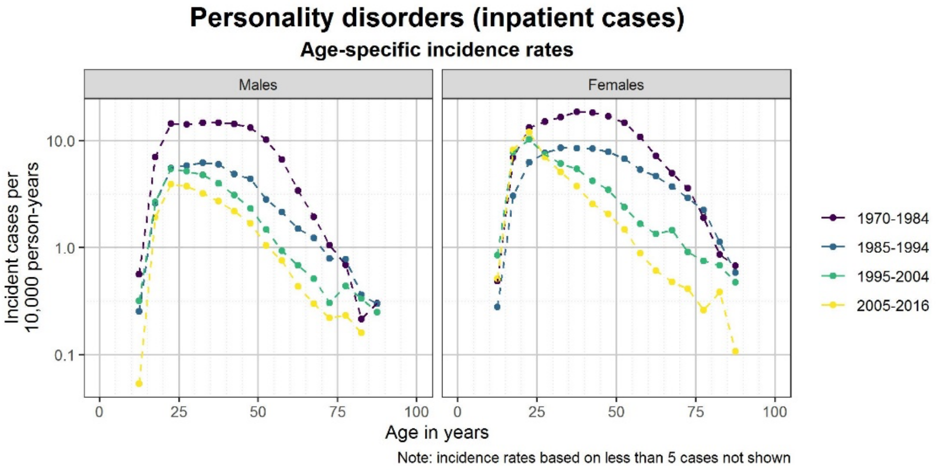

eFigure 3T.

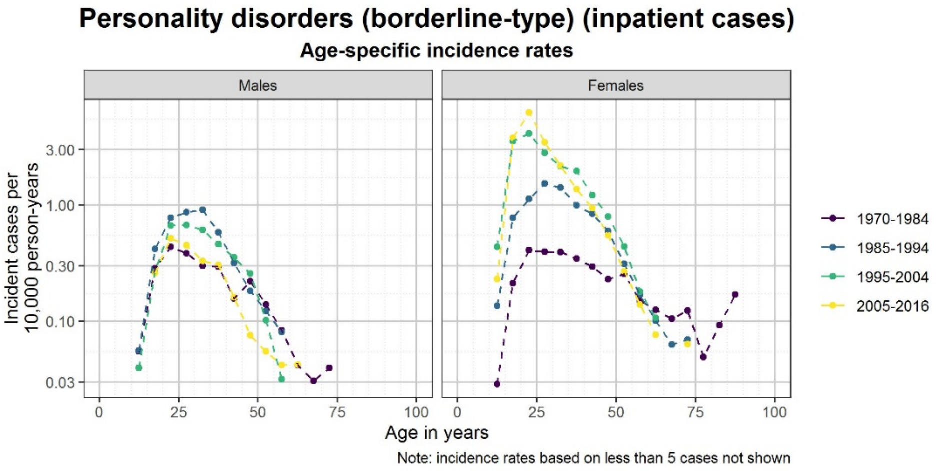

eFigure 3U.

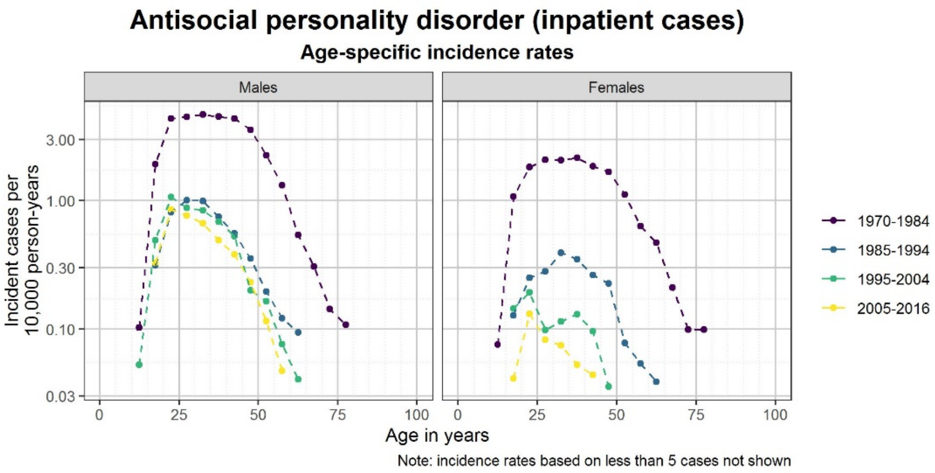

eFigure 3V.

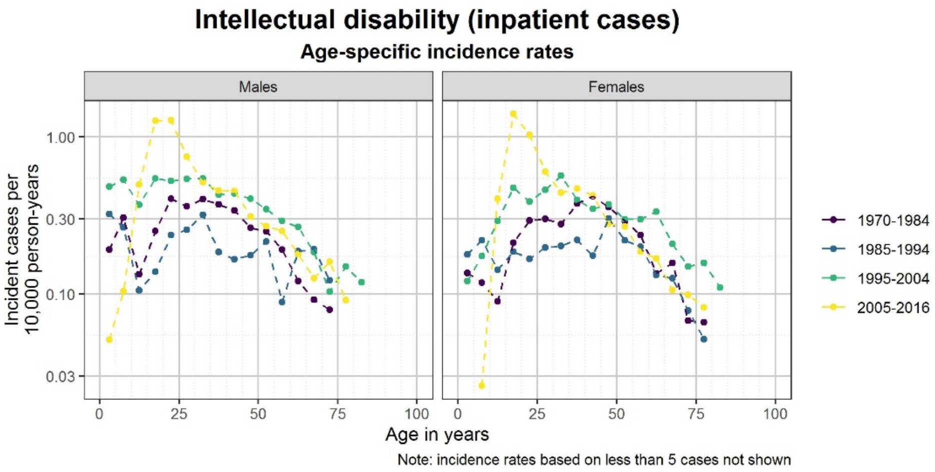

eFigure 3W.

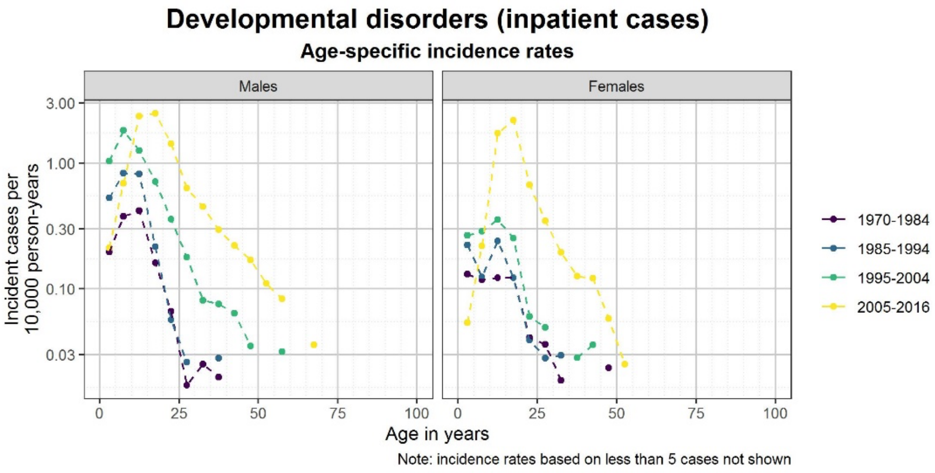

eFigure 3X.

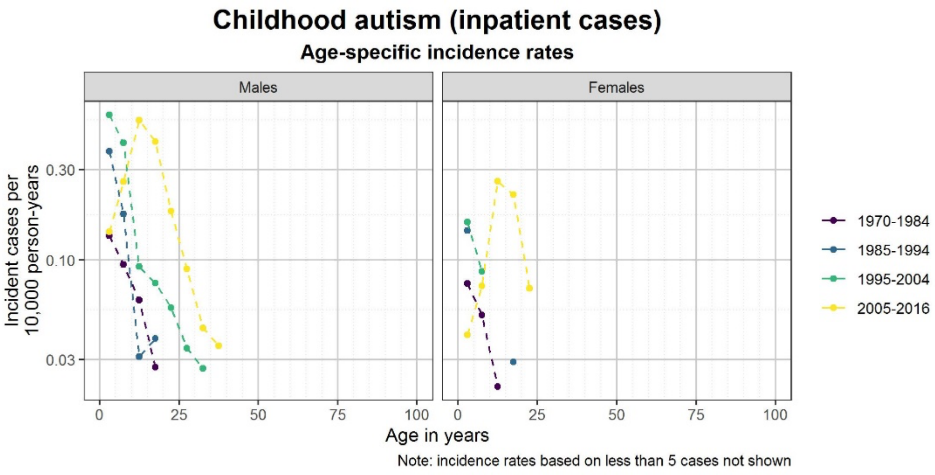

eFigure 3Y.

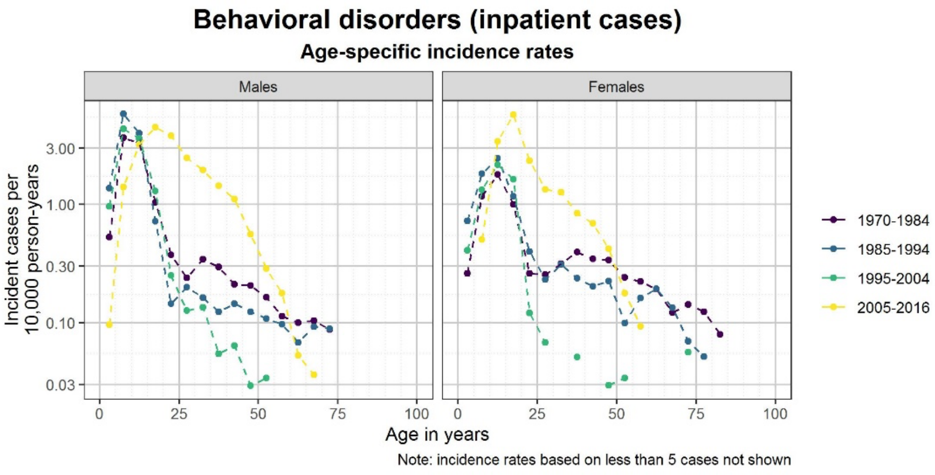

eFigure 3Z.

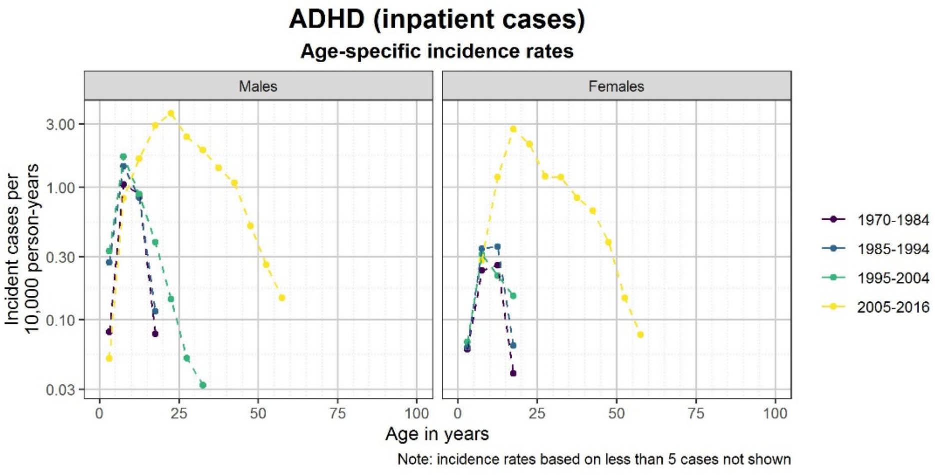

eTable 1. Diagnostic codes, number of cases and incidence rates (IR) per 10 000 person-years of mental disorders 1970- 2016, including all contacts.

Mental disorder	Diagnostic codes		Number of cases 1970-2016			IR with 95% CI		
	ICD-8	ICD-10[a]	All	Males	Females	All	Males	Females
Any mental disorder	290-315	F00-F99	866,524	397,296	469,228	38.99 (38.98-38.99)	36.01 (36.00-36.02)	41.92 (41.92-41.93)
Organic disorders	290.09, 290.10, 290.11, 290.18, 290.19, 292.x9, 293.x9, 294.x9, 309.x9	F00-F09	174,707	74,375	100,332	14.10 (14.10-14.11)	12.58 (12.57-12.58)	15.50 (15.49-15.50)
Alzheimer's disease	290.09, 290.10, 209.19	F00	72,383	27,553	44,830	5.81 (5.81-5.81)	4.64 (4.63-4.64)	6.88 (6.88-6.89)
Vascular dementia	293.09, 293.19	F01	31,348	14,924	16,424	2.51 (2.51-2.51)	2.51 (2.51-2.51)	2.52 (2.52-2.52)
Substance use disorder	291.x9, 294.39, 303.x9, 303.20, 303.28, 303.90, 304.x9	F10-F19	177,882	112,232	65,650	8.77 (8.77-8.78)	11.30 (11.30-11.30)	6.35 (6.35-6.35)
Alcohol use disorder	291.x9, 303.x9, 303.20, 303.38, 303.90	F10	129,796	85,628	44,168	6.38 (6.38-6.38)	8.59 (8.59-8.60)	4.26 (4.26-4.26)
Cannabis use disorder	304.59	F12	26,909	20,126	6,783	1.31 (1.31-1.31)	2.00 (2.00-2.00)	0.65 (0.65-0.65)
Schizophrenia spectrum disorder	295.x9, 296.89, 297.x9, 298.29-298.99, 299.04, 299.05, 299.09, 301.83	F20-F29	117,122	56,144	60,978	5.76 (5.76-5.76)	5.62 (5.61-5.62)	5.90 (5.89-5.90)
Schizophrenia	295.x9 (excluding 295.79)	F20	48,057	27,378	20,679	2.35 (2.35-2.35)	2.73 (2.73-2.73)	1.99 (1.99-1.99)
Schizoaffective disorders	295.79, 296.89	F25	10,130	3,863	6,267	0.49 (0.49-0.49)	0.38 (0.38-0.38)	0.60 (0.60-0.60)
Mood disorders	296.x9 (excluding 296.89), 298.09, 298.19, 300.49, 301.19	F30-F39	282,061	104,865	177,196	13.98 (13.97-13.98)	10.52 (10.52-10.52)	17.35 (17.34-17.35)
Bipolar disorder	296.19, 296.39, 298.19	F30-F31	39,233	15,774	23,459	1.92 (1.92-1.92)	1.57 (1.57-1.57)	2.25 (2.25-2.25)
Recurrent depression	296.09, 296.29, 298.09, 300.49[b]	F33	107,408	34,900	72,508	5.26 (5.26-5.26)	3.47 (3.47-3.47)	7.00 (6.99-7.00)
Single and recurrent depression	296.09, 296.29, 298.09, 300.49	F32-F33	252,645	91,203	161,442	12.49 (12.49-12.49)	9.13 (9.13-9.13)	15.77 (15.76-15.77)
Anxiety disorder	300.x9 (excluding 300.49), 305.x9, 305.68, 307.99	F40-F48	342,415	135,705	206,710	15.80 (15.80-15.80)	12.64 (12.64-12.64)	18.89 (18.89-18.90)
Obsessive-compulsive disorder	300.39	F42	19,857	7,734	12,123	0.90 (0.90-0.90)	0.71 (0.71-0.71)	1.08 (1.08-1.08)
Eating disorders	306.50, 306.58, 306.59	F50	23,797	1,517	22,280	1.02 (1.02-1.02)	0.13 (0.13-0.13)	1.89 (1.89-1.89)
Anorexia nervosa	306.50	F50.0	7,791	465	7,326	0.33 (0.33-0.33)	0.04 (0.04-0.04)	0.62 (0.62-0.62)
Personality disorders	301.x9 (excluding 301.19), 301.80, 301.81, 301.82, 301.84	F60	156,889	61,714	95,175	7.75 (7.75-7.75)	6.19 (6.19-6.19)	9.27 (9.26-9.27)
Personality disorders (borderline-type)	301.84	F60.31	22,227	3,885	18,342	1.08 (1.08-1.08)	0.39 (0.39-0.39)	1.76 (1.76-1.76)
Antisocial personality disorder	301.79, 301.82	F60.2	17,520	12,740	4,780	0.86 (0.85-0.86)	1.27 (1.27-1.27)	0.46 (0.46-0.46)
Intellectual disability	311.xx, 312.xx, 313.xx, 314.xx, 315.xx	F70-F79	25,358	15,072	10,286	1.09 (1.09-1.09)	1.31 (1.31-1.31)	0.87 (0.87-0.87)
Developmental disorders	299.00, 299.01, 299.02, 299.03	F84	34,668	25,673	8,995	1.49 (1.49-1.49)	2.24 (2.24-2.24)	0.76 (0.76-0.76)
Childhood autism	299.00	F84.0	10,004	7,872	2,132	0.43 (0.43-0.43)	0.68 (0.68-0.68)	0.18 (0.18-0.18)
Behavioral disorders	306.x9, 308.0x	F90-F98	90,240	58,204	32,036	3.89 (3.89-3.89)	5.08 (5.08-5.08)	2.72 (2.72-2.72)
ADHD	308.01	F90	51,787	35,522	16,265	2.23 (2.23-2.23)	3.09 (3.09-3.09)	1.38 (1.38-1.38)

Note:[a] Inclusive of diagnostic categories for which a valid conversion to ICD-8 was possible
[b] For recurrent depression, onset was defined as the second admission that occurred at least 8 weeks after last discharge with these ICD-8 codes

eTable 2. Number of *male* cases and incidence rate (IR) per 10 000 person-years of mental disorders, in four time periods, including all contacts.								
**Mental disorder**	**Number of incident male cases**				**IR with 95% CI in males**			
	**1970-1984**	**1985-1994**	**1995-2004**	**2005-2016**	**1970-1984**	**1985-1994**	**1995-2004**	**2005-2016**
Any mental disorder	93,811	39,196	99,283	165,006	26.15 (26.14-26.16)	16.80 (16.79-16.80)	42.59 (42.57-42.60)	59.34 (59.31-59.36)
Organic disorders	22,061	8,375	19,314	24,625	12.87 (12.87-12.88)	6.79 (6.79-6.80)	14.83 (14.82-14.84)	14.79 (14.79-14.80)
Alzheimer's disease	9,331	3,883	5,037	9,302	5.42 (5.42-5.43)	3.14 (3.13-3.14)	3.85 (3.84-3.85)	5.55 (5.55-5.56)
Vascular dementia	4,825	1,477	4,429	4,193	2.80 (2.80-2.80)	1.19 (1.19-1.19)	3.38 (3.38-3.38)	2.50 (2.50-2.50)
Substance use disorder	37,762	15,774	25,483	33,213	12.04 (12.03-12.04)	7.45 (7.45-7.46)	12.18 (12.17-12.18)	12.85 (12.84-12.85)
Alcohol use disorder	32,353	13,473	19,391	20,411	10.30 (10.30-10.30)	6.35 (6.35-6.35)	9.23 (9.23-9.24)	7.85 (7.84-7.85)
Cannabis use disorder	2,129	1,609	4,638	11,750	0.67 (0.67-0.67)	0.75 (0.75-0.75)	2.18 (2.18-2.18)	4.47 (4.47-4.47)
Schizophrenia spectrum disorder	16,007	9,423	12,664	18,050	5.08 (5.08-5.08)	4.42 (4.42-4.42)	6.00 (6.00-6.01)	6.92 (6.92-6.92)
Schizophrenia	7,198	4,111	7,178	8,891	2.28 (2.28-2.28)	1.92 (1.92-1.92)	3.39 (3.39-3.39)	3.39 (3.39-3.39)
Schizoaffective disorders	1,265	715	1,072	811	0.40 (0.40-0.40)	0.33 (0.33-0.33)	0.50 (0.50-0.50)	0.31 (0.31-0.31)
Mood disorders	23,941	8,105	26,181	46,638	7.61 (7.61-7.61)	3.81 (3.80-3.81)	12.43 (12.43-12.44)	18.03 (18.02-18.04)
Bipolar disorder	3,869	1,956	3,527	6,422	1.22 (1.22-1.22)	0.91 (0.91-0.91)	1.66 (1.66-1.66)	2.44 (2.44-2.44)
Recurrent depression	5,103	2,264	8,894	18,639	1.61 (1.61-1.62)	1.06 (1.06-1.06)	4.19 (4.19-4.19)	7.10 (7.10-7.10)
Single and recurrent depression	19,366	6,327	23,390	42,120	6.15 (6.15-6.15)	2.97 (2.96-2.97)	11.08 (11.08-11.09)	16.24 (16.23-16.24)
Anxiety disorder	19,923	8,901	34,431	72,450	5.80 (5.80-5.80)	3.92 (3.92-3.92)	15.18 (15.18-15.19)	26.24 (26.23-26.25)
Obsessive-compulsive disorder	360	168	1,762	5,444	0.10 (0.10-0.10)	0.07 (0.07-0.07)	0.77 (0.77-0.77)	1.92 (1.92-1.92)
Eating disorders	79	62	345	1,031	0.02 (0.02-0.02)	0.03 (0.03-0.03)	0.14 (0.14-0.14)	0.34 (0.34-0.34)
Anorexia nervosa	57	43	93	272	0.02 (0.02-0.02)	0.02 (0.02-0.02)	0.04 (0.04-0.04)	0.09 (0.09-0.09)
Personality disorders	28,440	7,805	12,521	12,948	9.06 (9.05-9.06)	3.68 (3.67-3.68)	5.95 (5.95-5.96)	4.97 (4.97-4.97)
Personality disorders (borderline-type)	644	851	1,149	1,241	0.20 (0.20-0.20)	0.40 (0.40-0.40)	0.54 (0.54-0.54)	0.47 (0.47-0.47)
Antisocial personality disorder	8,211	962	1,853	1,714	2.60 (2.60-2.60)	0.45 (0.45-0.45)	0.87 (0.87-0.87)	0.65 (0.65-0.65)
Intellectual disability	936	749	4,425	8,962	0.26 (0.26-0.26)	0.31 (0.31-0.31)	1.82 (1.82-1.82)	3.00 (3.00-3.00)
Developmental disorders	357	517	4,963	19,836	0.10 (0.10-0.10)	0.21 (0.21-0.21)	2.04 (2.04-2.04)	6.65 (6.65-6.65)
Childhood autism	90	153	1,276	6,353	0.02 (0.02-0.02)	0.06 (0.06-0.06)	0.52 (0.52-0.52)	2.12 (2.12-2.12)
Behavioral disorders	2,963	2,288	11,069	41,884	0.81 (0.81-0.81)	0.95 (0.95-0.95)	4.56 (4.56-4.56)	14.15 (14.14-14.15)
ADHD	600	439	3,823	30,660	0.16 (0.16-0.16)	0.18 (0.18-0.18)	1.57 (1.57-1.57)	10.29 (10.29-10.29)

eTable 3. Number of *female* cases and incidence rate (IR) per 10 000 person-years of mental disorders, in four time periods, including all contacts.								
**Mental disorder**	**Number of incident female cases**				**IR with 95% CI in females**			
	**1970-1984**	**1985-1994**	**1995-2004**	**2005-2016**	**1970-1984**	**1985-1994**	**1995-2004**	**2005-2016**
Any mental disorder	115,317	46,016	122,117	185,778	31.55 (31.54-31.56)	19.26 (19.25-19.26)	51.69 (51.67-51.71)	66.69 (66.67-66.72)
Organic disorders	25,514	10,589	28,809	35,420	13.45 (13.45-13.46)	7.69 (7.68-7.69)	20.21 (20.20-20.23)	19.96 (19.95-19.97)
Alzheimer's disease	12,738	5,573	9,852	16,667	6.69 (6.69-6.69)	4.02 (4.02-4.03)	6.86 (6.86-6.87)	9.32 (9.32-9.33)
Vascular dementia	4,667	1,445	5,118	5,194	2.45 (2.45-2.45)	1.04 (1.04-1.04)	3.56 (3.56-3.56)	2.90 (2.89-2.90)
Substance use disorder	23,317	10,083	14,346	17,904	7.15 (7.15-7.16)	4.54 (4.54-4.54)	6.56 (6.56-6.57)	6.69 (6.68-6.69)
Alcohol use disorder	14,673	7,671	10,709	11,115	4.49 (4.49-4.49)	3.45 (3.44-3.45)	4.88 (4.88-4.89)	4.13 (4.13-4.14)
Cannabis use disorder	655	385	1,296	4,447	0.20 (0.20-0.20)	0.17 (0.17-0.17)	0.59 (0.59-0.59)	1.64 (1.64-1.64)
Schizophrenia spectrum disorder	20,455	11,931	12,192	16,400	6.28 (6.28-6.28)	5.38 (5.37-5.38)	5.58 (5.58-5.58)	6.12 (6.12-6.12)
Schizophrenia	5,642	2,842	5,357	6,838	1.73 (1.73-1.73)	1.27 (1.27-1.27)	2.43 (2.43-2.43)	2.53 (2.53-2.53)
Schizoaffective disorders	2,170	1,268	1,603	1,226	0.66 (0.66-0.66)	0.57 (0.57-0.57)	0.73 (0.73-0.73)	0.45 (0.45-0.45)
Mood disorders	43,716	14,890	43,961	74,629	13.50 (13.49-13.50)	6.76 (6.76-6.76)	20.34 (20.34-20.35)	28.56 (28.55-28.57)
Bipolar disorder	6,005	3,262	5,150	9,042	1.84 (1.84-1.84)	1.46 (1.46-1.46)	2.34 (2.34-2.34)	3.35 (3.35-3.35)
Recurrent depression	12,678	5,904	18,569	35,357	3.88 (3.88-3.88)	2.65 (2.65-2.65)	8.46 (8.46-8.47)	13.22 (13.22-13.23)
Single and recurrent depression	38,855	12,853	40,413	69,321	11.98 (11.97-11.98)	5.82 (5.82-5.83)	18.65 (18.64-18.66)	26.44 (26.43-26.45)
Anxiety disorder	36,428	14,493	54,738	101,051	10.35 (10.35-10.36)	6.19 (6.19-6.19)	23.67 (23.66-23.68)	36.51 (36.50-36.53)
Obsessive-compulsive disorder	589	203	2,250	9,081	0.17 (0.17-0.17)	0.09 (0.09-0.09)	0.95 (0.95-0.95)	3.13 (3.13-3.14)
Eating disorders	740	999	6,864	13,677	0.20 (0.20-0.20)	0.40 (0.40-0.40)	2.75 (2.75-2.75)	4.50 (4.50-4.50)
Anorexia nervosa	651	611	1,949	4,115	0.17 (0.17-0.17)	0.25 (0.25-0.25)	0.78 (0.78-0.78)	1.35 (1.35-1.35)
Personality disorders	34,668	12,663	20,210	27,634	10.67 (10.67-10.68)	5.74 (5.74-5.74)	9.32 (9.32-9.32)	10.43 (10.43-10.44)
Personality disorders (borderline-type)	762	1,619	5,372	10,589	0.23 (0.23-0.23)	0.72 (0.72-0.72)	2.44 (2.43-2.44)	3.92 (3.92-3.92)
Antisocial personality disorder	3,773	364	338	305	1.15 (1.15-1.15)	0.16 (0.16-0.16)	0.15 (0.15-0.15)	0.11 (0.11-0.11)
Intellectual disability	830	661	3,060	5,735	0.22 (0.22-0.22)	0.27 (0.27-0.27)	1.22 (1.22-1.22)	1.88 (1.88-1.88)
Developmental disorders	171	178	1,118	7,528	0.05 (0.05-0.05)	0.07 (0.07-0.07)	0.45 (0.45-0.45)	2.47 (2.47-2.47)
Childhood autism	44	56	329	1,703	0.01 (0.01-0.01)	0.02 (0.02-0.02)	0.13 (0.13-0.13)	0.56 (0.56-0.56)
Behavioral disorders	1,829	1,445	4,673	24,089	0.49 (0.49-0.49)	0.58 (0.58-0.58)	1.87 (1.87-1.87)	7.94 (7.94-7.94)
ADHD	169	132	669	15,295	0.05 (0.05-0.05)	0.05 (0.05-0.05)	0.27 (0.27-0.27)	5.02 (5.02-5.02)

eTable 4. Sex- and age-specific time-trends for incidence rates of mental disorders in Denmark

			Both sexes		Males		Females	
Years	Age	Mental disorder	Cases	IR (95% CI)	Cases	IR (95% CI)	Cases	IR (95% CI)
1970-2016	1-5	Any mental disorder	9,462	7.81 (7.81-7.82)	6,912	11.15 (11.14-11.15)	2,550	4.32 (4.31-4.32)
1970-1984	1-5	Any mental disorder	308	0.76 (0.76-0.76)	195	0.94 (0.93-0.94)	113	0.57 (0.57-0.57)
1985-1994	1-5	Any mental disorder	487	2.12 (2.12-2.12)	338	2.87 (2.87-2.88)	149	1.33 (1.33-1.33)
1995-2004	1-5	Any mental disorder	3,149	11.63 (11.61-11.64)	2,260	16.29 (16.26-16.31)	889	6.73 (6.72-6.74)
2005-2016	1-5	Any mental disorder	5,518	18.18 (18.16-18.20)	4,119	26.50 (26.46-26.54)	1,399	9.44 (9.43-9.46)
1970-2016	5-10	Any mental disorder	38,251	24.71 (24.70-24.72)	29,122	36.88 (36.85-36.90)	9,129	12.04 (12.03-12.05)
1970-1984	5-10	Any mental disorder	1,623	2.92 (2.91-2.92)	1,241	4.36 (4.35-4.36)	382	1.41 (1.40-1.41)
1985-1994	5-10	Any mental disorder	1,561	5.58 (5.58-5.59)	1,193	8.36 (8.34-8.37)	368	2.69 (2.69-2.70)
1995-2004	5-10	Any mental disorder	10,244	31.41 (31.37-31.44)	7,921	47.54 (47.47-47.61)	2,323	14.56 (14.54-14.58)
2005-2016	5-10	Any mental disorder	24,823	64.39 (64.32-64.45)	18,767	95.99 (95.85-96.12)	6,056	31.87 (31.83-31.92)
1970-2016	10-15	Any mental disorder	48,109	30.90 (30.88-30.91)	25,831	32.61 (32.59-32.64)	22,278	29.12 (29.10-29.14)
1970-1984	10-15	Any mental disorder	2,437	4.29 (4.28-4.29)	1,437	4.94 (4.93-4.95)	1,000	3.60 (3.60-3.60)
1985-1994	10-15	Any mental disorder	1,887	6.00 (6.00-6.01)	1,098	6.85 (6.84-6.86)	789	5.12 (5.11-5.13)
1995-2004	10-15	Any mental disorder	11,232	38.68 (38.64-38.73)	6,184	41.83 (41.77-41.90)	5,048	35.41 (35.35-35.47)
2005-2016	10-15	Any mental disorder	32,553	84.87 (84.79-84.96)	17,112	88.67 (88.55-88.80)	15,441	81.03 (80.91-81.14)
1970-2016	15-20	Any mental disorder	80,923	51.74 (51.72-51.77)	31,213	38.91 (38.89-38.94)	49,710	65.24 (65.20-65.29)
1970-1984	15-20	Any mental disorder	10,409	18.33 (18.32-18.35)	5,167	17.73 (17.71-17.75)	5,242	18.98 (18.95-19.00)
1985-1994	15-20	Any mental disorder	4,140	11.79 (11.78-11.81)	1,912	10.63 (10.61-10.64)	2,228	13.02 (13.00-13.04)
1995-2004	15-20	Any mental disorder	18,151	65.51 (65.43-65.59)	6,257	44.04 (43.97-44.11)	11,894	88.10 (87.95-88.25)
2005-2016	15-20	Any mental disorder	48,223	130.98 (130.85-131.11)	17,877	94.79 (94.65-94.92)	30,346	168.99 (168.75-169.24)
1970-2016	20-25	Any mental disorder	81,153	52.28 (52.25-52.30)	37,349	46.39 (46.36-46.42)	43,804	58.62 (58.58-58.66)
1970-1984	20-25	Any mental disorder	17,117	31.32 (31.29-31.34)	9,340	33.11 (33.07-33.15)	7,777	29.40 (29.37-29.44)
1985-1994	20-25	Any mental disorder	7,144	19.40 (19.38-19.42)	3,772	19.70 (19.67-19.72)	3,372	19.09 (19.06-19.12)
1995-2004	20-25	Any mental disorder	19,774	66.04 (65.97-66.12)	8,137	52.25 (52.17-52.33)	11,637	80.99 (80.86-81.13)
2005-2016	20-25	Any mental disorder	37,118	109.75 (109.63-109.86)	16,100	91.61 (91.48-91.75)	21,018	129.36 (129.16-129.56)
1970-2016	25-30	Any mental disorder	69,817	45.05 (45.02-45.07)	33,301	41.53 (41.50-41.56)	36,516	48.81 (48.78-48.85)
1970-1984	25-30	Any mental disorder	18,436	33.26 (33.24-33.29)	9,224	32.38 (32.35-32.42)	9,212	34.19 (34.15-34.23)
1985-1994	25-30	Any mental disorder	7,607	21.12 (21.10-21.14)	3,922	21.02 (20.99-21.05)	3,685	21.24 (21.20-21.27)
1995-2004	25-30	Any mental disorder	18,260	56.38 (56.32-56.44)	8,290	49.27 (49.20-49.34)	9,970	64.07 (63.97-64.18)
2005-2016	25-30	Any mental disorder	25,514	81.87 (81.78-81.96)	11,865	73.20 (73.09-73.31)	13,649	91.26 (91.11-91.41)
1970-2016	30-35	Any mental disorder	66,371	43.42 (43.40-43.44)	31,699	40.31 (40.28-40.33)	34,672	46.72 (46.68-46.75)
1970-1984	30-35	Any mental disorder	19,191	36.33 (36.30-36.36)	9,224	34.23 (34.19-34.27)	9,967	38.52 (38.48-38.57)
1985-1994	30-35	Any mental disorder	7,797	23.38 (23.36-23.41)	3,989	23.25 (23.22-23.29)	3,808	23.52 (23.48-23.56)
1995-2004	30-35	Any mental disorder	17,547	50.90 (50.85-50.95)	8,366	46.95 (46.88-47.02)	9,181	55.13 (55.05-55.22)
2005-2016	30-35	Any mental disorder	21,836	67.76 (67.69-67.84)	10,120	60.52 (60.43-60.61)	11,716	75.58 (75.46-75.70)
1970-2016	35-40	Any mental disorder	63,858	42.46 (42.44-42.48)	30,068	39.11 (39.09-39.14)	33,790	45.96 (45.93-45.99)
1970-1984	35-40	Any mental disorder	18,893	39.43 (39.40-39.47)	8,668	35.67 (35.62-35.71)	10,225	43.31 (43.26-43.37)
1985-1994	35-40	Any mental disorder	7,657	22.99 (22.96-23.01)	3,801	22.43 (22.39-22.46)	3,856	23.57 (23.53-23.60)
1995-2004	35-40	Any mental disorder	16,559	48.63 (48.57-48.68)	7,979	45.66 (45.59-45.73)	8,580	51.75 (51.67-51.83)

			Both sexes		Males		Females	
**Years**	**Age**	**Mental disorder**	**Cases**	**IR (95% CI)**	**Cases**	**IR (95% CI)**	**Cases**	**IR (95% CI)**
2005-2016	35-40	Any mental disorder	20,749	59.08 (59.02-59.15)	9,620	53.02 (52.94-53.09)	11,129	65.57 (65.47-65.67)
1970-2016	40-45	Any mental disorder	58,905	40.22 (40.20-40.25)	27,605	37.13 (37.10-37.15)	31,300	43.42 (43.39-43.45)
1970-1984	40-45	Any mental disorder	17,621	42.04 (42.00-42.08)	7,879	37.42 (37.37-37.47)	9,742	46.71 (46.65-46.78)
1985-1994	40-45	Any mental disorder	7,460	21.36 (21.33-21.38)	3,522	19.92 (19.89-19.95)	3,938	22.83 (22.80-22.87)
1995-2004	40-45	Any mental disorder	14,020	44.23 (44.18-44.28)	6,759	41.84 (41.78-41.90)	7,261	46.72 (46.64-46.79)
2005-2016	40-45	Any mental disorder	19,804	52.25 (52.20-52.30)	9,445	48.53 (48.46-48.60)	10,359	56.18 (56.10-56.26)
1970-2016	45-50	Any mental disorder	54,048	38.07 (38.05-38.09)	24,994	34.92 (34.89-34.95)	29,054	41.28 (41.25-41.31)
1970-1984	45-50	Any mental disorder	17,870	44.10 (44.05-44.14)	7,849	38.93 (38.88-38.98)	10,021	49.21 (49.14-49.28)
1985-1994	45-50	Any mental disorder	6,492	20.34 (20.31-20.36)	2,994	18.57 (18.54-18.59)	3,498	22.15 (22.11-22.18)
1995-2004	45-50	Any mental disorder	11,910	37.71 (37.67-37.75)	5,619	35.21 (35.15-35.26)	6,291	40.27 (40.20-40.33)
2005-2016	45-50	Any mental disorder	17,776	46.86 (46.81-46.91)	8,532	44.15 (44.08-44.21)	9,244	49.68 (49.61-49.75)
1970-2016	50-55	Any mental disorder	46,327	34.12 (34.10-34.13)	20,876	30.71 (30.68-30.73)	25,451	37.53 (37.50-37.56)
1970-1984	50-55	Any mental disorder	17,092	41.92 (41.88-41.96)	7,179	35.63 (35.58-35.68)	9,913	48.07 (48.00-48.13)
1985-1994	50-55	Any mental disorder	4,926	18.71 (18.69-18.73)	2,104	15.97 (15.94-16.00)	2,822	21.45 (21.42-21.49)
1995-2004	50-55	Any mental disorder	10,339	31.49 (31.45-31.52)	4,811	29.13 (29.09-29.18)	5,528	33.87 (33.82-33.92)
2005-2016	50-55	Any mental disorder	13,970	38.96 (38.92-39.00)	6,782	37.37 (37.32-37.43)	7,188	40.58 (40.52-40.64)
1970-2016	55-60	Any mental disorder	37,586	29.22 (29.20-29.23)	17,031	26.70 (26.68-26.72)	20,555	31.70 (31.67-31.72)
1970-1984	55-60	Any mental disorder	14,700	36.42 (36.38-36.46)	6,085	30.79 (30.75-30.83)	8,615	41.82 (41.76-41.88)
1985-1994	55-60	Any mental disorder	4,051	16.99 (16.97-17.01)	1,710	14.54 (14.52-14.57)	2,341	19.37 (19.34-19.41)
1995-2004	55-60	Any mental disorder	7,996	27.04 (27.01-27.07)	3,743	25.29 (25.25-25.33)	4,253	28.80 (28.75-28.84)
2005-2016	55-60	Any mental disorder	10,839	31.08 (31.05-31.12)	5,493	31.43 (31.38-31.48)	5,346	30.74 (30.69-30.78)
1970-2016	60-65	Any mental disorder	29,998	24.85 (24.83-24.86)	13,191	22.39 (22.37-22.41)	16,807	27.19 (27.17-27.21)
1970-1984	60-65	Any mental disorder	12,119	31.41 (31.38-31.44)	4,845	26.09 (26.05-26.13)	7,274	36.34 (36.29-36.39)
1985-1994	60-65	Any mental disorder	3,919	16.90 (16.88-16.93)	1,556	13.91 (13.88-13.93)	2,363	19.70 (19.67-19.74)
1995-2004	60-65	Any mental disorder	5,953	25.11 (25.08-25.14)	2,727	23.40 (23.35-23.44)	3,226	26.77 (26.72-26.81)
2005-2016	60-65	Any mental disorder	8,007	22.71 (22.69-22.73)	4,063	23.22 (23.19-23.25)	3,944	22.21 (22.17-22.24)
1970-2016	65-70	Any mental disorder	28,611	26.33 (26.31-26.34)	12,062	23.37 (23.35-23.39)	16,549	29.00 (28.98-29.02)
1970-1984	65-70	Any mental disorder	10,490	30.57 (30.53-30.60)	4,033	25.28 (25.24-25.32)	6,457	35.16 (35.11-35.21)
1985-1994	65-70	Any mental disorder	4,021	18.05 (18.03-18.08)	1,536	14.75 (14.72-14.78)	2,485	20.95 (20.92-20.99)
1995-2004	65-70	Any mental disorder	6,422	31.56 (31.52-31.60)	2,774	28.53 (28.48-28.59)	3,648	34.33 (34.27-34.40)
2005-2016	65-70	Any mental disorder	7,678	24.20 (24.17-24.22)	3,719	23.97 (23.93-24.01)	3,959	24.41 (24.37-24.45)
1970-2016	70-75	Any mental disorder	32,665	36.26 (36.24-36.28)	13,184	32.25 (32.22-32.28)	19,481	39.59 (39.56-39.63)
1970-1984	70-75	Any mental disorder	9,881	35.25 (35.20-35.29)	3,597	29.20 (29.15-29.26)	6,284	39.98 (39.92-40.04)
1985-1994	70-75	Any mental disorder	4,514	23.14 (23.10-23.17)	1,668	19.20 (19.16-19.24)	2,846	26.30 (26.25-26.35)
1995-2004	70-75	Any mental disorder	8,971	49.73 (49.66-49.80)	3,662	44.54 (44.45-44.64)	5,309	54.07 (53.97-54.18)
2005-2016	70-75	Any mental disorder	9,299	37.96 (37.91-38.00)	4,257	36.54 (36.47-36.60)	5,042	39.24 (39.18-39.31)
1970-2016	75-80	Any mental disorder	39,391	57.46 (57.41-57.50)	15,343	52.84 (52.78-52.90)	24,048	60.85 (60.79-60.91)
1970-1984	75-80	Any mental disorder	9,231	46.41 (46.35-46.48)	3,403	41.81 (41.72-41.90)	5,828	49.60 (49.51-49.69)
1985-1994	75-80	Any mental disorder	4,725	30.29 (30.24-30.34)	1,775	27.75 (27.69-27.82)	2,950	32.06 (31.99-32.12)
1995-2004	75-80	Any mental disorder	12,786	85.06 (84.93-85.20)	4,829	76.15 (75.96-76.34)	7,957	91.57 (91.38-91.76)

			Both sexes		Males		Females	
Years	Age	Mental disorder	Cases	IR (95% CI)	Cases	IR (95% CI)	Cases	IR (95% CI)
2005-2016	75-80	Any mental disorder	12,649	70.12 (70.02-70.22)	5,336	65.39 (65.25-65.53)	7,313	74.03 (73.88-74.17)
1970-2016	80-85	Any mental disorder	40,120	88.15 (88.07-88.23)	14,715	84.12 (83.99-84.24)	25,405	90.67 (90.56-90.77)
1970-1984	80-85	Any mental disorder	7,003	59.23 (59.12-59.34)	2,574	56.73 (56.57-56.90)	4,429	60.78 (60.64-60.92)
1985-1994	80-85	Any mental disorder	3,984	38.14 (38.07-38.21)	1,405	37.43 (37.31-37.55)	2,579	38.54 (38.45-38.63)
1995-2004	80-85	Any mental disorder	13,540	127.97 (127.73-128.21)	4,780	120.26 (119.89-120.63)	8,760	132.61 (132.29-132.93)
2005-2016	80-85	Any mental disorder	15,593	123.12 (122.90-123.33)	5,956	113.92 (113.61-114.23)	9,637	129.58 (129.29-129.88)
1970-2016	85-90	Any mental disorder	28,494	118.26 (118.11-118.41)	9,454	116.11 (115.86-116.36)	19,040	119.36 (119.18-119.55)
1970-1984	85-90	Any mental disorder	3,686	68.99 (68.80-69.17)	1,465	75.35 (75.01-75.68)	2,221	65.35 (65.13-65.57)
1985-1994	85-90	Any mental disorder	2,215	42.19 (42.08-42.30)	681	41.65 (41.45-41.86)	1,534	42.43 (42.29-42.57)
1995-2004	85-90	Any mental disorder	10,176	170.99 (170.55-171.42)	3,119	164.63 (163.89-165.37)	7,057	173.96 (173.42-174.49)
2005-2016	85-90	Any mental disorder	12,417	164.48 (164.11-164.86)	4,189	156.98 (156.38-157.57)	8,228	168.59 (168.12-169.06)
1970-2016	90-95	Any mental disorder	10,443	119.13 (118.88-119.38)	2,905	116.31 (115.85-116.76)	7,538	120.26 (119.96-120.56)
1970-1984	90-95	Any mental disorder	919	59.50 (59.20-59.80)	355	66.01 (65.45-66.57)	564	56.02 (55.68-56.37)
1985-1994	90-95	Any mental disorder	552	30.76 (30.62-30.90)	195	39.37 (39.02-39.72)	357	27.48 (27.33-27.63)
1995-2004	90-95	Any mental disorder	3,701	163.16 (162.49-163.83)	942	163.42 (162.09-164.76)	2,759	163.07 (162.29-163.85)
2005-2016	90-95	Any mental disorder	5,271	166.89 (166.31-167.47)	1,413	159.10 (158.05-160.15)	3,858	169.94 (169.24-170.64)
1970-2016	95-100	Any mental disorder	1,992	108.58 (108.09-109.08)	441	104.33 (103.34-105.33)	1,551	109.86 (109.29-110.43)
1970-1984	95-100	Any mental disorder	102	41.58 (41.06-42.11)	51	64.51 (63.10-65.94)	51	30.68 (30.22-31.15)
1985-1994	95-100	Any mental disorder	73	21.40 (21.17-21.63)	25	29.34 (28.72-29.97)	48	18.75 (18.53-18.98)
1995-2004	95-100	Any mental disorder	670	142.62 (141.34-143.92)	124	130.82 (128.22-133.48)	546	145.60 (144.14-147.08)
2005-2016	95-100	Any mental disorder	1,147	147.38 (146.35-148.42)	241	147.27 (145.03-149.54)	906	147.41 (146.25-148.58)
1970-2016	1-5	Any mental disorder (inpatient)	1,168	0.96 (0.96-0.96)	828	1.33 (1.33-1.33)	340	0.58 (0.57-0.58)
1970-1984	1-5	Any mental disorder (inpatient)	305	0.75 (0.75-0.75)	193	0.93 (0.93-0.93)	112	0.56 (0.56-0.56)
1985-1994	1-5	Any mental disorder (inpatient)	380	1.66 (1.65-1.66)	264	2.24 (2.24-2.25)	116	1.04 (1.03-1.04)
1995-2004	1-5	Any mental disorder (inpatient)	421	1.55 (1.55-1.55)	323	2.32 (2.32-2.33)	98	0.74 (0.74-0.74)
2005-2016	1-5	Any mental disorder (inpatient)	62	0.20 (0.20-0.20)	48	0.31 (0.31-0.31)	14	0.09 (0.09-0.09)
1970-2016	5-10	Any mental disorder (inpatient)	4,811	3.09 (3.09-3.09)	3,708	4.65 (4.65-4.65)	1,103	1.45 (1.45-1.45)
1970-1984	5-10	Any mental disorder (inpatient)	1,623	2.92 (2.91-2.92)	1,241	4.36 (4.35-4.36)	382	1.41 (1.40-1.41)
1985-1994	5-10	Any mental disorder (inpatient)	1,291	4.62 (4.61-4.62)	999	7.00 (6.98-7.01)	292	2.13 (2.13-2.14)
1995-2004	5-10	Any mental disorder (inpatient)	1,394	4.24 (4.24-4.25)	1,102	6.54 (6.53-6.55)	292	1.82 (1.82-1.83)
2005-2016	5-10	Any mental disorder (inpatient)	503	1.28 (1.28-1.28)	366	1.82 (1.82-1.82)	137	0.71 (0.71-0.72)
1970-2016	10-15	Any mental disorder (inpatient)	9,257	5.87 (5.86-5.87)	4,573	5.67 (5.66-5.67)	4,684	6.08 (6.07-6.08)
1970-1984	10-15	Any mental disorder (inpatient)	2,437	4.29 (4.28-4.29)	1,437	4.94 (4.93-4.95)	1,000	3.60 (3.60-3.60)
1985-1994	10-15	Any mental disorder (inpatient)	1,551	4.93 (4.93-4.94)	899	5.61 (5.60-5.62)	652	4.23 (4.22-4.24)
1995-2004	10-15	Any mental disorder (inpatient)	1,893	6.43 (6.42-6.44)	941	6.25 (6.24-6.26)	952	6.62 (6.61-6.63)
2005-2016	10-15	Any mental disorder (inpatient)	3,376	8.43 (8.43-8.44)	1,296	6.32 (6.32-6.33)	2,080	10.65 (10.63-10.66)
1970-2016	15-20	Any mental disorder (inpatient)	31,833	19.96 (19.95-19.97)	13,705	16.75 (16.74-16.76)	18,128	23.35 (23.33-23.37)
1970-1984	15-20	Any mental disorder (inpatient)	10,409	18.33 (18.32-18.35)	5,167	17.73 (17.71-17.75)	5,242	18.98 (18.95-19.00)
1985-1994	15-20	Any mental disorder (inpatient)	3,860	11.00 (10.98-11.01)	1,805	10.03 (10.02-10.05)	2,055	12.01 (11.99-12.03)
1995-2004	15-20	Any mental disorder (inpatient)	5,566	19.74 (19.71-19.76)	2,165	15.01 (14.98-15.03)	3,401	24.70 (24.65-24.74)

			Both sexes		Males		Females	
Years	Age	Mental disorder	Cases	IR (95% CI)	Cases	IR (95% CI)	Cases	IR (95% CI)
2005-2016	15-20	Any mental disorder (inpatient)	11,998	30.46 (30.43-30.49)	4,568	22.55 (22.52-22.58)	7,430	38.84 (38.79-38.90)
1970-2016	20-25	Any mental disorder (inpatient)	44,553	28.11 (28.09-28.12)	22,876	27.91 (27.89-27.93)	21,677	28.31 (28.29-28.33)
1970-1984	20-25	Any mental disorder (inpatient)	17,117	31.32 (31.29-31.34)	9,340	33.11 (33.07-33.15)	7,777	29.40 (29.37-29.44)
1985-1994	20-25	Any mental disorder (inpatient)	6,778	18.41 (18.39-18.43)	3,648	19.05 (19.02-19.07)	3,130	17.72 (17.69-17.74)
1995-2004	20-25	Any mental disorder (inpatient)	7,751	25.41 (25.39-25.44)	3,718	23.57 (23.54-23.61)	4,033	27.39 (27.34-27.43)
2005-2016	20-25	Any mental disorder (inpatient)	12,907	35.32 (35.28-35.35)	6,170	32.77 (32.72-32.81)	6,737	38.03 (37.97-38.08)
1970-2016	25-30	Any mental disorder (inpatient)	41,674	26.39 (26.37-26.40)	21,227	26.08 (26.07-26.10)	20,447	26.71 (26.69-26.73)
1970-1984	25-30	Any mental disorder (inpatient)	18,436	33.26 (33.24-33.29)	9,224	32.38 (32.35-32.42)	9,212	34.19 (34.15-34.23)
1985-1994	25-30	Any mental disorder (inpatient)	7,180	19.94 (19.92-19.96)	3,758	20.14 (20.11-20.17)	3,422	19.72 (19.69-19.75)
1995-2004	25-30	Any mental disorder (inpatient)	7,342	22.28 (22.26-22.31)	3,720	21.83 (21.80-21.86)	3,622	22.77 (22.74-22.81)
2005-2016	25-30	Any mental disorder (inpatient)	8,716	25.99 (25.96-26.01)	4,525	26.32 (26.28-26.36)	4,191	25.64 (25.60-25.68)
1970-2016	30-35	Any mental disorder (inpatient)	41,448	26.65 (26.64-26.66)	20,791	26.08 (26.06-26.10)	20,657	27.25 (27.23-27.27)
1970-1984	30-35	Any mental disorder (inpatient)	19,191	36.33 (36.30-36.36)	9,224	34.23 (34.19-34.27)	9,967	38.52 (38.48-38.57)
1985-1994	30-35	Any mental disorder (inpatient)	7,397	22.18 (22.16-22.20)	3,830	22.32 (22.29-22.36)	3,567	22.03 (21.99-22.06)
1995-2004	30-35	Any mental disorder (inpatient)	7,271	20.77 (20.75-20.79)	3,728	20.65 (20.62-20.68)	3,543	20.89 (20.86-20.92)
2005-2016	30-35	Any mental disorder (inpatient)	7,589	22.10 (22.07-22.12)	4,009	22.84 (22.80-22.87)	3,580	21.32 (21.29-21.36)
1970-2016	35-40	Any mental disorder (inpatient)	40,640	26.57 (26.56-26.59)	19,897	25.54 (25.52-25.55)	20,743	27.65 (27.63-27.67)
1970-1984	35-40	Any mental disorder (inpatient)	18,893	39.43 (39.40-39.47)	8,668	35.67 (35.62-35.71)	10,225	43.31 (43.26-43.37)
1985-1994	35-40	Any mental disorder (inpatient)	7,300	21.91 (21.89-21.94)	3,647	21.52 (21.48-21.55)	3,653	22.33 (22.29-22.36)
1995-2004	35-40	Any mental disorder (inpatient)	7,180	20.76 (20.74-20.79)	3,725	21.03 (21.00-21.07)	3,455	20.48 (20.45-20.51)
2005-2016	35-40	Any mental disorder (inpatient)	7,267	19.57 (19.55-19.59)	3,857	20.35 (20.32-20.38)	3,410	18.76 (18.73-18.78)
1970-2016	40-45	Any mental disorder (inpatient)	38,305	25.74 (25.73-25.75)	18,383	24.39 (24.37-24.41)	19,922	27.13 (27.11-27.15)
1970-1984	40-45	Any mental disorder (inpatient)	17,621	42.04 (42.00-42.08)	7,879	37.42 (37.37-37.47)	9,742	46.71 (46.65-46.78)
1985-1994	40-45	Any mental disorder (inpatient)	7,106	20.34 (20.32-20.36)	3,354	18.97 (18.94-18.99)	3,752	21.75 (21.72-21.78)
1995-2004	40-45	Any mental disorder (inpatient)	6,275	19.52 (19.50-19.55)	3,169	19.38 (19.35-19.41)	3,106	19.68 (19.65-19.71)
2005-2016	40-45	Any mental disorder (inpatient)	7,303	18.34 (18.32-18.36)	3,981	19.64 (19.61-19.66)	3,322	16.99 (16.97-17.01)
1970-2016	45-50	Any mental disorder (inpatient)	36,732	25.49 (25.48-25.51)	17,311	23.88 (23.86-23.89)	19,421	27.13 (27.11-27.15)
1970-1984	45-50	Any mental disorder (inpatient)	17,869	44.09 (44.05-44.14)	7,848	38.92 (38.87-38.98)	10,021	49.21 (49.14-49.28)
1985-1994	45-50	Any mental disorder (inpatient)	6,214	19.47 (19.44-19.49)	2,878	17.85 (17.82-17.87)	3,336	21.12 (21.09-21.15)
1995-2004	45-50	Any mental disorder (inpatient)	5,474	17.13 (17.11-17.15)	2,691	16.68 (16.66-16.71)	2,783	17.58 (17.55-17.61)
2005-2016	45-50	Any mental disorder (inpatient)	7,175	18.08 (18.07-18.10)	3,894	19.39 (19.36-19.42)	3,281	16.74 (16.72-16.77)
1970-2016	50-55	Any mental disorder (inpatient)	32,550	23.66 (23.65-23.68)	14,640	21.29 (21.28-21.31)	17,910	26.03 (26.02-26.05)
1970-1984	50-55	Any mental disorder (inpatient)	17,092	41.92 (41.88-41.96)	7,179	35.63 (35.58-35.68)	9,913	48.07 (48.00-48.13)
1985-1994	50-55	Any mental disorder (inpatient)	4,736	17.99 (17.97-18.01)	2,004	15.21 (15.19-15.24)	2,732	20.77 (20.73-20.80)
1995-2004	50-55	Any mental disorder (inpatient)	4,913	14.82 (14.80-14.83)	2,326	13.96 (13.94-13.98)	2,587	15.68 (15.66-15.70)
2005-2016	50-55	Any mental disorder (inpatient)	5,809	15.58 (15.56-15.59)	3,131	16.68 (16.65-16.70)	2,678	14.46 (14.44-14.49)
1970-2016	55-60	Any mental disorder (inpatient)	27,187	20.91 (20.90-20.92)	12,165	18.89 (18.87-18.90)	15,022	22.89 (22.87-22.91)
1970-1984	55-60	Any mental disorder (inpatient)	14,700	36.42 (36.38-36.46)	6,085	30.79 (30.75-30.83)	8,615	41.82 (41.76-41.88)
1985-1994	55-60	Any mental disorder (inpatient)	3,906	16.38 (16.36-16.40)	1,649	14.02 (14.00-14.05)	2,257	18.68 (18.64-18.71)
1995-2004	55-60	Any mental disorder (inpatient)	3,869	12.97 (12.96-12.99)	1,843	12.36 (12.34-12.38)	2,026	13.59 (13.56-13.61)

			Both sexes		Males		Females	
**Years**	**Age**	**Mental disorder**	**Cases**	**IR (95% CI)**	**Cases**	**IR (95% CI)**	**Cases**	**IR (95% CI)**
2005-2016	55-60	Any mental disorder (inpatient)	4,712	13.09 (13.08-13.11)	2,588	14.40 (14.38-14.43)	2,124	11.78 (11.77-11.80)
1970-2016	60-65	Any mental disorder (inpatient)	22,872	18.78 (18.77-18.79)	9,892	16.66 (16.65-16.68)	12,980	20.80 (20.78-20.82)
1970-1984	60-65	Any mental disorder (inpatient)	12,119	31.41 (31.38-31.44)	4,845	26.09 (26.05-26.13)	7,274	36.34 (36.29-36.39)
1985-1994	60-65	Any mental disorder (inpatient)	3,818	16.47 (16.45-16.49)	1,523	13.61 (13.59-13.64)	2,295	19.13 (19.10-19.17)
1995-2004	60-65	Any mental disorder (inpatient)	2,963	12.41 (12.39-12.42)	1,410	12.02 (12.00-12.04)	1,553	12.78 (12.76-12.80)
2005-2016	60-65	Any mental disorder (inpatient)	3,972	11.00 (10.98-11.01)	2,114	11.83 (11.81-11.84)	1,858	10.18 (10.17-10.20)
1970-2016	65-70	Any mental disorder (inpatient)	20,637	18.85 (18.84-18.86)	8,448	16.26 (16.25-16.28)	12,189	21.19 (21.17-21.21)
1970-1984	65-70	Any mental disorder (inpatient)	10,490	30.57 (30.53-30.60)	4,033	25.28 (25.24-25.32)	6,457	35.16 (35.11-35.21)
1985-1994	65-70	Any mental disorder (inpatient)	3,879	17.41 (17.39-17.44)	1,485	14.26 (14.23-14.29)	2,394	20.19 (20.15-20.22)
1995-2004	65-70	Any mental disorder (inpatient)	2,880	14.06 (14.04-14.08)	1,213	12.40 (12.38-12.43)	1,667	15.57 (15.54-15.60)
2005-2016	65-70	Any mental disorder (inpatient)	3,388	10.46 (10.45-10.47)	1,717	10.87 (10.85-10.89)	1,671	10.07 (10.06-10.09)
1970-2016	70-75	Any mental disorder (inpatient)	20,654	22.75 (22.74-22.77)	8,090	19.66 (19.64-19.67)	12,564	25.32 (25.30-25.35)
1970-1984	70-75	Any mental disorder (inpatient)	9,881	35.25 (35.20-35.29)	3,597	29.20 (29.15-29.26)	6,284	39.98 (39.92-40.04)
1985-1994	70-75	Any mental disorder (inpatient)	4,282	21.95 (21.91-21.98)	1,590	18.30 (18.26-18.34)	2,692	24.87 (24.82-24.92)
1995-2004	70-75	Any mental disorder (inpatient)	3,286	18.04 (18.02-18.07)	1,358	16.39 (16.35-16.42)	1,928	19.42 (19.38-19.46)
2005-2016	70-75	Any mental disorder (inpatient)	3,205	12.81 (12.80-12.83)	1,545	13.02 (13.00-13.04)	1,660	12.63 (12.61-12.65)
1970-2016	75-80	Any mental disorder (inpatient)	20,950	30.21 (30.19-30.23)	8,227	28.05 (28.02-28.08)	12,723	31.79 (31.76-31.82)
1970-1984	75-80	Any mental disorder (inpatient)	9,231	46.41 (46.35-46.48)	3,403	41.81 (41.72-41.90)	5,828	49.60 (49.51-49.69)
1985-1994	75-80	Any mental disorder (inpatient)	4,455	28.56 (28.51-28.60)	1,705	26.66 (26.59-26.72)	2,750	29.88 (29.82-29.94)
1995-2004	75-80	Any mental disorder (inpatient)	4,044	26.46 (26.42-26.50)	1,635	25.45 (25.38-25.51)	2,409	27.20 (27.14-27.25)
2005-2016	75-80	Any mental disorder (inpatient)	3,220	17.33 (17.31-17.36)	1,484	17.74 (17.70-17.78)	1,736	17.00 (16.97-17.03)
1970-2016	80-85	Any mental disorder (inpatient)	17,113	36.85 (36.82-36.88)	6,587	37.04 (36.98-37.09)	10,526	36.73 (36.69-36.78)
1970-1984	80-85	Any mental disorder (inpatient)	7,003	59.23 (59.12-59.34)	2,574	56.73 (56.57-56.90)	4,429	60.78 (60.64-60.92)
1985-1994	80-85	Any mental disorder (inpatient)	3,631	34.75 (34.69-34.82)	1,307	34.81 (34.70-34.92)	2,324	34.72 (34.64-34.81)
1995-2004	80-85	Any mental disorder (inpatient)	3,481	32.04 (31.98-32.10)	1,384	34.12 (34.02-34.23)	2,097	30.80 (30.73-30.87)
2005-2016	80-85	Any mental disorder (inpatient)	2,998	22.53 (22.50-22.57)	1,322	24.31 (24.25-24.38)	1,676	21.30 (21.26-21.35)
1970-2016	85-90	Any mental disorder (inpatient)	10,044	40.33 (40.28-40.38)	3,730	44.67 (44.57-44.76)	6,314	38.14 (38.08-38.20)
1970-1984	85-90	Any mental disorder (inpatient)	3,686	68.99 (68.80-69.17)	1,465	75.35 (75.01-75.68)	2,221	65.35 (65.13-65.57)
1985-1994	85-90	Any mental disorder (inpatient)	1,985	37.80 (37.70-37.90)	637	38.96 (38.77-39.15)	1,348	37.27 (37.15-37.40)
1995-2004	85-90	Any mental disorder (inpatient)	2,294	37.08 (36.99-37.17)	831	42.60 (42.41-42.79)	1,463	34.54 (34.43-34.64)
2005-2016	85-90	Any mental disorder (inpatient)	2,079	25.59 (25.53-25.65)	797	28.26 (28.15-28.36)	1,282	24.17 (24.11-24.24)
1970-2016	90-95	Any mental disorder (inpatient)	2,993	32.71 (32.64-32.77)	1,030	40.02 (39.86-40.17)	1,963	29.84 (29.77-29.92)
1970-1984	90-95	Any mental disorder (inpatient)	919	59.50 (59.20-59.80)	355	66.01 (65.45-66.57)	564	56.02 (55.68-56.37)
1985-1994	90-95	Any mental disorder (inpatient)	487	27.13 (27.00-27.25)	180	36.33 (36.01-36.65)	307	23.62 (23.49-23.75)
1995-2004	90-95	Any mental disorder (inpatient)	740	31.23 (31.10-31.35)	227	38.08 (37.78-38.39)	513	28.92 (28.79-29.06)
2005-2016	90-95	Any mental disorder (inpatient)	847	24.61 (24.53-24.69)	268	28.37 (28.19-28.55)	579	23.19 (23.10-23.28)
1970-2016	95-100	Any mental disorder (inpatient)	465	24.22 (24.11-24.33)	141	32.39 (32.09-32.70)	324	21.82 (21.71-21.93)
1970-1984	95-100	Any mental disorder (inpatient)	102	41.58 (41.06-42.11)	51	64.51 (63.10-65.94)	51	30.68 (30.22-31.15)
1985-1994	95-100	Any mental disorder (inpatient)	62	18.17 (17.98-18.36)	23	26.99 (26.42-27.56)	39	15.23 (15.05-15.42)
1995-2004	95-100	Any mental disorder (inpatient)	107	21.88 (21.68-22.07)	24	24.59 (24.10-25.08)	83	21.20 (20.99-21.41)

			Both sexes		Males		Females	
**Years**	**Age**	**Mental disorder**	**Cases**	**IR (95% CI)**	**Cases**	**IR (95% CI)**	**Cases**	**IR (95% CI)**
2005-2016	95-100	Any mental disorder (inpatient)	194	22.97 (22.82-23.13)	43	24.80 (24.43-25.17)	151	22.50 (22.33-22.67)
1970-2016	35-40	Organic disorders	5,937	3.73 (3.73-3.73)	3,346	4.13 (4.13-4.13)	2,591	3.31 (3.31-3.31)
1970-1984	35-40	Organic disorders	2,962	5.99 (5.98-5.99)	1,518	6.07 (6.06-6.07)	1,444	5.91 (5.90-5.91)
1985-1994	35-40	Organic disorders	1,111	3.17 (3.17-3.18)	624	3.51 (3.50-3.51)	487	2.83 (2.82-2.83)
1995-2004	35-40	Organic disorders	901	2.50 (2.50-2.50)	587	3.18 (3.18-3.19)	314	1.78 (1.78-1.79)
2005-2016	35-40	Organic disorders	963	2.49 (2.48-2.49)	617	3.12 (3.12-3.13)	346	1.82 (1.82-1.83)
1970-2016	40-45	Organic disorders	5,133	3.30 (3.29-3.30)	2,829	3.60 (3.59-3.60)	2,304	2.99 (2.99-2.99)
1970-1984	40-45	Organic disorders	2,526	5.83 (5.82-5.84)	1,254	5.79 (5.78-5.79)	1,272	5.87 (5.87-5.88)
1985-1994	40-45	Organic disorders	997	2.71 (2.70-2.71)	558	3.00 (3.00-3.01)	439	2.40 (2.40-2.41)
1995-2004	40-45	Organic disorders	771	2.27 (2.27-2.28)	487	2.83 (2.82-2.83)	284	1.70 (1.70-1.71)
2005-2016	40-45	Organic disorders	839	2.01 (2.01-2.01)	530	2.50 (2.50-2.50)	309	1.51 (1.51-1.51)
1970-2016	45-50	Organic disorders	5,992	3.96 (3.96-3.96)	3,303	4.35 (4.35-4.36)	2,689	3.56 (3.56-3.56)
1970-1984	45-50	Organic disorders	2,977	7.11 (7.10-7.11)	1,596	7.69 (7.68-7.70)	1,381	6.53 (6.52-6.54)
1985-1994	45-50	Organic disorders	960	2.85 (2.84-2.85)	504	2.98 (2.97-2.98)	456	2.71 (2.71-2.72)
1995-2004	45-50	Organic disorders	1,010	2.98 (2.97-2.98)	604	3.54 (3.53-3.54)	406	2.41 (2.40-2.41)
2005-2016	45-50	Organic disorders	1,045	2.50 (2.50-2.50)	599	2.83 (2.83-2.84)	446	2.16 (2.15-2.16)
1970-2016	50-55	Organic disorders	7,039	4.86 (4.86-4.86)	3,814	5.30 (5.30-5.30)	3,225	4.43 (4.42-4.43)
1970-1984	50-55	Organic disorders	3,534	8.39 (8.38-8.40)	1,868	9.02 (9.01-9.03)	1,666	7.78 (7.77-7.79)
1985-1994	50-55	Organic disorders	978	3.51 (3.51-3.52)	507	3.67 (3.67-3.68)	471	3.35 (3.35-3.36)
1995-2004	50-55	Organic disorders	1,219	3.46 (3.46-3.46)	675	3.84 (3.84-3.85)	544	3.08 (3.08-3.09)
2005-2016	50-55	Organic disorders	1,308	3.30 (3.30-3.30)	764	3.84 (3.84-3.85)	544	2.76 (2.75-2.76)
1970-2016	55-60	Organic disorders	7,956	5.82 (5.81-5.82)	4,420	6.57 (6.57-6.58)	3,536	5.08 (5.08-5.09)
1970-1984	55-60	Organic disorders	3,867	9.29 (9.28-9.30)	2,153	10.63 (10.62-10.65)	1,714	8.02 (8.01-8.03)
1985-1994	55-60	Organic disorders	1,024	4.07 (4.06-4.07)	510	4.15 (4.14-4.16)	514	3.98 (3.98-3.99)
1995-2004	55-60	Organic disorders	1,435	4.53 (4.53-4.54)	795	5.07 (5.06-5.08)	640	4.00 (4.00-4.01)
2005-2016	55-60	Organic disorders	1,630	4.25 (4.25-4.25)	962	5.05 (5.05-5.06)	668	3.46 (3.45-3.46)
1970-2016	60-65	Organic disorders	8,910	6.98 (6.97-6.98)	4,652	7.54 (7.54-7.55)	4,258	6.45 (6.44-6.45)
1970-1984	60-65	Organic disorders	3,773	9.53 (9.52-9.54)	1,984	10.48 (10.46-10.49)	1,789	8.66 (8.65-8.67)
1985-1994	60-65	Organic disorders	1,218	4.99 (4.98-5.00)	552	4.74 (4.73-4.75)	666	5.21 (5.21-5.22)
1995-2004	60-65	Organic disorders	1,747	6.91 (6.90-6.91)	920	7.50 (7.48-7.51)	827	6.35 (6.34-6.36)
2005-2016	60-65	Organic disorders	2,172	5.66 (5.65-5.66)	1,196	6.35 (6.34-6.36)	976	4.99 (4.98-5.00)
1970-2016	65-70	Organic disorders	12,862	11.26 (11.25-11.26)	6,277	11.70 (11.69-11.71)	6,585	10.87 (10.86-10.88)
1970-1984	65-70	Organic disorders	4,496	12.82 (12.81-12.84)	2,186	13.49 (13.47-13.51)	2,310	12.25 (12.23-12.26)
1985-1994	65-70	Organic disorders	1,635	7.01 (7.00-7.02)	752	6.98 (6.97-7.00)	883	7.03 (7.01-7.04)
1995-2004	65-70	Organic disorders	2,923	13.53 (13.51-13.54)	1,408	13.84 (13.81-13.87)	1,515	13.25 (13.22-13.27)
2005-2016	65-70	Organic disorders	3,808	11.12 (11.11-11.14)	1,931	11.69 (11.68-11.71)	1,877	10.59 (10.58-10.61)
1970-2016	70-75	Organic disorders	20,668	21.95 (21.93-21.96)	9,136	21.63 (21.61-21.66)	11,532	22.20 (22.18-22.22)
1970-1984	70-75	Organic disorders	5,965	20.89 (20.87-20.91)	2,570	20.61 (20.57-20.64)	3,395	21.11 (21.08-21.14)
1985-1994	70-75	Organic disorders	2,489	12.26 (12.24-12.28)	1,041	11.67 (11.65-11.69)	1,448	12.72 (12.70-12.75)
1995-2004	70-75	Organic disorders	5,672	29.72 (29.68-29.76)	2,505	29.28 (29.22-29.34)	3,167	30.08 (30.02-30.14)

			Both sexes		Males		Females	
**Years**	**Age**	**Mental disorder**	**Cases**	**IR (95% CI)**	**Cases**	**IR (95% CI)**	**Cases**	**IR (95% CI)**
2005-2016	70-75	Organic disorders	6,542	24.93 (24.90-24.96)	3,020	24.59 (24.55-24.63)	3,522	25.23 (25.19-25.27)
1970-2016	75-80	Organic disorders	30,201	42.32 (42.29-42.35)	12,328	41.31 (41.26-41.36)	17,873	43.04 (43.00-43.09)
1970-1984	75-80	Organic disorders	7,004	34.67 (34.63-34.72)	2,803	34.10 (34.03-34.17)	4,201	35.07 (35.00-35.13)
1985-1994	75-80	Organic disorders	3,246	20.14 (20.11-20.17)	1,380	21.15 (21.10-21.20)	1,866	19.45 (19.41-19.49)
1995-2004	75-80	Organic disorders	9,467	59.72 (59.63-59.82)	3,731	56.82 (56.68-56.96)	5,736	61.78 (61.65-61.91)
2005-2016	75-80	Organic disorders	10,484	54.62 (54.54-54.70)	4,414	51.73 (51.62-51.84)	6,070	56.93 (56.82-57.04)
1970-2016	80-85	Organic disorders	33,930	71.81 (71.75-71.88)	12,823	71.58 (71.47-71.68)	21,107	71.96 (71.88-72.04)
1970-1984	80-85	Organic disorders	6,133	51.21 (51.12-51.31)	2,362	51.66 (51.51-51.81)	3,771	50.94 (50.82-51.05)
1985-1994	80-85	Organic disorders	2,979	27.76 (27.70-27.81)	1,153	30.25 (30.15-30.35)	1,826	26.38 (26.32-26.45)
1995-2004	80-85	Organic disorders	10,859	97.74 (97.56-97.92)	4,009	97.92 (97.62-98.22)	6,850	97.63 (97.41-97.86)
2005-2016	80-85	Organic disorders	13,959	103.94 (103.77-104.12)	5,299	97.46 (97.20-97.72)	8,660	108.35 (108.11-108.59)
1970-2016	85-90	Organic disorders	25,085	100.41 (100.29-100.54)	8,502	102.21 (101.99-102.43)	16,583	99.52 (99.37-99.67)
1970-1984	85-90	Organic disorders	3,380	62.63 (62.47-62.80)	1,380	70.54 (70.23-70.85)	2,000	58.14 (57.95-58.33)
1985-1994	85-90	Organic disorders	1,818	33.87 (33.78-33.96)	605	36.57 (36.39-36.74)	1,213	32.67 (32.56-32.77)
1995-2004	85-90	Organic disorders	8,500	136.65 (136.32-136.99)	2,688	138.37 (137.76-138.99)	5,812	135.87 (135.47-136.28)
2005-2016	85-90	Organic disorders	11,387	142.37 (142.06-142.69)	3,829	138.47 (137.96-138.99)	7,558	144.43 (144.04-144.83)
1970-2016	90-95	Organic disorders	9,231	101.66 (101.45-101.87)	2,558	100.40 (100.01-100.79)	6,673	102.15 (101.90-102.40)
1970-1984	90-95	Organic disorders	860	55.23 (54.95-55.50)	337	62.35 (61.82-62.87)	523	51.44 (51.13-51.76)
1985-1994	90-95	Organic disorders	449	24.61 (24.50-24.72)	166	33.18 (32.89-33.47)	283	21.37 (21.26-21.49)
1995-2004	90-95	Organic disorders	3,062	129.89 (129.37-130.42)	802	136.16 (135.06-137.26)	2,260	127.80 (127.21-128.40)
2005-2016	90-95	Organic disorders	4,860	145.45 (144.95-145.94)	1,253	136.51 (135.63-137.39)	3,607	148.83 (148.24-149.42)
1970-2016	95-100	Organic disorders	1,763	92.96 (92.55-93.38)	387	89.91 (89.07-90.77)	1,376	93.86 (93.38-94.34)
1970-1984	95-100	Organic disorders	98	39.66 (39.17-40.16)	50	62.94 (61.57-64.34)	48	28.63 (28.20-29.07)
1985-1994	95-100	Organic disorders	60	17.38 (17.20-17.57)	23	26.80 (26.24-27.37)	37	14.26 (14.09-14.44)
1995-2004	95-100	Organic disorders	557	114.83 (113.81-115.85)	103	106.76 (104.65-108.91)	454	116.83 (115.67-118.00)
2005-2016	95-100	Organic disorders	1,048	127.95 (127.08-128.83)	211	125.09 (123.22-127.00)	837	128.69 (127.71-129.68)
1970-2016	35-40	Organic disorders (inpatient)	4,954	3.11 (3.11-3.11)	2,733	3.37 (3.37-3.38)	2,221	2.84 (2.84-2.84)
1970-1984	35-40	Organic disorders (inpatient)	2,962	5.99 (5.98-5.99)	1,518	6.07 (6.06-6.07)	1,444	5.91 (5.90-5.91)
1985-1994	35-40	Organic disorders (inpatient)	1,079	3.08 (3.08-3.08)	602	3.38 (3.38-3.39)	477	2.77 (2.77-2.77)
1995-2004	35-40	Organic disorders (inpatient)	462	1.28 (1.28-1.28)	302	1.64 (1.64-1.64)	160	0.91 (0.91-0.91)
2005-2016	35-40	Organic disorders (inpatient)	451	1.16 (1.16-1.17)	311	1.57 (1.57-1.58)	140	0.74 (0.74-0.74)
1970-2016	40-45	Organic disorders (inpatient)	4,331	2.78 (2.78-2.78)	2,335	2.97 (2.96-2.97)	1,996	2.59 (2.59-2.59)
1970-1984	40-45	Organic disorders (inpatient)	2,526	5.83 (5.82-5.84)	1,254	5.79 (5.78-5.79)	1,272	5.87 (5.87-5.88)
1985-1994	40-45	Organic disorders (inpatient)	964	2.62 (2.61-2.62)	544	2.93 (2.92-2.93)	420	2.30 (2.30-2.30)
1995-2004	40-45	Organic disorders (inpatient)	404	1.19 (1.19-1.19)	251	1.46 (1.46-1.46)	153	0.92 (0.92-0.92)
2005-2016	40-45	Organic disorders (inpatient)	437	1.05 (1.05-1.05)	286	1.35 (1.35-1.35)	151	0.74 (0.74-0.74)
1970-2016	45-50	Organic disorders (inpatient)	5,031	3.32 (3.32-3.32)	2,770	3.65 (3.65-3.65)	2,261	2.99 (2.99-2.99)
1970-1984	45-50	Organic disorders (inpatient)	2,977	7.11 (7.10-7.11)	1,596	7.69 (7.68-7.70)	1,381	6.53 (6.52-6.54)
1985-1994	45-50	Organic disorders (inpatient)	935	2.77 (2.77-2.78)	490	2.90 (2.89-2.90)	445	2.65 (2.64-2.65)
1995-2004	45-50	Organic disorders (inpatient)	547	1.61 (1.61-1.61)	339	1.99 (1.98-1.99)	208	1.23 (1.23-1.23)

			Both sexes		Males		Females	
**Years**	**Age**	**Mental disorder**	**Cases**	**IR (95% CI)**	**Cases**	**IR (95% CI)**	**Cases**	**IR (95% CI)**
2005-2016	45-50	Organic disorders (inpatient)	572	1.37 (1.36-1.37)	345	1.63 (1.63-1.63)	227	1.10 (1.09-1.10)
1970-2016	50-55	Organic disorders (inpatient)	5,839	4.03 (4.03-4.03)	3,160	4.39 (4.39-4.39)	2,679	3.67 (3.67-3.68)
1970-1984	50-55	Organic disorders (inpatient)	3,534	8.39 (8.38-8.40)	1,868	9.02 (9.01-9.03)	1,666	7.78 (7.77-7.79)
1985-1994	50-55	Organic disorders (inpatient)	954	3.43 (3.42-3.43)	493	3.57 (3.57-3.58)	461	3.28 (3.28-3.29)
1995-2004	50-55	Organic disorders (inpatient)	647	1.84 (1.83-1.84)	359	2.04 (2.04-2.04)	288	1.63 (1.63-1.63)
2005-2016	50-55	Organic disorders (inpatient)	704	1.77 (1.77-1.78)	440	2.21 (2.21-2.21)	264	1.34 (1.33-1.34)
1970-2016	55-60	Organic disorders (inpatient)	6,415	4.69 (4.68-4.69)	3,548	5.27 (5.27-5.28)	2,867	4.12 (4.11-4.12)
1970-1984	55-60	Organic disorders (inpatient)	3,867	9.29 (9.28-9.30)	2,153	10.63 (10.62-10.65)	1,714	8.02 (8.01-8.03)
1985-1994	55-60	Organic disorders (inpatient)	993	3.94 (3.94-3.95)	497	4.05 (4.04-4.05)	496	3.84 (3.84-3.85)
1995-2004	55-60	Organic disorders (inpatient)	730	2.30 (2.30-2.31)	394	2.51 (2.51-2.52)	336	2.10 (2.10-2.10)
2005-2016	55-60	Organic disorders (inpatient)	825	2.15 (2.14-2.15)	504	2.64 (2.64-2.65)	321	1.66 (1.66-1.66)
1970-2016	60-65	Organic disorders (inpatient)	6,845	5.36 (5.35-5.36)	3,572	5.79 (5.78-5.79)	3,273	4.95 (4.95-4.96)
1970-1984	60-65	Organic disorders (inpatient)	3,773	9.53 (9.52-9.54)	1,984	10.48 (10.46-10.49)	1,789	8.66 (8.65-8.67)
1985-1994	60-65	Organic disorders (inpatient)	1,178	4.83 (4.82-4.83)	542	4.66 (4.65-4.66)	636	4.98 (4.97-4.99)
1995-2004	60-65	Organic disorders (inpatient)	831	3.28 (3.28-3.28)	444	3.61 (3.61-3.62)	387	2.97 (2.96-2.97)
2005-2016	60-65	Organic disorders (inpatient)	1,063	2.76 (2.76-2.76)	602	3.19 (3.18-3.19)	461	2.35 (2.35-2.36)
1970-2016	65-70	Organic disorders (inpatient)	8,540	7.46 (7.46-7.47)	4,170	7.76 (7.75-7.77)	4,370	7.20 (7.20-7.21)
1970-1984	65-70	Organic disorders (inpatient)	4,496	12.82 (12.81-12.84)	2,186	13.49 (13.47-13.51)	2,310	12.25 (12.23-12.26)
1985-1994	65-70	Organic disorders (inpatient)	1,560	6.69 (6.68-6.69)	715	6.64 (6.63-6.65)	845	6.72 (6.71-6.73)
1995-2004	65-70	Organic disorders (inpatient)	1,101	5.08 (5.08-5.09)	536	5.26 (5.25-5.27)	565	4.93 (4.92-4.94)
2005-2016	65-70	Organic disorders (inpatient)	1,383	4.03 (4.02-4.03)	733	4.42 (4.42-4.43)	650	3.66 (3.65-3.66)
1970-2016	70-75	Organic disorders (inpatient)	11,811	12.50 (12.49-12.51)	5,252	12.40 (12.39-12.41)	6,559	12.59 (12.57-12.60)
1970-1984	70-75	Organic disorders (inpatient)	5,965	20.89 (20.87-20.91)	2,570	20.61 (20.57-20.64)	3,395	21.11 (21.08-21.14)
1985-1994	70-75	Organic disorders (inpatient)	2,356	11.60 (11.59-11.62)	990	11.10 (11.08-11.12)	1,366	12.00 (11.98-12.02)
1995-2004	70-75	Organic disorders (inpatient)	1,775	9.26 (9.24-9.27)	820	9.54 (9.52-9.56)	955	9.03 (9.01-9.04)
2005-2016	70-75	Organic disorders (inpatient)	1,715	6.49 (6.48-6.49)	872	7.05 (7.03-7.06)	843	5.99 (5.98-6.00)
1970-2016	75-80	Organic disorders (inpatient)	14,723	20.48 (20.47-20.50)	6,234	20.75 (20.73-20.78)	8,489	20.29 (20.27-20.31)
1970-1984	75-80	Organic disorders (inpatient)	7,004	34.67 (34.63-34.72)	2,803	34.10 (34.03-34.17)	4,201	35.07 (35.00-35.13)
1985-1994	75-80	Organic disorders (inpatient)	3,047	18.90 (18.87-18.93)	1,322	20.26 (20.21-20.31)	1,725	17.98 (17.94-18.01)
1995-2004	75-80	Organic disorders (inpatient)	2,566	16.02 (16.00-16.04)	1,136	17.14 (17.10-17.19)	1,430	15.23 (15.20-15.26)
2005-2016	75-80	Organic disorders (inpatient)	2,106	10.78 (10.76-10.79)	973	11.22 (11.20-11.25)	1,133	10.42 (10.40-10.44)
1970-2016	80-85	Organic disorders (inpatient)	13,518	28.19 (28.17-28.22)	5,528	30.47 (30.42-30.51)	7,990	26.80 (26.77-26.84)
1970-1984	80-85	Organic disorders (inpatient)	6,133	51.21 (51.12-51.31)	2,362	51.66 (51.51-51.81)	3,771	50.94 (50.82-51.05)
1985-1994	80-85	Organic disorders (inpatient)	2,696	25.12 (25.07-25.16)	1,070	28.07 (27.98-28.16)	1,626	23.49 (23.43-23.54)
1995-2004	80-85	Organic disorders (inpatient)	2,481	21.92 (21.88-21.96)	1,083	26.05 (25.98-26.13)	1,398	19.52 (19.48-19.57)
2005-2016	80-85	Organic disorders (inpatient)	2,208	15.86 (15.83-15.88)	1,013	18.08 (18.03-18.13)	1,195	14.36 (14.33-14.39)
1970-2016	85-90	Organic disorders (inpatient)	8,369	32.65 (32.61-32.69)	3,255	38.33 (38.25-38.41)	5,114	29.83 (29.79-29.88)
1970-1984	85-90	Organic disorders (inpatient)	3,380	62.63 (62.47-62.80)	1,380	70.54 (70.23-70.85)	2,000	58.14 (57.95-58.33)
1985-1994	85-90	Organic disorders (inpatient)	1,634	30.43 (30.35-30.52)	566	34.21 (34.04-34.37)	1,068	28.75 (28.66-28.85)
1995-2004	85-90	Organic disorders (inpatient)	1,741	27.21 (27.14-27.27)	684	34.41 (34.26-34.56)	1,057	23.96 (23.89-24.03)

			Both sexes		Males		Females	
Years	Age	Mental disorder	Cases	IR (95% CI)	Cases	IR (95% CI)	Cases	IR (95% CI)
2005-2016	85-90	Organic disorders (inpatient)	1,614	19.05 (19.01-19.09)	625	21.60 (21.52-21.68)	989	17.73 (17.68-17.78)
1970-2016	90-95	Organic disorders (inpatient)	2,534	26.99 (26.94-27.05)	888	34.02 (33.89-34.15)	1,646	24.28 (24.23-24.34)
1970-1984	90-95	Organic disorders (inpatient)	860	55.23 (54.95-55.50)	337	62.35 (61.82-62.87)	523	51.44 (51.13-51.76)
1985-1994	90-95	Organic disorders (inpatient)	401	21.98 (21.88-22.08)	155	30.97 (30.70-31.25)	246	18.58 (18.48-18.68)
1995-2004	90-95	Organic disorders (inpatient)	595	24.44 (24.34-24.53)	190	31.43 (31.18-31.68)	405	22.13 (22.03-22.23)
2005-2016	90-95	Organic disorders (inpatient)	678	18.98 (18.92-19.05)	206	21.35 (21.22-21.48)	472	18.11 (18.04-18.18)
1970-2016	95-100	Organic disorders (inpatient)	391	19.93 (19.84-20.01)	119	27.03 (26.78-27.28)	272	17.87 (17.78-17.96)
1970-1984	95-100	Organic disorders (inpatient)	98	39.66 (39.17-40.16)	50	62.94 (61.57-64.34)	48	28.63 (28.20-29.07)
1985-1994	95-100	Organic disorders (inpatient)	49	14.19 (14.04-14.34)	21	24.47 (23.95-24.99)	28	10.79 (10.66-10.92)
1995-2004	95-100	Organic disorders (inpatient)	87	17.43 (17.27-17.58)	19	19.26 (18.88-19.64)	68	16.98 (16.81-17.14)
2005-2016	95-100	Organic disorders (inpatient)	157	18.03 (17.91-18.15)	29	16.44 (16.20-16.69)	128	18.44 (18.30-18.57)
1970-2016	35-40	Dementia in alzheimer	219	0.14 (0.14-0.14)	150	0.18 (0.18-0.19)	69	0.09 (0.09-0.09)
1970-1984	35-40	Dementia in alzheimer	131	0.26 (0.26-0.26)	91	0.36 (0.36-0.36)	40	0.16 (0.16-0.16)
1985-1994	35-40	Dementia in alzheimer	66	0.19 (0.19-0.19)	47	0.26 (0.26-0.26)	19	0.11 (0.11-0.11)
1995-2004	35-40	Dementia in alzheimer	6	0.02 (0.02-0.02)	0-4	NA	0-4	NA
2005-2016	35-40	Dementia in alzheimer	16	0.04 (0.04-0.04)	8	0.04 (0.04-0.04)	8	0.04 (0.04-0.04)
1970-2016	40-45	Dementia in alzheimer	419	0.27 (0.27-0.27)	257	0.33 (0.33-0.33)	162	0.21 (0.21-0.21)
1970-1984	40-45	Dementia in alzheimer	243	0.56 (0.56-0.56)	150	0.69 (0.69-0.69)	93	0.43 (0.43-0.43)
1985-1994	40-45	Dementia in alzheimer	126	0.34 (0.34-0.34)	83	0.45 (0.45-0.45)	43	0.23 (0.23-0.24)
1995-2004	40-45	Dementia in alzheimer	19	0.06 (0.06-0.06)	10	0.06 (0.06-0.06)	9	0.05 (0.05-0.05)
2005-2016	40-45	Dementia in alzheimer	31	0.07 (0.07-0.07)	14	0.07 (0.07-0.07)	17	0.08 (0.08-0.08)
1970-2016	45-50	Dementia in alzheimer	855	0.56 (0.56-0.56)	526	0.69 (0.69-0.69)	329	0.43 (0.43-0.43)
1970-1984	45-50	Dementia in alzheimer	545	1.30 (1.29-1.30)	365	1.75 (1.75-1.76)	180	0.85 (0.85-0.85)
1985-1994	45-50	Dementia in alzheimer	208	0.61 (0.61-0.61)	113	0.67 (0.66-0.67)	95	0.56 (0.56-0.56)
1995-2004	45-50	Dementia in alzheimer	35	0.10 (0.10-0.10)	15	0.09 (0.09-0.09)	20	0.12 (0.12-0.12)
2005-2016	45-50	Dementia in alzheimer	67	0.16 (0.16-0.16)	33	0.16 (0.16-0.16)	34	0.16 (0.16-0.16)
1970-2016	50-55	Dementia in alzheimer	1455	1.00 (1.00-1.00)	846	1.17 (1.17-1.17)	609	0.83 (0.83-0.83)
1970-1984	50-55	Dementia in alzheimer	974	2.30 (2.30-2.30)	609	2.93 (2.93-2.93)	365	1.70 (1.69-1.70)
1985-1994	50-55	Dementia in alzheimer	247	0.88 (0.88-0.88)	131	0.94 (0.94-0.95)	116	0.82 (0.82-0.82)
1995-2004	50-55	Dementia in alzheimer	95	0.27 (0.27-0.27)	42	0.24 (0.24-0.24)	53	0.30 (0.30-0.30)
2005-2016	50-55	Dementia in alzheimer	139	0.35 (0.35-0.35)	64	0.32 (0.32-0.32)	75	0.38 (0.38-0.38)
1970-2016	55-60	Dementia in alzheimer	2011	1.46 (1.46-1.46)	1126	1.67 (1.66-1.67)	885	1.27 (1.26-1.27)
1970-1984	55-60	Dementia in alzheimer	1249	2.99 (2.99-2.99)	755	3.71 (3.71-3.72)	494	2.30 (2.30-2.31)
1985-1994	55-60	Dementia in alzheimer	342	1.35 (1.35-1.35)	185	1.50 (1.49-1.50)	157	1.21 (1.21-1.21)
1995-2004	55-60	Dementia in alzheimer	168	0.53 (0.53-0.53)	71	0.45 (0.45-0.45)	97	0.60 (0.60-0.60)
2005-2016	55-60	Dementia in alzheimer	252	0.65 (0.65-0.65)	115	0.60 (0.60-0.60)	137	0.71 (0.71-0.71)
1970-2016	60-65	Dementia in alzheimer	2625	2.04 (2.04-2.04)	1308	2.11 (2.11-2.11)	1317	1.98 (1.98-1.98)
1970-1984	60-65	Dementia in alzheimer	1409	3.54 (3.54-3.55)	743	3.91 (3.90-3.91)	666	3.21 (3.21-3.21)
1985-1994	60-65	Dementia in alzheimer	489	1.99 (1.99-1.99)	230	1.96 (1.96-1.97)	259	2.01 (2.01-2.02)
1995-2004	60-65	Dementia in alzheimer	318	1.25 (1.25-1.25)	139	1.12 (1.12-1.13)	179	1.36 (1.36-1.37)

			Both sexes		Males		Females	
Years	Age	Mental disorder	Cases	IR (95% CI)	Cases	IR (95% CI)	Cases	IR (95% CI)
2005-2016	60-65	Dementia in alzheimer	409	1.06 (1.06-1.06)	196	1.03 (1.03-1.04)	213	1.08 (1.08-1.08)
1970-2016	65-70	Dementia in alzheimer	4691	4.08 (4.08-4.08)	2064	3.82 (3.82-3.82)	2627	4.31 (4.30-4.31)
1970-1984	65-70	Dementia in alzheimer	1971	5.60 (5.59-5.60)	871	5.35 (5.34-5.36)	1100	5.81 (5.80-5.82)
1985-1994	65-70	Dementia in alzheimer	786	3.35 (3.34-3.35)	351	3.24 (3.23-3.25)	435	3.44 (3.43-3.44)
1995-2004	65-70	Dementia in alzheimer	758	3.48 (3.47-3.48)	309	3.01 (3.01-3.02)	449	3.89 (3.88-3.90)
2005-2016	65-70	Dementia in alzheimer	1176	3.41 (3.41-3.41)	533	3.20 (3.20-3.21)	643	3.60 (3.60-3.61)
1970-2016	70-75	Dementia in alzheimer	9405	9.91 (9.90-9.91)	3772	8.86 (8.85-8.87)	5633	10.76 (10.75-10.76)
1970-1984	70-75	Dementia in alzheimer	3317	11.56 (11.55-11.58)	1335	10.65 (10.63-10.67)	1982	12.27 (12.25-12.29)
1985-1994	70-75	Dementia in alzheimer	1441	7.05 (7.04-7.06)	584	6.51 (6.49-6.52)	857	7.48 (7.46-7.49)
1995-2004	70-75	Dementia in alzheimer	1884	9.77 (9.76-9.79)	723	8.37 (8.35-8.39)	1161	10.91 (10.89-10.93)
2005-2016	70-75	Dementia in alzheimer	2763	10.41 (10.40-10.43)	1130	9.10 (9.09-9.12)	1633	11.57 (11.55-11.59)
1970-2016	75-80	Dementia in alzheimer	15354	21.29 (21.27-21.30)	5781	19.18 (19.16-19.20)	9573	22.80 (22.78-22.82)
1970-1984	75-80	Dementia in alzheimer	4560	22.45 (22.42-22.48)	1671	20.22 (20.18-20.27)	2889	23.98 (23.94-24.02)
1985-1994	75-80	Dementia in alzheimer	2134	13.16 (13.14-13.18)	870	13.26 (13.23-13.29)	1264	13.09 (13.06-13.11)
1995-2004	75-80	Dementia in alzheimer	3527	21.94 (21.90-21.97)	1255	18.87 (18.82-18.91)	2272	24.10 (24.05-24.15)
2005-2016	75-80	Dementia in alzheimer	5133	26.30 (26.27-26.34)	1985	22.91 (22.87-22.96)	3148	29.01 (28.96-29.07)
1970-2016	80-85	Dementia in alzheimer	17997	37.54 (37.50-37.57)	6372	35.11 (35.06-35.16)	11625	39.02 (38.97-39.06)
1970-1984	80-85	Dementia in alzheimer	4377	36.32 (36.26-36.39)	1529	33.25 (33.15-33.34)	2848	38.22 (38.13-38.31)
1985-1994	80-85	Dementia in alzheimer	2050	18.98 (18.94-19.02)	773	20.16 (20.10-20.22)	1277	18.33 (18.29-18.37)
1995-2004	80-85	Dementia in alzheimer	4121	36.38 (36.31-36.45)	1394	33.49 (33.39-33.59)	2727	38.06 (37.97-38.15)
2005-2016	80-85	Dementia in alzheimer	7449	54.11 (54.02-54.20)	2676	48.18 (48.06-48.31)	4773	58.12 (58.00-58.25)
1970-2016	85-90	Dementia in alzheimer	12696	49.79 (49.73-49.85)	4091	48.33 (48.23-48.43)	8605	50.51 (50.44-50.59)
1970-1984	85-90	Dementia in alzheimer	2553	46.97 (46.85-47.10)	943	47.86 (47.65-48.08)	1610	46.47 (46.31-46.62)
1985-1994	85-90	Dementia in alzheimer	1204	22.29 (22.23-22.35)	384	23.07 (22.96-23.18)	820	21.94 (21.87-22.01)
1995-2004	85-90	Dementia in alzheimer	2946	46.13 (46.01-46.24)	844	42.47 (42.28-42.65)	2102	47.78 (47.64-47.92)
2005-2016	85-90	Dementia in alzheimer	5993	72.41 (72.26-72.57)	1920	67.54 (67.30-67.79)	4073	74.96 (74.76-75.16)
1970-2016	90-95	Dementia in alzheimer	4079	43.81 (43.72-43.90)	1105	42.53 (42.37-42.70)	2974	44.30 (44.20-44.41)
1970-1984	90-95	Dementia in alzheimer	672	42.85 (42.64-43.06)	235	43.16 (42.80-43.52)	437	42.68 (42.42-42.94)
1985-1994	90-95	Dementia in alzheimer	324	17.66 (17.58-17.74)	115	22.85 (22.65-23.05)	209	15.70 (15.62-15.78)
1995-2004	90-95	Dementia in alzheimer	879	36.19 (36.05-36.33)	208	34.40 (34.13-34.68)	671	36.78 (36.61-36.95)
2005-2016	90-95	Dementia in alzheimer	2204	63.35 (63.14-63.56)	547	57.85 (57.48-58.22)	1657	65.41 (65.15-65.66)
1970-2016	95-100	Dementia in alzheimer	577	29.61 (29.48-29.74)	155	35.34 (35.01-35.67)	422	27.95 (27.81-28.09)
1970-1984	95-100	Dementia in alzheimer	68	27.37 (27.03-27.71)	34	42.54 (41.62-43.49)	34	20.18 (19.87-20.48)
1985-1994	95-100	Dementia in alzheimer	39	11.24 (11.12-11.36)	17	19.69 (19.28-20.11)	22	8.44 (8.34-8.54)
1995-2004	95-100	Dementia in alzheimer	133	26.69 (26.46-26.93)	23	23.33 (22.88-23.80)	110	27.52 (27.25-27.79)
2005-2016	95-100	Dementia in alzheimer	337	39.42 (39.16-39.68)	81	46.60 (45.91-47.30)	256	37.59 (37.31-37.87)
1970-2016	35-40	Dementia in alzheimer (inpatient)	197	0.12 (0.12-0.12)	137	0.17 (0.17-0.17)	60	0.08 (0.08-0.08)
1970-1984	35-40	Dementia in alzheimer (inpatient)	131	0.26 (0.26-0.26)	91	0.36 (0.36-0.36)	40	0.16 (0.16-0.16)
1985-1994	35-40	Dementia in alzheimer (inpatient)	65	0.19 (0.19-0.19)	46	0.26 (0.26-0.26)	19	0.11 (0.11-0.11)
1995-2004	35-40	Dementia in alzheimer (inpatient)	0-4	NA	0-4	NA	0-4	NA

			Both sexes		Males		Females	
**Years**	**Age**	**Mental disorder**	**Cases**	**IR (95% CI)**	**Cases**	**IR (95% CI)**	**Cases**	**IR (95% CI)**
2005-2016	35-40	Dementia in alzheimer (inpatient)	0-4	NA	0-4	NA	0-4	NA
1970-2016	40-45	Dementia in alzheimer (inpatient)	372	0.24 (0.24-0.24)	235	0.30 (0.30-0.30)	137	0.18 (0.18-0.18)
1970-1984	40-45	Dementia in alzheimer (inpatient)	243	0.56 (0.56-0.56)	150	0.69 (0.69-0.69)	93	0.43 (0.43-0.43)
1985-1994	40-45	Dementia in alzheimer (inpatient)	124	0.34 (0.34-0.34)	83	0.45 (0.45-0.45)	41	0.22 (0.22-0.22)
1995-2004	40-45	Dementia in alzheimer (inpatient)	5	0.01 (0.01-0.01)	0-4	NA	0-4	NA
2005-2016	40-45	Dementia in alzheimer (inpatient)	0-4	NA	0-4	NA	0-4	NA
1970-2016	45-50	Dementia in alzheimer (inpatient)	756	0.50 (0.50-0.50)	481	0.63 (0.63-0.63)	275	0.36 (0.36-0.36)
1970-1984	45-50	Dementia in alzheimer (inpatient)	545	1.30 (1.29-1.30)	365	1.75 (1.75-1.76)	180	0.85 (0.85-0.85)
1985-1994	45-50	Dementia in alzheimer (inpatient)	207	0.61 (0.61-0.61)	113	0.67 (0.66-0.67)	94	0.56 (0.56-0.56)
1995-2004	45-50	Dementia in alzheimer (inpatient)	0-4	NA	0-4	NA	0-4	NA
2005-2016	45-50	Dementia in alzheimer (inpatient)	0-4	NA	0-4	NA	0-4	NA
1970-2016	50-55	Dementia in alzheimer (inpatient)	1259	0.87 (0.87-0.87)	758	1.05 (1.05-1.05)	501	0.68 (0.68-0.69)
1970-1984	50-55	Dementia in alzheimer (inpatient)	974	2.30 (2.30-2.30)	609	2.93 (2.93-2.93)	365	1.70 (1.69-1.70)
1985-1994	50-55	Dementia in alzheimer (inpatient)	245	0.87 (0.87-0.88)	129	0.93 (0.93-0.93)	116	0.82 (0.82-0.82)
1995-2004	50-55	Dementia in alzheimer (inpatient)	19	0.05 (0.05-0.05)	9	0.05 (0.05-0.05)	10	0.06 (0.06-0.06)
2005-2016	50-55	Dementia in alzheimer (inpatient)	21	0.05 (0.05-0.05)	11	0.06 (0.06-0.06)	10	0.05 (0.05-0.05)
1970-2016	55-60	Dementia in alzheimer (inpatient)	1678	1.22 (1.22-1.22)	985	1.46 (1.46-1.46)	693	0.99 (0.99-0.99)
1970-1984	55-60	Dementia in alzheimer (inpatient)	1249	2.99 (2.99-2.99)	755	3.71 (3.71-3.72)	494	2.30 (2.30-2.31)
1985-1994	55-60	Dementia in alzheimer (inpatient)	335	1.32 (1.32-1.32)	185	1.50 (1.49-1.50)	150	1.15 (1.15-1.16)
1995-2004	55-60	Dementia in alzheimer (inpatient)	45	0.14 (0.14-0.14)	22	0.14 (0.14-0.14)	23	0.14 (0.14-0.14)
2005-2016	55-60	Dementia in alzheimer (inpatient)	49	0.13 (0.13-0.13)	23	0.12 (0.12-0.12)	26	0.13 (0.13-0.13)
1970-2016	60-65	Dementia in alzheimer (inpatient)	2079	1.62 (1.62-1.62)	1071	1.73 (1.72-1.73)	1008	1.52 (1.52-1.52)
1970-1984	60-65	Dementia in alzheimer (inpatient)	1409	3.54 (3.54-3.55)	743	3.91 (3.90-3.91)	666	3.21 (3.21-3.21)
1985-1994	60-65	Dementia in alzheimer (inpatient)	480	1.95 (1.95-1.96)	230	1.96 (1.96-1.97)	250	1.94 (1.94-1.95)
1995-2004	60-65	Dementia in alzheimer (inpatient)	92	0.36 (0.36-0.36)	54	0.44 (0.44-0.44)	38	0.29 (0.29-0.29)
2005-2016	60-65	Dementia in alzheimer (inpatient)	98	0.25 (0.25-0.25)	44	0.23 (0.23-0.23)	54	0.27 (0.27-0.27)
1970-2016	65-70	Dementia in alzheimer (inpatient)	3163	2.75 (2.75-2.75)	1434	2.65 (2.65-2.66)	1729	2.83 (2.83-2.84)
1970-1984	65-70	Dementia in alzheimer (inpatient)	1971	5.60 (5.59-5.60)	871	5.35 (5.34-5.36)	1100	5.81 (5.80-5.82)
1985-1994	65-70	Dementia in alzheimer (inpatient)	763	3.25 (3.24-3.25)	340	3.14 (3.13-3.14)	423	3.34 (3.34-3.35)
1995-2004	65-70	Dementia in alzheimer (inpatient)	209	0.96 (0.96-0.96)	104	1.01 (1.01-1.02)	105	0.91 (0.91-0.91)
2005-2016	65-70	Dementia in alzheimer (inpatient)	220	0.64 (0.64-0.64)	119	0.71 (0.71-0.72)	101	0.56 (0.56-0.57)
1970-2016	70-75	Dementia in alzheimer (inpatient)	5522	5.81 (5.81-5.81)	2286	5.37 (5.36-5.37)	3236	6.17 (6.16-6.18)
1970-1984	70-75	Dementia in alzheimer (inpatient)	3317	11.56 (11.55-11.58)	1335	10.65 (10.63-10.67)	1982	12.27 (12.25-12.29)
1985-1994	70-75	Dementia in alzheimer (inpatient)	1376	6.73 (6.72-6.74)	565	6.30 (6.28-6.31)	811	7.08 (7.06-7.09)
1995-2004	70-75	Dementia in alzheimer (inpatient)	392	2.03 (2.03-2.03)	176	2.03 (2.03-2.04)	216	2.03 (2.02-2.03)
2005-2016	70-75	Dementia in alzheimer (inpatient)	437	1.64 (1.64-1.64)	210	1.69 (1.68-1.69)	227	1.60 (1.60-1.60)
1970-2016	75-80	Dementia in alzheimer (inpatient)	7917	10.94 (10.93-10.95)	3099	10.25 (10.24-10.27)	4818	11.43 (11.42-11.44)
1970-1984	75-80	Dementia in alzheimer (inpatient)	4560	22.45 (22.42-22.48)	1671	20.22 (20.18-20.27)	2889	23.98 (23.94-24.02)
1985-1994	75-80	Dementia in alzheimer (inpatient)	2050	12.64 (12.62-12.66)	849	12.94 (12.91-12.97)	1201	12.43 (12.41-12.46)
1995-2004	75-80	Dementia in alzheimer (inpatient)	708	4.38 (4.38-4.39)	302	4.53 (4.51-4.54)	406	4.29 (4.28-4.29)

			Both sexes		Males		Females	
Years	Age	Mental disorder	Cases	IR (95% CI)	Cases	IR (95% CI)	Cases	IR (95% CI)
2005-2016	75-80	Dementia in alzheimer (inpatient)	599	3.04 (3.04-3.05)	277	3.18 (3.17-3.18)	322	2.94 (2.93-2.94)
1970-2016	80-85	Dementia in alzheimer (inpatient)	7736	16.01 (16.00-16.03)	2885	15.80 (15.77-15.82)	4851	16.14 (16.13-16.16)
1970-1984	80-85	Dementia in alzheimer (inpatient)	4377	36.32 (36.26-36.39)	1529	33.25 (33.15-33.34)	2848	38.22 (38.13-38.31)
1985-1994	80-85	Dementia in alzheimer (inpatient)	1916	17.74 (17.70-17.77)	736	19.19 (19.13-19.25)	1180	16.94 (16.90-16.98)
1995-2004	80-85	Dementia in alzheimer (inpatient)	722	6.33 (6.31-6.34)	292	6.97 (6.95-7.00)	430	5.95 (5.94-5.96)
2005-2016	80-85	Dementia in alzheimer (inpatient)	721	5.13 (5.12-5.14)	328	5.81 (5.80-5.83)	393	4.68 (4.67-4.69)
1970-2016	85-90	Dementia in alzheimer (inpatient)	4640	17.95 (17.93-17.97)	1684	19.69 (19.65-19.73)	2956	17.10 (17.07-17.12)
1970-1984	85-90	Dementia in alzheimer (inpatient)	2553	46.97 (46.85-47.10)	943	47.86 (47.65-48.08)	1610	46.47 (46.31-46.62)
1985-1994	85-90	Dementia in alzheimer (inpatient)	1129	20.90 (20.84-20.95)	375	22.53 (22.42-22.64)	754	20.17 (20.10-20.23)
1995-2004	85-90	Dementia in alzheimer (inpatient)	449	6.95 (6.94-6.97)	166	8.28 (8.25-8.32)	283	6.35 (6.34-6.37)
2005-2016	85-90	Dementia in alzheimer (inpatient)	509	5.95 (5.94-5.97)	200	6.86 (6.84-6.89)	309	5.48 (5.47-5.50)
1970-2016	90-95	Dementia in alzheimer (inpatient)	1306	13.80 (13.77-13.83)	439	16.70 (16.63-16.76)	867	12.68 (12.65-12.71)
1970-1984	90-95	Dementia in alzheimer (inpatient)	672	42.85 (42.64-43.06)	235	43.16 (42.80-43.52)	437	42.68 (42.42-42.94)
1985-1994	90-95	Dementia in alzheimer (inpatient)	303	16.51 (16.44-16.59)	111	22.05 (21.86-22.24)	192	14.42 (14.34-14.50)
1995-2004	90-95	Dementia in alzheimer (inpatient)	136	5.54 (5.51-5.56)	41	6.72 (6.67-6.78)	95	5.14 (5.12-5.17)
2005-2016	90-95	Dementia in alzheimer (inpatient)	195	5.41 (5.39-5.43)	52	5.35 (5.32-5.38)	143	5.43 (5.41-5.45)
1970-2016	95-100	Dementia in alzheimer (inpatient)	139	7.03 (7.00-7.06)	55	12.41 (12.29-12.52)	84	5.48 (5.45-5.50)
1970-1984	95-100	Dementia in alzheimer (inpatient)	68	27.37 (27.03-27.71)	34	42.54 (41.62-43.49)	34	20.18 (19.87-20.48)
1985-1994	95-100	Dementia in alzheimer (inpatient)	35	10.09 (9.98-10.19)	15	17.38 (17.01-17.75)	20	7.67 (7.58-7.77)
1995-2004	95-100	Dementia in alzheimer (inpatient)	13	2.58 (2.56-2.61)	0-4	NA	9-13	2.48 (2.45-2.50)
2005-2016	95-100	Dementia in alzheimer (inpatient)	23	2.62 (2.60-2.63)	0-4	NA	19-23	2.85 (2.83-2.87)
1970-2016	35-40	Vascular dementia	89	0.06 (0.06-0.06)	53	0.07 (0.07-0.07)	36	0.05 (0.05-0.05)
1970-1984	35-40	Vascular dementia	57	0.12 (0.11-0.12)	34	0.14 (0.14-0.14)	23	0.09 (0.09-0.09)
1985-1994	35-40	Vascular dementia	15	0.04 (0.04-0.04)	9	0.05 (0.05-0.05)	6	0.03 (0.03-0.03)
1995-2004	35-40	Vascular dementia	12	0.03 (0.03-0.03)	8-12	0.04 (0.04-0.04)	0-4	NA
2005-2016	35-40	Vascular dementia	5	0.01 (0.01-0.01)	0-4	NA	0-4	NA
1970-2016	40-45	Vascular dementia	136	0.09 (0.09-0.09)	81	0.10 (0.10-0.10)	55	0.07 (0.07-0.07)
1970-1984	40-45	Vascular dementia	79	0.18 (0.18-0.18)	52	0.24 (0.24-0.24)	27	0.12 (0.12-0.12)
1985-1994	40-45	Vascular dementia	17	0.05 (0.05-0.05)	10	0.05 (0.05-0.05)	7	0.04 (0.04-0.04)
1995-2004	40-45	Vascular dementia	29	0.09 (0.09-0.09)	14	0.08 (0.08-0.08)	15	0.09 (0.09-0.09)
2005-2016	40-45	Vascular dementia	11	0.03 (0.03-0.03)	5	0.02 (0.02-0.02)	6	0.03 (0.03-0.03)
1970-2016	45-50	Vascular dementia	235	0.15 (0.15-0.15)	152	0.20 (0.20-0.20)	83	0.11 (0.11-0.11)
1970-1984	45-50	Vascular dementia	126	0.30 (0.30-0.30)	79	0.38 (0.38-0.38)	47	0.22 (0.22-0.22)
1985-1994	45-50	Vascular dementia	34	0.10 (0.10-0.10)	22	0.13 (0.13-0.13)	12	0.07 (0.07-0.07)
1995-2004	45-50	Vascular dementia	51	0.15 (0.15-0.15)	35	0.20 (0.20-0.20)	16	0.09 (0.09-0.09)
2005-2016	45-50	Vascular dementia	24	0.06 (0.06-0.06)	16	0.08 (0.08-0.08)	8	0.04 (0.04-0.04)
1970-2016	50-55	Vascular dementia	464	0.32 (0.32-0.32)	302	0.42 (0.42-0.42)	162	0.22 (0.22-0.22)
1970-1984	50-55	Vascular dementia	241	0.57 (0.57-0.57)	152	0.73 (0.73-0.73)	89	0.41 (0.41-0.41)
1985-1994	50-55	Vascular dementia	59	0.21 (0.21-0.21)	40	0.29 (0.29-0.29)	19	0.13 (0.13-0.13)
1995-2004	50-55	Vascular dementia	104	0.29 (0.29-0.29)	75	0.42 (0.42-0.43)	29	0.16 (0.16-0.16)

			Both sexes		Males		Females	
**Years**	**Age**	**Mental disorder**	**Cases**	**IR (95% CI)**	**Cases**	**IR (95% CI)**	**Cases**	**IR (95% CI)**
2005-2016	50-55	Vascular dementia	60	0.15 (0.15-0.15)	35	0.18 (0.18-0.18)	25	0.13 (0.13-0.13)
1970-2016	55-60	Vascular dementia	763	0.55 (0.55-0.55)	500	0.74 (0.74-0.74)	263	0.38 (0.38-0.38)
1970-1984	55-60	Vascular dementia	411	0.98 (0.98-0.98)	277	1.36 (1.36-1.36)	134	0.62 (0.62-0.62)
1985-1994	55-60	Vascular dementia	83	0.33 (0.33-0.33)	49	0.40 (0.39-0.40)	34	0.26 (0.26-0.26)
1995-2004	55-60	Vascular dementia	164	0.51 (0.51-0.52)	106	0.67 (0.67-0.67)	58	0.36 (0.36-0.36)
2005-2016	55-60	Vascular dementia	105	0.27 (0.27-0.27)	68	0.36 (0.35-0.36)	37	0.19 (0.19-0.19)
1970-2016	60-65	Vascular dementia	1319	1.03 (1.03-1.03)	810	1.30 (1.30-1.31)	509	0.77 (0.77-0.77)
1970-1984	60-65	Vascular dementia	692	1.74 (1.74-1.74)	424	2.23 (2.22-2.23)	268	1.29 (1.29-1.29)
1985-1994	60-65	Vascular dementia	147	0.60 (0.60-0.60)	81	0.69 (0.69-0.69)	66	0.51 (0.51-0.51)
1995-2004	60-65	Vascular dementia	256	1.00 (1.00-1.00)	158	1.28 (1.28-1.28)	98	0.75 (0.74-0.75)
2005-2016	60-65	Vascular dementia	224	0.58 (0.58-0.58)	147	0.78 (0.77-0.78)	77	0.39 (0.39-0.39)
1970-2016	65-70	Vascular dementia	2715	2.36 (2.36-2.36)	1504	2.78 (2.78-2.78)	1211	1.98 (1.98-1.98)
1970-1984	65-70	Vascular dementia	1318	3.74 (3.73-3.74)	721	4.42 (4.42-4.43)	597	3.15 (3.14-3.15)
1985-1994	65-70	Vascular dementia	278	1.18 (1.18-1.18)	151	1.39 (1.39-1.39)	127	1.00 (1.00-1.00)
1995-2004	65-70	Vascular dementia	591	2.71 (2.70-2.71)	331	3.22 (3.22-3.23)	260	2.25 (2.25-2.25)
2005-2016	65-70	Vascular dementia	528	1.53 (1.53-1.53)	301	1.81 (1.80-1.81)	227	1.27 (1.27-1.27)
1970-2016	70-75	Vascular dementia	4855	5.10 (5.10-5.10)	2493	5.85 (5.84-5.85)	2362	4.50 (4.49-4.50)
1970-1984	70-75	Vascular dementia	1891	6.58 (6.57-6.59)	904	7.20 (7.19-7.22)	987	6.10 (6.09-6.10)
1985-1994	70-75	Vascular dementia	545	2.66 (2.65-2.66)	278	3.09 (3.08-3.09)	267	2.32 (2.32-2.33)
1995-2004	70-75	Vascular dementia	1295	6.70 (6.69-6.71)	684	7.90 (7.89-7.92)	611	5.72 (5.71-5.73)
2005-2016	70-75	Vascular dementia	1124	4.23 (4.22-4.23)	627	5.04 (5.03-5.05)	497	3.51 (3.50-3.52)
1970-2016	75-80	Vascular dementia	6947	9.58 (9.58-9.59)	3326	11.00 (10.99-11.01)	3621	8.57 (8.56-8.58)
1970-1984	75-80	Vascular dementia	2104	10.32 (10.31-10.34)	964	11.64 (11.62-11.67)	1140	9.42 (9.40-9.44)
1985-1994	75-80	Vascular dementia	708	4.34 (4.34-4.35)	361	5.48 (5.47-5.49)	347	3.57 (3.56-3.58)
1995-2004	75-80	Vascular dementia	2234	13.84 (13.82-13.86)	1061	15.91 (15.87-15.95)	1173	12.38 (12.35-12.40)
2005-2016	75-80	Vascular dementia	1901	9.68 (9.66-9.69)	940	10.80 (10.78-10.82)	961	8.78 (8.76-8.80)
1970-2016	80-85	Vascular dementia	7162	14.80 (14.78-14.81)	3189	17.46 (17.43-17.48)	3973	13.18 (13.17-13.20)
1970-1984	80-85	Vascular dementia	1595	13.14 (13.12-13.16)	730	15.81 (15.77-15.86)	865	11.50 (11.47-11.53)
1985-1994	80-85	Vascular dementia	590	5.42 (5.41-5.43)	278	7.21 (7.19-7.24)	312	4.44 (4.43-4.45)
1995-2004	80-85	Vascular dementia	2452	21.50 (21.47-21.54)	1091	26.11 (26.03-26.18)	1361	18.84 (18.80-18.89)
2005-2016	80-85	Vascular dementia	2525	18.06 (18.03-18.09)	1090	19.41 (19.35-19.46)	1435	17.15 (17.12-17.19)
1970-2016	85-90	Vascular dementia	4944	19.10 (19.07-19.12)	1975	23.10 (23.05-23.15)	2969	17.12 (17.10-17.15)
1970-1984	85-90	Vascular dementia	772	14.04 (14.00-14.08)	387	19.53 (19.44-19.61)	385	10.95 (10.91-10.98)
1985-1994	85-90	Vascular dementia	370	6.78 (6.76-6.80)	161	9.61 (9.57-9.66)	209	5.53 (5.51-5.54)
1995-2004	85-90	Vascular dementia	1792	27.80 (27.74-27.87)	698	34.95 (34.80-35.11)	1094	24.59 (24.52-24.67)
2005-2016	85-90	Vascular dementia	2010	23.68 (23.63-23.73)	729	25.17 (25.08-25.26)	1281	22.90 (22.84-22.96)
1970-2016	90-95	Vascular dementia	1467	15.48 (15.45-15.51)	473	18.00 (17.93-18.07)	994	14.51 (14.47-14.54)
1970-1984	90-95	Vascular dementia	179	11.25 (11.20-11.31)	86	15.69 (15.56-15.82)	93	8.92 (8.87-8.98)
1985-1994	90-95	Vascular dementia	69	3.72 (3.70-3.74)	34	6.72 (6.66-6.77)	35	2.60 (2.58-2.61)
1995-2004	90-95	Vascular dementia	496	20.21 (20.13-20.29)	151	24.84 (24.65-25.04)	345	18.69 (18.60-18.77)

			Both sexes		Males		Females	
**Years**	**Age**	**Mental disorder**	**Cases**	**IR (95% CI)**	**Cases**	**IR (95% CI)**	**Cases**	**IR (95% CI)**
2005-2016	90-95	Vascular dementia	723	20.20 (20.13-20.27)	202	20.92 (20.79-21.06)	521	19.93 (19.86-20.01)
1970-2016	95-100	Vascular dementia	252	12.73 (12.68-12.79)	66	14.90 (14.76-15.04)	186	12.11 (12.05-12.17)
1970-1984	95-100	Vascular dementia	27	10.70 (10.57-10.83)	15	18.65 (18.25-19.07)	12	6.98 (6.87-7.08)
1985-1994	95-100	Vascular dementia	7	2.00 (1.98-2.02)	0-4	NA	0-4	NA
1995-2004	95-100	Vascular dementia	71	14.12 (13.99-14.24)	17	17.16 (16.83-17.50)	54	13.37 (13.24-13.50)
2005-2016	95-100	Vascular dementia	147	16.83 (16.72-16.94)	31	17.54 (17.29-17.80)	116	16.65 (16.52-16.77)
1970-2016	35-40	Vascular dementia (inpatient)	80	0.05 (0.05-0.05)	47	0.06 (0.06-0.06)	33	0.04 (0.04-0.04)
1970-1984	35-40	Vascular dementia (inpatient)	57	0.12 (0.11-0.12)	34	0.14 (0.14-0.14)	23	0.09 (0.09-0.09)
1985-1994	35-40	Vascular dementia (inpatient)	15	0.04 (0.04-0.04)	9	0.05 (0.05-0.05)	6	0.03 (0.03-0.03)
1995-2004	35-40	Vascular dementia (inpatient)	6	0.02 (0.02-0.02)	0-4	NA	0-4	NA
2005-2016	35-40	Vascular dementia (inpatient)	0-4	NA	0-4	NA	0-4	NA
1970-2016	40-45	Vascular dementia (inpatient)	115	0.07 (0.07-0.07)	70	0.09 (0.09-0.09)	45	0.06 (0.06-0.06)
1970-1984	40-45	Vascular dementia (inpatient)	79	0.18 (0.18-0.18)	52	0.24 (0.24-0.24)	27	0.12 (0.12-0.12)
1985-1994	40-45	Vascular dementia (inpatient)	15	0.04 (0.04-0.04)	10	0.05 (0.05-0.05)	5	0.03 (0.03-0.03)
1995-2004	40-45	Vascular dementia (inpatient)	15	0.04 (0.04-0.04)	0-4	NA	11-15	0.07 (0.07-0.07)
2005-2016	40-45	Vascular dementia (inpatient)	6	0.01 (0.01-0.01)	0-4	NA	0-4	NA
1970-2016	45-50	Vascular dementia (inpatient)	198	0.13 (0.13-0.13)	125	0.16 (0.16-0.16)	73	0.10 (0.10-0.10)
1970-1984	45-50	Vascular dementia (inpatient)	126	0.30 (0.30-0.30)	79	0.38 (0.38-0.38)	47	0.22 (0.22-0.22)
1985-1994	45-50	Vascular dementia (inpatient)	33	0.10 (0.10-0.10)	22	0.13 (0.13-0.13)	11	0.07 (0.07-0.07)
1995-2004	45-50	Vascular dementia (inpatient)	25	0.07 (0.07-0.07)	15	0.09 (0.09-0.09)	10	0.06 (0.06-0.06)
2005-2016	45-50	Vascular dementia (inpatient)	14	0.03 (0.03-0.03)	9	0.04 (0.04-0.04)	5	0.02 (0.02-0.02)
1970-2016	50-55	Vascular dementia (inpatient)	367	0.25 (0.25-0.25)	239	0.33 (0.33-0.33)	128	0.17 (0.17-0.17)
1970-1984	50-55	Vascular dementia (inpatient)	241	0.57 (0.57-0.57)	152	0.73 (0.73-0.73)	89	0.41 (0.41-0.41)
1985-1994	50-55	Vascular dementia (inpatient)	57	0.20 (0.20-0.20)	39	0.28 (0.28-0.28)	18	0.13 (0.13-0.13)
1995-2004	50-55	Vascular dementia (inpatient)	48	0.14 (0.14-0.14)	34	0.19 (0.19-0.19)	14	0.08 (0.08-0.08)
2005-2016	50-55	Vascular dementia (inpatient)	21	0.05 (0.05-0.05)	14	0.07 (0.07-0.07)	7	0.04 (0.04-0.04)
1970-2016	55-60	Vascular dementia (inpatient)	605	0.44 (0.44-0.44)	393	0.58 (0.58-0.58)	212	0.30 (0.30-0.30)
1970-1984	55-60	Vascular dementia (inpatient)	411	0.98 (0.98-0.98)	277	1.36 (1.36-1.36)	134	0.62 (0.62-0.62)
1985-1994	55-60	Vascular dementia (inpatient)	82	0.32 (0.32-0.32)	48	0.39 (0.39-0.39)	34	0.26 (0.26-0.26)
1995-2004	55-60	Vascular dementia (inpatient)	78	0.24 (0.24-0.25)	45	0.29 (0.28-0.29)	33	0.21 (0.20-0.21)
2005-2016	55-60	Vascular dementia (inpatient)	34	0.09 (0.09-0.09)	23	0.12 (0.12-0.12)	11	0.06 (0.06-0.06)
1970-2016	60-65	Vascular dementia (inpatient)	1000	0.78 (0.78-0.78)	608	0.98 (0.98-0.98)	392	0.59 (0.59-0.59)
1970-1984	60-65	Vascular dementia (inpatient)	692	1.74 (1.74-1.74)	424	2.23 (2.22-2.23)	268	1.29 (1.29-1.29)
1985-1994	60-65	Vascular dementia (inpatient)	141	0.57 (0.57-0.57)	78	0.66 (0.66-0.67)	63	0.49 (0.49-0.49)
1995-2004	60-65	Vascular dementia (inpatient)	101	0.40 (0.40-0.40)	60	0.48 (0.48-0.49)	41	0.31 (0.31-0.31)
2005-2016	60-65	Vascular dementia (inpatient)	66	0.17 (0.17-0.17)	46	0.24 (0.24-0.24)	20	0.10 (0.10-0.10)
1970-2016	65-70	Vascular dementia (inpatient)	1893	1.64 (1.64-1.64)	1026	1.90 (1.90-1.90)	867	1.42 (1.42-1.42)
1970-1984	65-70	Vascular dementia (inpatient)	1318	3.74 (3.73-3.74)	721	4.42 (4.42-4.43)	597	3.15 (3.14-3.15)
1985-1994	65-70	Vascular dementia (inpatient)	256	1.09 (1.09-1.09)	142	1.31 (1.30-1.31)	114	0.90 (0.90-0.90)
1995-2004	65-70	Vascular dementia (inpatient)	192	0.88 (0.88-0.88)	94	0.91 (0.91-0.92)	98	0.85 (0.85-0.85)

			Both sexes		Males		Females	
Years	Age	Mental disorder	Cases	IR (95% CI)	Cases	IR (95% CI)	Cases	IR (95% CI)
2005-2016	65-70	Vascular dementia (inpatient)	127	0.37 (0.37-0.37)	69	0.41 (0.41-0.41)	58	0.32 (0.32-0.32)
1970-2016	70-75	Vascular dementia (inpatient)	2968	3.12 (3.12-3.12)	1454	3.41 (3.40-3.41)	1514	2.88 (2.88-2.88)
1970-1984	70-75	Vascular dementia (inpatient)	1891	6.58 (6.57-6.59)	904	7.20 (7.19-7.22)	987	6.10 (6.09-6.10)
1985-1994	70-75	Vascular dementia (inpatient)	505	2.46 (2.46-2.47)	254	2.82 (2.82-2.83)	251	2.18 (2.18-2.19)
1995-2004	70-75	Vascular dementia (inpatient)	379	1.96 (1.96-1.96)	189	2.18 (2.18-2.19)	190	1.78 (1.78-1.78)
2005-2016	70-75	Vascular dementia (inpatient)	193	0.72 (0.72-0.73)	107	0.86 (0.86-0.86)	86	0.61 (0.61-0.61)
1970-2016	75-80	Vascular dementia (inpatient)	3543	4.88 (4.88-4.89)	1701	5.62 (5.61-5.62)	1842	4.36 (4.35-4.36)
1970-1984	75-80	Vascular dementia (inpatient)	2104	10.32 (10.31-10.34)	964	11.64 (11.62-11.67)	1140	9.42 (9.40-9.44)
1985-1994	75-80	Vascular dementia (inpatient)	652	4.00 (3.99-4.01)	341	5.18 (5.16-5.19)	311	3.20 (3.19-3.21)
1995-2004	75-80	Vascular dementia (inpatient)	525	3.24 (3.24-3.25)	265	3.96 (3.95-3.97)	260	2.74 (2.73-2.74)
2005-2016	75-80	Vascular dementia (inpatient)	262	1.33 (1.33-1.33)	131	1.50 (1.50-1.50)	131	1.19 (1.19-1.20)
1970-2016	80-85	Vascular dementia (inpatient)	2940	6.06 (6.05-6.06)	1416	7.73 (7.72-7.74)	1524	5.04 (5.04-5.05)
1970-1984	80-85	Vascular dementia (inpatient)	1595	13.14 (13.12-13.16)	730	15.81 (15.77-15.86)	865	11.50 (11.47-11.53)
1985-1994	80-85	Vascular dementia (inpatient)	520	4.78 (4.77-4.79)	251	6.51 (6.49-6.53)	269	3.83 (3.82-3.84)
1995-2004	80-85	Vascular dementia (inpatient)	553	4.83 (4.82-4.84)	289	6.89 (6.87-6.91)	264	3.64 (3.63-3.65)
2005-2016	80-85	Vascular dementia (inpatient)	272	1.93 (1.93-1.94)	146	2.58 (2.58-2.59)	126	1.50 (1.49-1.50)
1970-2016	85-90	Vascular dementia (inpatient)	1640	6.31 (6.30-6.31)	764	8.90 (8.88-8.92)	876	5.03 (5.02-5.04)
1970-1984	85-90	Vascular dementia (inpatient)	772	14.04 (14.00-14.08)	387	19.53 (19.44-19.61)	385	10.95 (10.91-10.98)
1985-1994	85-90	Vascular dementia (inpatient)	324	5.94 (5.92-5.95)	141	8.42 (8.38-8.46)	183	4.84 (4.82-4.85)
1995-2004	85-90	Vascular dementia (inpatient)	355	5.48 (5.46-5.49)	177	8.81 (8.77-8.85)	178	3.98 (3.97-3.99)
2005-2016	85-90	Vascular dementia (inpatient)	189	2.21 (2.20-2.21)	59	2.02 (2.01-2.03)	130	2.30 (2.30-2.31)
1970-2016	90-95	Vascular dementia (inpatient)	369	3.87 (3.87-3.88)	162	6.14 (6.11-6.16)	207	3.01 (3.00-3.01)
1970-1984	90-95	Vascular dementia (inpatient)	179	11.25 (11.20-11.31)	86	15.69 (15.56-15.82)	93	8.92 (8.87-8.98)
1985-1994	90-95	Vascular dementia (inpatient)	58	3.13 (3.11-3.14)	32	6.32 (6.27-6.38)	26	1.93 (1.92-1.94)
1995-2004	90-95	Vascular dementia (inpatient)	71	2.88 (2.87-2.89)	20	3.27 (3.25-3.30)	51	2.75 (2.74-2.76)
2005-2016	90-95	Vascular dementia (inpatient)	61	1.69 (1.68-1.69)	24	2.46 (2.45-2.48)	37	1.40 (1.40-1.41)
1970-2016	95-100	Vascular dementia (inpatient)	54	2.72 (2.70-2.73)	22	4.95 (4.90-4.99)	32	2.07 (2.06-2.08)
1970-1984	95-100	Vascular dementia (inpatient)	27	10.70 (10.57-10.83)	15	18.65 (18.25-19.07)	12	6.98 (6.87-7.08)
1985-1994	95-100	Vascular dementia (inpatient)	0-4	NA	0-4	NA	0-4	NA
1995-2004	95-100	Vascular dementia (inpatient)	8	1.58 (1.57-1.60)	0-4	NA	4-8	1.48 (1.47-1.49)
2005-2016	95-100	Vascular dementia (inpatient)	15	1.70 (1.69-1.71)	0-4	NA	11-15	1.85 (1.84-1.86)
1970-2016	10-15	Substance use disorder	578	0.37 (0.37-0.37)	251	0.31 (0.31-0.31)	327	0.42 (0.42-0.42)
1970-1984	10-15	Substance use disorder	98	0.17 (0.17-0.17)	42	0.14 (0.14-0.14)	56	0.20 (0.20-0.20)
1985-1994	10-15	Substance use disorder	17	0.05 (0.05-0.05)	6	0.04 (0.04-0.04)	11	0.07 (0.07-0.07)
1995-2004	10-15	Substance use disorder	102	0.34 (0.34-0.35)	45	0.30 (0.30-0.30)	57	0.40 (0.39-0.40)
2005-2016	10-15	Substance use disorder	361	0.90 (0.90-0.90)	158	0.77 (0.77-0.77)	203	1.04 (1.04-1.04)
1970-2016	15-20	Substance use disorder	13040	8.12 (8.12-8.13)	8309	10.09 (10.08-10.10)	4731	6.05 (6.05-6.06)
1970-1984	15-20	Substance use disorder	3130	5.49 (5.48-5.49)	2019	6.89 (6.89-6.90)	1111	4.00 (4.00-4.01)
1985-1994	15-20	Substance use disorder	849	2.40 (2.40-2.41)	591	3.26 (3.26-3.27)	258	1.50 (1.50-1.50)
1995-2004	15-20	Substance use disorder	2330	8.20 (8.19-8.21)	1517	10.43 (10.41-10.44)	813	5.86 (5.85-5.87)

			Both sexes		Males		Females	
Years	Age	Mental disorder	Cases	IR (95% CI)	Cases	IR (95% CI)	Cases	IR (95% CI)
2005-2016	15-20	Substance use disorder	6731	16.93 (16.92-16.95)	4182	20.50 (20.47-20.53)	2549	13.18 (13.16-13.20)
1970-2016	20-25	Substance use disorder	20123	12.54 (12.53-12.55)	14441	17.44 (17.43-17.46)	5682	7.31 (7.31-7.32)
1970-1984	20-25	Substance use disorder	6050	10.95 (10.94-10.96)	4365	15.32 (15.31-15.34)	1685	6.30 (6.29-6.30)
1985-1994	20-25	Substance use disorder	2204	5.92 (5.91-5.93)	1616	8.35 (8.34-8.36)	588	3.29 (3.29-3.30)
1995-2004	20-25	Substance use disorder	3880	12.57 (12.56-12.58)	2891	18.15 (18.12-18.18)	989	6.62 (6.61-6.63)
2005-2016	20-25	Substance use disorder	7989	21.52 (21.50-21.54)	5569	29.30 (29.26-29.34)	2420	13.36 (13.34-13.38)
1970-2016	25-30	Substance use disorder	18489	11.51 (11.50-11.51)	13368	16.21 (16.20-16.22)	5121	6.55 (6.55-6.55)
1970-1984	25-30	Substance use disorder	6730	11.95 (11.94-11.96)	4656	16.14 (16.12-16.16)	2074	7.56 (7.55-7.57)
1985-1994	25-30	Substance use disorder	2903	7.92 (7.91-7.92)	2063	10.88 (10.86-10.89)	840	4.75 (4.74-4.75)
1995-2004	25-30	Substance use disorder	3778	11.28 (11.27-11.30)	2865	16.60 (16.57-16.62)	913	5.63 (5.62-5.64)
2005-2016	25-30	Substance use disorder	5078	14.84 (14.83-14.86)	3784	21.75 (21.72-21.79)	1294	7.70 (7.68-7.71)
1970-2016	30-35	Substance use disorder	19392	12.20 (12.19-12.21)	13464	16.61 (16.60-16.63)	5928	7.61 (7.60-7.61)
1970-1984	30-35	Substance use disorder	7592	14.09 (14.08-14.11)	4944	18.07 (18.05-18.09)	2648	9.99 (9.98-10.00)
1985-1994	30-35	Substance use disorder	3414	9.98 (9.97-9.99)	2304	13.15 (13.13-13.17)	1110	6.65 (6.64-6.66)
1995-2004	30-35	Substance use disorder	4318	12.08 (12.07-12.10)	3177	17.32 (17.29-17.34)	1141	6.56 (6.55-6.57)
2005-2016	30-35	Substance use disorder	4068	11.58 (11.57-11.59)	3039	17.07 (17.04-17.09)	1029	5.94 (5.93-5.95)
1970-2016	35-40	Substance use disorder	20501	13.06 (13.06-13.07)	13393	16.86 (16.85-16.87)	7108	9.17 (9.16-9.18)
1970-1984	35-40	Substance use disorder	7727	15.77 (15.76-15.78)	4730	19.14 (19.12-19.17)	2997	12.34 (12.32-12.36)
1985-1994	35-40	Substance use disorder	3720	10.82 (10.81-10.83)	2345	13.50 (13.48-13.52)	1375	8.09 (8.07-8.10)
1995-2004	35-40	Substance use disorder	4982	14.04 (14.02-14.05)	3404	18.84 (18.81-18.87)	1578	9.06 (9.04-9.07)
2005-2016	35-40	Substance use disorder	4072	10.69 (10.68-10.70)	2914	15.11 (15.09-15.13)	1158	6.16 (6.15-6.17)
1970-2016	40-45	Substance use disorder	20221	13.20 (13.19-13.20)	12450	16.17 (16.16-16.18)	7771	10.19 (10.18-10.20)
1970-1984	40-45	Substance use disorder	7173	16.71 (16.69-16.72)	4316	20.15 (20.12-20.18)	2857	13.28 (13.26-13.30)
1985-1994	40-45	Substance use disorder	3684	10.18 (10.17-10.19)	2123	11.70 (11.68-11.72)	1561	8.66 (8.65-8.67)
1995-2004	40-45	Substance use disorder	4928	14.85 (14.84-14.87)	3056	18.25 (18.22-18.28)	1872	11.39 (11.38-11.41)
2005-2016	40-45	Substance use disorder	4436	10.83 (10.82-10.84)	2955	14.29 (14.27-14.31)	1481	7.31 (7.30-7.32)
1970-2016	45-50	Substance use disorder	18828	12.65 (12.65-12.66)	11236	15.15 (15.13-15.16)	7592	10.18 (10.17-10.18)
1970-1984	45-50	Substance use disorder	6673	16.06 (16.04-16.08)	4000	19.49 (19.47-19.52)	2673	12.71 (12.69-12.73)
1985-1994	45-50	Substance use disorder	2913	8.78 (8.77-8.79)	1653	9.98 (9.96-9.99)	1260	7.59 (7.58-7.60)
1995-2004	45-50	Substance use disorder	4515	13.63 (13.61-13.64)	2679	16.19 (16.16-16.21)	1836	11.07 (11.06-11.09)
2005-2016	45-50	Substance use disorder	4727	11.55 (11.54-11.56)	2904	14.13 (14.11-14.15)	1823	8.94 (8.93-8.96)
1970-2016	50-55	Substance use disorder	15230	10.70 (10.69-10.70)	8869	12.59 (12.58-12.60)	6361	8.84 (8.83-8.85)
1970-1984	50-55	Substance use disorder	5569	13.31 (13.29-13.32)	3264	15.91 (15.88-15.93)	2305	10.81 (10.79-10.82)
1985-1994	50-55	Substance use disorder	1998	7.28 (7.27-7.29)	1075	7.93 (7.92-7.95)	923	6.65 (6.63-6.66)
1995-2004	50-55	Substance use disorder	3771	10.94 (10.93-10.95)	2194	12.82 (12.80-12.84)	1577	9.08 (9.07-9.09)
2005-2016	50-55	Substance use disorder	3892	10.08 (10.07-10.09)	2336	12.13 (12.11-12.15)	1556	8.04 (8.02-8.05)
1970-2016	55-60	Substance use disorder	11463	8.50 (8.50-8.51)	6566	9.94 (9.94-9.95)	4897	7.12 (7.11-7.12)
1970-1984	55-60	Substance use disorder	4270	10.30 (10.29-10.31)	2390	11.87 (11.85-11.89)	1880	8.82 (8.81-8.84)
1985-1994	55-60	Substance use disorder	1393	5.60 (5.59-5.60)	754	6.23 (6.22-6.24)	639	5.00 (4.99-5.00)
1995-2004	55-60	Substance use disorder	2618	8.42 (8.41-8.43)	1446	9.43 (9.41-9.44)	1172	7.44 (7.42-7.45)

			Both sexes		Males		Females	
**Years**	**Age**	**Mental disorder**	**Cases**	**IR (95% CI)**	**Cases**	**IR (95% CI)**	**Cases**	**IR (95% CI)**
2005-2016	55-60	Substance use disorder	3182	8.51 (8.50-8.52)	1976	10.71 (10.69-10.72)	1206	6.37 (6.36-6.38)
1970-2016	60-65	Substance use disorder	7809	6.18 (6.18-6.18)	4287	7.04 (7.04-7.05)	3522	5.38 (5.38-5.38)
1970-1984	60-65	Substance use disorder	2811	7.11 (7.10-7.12)	1522	8.05 (8.04-8.06)	1289	6.24 (6.24-6.25)
1985-1994	60-65	Substance use disorder	1077	4.45 (4.44-4.45)	548	4.76 (4.75-4.76)	529	4.16 (4.16-4.17)
1995-2004	60-65	Substance use disorder	1618	6.48 (6.47-6.49)	888	7.35 (7.34-7.36)	730	5.67 (5.66-5.68)
2005-2016	60-65	Substance use disorder	2303	6.12 (6.12-6.13)	1329	7.23 (7.22-7.24)	974	5.06 (5.06-5.07)
1970-2016	65-70	Substance use disorder	5144	4.53 (4.52-4.53)	2673	5.02 (5.01-5.02)	2471	4.10 (4.09-4.10)
1970-1984	65-70	Substance use disorder	1735	4.94 (4.93-4.94)	868	5.35 (5.34-5.36)	867	4.59 (4.58-4.59)
1985-1994	65-70	Substance use disorder	704	3.03 (3.02-3.03)	347	3.24 (3.23-3.24)	357	2.85 (2.84-2.85)
1995-2004	65-70	Substance use disorder	1071	4.99 (4.99-5.00)	562	5.58 (5.57-5.59)	509	4.48 (4.47-4.48)
2005-2016	65-70	Substance use disorder	1634	4.84 (4.83-4.84)	896	5.51 (5.50-5.52)	738	4.21 (4.20-4.22)
1970-2016	70-75	Substance use disorder	3282	3.48 (3.48-3.48)	1469	3.48 (3.48-3.48)	1813	3.48 (3.48-3.48)
1970-1984	70-75	Substance use disorder	908	3.16 (3.16-3.17)	394	3.14 (3.14-3.15)	514	3.18 (3.17-3.18)
1985-1994	70-75	Substance use disorder	517	2.54 (2.54-2.55)	199	2.23 (2.22-2.23)	318	2.79 (2.78-2.79)
1995-2004	70-75	Substance use disorder	776	4.06 (4.06-4.07)	338	3.95 (3.95-3.96)	438	4.15 (4.14-4.16)
2005-2016	70-75	Substance use disorder	1081	4.13 (4.13-4.14)	538	4.40 (4.40-4.41)	543	3.90 (3.89-3.90)
1970-2016	75-80	Substance use disorder	2045	2.83 (2.83-2.84)	865	2.87 (2.87-2.88)	1180	2.81 (2.80-2.81)
1970-1984	75-80	Substance use disorder	403	1.97 (1.97-1.98)	163	1.97 (1.96-1.97)	240	1.98 (1.98-1.98)
1985-1994	75-80	Substance use disorder	288	1.77 (1.77-1.78)	97	1.48 (1.47-1.48)	191	1.97 (1.97-1.98)
1995-2004	75-80	Substance use disorder	583	3.63 (3.63-3.64)	246	3.71 (3.70-3.72)	337	3.58 (3.57-3.59)
2005-2016	75-80	Substance use disorder	771	3.97 (3.96-3.97)	359	4.17 (4.16-4.18)	412	3.80 (3.80-3.81)
1970-2016	80-85	Substance use disorder	1097	2.27 (2.26-2.27)	412	2.26 (2.25-2.26)	685	2.27 (2.27-2.28)
1970-1984	80-85	Substance use disorder	157	1.29 (1.29-1.29)	75	1.62 (1.61-1.62)	82	1.09 (1.08-1.09)
1985-1994	80-85	Substance use disorder	122	1.12 (1.12-1.12)	33	0.86 (0.85-0.86)	89	1.27 (1.26-1.27)
1995-2004	80-85	Substance use disorder	343	3.01 (3.00-3.02)	116	2.78 (2.77-2.78)	227	3.15 (3.14-3.15)
2005-2016	80-85	Substance use disorder	475	3.41 (3.40-3.41)	188	3.36 (3.35-3.36)	287	3.44 (3.43-3.45)
1970-2016	85-90	Substance use disorder	493	1.90 (1.90-1.90)	142	1.65 (1.65-1.66)	351	2.02 (2.01-2.02)
1970-1984	85-90	Substance use disorder	43	0.78 (0.78-0.78)	13	0.65 (0.65-0.66)	30	0.85 (0.85-0.85)
1985-1994	85-90	Substance use disorder	44	0.81 (0.80-0.81)	15	0.89 (0.89-0.90)	29	0.77 (0.76-0.77)
1995-2004	85-90	Substance use disorder	162	2.50 (2.50-2.51)	47	2.34 (2.33-2.35)	115	2.58 (2.57-2.58)
2005-2016	85-90	Substance use disorder	244	2.86 (2.86-2.87)	67	2.30 (2.30-2.31)	177	3.15 (3.15-3.16)
1970-2016	90-95	Substance use disorder	130	1.36 (1.36-1.37)	36	1.36 (1.36-1.37)	94	1.37 (1.36-1.37)
1970-1984	90-95	Substance use disorder	10	0.62 (0.62-0.63)	0-4	NA	6-10	0.86 (0.85-0.86)
1985-1994	90-95	Substance use disorder	10	0.54 (0.54-0.54)	5	0.99 (0.98-0.99)	5	0.37 (0.37-0.37)
1995-2004	90-95	Substance use disorder	47	1.91 (1.90-1.91)	12	1.96 (1.95-1.98)	35	1.89 (1.88-1.90)
2005-2016	90-95	Substance use disorder	63	1.75 (1.74-1.76)	18	1.85 (1.84-1.86)	45	1.71 (1.71-1.72)
1970-2016	95-100	Substance use disorder	17	0.86 (0.85-0.86)	0-4	NA	13-17	1.04 (1.03-1.04)
1970-1984	95-100	Substance use disorder	0-4	NA	0-4	NA	0-4	NA
1985-1994	95-100	Substance use disorder	0-4	NA	0-4	NA	0-4	NA
1995-2004	95-100	Substance use disorder	7	1.39 (1.37-1.40)	0-4	NA	3-7	1.73 (1.71-1.74)

			Both sexes		Males		Females	
**Years**	**Age**	**Mental disorder**	**Cases**	**IR (95% CI)**	**Cases**	**IR (95% CI)**	**Cases**	**IR (95% CI)**
2005-2016	95-100	Substance use disorder	10	1.14 (1.13-1.15)	0-4	NA	6-10	1.28 (1.28-1.29)
1970-2016	10-15	Substance use disorder (inpatient)	228	0.14 (0.14-0.14)	95	0.12 (0.12-0.12)	133	0.17 (0.17-0.17)
1970-1984	10-15	Substance use disorder (inpatient)	98	0.17 (0.17-0.17)	42	0.14 (0.14-0.14)	56	0.20 (0.20-0.20)
1985-1994	10-15	Substance use disorder (inpatient)	17	0.05 (0.05-0.05)	6	0.04 (0.04-0.04)	11	0.07 (0.07-0.07)
1995-2004	10-15	Substance use disorder (inpatient)	30	0.10 (0.10-0.10)	12	0.08 (0.08-0.08)	18	0.12 (0.12-0.13)
2005-2016	10-15	Substance use disorder (inpatient)	83	0.21 (0.21-0.21)	35	0.17 (0.17-0.17)	48	0.25 (0.24-0.25)
1970-2016	15-20	Substance use disorder (inpatient)	7522	4.68 (4.68-4.68)	4853	5.89 (5.88-5.89)	2669	3.41 (3.41-3.41)
1970-1984	15-20	Substance use disorder (inpatient)	3130	5.49 (5.48-5.49)	2019	6.89 (6.89-6.90)	1111	4.00 (4.00-4.01)
1985-1994	15-20	Substance use disorder (inpatient)	842	2.38 (2.38-2.39)	585	3.23 (3.23-3.24)	257	1.49 (1.49-1.50)
1995-2004	15-20	Substance use disorder (inpatient)	1132	3.98 (3.97-3.98)	756	5.19 (5.18-5.20)	376	2.71 (2.70-2.71)
2005-2016	15-20	Substance use disorder (inpatient)	2418	6.07 (6.06-6.07)	1493	7.30 (7.29-7.31)	925	4.77 (4.77-4.78)
1970-2016	20-25	Substance use disorder (inpatient)	14432	8.98 (8.97-8.98)	10407	12.54 (12.53-12.55)	4025	5.17 (5.17-5.18)
1970-1984	20-25	Substance use disorder (inpatient)	6050	10.95 (10.94-10.96)	4365	15.32 (15.31-15.34)	1685	6.30 (6.29-6.30)
1985-1994	20-25	Substance use disorder (inpatient)	2179	5.85 (5.85-5.86)	1597	8.25 (8.24-8.26)	582	3.26 (3.25-3.26)
1995-2004	20-25	Substance use disorder (inpatient)	2135	6.90 (6.90-6.91)	1615	10.11 (10.10-10.13)	520	3.48 (3.47-3.48)
2005-2016	20-25	Substance use disorder (inpatient)	4068	10.88 (10.87-10.90)	2830	14.76 (14.74-14.78)	1238	6.80 (6.79-6.81)
1970-2016	25-30	Substance use disorder (inpatient)	14553	9.04 (9.03-9.04)	10393	12.56 (12.55-12.57)	4160	5.31 (5.31-5.32)
1970-1984	25-30	Substance use disorder (inpatient)	6730	11.95 (11.94-11.96)	4656	16.14 (16.12-16.16)	2074	7.56 (7.55-7.57)
1985-1994	25-30	Substance use disorder (inpatient)	2840	7.74 (7.74-7.75)	2008	10.59 (10.57-10.60)	832	4.70 (4.69-4.71)
1995-2004	25-30	Substance use disorder (inpatient)	2112	6.29 (6.29-6.30)	1608	9.28 (9.27-9.30)	504	3.10 (3.10-3.11)
2005-2016	25-30	Substance use disorder (inpatient)	2871	8.33 (8.32-8.33)	2121	12.06 (12.04-12.08)	750	4.44 (4.43-4.44)
1970-2016	30-35	Substance use disorder (inpatient)	15941	10.01 (10.00-10.01)	10842	13.34 (13.33-13.35)	5099	6.54 (6.53-6.54)
1970-1984	30-35	Substance use disorder (inpatient)	7592	14.09 (14.08-14.11)	4944	18.07 (18.05-18.09)	2648	9.99 (9.98-10.00)
1985-1994	30-35	Substance use disorder (inpatient)	3356	9.81 (9.80-9.82)	2261	12.90 (12.88-12.92)	1095	6.56 (6.55-6.57)
1995-2004	30-35	Substance use disorder (inpatient)	2466	6.88 (6.88-6.89)	1769	9.61 (9.59-9.62)	697	4.00 (4.00-4.01)
2005-2016	30-35	Substance use disorder (inpatient)	2527	7.14 (7.13-7.15)	1868	10.38 (10.37-10.40)	659	3.79 (3.78-3.79)
1970-2016	35-40	Substance use disorder (inpatient)	16817	10.69 (10.68-10.70)	10808	13.56 (13.55-13.57)	6009	7.74 (7.74-7.75)
1970-1984	35-40	Substance use disorder (inpatient)	7727	15.77 (15.76-15.78)	4730	19.14 (19.12-19.17)	2997	12.34 (12.32-12.36)
1985-1994	35-40	Substance use disorder (inpatient)	3636	10.58 (10.57-10.59)	2295	13.21 (13.20-13.23)	1341	7.89 (7.87-7.90)
1995-2004	35-40	Substance use disorder (inpatient)	2862	8.04 (8.03-8.05)	1938	10.68 (10.66-10.69)	924	5.29 (5.29-5.30)
2005-2016	35-40	Substance use disorder (inpatient)	2592	6.76 (6.75-6.77)	1845	9.48 (9.46-9.49)	747	3.96 (3.95-3.96)
1970-2016	40-45	Substance use disorder (inpatient)	16687	10.86 (10.86-10.87)	10213	13.22 (13.21-13.23)	6474	8.48 (8.47-8.48)
1970-1984	40-45	Substance use disorder (inpatient)	7173	16.71 (16.69-16.72)	4316	20.15 (20.12-20.18)	2857	13.28 (13.26-13.30)
1985-1994	40-45	Substance use disorder (inpatient)	3606	9.97 (9.96-9.98)	2064	11.37 (11.36-11.39)	1542	8.55 (8.54-8.57)
1995-2004	40-45	Substance use disorder (inpatient)	2886	8.67 (8.66-8.68)	1779	10.57 (10.56-10.59)	1107	6.72 (6.71-6.73)
2005-2016	40-45	Substance use disorder (inpatient)	3022	7.33 (7.32-7.34)	2054	9.84 (9.82-9.85)	968	4.76 (4.75-4.76)
1970-2016	45-50	Substance use disorder (inpatient)	15421	10.34 (10.33-10.34)	9206	12.37 (12.36-12.37)	6215	8.32 (8.31-8.32)
1970-1984	45-50	Substance use disorder (inpatient)	6673	16.06 (16.04-16.08)	4000	19.49 (19.47-19.52)	2673	12.71 (12.69-12.73)
1985-1994	45-50	Substance use disorder (inpatient)	2853	8.60 (8.59-8.61)	1614	9.74 (9.73-9.75)	1239	7.46 (7.45-7.48)
1995-2004	45-50	Substance use disorder (inpatient)	2575	7.75 (7.74-7.75)	1497	9.01 (8.99-9.02)	1078	6.48 (6.47-6.49)

			Both sexes		Males		Females	
**Years**	**Age**	**Mental disorder**	**Cases**	**IR (95% CI)**	**Cases**	**IR (95% CI)**	**Cases**	**IR (95% CI)**
2005-2016	45-50	Substance use disorder (inpatient)	3320	8.05 (8.05-8.06)	2095	10.10 (10.09-10.12)	1225	5.98 (5.97-5.99)
1970-2016	50-55	Substance use disorder (inpatient)	12606	8.83 (8.83-8.83)	7292	10.32 (10.31-10.33)	5314	7.37 (7.37-7.38)
1970-1984	50-55	Substance use disorder (inpatient)	5569	13.31 (13.29-13.32)	3264	15.91 (15.88-15.93)	2305	10.81 (10.79-10.82)
1985-1994	50-55	Substance use disorder (inpatient)	1957	7.13 (7.12-7.14)	1044	7.70 (7.69-7.72)	913	6.57 (6.56-6.58)
1995-2004	50-55	Substance use disorder (inpatient)	2232	6.46 (6.45-6.46)	1263	7.35 (7.34-7.37)	969	5.57 (5.56-5.58)
2005-2016	50-55	Substance use disorder (inpatient)	2848	7.32 (7.32-7.33)	1721	8.86 (8.85-8.87)	1127	5.79 (5.78-5.80)
1970-2016	55-60	Substance use disorder (inpatient)	9410	6.96 (6.96-6.97)	5364	8.10 (8.09-8.11)	4046	5.87 (5.87-5.87)
1970-1984	55-60	Substance use disorder (inpatient)	4270	10.30 (10.29-10.31)	2390	11.87 (11.85-11.89)	1880	8.82 (8.81-8.84)
1985-1994	55-60	Substance use disorder (inpatient)	1375	5.52 (5.52-5.53)	741	6.12 (6.11-6.13)	634	4.96 (4.95-4.96)
1995-2004	55-60	Substance use disorder (inpatient)	1524	4.89 (4.88-4.89)	832	5.41 (5.40-5.42)	692	4.38 (4.38-4.39)
2005-2016	55-60	Substance use disorder (inpatient)	2241	5.96 (5.95-5.96)	1401	7.54 (7.52-7.55)	840	4.41 (4.41-4.42)
1970-2016	60-65	Substance use disorder (inpatient)	6442	5.09 (5.09-5.09)	3507	5.75 (5.74-5.75)	2935	4.48 (4.47-4.48)
1970-1984	60-65	Substance use disorder (inpatient)	2811	7.11 (7.10-7.12)	1522	8.05 (8.04-8.06)	1289	6.24 (6.24-6.25)
1985-1994	60-65	Substance use disorder (inpatient)	1065	4.40 (4.39-4.40)	545	4.73 (4.72-4.74)	520	4.09 (4.09-4.10)
1995-2004	60-65	Substance use disorder (inpatient)	961	3.84 (3.84-3.85)	497	4.11 (4.10-4.11)	464	3.60 (3.59-3.60)
2005-2016	60-65	Substance use disorder (inpatient)	1605	4.25 (4.24-4.25)	943	5.10 (5.09-5.11)	662	3.43 (3.42-3.43)
1970-2016	65-70	Substance use disorder (inpatient)	4082	3.59 (3.59-3.59)	2108	3.95 (3.95-3.95)	1974	3.27 (3.26-3.27)
1970-1984	65-70	Substance use disorder (inpatient)	1735	4.94 (4.93-4.94)	868	5.35 (5.34-5.36)	867	4.59 (4.58-4.59)
1985-1994	65-70	Substance use disorder (inpatient)	700	3.01 (3.01-3.01)	346	3.23 (3.22-3.23)	354	2.82 (2.82-2.83)
1995-2004	65-70	Substance use disorder (inpatient)	601	2.80 (2.79-2.80)	308	3.05 (3.05-3.06)	293	2.57 (2.57-2.58)
2005-2016	65-70	Substance use disorder (inpatient)	1046	3.08 (3.08-3.09)	586	3.59 (3.58-3.60)	460	2.62 (2.61-2.62)
1970-2016	70-75	Substance use disorder (inpatient)	2382	2.52 (2.52-2.53)	1044	2.47 (2.47-2.47)	1338	2.57 (2.56-2.57)
1970-1984	70-75	Substance use disorder (inpatient)	908	3.16 (3.16-3.17)	394	3.14 (3.14-3.15)	514	3.18 (3.17-3.18)
1985-1994	70-75	Substance use disorder (inpatient)	509	2.50 (2.50-2.51)	195	2.18 (2.18-2.19)	314	2.75 (2.75-2.76)
1995-2004	70-75	Substance use disorder (inpatient)	400	2.09 (2.09-2.10)	167	1.95 (1.95-1.96)	233	2.21 (2.20-2.21)
2005-2016	70-75	Substance use disorder (inpatient)	565	2.15 (2.15-2.16)	288	2.35 (2.35-2.35)	277	1.98 (1.98-1.99)
1970-2016	75-80	Substance use disorder (inpatient)	1255	1.74 (1.74-1.74)	520	1.73 (1.72-1.73)	735	1.75 (1.75-1.75)
1970-1984	75-80	Substance use disorder (inpatient)	403	1.97 (1.97-1.98)	163	1.97 (1.96-1.97)	240	1.98 (1.98-1.98)
1985-1994	75-80	Substance use disorder (inpatient)	281	1.73 (1.73-1.73)	95	1.45 (1.44-1.45)	186	1.92 (1.92-1.93)
1995-2004	75-80	Substance use disorder (inpatient)	253	1.58 (1.57-1.58)	103	1.55 (1.55-1.56)	150	1.59 (1.59-1.60)
2005-2016	75-80	Substance use disorder (inpatient)	318	1.63 (1.63-1.63)	159	1.84 (1.84-1.85)	159	1.46 (1.46-1.47)
1970-2016	80-85	Substance use disorder (inpatient)	551	1.14 (1.14-1.14)	214	1.17 (1.17-1.17)	337	1.12 (1.12-1.12)
1970-1984	80-85	Substance use disorder (inpatient)	157	1.29 (1.29-1.29)	75	1.62 (1.61-1.62)	82	1.09 (1.08-1.09)
1985-1994	80-85	Substance use disorder (inpatient)	119	1.09 (1.09-1.10)	31	0.80 (0.80-0.81)	88	1.25 (1.25-1.26)
1995-2004	80-85	Substance use disorder (inpatient)	126	1.11 (1.10-1.11)	47	1.12 (1.12-1.13)	79	1.09 (1.09-1.10)
2005-2016	80-85	Substance use disorder (inpatient)	149	1.07 (1.06-1.07)	61	1.09 (1.08-1.09)	88	1.05 (1.05-1.06)
1970-2016	85-90	Substance use disorder (inpatient)	184	0.71 (0.71-0.71)	58	0.68 (0.67-0.68)	126	0.72 (0.72-0.72)
1970-1984	85-90	Substance use disorder (inpatient)	43	0.78 (0.78-0.78)	13	0.65 (0.65-0.66)	30	0.85 (0.85-0.85)
1985-1994	85-90	Substance use disorder (inpatient)	43	0.79 (0.78-0.79)	15	0.89 (0.89-0.90)	28	0.74 (0.74-0.74)
1995-2004	85-90	Substance use disorder (inpatient)	47	0.73 (0.72-0.73)	13	0.65 (0.64-0.65)	34	0.76 (0.76-0.76)

			Both sexes		Males		Females	
Years	Age	Mental disorder	Cases	IR (95% CI)	Cases	IR (95% CI)	Cases	IR (95% CI)
2005-2016	85-90	Substance use disorder (inpatient)	51	0.60 (0.60-0.60)	17	0.58 (0.58-0.59)	34	0.60 (0.60-0.61)
1970-2016	90-95	Substance use disorder (inpatient)	47	0.49 (0.49-0.49)	15	0.57 (0.57-0.57)	32	0.46 (0.46-0.47)
1970-1984	90-95	Substance use disorder (inpatient)	10	0.62 (0.62-0.63)	0-4	NA	6-10	0.86 (0.85-0.86)
1985-1994	90-95	Substance use disorder (inpatient)	10	0.54 (0.54-0.54)	5	0.99 (0.98-0.99)	5	0.37 (0.37-0.37)
1995-2004	90-95	Substance use disorder (inpatient)	14	0.57 (0.57-0.57)	5	0.82 (0.81-0.82)	9	0.48 (0.48-0.49)
2005-2016	90-95	Substance use disorder (inpatient)	13	0.36 (0.36-0.36)	0-4	NA	9-13	0.34 (0.34-0.34)
1970-2016	95-100	Substance use disorder (inpatient)	0-4	NA	0-4	NA	0-4	NA
1970-1984	95-100	Substance use disorder (inpatient)	0-4	NA	0-4	NA	0-4	NA
1985-1994	95-100	Substance use disorder (inpatient)	0-4	NA	0-4	NA	0-4	NA
1995-2004	95-100	Substance use disorder (inpatient)	0-4	NA	0-4	NA	0-4	NA
2005-2016	95-100	Substance use disorder (inpatient)	0-4	NA	0-4	NA	0-4	NA
1970-2016	10-15	Alcohol use disorder	102	0.06 (0.06-0.06)	42	0.05 (0.05-0.05)	60	0.08 (0.08-0.08)
1970-1984	10-15	Alcohol use disorder	32	0.06 (0.06-0.06)	15	0.05 (0.05-0.05)	17	0.06 (0.06-0.06)
1985-1994	10-15	Alcohol use disorder	10	0.03 (0.03-0.03)	0-4	NA	6-10	0.05 (0.05-0.05)
1995-2004	10-15	Alcohol use disorder	18	0.06 (0.06-0.06)	7	0.05 (0.05-0.05)	11	0.08 (0.08-0.08)
2005-2016	10-15	Alcohol use disorder	42	0.10 (0.10-0.10)	18	0.09 (0.09-0.09)	24	0.12 (0.12-0.12)
1970-2016	15-20	Alcohol use disorder	3123	1.94 (1.94-1.94)	1899	2.30 (2.30-2.30)	1224	1.56 (1.56-1.57)
1970-1984	15-20	Alcohol use disorder	1036	1.81 (1.81-1.82)	676	2.31 (2.30-2.31)	360	1.30 (1.29-1.30)
1985-1994	15-20	Alcohol use disorder	327	0.93 (0.93-0.93)	229	1.26 (1.26-1.27)	98	0.57 (0.57-0.57)
1995-2004	15-20	Alcohol use disorder	545	1.92 (1.91-1.92)	346	2.38 (2.37-2.38)	199	1.43 (1.43-1.43)
2005-2016	15-20	Alcohol use disorder	1215	3.05 (3.04-3.05)	648	3.17 (3.16-3.17)	567	2.92 (2.92-2.93)
1970-2016	20-25	Alcohol use disorder	8748	5.43 (5.43-5.43)	6257	7.52 (7.51-7.52)	2491	3.20 (3.20-3.20)
1970-1984	20-25	Alcohol use disorder	3621	6.53 (6.53-6.54)	2672	9.35 (9.33-9.36)	949	3.54 (3.53-3.54)
1985-1994	20-25	Alcohol use disorder	1204	3.23 (3.23-3.23)	872	4.50 (4.49-4.50)	332	1.86 (1.85-1.86)
1995-2004	20-25	Alcohol use disorder	1462	4.72 (4.71-4.73)	1073	6.71 (6.69-6.72)	389	2.60 (2.59-2.60)
2005-2016	20-25	Alcohol use disorder	2461	6.57 (6.56-6.58)	1640	8.53 (8.51-8.54)	821	4.50 (4.50-4.51)
1970-2016	25-30	Alcohol use disorder	12297	7.62 (7.61-7.62)	9071	10.93 (10.92-10.94)	3226	4.11 (4.11-4.12)
1970-1984	25-30	Alcohol use disorder	5561	9.85 (9.84-9.85)	4101	14.15 (14.14-14.17)	1460	5.31 (5.30-5.31)
1985-1994	25-30	Alcohol use disorder	2200	5.99 (5.98-5.99)	1577	8.29 (8.28-8.30)	623	3.51 (3.51-3.52)
1995-2004	25-30	Alcohol use disorder	2235	6.65 (6.64-6.66)	1717	9.89 (9.88-9.91)	518	3.19 (3.18-3.19)
2005-2016	25-30	Alcohol use disorder	2301	6.65 (6.65-6.66)	1676	9.50 (9.48-9.51)	625	3.69 (3.69-3.70)
1970-2016	30-35	Alcohol use disorder	15584	9.76 (9.76-9.76)	11112	13.63 (13.62-13.64)	4472	5.72 (5.72-5.73)
1970-1984	30-35	Alcohol use disorder	6752	12.51 (12.49-12.52)	4735	17.26 (17.24-17.28)	2017	7.59 (7.58-7.60)
1985-1994	30-35	Alcohol use disorder	3013	8.77 (8.76-8.78)	2091	11.87 (11.86-11.89)	922	5.51 (5.50-5.52)
1995-2004	30-35	Alcohol use disorder	3268	9.11 (9.10-9.12)	2389	12.96 (12.94-12.97)	879	5.04 (5.04-5.05)
2005-2016	30-35	Alcohol use disorder	2551	7.19 (7.19-7.20)	1897	10.51 (10.50-10.53)	654	3.75 (3.75-3.76)
1970-2016	35-40	Alcohol use disorder	17421	11.05 (11.05-11.06)	11877	14.88 (14.87-14.89)	5544	7.13 (7.12-7.13)
1970-1984	35-40	Alcohol use disorder	6681	13.61 (13.60-13.62)	4505	18.21 (18.19-18.23)	2176	8.94 (8.93-8.95)
1985-1994	35-40	Alcohol use disorder	3434	9.94 (9.93-9.95)	2259	12.93 (12.91-12.95)	1175	6.88 (6.87-6.89)
1995-2004	35-40	Alcohol use disorder	4269	11.98 (11.97-12.00)	2927	16.12 (16.10-16.15)	1342	7.68 (7.67-7.69)

			Both sexes		Males		Females	
Years	Age	Mental disorder	Cases	IR (95% CI)	Cases	IR (95% CI)	Cases	IR (95% CI)
2005-2016	35-40	Alcohol use disorder	3037	7.92 (7.91-7.92)	2186	11.22 (11.20-11.24)	851	4.51 (4.50-4.51)
1970-2016	40-45	Alcohol use disorder	17880	11.62 (11.62-11.63)	11534	14.92 (14.91-14.93)	6346	8.29 (8.29-8.30)
1970-1984	40-45	Alcohol use disorder	6192	14.39 (14.38-14.41)	4121	19.22 (19.20-19.25)	2071	9.59 (9.58-9.61)
1985-1994	40-45	Alcohol use disorder	3412	9.40 (9.39-9.41)	2055	11.29 (11.27-11.30)	1357	7.50 (7.49-7.51)
1995-2004	40-45	Alcohol use disorder	4524	13.57 (13.56-13.59)	2842	16.88 (16.85-16.91)	1682	10.20 (10.18-10.21)
2005-2016	40-45	Alcohol use disorder	3752	9.11 (9.10-9.12)	2516	12.07 (12.05-12.09)	1236	6.08 (6.07-6.08)
1970-2016	45-50	Alcohol use disorder	16683	11.17 (11.17-11.18)	10534	14.15 (14.14-14.16)	6149	8.21 (8.20-8.22)
1970-1984	45-50	Alcohol use disorder	5590	13.42 (13.41-13.44)	3767	18.34 (18.31-18.36)	1823	8.64 (8.63-8.65)
1985-1994	45-50	Alcohol use disorder	2655	7.98 (7.97-7.99)	1586	9.56 (9.54-9.57)	1069	6.41 (6.40-6.42)
1995-2004	45-50	Alcohol use disorder	4257	12.78 (12.77-12.80)	2570	15.45 (15.42-15.47)	1687	10.13 (10.11-10.14)
2005-2016	45-50	Alcohol use disorder	4181	10.17 (10.16-10.18)	2611	12.63 (12.61-12.65)	1570	7.67 (7.66-7.68)
1970-2016	50-55	Alcohol use disorder	13362	9.35 (9.35-9.36)	8427	11.93 (11.92-11.94)	4935	6.83 (6.82-6.83)
1970-1984	50-55	Alcohol use disorder	4474	10.67 (10.65-10.68)	3073	14.96 (14.94-14.98)	1401	6.54 (6.54-6.55)
1985-1994	50-55	Alcohol use disorder	1777	6.46 (6.45-6.46)	1022	7.53 (7.52-7.55)	755	5.41 (5.40-5.42)
1995-2004	50-55	Alcohol use disorder	3525	10.18 (10.17-10.20)	2119	12.34 (12.32-12.36)	1406	8.06 (8.05-8.07)
2005-2016	50-55	Alcohol use disorder	3586	9.24 (9.23-9.25)	2213	11.43 (11.41-11.45)	1373	7.06 (7.05-7.07)
1970-2016	55-60	Alcohol use disorder	9865	7.29 (7.29-7.30)	6200	9.37 (9.36-9.38)	3665	5.30 (5.30-5.31)
1970-1984	55-60	Alcohol use disorder	3280	7.90 (7.89-7.90)	2190	10.87 (10.85-10.88)	1090	5.10 (5.09-5.10)
1985-1994	55-60	Alcohol use disorder	1203	4.82 (4.81-4.82)	711	5.87 (5.86-5.88)	492	3.83 (3.82-3.83)
1995-2004	55-60	Alcohol use disorder	2424	7.77 (7.76-7.78)	1410	9.18 (9.16-9.19)	1014	6.40 (6.39-6.41)
2005-2016	55-60	Alcohol use disorder	2958	7.87 (7.87-7.88)	1889	10.19 (10.17-10.20)	1069	5.62 (5.61-5.63)
1970-2016	60-65	Alcohol use disorder	6417	5.06 (5.06-5.07)	3964	6.50 (6.49-6.50)	2453	3.73 (3.73-3.73)
1970-1984	60-65	Alcohol use disorder	1981	5.00 (4.99-5.00)	1333	7.05 (7.04-7.06)	648	3.13 (3.13-3.13)
1985-1994	60-65	Alcohol use disorder	872	3.59 (3.58-3.59)	508	4.40 (4.39-4.41)	364	2.85 (2.85-2.86)
1995-2004	60-65	Alcohol use disorder	1440	5.75 (5.74-5.76)	845	6.99 (6.97-7.00)	595	4.59 (4.59-4.60)
2005-2016	60-65	Alcohol use disorder	2124	5.62 (5.62-5.63)	1278	6.93 (6.92-6.94)	846	4.38 (4.37-4.38)
1970-2016	65-70	Alcohol use disorder	3917	3.44 (3.44-3.44)	2367	4.44 (4.43-4.44)	1550	2.56 (2.56-2.56)
1970-1984	65-70	Alcohol use disorder	1071	3.04 (3.04-3.05)	709	4.37 (4.36-4.37)	362	1.91 (1.91-1.91)
1985-1994	65-70	Alcohol use disorder	498	2.13 (2.13-2.14)	297	2.77 (2.76-2.77)	201	1.59 (1.59-1.60)
1995-2004	65-70	Alcohol use disorder	911	4.23 (4.23-4.24)	525	5.20 (5.19-5.21)	386	3.38 (3.37-3.38)
2005-2016	65-70	Alcohol use disorder	1437	4.24 (4.24-4.25)	836	5.13 (5.13-5.14)	601	3.41 (3.41-3.42)
1970-2016	70-75	Alcohol use disorder	2268	2.40 (2.40-2.40)	1252	2.96 (2.96-2.97)	1016	1.94 (1.94-1.95)
1970-1984	70-75	Alcohol use disorder	483	1.68 (1.68-1.68)	296	2.36 (2.36-2.36)	187	1.15 (1.15-1.16)
1985-1994	70-75	Alcohol use disorder	322	1.58 (1.58-1.58)	168	1.88 (1.88-1.88)	154	1.34 (1.34-1.35)
1995-2004	70-75	Alcohol use disorder	594	3.10 (3.10-3.10)	300	3.51 (3.50-3.51)	294	2.77 (2.77-2.78)
2005-2016	70-75	Alcohol use disorder	869	3.31 (3.31-3.32)	488	3.99 (3.98-4.00)	381	2.72 (2.72-2.73)
1970-2016	75-80	Alcohol use disorder	1276	1.76 (1.76-1.77)	688	2.28 (2.28-2.29)	588	1.39 (1.39-1.40)
1970-1984	75-80	Alcohol use disorder	192	0.94 (0.94-0.94)	110	1.33 (1.32-1.33)	82	0.68 (0.67-0.68)
1985-1994	75-80	Alcohol use disorder	154	0.95 (0.94-0.95)	67	1.02 (1.02-1.02)	87	0.90 (0.89-0.90)
1995-2004	75-80	Alcohol use disorder	368	2.29 (2.28-2.29)	201	3.03 (3.02-3.04)	167	1.77 (1.76-1.77)

			Both sexes		Males		Females	
**Years**	**Age**	**Mental disorder**	**Cases**	**IR (95% CI)**	**Cases**	**IR (95% CI)**	**Cases**	**IR (95% CI)**
2005-2016	75-80	Alcohol use disorder	562	2.88 (2.88-2.89)	310	3.59 (3.59-3.60)	252	2.32 (2.31-2.32)
1970-2016	80-85	Alcohol use disorder	600	1.24 (1.24-1.24)	296	1.62 (1.62-1.62)	304	1.01 (1.00-1.01)
1970-1984	80-85	Alcohol use disorder	67	0.55 (0.55-0.55)	43	0.93 (0.93-0.93)	24	0.32 (0.32-0.32)
1985-1994	80-85	Alcohol use disorder	44	0.40 (0.40-0.40)	19	0.49 (0.49-0.49)	25	0.36 (0.35-0.36)
1995-2004	80-85	Alcohol use disorder	183	1.60 (1.60-1.60)	89	2.13 (2.12-2.13)	94	1.30 (1.29-1.30)
2005-2016	80-85	Alcohol use disorder	306	2.19 (2.18-2.19)	145	2.58 (2.58-2.59)	161	1.92 (1.92-1.93)
1970-2016	85-90	Alcohol use disorder	213	0.82 (0.82-0.82)	94	1.09 (1.09-1.10)	119	0.68 (0.68-0.68)
1970-1984	85-90	Alcohol use disorder	11	0.20 (0.20-0.20)	7-11	0.35 (0.35-0.35)	0-4	NA
1985-1994	85-90	Alcohol use disorder	17	0.31 (0.31-0.31)	9	0.54 (0.53-0.54)	8	0.21 (0.21-0.21)
1995-2004	85-90	Alcohol use disorder	67	1.03 (1.03-1.04)	28	1.39 (1.39-1.40)	39	0.87 (0.87-0.87)
2005-2016	85-90	Alcohol use disorder	118	1.38 (1.38-1.38)	50	1.72 (1.71-1.72)	68	1.21 (1.20-1.21)
1970-2016	90-95	Alcohol use disorder	37	0.39 (0.39-0.39)	13	0.49 (0.49-0.49)	24	0.35 (0.35-0.35)
1970-1984	90-95	Alcohol use disorder	0-4	NA	0-4	NA	0-4	NA
1985-1994	90-95	Alcohol use disorder	0-4	NA	0-4	NA	0-4	NA
1995-2004	90-95	Alcohol use disorder	10	0.40 (0.40-0.41)	0-4	NA	6-10	0.38 (0.37-0.38)
2005-2016	90-95	Alcohol use disorder	23	0.64 (0.63-0.64)	9	0.93 (0.92-0.93)	14	0.53 (0.53-0.53)
1970-2016	95-100	Alcohol use disorder	0-4	NA	0-4	NA	0-4	NA
1970-1984	95-100	Alcohol use disorder	0-4	NA	0-4	NA	0-4	NA
1985-1994	95-100	Alcohol use disorder	0-4	NA	0-4	NA	0-4	NA
1995-2004	95-100	Alcohol use disorder	0-4	NA	0-4	NA	0-4	NA
2005-2016	95-100	Alcohol use disorder	0-4	NA	0-4	NA	0-4	NA
1970-2016	10-15	Alcohol use disorder (inpatient)	63	0.04 (0.04-0.04)	27	0.03 (0.03-0.03)	36	0.05 (0.05-0.05)
1970-1984	10-15	Alcohol use disorder (inpatient)	32	0.06 (0.06-0.06)	15	0.05 (0.05-0.05)	17	0.06 (0.06-0.06)
1985-1994	10-15	Alcohol use disorder (inpatient)	10	0.03 (0.03-0.03)	0-4	NA	6-10	0.05 (0.05-0.05)
1995-2004	10-15	Alcohol use disorder (inpatient)	5	0.02 (0.02-0.02)	0-4	NA	0-4	NA
2005-2016	10-15	Alcohol use disorder (inpatient)	16	0.04 (0.04-0.04)	7	0.03 (0.03-0.03)	9	0.05 (0.05-0.05)
1970-2016	15-20	Alcohol use disorder (inpatient)	2135	1.33 (1.33-1.33)	1354	1.64 (1.64-1.64)	781	1.00 (1.00-1.00)
1970-1984	15-20	Alcohol use disorder (inpatient)	1036	1.81 (1.81-1.82)	676	2.31 (2.30-2.31)	360	1.30 (1.29-1.30)
1985-1994	15-20	Alcohol use disorder (inpatient)	326	0.92 (0.92-0.92)	228	1.26 (1.26-1.26)	98	0.57 (0.57-0.57)
1995-2004	15-20	Alcohol use disorder (inpatient)	247	0.87 (0.87-0.87)	163	1.12 (1.12-1.12)	84	0.60 (0.60-0.61)
2005-2016	15-20	Alcohol use disorder (inpatient)	526	1.32 (1.32-1.32)	287	1.40 (1.40-1.40)	239	1.23 (1.23-1.23)
1970-2016	20-25	Alcohol use disorder (inpatient)	6756	4.19 (4.19-4.19)	4850	5.82 (5.82-5.83)	1906	2.45 (2.44-2.45)
1970-1984	20-25	Alcohol use disorder (inpatient)	3621	6.53 (6.53-6.54)	2672	9.35 (9.33-9.36)	949	3.54 (3.53-3.54)
1985-1994	20-25	Alcohol use disorder (inpatient)	1185	3.18 (3.18-3.18)	857	4.42 (4.41-4.43)	328	1.83 (1.83-1.84)
1995-2004	20-25	Alcohol use disorder (inpatient)	689	2.22 (2.22-2.23)	503	3.14 (3.14-3.15)	186	1.24 (1.24-1.24)
2005-2016	20-25	Alcohol use disorder (inpatient)	1261	3.36 (3.36-3.36)	818	4.24 (4.24-4.25)	443	2.43 (2.42-2.43)
1970-2016	25-30	Alcohol use disorder (inpatient)	10247	6.34 (6.34-6.35)	7512	9.04 (9.03-9.05)	2735	3.49 (3.48-3.49)
1970-1984	25-30	Alcohol use disorder (inpatient)	5561	9.85 (9.84-9.85)	4101	14.15 (14.14-14.17)	1460	5.31 (5.30-5.31)
1985-1994	25-30	Alcohol use disorder (inpatient)	2146	5.84 (5.83-5.84)	1531	8.05 (8.04-8.06)	615	3.47 (3.46-3.47)
1995-2004	25-30	Alcohol use disorder (inpatient)	1178	3.50 (3.50-3.50)	892	5.13 (5.12-5.14)	286	1.76 (1.76-1.76)

			Both sexes		Males		Females	
Years	Age	Mental disorder	Cases	IR (95% CI)	Cases	IR (95% CI)	Cases	IR (95% CI)
2005-2016	25-30	Alcohol use disorder (inpatient)	1362	3.93 (3.92-3.93)	988	5.58 (5.57-5.58)	374	2.21 (2.20-2.21)
1970-2016	30-35	Alcohol use disorder (inpatient)	13119	8.21 (8.20-8.21)	9275	11.36 (11.35-11.37)	3844	4.92 (4.91-4.92)
1970-1984	30-35	Alcohol use disorder (inpatient)	6752	12.51 (12.49-12.52)	4735	17.26 (17.24-17.28)	2017	7.59 (7.58-7.60)
1985-1994	30-35	Alcohol use disorder (inpatient)	2956	8.61 (8.60-8.61)	2047	11.62 (11.61-11.64)	909	5.43 (5.42-5.44)
1995-2004	30-35	Alcohol use disorder (inpatient)	1796	5.00 (4.99-5.00)	1290	6.98 (6.97-6.99)	506	2.90 (2.90-2.91)
2005-2016	30-35	Alcohol use disorder (inpatient)	1615	4.54 (4.53-4.54)	1203	6.64 (6.63-6.65)	412	2.36 (2.36-2.36)
1970-2016	35-40	Alcohol use disorder (inpatient)	14430	9.14 (9.14-9.15)	9763	12.20 (12.19-12.21)	4667	6.00 (5.99-6.00)
1970-1984	35-40	Alcohol use disorder (inpatient)	6681	13.61 (13.60-13.62)	4505	18.21 (18.19-18.23)	2176	8.94 (8.93-8.95)
1985-1994	35-40	Alcohol use disorder (inpatient)	3357	9.72 (9.71-9.73)	2214	12.68 (12.66-12.69)	1143	6.69 (6.68-6.70)
1995-2004	35-40	Alcohol use disorder (inpatient)	2393	6.70 (6.69-6.71)	1622	8.90 (8.89-8.91)	771	4.41 (4.40-4.41)
2005-2016	35-40	Alcohol use disorder (inpatient)	1999	5.19 (5.18-5.19)	1422	7.26 (7.25-7.27)	577	3.05 (3.05-3.05)
1970-2016	40-45	Alcohol use disorder (inpatient)	14711	9.54 (9.54-9.55)	9523	12.28 (12.27-12.29)	5188	6.77 (6.77-6.78)
1970-1984	40-45	Alcohol use disorder (inpatient)	6192	14.39 (14.38-14.41)	4121	19.22 (19.20-19.25)	2071	9.59 (9.58-9.61)
1985-1994	40-45	Alcohol use disorder (inpatient)	3327	9.16 (9.15-9.17)	1990	10.93 (10.91-10.94)	1337	7.39 (7.38-7.40)
1995-2004	40-45	Alcohol use disorder (inpatient)	2600	7.78 (7.77-7.78)	1634	9.66 (9.65-9.68)	966	5.84 (5.83-5.85)
2005-2016	40-45	Alcohol use disorder (inpatient)	2592	6.26 (6.26-6.27)	1778	8.47 (8.46-8.48)	814	3.99 (3.98-4.00)
1970-2016	45-50	Alcohol use disorder (inpatient)	13565	9.06 (9.06-9.07)	8640	11.57 (11.56-11.58)	4925	6.57 (6.56-6.57)
1970-1984	45-50	Alcohol use disorder (inpatient)	5590	13.42 (13.41-13.44)	3767	18.34 (18.31-18.36)	1823	8.64 (8.63-8.65)
1985-1994	45-50	Alcohol use disorder (inpatient)	2597	7.81 (7.80-7.82)	1547	9.32 (9.31-9.34)	1050	6.30 (6.29-6.31)
1995-2004	45-50	Alcohol use disorder (inpatient)	2385	7.14 (7.13-7.15)	1414	8.46 (8.45-8.48)	971	5.81 (5.81-5.82)
2005-2016	45-50	Alcohol use disorder (inpatient)	2993	7.23 (7.23-7.24)	1912	9.18 (9.16-9.19)	1081	5.26 (5.26-5.27)
1970-2016	50-55	Alcohol use disorder (inpatient)	10936	7.63 (7.63-7.64)	6940	9.80 (9.79-9.80)	3996	5.52 (5.52-5.52)
1970-1984	50-55	Alcohol use disorder (inpatient)	4474	10.67 (10.65-10.68)	3073	14.96 (14.94-14.98)	1401	6.54 (6.54-6.55)
1985-1994	50-55	Alcohol use disorder (inpatient)	1737	6.31 (6.30-6.32)	992	7.31 (7.30-7.32)	745	5.34 (5.33-5.35)
1995-2004	50-55	Alcohol use disorder (inpatient)	2047	5.90 (5.89-5.90)	1208	7.01 (7.00-7.02)	839	4.80 (4.79-4.81)
2005-2016	50-55	Alcohol use disorder (inpatient)	2678	6.86 (6.85-6.86)	1667	8.54 (8.53-8.55)	1011	5.17 (5.17-5.18)
1970-2016	55-60	Alcohol use disorder (inpatient)	7946	5.86 (5.86-5.86)	5021	7.57 (7.56-7.57)	2925	4.23 (4.22-4.23)
1970-1984	55-60	Alcohol use disorder (inpatient)	3280	7.90 (7.89-7.90)	2190	10.87 (10.85-10.88)	1090	5.10 (5.09-5.10)
1985-1994	55-60	Alcohol use disorder (inpatient)	1185	4.74 (4.74-4.75)	696	5.74 (5.73-5.75)	489	3.80 (3.80-3.81)
1995-2004	55-60	Alcohol use disorder (inpatient)	1388	4.44 (4.43-4.44)	803	5.21 (5.20-5.22)	585	3.69 (3.68-3.69)
2005-2016	55-60	Alcohol use disorder (inpatient)	2093	5.54 (5.53-5.54)	1332	7.13 (7.12-7.14)	761	3.98 (3.98-3.99)
1970-2016	60-65	Alcohol use disorder (inpatient)	5156	4.06 (4.06-4.06)	3228	5.28 (5.28-5.29)	1928	2.93 (2.93-2.93)
1970-1984	60-65	Alcohol use disorder (inpatient)	1981	5.00 (4.99-5.00)	1333	7.05 (7.04-7.06)	648	3.13 (3.13-3.13)
1985-1994	60-65	Alcohol use disorder (inpatient)	863	3.55 (3.55-3.55)	505	4.38 (4.37-4.38)	358	2.80 (2.80-2.81)
1995-2004	60-65	Alcohol use disorder (inpatient)	830	3.31 (3.30-3.31)	470	3.88 (3.87-3.89)	360	2.78 (2.77-2.78)
2005-2016	60-65	Alcohol use disorder (inpatient)	1482	3.91 (3.90-3.91)	920	4.96 (4.96-4.97)	562	2.90 (2.89-2.90)
1970-2016	65-70	Alcohol use disorder (inpatient)	3004	2.63 (2.63-2.63)	1841	3.45 (3.44-3.45)	1163	1.92 (1.92-1.92)
1970-1984	65-70	Alcohol use disorder (inpatient)	1071	3.04 (3.04-3.05)	709	4.37 (4.36-4.37)	362	1.91 (1.91-1.91)
1985-1994	65-70	Alcohol use disorder (inpatient)	494	2.12 (2.11-2.12)	296	2.76 (2.75-2.76)	198	1.57 (1.57-1.57)
1995-2004	65-70	Alcohol use disorder (inpatient)	500	2.32 (2.32-2.32)	286	2.83 (2.83-2.84)	214	1.87 (1.87-1.87)

			Both sexes		Males		Females	
**Years**	**Age**	**Mental disorder**	**Cases**	**IR (95% CI)**	**Cases**	**IR (95% CI)**	**Cases**	**IR (95% CI)**
2005-2016	65-70	Alcohol use disorder (inpatient)	939	2.76 (2.76-2.76)	550	3.36 (3.36-3.37)	389	2.20 (2.20-2.21)
1970-2016	70-75	Alcohol use disorder (inpatient)	1548	1.64 (1.63-1.64)	878	2.08 (2.07-2.08)	670	1.28 (1.28-1.28)
1970-1984	70-75	Alcohol use disorder (inpatient)	483	1.68 (1.68-1.68)	296	2.36 (2.36-2.36)	187	1.15 (1.15-1.16)
1985-1994	70-75	Alcohol use disorder (inpatient)	314	1.54 (1.54-1.54)	164	1.83 (1.83-1.84)	150	1.31 (1.31-1.31)
1995-2004	70-75	Alcohol use disorder (inpatient)	291	1.52 (1.52-1.52)	150	1.75 (1.75-1.76)	141	1.33 (1.33-1.33)
2005-2016	70-75	Alcohol use disorder (inpatient)	460	1.75 (1.75-1.75)	268	2.18 (2.18-2.19)	192	1.37 (1.37-1.37)
1970-2016	75-80	Alcohol use disorder (inpatient)	718	0.99 (0.99-0.99)	389	1.29 (1.29-1.29)	329	0.78 (0.78-0.78)
1970-1984	75-80	Alcohol use disorder (inpatient)	192	0.94 (0.94-0.94)	110	1.33 (1.32-1.33)	82	0.68 (0.67-0.68)
1985-1994	75-80	Alcohol use disorder (inpatient)	148	0.91 (0.91-0.91)	66	1.00 (1.00-1.01)	82	0.84 (0.84-0.85)
1995-2004	75-80	Alcohol use disorder (inpatient)	144	0.89 (0.89-0.90)	79	1.19 (1.19-1.19)	65	0.69 (0.69-0.69)
2005-2016	75-80	Alcohol use disorder (inpatient)	234	1.20 (1.20-1.20)	134	1.55 (1.55-1.55)	100	0.92 (0.92-0.92)
1970-2016	80-85	Alcohol use disorder (inpatient)	257	0.53 (0.53-0.53)	141	0.77 (0.77-0.77)	116	0.38 (0.38-0.38)
1970-1984	80-85	Alcohol use disorder (inpatient)	67	0.55 (0.55-0.55)	43	0.93 (0.93-0.93)	24	0.32 (0.32-0.32)
1985-1994	80-85	Alcohol use disorder (inpatient)	42	0.39 (0.38-0.39)	18	0.47 (0.47-0.47)	24	0.34 (0.34-0.34)
1995-2004	80-85	Alcohol use disorder (inpatient)	63	0.55 (0.55-0.55)	34	0.81 (0.81-0.81)	29	0.40 (0.40-0.40)
2005-2016	80-85	Alcohol use disorder (inpatient)	85	0.61 (0.61-0.61)	46	0.82 (0.82-0.82)	39	0.47 (0.46-0.47)
1970-2016	85-90	Alcohol use disorder (inpatient)	76	0.29 (0.29-0.29)	37	0.43 (0.43-0.43)	39	0.22 (0.22-0.22)
1970-1984	85-90	Alcohol use disorder (inpatient)	11	0.20 (0.20-0.20)	7-11	0.35 (0.35-0.35)	0-4	NA
1985-1994	85-90	Alcohol use disorder (inpatient)	16	0.29 (0.29-0.29)	9	0.54 (0.53-0.54)	7	0.18 (0.18-0.19)
1995-2004	85-90	Alcohol use disorder (inpatient)	20	0.31 (0.31-0.31)	7	0.35 (0.35-0.35)	13	0.29 (0.29-0.29)
2005-2016	85-90	Alcohol use disorder (inpatient)	29	0.34 (0.34-0.34)	14	0.48 (0.48-0.48)	15	0.27 (0.27-0.27)
1970-2016	90-95	Alcohol use disorder (inpatient)	11	0.12 (0.11-0.12)	0-4	NA	7-11	0.13 (0.13-0.13)
1970-1984	90-95	Alcohol use disorder (inpatient)	0-4	NA	0-4	NA	0-4	NA
1985-1994	90-95	Alcohol use disorder (inpatient)	0-4	NA	0-4	NA	0-4	NA
1995-2004	90-95	Alcohol use disorder (inpatient)	0-4	NA	0-4	NA	0-4	NA
2005-2016	90-95	Alcohol use disorder (inpatient)	0-4	NA	0-4	NA	0-4	NA
1970-2016	95-100	Alcohol use disorder (inpatient)	0-4	NA	0-4	NA	0-4	NA
1970-1984	95-100	Alcohol use disorder (inpatient)	0-4	NA	0-4	NA	0-4	NA
1985-1994	95-100	Alcohol use disorder (inpatient)	0-4	NA	0-4	NA	0-4	NA
1995-2004	95-100	Alcohol use disorder (inpatient)	0-4	NA	0-4	NA	0-4	NA
2005-2016	95-100	Alcohol use disorder (inpatient)	0-4	NA	0-4	NA	0-4	NA
1970-2016	10-15	Cannabis use disorder	315	0.20 (0.20-0.20)	142	0.18 (0.18-0.18)	173	0.22 (0.22-0.22)
1970-1984	10-15	Cannabis use disorder	35	0.06 (0.06-0.06)	15	0.05 (0.05-0.05)	20	0.07 (0.07-0.07)
1985-1994	10-15	Cannabis use disorder	0-4	NA	0-4	NA	0-4	NA
1995-2004	10-15	Cannabis use disorder	52	0.18 (0.18-0.18)	23	0.15 (0.15-0.15)	29	0.20 (0.20-0.20)
2005-2016	10-15	Cannabis use disorder	224	0.56 (0.56-0.56)	102	0.50 (0.50-0.50)	122	0.62 (0.62-0.62)
1970-2016	15-20	Cannabis use disorder	6256	3.89 (3.89-3.90)	4226	5.13 (5.12-5.13)	2030	2.59 (2.59-2.60)
1970-1984	15-20	Cannabis use disorder	946	1.66 (1.66-1.66)	641	2.19 (2.18-2.19)	305	1.10 (1.10-1.10)
1985-1994	15-20	Cannabis use disorder	288	0.82 (0.81-0.82)	227	1.25 (1.25-1.25)	61	0.35 (0.35-0.36)
1995-2004	15-20	Cannabis use disorder	970	3.41 (3.41-3.41)	699	4.80 (4.79-4.81)	271	1.95 (1.95-1.95)

			Both sexes		Males		Females	
Years	Age	Mental disorder	Cases	IR (95% CI)	Cases	IR (95% CI)	Cases	IR (95% CI)
2005-2016	15-20	Cannabis use disorder	4052	10.18 (10.17-10.19)	2659	13.01 (12.99-13.03)	1393	7.19 (7.18-7.20)
1970-2016	20-25	Cannabis use disorder	7698	4.78 (4.78-4.78)	5882	7.07 (7.07-7.08)	1816	2.33 (2.33-2.33)
1970-1984	20-25	Cannabis use disorder	1041	1.88 (1.88-1.88)	852	2.98 (2.97-2.98)	189	0.70 (0.70-0.71)
1985-1994	20-25	Cannabis use disorder	647	1.73 (1.73-1.74)	548	2.82 (2.82-2.83)	99	0.55 (0.55-0.55)
1995-2004	20-25	Cannabis use disorder	1457	4.71 (4.70-4.71)	1153	7.21 (7.20-7.22)	304	2.03 (2.03-2.03)
2005-2016	20-25	Cannabis use disorder	4553	12.19 (12.18-12.21)	3329	17.39 (17.37-17.42)	1224	6.73 (6.72-6.74)
1970-2016	25-30	Cannabis use disorder	4522	2.80 (2.80-2.80)	3599	4.33 (4.32-4.33)	923	1.18 (1.17-1.18)
1970-1984	25-30	Cannabis use disorder	437	0.77 (0.77-0.77)	360	1.24 (1.24-1.24)	77	0.28 (0.28-0.28)
1985-1994	25-30	Cannabis use disorder	450	1.22 (1.22-1.22)	360	1.88 (1.88-1.89)	90	0.51 (0.51-0.51)
1995-2004	25-30	Cannabis use disorder	1132	3.36 (3.36-3.37)	925	5.32 (5.31-5.33)	207	1.27 (1.27-1.27)
2005-2016	25-30	Cannabis use disorder	2503	7.25 (7.24-7.26)	1954	11.11 (11.09-11.12)	549	3.24 (3.24-3.25)
1970-2016	30-35	Cannabis use disorder	2916	1.82 (1.82-1.82)	2346	2.86 (2.86-2.86)	570	0.73 (0.73-0.73)
1970-1984	30-35	Cannabis use disorder	182	0.33 (0.33-0.34)	153	0.55 (0.55-0.55)	29	0.11 (0.11-0.11)
1985-1994	30-35	Cannabis use disorder	315	0.91 (0.91-0.91)	256	1.44 (1.44-1.44)	59	0.35 (0.35-0.35)
1995-2004	30-35	Cannabis use disorder	863	2.40 (2.39-2.40)	709	3.82 (3.82-3.83)	154	0.88 (0.88-0.88)
2005-2016	30-35	Cannabis use disorder	1556	4.38 (4.38-4.39)	1228	6.80 (6.79-6.81)	328	1.88 (1.88-1.88)
1970-2016	35-40	Cannabis use disorder	2012	1.27 (1.26-1.27)	1596	1.98 (1.97-1.98)	416	0.53 (0.53-0.53)
1970-1984	35-40	Cannabis use disorder	83	0.17 (0.17-0.17)	64	0.26 (0.26-0.26)	19	0.08 (0.08-0.08)
1985-1994	35-40	Cannabis use disorder	152	0.43 (0.43-0.44)	120	0.68 (0.67-0.68)	32	0.19 (0.19-0.19)
1995-2004	35-40	Cannabis use disorder	634	1.76 (1.76-1.77)	501	2.73 (2.72-2.73)	133	0.76 (0.76-0.76)
2005-2016	35-40	Cannabis use disorder	1143	2.97 (2.96-2.97)	911	4.65 (4.64-4.65)	232	1.22 (1.22-1.23)
1970-2016	40-45	Cannabis use disorder	1364	0.88 (0.88-0.88)	1029	1.31 (1.31-1.31)	335	0.43 (0.43-0.43)
1970-1984	40-45	Cannabis use disorder	32	0.07 (0.07-0.07)	27	0.12 (0.12-0.12)	5	0.02 (0.02-0.02)
1985-1994	40-45	Cannabis use disorder	76	0.21 (0.21-0.21)	55	0.30 (0.30-0.30)	21	0.11 (0.11-0.11)
1995-2004	40-45	Cannabis use disorder	409	1.21 (1.21-1.21)	313	1.82 (1.82-1.82)	96	0.58 (0.57-0.58)
2005-2016	40-45	Cannabis use disorder	847	2.04 (2.03-2.04)	634	3.00 (3.00-3.01)	213	1.04 (1.04-1.04)
1970-2016	45-50	Cannabis use disorder	959	0.63 (0.63-0.63)	696	0.92 (0.92-0.92)	263	0.35 (0.35-0.35)
1970-1984	45-50	Cannabis use disorder	15	0.04 (0.04-0.04)	10	0.05 (0.05-0.05)	5	0.02 (0.02-0.02)
1985-1994	45-50	Cannabis use disorder	38	0.11 (0.11-0.11)	30	0.18 (0.18-0.18)	8	0.05 (0.05-0.05)
1995-2004	45-50	Cannabis use disorder	256	0.75 (0.75-0.75)	199	1.17 (1.16-1.17)	57	0.34 (0.34-0.34)
2005-2016	45-50	Cannabis use disorder	650	1.56 (1.55-1.56)	457	2.17 (2.17-2.17)	193	0.93 (0.93-0.93)
1970-2016	50-55	Cannabis use disorder	485	0.33 (0.33-0.33)	344	0.48 (0.48-0.48)	141	0.19 (0.19-0.19)
1970-1984	50-55	Cannabis use disorder	8	0.02 (0.02-0.02)	0-4	NA	0-4	NA
1985-1994	50-55	Cannabis use disorder	14	0.05 (0.05-0.05)	5	0.04 (0.04-0.04)	9	0.06 (0.06-0.06)
1995-2004	50-55	Cannabis use disorder	104	0.29 (0.29-0.29)	77	0.44 (0.44-0.44)	27	0.15 (0.15-0.15)
2005-2016	50-55	Cannabis use disorder	359	0.91 (0.91-0.91)	258	1.30 (1.30-1.30)	101	0.51 (0.51-0.51)
1970-2016	55-60	Cannabis use disorder	235	0.17 (0.17-0.17)	175	0.26 (0.26-0.26)	60	0.09 (0.09-0.09)
1970-1984	55-60	Cannabis use disorder	0-4	NA	0-4	NA	0-4	NA
1985-1994	55-60	Cannabis use disorder	5	0.02 (0.02-0.02)	0-4	NA	0-4	NA
1995-2004	55-60	Cannabis use disorder	39	0.12 (0.12-0.12)	30	0.19 (0.19-0.19)	9	0.06 (0.06-0.06)

			Both sexes		Males		Females	
**Years**	**Age**	**Mental disorder**	**Cases**	**IR (95% CI)**	**Cases**	**IR (95% CI)**	**Cases**	**IR (95% CI)**
2005-2016	55-60	Cannabis use disorder	190	0.49 (0.49-0.49)	141	0.74 (0.74-0.74)	49	0.25 (0.25-0.25)
1970-2016	60-65	Cannabis use disorder	99	0.08 (0.08-0.08)	65	0.10 (0.10-0.10)	34	0.05 (0.05-0.05)
1970-1984	60-65	Cannabis use disorder	0-4	NA	0-4	NA	0-4	NA
1985-1994	60-65	Cannabis use disorder	0-4	NA	0-4	NA	0-4	NA
1995-2004	60-65	Cannabis use disorder	13	0.05 (0.05-0.05)	8	0.06 (0.06-0.06)	5	0.04 (0.04-0.04)
2005-2016	60-65	Cannabis use disorder	81	0.21 (0.21-0.21)	55	0.29 (0.29-0.29)	26	0.13 (0.13-0.13)
1970-2016	65-70	Cannabis use disorder	31	0.03 (0.03-0.03)	20	0.04 (0.04-0.04)	11	0.02 (0.02-0.02)
1970-1984	65-70	Cannabis use disorder	0-4	NA	0-4	NA	0-4	NA
1985-1994	65-70	Cannabis use disorder	0-4	NA	0-4	NA	0-4	NA
1995-2004	65-70	Cannabis use disorder	0-4	NA	0-4	NA	0-4	NA
2005-2016	65-70	Cannabis use disorder	25	0.07 (0.07-0.07)	17	0.10 (0.10-0.10)	8	0.04 (0.04-0.04)
1970-2016	70-75	Cannabis use disorder	10	0.01 (0.01-0.01)	5	0.01 (0.01-0.01)	5	0.01 (0.01-0.01)
1970-1984	70-75	Cannabis use disorder	0-4	NA	0-4	NA	0-4	NA
1985-1994	70-75	Cannabis use disorder	0-4	NA	0-4	NA	0-4	NA
1995-2004	70-75	Cannabis use disorder	0-4	NA	0-4	NA	0-4	NA
2005-2016	70-75	Cannabis use disorder	9	0.03 (0.03-0.03)	0-4	NA	5-9	0.04 (0.04-0.04)
1970-2016	75-80	Cannabis use disorder	0-4	NA	0-4	NA	0-4	NA
1970-1984	75-80	Cannabis use disorder	0-4	NA	0-4	NA	0-4	NA
1985-1994	75-80	Cannabis use disorder	0-4	NA	0-4	NA	0-4	NA
1995-2004	75-80	Cannabis use disorder	0-4	NA	0-4	NA	0-4	NA
2005-2016	75-80	Cannabis use disorder	0-4	NA	0-4	NA	0-4	NA
1970-2016	80-85	Cannabis use disorder	0-4	NA	0-4	NA	0-4	NA
1970-1984	80-85	Cannabis use disorder	0-4	NA	0-4	NA	0-4	NA
1985-1994	80-85	Cannabis use disorder	0-4	NA	0-4	NA	0-4	NA
1995-2004	80-85	Cannabis use disorder	0-4	NA	0-4	NA	0-4	NA
2005-2016	80-85	Cannabis use disorder	0-4	NA	0-4	NA	0-4	NA
1970-2016	85-90	Cannabis use disorder	0-4	NA	0-4	NA	0-4	NA
1970-1984	85-90	Cannabis use disorder	0-4	NA	0-4	NA	0-4	NA
1985-1994	85-90	Cannabis use disorder	0-4	NA	0-4	NA	0-4	NA
1995-2004	85-90	Cannabis use disorder	0-4	NA	0-4	NA	0-4	NA
2005-2016	85-90	Cannabis use disorder	0-4	NA	0-4	NA	0-4	NA
1970-2016	90-95	Cannabis use disorder	0-4	NA	0-4	NA	0-4	NA
1970-1984	90-95	Cannabis use disorder	0-4	NA	0-4	NA	0-4	NA
1985-1994	90-95	Cannabis use disorder	0-4	NA	0-4	NA	0-4	NA
1995-2004	90-95	Cannabis use disorder	0-4	NA	0-4	NA	0-4	NA
2005-2016	90-95	Cannabis use disorder	0-4	NA	0-4	NA	0-4	NA
1970-2016	95-100	Cannabis use disorder	0-4	NA	0-4	NA	0-4	NA
1970-1984	95-100	Cannabis use disorder	0-4	NA	0-4	NA	0-4	NA
1985-1994	95-100	Cannabis use disorder	0-4	NA	0-4	NA	0-4	NA
1995-2004	95-100	Cannabis use disorder	0-4	NA	0-4	NA	0-4	NA

			Both sexes		Males		Females	
Years	Age	Mental disorder	Cases	IR (95% CI)	Cases	IR (95% CI)	Cases	IR (95% CI)
2005-2016	95-100	Cannabis use disorder	0-4	NA	0-4	NA	0-4	NA
1970-2016	10-15	Cannabis use disorder (inpatient)	91	0.06 (0.06-0.06)	36	0.04 (0.04-0.04)	55	0.07 (0.07-0.07)
1970-1984	10-15	Cannabis use disorder (inpatient)	35	0.06 (0.06-0.06)	15	0.05 (0.05-0.05)	20	0.07 (0.07-0.07)
1985-1994	10-15	Cannabis use disorder (inpatient)	0-4	NA	0-4	NA	0-4	NA
1995-2004	10-15	Cannabis use disorder (inpatient)	13	0.04 (0.04-0.04)	0-4	NA	9-13	0.06 (0.06-0.06)
2005-2016	10-15	Cannabis use disorder (inpatient)	39	0.10 (0.10-0.10)	15	0.07 (0.07-0.07)	24	0.12 (0.12-0.12)
1970-2016	15-20	Cannabis use disorder (inpatient)	2928	1.82 (1.82-1.82)	2012	2.44 (2.44-2.44)	916	1.17 (1.17-1.17)
1970-1984	15-20	Cannabis use disorder (inpatient)	946	1.66 (1.66-1.66)	641	2.19 (2.18-2.19)	305	1.10 (1.10-1.10)
1985-1994	15-20	Cannabis use disorder (inpatient)	282	0.80 (0.80-0.80)	222	1.23 (1.22-1.23)	60	0.35 (0.35-0.35)
1995-2004	15-20	Cannabis use disorder (inpatient)	472	1.66 (1.66-1.66)	346	2.38 (2.37-2.38)	126	0.91 (0.91-0.91)
2005-2016	15-20	Cannabis use disorder (inpatient)	1228	3.08 (3.08-3.08)	803	3.92 (3.92-3.93)	425	2.19 (2.19-2.19)
1970-2016	20-25	Cannabis use disorder (inpatient)	4595	2.85 (2.85-2.85)	3592	4.31 (4.31-4.32)	1003	1.29 (1.29-1.29)
1970-1984	20-25	Cannabis use disorder (inpatient)	1041	1.88 (1.88-1.88)	852	2.98 (2.97-2.98)	189	0.70 (0.70-0.71)
1985-1994	20-25	Cannabis use disorder (inpatient)	642	1.72 (1.72-1.72)	545	2.81 (2.80-2.81)	97	0.54 (0.54-0.54)
1995-2004	20-25	Cannabis use disorder (inpatient)	775	2.50 (2.50-2.50)	629	3.93 (3.92-3.94)	146	0.97 (0.97-0.98)
2005-2016	20-25	Cannabis use disorder (inpatient)	2137	5.70 (5.70-5.71)	1566	8.14 (8.13-8.15)	571	3.13 (3.13-3.13)
1970-2016	25-30	Cannabis use disorder (inpatient)	2852	1.76 (1.76-1.76)	2284	2.74 (2.74-2.74)	568	0.72 (0.72-0.72)
1970-1984	25-30	Cannabis use disorder (inpatient)	437	0.77 (0.77-0.77)	360	1.24 (1.24-1.24)	77	0.28 (0.28-0.28)
1985-1994	25-30	Cannabis use disorder (inpatient)	446	1.21 (1.21-1.21)	357	1.87 (1.87-1.87)	89	0.50 (0.50-0.50)
1995-2004	25-30	Cannabis use disorder (inpatient)	666	1.98 (1.98-1.98)	547	3.14 (3.14-3.15)	119	0.73 (0.73-0.73)
2005-2016	25-30	Cannabis use disorder (inpatient)	1303	3.76 (3.76-3.76)	1020	5.77 (5.76-5.77)	283	1.67 (1.67-1.67)
1970-2016	30-35	Cannabis use disorder (inpatient)	1861	1.16 (1.16-1.16)	1486	1.81 (1.81-1.81)	375	0.48 (0.48-0.48)
1970-1984	30-35	Cannabis use disorder (inpatient)	182	0.33 (0.33-0.34)	153	0.55 (0.55-0.55)	29	0.11 (0.11-0.11)
1985-1994	30-35	Cannabis use disorder (inpatient)	307	0.89 (0.89-0.89)	250	1.41 (1.40-1.41)	57	0.34 (0.34-0.34)
1995-2004	30-35	Cannabis use disorder (inpatient)	525	1.46 (1.46-1.46)	422	2.27 (2.27-2.28)	103	0.59 (0.59-0.59)
2005-2016	30-35	Cannabis use disorder (inpatient)	847	2.38 (2.38-2.38)	661	3.64 (3.64-3.65)	186	1.06 (1.06-1.07)
1970-2016	35-40	Cannabis use disorder (inpatient)	1296	0.81 (0.81-0.82)	1030	1.27 (1.27-1.27)	266	0.34 (0.34-0.34)
1970-1984	35-40	Cannabis use disorder (inpatient)	83	0.17 (0.17-0.17)	64	0.26 (0.26-0.26)	19	0.08 (0.08-0.08)
1985-1994	35-40	Cannabis use disorder (inpatient)	146	0.42 (0.42-0.42)	115	0.65 (0.65-0.65)	31	0.18 (0.18-0.18)
1995-2004	35-40	Cannabis use disorder (inpatient)	398	1.11 (1.11-1.11)	319	1.73 (1.73-1.74)	79	0.45 (0.45-0.45)
2005-2016	35-40	Cannabis use disorder (inpatient)	669	1.73 (1.73-1.73)	532	2.70 (2.70-2.71)	137	0.72 (0.72-0.72)
1970-2016	40-45	Cannabis use disorder (inpatient)	924	0.59 (0.59-0.59)	696	0.88 (0.88-0.89)	228	0.30 (0.30-0.30)
1970-1984	40-45	Cannabis use disorder (inpatient)	32	0.07 (0.07-0.07)	27	0.12 (0.12-0.12)	5	0.02 (0.02-0.02)
1985-1994	40-45	Cannabis use disorder (inpatient)	74	0.20 (0.20-0.20)	54	0.29 (0.29-0.29)	20	0.11 (0.11-0.11)
1995-2004	40-45	Cannabis use disorder (inpatient)	271	0.80 (0.80-0.80)	213	1.24 (1.24-1.24)	58	0.35 (0.35-0.35)
2005-2016	40-45	Cannabis use disorder (inpatient)	547	1.31 (1.31-1.31)	402	1.90 (1.90-1.90)	145	0.71 (0.71-0.71)
1970-2016	45-50	Cannabis use disorder (inpatient)	637	0.42 (0.42-0.42)	474	0.62 (0.62-0.62)	163	0.22 (0.22-0.22)
1970-1984	45-50	Cannabis use disorder (inpatient)	15	0.04 (0.04-0.04)	10	0.05 (0.05-0.05)	5	0.02 (0.02-0.02)
1985-1994	45-50	Cannabis use disorder (inpatient)	36	0.11 (0.11-0.11)	28	0.16 (0.16-0.17)	8	0.05 (0.05-0.05)
1995-2004	45-50	Cannabis use disorder (inpatient)	165	0.49 (0.49-0.49)	126	0.74 (0.74-0.74)	39	0.23 (0.23-0.23)

			Both sexes		Males		Females	
**Years**	**Age**	**Mental disorder**	**Cases**	**IR (95% CI)**	**Cases**	**IR (95% CI)**	**Cases**	**IR (95% CI)**
2005-2016	45-50	Cannabis use disorder (inpatient)	421	1.01 (1.01-1.01)	310	1.47 (1.47-1.47)	111	0.54 (0.54-0.54)
1970-2016	50-55	Cannabis use disorder (inpatient)	319	0.22 (0.22-0.22)	220	0.30 (0.30-0.30)	99	0.14 (0.14-0.14)
1970-1984	50-55	Cannabis use disorder (inpatient)	8	0.02 (0.02-0.02)	0-4	NA	0-4	NA
1985-1994	50-55	Cannabis use disorder (inpatient)	14	0.05 (0.05-0.05)	5	0.04 (0.04-0.04)	9	0.06 (0.06-0.06)
1995-2004	50-55	Cannabis use disorder (inpatient)	66	0.19 (0.19-0.19)	48	0.27 (0.27-0.27)	18	0.10 (0.10-0.10)
2005-2016	50-55	Cannabis use disorder (inpatient)	231	0.58 (0.58-0.58)	163	0.82 (0.82-0.82)	68	0.34 (0.34-0.34)
1970-2016	55-60	Cannabis use disorder (inpatient)	163	0.12 (0.12-0.12)	123	0.18 (0.18-0.18)	40	0.06 (0.06-0.06)
1970-1984	55-60	Cannabis use disorder (inpatient)	0-4	NA	0-4	NA	0-4	NA
1985-1994	55-60	Cannabis use disorder (inpatient)	5	0.02 (0.02-0.02)	0-4	NA	0-4	NA
1995-2004	55-60	Cannabis use disorder (inpatient)	30	0.09 (0.09-0.09)	23	0.15 (0.15-0.15)	7	0.04 (0.04-0.04)
2005-2016	55-60	Cannabis use disorder (inpatient)	127	0.33 (0.33-0.33)	96	0.50 (0.50-0.50)	31	0.16 (0.16-0.16)
1970-2016	60-65	Cannabis use disorder (inpatient)	63	0.05 (0.05-0.05)	46	0.07 (0.07-0.07)	17	0.03 (0.03-0.03)
1970-1984	60-65	Cannabis use disorder (inpatient)	0-4	NA	0-4	NA	0-4	NA
1985-1994	60-65	Cannabis use disorder (inpatient)	0-4	NA	0-4	NA	0-4	NA
1995-2004	60-65	Cannabis use disorder (inpatient)	6	0.02 (0.02-0.02)	0-4	NA	0-4	NA
2005-2016	60-65	Cannabis use disorder (inpatient)	52	0.13 (0.13-0.13)	40	0.21 (0.21-0.21)	12	0.06 (0.06-0.06)
1970-2016	65-70	Cannabis use disorder (inpatient)	16	0.01 (0.01-0.01)	11	0.02 (0.02-0.02)	5	0.01 (0.01-0.01)
1970-1984	65-70	Cannabis use disorder (inpatient)	0-4	NA	0-4	NA	0-4	NA
1985-1994	65-70	Cannabis use disorder (inpatient)	0-4	NA	0-4	NA	0-4	NA
1995-2004	65-70	Cannabis use disorder (inpatient)	0-4	NA	0-4	NA	0-4	NA
2005-2016	65-70	Cannabis use disorder (inpatient)	12	0.03 (0.03-0.03)	8-12	0.05 (0.05-0.05)	0-4	NA
1970-2016	70-75	Cannabis use disorder (inpatient)	6	0.01 (0.01-0.01)	0-4	NA	0-4	NA
1970-1984	70-75	Cannabis use disorder (inpatient)	0-4	NA	0-4	NA	0-4	NA
1985-1994	70-75	Cannabis use disorder (inpatient)	0-4	NA	0-4	NA	0-4	NA
1995-2004	70-75	Cannabis use disorder (inpatient)	0-4	NA	0-4	NA	0-4	NA
2005-2016	70-75	Cannabis use disorder (inpatient)	5	0.02 (0.02-0.02)	0-4	NA	0-4	NA
1970-2016	75-80	Cannabis use disorder (inpatient)	0-4	NA	0-4	NA	0-4	NA
1970-1984	75-80	Cannabis use disorder (inpatient)	0-4	NA	0-4	NA	0-4	NA
1985-1994	75-80	Cannabis use disorder (inpatient)	0-4	NA	0-4	NA	0-4	NA
1995-2004	75-80	Cannabis use disorder (inpatient)	0-4	NA	0-4	NA	0-4	NA
2005-2016	75-80	Cannabis use disorder (inpatient)	0-4	NA	0-4	NA	0-4	NA
1970-2016	80-85	Cannabis use disorder (inpatient)	0-4	NA	0-4	NA	0-4	NA
1970-1984	80-85	Cannabis use disorder (inpatient)	0-4	NA	0-4	NA	0-4	NA
1985-1994	80-85	Cannabis use disorder (inpatient)	0-4	NA	0-4	NA	0-4	NA
1995-2004	80-85	Cannabis use disorder (inpatient)	0-4	NA	0-4	NA	0-4	NA
2005-2016	80-85	Cannabis use disorder (inpatient)	0-4	NA	0-4	NA	0-4	NA
1970-2016	85-90	Cannabis use disorder (inpatient)	0-4	NA	0-4	NA	0-4	NA
1970-1984	85-90	Cannabis use disorder (inpatient)	0-4	NA	0-4	NA	0-4	NA
1985-1994	85-90	Cannabis use disorder (inpatient)	0-4	NA	0-4	NA	0-4	NA
1995-2004	85-90	Cannabis use disorder (inpatient)	0-4	NA	0-4	NA	0-4	NA

			Both sexes		Males		Females	
Years	Age	Mental disorder	Cases	IR (95% CI)	Cases	IR (95% CI)	Cases	IR (95% CI)
2005-2016	85-90	Cannabis use disorder (inpatient)	0-4	NA	0-4	NA	0-4	NA
1970-2016	90-95	Cannabis use disorder (inpatient)	0-4	NA	0-4	NA	0-4	NA
1970-1984	90-95	Cannabis use disorder (inpatient)	0-4	NA	0-4	NA	0-4	NA
1985-1994	90-95	Cannabis use disorder (inpatient)	0-4	NA	0-4	NA	0-4	NA
1995-2004	90-95	Cannabis use disorder (inpatient)	0-4	NA	0-4	NA	0-4	NA
2005-2016	90-95	Cannabis use disorder (inpatient)	0-4	NA	0-4	NA	0-4	NA
1970-2016	95-100	Cannabis use disorder (inpatient)	0-4	NA	0-4	NA	0-4	NA
1970-1984	95-100	Cannabis use disorder (inpatient)	0-4	NA	0-4	NA	0-4	NA
1985-1994	95-100	Cannabis use disorder (inpatient)	0-4	NA	0-4	NA	0-4	NA
1995-2004	95-100	Cannabis use disorder (inpatient)	0-4	NA	0-4	NA	0-4	NA
2005-2016	95-100	Cannabis use disorder (inpatient)	0-4	NA	0-4	NA	0-4	NA
1970-2016	10-15	Schizophrenia spectrum disorder	2,166	1.37 (1.37-1.37)	979	1.21 (1.21-1.21)	1,187	1.54 (1.54-1.54)
1970-1984	10-15	Schizophrenia spectrum disorder	177	0.31 (0.31-0.31)	107	0.37 (0.37-0.37)	70	0.25 (0.25-0.25)
1985-1994	10-15	Schizophrenia spectrum disorder	135	0.43 (0.43-0.43)	80	0.50 (0.50-0.50)	55	0.36 (0.36-0.36)
1995-2004	10-15	Schizophrenia spectrum disorder	434	1.47 (1.47-1.47)	230	1.52 (1.51-1.52)	204	1.42 (1.41-1.42)
2005-2016	10-15	Schizophrenia spectrum disorder	1,420	3.54 (3.54-3.54)	562	2.73 (2.73-2.74)	858	4.39 (4.38-4.39)
1970-2016	15-20	Schizophrenia spectrum disorder	11,471	7.15 (7.14-7.15)	5,957	7.23 (7.23-7.24)	5,514	7.06 (7.05-7.06)
1970-1984	15-20	Schizophrenia spectrum disorder	2,204	3.86 (3.86-3.87)	1,317	4.50 (4.49-4.50)	887	3.20 (3.19-3.20)
1985-1994	15-20	Schizophrenia spectrum disorder	1,348	3.82 (3.82-3.82)	731	4.04 (4.03-4.04)	617	3.59 (3.58-3.60)
1995-2004	15-20	Schizophrenia spectrum disorder	2,060	7.25 (7.24-7.26)	1,124	7.73 (7.71-7.74)	936	6.75 (6.74-6.76)
2005-2016	15-20	Schizophrenia spectrum disorder	5,859	14.75 (14.74-14.77)	2,785	13.65 (13.63-13.66)	3,074	15.92 (15.90-15.94)
1970-2016	20-25	Schizophrenia spectrum disorder	16,793	10.46 (10.45-10.46)	10,239	12.34 (12.34-12.35)	6,554	8.44 (8.44-8.45)
1970-1984	20-25	Schizophrenia spectrum disorder	4,114	7.43 (7.43-7.44)	2,643	9.26 (9.24-9.27)	1,471	5.49 (5.49-5.50)
1985-1994	20-25	Schizophrenia spectrum disorder	2,668	7.17 (7.17-7.18)	1,595	8.24 (8.23-8.26)	1,073	6.01 (6.01-6.02)
1995-2004	20-25	Schizophrenia spectrum disorder	3,297	10.68 (10.67-10.69)	2,045	12.83 (12.81-12.85)	1,252	8.39 (8.38-8.41)
2005-2016	20-25	Schizophrenia spectrum disorder	6,714	18.06 (18.04-18.08)	3,956	20.72 (20.69-20.75)	2,758	15.25 (15.23-15.27)
1970-2016	25-30	Schizophrenia spectrum disorder	13,604	8.46 (8.45-8.46)	8,126	9.82 (9.81-9.83)	5,478	7.01 (7.01-7.02)
1970-1984	25-30	Schizophrenia spectrum disorder	4,027	7.13 (7.13-7.14)	2,251	7.77 (7.76-7.78)	1,776	6.47 (6.46-6.47)
1985-1994	25-30	Schizophrenia spectrum disorder	2,719	7.42 (7.41-7.42)	1,505	7.93 (7.92-7.94)	1,214	6.87 (6.86-6.88)
1995-2004	25-30	Schizophrenia spectrum disorder	2,886	8.62 (8.61-8.63)	1,880	10.88 (10.86-10.89)	1,006	6.22 (6.21-6.23)
2005-2016	25-30	Schizophrenia spectrum disorder	3,972	11.58 (11.57-11.59)	2,490	14.23 (14.20-14.25)	1,482	8.83 (8.81-8.84)
1970-2016	30-35	Schizophrenia spectrum disorder	11,519	7.23 (7.23-7.23)	6,442	7.90 (7.90-7.91)	5,077	6.52 (6.52-6.53)
1970-1984	30-35	Schizophrenia spectrum disorder	3,670	6.79 (6.78-6.80)	1,819	6.61 (6.60-6.62)	1,851	6.98 (6.97-6.99)
1985-1994	30-35	Schizophrenia spectrum disorder	2,294	6.68 (6.67-6.69)	1,197	6.78 (6.77-6.79)	1,097	6.57 (6.56-6.58)
1995-2004	30-35	Schizophrenia spectrum disorder	2,695	7.54 (7.54-7.55)	1,645	8.95 (8.93-8.96)	1,050	6.06 (6.05-6.07)
2005-2016	30-35	Schizophrenia spectrum disorder	2,860	8.12 (8.11-8.13)	1,781	9.93 (9.92-9.95)	1,079	6.24 (6.23-6.25)
1970-2016	35-40	Schizophrenia spectrum disorder	10,184	6.46 (6.46-6.46)	5,313	6.63 (6.63-6.64)	4,871	6.28 (6.28-6.29)
1970-1984	35-40	Schizophrenia spectrum disorder	3,267	6.64 (6.63-6.64)	1,464	5.88 (5.87-5.89)	1,803	7.41 (7.40-7.42)
1985-1994	35-40	Schizophrenia spectrum disorder	2,125	6.14 (6.13-6.14)	1,015	5.77 (5.76-5.78)	1,110	6.51 (6.50-6.52)
1995-2004	35-40	Schizophrenia spectrum disorder	2,347	6.60 (6.59-6.60)	1,377	7.57 (7.56-7.58)	970	5.58 (5.57-5.58)

			Both sexes		Males		Females	
**Years**	**Age**	**Mental disorder**	**Cases**	**IR (95% CI)**	**Cases**	**IR (95% CI)**	**Cases**	**IR (95% CI)**
2005-2016	35-40	Schizophrenia spectrum disorder	2,445	6.40 (6.40-6.41)	1,457	7.50 (7.49-7.51)	988	5.27 (5.26-5.27)
1970-2016	40-45	Schizophrenia spectrum disorder	9,014	5.84 (5.84-5.85)	4,325	5.55 (5.55-5.56)	4,689	6.14 (6.14-6.14)
1970-1984	40-45	Schizophrenia spectrum disorder	3,099	7.18 (7.17-7.18)	1,286	5.95 (5.94-5.96)	1,813	8.41 (8.40-8.42)
1985-1994	40-45	Schizophrenia spectrum disorder	1,806	4.95 (4.94-4.95)	742	4.03 (4.03-4.04)	1,064	5.88 (5.87-5.89)
1995-2004	40-45	Schizophrenia spectrum disorder	1,931	5.77 (5.77-5.78)	1,041	6.13 (6.12-6.14)	890	5.41 (5.40-5.41)
2005-2016	40-45	Schizophrenia spectrum disorder	2,178	5.30 (5.29-5.30)	1,256	6.02 (6.01-6.03)	922	4.55 (4.55-4.56)
1970-2016	45-50	Schizophrenia spectrum disorder	8,135	5.42 (5.42-5.43)	3,532	4.70 (4.69-4.70)	4,603	6.15 (6.15-6.16)
1970-1984	45-50	Schizophrenia spectrum disorder	3,103	7.43 (7.42-7.43)	1,136	5.48 (5.48-5.49)	1,967	9.33 (9.32-9.35)
1985-1994	45-50	Schizophrenia spectrum disorder	1,523	4.55 (4.54-4.55)	581	3.46 (3.45-3.46)	942	5.65 (5.64-5.66)
1995-2004	45-50	Schizophrenia spectrum disorder	1,631	4.87 (4.86-4.87)	828	4.91 (4.91-4.92)	803	4.82 (4.81-4.83)
2005-2016	45-50	Schizophrenia spectrum disorder	1,878	4.55 (4.55-4.56)	987	4.74 (4.74-4.75)	891	4.36 (4.36-4.37)
1970-2016	50-55	Schizophrenia spectrum disorder	7,036	4.90 (4.89-4.90)	2,721	3.81 (3.81-3.81)	4,315	5.98 (5.97-5.98)
1970-1984	50-55	Schizophrenia spectrum disorder	2,979	7.09 (7.08-7.09)	1,008	4.87 (4.86-4.88)	1,971	9.23 (9.22-9.25)
1985-1994	50-55	Schizophrenia spectrum disorder	1,181	4.26 (4.26-4.27)	391	2.84 (2.84-2.85)	790	5.66 (5.66-5.67)
1995-2004	50-55	Schizophrenia spectrum disorder	1,425	4.09 (4.08-4.09)	604	3.47 (3.46-3.47)	821	4.70 (4.70-4.71)
2005-2016	50-55	Schizophrenia spectrum disorder	1,451	3.72 (3.71-3.72)	718	3.66 (3.66-3.67)	733	3.77 (3.76-3.77)
1970-2016	55-60	Schizophrenia spectrum disorder	5,596	4.12 (4.11-4.12)	2,078	3.10 (3.10-3.11)	3,518	5.10 (5.09-5.10)
1970-1984	55-60	Schizophrenia spectrum disorder	2,509	6.04 (6.03-6.04)	829	4.09 (4.09-4.10)	1,680	7.89 (7.88-7.90)
1985-1994	55-60	Schizophrenia spectrum disorder	978	3.90 (3.89-3.90)	330	2.69 (2.68-2.69)	648	5.05 (5.04-5.06)
1995-2004	55-60	Schizophrenia spectrum disorder	982	3.13 (3.12-3.13)	389	2.50 (2.49-2.50)	593	3.74 (3.74-3.75)
2005-2016	55-60	Schizophrenia spectrum disorder	1,127	2.98 (2.97-2.98)	530	2.82 (2.81-2.82)	597	3.13 (3.13-3.14)
1970-2016	60-65	Schizophrenia spectrum disorder	4,583	3.60 (3.60-3.61)	1,656	2.69 (2.69-2.69)	2,927	4.46 (4.46-4.47)
1970-1984	60-65	Schizophrenia spectrum disorder	2,045	5.17 (5.16-5.17)	661	3.48 (3.48-3.49)	1,384	6.71 (6.71-6.72)
1985-1994	60-65	Schizophrenia spectrum disorder	901	3.70 (3.69-3.70)	292	2.51 (2.50-2.51)	609	4.79 (4.79-4.80)
1995-2004	60-65	Schizophrenia spectrum disorder	781	3.10 (3.10-3.11)	328	2.68 (2.67-2.68)	453	3.50 (3.50-3.51)
2005-2016	60-65	Schizophrenia spectrum disorder	856	2.25 (2.25-2.25)	375	2.01 (2.00-2.01)	481	2.49 (2.48-2.49)
1970-2016	65-70	Schizophrenia spectrum disorder	4,017	3.52 (3.52-3.52)	1,343	2.50 (2.50-2.50)	2,674	4.43 (4.43-4.43)
1970-1984	65-70	Schizophrenia spectrum disorder	1,676	4.78 (4.77-4.78)	524	3.23 (3.22-3.23)	1,152	6.11 (6.10-6.12)
1985-1994	65-70	Schizophrenia spectrum disorder	888	3.81 (3.80-3.81)	269	2.49 (2.49-2.50)	619	4.94 (4.93-4.95)
1995-2004	65-70	Schizophrenia spectrum disorder	702	3.25 (3.25-3.26)	237	2.33 (2.32-2.33)	465	4.08 (4.08-4.09)
2005-2016	65-70	Schizophrenia spectrum disorder	751	2.20 (2.20-2.21)	313	1.90 (1.90-1.90)	438	2.49 (2.48-2.49)
1970-2016	70-75	Schizophrenia spectrum disorder	3,643	3.86 (3.86-3.86)	1,116	2.63 (2.63-2.63)	2,527	4.86 (4.86-4.87)
1970-1984	70-75	Schizophrenia spectrum disorder	1,400	4.89 (4.88-4.90)	412	3.29 (3.29-3.30)	988	6.13 (6.12-6.14)
1985-1994	70-75	Schizophrenia spectrum disorder	846	4.16 (4.16-4.17)	237	2.65 (2.64-2.65)	609	5.36 (5.35-5.37)
1995-2004	70-75	Schizophrenia spectrum disorder	756	3.95 (3.95-3.96)	231	2.68 (2.68-2.69)	525	4.99 (4.98-5.00)
2005-2016	70-75	Schizophrenia spectrum disorder	641	2.44 (2.43-2.44)	236	1.91 (1.91-1.91)	405	2.90 (2.89-2.90)
1970-2016	75-80	Schizophrenia spectrum disorder	3,501	4.86 (4.86-4.86)	999	3.31 (3.31-3.32)	2,502	5.97 (5.97-5.98)
1970-1984	75-80	Schizophrenia spectrum disorder	1,096	5.39 (5.38-5.40)	291	3.52 (3.51-3.53)	805	6.67 (6.66-6.69)
1985-1994	75-80	Schizophrenia spectrum disorder	822	5.08 (5.07-5.08)	219	3.34 (3.33-3.34)	603	6.27 (6.25-6.28)
1995-2004	75-80	Schizophrenia spectrum disorder	945	5.90 (5.89-5.91)	279	4.19 (4.18-4.20)	666	7.11 (7.09-7.12)

			Both sexes		Males		Females	
Years	Age	Mental disorder	Cases	IR (95% CI)	Cases	IR (95% CI)	Cases	IR (95% CI)
2005-2016	75-80	Schizophrenia spectrum disorder	638	3.27 (3.27-3.28)	210	2.42 (2.42-2.43)	428	3.95 (3.95-3.96)
1970-2016	80-85	Schizophrenia spectrum disorder	2,953	6.12 (6.12-6.13)	716	3.92 (3.91-3.93)	2,237	7.47 (7.46-7.48)
1970-1984	80-85	Schizophrenia spectrum disorder	740	6.10 (6.09-6.11)	175	3.79 (3.78-3.80)	565	7.53 (7.51-7.54)
1985-1994	80-85	Schizophrenia spectrum disorder	651	6.02 (6.00-6.03)	142	3.69 (3.68-3.70)	509	7.30 (7.28-7.32)
1995-2004	80-85	Schizophrenia spectrum disorder	900	7.93 (7.92-7.95)	229	5.48 (5.46-5.50)	671	9.36 (9.34-9.38)
2005-2016	80-85	Schizophrenia spectrum disorder	662	4.75 (4.74-4.76)	170	3.02 (3.01-3.03)	492	5.92 (5.91-5.93)
1970-2016	85-90	Schizophrenia spectrum disorder	1,970	7.62 (7.61-7.63)	412	4.81 (4.80-4.82)	1,558	9.02 (9.00-9.03)
1970-1984	85-90	Schizophrenia spectrum disorder	286	5.20 (5.19-5.22)	63	3.17 (3.16-3.19)	223	6.35 (6.33-6.37)
1985-1994	85-90	Schizophrenia spectrum disorder	364	6.70 (6.69-6.72)	69	4.13 (4.11-4.15)	295	7.85 (7.82-7.87)
1995-2004	85-90	Schizophrenia spectrum disorder	719	11.19 (11.16-11.21)	142	7.09 (7.06-7.12)	577	13.04 (13.00-13.08)
2005-2016	85-90	Schizophrenia spectrum disorder	601	7.08 (7.06-7.09)	138	4.75 (4.73-4.76)	463	8.29 (8.27-8.32)
1970-2016	90-95	Schizophrenia spectrum disorder	758	8.01 (7.99-8.02)	160	6.07 (6.05-6.10)	598	8.75 (8.73-8.77)
1970-1984	90-95	Schizophrenia spectrum disorder	64	4.02 (4.00-4.04)	19	3.46 (3.43-3.49)	45	4.32 (4.29-4.35)
1985-1994	90-95	Schizophrenia spectrum disorder	91	4.93 (4.91-4.95)	25	4.95 (4.90-4.99)	66	4.92 (4.90-4.95)
1995-2004	90-95	Schizophrenia spectrum disorder	303	12.38 (12.34-12.43)	46	7.55 (7.49-7.61)	257	13.99 (13.92-14.05)
2005-2016	90-95	Schizophrenia spectrum disorder	300	8.38 (8.35-8.40)	70	7.22 (7.17-7.26)	230	8.81 (8.77-8.84)
1970-2016	95-100	Schizophrenia spectrum disorder	179	9.07 (9.03-9.11)	30	6.76 (6.70-6.83)	149	9.74 (9.69-9.79)
1970-1984	95-100	Schizophrenia spectrum disorder	6	2.38 (2.35-2.41)	0-4	NA	0-4	NA
1985-1994	95-100	Schizophrenia spectrum disorder	14	4.01 (3.97-4.06)	0-4	NA	10-14	4.20 (4.15-4.25)
1995-2004	95-100	Schizophrenia spectrum disorder	62	12.39 (12.28-12.50)	9	9.09 (8.91-9.27)	53	13.21 (13.08-13.34)
2005-2016	95-100	Schizophrenia spectrum disorder	97	11.12 (11.04-11.19)	16	9.03 (8.90-9.17)	81	11.65 (11.56-11.74)
1970-2016	10-15	Schizophrenia spectrum disorder (inpatient)	1,030	0.65 (0.65-0.65)	482	0.60 (0.59-0.60)	548	0.71 (0.71-0.71)
1970-1984	10-15	Schizophrenia spectrum disorder (inpatient)	177	0.31 (0.31-0.31)	107	0.37 (0.37-0.37)	70	0.25 (0.25-0.25)
1985-1994	10-15	Schizophrenia spectrum disorder (inpatient)	134	0.42 (0.42-0.43)	80	0.50 (0.50-0.50)	54	0.35 (0.35-0.35)
1995-2004	10-15	Schizophrenia spectrum disorder (inpatient)	217	0.73 (0.73-0.73)	112	0.74 (0.74-0.74)	105	0.73 (0.73-0.73)
2005-2016	10-15	Schizophrenia spectrum disorder (inpatient)	502	1.25 (1.25-1.25)	183	0.89 (0.89-0.89)	319	1.63 (1.63-1.63)
1970-2016	15-20	Schizophrenia spectrum disorder (inpatient)	7,714	4.80 (4.80-4.81)	4,071	4.94 (4.94-4.94)	3,643	4.66 (4.66-4.66)
1970-1984	15-20	Schizophrenia spectrum disorder (inpatient)	2,204	3.86 (3.86-3.87)	1,317	4.50 (4.49-4.50)	887	3.20 (3.19-3.20)
1985-1994	15-20	Schizophrenia spectrum disorder (inpatient)	1,327	3.76 (3.76-3.76)	714	3.94 (3.94-3.95)	613	3.57 (3.56-3.57)
1995-2004	15-20	Schizophrenia spectrum disorder (inpatient)	1,340	4.71 (4.71-4.72)	719	4.94 (4.93-4.95)	621	4.47 (4.47-4.48)
2005-2016	15-20	Schizophrenia spectrum disorder (inpatient)	2,843	7.14 (7.13-7.15)	1,321	6.46 (6.45-6.47)	1,522	7.86 (7.85-7.87)
1970-2016	20-25	Schizophrenia spectrum disorder (inpatient)	12,878	8.01 (8.01-8.01)	7,866	9.47 (9.46-9.48)	5,012	6.45 (6.44-6.45)
1970-1984	20-25	Schizophrenia spectrum disorder (inpatient)	4,114	7.43 (7.43-7.44)	2,643	9.26 (9.24-9.27)	1,471	5.49 (5.49-5.50)
1985-1994	20-25	Schizophrenia spectrum disorder (inpatient)	2,618	7.04 (7.03-7.05)	1,565	8.09 (8.08-8.10)	1,053	5.90 (5.89-5.91)
1995-2004	20-25	Schizophrenia spectrum disorder (inpatient)	2,388	7.73 (7.72-7.74)	1,481	9.28 (9.26-9.29)	907	6.07 (6.06-6.08)
2005-2016	20-25	Schizophrenia spectrum disorder (inpatient)	3,758	10.06 (10.05-10.07)	2,177	11.34 (11.33-11.36)	1,581	8.70 (8.69-8.71)
1970-2016	25-30	Schizophrenia spectrum disorder (inpatient)	11,156	6.93 (6.92-6.93)	6,552	7.90 (7.90-7.91)	4,604	5.89 (5.88-5.89)
1970-1984	25-30	Schizophrenia spectrum disorder (inpatient)	4,027	7.13 (7.13-7.14)	2,251	7.77 (7.76-7.78)	1,776	6.47 (6.46-6.47)
1985-1994	25-30	Schizophrenia spectrum disorder (inpatient)	2,659	7.25 (7.25-7.26)	1,460	7.69 (7.68-7.70)	1,199	6.78 (6.77-6.79)
1995-2004	25-30	Schizophrenia spectrum disorder (inpatient)	2,090	6.24 (6.23-6.24)	1,361	7.86 (7.85-7.87)	729	4.50 (4.50-4.51)

			Both sexes		Males		Females	
Years	Age	Mental disorder	Cases	IR (95% CI)	Cases	IR (95% CI)	Cases	IR (95% CI)
2005-2016	25-30	Schizophrenia spectrum disorder (inpatient)	2,380	6.90 (6.90-6.91)	1,480	8.40 (8.39-8.41)	900	5.34 (5.33-5.34)
1970-2016	30-35	Schizophrenia spectrum disorder (inpatient)	9,508	5.96 (5.96-5.96)	5,153	6.31 (6.31-6.32)	4,355	5.59 (5.59-5.59)
1970-1984	30-35	Schizophrenia spectrum disorder (inpatient)	3,670	6.79 (6.78-6.80)	1,819	6.61 (6.60-6.62)	1,851	6.98 (6.97-6.99)
1985-1994	30-35	Schizophrenia spectrum disorder (inpatient)	2,234	6.50 (6.50-6.51)	1,165	6.60 (6.59-6.61)	1,069	6.40 (6.39-6.41)
1995-2004	30-35	Schizophrenia spectrum disorder (inpatient)	1,889	5.28 (5.28-5.29)	1,100	5.97 (5.96-5.98)	789	4.55 (4.54-4.55)
2005-2016	30-35	Schizophrenia spectrum disorder (inpatient)	1,715	4.85 (4.84-4.85)	1,069	5.93 (5.92-5.94)	646	3.72 (3.72-3.73)
1970-2016	35-40	Schizophrenia spectrum disorder (inpatient)	8,544	5.41 (5.41-5.42)	4,308	5.37 (5.37-5.37)	4,236	5.46 (5.46-5.46)
1970-1984	35-40	Schizophrenia spectrum disorder (inpatient)	3,267	6.64 (6.63-6.64)	1,464	5.88 (5.87-5.89)	1,803	7.41 (7.40-7.42)
1985-1994	35-40	Schizophrenia spectrum disorder (inpatient)	2,080	6.01 (6.00-6.01)	986	5.60 (5.60-5.61)	1,094	6.42 (6.41-6.43)
1995-2004	35-40	Schizophrenia spectrum disorder (inpatient)	1,665	4.67 (4.67-4.68)	959	5.27 (5.26-5.27)	706	4.06 (4.05-4.06)
2005-2016	35-40	Schizophrenia spectrum disorder (inpatient)	1,532	4.00 (3.99-4.00)	899	4.60 (4.60-4.61)	633	3.36 (3.36-3.37)
1970-2016	40-45	Schizophrenia spectrum disorder (inpatient)	7,552	4.89 (4.89-4.89)	3,479	4.46 (4.46-4.47)	4,073	5.33 (5.32-5.33)
1970-1984	40-45	Schizophrenia spectrum disorder (inpatient)	3,099	7.18 (7.17-7.18)	1,286	5.95 (5.94-5.96)	1,813	8.41 (8.40-8.42)
1985-1994	40-45	Schizophrenia spectrum disorder (inpatient)	1,760	4.82 (4.82-4.83)	717	3.90 (3.89-3.90)	1,043	5.76 (5.76-5.77)
1995-2004	40-45	Schizophrenia spectrum disorder (inpatient)	1,309	3.91 (3.91-3.91)	675	3.97 (3.97-3.98)	634	3.85 (3.84-3.85)
2005-2016	40-45	Schizophrenia spectrum disorder (inpatient)	1,384	3.35 (3.35-3.36)	801	3.82 (3.82-3.83)	583	2.87 (2.87-2.88)
1970-2016	45-50	Schizophrenia spectrum disorder (inpatient)	6,859	4.57 (4.57-4.57)	2,820	3.75 (3.74-3.75)	4,039	5.39 (5.39-5.40)
1970-1984	45-50	Schizophrenia spectrum disorder (inpatient)	3,102	7.42 (7.42-7.43)	1,135	5.48 (5.47-5.49)	1,967	9.33 (9.32-9.35)
1985-1994	45-50	Schizophrenia spectrum disorder (inpatient)	1,485	4.44 (4.43-4.44)	559	3.32 (3.32-3.33)	926	5.56 (5.55-5.56)
1995-2004	45-50	Schizophrenia spectrum disorder (inpatient)	1,077	3.21 (3.21-3.21)	520	3.08 (3.08-3.09)	557	3.34 (3.33-3.34)
2005-2016	45-50	Schizophrenia spectrum disorder (inpatient)	1,195	2.89 (2.89-2.89)	606	2.90 (2.90-2.91)	589	2.88 (2.87-2.88)
1970-2016	50-55	Schizophrenia spectrum disorder (inpatient)	5,984	4.16 (4.16-4.16)	2,178	3.04 (3.04-3.05)	3,806	5.27 (5.26-5.27)
1970-1984	50-55	Schizophrenia spectrum disorder (inpatient)	2,979	7.09 (7.08-7.09)	1,008	4.87 (4.86-4.88)	1,971	9.23 (9.22-9.25)
1985-1994	50-55	Schizophrenia spectrum disorder (inpatient)	1,159	4.18 (4.18-4.19)	376	2.73 (2.73-2.74)	783	5.61 (5.61-5.62)
1995-2004	50-55	Schizophrenia spectrum disorder (inpatient)	927	2.66 (2.65-2.66)	357	2.05 (2.05-2.05)	570	3.26 (3.26-3.27)
2005-2016	50-55	Schizophrenia spectrum disorder (inpatient)	919	2.35 (2.34-2.35)	437	2.22 (2.22-2.23)	482	2.47 (2.47-2.47)
1970-2016	55-60	Schizophrenia spectrum disorder (inpatient)	4,859	3.57 (3.57-3.57)	1,750	2.61 (2.61-2.61)	3,109	4.50 (4.50-4.50)
1970-1984	55-60	Schizophrenia spectrum disorder (inpatient)	2,509	6.04 (6.03-6.04)	829	4.09 (4.09-4.10)	1,680	7.89 (7.88-7.90)
1985-1994	55-60	Schizophrenia spectrum disorder (inpatient)	959	3.82 (3.82-3.82)	322	2.62 (2.62-2.63)	637	4.97 (4.96-4.98)
1995-2004	55-60	Schizophrenia spectrum disorder (inpatient)	633	2.01 (2.01-2.02)	242	1.55 (1.55-1.55)	391	2.47 (2.46-2.47)
2005-2016	55-60	Schizophrenia spectrum disorder (inpatient)	758	2.00 (1.99-2.00)	357	1.89 (1.89-1.90)	401	2.10 (2.10-2.10)
1970-2016	60-65	Schizophrenia spectrum disorder (inpatient)	3,968	3.12 (3.12-3.12)	1,383	2.24 (2.24-2.25)	2,585	3.94 (3.93-3.94)
1970-1984	60-65	Schizophrenia spectrum disorder (inpatient)	2,045	5.17 (5.16-5.17)	661	3.48 (3.48-3.49)	1,384	6.71 (6.71-6.72)
1985-1994	60-65	Schizophrenia spectrum disorder (inpatient)	873	3.58 (3.58-3.59)	279	2.39 (2.39-2.40)	594	4.68 (4.67-4.68)
1995-2004	60-65	Schizophrenia spectrum disorder (inpatient)	472	1.87 (1.87-1.88)	194	1.58 (1.58-1.59)	278	2.15 (2.14-2.15)
2005-2016	60-65	Schizophrenia spectrum disorder (inpatient)	578	1.52 (1.51-1.52)	249	1.33 (1.33-1.33)	329	1.70 (1.69-1.70)
1970-2016	65-70	Schizophrenia spectrum disorder (inpatient)	3,424	3.00 (3.00-3.00)	1,109	2.06 (2.06-2.07)	2,315	3.83 (3.83-3.84)
1970-1984	65-70	Schizophrenia spectrum disorder (inpatient)	1,676	4.78 (4.77-4.78)	524	3.23 (3.22-3.23)	1,152	6.11 (6.10-6.12)
1985-1994	65-70	Schizophrenia spectrum disorder (inpatient)	864	3.71 (3.70-3.71)	263	2.44 (2.43-2.44)	601	4.80 (4.79-4.81)
1995-2004	65-70	Schizophrenia spectrum disorder (inpatient)	401	1.86 (1.86-1.86)	121	1.19 (1.18-1.19)	280	2.46 (2.45-2.46)

			Both sexes		Males		Females	
Years	Age	Mental disorder	Cases	IR (95% CI)	Cases	IR (95% CI)	Cases	IR (95% CI)
2005-2016	65-70	Schizophrenia spectrum disorder (inpatient)	483	1.41 (1.41-1.42)	201	1.22 (1.22-1.22)	282	1.60 (1.60-1.60)
1970-2016	70-75	Schizophrenia spectrum disorder (inpatient)	2,972	3.15 (3.14-3.15)	888	2.09 (2.09-2.09)	2,084	4.01 (4.00-4.01)
1970-1984	70-75	Schizophrenia spectrum disorder (inpatient)	1,400	4.89 (4.88-4.90)	412	3.29 (3.29-3.30)	988	6.13 (6.12-6.14)
1985-1994	70-75	Schizophrenia spectrum disorder (inpatient)	820	4.03 (4.03-4.04)	231	2.58 (2.57-2.58)	589	5.18 (5.17-5.19)
1995-2004	70-75	Schizophrenia spectrum disorder (inpatient)	408	2.13 (2.13-2.13)	122	1.42 (1.41-1.42)	286	2.71 (2.71-2.72)
2005-2016	70-75	Schizophrenia spectrum disorder (inpatient)	344	1.31 (1.30-1.31)	123	1.00 (0.99-1.00)	221	1.58 (1.58-1.58)
1970-2016	75-80	Schizophrenia spectrum disorder (inpatient)	2,713	3.76 (3.76-3.77)	755	2.50 (2.50-2.50)	1,958	4.67 (4.67-4.68)
1970-1984	75-80	Schizophrenia spectrum disorder (inpatient)	1,096	5.39 (5.38-5.40)	291	3.52 (3.51-3.53)	805	6.67 (6.66-6.69)
1985-1994	75-80	Schizophrenia spectrum disorder (inpatient)	801	4.95 (4.94-4.95)	215	3.27 (3.27-3.28)	586	6.09 (6.08-6.10)
1995-2004	75-80	Schizophrenia spectrum disorder (inpatient)	467	2.91 (2.91-2.92)	137	2.06 (2.05-2.06)	330	3.52 (3.51-3.52)
2005-2016	75-80	Schizophrenia spectrum disorder (inpatient)	349	1.79 (1.78-1.79)	112	1.29 (1.29-1.29)	237	2.19 (2.18-2.19)
1970-2016	80-85	Schizophrenia spectrum disorder (inpatient)	2,035	4.22 (4.21-4.22)	496	2.71 (2.71-2.72)	1,539	5.13 (5.13-5.14)
1970-1984	80-85	Schizophrenia spectrum disorder (inpatient)	740	6.10 (6.09-6.11)	175	3.79 (3.78-3.80)	565	7.53 (7.51-7.54)
1985-1994	80-85	Schizophrenia spectrum disorder (inpatient)	626	5.78 (5.77-5.80)	139	3.61 (3.60-3.62)	487	6.98 (6.97-7.00)
1995-2004	80-85	Schizophrenia spectrum disorder (inpatient)	368	3.24 (3.23-3.24)	99	2.37 (2.36-2.37)	269	3.75 (3.74-3.75)
2005-2016	80-85	Schizophrenia spectrum disorder (inpatient)	301	2.16 (2.15-2.16)	83	1.47 (1.47-1.48)	218	2.62 (2.61-2.62)
1970-2016	85-90	Schizophrenia spectrum disorder (inpatient)	1,111	4.29 (4.29-4.30)	231	2.69 (2.69-2.70)	880	5.08 (5.08-5.09)
1970-1984	85-90	Schizophrenia spectrum disorder (inpatient)	286	5.20 (5.19-5.22)	63	3.17 (3.16-3.19)	223	6.35 (6.33-6.37)
1985-1994	85-90	Schizophrenia spectrum disorder (inpatient)	344	6.34 (6.32-6.35)	67	4.01 (3.99-4.03)	277	7.37 (7.35-7.39)
1995-2004	85-90	Schizophrenia spectrum disorder (inpatient)	266	4.13 (4.12-4.14)	57	2.84 (2.83-2.85)	209	4.71 (4.70-4.72)
2005-2016	85-90	Schizophrenia spectrum disorder (inpatient)	215	2.53 (2.52-2.53)	44	1.51 (1.51-1.52)	171	3.05 (3.04-3.06)
1970-2016	90-95	Schizophrenia spectrum disorder (inpatient)	347	3.66 (3.65-3.66)	89	3.37 (3.36-3.39)	258	3.77 (3.76-3.78)
1970-1984	90-95	Schizophrenia spectrum disorder (inpatient)	64	4.02 (4.00-4.04)	19	3.46 (3.43-3.49)	45	4.32 (4.29-4.35)
1985-1994	90-95	Schizophrenia spectrum disorder (inpatient)	82	4.44 (4.42-4.46)	21	4.15 (4.12-4.19)	61	4.55 (4.53-4.57)
1995-2004	90-95	Schizophrenia spectrum disorder (inpatient)	95	3.87 (3.86-3.89)	17	2.79 (2.76-2.81)	78	4.23 (4.21-4.25)
2005-2016	90-95	Schizophrenia spectrum disorder (inpatient)	106	2.95 (2.94-2.96)	32	3.29 (3.27-3.31)	74	2.82 (2.81-2.83)
1970-2016	95-100	Schizophrenia spectrum disorder (inpatient)	73	3.69 (3.67-3.70)	16	3.60 (3.57-3.63)	57	3.71 (3.69-3.73)
1970-1984	95-100	Schizophrenia spectrum disorder (inpatient)	6	2.38 (2.35-2.41)	0-4	NA	0-4	NA
1985-1994	95-100	Schizophrenia spectrum disorder (inpatient)	14	4.01 (3.97-4.06)	0-4	NA	10-14	4.20 (4.15-4.25)
1995-2004	95-100	Schizophrenia spectrum disorder (inpatient)	17	3.38 (3.35-3.41)	0-4	NA	13-17	3.47 (3.44-3.51)
2005-2016	95-100	Schizophrenia spectrum disorder (inpatient)	36	4.11 (4.08-4.13)	8	4.51 (4.44-4.57)	28	4.01 (3.98-4.04)
1970-2016	10-15	Schizophrenia	416	0.26 (0.26-0.26)	195	0.24 (0.24-0.24)	221	0.29 (0.29-0.29)
1970-1984	10-15	Schizophrenia	44	0.08 (0.08-0.08)	36	0.12 (0.12-0.12)	8	0.03 (0.03-0.03)
1985-1994	10-15	Schizophrenia	12	0.04 (0.04-0.04)	44,538	0.06 (0.06-0.06)	0-4	NA
1995-2004	10-15	Schizophrenia	88	0.30 (0.30-0.30)	46	0.30 (0.30-0.30)	42	0.29 (0.29-0.29)
2005-2016	10-15	Schizophrenia	272	0.68 (0.68-0.68)	103	0.50 (0.50-0.50)	169	0.86 (0.86-0.86)
1970-2016	15-20	Schizophrenia	5,167	3.22 (3.21-3.22)	2,896	3.51 (3.51-3.52)	2,271	2.90 (2.90-2.91)
1970-1984	15-20	Schizophrenia	1,091	1.91 (1.91-1.91)	764	2.61 (2.60-2.61)	327	1.18 (1.18-1.18)
1985-1994	15-20	Schizophrenia	433	1.23 (1.23-1.23)	291	1.61 (1.60-1.61)	142	0.83 (0.82-0.83)
1995-2004	15-20	Schizophrenia	966	3.40 (3.39-3.40)	552	3.79 (3.78-3.80)	414	2.98 (2.98-2.99)

			Both sexes		Males		Females	
**Years**	**Age**	**Mental disorder**	**Cases**	**IR (95% CI)**	**Cases**	**IR (95% CI)**	**Cases**	**IR (95% CI)**
2005-2016	15-20	Schizophrenia	2,677	6.72 (6.71-6.73)	1,289	6.30 (6.29-6.31)	1,388	7.17 (7.16-7.18)
1970-2016	20-25	Schizophrenia	9,856	6.12 (6.12-6.12)	6,408	7.71 (7.70-7.71)	3,448	4.43 (4.43-4.43)
1970-1984	20-25	Schizophrenia	2,362	4.26 (4.26-4.27)	1,713	5.99 (5.98-6.00)	649	2.42 (2.42-2.42)
1985-1994	20-25	Schizophrenia	1,267	3.40 (3.40-3.40)	875	4.51 (4.51-4.52)	392	2.19 (2.19-2.20)
1995-2004	20-25	Schizophrenia	2,138	6.91 (6.90-6.92)	1,394	8.72 (8.71-8.73)	744	4.97 (4.97-4.98)
2005-2016	20-25	Schizophrenia	4,089	10.94 (10.93-10.95)	2,426	12.64 (12.62-12.66)	1,663	9.15 (9.13-9.16)
1970-2016	25-30	Schizophrenia	7,470	4.63 (4.62-4.63)	5,030	6.06 (6.05-6.06)	2,440	3.11 (3.11-3.11)
1970-1984	25-30	Schizophrenia	2,015	3.56 (3.56-3.56)	1,348	4.64 (4.64-4.65)	667	2.42 (2.42-2.43)
1985-1994	25-30	Schizophrenia	1,335	3.63 (3.62-3.63)	920	4.83 (4.82-4.83)	415	2.34 (2.34-2.34)
1995-2004	25-30	Schizophrenia	1,847	5.50 (5.49-5.51)	1,273	7.34 (7.33-7.35)	574	3.54 (3.53-3.54)
2005-2016	25-30	Schizophrenia	2,273	6.59 (6.58-6.60)	1,489	8.45 (8.44-8.46)	784	4.64 (4.64-4.65)
1970-2016	30-35	Schizophrenia	5,811	3.63 (3.63-3.63)	3,686	4.50 (4.50-4.51)	2,125	2.72 (2.72-2.72)
1970-1984	30-35	Schizophrenia	1,539	2.84 (2.84-2.84)	931	3.37 (3.37-3.38)	608	2.29 (2.28-2.29)
1985-1994	30-35	Schizophrenia	979	2.84 (2.83-2.84)	638	3.60 (3.59-3.60)	341	2.03 (2.03-2.03)
1995-2004	30-35	Schizophrenia	1,730	4.82 (4.81-4.82)	1,109	6.00 (5.99-6.01)	621	3.56 (3.56-3.57)
2005-2016	30-35	Schizophrenia	1,563	4.41 (4.41-4.42)	1,008	5.58 (5.58-5.59)	555	3.19 (3.19-3.20)
1970-2016	35-40	Schizophrenia	4,587	2.89 (2.89-2.90)	2,729	3.39 (3.39-3.39)	1,858	2.38 (2.38-2.38)
1970-1984	35-40	Schizophrenia	1,166	2.36 (2.36-2.36)	651	2.61 (2.61-2.61)	515	2.11 (2.11-2.11)
1985-1994	35-40	Schizophrenia	801	2.30 (2.30-2.30)	457	2.58 (2.58-2.59)	344	2.00 (2.00-2.01)
1995-2004	35-40	Schizophrenia	1,457	4.07 (4.07-4.07)	893	4.88 (4.88-4.89)	564	3.22 (3.22-3.22)
2005-2016	35-40	Schizophrenia	1,163	3.03 (3.02-3.03)	728	3.72 (3.72-3.73)	435	2.30 (2.30-2.31)
1970-2016	40-45	Schizophrenia	3,667	2.36 (2.36-2.36)	1,998	2.55 (2.55-2.55)	1,669	2.17 (2.17-2.17)
1970-1984	40-45	Schizophrenia	968	2.23 (2.23-2.24)	460	2.12 (2.12-2.13)	508	2.34 (2.34-2.35)
1985-1994	40-45	Schizophrenia	592	1.61 (1.61-1.61)	320	1.73 (1.73-1.73)	272	1.49 (1.49-1.49)
1995-2004	40-45	Schizophrenia	1,099	3.26 (3.26-3.27)	608	3.56 (3.55-3.56)	491	2.96 (2.95-2.96)
2005-2016	40-45	Schizophrenia	1,008	2.43 (2.43-2.44)	610	2.90 (2.90-2.91)	398	1.95 (1.95-1.95)
1970-2016	45-50	Schizophrenia	2,981	1.97 (1.97-1.97)	1,499	1.98 (1.98-1.98)	1,482	1.96 (1.96-1.97)
1970-1984	45-50	Schizophrenia	838	2.00 (2.00-2.00)	364	1.75 (1.75-1.75)	474	2.24 (2.23-2.24)
1985-1994	45-50	Schizophrenia	459	1.36 (1.36-1.36)	209	1.24 (1.23-1.24)	250	1.49 (1.48-1.49)
1995-2004	45-50	Schizophrenia	893	2.64 (2.64-2.65)	487	2.87 (2.87-2.88)	406	2.41 (2.41-2.42)
2005-2016	45-50	Schizophrenia	791	1.90 (1.90-1.90)	439	2.09 (2.09-2.10)	352	1.71 (1.71-1.71)
1970-2016	50-55	Schizophrenia	2,408	1.66 (1.66-1.66)	1,011	1.41 (1.41-1.41)	1,397	1.92 (1.92-1.92)
1970-1984	50-55	Schizophrenia	815	1.93 (1.93-1.93)	330	1.59 (1.59-1.59)	485	2.26 (2.26-2.26)
1985-1994	50-55	Schizophrenia	285	1.02 (1.02-1.02)	105	0.76 (0.76-0.76)	180	1.28 (1.28-1.28)
1995-2004	50-55	Schizophrenia	698	1.99 (1.98-1.99)	279	1.59 (1.59-1.59)	419	2.38 (2.37-2.38)
2005-2016	50-55	Schizophrenia	610	1.55 (1.55-1.55)	297	1.50 (1.50-1.51)	313	1.59 (1.59-1.59)
1970-2016	55-60	Schizophrenia	1,811	1.32 (1.32-1.32)	710	1.05 (1.05-1.06)	1,101	1.58 (1.58-1.58)
1970-1984	55-60	Schizophrenia	687	1.65 (1.64-1.65)	232	1.14 (1.14-1.14)	455	2.12 (2.12-2.13)
1985-1994	55-60	Schizophrenia	228	0.90 (0.90-0.90)	90	0.73 (0.73-0.73)	138	1.06 (1.06-1.07)
1995-2004	55-60	Schizophrenia	489	1.54 (1.54-1.55)	198	1.26 (1.26-1.26)	291	1.82 (1.82-1.82)

			Both sexes		Males		Females	
**Years**	**Age**	**Mental disorder**	**Cases**	**IR (95% CI)**	**Cases**	**IR (95% CI)**	**Cases**	**IR (95% CI)**
2005-2016	55-60	Schizophrenia	407	1.06 (1.06-1.07)	190	1.00 (1.00-1.00)	217	1.13 (1.12-1.13)
1970-2016	60-65	Schizophrenia	1,282	1.00 (1.00-1.00)	456	0.74 (0.74-0.74)	826	1.25 (1.25-1.25)
1970-1984	60-65	Schizophrenia	487	1.23 (1.22-1.23)	141	0.74 (0.74-0.74)	346	1.67 (1.67-1.67)
1985-1994	60-65	Schizophrenia	163	0.66 (0.66-0.66)	69	0.59 (0.59-0.59)	94	0.73 (0.73-0.73)
1995-2004	60-65	Schizophrenia	323	1.27 (1.27-1.27)	123	1.00 (1.00-1.00)	200	1.53 (1.53-1.53)
2005-2016	60-65	Schizophrenia	309	0.80 (0.80-0.81)	123	0.65 (0.65-0.65)	186	0.95 (0.95-0.95)
1970-2016	65-70	Schizophrenia	982	0.86 (0.85-0.86)	310	0.57 (0.57-0.58)	672	1.10 (1.10-1.10)
1970-1984	65-70	Schizophrenia	363	1.03 (1.03-1.03)	102	0.63 (0.63-0.63)	261	1.38 (1.38-1.38)
1985-1994	65-70	Schizophrenia	167	0.71 (0.71-0.71)	67	0.62 (0.62-0.62)	100	0.79 (0.79-0.79)
1995-2004	65-70	Schizophrenia	249	1.14 (1.14-1.15)	70	0.68 (0.68-0.68)	179	1.55 (1.55-1.56)
2005-2016	65-70	Schizophrenia	203	0.59 (0.59-0.59)	71	0.43 (0.43-0.43)	132	0.74 (0.74-0.74)
1970-2016	70-75	Schizophrenia	695	0.73 (0.73-0.73)	219	0.51 (0.51-0.51)	476	0.91 (0.91-0.91)
1970-1984	70-75	Schizophrenia	224	0.78 (0.78-0.78)	71	0.57 (0.57-0.57)	153	0.95 (0.94-0.95)
1985-1994	70-75	Schizophrenia	95	0.46 (0.46-0.46)	24	0.27 (0.27-0.27)	71	0.62 (0.62-0.62)
1995-2004	70-75	Schizophrenia	228	1.18 (1.18-1.18)	67	0.77 (0.77-0.78)	161	1.51 (1.51-1.51)
2005-2016	70-75	Schizophrenia	148	0.56 (0.56-0.56)	57	0.46 (0.46-0.46)	91	0.64 (0.64-0.65)
1970-2016	75-80	Schizophrenia	479	0.66 (0.66-0.66)	133	0.44 (0.44-0.44)	346	0.82 (0.82-0.82)
1970-1984	75-80	Schizophrenia	153	0.75 (0.75-0.75)	41	0.49 (0.49-0.50)	112	0.93 (0.92-0.93)
1985-1994	75-80	Schizophrenia	65	0.40 (0.40-0.40)	16	0.24 (0.24-0.24)	49	0.50 (0.50-0.51)
1995-2004	75-80	Schizophrenia	168	1.04 (1.04-1.04)	46	0.69 (0.69-0.69)	122	1.29 (1.28-1.29)
2005-2016	75-80	Schizophrenia	93	0.47 (0.47-0.47)	30	0.34 (0.34-0.34)	63	0.58 (0.57-0.58)
1970-2016	80-85	Schizophrenia	287	0.59 (0.59-0.59)	69	0.38 (0.38-0.38)	218	0.72 (0.72-0.72)
1970-1984	80-85	Schizophrenia	65	0.53 (0.53-0.54)	9	0.19 (0.19-0.20)	56	0.74 (0.74-0.74)
1985-1994	80-85	Schizophrenia	52	0.48 (0.48-0.48)	16	0.41 (0.41-0.42)	36	0.51 (0.51-0.51)
1995-2004	80-85	Schizophrenia	106	0.93 (0.92-0.93)	24	0.57 (0.57-0.57)	82	1.13 (1.13-1.13)
2005-2016	80-85	Schizophrenia	64	0.46 (0.45-0.46)	20	0.35 (0.35-0.35)	44	0.52 (0.52-0.52)
1970-2016	85-90	Schizophrenia	121	0.46 (0.46-0.47)	23	0.27 (0.27-0.27)	98	0.56 (0.56-0.56)
1970-1984	85-90	Schizophrenia	17	0.31 (0.31-0.31)	0-4	NA	13-17	0.40 (0.40-0.40)
1985-1994	85-90	Schizophrenia	19	0.35 (0.35-0.35)	0-4	NA	15-19	0.40 (0.39-0.40)
1995-2004	85-90	Schizophrenia	41	0.63 (0.63-0.63)	7	0.35 (0.35-0.35)	34	0.76 (0.76-0.76)
2005-2016	85-90	Schizophrenia	44	0.51 (0.51-0.51)	9	0.31 (0.31-0.31)	35	0.62 (0.62-0.62)
1970-2016	90-95	Schizophrenia	33	0.35 (0.35-0.35)	5	0.19 (0.19-0.19)	28	0.41 (0.41-0.41)
1970-1984	90-95	Schizophrenia	6	0.38 (0.37-0.38)	0-4	NA	0-4	NA
1985-1994	90-95	Schizophrenia	0-4	NA	0-4	NA	0-4	NA
1995-2004	90-95	Schizophrenia	14	0.57 (0.56-0.57)	0-4	NA	10-14	0.65 (0.64-0.65)
2005-2016	90-95	Schizophrenia	12	0.33 (0.33-0.33)	0-4	NA	8-12	0.42 (0.42-0.42)
1970-2016	95-100	Schizophrenia	0-4	NA	0-4	NA	0-4	NA
1970-1984	95-100	Schizophrenia	0-4	NA	0-4	NA	0-4	NA
1985-1994	95-100	Schizophrenia	0-4	NA	0-4	NA	0-4	NA
1995-2004	95-100	Schizophrenia	0-4	NA	0-4	NA	0-4	NA

			Both sexes		Males		Females	
**Years**	**Age**	**Mental disorder**	**Cases**	**IR (95% CI)**	**Cases**	**IR (95% CI)**	**Cases**	**IR (95% CI)**
2005-2016	95-100	Schizophrenia	0-4	NA	0-4	NA	0-4	NA
1970-2016	10-15	Schizophrenia (inpatient)	249	0.16 (0.16-0.16)	118	0.15 (0.15-0.15)	131	0.17 (0.17-0.17)
1970-1984	10-15	Schizophrenia (inpatient)	44	0.08 (0.08-0.08)	36	0.12 (0.12-0.12)	8	0.03 (0.03-0.03)
1985-1994	10-15	Schizophrenia (inpatient)	12	0.04 (0.04-0.04)	8-12	0.06 (0.06-0.06)	0-4	NA
1995-2004	10-15	Schizophrenia (inpatient)	49	0.17 (0.17-0.17)	27	0.18 (0.18-0.18)	22	0.15 (0.15-0.15)
2005-2016	10-15	Schizophrenia (inpatient)	144	0.36 (0.36-0.36)	45	0.22 (0.22-0.22)	99	0.51 (0.51-0.51)
1970-2016	15-20	Schizophrenia (inpatient)	3646	2.27 (2.27-2.27)	2090	2.53 (2.53-2.54)	1556	1.99 (1.99-1.99)
1970-1984	15-20	Schizophrenia (inpatient)	1091	1.91 (1.91-1.91)	764	2.61 (2.60-2.61)	327	1.18 (1.18-1.18)
1985-1994	15-20	Schizophrenia (inpatient)	425	1.20 (1.20-1.20)	287	1.58 (1.58-1.59)	138	0.80 (0.80-0.80)
1995-2004	15-20	Schizophrenia (inpatient)	697	2.45 (2.45-2.45)	396	2.72 (2.71-2.72)	301	2.17 (2.16-2.17)
2005-2016	15-20	Schizophrenia (inpatient)	1433	3.60 (3.59-3.60)	643	3.14 (3.14-3.15)	790	4.08 (4.07-4.08)
1970-2016	20-25	Schizophrenia (inpatient)	7550	4.69 (4.68-4.69)	4990	6.00 (5.99-6.00)	2560	3.29 (3.28-3.29)
1970-1984	20-25	Schizophrenia (inpatient)	2362	4.26 (4.26-4.27)	1713	5.99 (5.98-6.00)	649	2.42 (2.42-2.42)
1985-1994	20-25	Schizophrenia (inpatient)	1231	3.30 (3.30-3.31)	855	4.41 (4.40-4.42)	376	2.10 (2.10-2.11)
1995-2004	20-25	Schizophrenia (inpatient)	1591	5.14 (5.13-5.14)	1035	6.47 (6.46-6.48)	556	3.72 (3.71-3.72)
2005-2016	20-25	Schizophrenia (inpatient)	2366	6.32 (6.31-6.32)	1387	7.21 (7.20-7.22)	979	5.37 (5.37-5.38)
1970-2016	25-30	Schizophrenia (inpatient)	6216	3.85 (3.85-3.85)	4183	5.03 (5.03-5.03)	2033	2.59 (2.59-2.59)
1970-1984	25-30	Schizophrenia (inpatient)	2015	3.56 (3.56-3.56)	1348	4.64 (4.64-4.65)	667	2.42 (2.42-2.43)
1985-1994	25-30	Schizophrenia (inpatient)	1282	3.48 (3.48-3.49)	879	4.61 (4.61-4.62)	403	2.27 (2.27-2.27)
1995-2004	25-30	Schizophrenia (inpatient)	1397	4.16 (4.15-4.16)	977	5.63 (5.62-5.63)	420	2.59 (2.58-2.59)
2005-2016	25-30	Schizophrenia (inpatient)	1522	4.40 (4.40-4.40)	979	5.54 (5.53-5.55)	543	3.21 (3.20-3.21)
1970-2016	30-35	Schizophrenia (inpatient)	4700	2.93 (2.93-2.94)	2964	3.62 (3.62-3.62)	1736	2.22 (2.22-2.22)
1970-1984	30-35	Schizophrenia (inpatient)	1539	2.84 (2.84-2.84)	931	3.37 (3.37-3.38)	608	2.29 (2.28-2.29)
1985-1994	30-35	Schizophrenia (inpatient)	923	2.67 (2.67-2.68)	602	3.39 (3.39-3.40)	321	1.91 (1.91-1.91)
1995-2004	30-35	Schizophrenia (inpatient)	1228	3.42 (3.41-3.42)	761	4.11 (4.11-4.12)	467	2.68 (2.67-2.68)
2005-2016	30-35	Schizophrenia (inpatient)	1010	2.84 (2.84-2.85)	670	3.70 (3.70-3.71)	340	1.95 (1.95-1.95)
1970-2016	35-40	Schizophrenia (inpatient)	3798	2.39 (2.39-2.40)	2266	2.81 (2.81-2.81)	1532	1.96 (1.96-1.97)
1970-1984	35-40	Schizophrenia (inpatient)	1166	2.36 (2.36-2.36)	651	2.61 (2.61-2.61)	515	2.11 (2.11-2.11)
1985-1994	35-40	Schizophrenia (inpatient)	745	2.14 (2.14-2.14)	428	2.42 (2.42-2.42)	317	1.85 (1.84-1.85)
1995-2004	35-40	Schizophrenia (inpatient)	1079	3.01 (3.01-3.01)	668	3.65 (3.64-3.65)	411	2.35 (2.34-2.35)
2005-2016	35-40	Schizophrenia (inpatient)	808	2.10 (2.10-2.10)	519	2.65 (2.64-2.65)	289	1.53 (1.53-1.53)
1970-2016	40-45	Schizophrenia (inpatient)	2943	1.90 (1.89-1.90)	1579	2.02 (2.01-2.02)	1364	1.77 (1.77-1.77)
1970-1984	40-45	Schizophrenia (inpatient)	968	2.23 (2.23-2.24)	460	2.12 (2.12-2.13)	508	2.34 (2.34-2.35)
1985-1994	40-45	Schizophrenia (inpatient)	544	1.48 (1.48-1.48)	291	1.57 (1.57-1.57)	253	1.39 (1.39-1.39)
1995-2004	40-45	Schizophrenia (inpatient)	717	2.13 (2.12-2.13)	390	2.28 (2.28-2.28)	327	1.97 (1.96-1.97)
2005-2016	40-45	Schizophrenia (inpatient)	714	1.72 (1.72-1.72)	438	2.08 (2.08-2.08)	276	1.35 (1.35-1.35)
1970-2016	45-50	Schizophrenia (inpatient)	2402	1.59 (1.59-1.59)	1175	1.55 (1.55-1.55)	1227	1.63 (1.62-1.63)
1970-1984	45-50	Schizophrenia (inpatient)	835	1.99 (1.99-1.99)	362	1.74 (1.74-1.75)	473	2.23 (2.23-2.24)
1985-1994	45-50	Schizophrenia (inpatient)	413	1.22 (1.22-1.23)	184	1.09 (1.09-1.09)	229	1.36 (1.36-1.36)
1995-2004	45-50	Schizophrenia (inpatient)	622	1.84 (1.84-1.84)	342	2.01 (2.01-2.02)	280	1.66 (1.66-1.67)

			Both sexes		Males		Females	
**Years**	**Age**	**Mental disorder**	**Cases**	**IR (95% CI)**	**Cases**	**IR (95% CI)**	**Cases**	**IR (95% CI)**
2005-2016	45-50	Schizophrenia (inpatient)	532	1.28 (1.28-1.28)	287	1.37 (1.36-1.37)	245	1.19 (1.19-1.19)
1970-2016	50-55	Schizophrenia (inpatient)	1971	1.36 (1.36-1.36)	824	1.15 (1.15-1.15)	1147	1.57 (1.57-1.57)
1970-1984	50-55	Schizophrenia (inpatient)	815	1.93 (1.93-1.93)	330	1.59 (1.59-1.59)	485	2.26 (2.26-2.26)
1985-1994	50-55	Schizophrenia (inpatient)	253	0.91 (0.91-0.91)	87	0.63 (0.63-0.63)	166	1.18 (1.18-1.18)
1995-2004	50-55	Schizophrenia (inpatient)	473	1.34 (1.34-1.35)	191	1.09 (1.09-1.09)	282	1.60 (1.60-1.60)
2005-2016	50-55	Schizophrenia (inpatient)	430	1.09 (1.09-1.09)	216	1.09 (1.09-1.09)	214	1.09 (1.09-1.09)
1970-2016	55-60	Schizophrenia (inpatient)	1466	1.07 (1.07-1.07)	545	0.81 (0.81-0.81)	921	1.32 (1.32-1.32)
1970-1984	55-60	Schizophrenia (inpatient)	687	1.65 (1.64-1.65)	232	1.14 (1.14-1.14)	455	2.12 (2.12-2.13)
1985-1994	55-60	Schizophrenia (inpatient)	200	0.79 (0.79-0.79)	77	0.62 (0.62-0.62)	123	0.95 (0.95-0.95)
1995-2004	55-60	Schizophrenia (inpatient)	293	0.92 (0.92-0.93)	113	0.72 (0.72-0.72)	180	1.12 (1.12-1.13)
2005-2016	55-60	Schizophrenia (inpatient)	286	0.75 (0.75-0.75)	123	0.65 (0.65-0.65)	163	0.84 (0.84-0.85)
1970-2016	60-65	Schizophrenia (inpatient)	1032	0.81 (0.81-0.81)	343	0.55 (0.55-0.55)	689	1.04 (1.04-1.04)
1970-1984	60-65	Schizophrenia (inpatient)	487	1.23 (1.22-1.23)	141	0.74 (0.74-0.74)	346	1.67 (1.67-1.67)
1985-1994	60-65	Schizophrenia (inpatient)	151	0.61 (0.61-0.62)	64	0.55 (0.55-0.55)	87	0.68 (0.68-0.68)
1995-2004	60-65	Schizophrenia (inpatient)	191	0.75 (0.75-0.75)	68	0.55 (0.55-0.55)	123	0.94 (0.94-0.94)
2005-2016	60-65	Schizophrenia (inpatient)	203	0.53 (0.53-0.53)	70	0.37 (0.37-0.37)	133	0.68 (0.68-0.68)
1970-2016	65-70	Schizophrenia (inpatient)	799	0.70 (0.70-0.70)	253	0.47 (0.47-0.47)	546	0.90 (0.90-0.90)
1970-1984	65-70	Schizophrenia (inpatient)	363	1.03 (1.03-1.03)	102	0.63 (0.63-0.63)	261	1.38 (1.38-1.38)
1985-1994	65-70	Schizophrenia (inpatient)	149	0.63 (0.63-0.63)	63	0.58 (0.58-0.58)	86	0.68 (0.68-0.68)
1995-2004	65-70	Schizophrenia (inpatient)	140	0.64 (0.64-0.64)	29	0.28 (0.28-0.28)	111	0.96 (0.96-0.97)
2005-2016	65-70	Schizophrenia (inpatient)	147	0.43 (0.43-0.43)	59	0.36 (0.36-0.36)	88	0.49 (0.49-0.49)
1970-2016	70-75	Schizophrenia (inpatient)	526	0.55 (0.55-0.55)	162	0.38 (0.38-0.38)	364	0.69 (0.69-0.69)
1970-1984	70-75	Schizophrenia (inpatient)	224	0.78 (0.78-0.78)	71	0.57 (0.57-0.57)	153	0.95 (0.94-0.95)
1985-1994	70-75	Schizophrenia (inpatient)	84	0.41 (0.41-0.41)	23	0.26 (0.26-0.26)	61	0.53 (0.53-0.53)
1995-2004	70-75	Schizophrenia (inpatient)	131	0.68 (0.68-0.68)	39	0.45 (0.45-0.45)	92	0.86 (0.86-0.87)
2005-2016	70-75	Schizophrenia (inpatient)	87	0.33 (0.33-0.33)	29	0.23 (0.23-0.23)	58	0.41 (0.41-0.41)
1970-2016	75-80	Schizophrenia (inpatient)	346	0.48 (0.48-0.48)	95	0.31 (0.31-0.31)	251	0.59 (0.59-0.59)
1970-1984	75-80	Schizophrenia (inpatient)	153	0.75 (0.75-0.75)	41	0.49 (0.49-0.50)	112	0.93 (0.92-0.93)
1985-1994	75-80	Schizophrenia (inpatient)	58	0.36 (0.36-0.36)	15	0.23 (0.23-0.23)	43	0.44 (0.44-0.44)
1995-2004	75-80	Schizophrenia (inpatient)	83	0.51 (0.51-0.51)	21	0.31 (0.31-0.31)	62	0.65 (0.65-0.66)
2005-2016	75-80	Schizophrenia (inpatient)	52	0.26 (0.26-0.26)	18	0.21 (0.21-0.21)	34	0.31 (0.31-0.31)
1970-2016	80-85	Schizophrenia (inpatient)	187	0.39 (0.38-0.39)	42	0.23 (0.23-0.23)	145	0.48 (0.48-0.48)
1970-1984	80-85	Schizophrenia (inpatient)	65	0.53 (0.53-0.54)	9	0.19 (0.19-0.20)	56	0.74 (0.74-0.74)
1985-1994	80-85	Schizophrenia (inpatient)	48	0.44 (0.44-0.44)	15	0.39 (0.39-0.39)	33	0.47 (0.47-0.47)
1995-2004	80-85	Schizophrenia (inpatient)	40	0.35 (0.35-0.35)	7	0.17 (0.17-0.17)	33	0.46 (0.45-0.46)
2005-2016	80-85	Schizophrenia (inpatient)	34	0.24 (0.24-0.24)	11	0.19 (0.19-0.20)	23	0.27 (0.27-0.27)
1970-2016	85-90	Schizophrenia (inpatient)	66	0.25 (0.25-0.25)	10	0.12 (0.12-0.12)	56	0.32 (0.32-0.32)
1970-1984	85-90	Schizophrenia (inpatient)	17	0.31 (0.31-0.31)	0-4	NA	13-17	0.40 (0.40-0.40)
1985-1994	85-90	Schizophrenia (inpatient)	17	0.31 (0.31-0.31)	0-4	NA	13-17	0.34 (0.34-0.34)
1995-2004	85-90	Schizophrenia (inpatient)	15	0.23 (0.23-0.23)	0-4	NA	11-15	0.29 (0.29-0.29)

			Both sexes		Males		Females	
**Years**	**Age**	**Mental disorder**	**Cases**	**IR (95% CI)**	**Cases**	**IR (95% CI)**	**Cases**	**IR (95% CI)**
2005-2016	85-90	Schizophrenia (inpatient)	17	0.20 (0.20-0.20)	0-4	NA	13-17	0.28 (0.28-0.28)
1970-2016	90-95	Schizophrenia (inpatient)	18	0.19 (0.19-0.19)	0-4	NA	14-18	0.20 (0.20-0.20)
1970-1984	90-95	Schizophrenia (inpatient)	6	0.38 (0.37-0.38)	0-4	NA	0-4	NA
1985-1994	90-95	Schizophrenia (inpatient)	0-4	NA	0-4	NA	0-4	NA
1995-2004	90-95	Schizophrenia (inpatient)	6	0.24 (0.24-0.24)	0-4	NA	2-6	0.27 (0.27-0.27)
2005-2016	90-95	Schizophrenia (inpatient)	5	0.14 (0.14-0.14)	0-4	NA	0-4	NA
1970-2016	95-100	Schizophrenia (inpatient)	0-4	NA	0-4	NA	0-4	NA
1970-1984	95-100	Schizophrenia (inpatient)	0-4	NA	0-4	NA	0-4	NA
1985-1994	95-100	Schizophrenia (inpatient)	0-4	NA	0-4	NA	0-4	NA
1995-2004	95-100	Schizophrenia (inpatient)	0-4	NA	0-4	NA	0-4	NA
2005-2016	95-100	Schizophrenia (inpatient)	0-4	NA	0-4	NA	0-4	NA
1970-2016	10-15	Schizoaffective disorders	36	0.02 (0.02-0.02)	13	0.02 (0.02-0.02)	23	0.03 (0.03-0.03)
1970-1984	10-15	Schizoaffective disorders	0-4	NA	0-4	NA	0-4	NA
1985-1994	10-15	Schizoaffective disorders	0-4	NA	0-4	NA	0-4	NA
1995-2004	10-15	Schizoaffective disorders	14	0.05 (0.05-0.05)	7	0.05 (0.05-0.05)	7	0.05 (0.05-0.05)
2005-2016	10-15	Schizoaffective disorders	17	0.04 (0.04-0.04)	0-4	NA	13-17	0.07 (0.07-0.07)
1970-2016	15-20	Schizoaffective disorders	406	0.25 (0.25-0.25)	133	0.16 (0.16-0.16)	273	0.35 (0.35-0.35)
1970-1984	15-20	Schizoaffective disorders	73	0.13 (0.13-0.13)	34	0.12 (0.12-0.12)	39	0.14 (0.14-0.14)
1985-1994	15-20	Schizoaffective disorders	40	0.11 (0.11-0.11)	14	0.08 (0.08-0.08)	26	0.15 (0.15-0.15)
1995-2004	15-20	Schizoaffective disorders	109	0.38 (0.38-0.38)	39	0.27 (0.27-0.27)	70	0.50 (0.50-0.50)
2005-2016	15-20	Schizoaffective disorders	184	0.46 (0.46-0.46)	46	0.22 (0.22-0.22)	138	0.71 (0.71-0.71)
1970-2016	20-25	Schizoaffective disorders	835	0.52 (0.52-0.52)	423	0.51 (0.51-0.51)	412	0.53 (0.53-0.53)
1970-1984	20-25	Schizoaffective disorders	223	0.40 (0.40-0.40)	129	0.45 (0.45-0.45)	94	0.35 (0.35-0.35)
1985-1994	20-25	Schizoaffective disorders	99	0.27 (0.27-0.27)	59	0.30 (0.30-0.30)	40	0.22 (0.22-0.22)
1995-2004	20-25	Schizoaffective disorders	269	0.87 (0.87-0.87)	125	0.78 (0.78-0.78)	144	0.96 (0.96-0.96)
2005-2016	20-25	Schizoaffective disorders	244	0.65 (0.65-0.65)	110	0.57 (0.57-0.57)	134	0.73 (0.73-0.73)
1970-2016	25-30	Schizoaffective disorders	959	0.59 (0.59-0.59)	479	0.57 (0.57-0.57)	480	0.61 (0.61-0.61)
1970-1984	25-30	Schizoaffective disorders	300	0.53 (0.53-0.53)	156	0.54 (0.53-0.54)	144	0.52 (0.52-0.52)
1985-1994	25-30	Schizoaffective disorders	152	0.41 (0.41-0.41)	84	0.44 (0.44-0.44)	68	0.38 (0.38-0.38)
1995-2004	25-30	Schizoaffective disorders	266	0.79 (0.79-0.79)	142	0.81 (0.81-0.82)	124	0.76 (0.76-0.76)
2005-2016	25-30	Schizoaffective disorders	241	0.69 (0.69-0.69)	97	0.55 (0.54-0.55)	144	0.85 (0.85-0.85)
1970-2016	30-35	Schizoaffective disorders	1090	0.68 (0.68-0.68)	489	0.59 (0.59-0.59)	601	0.77 (0.77-0.77)
1970-1984	30-35	Schizoaffective disorders	345	0.63 (0.63-0.64)	146	0.53 (0.53-0.53)	199	0.75 (0.75-0.75)
1985-1994	30-35	Schizoaffective disorders	210	0.61 (0.61-0.61)	91	0.51 (0.51-0.51)	119	0.71 (0.71-0.71)
1995-2004	30-35	Schizoaffective disorders	327	0.91 (0.91-0.91)	157	0.84 (0.84-0.85)	170	0.97 (0.97-0.97)
2005-2016	30-35	Schizoaffective disorders	208	0.58 (0.58-0.58)	95	0.52 (0.52-0.52)	113	0.65 (0.65-0.65)
1970-2016	35-40	Schizoaffective disorders	1131	0.71 (0.71-0.71)	456	0.56 (0.56-0.56)	675	0.86 (0.86-0.86)
1970-1984	35-40	Schizoaffective disorders	329	0.66 (0.66-0.67)	127	0.51 (0.51-0.51)	202	0.83 (0.82-0.83)
1985-1994	35-40	Schizoaffective disorders	253	0.72 (0.72-0.72)	110	0.62 (0.62-0.62)	143	0.83 (0.83-0.83)
1995-2004	35-40	Schizoaffective disorders	347	0.96 (0.96-0.96)	135	0.73 (0.73-0.73)	212	1.21 (1.20-1.21)

			Both sexes		Males		Females	
Years	Age	Mental disorder	Cases	IR (95% CI)	Cases	IR (95% CI)	Cases	IR (95% CI)
2005-2016	35-40	Schizoaffective disorders	202	0.52 (0.52-0.52)	84	0.43 (0.42-0.43)	118	0.62 (0.62-0.62)
1970-2016	40-45	Schizoaffective disorders	1123	0.72 (0.72-0.72)	467	0.59 (0.59-0.59)	656	0.85 (0.85-0.85)
1970-1984	40-45	Schizoaffective disorders	363	0.84 (0.83-0.84)	152	0.70 (0.70-0.70)	211	0.97 (0.97-0.97)
1985-1994	40-45	Schizoaffective disorders	225	0.61 (0.61-0.61)	83	0.45 (0.45-0.45)	142	0.78 (0.78-0.78)
1995-2004	40-45	Schizoaffective disorders	327	0.96 (0.96-0.97)	137	0.80 (0.79-0.80)	190	1.14 (1.14-1.14)
2005-2016	40-45	Schizoaffective disorders	208	0.50 (0.50-0.50)	95	0.45 (0.45-0.45)	113	0.55 (0.55-0.55)
1970-2016	45-50	Schizoaffective disorders	1048	0.69 (0.69-0.69)	386	0.51 (0.51-0.51)	662	0.87 (0.87-0.88)
1970-1984	45-50	Schizoaffective disorders	368	0.88 (0.87-0.88)	130	0.62 (0.62-0.63)	238	1.12 (1.12-1.12)
1985-1994	45-50	Schizoaffective disorders	211	0.62 (0.62-0.62)	65	0.38 (0.38-0.38)	146	0.87 (0.86-0.87)
1995-2004	45-50	Schizoaffective disorders	290	0.85 (0.85-0.85)	110	0.64 (0.64-0.64)	180	1.07 (1.06-1.07)
2005-2016	45-50	Schizoaffective disorders	179	0.43 (0.43-0.43)	81	0.38 (0.38-0.38)	98	0.47 (0.47-0.47)
1970-2016	50-55	Schizoaffective disorders	1009	0.69 (0.69-0.69)	332	0.46 (0.46-0.46)	677	0.93 (0.93-0.93)
1970-1984	50-55	Schizoaffective disorders	368	0.87 (0.87-0.87)	112	0.54 (0.54-0.54)	256	1.19 (1.19-1.19)
1985-1994	50-55	Schizoaffective disorders	185	0.66 (0.66-0.66)	52	0.37 (0.37-0.38)	133	0.94 (0.94-0.94)
1995-2004	50-55	Schizoaffective disorders	255	0.72 (0.72-0.72)	92	0.52 (0.52-0.52)	163	0.92 (0.92-0.92)
2005-2016	50-55	Schizoaffective disorders	201	0.51 (0.51-0.51)	76	0.38 (0.38-0.38)	125	0.63 (0.63-0.63)
1970-2016	55-60	Schizoaffective disorders	717	0.52 (0.52-0.52)	224	0.33 (0.33-0.33)	493	0.71 (0.71-0.71)
1970-1984	55-60	Schizoaffective disorders	329	0.79 (0.79-0.79)	94	0.46 (0.46-0.46)	235	1.09 (1.09-1.10)
1985-1994	55-60	Schizoaffective disorders	120	0.47 (0.47-0.47)	37	0.30 (0.30-0.30)	83	0.64 (0.64-0.64)
1995-2004	55-60	Schizoaffective disorders	152	0.48 (0.48-0.48)	47	0.30 (0.30-0.30)	105	0.65 (0.65-0.65)
2005-2016	55-60	Schizoaffective disorders	116	0.30 (0.30-0.30)	46	0.24 (0.24-0.24)	70	0.36 (0.36-0.36)
1970-2016	60-65	Schizoaffective disorders	596	0.46 (0.46-0.46)	179	0.29 (0.29-0.29)	417	0.63 (0.63-0.63)
1970-1984	60-65	Schizoaffective disorders	276	0.69 (0.69-0.69)	78	0.41 (0.41-0.41)	198	0.95 (0.95-0.96)
1985-1994	60-65	Schizoaffective disorders	140	0.57 (0.57-0.57)	37	0.32 (0.31-0.32)	103	0.80 (0.80-0.80)
1995-2004	60-65	Schizoaffective disorders	104	0.41 (0.41-0.41)	35	0.28 (0.28-0.28)	69	0.53 (0.53-0.53)
2005-2016	60-65	Schizoaffective disorders	76	0.20 (0.20-0.20)	29	0.15 (0.15-0.15)	47	0.24 (0.24-0.24)
1970-2016	65-70	Schizoaffective disorders	478	0.42 (0.41-0.42)	124	0.23 (0.23-0.23)	354	0.58 (0.58-0.58)
1970-1984	65-70	Schizoaffective disorders	203	0.58 (0.57-0.58)	52	0.32 (0.32-0.32)	151	0.80 (0.79-0.80)
1985-1994	65-70	Schizoaffective disorders	124	0.53 (0.53-0.53)	29	0.27 (0.27-0.27)	95	0.75 (0.75-0.75)
1995-2004	65-70	Schizoaffective disorders	82	0.38 (0.38-0.38)	23	0.22 (0.22-0.22)	59	0.51 (0.51-0.51)
2005-2016	65-70	Schizoaffective disorders	69	0.20 (0.20-0.20)	20	0.12 (0.12-0.12)	49	0.27 (0.27-0.27)
1970-2016	70-75	Schizoaffective disorders	340	0.36 (0.36-0.36)	80	0.19 (0.19-0.19)	260	0.49 (0.49-0.50)
1970-1984	70-75	Schizoaffective disorders	142	0.49 (0.49-0.49)	32	0.25 (0.25-0.25)	110	0.68 (0.68-0.68)
1985-1994	70-75	Schizoaffective disorders	95	0.46 (0.46-0.46)	24	0.27 (0.27-0.27)	71	0.62 (0.62-0.62)
1995-2004	70-75	Schizoaffective disorders	58	0.30 (0.30-0.30)	11	0.13 (0.13-0.13)	47	0.44 (0.44-0.44)
2005-2016	70-75	Schizoaffective disorders	45	0.17 (0.17-0.17)	13	0.10 (0.10-0.10)	32	0.23 (0.23-0.23)
1970-2016	75-80	Schizoaffective disorders	209	0.29 (0.29-0.29)	47	0.15 (0.15-0.16)	162	0.38 (0.38-0.38)
1970-1984	75-80	Schizoaffective disorders	69	0.34 (0.34-0.34)	14	0.17 (0.17-0.17)	55	0.45 (0.45-0.45)
1985-1994	75-80	Schizoaffective disorders	75	0.46 (0.46-0.46)	20	0.30 (0.30-0.30)	55	0.57 (0.56-0.57)
1995-2004	75-80	Schizoaffective disorders	43	0.27 (0.27-0.27)	0-4	NA	39-43	0.41 (0.41-0.41)

			Both sexes		Males		Females	
Years	Age	Mental disorder	Cases	IR (95% CI)	Cases	IR (95% CI)	Cases	IR (95% CI)
2005-2016	75-80	Schizoaffective disorders	22	0.11 (0.11-0.11)	9	0.10 (0.10-0.10)	13	0.12 (0.12-0.12)
1970-2016	80-85	Schizoaffective disorders	112	0.23 (0.23-0.23)	23	0.13 (0.13-0.13)	89	0.29 (0.29-0.29)
1970-1984	80-85	Schizoaffective disorders	35	0.29 (0.29-0.29)	5	0.11 (0.11-0.11)	30	0.40 (0.40-0.40)
1985-1994	80-85	Schizoaffective disorders	34	0.31 (0.31-0.31)	6	0.16 (0.15-0.16)	28	0.40 (0.40-0.40)
1995-2004	80-85	Schizoaffective disorders	24	0.21 (0.21-0.21)	6	0.14 (0.14-0.14)	18	0.25 (0.25-0.25)
2005-2016	80-85	Schizoaffective disorders	19	0.14 (0.13-0.14)	6	0.11 (0.11-0.11)	13	0.15 (0.15-0.15)
1970-2016	85-90	Schizoaffective disorders	34	0.13 (0.13-0.13)	8	0.09 (0.09-0.09)	26	0.15 (0.15-0.15)
1970-1984	85-90	Schizoaffective disorders	8	0.14 (0.14-0.15)	0-4	NA	4-8	0.17 (0.17-0.17)
1985-1994	85-90	Schizoaffective disorders	17	0.31 (0.31-0.31)	0-4	NA	13-17	0.37 (0.37-0.37)
1995-2004	85-90	Schizoaffective disorders	7	0.11 (0.11-0.11)	0-4	NA	3-7	0.11 (0.11-0.11)
2005-2016	85-90	Schizoaffective disorders	0-4	NA	0-4	NA	0-4	NA
1970-2016	90-95	Schizoaffective disorders	6	0.06 (0.06-0.06)	0-4	NA	2-6	0.09 (0.09-0.09)
1970-1984	90-95	Schizoaffective disorders	0-4	NA	0-4	NA	0-4	NA
1985-1994	90-95	Schizoaffective disorders	0-4	NA	0-4	NA	0-4	NA
1995-2004	90-95	Schizoaffective disorders	0-4	NA	0-4	NA	0-4	NA
2005-2016	90-95	Schizoaffective disorders	0-4	NA	0-4	NA	0-4	NA
1970-2016	95-100	Schizoaffective disorders	0-4	NA	0-4	NA	0-4	NA
1970-1984	95-100	Schizoaffective disorders	0-4	NA	0-4	NA	0-4	NA
1985-1994	95-100	Schizoaffective disorders	0-4	NA	0-4	NA	0-4	NA
1995-2004	95-100	Schizoaffective disorders	0-4	NA	0-4	NA	0-4	NA
2005-2016	95-100	Schizoaffective disorders	0-4	NA	0-4	NA	0-4	NA
1970-2016	10-15	Schizoaffective disorders (inpatient)	24	0.02 (0.02-0.02)	8	0.01 (0.01-0.01)	16	0.02 (0.02-0.02)
1970-1984	10-15	Schizoaffective disorders (inpatient)	0-4	NA	0-4	NA	0-4	NA
1985-1994	10-15	Schizoaffective disorders (inpatient)	0-4	NA	0-4	NA	0-4	NA
1995-2004	10-15	Schizoaffective disorders (inpatient)	11	0.04 (0.04-0.04)	0-4	NA	7-11	0.05 (0.05-0.05)
2005-2016	10-15	Schizoaffective disorders (inpatient)	8	0.02 (0.02-0.02)	0-4	NA	4-8	0.04 (0.04-0.04)
1970-2016	15-20	Schizoaffective disorders (inpatient)	284	0.18 (0.18-0.18)	95	0.12 (0.12-0.12)	189	0.24 (0.24-0.24)
1970-1984	15-20	Schizoaffective disorders (inpatient)	73	0.13 (0.13-0.13)	34	0.12 (0.12-0.12)	39	0.14 (0.14-0.14)
1985-1994	15-20	Schizoaffective disorders (inpatient)	37	0.10 (0.10-0.10)	13	0.07 (0.07-0.07)	24	0.14 (0.14-0.14)
1995-2004	15-20	Schizoaffective disorders (inpatient)	75	0.26 (0.26-0.26)	26	0.18 (0.18-0.18)	49	0.35 (0.35-0.35)
2005-2016	15-20	Schizoaffective disorders (inpatient)	99	0.25 (0.25-0.25)	22	0.11 (0.11-0.11)	77	0.40 (0.40-0.40)
1970-2016	20-25	Schizoaffective disorders (inpatient)	638	0.40 (0.40-0.40)	333	0.40 (0.40-0.40)	305	0.39 (0.39-0.39)
1970-1984	20-25	Schizoaffective disorders (inpatient)	223	0.40 (0.40-0.40)	129	0.45 (0.45-0.45)	94	0.35 (0.35-0.35)
1985-1994	20-25	Schizoaffective disorders (inpatient)	94	0.25 (0.25-0.25)	56	0.29 (0.29-0.29)	38	0.21 (0.21-0.21)
1995-2004	20-25	Schizoaffective disorders (inpatient)	176	0.57 (0.57-0.57)	81	0.51 (0.50-0.51)	95	0.63 (0.63-0.64)
2005-2016	20-25	Schizoaffective disorders (inpatient)	145	0.39 (0.39-0.39)	67	0.35 (0.35-0.35)	78	0.43 (0.43-0.43)
1970-2016	25-30	Schizoaffective disorders (inpatient)	773	0.48 (0.48-0.48)	393	0.47 (0.47-0.47)	380	0.48 (0.48-0.48)
1970-1984	25-30	Schizoaffective disorders (inpatient)	300	0.53 (0.53-0.53)	156	0.54 (0.53-0.54)	144	0.52 (0.52-0.52)
1985-1994	25-30	Schizoaffective disorders (inpatient)	144	0.39 (0.39-0.39)	79	0.41 (0.41-0.41)	65	0.37 (0.37-0.37)
1995-2004	25-30	Schizoaffective disorders (inpatient)	186	0.55 (0.55-0.55)	102	0.58 (0.58-0.59)	84	0.52 (0.52-0.52)

			Both sexes		Males		Females	
Years	Age	Mental disorder	Cases	IR (95% CI)	Cases	IR (95% CI)	Cases	IR (95% CI)
2005-2016	25-30	Schizoaffective disorders (inpatient)	143	0.41 (0.41-0.41)	56	0.32 (0.31-0.32)	87	0.51 (0.51-0.51)
1970-2016	30-35	Schizoaffective disorders (inpatient)	905	0.56 (0.56-0.56)	398	0.48 (0.48-0.48)	507	0.65 (0.65-0.65)
1970-1984	30-35	Schizoaffective disorders (inpatient)	345	0.63 (0.63-0.64)	146	0.53 (0.53-0.53)	199	0.75 (0.75-0.75)
1985-1994	30-35	Schizoaffective disorders (inpatient)	192	0.55 (0.55-0.55)	86	0.48 (0.48-0.48)	106	0.63 (0.63-0.63)
1995-2004	30-35	Schizoaffective disorders (inpatient)	231	0.64 (0.64-0.64)	104	0.56 (0.56-0.56)	127	0.73 (0.73-0.73)
2005-2016	30-35	Schizoaffective disorders (inpatient)	137	0.38 (0.38-0.38)	62	0.34 (0.34-0.34)	75	0.43 (0.43-0.43)
1970-2016	35-40	Schizoaffective disorders (inpatient)	943	0.59 (0.59-0.59)	379	0.47 (0.47-0.47)	564	0.72 (0.72-0.72)
1970-1984	35-40	Schizoaffective disorders (inpatient)	328	0.66 (0.66-0.66)	127	0.51 (0.51-0.51)	201	0.82 (0.82-0.82)
1985-1994	35-40	Schizoaffective disorders (inpatient)	236	0.67 (0.67-0.67)	100	0.56 (0.56-0.56)	136	0.79 (0.79-0.79)
1995-2004	35-40	Schizoaffective disorders (inpatient)	234	0.65 (0.65-0.65)	94	0.51 (0.51-0.51)	140	0.80 (0.80-0.80)
2005-2016	35-40	Schizoaffective disorders (inpatient)	145	0.37 (0.37-0.37)	58	0.29 (0.29-0.29)	87	0.46 (0.46-0.46)
1970-2016	40-45	Schizoaffective disorders (inpatient)	973	0.62 (0.62-0.62)	393	0.50 (0.50-0.50)	580	0.75 (0.75-0.75)
1970-1984	40-45	Schizoaffective disorders (inpatient)	363	0.84 (0.83-0.84)	152	0.70 (0.70-0.70)	211	0.97 (0.97-0.97)
1985-1994	40-45	Schizoaffective disorders (inpatient)	215	0.58 (0.58-0.58)	79	0.42 (0.42-0.43)	136	0.74 (0.74-0.74)
1995-2004	40-45	Schizoaffective disorders (inpatient)	233	0.69 (0.69-0.69)	95	0.55 (0.55-0.55)	138	0.83 (0.83-0.83)
2005-2016	40-45	Schizoaffective disorders (inpatient)	162	0.39 (0.39-0.39)	67	0.32 (0.32-0.32)	95	0.46 (0.46-0.46)
1970-2016	45-50	Schizoaffective disorders (inpatient)	910	0.60 (0.60-0.60)	323	0.42 (0.42-0.42)	587	0.78 (0.78-0.78)
1970-1984	45-50	Schizoaffective disorders (inpatient)	368	0.88 (0.87-0.88)	130	0.62 (0.62-0.63)	238	1.12 (1.12-1.12)
1985-1994	45-50	Schizoaffective disorders (inpatient)	199	0.59 (0.59-0.59)	60	0.35 (0.35-0.35)	139	0.82 (0.82-0.83)
1995-2004	45-50	Schizoaffective disorders (inpatient)	202	0.59 (0.59-0.59)	69	0.40 (0.40-0.40)	133	0.79 (0.79-0.79)
2005-2016	45-50	Schizoaffective disorders (inpatient)	141	0.34 (0.34-0.34)	64	0.30 (0.30-0.30)	77	0.37 (0.37-0.37)
1970-2016	50-55	Schizoaffective disorders (inpatient)	851	0.59 (0.59-0.59)	270	0.37 (0.37-0.37)	581	0.79 (0.79-0.80)
1970-1984	50-55	Schizoaffective disorders (inpatient)	368	0.87 (0.87-0.87)	112	0.54 (0.54-0.54)	256	1.19 (1.19-1.19)
1985-1994	50-55	Schizoaffective disorders (inpatient)	168	0.60 (0.60-0.60)	46	0.33 (0.33-0.33)	122	0.86 (0.86-0.87)
1995-2004	50-55	Schizoaffective disorders (inpatient)	160	0.45 (0.45-0.45)	54	0.31 (0.31-0.31)	106	0.60 (0.60-0.60)
2005-2016	50-55	Schizoaffective disorders (inpatient)	155	0.39 (0.39-0.39)	58	0.29 (0.29-0.29)	97	0.49 (0.49-0.49)
1970-2016	55-60	Schizoaffective disorders (inpatient)	656	0.48 (0.48-0.48)	206	0.30 (0.30-0.30)	450	0.64 (0.64-0.64)
1970-1984	55-60	Schizoaffective disorders (inpatient)	329	0.79 (0.79-0.79)	94	0.46 (0.46-0.46)	235	1.09 (1.09-1.10)
1985-1994	55-60	Schizoaffective disorders (inpatient)	117	0.46 (0.46-0.46)	37	0.30 (0.30-0.30)	80	0.62 (0.61-0.62)
1995-2004	55-60	Schizoaffective disorders (inpatient)	117	0.37 (0.37-0.37)	35	0.22 (0.22-0.22)	82	0.51 (0.51-0.51)
2005-2016	55-60	Schizoaffective disorders (inpatient)	93	0.24 (0.24-0.24)	40	0.21 (0.21-0.21)	53	0.27 (0.27-0.27)
1970-2016	60-65	Schizoaffective disorders (inpatient)	553	0.43 (0.43-0.43)	165	0.27 (0.27-0.27)	388	0.58 (0.58-0.58)
1970-1984	60-65	Schizoaffective disorders (inpatient)	276	0.69 (0.69-0.69)	78	0.41 (0.41-0.41)	198	0.95 (0.95-0.96)
1985-1994	60-65	Schizoaffective disorders (inpatient)	130	0.53 (0.53-0.53)	35	0.30 (0.30-0.30)	95	0.74 (0.74-0.74)
1995-2004	60-65	Schizoaffective disorders (inpatient)	80	0.31 (0.31-0.31)	25	0.20 (0.20-0.20)	55	0.42 (0.42-0.42)
2005-2016	60-65	Schizoaffective disorders (inpatient)	67	0.17 (0.17-0.17)	27	0.14 (0.14-0.14)	40	0.20 (0.20-0.20)
1970-2016	65-70	Schizoaffective disorders (inpatient)	440	0.38 (0.38-0.38)	115	0.21 (0.21-0.21)	325	0.53 (0.53-0.53)
1970-1984	65-70	Schizoaffective disorders (inpatient)	203	0.58 (0.57-0.58)	52	0.32 (0.32-0.32)	151	0.80 (0.79-0.80)
1985-1994	65-70	Schizoaffective disorders (inpatient)	122	0.52 (0.52-0.52)	29	0.27 (0.27-0.27)	93	0.73 (0.73-0.73)
1995-2004	65-70	Schizoaffective disorders (inpatient)	64	0.29 (0.29-0.29)	18	0.18 (0.17-0.18)	46	0.40 (0.40-0.40)

			Both sexes		Males		Females	
Years	Age	Mental disorder	Cases	IR (95% CI)	Cases	IR (95% CI)	Cases	IR (95% CI)
2005-2016	65-70	Schizoaffective disorders (inpatient)	51	0.15 (0.15-0.15)	16	0.10 (0.10-0.10)	35	0.20 (0.20-0.20)
1970-2016	70-75	Schizoaffective disorders (inpatient)	300	0.31 (0.31-0.32)	71	0.17 (0.17-0.17)	229	0.44 (0.44-0.44)
1970-1984	70-75	Schizoaffective disorders (inpatient)	142	0.49 (0.49-0.49)	32	0.25 (0.25-0.25)	110	0.68 (0.68-0.68)
1985-1994	70-75	Schizoaffective disorders (inpatient)	93	0.45 (0.45-0.45)	24	0.27 (0.27-0.27)	69	0.60 (0.60-0.60)
1995-2004	70-75	Schizoaffective disorders (inpatient)	34	0.18 (0.18-0.18)	8	0.09 (0.09-0.09)	26	0.24 (0.24-0.24)
2005-2016	70-75	Schizoaffective disorders (inpatient)	31	0.12 (0.12-0.12)	7	0.06 (0.06-0.06)	24	0.17 (0.17-0.17)
1970-2016	75-80	Schizoaffective disorders (inpatient)	182	0.25 (0.25-0.25)	40	0.13 (0.13-0.13)	142	0.34 (0.34-0.34)
1970-1984	75-80	Schizoaffective disorders (inpatient)	69	0.34 (0.34-0.34)	14	0.17 (0.17-0.17)	55	0.45 (0.45-0.45)
1985-1994	75-80	Schizoaffective disorders (inpatient)	75	0.46 (0.46-0.46)	20	0.30 (0.30-0.30)	55	0.57 (0.56-0.57)
1995-2004	75-80	Schizoaffective disorders (inpatient)	25	0.15 (0.15-0.15)	0-4	NA	21-25	0.23 (0.23-0.23)
2005-2016	75-80	Schizoaffective disorders (inpatient)	13	0.07 (0.07-0.07)	0-4	NA	9-13	0.09 (0.09-0.09)
1970-2016	80-85	Schizoaffective disorders (inpatient)	88	0.18 (0.18-0.18)	15	0.08 (0.08-0.08)	73	0.24 (0.24-0.24)
1970-1984	80-85	Schizoaffective disorders (inpatient)	35	0.29 (0.29-0.29)	5	0.11 (0.11-0.11)	30	0.40 (0.40-0.40)
1985-1994	80-85	Schizoaffective disorders (inpatient)	33	0.30 (0.30-0.30)	5	0.13 (0.13-0.13)	28	0.40 (0.40-0.40)
1995-2004	80-85	Schizoaffective disorders (inpatient)	14	0.12 (0.12-0.12)	0-4	NA	10-14	0.14 (0.14-0.14)
2005-2016	80-85	Schizoaffective disorders (inpatient)	6	0.04 (0.04-0.04)	0-4	NA	2-6	0.06 (0.06-0.06)
1970-2016	85-90	Schizoaffective disorders (inpatient)	29	0.11 (0.11-0.11)	6	0.07 (0.07-0.07)	23	0.13 (0.13-0.13)
1970-1984	85-90	Schizoaffective disorders (inpatient)	8	0.14 (0.14-0.15)	0-4	NA	4-8	0.17 (0.17-0.17)
1985-1994	85-90	Schizoaffective disorders (inpatient)	16	0.29 (0.29-0.29)	0-4	NA	12-16	0.34 (0.34-0.34)
1995-2004	85-90	Schizoaffective disorders (inpatient)	0-4	NA	0-4	NA	0-4	NA
2005-2016	85-90	Schizoaffective disorders (inpatient)	0-4	NA	0-4	NA	0-4	NA
1970-2016	90-95	Schizoaffective disorders (inpatient)	0-4	NA	0-4	NA	0-4	NA
1970-1984	90-95	Schizoaffective disorders (inpatient)	0-4	NA	0-4	NA	0-4	NA
1985-1994	90-95	Schizoaffective disorders (inpatient)	0-4	NA	0-4	NA	0-4	NA
1995-2004	90-95	Schizoaffective disorders (inpatient)	0-4	NA	0-4	NA	0-4	NA
2005-2016	90-95	Schizoaffective disorders (inpatient)	0-4	NA	0-4	NA	0-4	NA
1970-2016	95-100	Schizoaffective disorders (inpatient)	0-4	NA	0-4	NA	0-4	NA
1970-1984	95-100	Schizoaffective disorders (inpatient)	0-4	NA	0-4	NA	0-4	NA
1985-1994	95-100	Schizoaffective disorders (inpatient)	0-4	NA	0-4	NA	0-4	NA
1995-2004	95-100	Schizoaffective disorders (inpatient)	0-4	NA	0-4	NA	0-4	NA
2005-2016	95-100	Schizoaffective disorders (inpatient)	0-4	NA	0-4	NA	0-4	NA
1970-2016	10-15	Mood disorders	4,612	2.92 (2.91-2.92)	1,696	2.09 (2.09-2.10)	2,916	3.78 (3.77-3.78)
1970-1984	10-15	Mood disorders	77	0.14 (0.14-0.14)	35	0.12 (0.12-0.12)	42	0.15 (0.15-0.15)
1985-1994	10-15	Mood disorders	29	0.09 (0.09-0.09)	11	0.07 (0.07-0.07)	18	0.12 (0.12-0.12)
1995-2004	10-15	Mood disorders	792	2.68 (2.68-2.68)	313	2.07 (2.06-2.07)	479	3.32 (3.32-3.33)
2005-2016	10-15	Mood disorders	3,714	9.27 (9.26-9.27)	1,337	6.51 (6.50-6.52)	2,377	12.16 (12.14-12.18)
1970-2016	15-20	Mood disorders	19,340	12.07 (12.06-12.08)	5,658	6.87 (6.87-6.88)	13,682	17.56 (17.55-17.57)
1970-1984	15-20	Mood disorders	1,318	2.31 (2.31-2.31)	511	1.74 (1.74-1.75)	807	2.91 (2.90-2.91)
1985-1994	15-20	Mood disorders	366	1.04 (1.04-1.04)	150	0.83 (0.83-0.83)	216	1.26 (1.25-1.26)
1995-2004	15-20	Mood disorders	3,392	11.94 (11.93-11.96)	947	6.51 (6.50-6.52)	2,445	17.66 (17.63-17.69)

			Both sexes		Males		Females	
**Years**	**Age**	**Mental disorder**	**Cases**	**IR (95% CI)**	**Cases**	**IR (95% CI)**	**Cases**	**IR (95% CI)**
2005-2016	15-20	Mood disorders	14,264	36.15 (36.11-36.18)	4,050	19.90 (19.87-19.92)	10,214	53.47 (53.39-53.54)
1970-2016	20-25	Mood disorders	23,994	14.98 (14.97-14.98)	8,720	10.50 (10.49-10.51)	15,274	19.79 (19.77-19.80)
1970-1984	20-25	Mood disorders	3,372	6.09 (6.08-6.09)	1,408	4.92 (4.91-4.93)	1,964	7.34 (7.33-7.35)
1985-1994	20-25	Mood disorders	854	2.29 (2.29-2.29)	380	1.96 (1.96-1.96)	474	2.65 (2.65-2.66)
1995-2004	20-25	Mood disorders	5,078	16.46 (16.44-16.47)	1,675	10.48 (10.46-10.50)	3,403	22.88 (22.84-22.91)
2005-2016	20-25	Mood disorders	14,690	40.03 (39.99-40.07)	5,257	27.62 (27.58-27.66)	9,433	53.40 (53.32-53.48)
1970-2016	25-30	Mood disorders	23,045	14.36 (14.35-14.37)	8,888	10.72 (10.71-10.72)	14,157	18.25 (18.24-18.27)
1970-1984	25-30	Mood disorders	4,854	8.60 (8.59-8.60)	1,954	6.73 (6.72-6.73)	2,900	10.58 (10.57-10.59)
1985-1994	25-30	Mood disorders	1,175	3.19 (3.19-3.20)	475	2.49 (2.48-2.49)	700	3.95 (3.94-3.96)
1995-2004	25-30	Mood disorders	5,525	16.50 (16.48-16.51)	2,093	12.06 (12.04-12.07)	3,432	21.27 (21.24-21.30)
2005-2016	25-30	Mood disorders	11,491	34.07 (34.04-34.11)	4,366	25.05 (25.01-25.08)	7,125	43.73 (43.66-43.80)
1970-2016	30-35	Mood disorders	23,813	14.99 (14.99-15.00)	9,421	11.54 (11.53-11.55)	14,392	18.65 (18.63-18.66)
1970-1984	30-35	Mood disorders	5,789	10.73 (10.72-10.74)	2,277	8.26 (8.25-8.27)	3,512	13.30 (13.28-13.32)
1985-1994	30-35	Mood disorders	1,531	4.44 (4.44-4.45)	609	3.43 (3.42-3.43)	922	5.52 (5.51-5.53)
1995-2004	30-35	Mood disorders	5,837	16.31 (16.29-16.33)	2,332	12.61 (12.59-12.63)	3,505	20.26 (20.23-20.29)
2005-2016	30-35	Mood disorders	10,656	30.79 (30.76-30.82)	4,203	23.56 (23.52-23.59)	6,453	38.49 (38.43-38.55)
1970-2016	35-40	Mood disorders	24,708	15.75 (15.74-15.76)	9,794	12.22 (12.21-12.23)	14,914	19.44 (19.42-19.45)
1970-1984	35-40	Mood disorders	6,259	12.77 (12.75-12.78)	2,316	9.31 (9.30-9.32)	3,943	16.32 (16.30-16.34)
1985-1994	35-40	Mood disorders	1,843	5.31 (5.31-5.32)	716	4.05 (4.04-4.06)	1,127	6.63 (6.62-6.64)
1995-2004	35-40	Mood disorders	6,318	17.73 (17.71-17.75)	2,681	14.66 (14.64-14.69)	3,637	20.96 (20.93-20.99)
2005-2016	35-40	Mood disorders	10,288	27.41 (27.38-27.43)	4,081	21.13 (21.10-21.16)	6,207	34.06 (34.01-34.11)
1970-2016	40-45	Mood disorders	24,509	16.00 (15.99-16.01)	10,058	12.94 (12.93-12.94)	14,451	19.15 (19.14-19.17)
1970-1984	40-45	Mood disorders	6,469	15.07 (15.06-15.08)	2,382	11.05 (11.04-11.07)	4,087	19.12 (19.10-19.15)
1985-1994	40-45	Mood disorders	2,150	5.91 (5.90-5.91)	877	4.76 (4.75-4.77)	1,273	7.08 (7.07-7.09)
1995-2004	40-45	Mood disorders	5,860	17.54 (17.52-17.56)	2,554	14.99 (14.97-15.01)	3,306	20.19 (20.15-20.22)
2005-2016	40-45	Mood disorders	10,030	24.79 (24.76-24.81)	4,245	20.47 (20.44-20.50)	5,785	29.33 (29.29-29.37)
1970-2016	45-50	Mood disorders	24,485	16.46 (16.46-16.47)	10,011	13.36 (13.35-13.37)	14,474	19.62 (19.60-19.63)
1970-1984	45-50	Mood disorders	7,125	17.17 (17.15-17.19)	2,612	12.66 (12.64-12.68)	4,513	21.63 (21.60-21.66)
1985-1994	45-50	Mood disorders	2,272	6.83 (6.82-6.84)	881	5.25 (5.24-5.26)	1,391	8.43 (8.42-8.44)
1995-2004	45-50	Mood disorders	5,599	16.78 (16.76-16.80)	2,395	14.21 (14.18-14.23)	3,204	19.41 (19.38-19.44)
2005-2016	45-50	Mood disorders	9,489	23.39 (23.37-23.41)	4,123	19.97 (19.94-19.99)	5,366	26.94 (26.90-26.98)
1970-2016	50-55	Mood disorders	22,766	16.02 (16.01-16.03)	9,249	13.02 (13.01-13.03)	13,517	19.02 (19.00-19.03)
1970-1984	50-55	Mood disorders	7,526	18.04 (18.02-18.06)	2,648	12.86 (12.84-12.87)	4,878	23.10 (23.07-23.13)
1985-1994	50-55	Mood disorders	2,055	7.49 (7.48-7.50)	765	5.59 (5.58-5.60)	1,290	9.38 (9.37-9.40)
1995-2004	50-55	Mood disorders	5,418	15.67 (15.66-15.69)	2,307	13.30 (13.28-13.32)	3,111	18.06 (18.04-18.09)
2005-2016	50-55	Mood disorders	7,767	20.23 (20.21-20.25)	3,529	18.19 (18.16-18.21)	4,238	22.32 (22.29-22.35)
1970-2016	55-60	Mood disorders	19,664	14.65 (14.64-14.66)	7,972	12.01 (12.00-12.02)	11,692	17.24 (17.23-17.26)
1970-1984	55-60	Mood disorders	6,921	16.79 (16.78-16.81)	2,407	11.94 (11.93-11.96)	4,514	21.44 (21.41-21.47)
1985-1994	55-60	Mood disorders	1,998	8.06 (8.05-8.07)	728	5.97 (5.96-5.98)	1,270	10.08 (10.06-10.09)
1995-2004	55-60	Mood disorders	4,518	14.56 (14.55-14.58)	1,920	12.41 (12.39-12.43)	2,598	16.70 (16.67-16.72)

			Both sexes		Males		Females	
**Years**	**Age**	**Mental disorder**	**Cases**	**IR (95% CI)**	**Cases**	**IR (95% CI)**	**Cases**	**IR (95% CI)**
2005-2016	55-60	Mood disorders	6,227	16.75 (16.73-16.76)	2,917	15.69 (15.66-15.71)	3,310	17.81 (17.78-17.83)
1970-2016	60-65	Mood disorders	15,783	12.59 (12.58-12.60)	6,083	9.97 (9.97-9.98)	9,700	15.07 (15.06-15.08)
1970-1984	60-65	Mood disorders	6,005	15.30 (15.28-15.31)	1,985	10.52 (10.50-10.53)	4,020	19.72 (19.69-19.74)
1985-1994	60-65	Mood disorders	2,004	8.35 (8.34-8.36)	688	5.96 (5.95-5.97)	1,316	10.56 (10.54-10.58)
1995-2004	60-65	Mood disorders	3,262	13.16 (13.15-13.18)	1,290	10.64 (10.62-10.66)	1,972	15.58 (15.56-15.61)
2005-2016	60-65	Mood disorders	4,512	12.09 (12.08-12.11)	2,120	11.49 (11.48-11.51)	2,392	12.68 (12.66-12.70)
1970-2016	65-70	Mood disorders	13,870	12.35 (12.34-12.35)	4,849	9.12 (9.11-9.13)	9,021	15.25 (15.23-15.26)
1970-1984	65-70	Mood disorders	4,877	14.00 (13.98-14.01)	1,491	9.22 (9.20-9.23)	3,386	18.14 (18.12-18.17)
1985-1994	65-70	Mood disorders	1,980	8.63 (8.62-8.64)	618	5.78 (5.77-5.79)	1,362	11.11 (11.09-11.13)
1995-2004	65-70	Mood disorders	3,221	15.21 (15.19-15.23)	1,164	11.56 (11.54-11.58)	2,057	18.51 (18.48-18.55)
2005-2016	65-70	Mood disorders	3,792	11.37 (11.35-11.38)	1,576	9.71 (9.70-9.73)	2,216	12.93 (12.91-12.95)
1970-2016	70-75	Mood disorders	12,889	13.88 (13.87-13.88)	4,212	10.03 (10.02-10.04)	8,677	17.05 (17.03-17.06)
1970-1984	70-75	Mood disorders	3,621	12.73 (12.71-12.74)	964	7.72 (7.71-7.74)	2,657	16.64 (16.61-16.66)
1985-1994	70-75	Mood disorders	1,822	9.10 (9.09-9.11)	493	5.56 (5.54-5.57)	1,329	11.93 (11.90-11.95)
1995-2004	70-75	Mood disorders	3,798	20.28 (20.25-20.31)	1,287	15.16 (15.13-15.19)	2,511	24.52 (24.47-24.56)
2005-2016	70-75	Mood disorders	3,648	14.20 (14.19-14.22)	1,468	12.09 (12.07-12.11)	2,180	16.09 (16.07-16.12)
1970-2016	75-80	Mood disorders	11,971	16.91 (16.90-16.92)	3,693	12.38 (12.36-12.39)	8,278	20.21 (20.19-20.23)
1970-1984	75-80	Mood disorders	2,226	11.00 (10.98-11.01)	611	7.40 (7.38-7.42)	1,615	13.48 (13.45-13.50)
1985-1994	75-80	Mood disorders	1,554	9.73 (9.72-9.75)	410	6.29 (6.28-6.31)	1,144	12.10 (12.08-12.13)
1995-2004	75-80	Mood disorders	4,437	28.39 (28.35-28.43)	1,354	20.68 (20.63-20.73)	3,083	33.95 (33.88-34.02)
2005-2016	75-80	Mood disorders	3,754	19.80 (19.77-19.82)	1,318	15.48 (15.45-15.51)	2,436	23.31 (23.27-23.36)
1970-2016	80-85	Mood disorders	9,192	19.43 (19.41-19.44)	2,732	15.13 (15.11-15.15)	6,460	22.07 (22.05-22.10)
1970-1984	80-85	Mood disorders	916	7.58 (7.56-7.59)	246	5.33 (5.32-5.35)	670	8.96 (8.94-8.98)
1985-1994	80-85	Mood disorders	944	8.83 (8.81-8.84)	226	5.91 (5.89-5.93)	718	10.45 (10.43-10.48)
1995-2004	80-85	Mood disorders	3,831	34.67 (34.61-34.74)	1,111	27.02 (26.93-27.10)	2,720	39.21 (39.12-39.30)
2005-2016	80-85	Mood disorders	3,501	25.96 (25.92-26.01)	1,149	20.88 (20.83-20.94)	2,352	29.46 (29.40-29.53)
1970-2016	85-90	Mood disorders	5,342	21.09 (21.07-21.12)	1,380	16.30 (16.27-16.34)	3,962	23.50 (23.46-23.54)
1970-1984	85-90	Mood disorders	268	4.88 (4.86-4.89)	85	4.28 (4.26-4.30)	183	5.21 (5.19-5.23)
1985-1994	85-90	Mood disorders	347	6.44 (6.42-6.46)	63	3.78 (3.76-3.80)	284	7.64 (7.61-7.66)
1995-2004	85-90	Mood disorders	2,359	37.67 (37.57-37.76)	574	29.12 (28.99-29.25)	1,785	41.59 (41.47-41.72)
2005-2016	85-90	Mood disorders	2,368	28.96 (28.89-29.02)	658	23.17 (23.08-23.25)	1,710	32.04 (31.95-32.12)
1970-2016	90-95	Mood disorders	1,780	19.20 (19.16-19.24)	391	15.01 (14.95-15.07)	1,389	20.84 (20.79-20.89)
1970-1984	90-95	Mood disorders	32	2.01 (2.00-2.02)	8	1.45 (1.44-1.47)	24	2.30 (2.28-2.31)
1985-1994	90-95	Mood disorders	68	3.69 (3.68-3.71)	15	2.97 (2.94-3.00)	53	3.97 (3.95-3.99)
1995-2004	90-95	Mood disorders	781	32.65 (32.52-32.78)	161	26.76 (26.55-26.98)	620	34.63 (34.47-34.79)
2005-2016	90-95	Mood disorders	899	26.11 (26.02-26.19)	207	21.84 (21.70-21.98)	692	27.72 (27.62-27.83)
1970-2016	95-100	Mood disorders	298	15.39 (15.32-15.45)	58	13.21 (13.09-13.34)	240	16.02 (15.94-16.10)
1970-1984	95-100	Mood disorders	0-4	NA	0-4	NA	0-4	NA
1985-1994	95-100	Mood disorders	0-4	NA	0-4	NA	0-4	NA
1995-2004	95-100	Mood disorders	116	23.54 (23.33-23.75)	23	23.43 (22.97-23.89)	93	23.57 (23.34-23.80)

			Both sexes		Males		Females	
**Years**	**Age**	**Mental disorder**	**Cases**	**IR (95% CI)**	**Cases**	**IR (95% CI)**	**Cases**	**IR (95% CI)**
2005-2016	95-100	Mood disorders	177	21.02 (20.88-21.17)	34	19.62 (19.33-19.91)	143	21.39 (21.23-21.55)
1970-2016	10-15	Mood disorders (inpatient)	902	0.57 (0.57-0.57)	280	0.35 (0.35-0.35)	622	0.81 (0.80-0.81)
1970-1984	10-15	Mood disorders (inpatient)	77	0.14 (0.14-0.14)	35	0.12 (0.12-0.12)	42	0.15 (0.15-0.15)
1985-1994	10-15	Mood disorders (inpatient)	26	0.08 (0.08-0.08)	11	0.07 (0.07-0.07)	15	0.10 (0.10-0.10)
1995-2004	10-15	Mood disorders (inpatient)	162	0.55 (0.55-0.55)	56	0.37 (0.37-0.37)	106	0.74 (0.73-0.74)
2005-2016	10-15	Mood disorders (inpatient)	637	1.59 (1.59-1.59)	178	0.87 (0.86-0.87)	459	2.35 (2.34-2.35)
1970-2016	15-20	Mood disorders (inpatient)	5,896	3.67 (3.67-3.67)	1,887	2.29 (2.29-2.29)	4,009	5.13 (5.12-5.13)
1970-1984	15-20	Mood disorders (inpatient)	1,318	2.31 (2.31-2.31)	511	1.74 (1.74-1.75)	807	2.91 (2.90-2.91)
1985-1994	15-20	Mood disorders (inpatient)	354	1.00 (1.00-1.00)	146	0.81 (0.80-0.81)	208	1.21 (1.21-1.21)
1995-2004	15-20	Mood disorders (inpatient)	929	3.27 (3.26-3.27)	274	1.88 (1.88-1.88)	655	4.72 (4.71-4.73)
2005-2016	15-20	Mood disorders (inpatient)	3,295	8.28 (8.27-8.29)	956	4.67 (4.67-4.68)	2,339	12.10 (12.08-12.11)
1970-2016	20-25	Mood disorders (inpatient)	9,911	6.16 (6.15-6.16)	3,828	4.60 (4.59-4.60)	6,083	7.83 (7.82-7.83)
1970-1984	20-25	Mood disorders (inpatient)	3,372	6.09 (6.08-6.09)	1,408	4.92 (4.91-4.93)	1,964	7.34 (7.33-7.35)
1985-1994	20-25	Mood disorders (inpatient)	811	2.18 (2.17-2.18)	358	1.84 (1.84-1.85)	453	2.53 (2.53-2.54)
1995-2004	20-25	Mood disorders (inpatient)	1,716	5.54 (5.54-5.55)	602	3.76 (3.75-3.76)	1,114	7.45 (7.44-7.47)
2005-2016	20-25	Mood disorders (inpatient)	4,012	10.74 (10.73-10.75)	1,460	7.59 (7.58-7.60)	2,552	14.08 (14.06-14.11)
1970-2016	25-30	Mood disorders (inpatient)	11,399	7.06 (7.06-7.07)	4,556	5.47 (5.47-5.48)	6,843	8.76 (8.75-8.76)
1970-1984	25-30	Mood disorders (inpatient)	4,854	8.60 (8.59-8.60)	1,954	6.73 (6.72-6.73)	2,900	10.58 (10.57-10.59)
1985-1994	25-30	Mood disorders (inpatient)	1,109	3.01 (3.01-3.02)	458	2.40 (2.40-2.40)	651	3.67 (3.67-3.68)
1995-2004	25-30	Mood disorders (inpatient)	2,050	6.10 (6.09-6.10)	800	4.60 (4.59-4.60)	1,250	7.71 (7.70-7.72)
2005-2016	25-30	Mood disorders (inpatient)	3,386	9.82 (9.81-9.83)	1,344	7.60 (7.59-7.61)	2,042	12.16 (12.14-12.18)
1970-2016	30-35	Mood disorders (inpatient)	13,217	8.28 (8.27-8.28)	5,369	6.55 (6.55-6.56)	7,848	10.09 (10.09-10.10)
1970-1984	30-35	Mood disorders (inpatient)	5,789	10.73 (10.72-10.74)	2,277	8.26 (8.25-8.27)	3,512	13.30 (13.28-13.32)
1985-1994	30-35	Mood disorders (inpatient)	1,460	4.24 (4.23-4.24)	585	3.29 (3.29-3.30)	875	5.24 (5.23-5.24)
1995-2004	30-35	Mood disorders (inpatient)	2,518	7.01 (7.00-7.02)	1,054	5.68 (5.68-5.69)	1,464	8.42 (8.41-8.43)
2005-2016	30-35	Mood disorders (inpatient)	3,450	9.76 (9.75-9.77)	1,453	8.03 (8.02-8.04)	1,997	11.57 (11.55-11.59)
1970-2016	35-40	Mood disorders (inpatient)	14,411	9.13 (9.13-9.14)	5,796	7.20 (7.20-7.21)	8,615	11.14 (11.14-11.15)
1970-1984	35-40	Mood disorders (inpatient)	6,259	12.77 (12.75-12.78)	2,316	9.31 (9.30-9.32)	3,943	16.32 (16.30-16.34)
1985-1994	35-40	Mood disorders (inpatient)	1,769	5.10 (5.10-5.11)	690	3.90 (3.90-3.91)	1,079	6.35 (6.34-6.36)
1995-2004	35-40	Mood disorders (inpatient)	2,874	8.03 (8.02-8.04)	1,262	6.88 (6.87-6.89)	1,612	9.24 (9.23-9.26)
2005-2016	35-40	Mood disorders (inpatient)	3,509	9.16 (9.15-9.17)	1,528	7.80 (7.79-7.81)	1,981	10.59 (10.57-10.60)
1970-2016	40-45	Mood disorders (inpatient)	15,100	9.80 (9.79-9.80)	6,209	7.95 (7.95-7.96)	8,891	11.70 (11.69-11.71)
1970-1984	40-45	Mood disorders (inpatient)	6,469	15.07 (15.06-15.08)	2,382	11.05 (11.04-11.07)	4,087	19.12 (19.10-19.15)
1985-1994	40-45	Mood disorders (inpatient)	2,038	5.60 (5.59-5.60)	832	4.51 (4.51-4.52)	1,206	6.71 (6.70-6.72)
1995-2004	40-45	Mood disorders (inpatient)	2,821	8.41 (8.40-8.42)	1,261	7.38 (7.37-7.39)	1,560	9.48 (9.46-9.49)
2005-2016	40-45	Mood disorders (inpatient)	3,772	9.15 (9.14-9.16)	1,734	8.25 (8.24-8.26)	2,038	10.09 (10.08-10.11)
1970-2016	45-50	Mood disorders (inpatient)	15,824	10.58 (10.58-10.59)	6,422	8.53 (8.53-8.54)	9,402	12.65 (12.65-12.66)
1970-1984	45-50	Mood disorders (inpatient)	7,125	17.17 (17.15-17.19)	2,612	12.66 (12.64-12.68)	4,513	21.63 (21.60-21.66)
1985-1994	45-50	Mood disorders (inpatient)	2,154	6.47 (6.47-6.48)	841	5.01 (5.00-5.02)	1,313	7.96 (7.95-7.97)
1995-2004	45-50	Mood disorders (inpatient)	2,795	8.34 (8.33-8.35)	1,194	7.06 (7.05-7.07)	1,601	9.65 (9.64-9.67)

			Both sexes		Males		Females	
Years	Age	Mental disorder	Cases	IR (95% CI)	Cases	IR (95% CI)	Cases	IR (95% CI)
2005-2016	45-50	Mood disorders (inpatient)	3,750	9.09 (9.08-9.09)	1,775	8.48 (8.47-8.49)	1,975	9.71 (9.69-9.72)
1970-2016	50-55	Mood disorders (inpatient)	15,699	10.99 (10.98-10.99)	6,210	8.70 (8.70-8.71)	9,489	13.27 (13.26-13.28)
1970-1984	50-55	Mood disorders (inpatient)	7,526	18.04 (18.02-18.06)	2,648	12.86 (12.84-12.87)	4,878	23.10 (23.07-23.13)
1985-1994	50-55	Mood disorders (inpatient)	1,976	7.20 (7.19-7.21)	736	5.38 (5.37-5.39)	1,240	9.02 (9.00-9.03)
1995-2004	50-55	Mood disorders (inpatient)	2,873	8.28 (8.27-8.29)	1,205	6.92 (6.91-6.93)	1,668	9.64 (9.63-9.66)
2005-2016	50-55	Mood disorders (inpatient)	3,324	8.52 (8.51-8.53)	1,621	8.25 (8.24-8.26)	1,703	8.80 (8.79-8.81)
1970-2016	55-60	Mood disorders (inpatient)	14,149	10.49 (10.49-10.50)	5,538	8.31 (8.30-8.31)	8,611	12.63 (12.62-12.64)
1970-1984	55-60	Mood disorders (inpatient)	6,921	16.79 (16.78-16.81)	2,407	11.94 (11.93-11.96)	4,514	21.44 (21.41-21.47)
1985-1994	55-60	Mood disorders (inpatient)	1,928	7.78 (7.77-7.79)	699	5.74 (5.73-5.75)	1,229	9.75 (9.73-9.77)
1995-2004	55-60	Mood disorders (inpatient)	2,433	7.81 (7.80-7.82)	1,043	6.72 (6.71-6.73)	1,390	8.89 (8.88-8.91)
2005-2016	55-60	Mood disorders (inpatient)	2,867	7.61 (7.60-7.61)	1,389	7.39 (7.38-7.40)	1,478	7.83 (7.81-7.84)
1970-2016	60-65	Mood disorders (inpatient)	12,176	9.68 (9.67-9.68)	4,558	7.45 (7.44-7.45)	7,618	11.78 (11.77-11.79)
1970-1984	60-65	Mood disorders (inpatient)	6,005	15.30 (15.28-15.31)	1,985	10.52 (10.50-10.53)	4,020	19.72 (19.69-19.74)
1985-1994	60-65	Mood disorders (inpatient)	1,956	8.15 (8.14-8.16)	676	5.85 (5.84-5.86)	1,280	10.27 (10.26-10.29)
1995-2004	60-65	Mood disorders (inpatient)	1,790	7.20 (7.19-7.21)	721	5.93 (5.92-5.94)	1,069	8.41 (8.40-8.43)
2005-2016	60-65	Mood disorders (inpatient)	2,425	6.43 (6.42-6.44)	1,176	6.32 (6.31-6.33)	1,249	6.54 (6.53-6.55)
1970-2016	65-70	Mood disorders (inpatient)	10,517	9.33 (9.33-9.34)	3,576	6.71 (6.70-6.71)	6,941	11.69 (11.68-11.69)
1970-1984	65-70	Mood disorders (inpatient)	4,877	14.00 (13.98-14.01)	1,491	9.22 (9.20-9.23)	3,386	18.14 (18.12-18.17)
1985-1994	65-70	Mood disorders (inpatient)	1,907	8.31 (8.30-8.32)	597	5.59 (5.58-5.60)	1,310	10.68 (10.66-10.70)
1995-2004	65-70	Mood disorders (inpatient)	1,707	8.04 (8.02-8.05)	606	6.01 (5.99-6.02)	1,101	9.87 (9.85-9.89)
2005-2016	65-70	Mood disorders (inpatient)	2,026	6.02 (6.01-6.02)	882	5.39 (5.39-5.40)	1,144	6.61 (6.60-6.62)
1970-2016	70-75	Mood disorders (inpatient)	8,886	9.54 (9.53-9.54)	2,755	6.55 (6.54-6.55)	6,131	12.00 (11.99-12.01)
1970-1984	70-75	Mood disorders (inpatient)	3,621	12.73 (12.71-12.74)	964	7.72 (7.71-7.74)	2,657	16.64 (16.61-16.66)
1985-1994	70-75	Mood disorders (inpatient)	1,730	8.64 (8.63-8.65)	470	5.30 (5.29-5.31)	1,260	11.31 (11.29-11.33)
1995-2004	70-75	Mood disorders (inpatient)	1,768	9.40 (9.39-9.42)	590	6.93 (6.92-6.95)	1,178	11.44 (11.42-11.47)
2005-2016	70-75	Mood disorders (inpatient)	1,767	6.82 (6.81-6.83)	731	5.98 (5.97-5.99)	1,036	7.57 (7.56-7.58)
1970-2016	75-80	Mood disorders (inpatient)	7,012	9.86 (9.86-9.87)	2,145	7.17 (7.16-7.18)	4,867	11.82 (11.81-11.83)
1970-1984	75-80	Mood disorders (inpatient)	2,226	11.00 (10.98-11.01)	611	7.40 (7.38-7.42)	1,615	13.48 (13.45-13.50)
1985-1994	75-80	Mood disorders (inpatient)	1,477	9.25 (9.23-9.26)	393	6.03 (6.02-6.05)	1,084	11.47 (11.44-11.49)
1995-2004	75-80	Mood disorders (inpatient)	1,811	11.52 (11.50-11.54)	570	8.67 (8.65-8.69)	1,241	13.57 (13.54-13.59)
2005-2016	75-80	Mood disorders (inpatient)	1,498	7.82 (7.81-7.83)	571	6.66 (6.65-6.67)	927	8.76 (8.74-8.78)
1970-2016	80-85	Mood disorders (inpatient)	4,236	8.90 (8.89-8.91)	1,252	6.91 (6.90-6.92)	2,984	10.12 (10.11-10.14)
1970-1984	80-85	Mood disorders (inpatient)	916	7.58 (7.56-7.59)	246	5.33 (5.32-5.35)	670	8.96 (8.94-8.98)
1985-1994	80-85	Mood disorders (inpatient)	878	8.21 (8.19-8.23)	212	5.54 (5.53-5.56)	666	9.69 (9.67-9.72)
1995-2004	80-85	Mood disorders (inpatient)	1,316	11.81 (11.79-11.83)	413	9.99 (9.96-10.02)	903	12.89 (12.86-12.92)
2005-2016	80-85	Mood disorders (inpatient)	1,126	8.24 (8.22-8.25)	381	6.86 (6.84-6.88)	745	9.18 (9.16-9.20)
1970-2016	85-90	Mood disorders (inpatient)	1,899	7.44 (7.43-7.44)	530	6.23 (6.21-6.24)	1,369	8.04 (8.03-8.05)
1970-1984	85-90	Mood disorders (inpatient)	268	4.88 (4.86-4.89)	85	4.28 (4.26-4.30)	183	5.21 (5.19-5.23)
1985-1994	85-90	Mood disorders (inpatient)	308	5.72 (5.70-5.73)	58	3.48 (3.46-3.50)	250	6.72 (6.70-6.74)
1995-2004	85-90	Mood disorders (inpatient)	645	10.19 (10.17-10.22)	174	8.77 (8.73-8.81)	471	10.84 (10.81-10.87)

			Both sexes		Males		Females	
Years	Age	Mental disorder	Cases	IR (95% CI)	Cases	IR (95% CI)	Cases	IR (95% CI)
2005-2016	85-90	Mood disorders (inpatient)	678	8.14 (8.13-8.16)	213	7.41 (7.39-7.44)	465	8.53 (8.50-8.55)
1970-2016	90-95	Mood disorders (inpatient)	433	4.62 (4.61-4.63)	108	4.12 (4.10-4.14)	325	4.81 (4.80-4.83)
1970-1984	90-95	Mood disorders (inpatient)	32	2.01 (2.00-2.02)	8	1.45 (1.44-1.47)	24	2.30 (2.28-2.31)
1985-1994	90-95	Mood disorders (inpatient)	62	3.37 (3.35-3.38)	15	2.97 (2.94-3.00)	47	3.52 (3.50-3.54)
1995-2004	90-95	Mood disorders (inpatient)	153	6.33 (6.30-6.35)	38	6.27 (6.22-6.32)	115	6.34 (6.31-6.37)
2005-2016	90-95	Mood disorders (inpatient)	186	5.29 (5.27-5.31)	47	4.90 (4.87-4.93)	139	5.43 (5.41-5.46)
1970-2016	95-100	Mood disorders (inpatient)	49	2.50 (2.49-2.51)	14	3.17 (3.14-3.20)	35	2.31 (2.29-2.32)
1970-1984	95-100	Mood disorders (inpatient)	0-4	NA	0-4	NA	0-4	NA
1985-1994	95-100	Mood disorders (inpatient)	0-4	NA	0-4	NA	0-4	NA
1995-2004	95-100	Mood disorders (inpatient)	16	3.22 (3.19-3.24)	6	6.08 (5.96-6.20)	10	2.51 (2.48-2.53)
2005-2016	95-100	Mood disorders (inpatient)	28	3.25 (3.23-3.28)	7	3.98 (3.92-4.04)	21	3.07 (3.04-3.09)
1970-2016	10-15	Bipolar disorder	154	0.10 (0.10-0.10)	70	0.09 (0.09-0.09)	84	0.11 (0.11-0.11)
1970-1984	10-15	Bipolar disorder	20	0.04 (0.04-0.04)	15	0.05 (0.05-0.05)	5	0.02 (0.02-0.02)
1985-1994	10-15	Bipolar disorder	8	0.03 (0.03-0.03)	4-8	0.03 (0.03-0.03)	0-4	NA
1995-2004	10-15	Bipolar disorder	27	0.09 (0.09-0.09)	13	0.09 (0.09-0.09)	14	0.10 (0.10-0.10)
2005-2016	10-15	Bipolar disorder	99	0.25 (0.25-0.25)	37	0.18 (0.18-0.18)	62	0.32 (0.32-0.32)
1970-2016	15-20	Bipolar disorder	1594	0.99 (0.99-0.99)	653	0.79 (0.79-0.79)	941	1.20 (1.20-1.20)
1970-1984	15-20	Bipolar disorder	380	0.67 (0.67-0.67)	180	0.61 (0.61-0.61)	200	0.72 (0.72-0.72)
1985-1994	15-20	Bipolar disorder	161	0.46 (0.46-0.46)	69	0.38 (0.38-0.38)	92	0.53 (0.53-0.54)
1995-2004	15-20	Bipolar disorder	197	0.69 (0.69-0.69)	82	0.56 (0.56-0.56)	115	0.83 (0.83-0.83)
2005-2016	15-20	Bipolar disorder	856	2.15 (2.14-2.15)	322	1.57 (1.57-1.57)	534	2.75 (2.75-2.76)
1970-2016	20-25	Bipolar disorder	3125	1.94 (1.94-1.94)	1297	1.56 (1.55-1.56)	1828	2.35 (2.34-2.35)
1970-1984	20-25	Bipolar disorder	739	1.33 (1.33-1.33)	361	1.26 (1.26-1.26)	378	1.41 (1.41-1.41)
1985-1994	20-25	Bipolar disorder	286	0.77 (0.77-0.77)	126	0.65 (0.65-0.65)	160	0.89 (0.89-0.90)
1995-2004	20-25	Bipolar disorder	389	1.25 (1.25-1.26)	189	1.18 (1.18-1.18)	200	1.34 (1.33-1.34)
2005-2016	20-25	Bipolar disorder	1711	4.56 (4.56-4.57)	621	3.22 (3.22-3.22)	1090	5.98 (5.97-5.99)
1970-2016	25-30	Bipolar disorder	3191	1.97 (1.97-1.97)	1340	1.61 (1.60-1.61)	1851	2.36 (2.36-2.36)
1970-1984	25-30	Bipolar disorder	810	1.43 (1.43-1.43)	369	1.27 (1.26-1.27)	441	1.60 (1.60-1.60)
1985-1994	25-30	Bipolar disorder	328	0.89 (0.89-0.89)	120	0.63 (0.63-0.63)	208	1.17 (1.17-1.17)
1995-2004	25-30	Bipolar disorder	485	1.44 (1.44-1.44)	223	1.28 (1.28-1.28)	262	1.61 (1.61-1.61)
2005-2016	25-30	Bipolar disorder	1568	4.52 (4.52-4.53)	628	3.54 (3.53-3.54)	940	5.55 (5.54-5.56)
1970-2016	30-35	Bipolar disorder	3385	2.11 (2.11-2.11)	1395	1.70 (1.69-1.70)	1990	2.54 (2.54-2.54)
1970-1984	30-35	Bipolar disorder	882	1.62 (1.62-1.63)	362	1.31 (1.31-1.31)	520	1.95 (1.95-1.96)
1985-1994	30-35	Bipolar disorder	359	1.04 (1.04-1.04)	133	0.75 (0.75-0.75)	226	1.34 (1.34-1.35)
1995-2004	30-35	Bipolar disorder	588	1.63 (1.63-1.63)	265	1.43 (1.42-1.43)	323	1.85 (1.85-1.85)
2005-2016	30-35	Bipolar disorder	1556	4.37 (4.36-4.37)	635	3.49 (3.49-3.50)	921	5.28 (5.28-5.29)
1970-2016	35-40	Bipolar disorder	3568	2.24 (2.24-2.24)	1438	1.78 (1.78-1.78)	2130	2.73 (2.73-2.73)
1970-1984	35-40	Bipolar disorder	876	1.77 (1.77-1.77)	334	1.33 (1.33-1.34)	542	2.22 (2.21-2.22)
1985-1994	35-40	Bipolar disorder	447	1.28 (1.28-1.28)	163	0.92 (0.92-0.92)	284	1.65 (1.65-1.66)
1995-2004	35-40	Bipolar disorder	734	2.04 (2.04-2.04)	327	1.78 (1.77-1.78)	407	2.32 (2.31-2.32)

			Both sexes		Males		Females	
Years	Age	Mental disorder	Cases	IR (95% CI)	Cases	IR (95% CI)	Cases	IR (95% CI)
2005-2016	35-40	Bipolar disorder	1511	3.91 (3.91-3.92)	614	3.11 (3.11-3.12)	897	4.74 (4.74-4.75)
1970-2016	40-45	Bipolar disorder	3708	2.38 (2.38-2.38)	1509	1.92 (1.92-1.92)	2199	2.86 (2.85-2.86)
1970-1984	40-45	Bipolar disorder	903	2.08 (2.08-2.08)	339	1.56 (1.56-1.56)	564	2.60 (2.60-2.61)
1985-1994	40-45	Bipolar disorder	523	1.42 (1.42-1.42)	209	1.12 (1.12-1.13)	314	1.72 (1.72-1.72)
1995-2004	40-45	Bipolar disorder	779	2.30 (2.30-2.30)	334	1.94 (1.94-1.94)	445	2.67 (2.67-2.68)
2005-2016	40-45	Bipolar disorder	1503	3.61 (3.61-3.62)	627	2.96 (2.96-2.96)	876	4.29 (4.28-4.30)
1970-2016	45-50	Bipolar disorder	3915	2.59 (2.58-2.59)	1588	2.09 (2.09-2.09)	2327	3.08 (3.08-3.09)
1970-1984	45-50	Bipolar disorder	999	2.38 (2.38-2.38)	355	1.71 (1.70-1.71)	644	3.04 (3.03-3.04)
1985-1994	45-50	Bipolar disorder	564	1.67 (1.67-1.67)	227	1.34 (1.34-1.34)	337	2.00 (2.00-2.01)
1995-2004	45-50	Bipolar disorder	916	2.70 (2.70-2.70)	369	2.16 (2.16-2.17)	547	3.25 (3.24-3.25)
2005-2016	45-50	Bipolar disorder	1436	3.44 (3.44-3.44)	637	3.02 (3.01-3.02)	799	3.88 (3.87-3.88)
1970-2016	50-55	Bipolar disorder	3829	2.64 (2.64-2.64)	1615	2.24 (2.24-2.24)	2214	3.04 (3.04-3.04)
1970-1984	50-55	Bipolar disorder	1062	2.51 (2.51-2.52)	386	1.86 (1.85-1.86)	676	3.15 (3.14-3.15)
1985-1994	50-55	Bipolar disorder	495	1.77 (1.77-1.77)	183	1.32 (1.32-1.32)	312	2.22 (2.21-2.22)
1995-2004	50-55	Bipolar disorder	947	2.69 (2.69-2.69)	429	2.44 (2.43-2.44)	518	2.94 (2.93-2.94)
2005-2016	50-55	Bipolar disorder	1325	3.35 (3.35-3.35)	617	3.11 (3.10-3.11)	708	3.60 (3.59-3.60)
1970-2016	55-60	Bipolar disorder	3359	2.45 (2.45-2.45)	1435	2.13 (2.13-2.13)	1924	2.76 (2.76-2.76)
1970-1984	55-60	Bipolar disorder	956	2.29 (2.29-2.29)	352	1.73 (1.73-1.73)	604	2.82 (2.82-2.82)
1985-1994	55-60	Bipolar disorder	483	1.91 (1.91-1.91)	214	1.73 (1.73-1.73)	269	2.08 (2.07-2.08)
1995-2004	55-60	Bipolar disorder	871	2.75 (2.74-2.75)	375	2.39 (2.38-2.39)	496	3.10 (3.10-3.11)
2005-2016	55-60	Bipolar disorder	1049	2.74 (2.74-2.74)	494	2.59 (2.59-2.60)	555	2.88 (2.88-2.89)
1970-2016	60-65	Bipolar disorder	2801	2.19 (2.19-2.19)	1195	1.93 (1.93-1.93)	1606	2.43 (2.43-2.43)
1970-1984	60-65	Bipolar disorder	801	2.01 (2.01-2.02)	314	1.65 (1.65-1.65)	487	2.35 (2.35-2.35)
1985-1994	60-65	Bipolar disorder	447	1.82 (1.82-1.82)	169	1.44 (1.44-1.44)	278	2.17 (2.16-2.17)
1995-2004	60-65	Bipolar disorder	673	2.65 (2.65-2.65)	270	2.19 (2.19-2.19)	403	3.09 (3.08-3.09)
2005-2016	60-65	Bipolar disorder	880	2.29 (2.29-2.29)	442	2.34 (2.34-2.35)	438	2.24 (2.24-2.24)
1970-2016	65-70	Bipolar disorder	2250	1.96 (1.96-1.96)	871	1.61 (1.61-1.62)	1379	2.27 (2.27-2.27)
1970-1984	65-70	Bipolar disorder	656	1.86 (1.86-1.86)	246	1.51 (1.51-1.51)	410	2.16 (2.16-2.17)
1985-1994	65-70	Bipolar disorder	394	1.68 (1.68-1.68)	130	1.20 (1.20-1.20)	264	2.09 (2.09-2.09)
1995-2004	65-70	Bipolar disorder	546	2.51 (2.51-2.52)	203	1.98 (1.98-1.99)	343	2.98 (2.98-2.99)
2005-2016	65-70	Bipolar disorder	654	1.90 (1.90-1.91)	292	1.76 (1.76-1.76)	362	2.04 (2.04-2.04)
1970-2016	70-75	Bipolar disorder	1813	1.91 (1.91-1.91)	621	1.46 (1.46-1.46)	1192	2.28 (2.27-2.28)
1970-1984	70-75	Bipolar disorder	440	1.53 (1.53-1.53)	143	1.14 (1.14-1.14)	297	1.83 (1.83-1.84)
1985-1994	70-75	Bipolar disorder	314	1.53 (1.53-1.54)	96	1.07 (1.07-1.07)	218	1.90 (1.90-1.90)
1995-2004	70-75	Bipolar disorder	532	2.76 (2.76-2.77)	189	2.19 (2.18-2.19)	343	3.23 (3.22-3.24)
2005-2016	70-75	Bipolar disorder	527	1.99 (1.99-1.99)	193	1.56 (1.55-1.56)	334	2.37 (2.37-2.38)
1970-2016	75-80	Bipolar disorder	1277	1.76 (1.76-1.76)	397	1.31 (1.31-1.31)	880	2.09 (2.08-2.09)
1970-1984	75-80	Bipolar disorder	224	1.10 (1.09-1.10)	71	0.86 (0.85-0.86)	153	1.26 (1.26-1.26)
1985-1994	75-80	Bipolar disorder	236	1.45 (1.45-1.45)	75	1.14 (1.14-1.14)	161	1.66 (1.66-1.66)
1995-2004	75-80	Bipolar disorder	471	2.92 (2.92-2.92)	126	1.89 (1.88-1.89)	345	3.65 (3.64-3.66)

			Both sexes		Males		Females	
**Years**	**Age**	**Mental disorder**	**Cases**	**IR (95% CI)**	**Cases**	**IR (95% CI)**	**Cases**	**IR (95% CI)**
2005-2016	75-80	Bipolar disorder	346	1.76 (1.76-1.77)	125	1.44 (1.43-1.44)	221	2.03 (2.02-2.03)
1970-2016	80-85	Bipolar disorder	781	1.61 (1.61-1.61)	239	1.30 (1.30-1.31)	542	1.80 (1.79-1.80)
1970-1984	80-85	Bipolar disorder	92	0.75 (0.75-0.76)	31	0.67 (0.67-0.67)	61	0.81 (0.81-0.81)
1985-1994	80-85	Bipolar disorder	114	1.05 (1.04-1.05)	27	0.70 (0.70-0.70)	87	1.24 (1.23-1.24)
1995-2004	80-85	Bipolar disorder	323	2.83 (2.82-2.83)	88	2.10 (2.09-2.10)	235	3.25 (3.24-3.26)
2005-2016	80-85	Bipolar disorder	252	1.80 (1.80-1.80)	93	1.65 (1.65-1.65)	159	1.90 (1.89-1.90)
1970-2016	85-90	Bipolar disorder	372	1.43 (1.43-1.43)	89	1.04 (1.03-1.04)	283	1.63 (1.62-1.63)
1970-1984	85-90	Bipolar disorder	28	0.51 (0.51-0.51)	8	0.40 (0.40-0.40)	20	0.57 (0.56-0.57)
1985-1994	85-90	Bipolar disorder	47	0.86 (0.86-0.86)	8	0.48 (0.47-0.48)	39	1.03 (1.03-1.03)
1995-2004	85-90	Bipolar disorder	156	2.41 (2.40-2.42)	38	1.89 (1.88-1.90)	118	2.64 (2.64-2.65)
2005-2016	85-90	Bipolar disorder	141	1.65 (1.65-1.65)	35	1.20 (1.20-1.20)	106	1.88 (1.88-1.89)
1970-2016	90-95	Bipolar disorder	96	1.01 (1.01-1.01)	19	0.72 (0.72-0.72)	77	1.12 (1.12-1.12)
1970-1984	90-95	Bipolar disorder	6	0.37 (0.37-0.38)	0-4	NA	0-4	NA
1985-1994	90-95	Bipolar disorder	11	0.59 (0.59-0.59)	0-4	NA	7-11	0.67 (0.66-0.67)
1995-2004	90-95	Bipolar disorder	38	1.54 (1.54-1.55)	6	0.98 (0.97-0.99)	32	1.73 (1.72-1.73)
2005-2016	90-95	Bipolar disorder	41	1.14 (1.13-1.14)	8	0.82 (0.82-0.83)	33	1.25 (1.25-1.26)
1970-2016	95-100	Bipolar disorder	15	0.75 (0.75-0.76)	0-4	NA	11-15	0.78 (0.77-0.78)
1970-1984	95-100	Bipolar disorder	0-4	NA	0-4	NA	0-4	NA
1985-1994	95-100	Bipolar disorder	0-4	NA	0-4	NA	0-4	NA
1995-2004	95-100	Bipolar disorder	5	0.99 (0.98-1.00)	0-4	NA	0-4	NA
2005-2016	95-100	Bipolar disorder	9	1.02 (1.02-1.03)	0-4	NA	5-9	1.00 (0.99-1.01)
1970-2016	10-15	Bipolar disorder (inpatient)	80	0.05 (0.05-0.05)	38	0.05 (0.05-0.05)	42	0.05 (0.05-0.05)
1970-1984	10-15	Bipolar disorder (inpatient)	20	0.04 (0.04-0.04)	15	0.05 (0.05-0.05)	5	0.02 (0.02-0.02)
1985-1994	10-15	Bipolar disorder (inpatient)	8	0.03 (0.03-0.03)	4-8	0.03 (0.03-0.03)	0-4	NA
1995-2004	10-15	Bipolar disorder (inpatient)	10	0.03 (0.03-0.03)	0-4	NA	6-10	0.05 (0.05-0.05)
2005-2016	10-15	Bipolar disorder (inpatient)	42	0.10 (0.10-0.10)	15	0.07 (0.07-0.07)	27	0.14 (0.14-0.14)
1970-2016	15-20	Bipolar disorder (inpatient)	1016	0.63 (0.63-0.63)	456	0.55 (0.55-0.55)	560	0.72 (0.72-0.72)
1970-1984	15-20	Bipolar disorder (inpatient)	380	0.67 (0.67-0.67)	180	0.61 (0.61-0.61)	200	0.72 (0.72-0.72)
1985-1994	15-20	Bipolar disorder (inpatient)	155	0.44 (0.44-0.44)	67	0.37 (0.37-0.37)	88	0.51 (0.51-0.51)
1995-2004	15-20	Bipolar disorder (inpatient)	134	0.47 (0.47-0.47)	65	0.45 (0.45-0.45)	69	0.50 (0.50-0.50)
2005-2016	15-20	Bipolar disorder (inpatient)	347	0.87 (0.87-0.87)	144	0.70 (0.70-0.70)	203	1.05 (1.05-1.05)
1970-2016	20-25	Bipolar disorder (inpatient)	1928	1.19 (1.19-1.20)	874	1.05 (1.05-1.05)	1054	1.35 (1.35-1.35)
1970-1984	20-25	Bipolar disorder (inpatient)	739	1.33 (1.33-1.33)	361	1.26 (1.26-1.26)	378	1.41 (1.41-1.41)
1985-1994	20-25	Bipolar disorder (inpatient)	280	0.75 (0.75-0.75)	125	0.64 (0.64-0.64)	155	0.87 (0.87-0.87)
1995-2004	20-25	Bipolar disorder (inpatient)	254	0.82 (0.82-0.82)	132	0.82 (0.82-0.82)	122	0.81 (0.81-0.82)
2005-2016	20-25	Bipolar disorder (inpatient)	655	1.74 (1.74-1.75)	256	1.33 (1.32-1.33)	399	2.19 (2.18-2.19)
1970-2016	25-30	Bipolar disorder (inpatient)	2077	1.28 (1.28-1.28)	885	1.06 (1.06-1.06)	1192	1.52 (1.52-1.52)
1970-1984	25-30	Bipolar disorder (inpatient)	810	1.43 (1.43-1.43)	369	1.27 (1.26-1.27)	441	1.60 (1.60-1.60)
1985-1994	25-30	Bipolar disorder (inpatient)	311	0.84 (0.84-0.84)	113	0.59 (0.59-0.59)	198	1.11 (1.11-1.12)
1995-2004	25-30	Bipolar disorder (inpatient)	289	0.86 (0.86-0.86)	131	0.75 (0.75-0.75)	158	0.97 (0.97-0.97)

			Both sexes		Males		Females	
**Years**	**Age**	**Mental disorder**	**Cases**	**IR (95% CI)**	**Cases**	**IR (95% CI)**	**Cases**	**IR (95% CI)**
2005-2016	25-30	Bipolar disorder (inpatient)	667	1.92 (1.92-1.92)	272	1.53 (1.53-1.53)	395	2.33 (2.32-2.33)
1970-2016	30-35	Bipolar disorder (inpatient)	2319	1.44 (1.44-1.44)	955	1.16 (1.16-1.16)	1364	1.74 (1.74-1.74)
1970-1984	30-35	Bipolar disorder (inpatient)	882	1.62 (1.62-1.63)	362	1.31 (1.31-1.31)	520	1.95 (1.95-1.96)
1985-1994	30-35	Bipolar disorder (inpatient)	346	1.00 (1.00-1.00)	128	0.72 (0.72-0.72)	218	1.30 (1.29-1.30)
1995-2004	30-35	Bipolar disorder (inpatient)	394	1.09 (1.09-1.09)	169	0.91 (0.91-0.91)	225	1.29 (1.29-1.29)
2005-2016	30-35	Bipolar disorder (inpatient)	697	1.95 (1.95-1.96)	296	1.63 (1.62-1.63)	401	2.30 (2.29-2.30)
1970-2016	35-40	Bipolar disorder (inpatient)	2466	1.55 (1.55-1.55)	1021	1.26 (1.26-1.26)	1445	1.85 (1.85-1.85)
1970-1984	35-40	Bipolar disorder (inpatient)	876	1.77 (1.77-1.77)	334	1.33 (1.33-1.34)	542	2.22 (2.21-2.22)
1985-1994	35-40	Bipolar disorder (inpatient)	421	1.20 (1.20-1.21)	151	0.85 (0.85-0.85)	270	1.57 (1.57-1.57)
1995-2004	35-40	Bipolar disorder (inpatient)	458	1.27 (1.27-1.27)	212	1.15 (1.15-1.15)	246	1.40 (1.40-1.40)
2005-2016	35-40	Bipolar disorder (inpatient)	711	1.84 (1.84-1.84)	324	1.64 (1.64-1.64)	387	2.04 (2.04-2.05)
1970-2016	40-45	Bipolar disorder (inpatient)	2703	1.74 (1.73-1.74)	1086	1.38 (1.38-1.38)	1617	2.10 (2.10-2.10)
1970-1984	40-45	Bipolar disorder (inpatient)	902	2.08 (2.08-2.08)	338	1.56 (1.55-1.56)	564	2.60 (2.60-2.61)
1985-1994	40-45	Bipolar disorder (inpatient)	496	1.35 (1.35-1.35)	194	1.04 (1.04-1.05)	302	1.65 (1.65-1.66)
1995-2004	40-45	Bipolar disorder (inpatient)	494	1.46 (1.46-1.46)	209	1.21 (1.21-1.22)	285	1.71 (1.71-1.71)
2005-2016	40-45	Bipolar disorder (inpatient)	811	1.95 (1.94-1.95)	345	1.63 (1.62-1.63)	466	2.28 (2.28-2.28)
1970-2016	45-50	Bipolar disorder (inpatient)	2955	1.95 (1.95-1.95)	1191	1.57 (1.57-1.57)	1764	2.34 (2.33-2.34)
1970-1984	45-50	Bipolar disorder (inpatient)	999	2.38 (2.38-2.38)	355	1.71 (1.70-1.71)	644	3.04 (3.03-3.04)
1985-1994	45-50	Bipolar disorder (inpatient)	529	1.57 (1.56-1.57)	211	1.24 (1.24-1.25)	318	1.89 (1.89-1.89)
1995-2004	45-50	Bipolar disorder (inpatient)	613	1.81 (1.80-1.81)	246	1.44 (1.44-1.44)	367	2.18 (2.17-2.18)
2005-2016	45-50	Bipolar disorder (inpatient)	814	1.95 (1.95-1.95)	379	1.79 (1.79-1.79)	435	2.11 (2.10-2.11)
1970-2016	50-55	Bipolar disorder (inpatient)	3002	2.07 (2.07-2.07)	1227	1.70 (1.70-1.70)	1775	2.43 (2.43-2.44)
1970-1984	50-55	Bipolar disorder (inpatient)	1061	2.51 (2.51-2.51)	386	1.86 (1.85-1.86)	675	3.14 (3.14-3.15)
1985-1994	50-55	Bipolar disorder (inpatient)	465	1.66 (1.66-1.67)	176	1.27 (1.27-1.27)	289	2.05 (2.05-2.06)
1995-2004	50-55	Bipolar disorder (inpatient)	646	1.83 (1.83-1.83)	277	1.57 (1.57-1.58)	369	2.09 (2.09-2.09)
2005-2016	50-55	Bipolar disorder (inpatient)	830	2.10 (2.09-2.10)	388	1.95 (1.95-1.95)	442	2.24 (2.24-2.25)
1970-2016	55-60	Bipolar disorder (inpatient)	2715	1.98 (1.98-1.98)	1138	1.69 (1.69-1.69)	1577	2.26 (2.26-2.26)
1970-1984	55-60	Bipolar disorder (inpatient)	956	2.29 (2.29-2.29)	352	1.73 (1.73-1.73)	604	2.82 (2.82-2.82)
1985-1994	55-60	Bipolar disorder (inpatient)	449	1.77 (1.77-1.78)	197	1.59 (1.59-1.60)	252	1.94 (1.94-1.95)
1995-2004	55-60	Bipolar disorder (inpatient)	612	1.93 (1.93-1.93)	261	1.66 (1.66-1.66)	351	2.19 (2.19-2.20)
2005-2016	55-60	Bipolar disorder (inpatient)	698	1.82 (1.82-1.82)	328	1.72 (1.72-1.72)	370	1.92 (1.92-1.92)
1970-2016	60-65	Bipolar disorder (inpatient)	2305	1.80 (1.80-1.80)	969	1.56 (1.56-1.56)	1336	2.02 (2.02-2.02)
1970-1984	60-65	Bipolar disorder (inpatient)	801	2.01 (2.01-2.02)	314	1.65 (1.65-1.65)	487	2.35 (2.35-2.35)
1985-1994	60-65	Bipolar disorder (inpatient)	406	1.65 (1.65-1.66)	154	1.31 (1.31-1.32)	252	1.96 (1.96-1.97)
1995-2004	60-65	Bipolar disorder (inpatient)	464	1.83 (1.82-1.83)	180	1.46 (1.46-1.46)	284	2.17 (2.17-2.18)
2005-2016	60-65	Bipolar disorder (inpatient)	634	1.65 (1.65-1.65)	321	1.70 (1.70-1.70)	313	1.60 (1.60-1.60)
1970-2016	65-70	Bipolar disorder (inpatient)	1847	1.61 (1.61-1.61)	717	1.33 (1.33-1.33)	1130	1.86 (1.86-1.86)
1970-1984	65-70	Bipolar disorder (inpatient)	656	1.86 (1.86-1.86)	246	1.51 (1.51-1.51)	410	2.16 (2.16-2.17)
1985-1994	65-70	Bipolar disorder (inpatient)	349	1.49 (1.48-1.49)	114	1.05 (1.05-1.05)	235	1.86 (1.86-1.86)
1995-2004	65-70	Bipolar disorder (inpatient)	383	1.76 (1.76-1.76)	144	1.41 (1.40-1.41)	239	2.08 (2.07-2.08)

			Both sexes		Males		Females	
Years	Age	Mental disorder	Cases	IR (95% CI)	Cases	IR (95% CI)	Cases	IR (95% CI)
2005-2016	65-70	Bipolar disorder (inpatient)	459	1.34 (1.33-1.34)	213	1.28 (1.28-1.29)	246	1.38 (1.38-1.39)
1970-2016	70-75	Bipolar disorder (inpatient)	1436	1.51 (1.51-1.51)	493	1.16 (1.16-1.16)	943	1.80 (1.80-1.80)
1970-1984	70-75	Bipolar disorder (inpatient)	440	1.53 (1.53-1.53)	143	1.14 (1.14-1.14)	297	1.83 (1.83-1.84)
1985-1994	70-75	Bipolar disorder (inpatient)	279	1.36 (1.36-1.37)	87	0.97 (0.97-0.97)	192	1.67 (1.67-1.68)
1995-2004	70-75	Bipolar disorder (inpatient)	335	1.74 (1.74-1.74)	116	1.34 (1.34-1.34)	219	2.06 (2.06-2.06)
2005-2016	70-75	Bipolar disorder (inpatient)	382	1.44 (1.44-1.44)	147	1.18 (1.18-1.19)	235	1.67 (1.66-1.67)
1970-2016	75-80	Bipolar disorder (inpatient)	944	1.30 (1.30-1.30)	297	0.98 (0.98-0.98)	647	1.53 (1.53-1.53)
1970-1984	75-80	Bipolar disorder (inpatient)	224	1.10 (1.09-1.10)	71	0.86 (0.85-0.86)	153	1.26 (1.26-1.26)
1985-1994	75-80	Bipolar disorder (inpatient)	211	1.29 (1.29-1.30)	68	1.03 (1.03-1.03)	143	1.47 (1.47-1.48)
1995-2004	75-80	Bipolar disorder (inpatient)	291	1.80 (1.80-1.81)	81	1.21 (1.21-1.22)	210	2.22 (2.22-2.22)
2005-2016	75-80	Bipolar disorder (inpatient)	218	1.11 (1.11-1.11)	77	0.88 (0.88-0.89)	141	1.29 (1.29-1.29)
1970-2016	80-85	Bipolar disorder (inpatient)	523	1.08 (1.08-1.08)	167	0.91 (0.91-0.91)	356	1.18 (1.18-1.18)
1970-1984	80-85	Bipolar disorder (inpatient)	92	0.75 (0.75-0.76)	31	0.67 (0.67-0.67)	61	0.81 (0.81-0.81)
1985-1994	80-85	Bipolar disorder (inpatient)	102	0.94 (0.93-0.94)	25	0.65 (0.65-0.65)	77	1.10 (1.09-1.10)
1995-2004	80-85	Bipolar disorder (inpatient)	187	1.64 (1.63-1.64)	59	1.41 (1.40-1.41)	128	1.77 (1.77-1.77)
2005-2016	80-85	Bipolar disorder (inpatient)	142	1.01 (1.01-1.01)	52	0.92 (0.92-0.92)	90	1.07 (1.07-1.08)
1970-2016	85-90	Bipolar disorder (inpatient)	218	0.84 (0.84-0.84)	54	0.63 (0.63-0.63)	164	0.94 (0.94-0.94)
1970-1984	85-90	Bipolar disorder (inpatient)	28	0.51 (0.51-0.51)	8	0.40 (0.40-0.40)	20	0.57 (0.56-0.57)
1985-1994	85-90	Bipolar disorder (inpatient)	43	0.79 (0.78-0.79)	8	0.48 (0.47-0.48)	35	0.92 (0.92-0.93)
1995-2004	85-90	Bipolar disorder (inpatient)	79	1.22 (1.22-1.22)	18	0.90 (0.89-0.90)	61	1.37 (1.36-1.37)
2005-2016	85-90	Bipolar disorder (inpatient)	68	0.80 (0.79-0.80)	20	0.69 (0.68-0.69)	48	0.85 (0.85-0.85)
1970-2016	90-95	Bipolar disorder (inpatient)	48	0.50 (0.50-0.50)	11	0.42 (0.41-0.42)	37	0.54 (0.54-0.54)
1970-1984	90-95	Bipolar disorder (inpatient)	6	0.37 (0.37-0.38)	0-4	NA	0-4	NA
1985-1994	90-95	Bipolar disorder (inpatient)	9	0.48 (0.48-0.49)	0-4	NA	5-9	0.52 (0.52-0.52)
1995-2004	90-95	Bipolar disorder (inpatient)	16	0.65 (0.65-0.65)	0-4	NA	12-16	0.70 (0.70-0.70)
2005-2016	90-95	Bipolar disorder (inpatient)	17	0.47 (0.47-0.47)	0-4	NA	13-17	0.53 (0.53-0.53)
1970-2016	95-100	Bipolar disorder (inpatient)	7	0.35 (0.35-0.35)	0-4	NA	3-7	0.39 (0.39-0.39)
1970-1984	95-100	Bipolar disorder (inpatient)	0-4	NA	0-4	NA	0-4	NA
1985-1994	95-100	Bipolar disorder (inpatient)	0-4	NA	0-4	NA	0-4	NA
1995-2004	95-100	Bipolar disorder (inpatient)	0-4	NA	0-4	NA	0-4	NA
2005-2016	95-100	Bipolar disorder (inpatient)	5	0.57 (0.56-0.57)	0-4	NA	0-4	NA
1970-2016	10-15	Recurrent depression	297	0.19 (0.19-0.19)	127	0.16 (0.16-0.16)	170	0.22 (0.22-0.22)
1970-1984	10-15	Recurrent depression	0-4	NA	0-4	NA	0-4	NA
1985-1994	10-15	Recurrent depression	0-4	NA	0-4	NA	0-4	NA
1995-2004	10-15	Recurrent depression	92	0.31 (0.31-0.31)	38	0.25 (0.25-0.25)	54	0.37 (0.37-0.38)
2005-2016	10-15	Recurrent depression	202	0.50 (0.50-0.50)	88	0.43 (0.43-0.43)	114	0.58 (0.58-0.58)
1970-2016	15-20	Recurrent depression	3019	1.88 (1.88-1.88)	768	0.93 (0.93-0.93)	2251	2.88 (2.88-2.88)
1970-1984	15-20	Recurrent depression	69	0.12 (0.12-0.12)	22	0.08 (0.07-0.08)	47	0.17 (0.17-0.17)
1985-1994	15-20	Recurrent depression	13	0.04 (0.04-0.04)	0-4	NA	9-13	0.06 (0.06-0.06)
1995-2004	15-20	Recurrent depression	568	2.00 (1.99-2.00)	138	0.95 (0.95-0.95)	430	3.10 (3.09-3.10)

			Both sexes		Males		Females	
Years	Age	Mental disorder	Cases	IR (95% CI)	Cases	IR (95% CI)	Cases	IR (95% CI)
2005-2016	15-20	Recurrent depression	2369	5.95 (5.94-5.95)	605	2.96 (2.95-2.96)	1764	9.11 (9.09-9.12)
1970-2016	20-25	Recurrent depression	7468	4.63 (4.63-4.64)	2184	2.62 (2.62-2.62)	5284	6.79 (6.79-6.80)
1970-1984	20-25	Recurrent depression	306	0.55 (0.55-0.55)	111	0.39 (0.39-0.39)	195	0.73 (0.73-0.73)
1985-1994	20-25	Recurrent depression	80	0.21 (0.21-0.21)	30	0.15 (0.15-0.15)	50	0.28 (0.28-0.28)
1995-2004	20-25	Recurrent depression	1361	4.39 (4.39-4.40)	357	2.23 (2.22-2.23)	1004	6.71 (6.70-6.72)
2005-2016	20-25	Recurrent depression	5721	15.31 (15.30-15.33)	1686	8.76 (8.75-8.77)	4035	22.27 (22.24-22.31)
1970-2016	25-30	Recurrent depression	7933	4.91 (4.90-4.91)	2565	3.08 (3.07-3.08)	5368	6.85 (6.85-6.86)
1970-1984	25-30	Recurrent depression	675	1.19 (1.19-1.19)	239	0.82 (0.82-0.82)	436	1.58 (1.58-1.58)
1985-1994	25-30	Recurrent depression	153	0.41 (0.41-0.41)	49	0.26 (0.26-0.26)	104	0.59 (0.58-0.59)
1995-2004	25-30	Recurrent depression	1762	5.23 (5.23-5.24)	580	3.33 (3.32-3.33)	1182	7.28 (7.27-7.29)
2005-2016	25-30	Recurrent depression	5343	15.52 (15.50-15.54)	1697	9.60 (9.58-9.61)	3646	21.78 (21.74-21.81)
1970-2016	30-35	Recurrent depression	8594	5.36 (5.36-5.36)	2785	3.39 (3.38-3.39)	5809	7.44 (7.43-7.44)
1970-1984	30-35	Recurrent depression	1089	2.00 (2.00-2.01)	357	1.29 (1.29-1.29)	732	2.75 (2.75-2.75)
1985-1994	30-35	Recurrent depression	352	1.02 (1.01-1.02)	112	0.63 (0.63-0.63)	240	1.43 (1.42-1.43)
1995-2004	30-35	Recurrent depression	1997	5.54 (5.54-5.55)	655	3.52 (3.52-3.53)	1342	7.70 (7.68-7.71)
2005-2016	30-35	Recurrent depression	5156	14.60 (14.58-14.61)	1661	9.17 (9.16-9.18)	3495	20.32 (20.29-20.35)
1970-2016	35-40	Recurrent depression	9515	6.00 (5.99-6.00)	3296	4.08 (4.07-4.08)	6219	7.99 (7.99-8.00)
1970-1984	35-40	Recurrent depression	1461	2.95 (2.95-2.96)	490	1.96 (1.96-1.96)	971	3.97 (3.97-3.98)
1985-1994	35-40	Recurrent depression	488	1.39 (1.39-1.40)	153	0.86 (0.86-0.86)	335	1.95 (1.95-1.95)
1995-2004	35-40	Recurrent depression	2382	6.63 (6.62-6.63)	866	4.70 (4.70-4.71)	1516	8.65 (8.64-8.66)
2005-2016	35-40	Recurrent depression	5184	13.54 (13.53-13.55)	1787	9.11 (9.09-9.12)	3397	18.20 (18.17-18.22)
1970-2016	40-45	Recurrent depression	9679	6.23 (6.23-6.24)	3572	4.55 (4.54-4.55)	6107	7.96 (7.96-7.97)
1970-1984	40-45	Recurrent depression	1626	3.75 (3.75-3.75)	492	2.27 (2.26-2.27)	1134	5.24 (5.23-5.24)
1985-1994	40-45	Recurrent depression	654	1.78 (1.77-1.78)	217	1.17 (1.17-1.17)	437	2.40 (2.39-2.40)
1995-2004	40-45	Recurrent depression	2372	7.02 (7.01-7.02)	933	5.42 (5.42-5.43)	1439	8.66 (8.65-8.68)
2005-2016	40-45	Recurrent depression	5027	12.18 (12.17-12.20)	1930	9.16 (9.14-9.17)	3097	15.35 (15.32-15.37)
1970-2016	45-50	Recurrent depression	10056	6.66 (6.66-6.66)	3715	4.90 (4.90-4.90)	6341	8.44 (8.43-8.44)
1970-1984	45-50	Recurrent depression	1889	4.50 (4.50-4.51)	598	2.88 (2.87-2.88)	1291	6.10 (6.09-6.11)
1985-1994	45-50	Recurrent depression	798	2.36 (2.36-2.37)	263	1.55 (1.55-1.55)	535	3.19 (3.18-3.19)
1995-2004	45-50	Recurrent depression	2407	7.11 (7.10-7.12)	928	5.44 (5.43-5.45)	1479	8.80 (8.79-8.81)
2005-2016	45-50	Recurrent depression	4962	11.99 (11.98-12.00)	1926	9.17 (9.16-9.18)	3036	14.89 (14.87-14.91)
1970-2016	50-55	Recurrent depression	9550	6.61 (6.61-6.61)	3506	4.87 (4.87-4.88)	6044	8.33 (8.33-8.34)
1970-1984	50-55	Recurrent depression	2150	5.09 (5.09-5.10)	637	3.07 (3.06-3.07)	1513	7.06 (7.05-7.07)
1985-1994	50-55	Recurrent depression	795	2.85 (2.85-2.85)	247	1.78 (1.78-1.79)	548	3.90 (3.90-3.91)
1995-2004	50-55	Recurrent depression	2487	7.07 (7.07-7.08)	962	5.47 (5.46-5.48)	1525	8.68 (8.66-8.69)
2005-2016	50-55	Recurrent depression	4118	10.50 (10.49-10.51)	1660	8.40 (8.39-8.41)	2458	12.63 (12.61-12.65)
1970-2016	55-60	Recurrent depression	8646	6.33 (6.33-6.33)	3160	4.69 (4.69-4.70)	5486	7.92 (7.91-7.93)
1970-1984	55-60	Recurrent depression	2185	5.24 (5.23-5.24)	634	3.12 (3.11-3.12)	1551	7.26 (7.25-7.27)
1985-1994	55-60	Recurrent depression	827	3.28 (3.27-3.28)	249	2.02 (2.01-2.02)	578	4.48 (4.47-4.49)
1995-2004	55-60	Recurrent depression	2131	6.74 (6.73-6.75)	805	5.13 (5.12-5.14)	1326	8.33 (8.32-8.34)

			Both sexes		Males		Females	
Years	Age	Mental disorder	Cases	IR (95% CI)	Cases	IR (95% CI)	Cases	IR (95% CI)
2005-2016	55-60	Recurrent depression	3503	9.21 (9.20-9.22)	1472	7.77 (7.76-7.78)	2031	10.65 (10.64-10.67)
1970-2016	60-65	Recurrent depression	7084	5.55 (5.55-5.56)	2400	3.88 (3.88-3.89)	4684	7.12 (7.12-7.13)
1970-1984	60-65	Recurrent depression	2005	5.05 (5.05-5.06)	522	2.74 (2.74-2.75)	1483	7.18 (7.17-7.19)
1985-1994	60-65	Recurrent depression	894	3.65 (3.65-3.66)	240	2.05 (2.05-2.05)	654	5.13 (5.12-5.13)
1995-2004	60-65	Recurrent depression	1618	6.40 (6.39-6.41)	549	4.46 (4.45-4.47)	1069	8.24 (8.22-8.25)
2005-2016	60-65	Recurrent depression	2567	6.73 (6.72-6.74)	1089	5.80 (5.79-5.81)	1478	7.63 (7.62-7.64)
1970-2016	65-70	Recurrent depression	6638	5.81 (5.80-5.81)	2095	3.89 (3.89-3.89)	4543	7.52 (7.51-7.52)
1970-1984	65-70	Recurrent depression	1745	4.96 (4.96-4.97)	465	2.85 (2.85-2.86)	1280	6.78 (6.77-6.79)
1985-1994	65-70	Recurrent depression	954	4.08 (4.07-4.08)	257	2.37 (2.37-2.38)	697	5.55 (5.54-5.56)
1995-2004	65-70	Recurrent depression	1651	7.63 (7.62-7.64)	500	4.89 (4.88-4.90)	1151	10.09 (10.07-10.11)
2005-2016	65-70	Recurrent depression	2288	6.71 (6.70-6.72)	873	5.29 (5.28-5.29)	1415	8.04 (8.03-8.05)
1970-2016	70-75	Recurrent depression	6289	6.66 (6.65-6.66)	1745	4.11 (4.10-4.11)	4544	8.74 (8.73-8.75)
1970-1984	70-75	Recurrent depression	1370	4.77 (4.77-4.78)	283	2.25 (2.25-2.26)	1087	6.73 (6.72-6.74)
1985-1994	70-75	Recurrent depression	890	4.37 (4.36-4.38)	207	2.31 (2.30-2.31)	683	6.00 (5.99-6.01)
1995-2004	70-75	Recurrent depression	1897	9.91 (9.90-9.92)	520	6.03 (6.02-6.05)	1377	13.08 (13.06-13.11)
2005-2016	70-75	Recurrent depression	2132	8.11 (8.10-8.12)	735	5.95 (5.94-5.96)	1397	10.03 (10.02-10.05)
1970-2016	75-80	Recurrent depression	5512	7.65 (7.65-7.66)	1406	4.66 (4.65-4.66)	4106	9.82 (9.81-9.83)
1970-1984	75-80	Recurrent depression	820	4.02 (4.02-4.03)	169	2.04 (2.03-2.04)	651	5.38 (5.37-5.39)
1985-1994	75-80	Recurrent depression	714	4.40 (4.40-4.41)	146	2.22 (2.21-2.22)	568	5.90 (5.88-5.91)
1995-2004	75-80	Recurrent depression	1956	12.23 (12.21-12.25)	469	7.05 (7.03-7.07)	1487	15.92 (15.89-15.95)
2005-2016	75-80	Recurrent depression	2022	10.41 (10.40-10.43)	622	7.18 (7.17-7.20)	1400	13.02 (12.99-13.04)
1970-2016	80-85	Recurrent depression	4073	8.46 (8.45-8.47)	974	5.33 (5.32-5.34)	3099	10.37 (10.36-10.38)
1970-1984	80-85	Recurrent depression	315	2.59 (2.58-2.59)	63	1.36 (1.36-1.36)	252	3.34 (3.34-3.35)
1985-1994	80-85	Recurrent depression	391	3.61 (3.60-3.61)	68	1.76 (1.76-1.77)	323	4.63 (4.62-4.64)
1995-2004	80-85	Recurrent depression	1608	14.22 (14.19-14.25)	379	9.08 (9.05-9.10)	1229	17.23 (17.19-17.27)
2005-2016	80-85	Recurrent depression	1759	12.71 (12.69-12.73)	464	8.28 (8.26-8.30)	1295	15.73 (15.69-15.76)
1970-2016	85-90	Recurrent depression	2252	8.73 (8.72-8.74)	466	5.44 (5.43-5.45)	1786	10.37 (10.36-10.39)
1970-1984	85-90	Recurrent depression	70	1.27 (1.26-1.27)	20	1.00 (1.00-1.01)	50	1.42 (1.41-1.42)
1985-1994	85-90	Recurrent depression	150	2.76 (2.75-2.76)	23	1.37 (1.36-1.38)	127	3.37 (3.36-3.38)
1995-2004	85-90	Recurrent depression	882	13.76 (13.73-13.80)	167	8.34 (8.31-8.38)	715	16.23 (16.18-16.27)
2005-2016	85-90	Recurrent depression	1150	13.68 (13.65-13.70)	256	8.84 (8.81-8.88)	894	16.21 (16.17-16.25)
1970-2016	90-95	Recurrent depression	691	7.32 (7.31-7.34)	120	4.56 (4.54-4.57)	571	8.39 (8.37-8.41)
1970-1984	90-95	Recurrent depression	0-4	NA	0-4	NA	0-4	NA
1985-1994	90-95	Recurrent depression	12	0.65 (0.64-0.65)	0-4	NA	8-12	0.89 (0.89-0.90)
1995-2004	90-95	Recurrent depression	259	10.61 (10.56-10.65)	44	7.22 (7.16-7.28)	215	11.73 (11.68-11.78)
2005-2016	90-95	Recurrent depression	416	11.74 (11.70-11.78)	76	7.87 (7.82-7.92)	340	13.19 (13.14-13.24)
1970-2016	95-100	Recurrent depression	112	5.68 (5.66-5.71)	16	3.61 (3.57-3.64)	96	6.29 (6.26-6.32)
1970-1984	95-100	Recurrent depression	0-4	NA	0-4	NA	0-4	NA
1985-1994	95-100	Recurrent depression	0-4	NA	0-4	NA	0-4	NA
1995-2004	95-100	Recurrent depression	33	6.58 (6.52-6.64)	0-4	NA	29-33	7.21 (7.14-7.29)

			Both sexes		Males		Females	
Years	Age	Mental disorder	Cases	IR (95% CI)	Cases	IR (95% CI)	Cases	IR (95% CI)
2005-2016	95-100	Recurrent depression	77	8.91 (8.85-8.97)	12	6.80 (6.70-6.90)	65	9.44 (9.37-9.51)
1970-2016	10-15	Recurrent depression (inpatient)	40	0.03 (0.03-0.03)	17	0.02 (0.02-0.02)	23	0.03 (0.03-0.03)
1970-1984	10-15	Recurrent depression (inpatient)	0-4	NA	0-4	NA	0-4	NA
1985-1994	10-15	Recurrent depression (inpatient)	0-4	NA	0-4	NA	0-4	NA
1995-2004	10-15	Recurrent depression (inpatient)	7	0.02 (0.02-0.02)	3-7	0.04 (0.04-0.04)	0-4	NA
2005-2016	10-15	Recurrent depression (inpatient)	30	0.07 (0.07-0.07)	10	0.05 (0.05-0.05)	20	0.10 (0.10-0.10)
1970-2016	15-20	Recurrent depression (inpatient)	752	0.47 (0.47-0.47)	177	0.21 (0.21-0.21)	575	0.73 (0.73-0.74)
1970-1984	15-20	Recurrent depression (inpatient)	69	0.12 (0.12-0.12)	22	0.08 (0.07-0.08)	47	0.17 (0.17-0.17)
1985-1994	15-20	Recurrent depression (inpatient)	13	0.04 (0.04-0.04)	0-4	NA	9-13	0.06 (0.06-0.06)
1995-2004	15-20	Recurrent depression (inpatient)	151	0.53 (0.53-0.53)	25	0.17 (0.17-0.17)	126	0.91 (0.91-0.91)
2005-2016	15-20	Recurrent depression (inpatient)	519	1.30 (1.30-1.30)	127	0.62 (0.62-0.62)	392	2.02 (2.02-2.02)
1970-2016	20-25	Recurrent depression (inpatient)	2157	1.34 (1.34-1.34)	664	0.80 (0.80-0.80)	1493	1.92 (1.91-1.92)
1970-1984	20-25	Recurrent depression (inpatient)	306	0.55 (0.55-0.55)	111	0.39 (0.39-0.39)	195	0.73 (0.73-0.73)
1985-1994	20-25	Recurrent depression (inpatient)	64	0.17 (0.17-0.17)	24	0.12 (0.12-0.12)	40	0.22 (0.22-0.22)
1995-2004	20-25	Recurrent depression (inpatient)	398	1.28 (1.28-1.28)	97	0.60 (0.60-0.61)	301	2.01 (2.01-2.01)
2005-2016	20-25	Recurrent depression (inpatient)	1389	3.70 (3.70-3.71)	432	2.24 (2.24-2.24)	957	5.25 (5.24-5.26)
1970-2016	25-30	Recurrent depression (inpatient)	2750	1.70 (1.70-1.70)	956	1.15 (1.14-1.15)	1794	2.28 (2.28-2.29)
1970-1984	25-30	Recurrent depression (inpatient)	675	1.19 (1.19-1.19)	239	0.82 (0.82-0.82)	436	1.58 (1.58-1.58)
1985-1994	25-30	Recurrent depression (inpatient)	125	0.34 (0.34-0.34)	44	0.23 (0.23-0.23)	81	0.46 (0.45-0.46)
1995-2004	25-30	Recurrent depression (inpatient)	588	1.74 (1.74-1.75)	203	1.16 (1.16-1.17)	385	2.37 (2.36-2.37)
2005-2016	25-30	Recurrent depression (inpatient)	1362	3.93 (3.92-3.93)	470	2.65 (2.64-2.65)	892	5.27 (5.26-5.28)
1970-2016	30-35	Recurrent depression (inpatient)	3822	2.38 (2.38-2.38)	1328	1.61 (1.61-1.61)	2494	3.18 (3.18-3.19)
1970-1984	30-35	Recurrent depression (inpatient)	1088	2.00 (2.00-2.00)	357	1.29 (1.29-1.29)	731	2.75 (2.74-2.75)
1985-1994	30-35	Recurrent depression (inpatient)	312	0.90 (0.90-0.90)	103	0.58 (0.58-0.58)	209	1.24 (1.24-1.24)
1995-2004	30-35	Recurrent depression (inpatient)	830	2.30 (2.30-2.30)	299	1.61 (1.61-1.61)	531	3.04 (3.04-3.04)
2005-2016	30-35	Recurrent depression (inpatient)	1592	4.47 (4.47-4.48)	569	3.13 (3.12-3.13)	1023	5.88 (5.87-5.88)
1970-2016	35-40	Recurrent depression (inpatient)	4699	2.95 (2.95-2.96)	1644	2.03 (2.03-2.03)	3055	3.91 (3.91-3.92)
1970-1984	35-40	Recurrent depression (inpatient)	1461	2.95 (2.95-2.96)	490	1.96 (1.96-1.96)	971	3.97 (3.97-3.98)
1985-1994	35-40	Recurrent depression (inpatient)	448	1.28 (1.28-1.28)	143	0.80 (0.80-0.80)	305	1.77 (1.77-1.78)
1995-2004	35-40	Recurrent depression (inpatient)	1046	2.91 (2.90-2.91)	381	2.07 (2.06-2.07)	665	3.79 (3.78-3.79)
2005-2016	35-40	Recurrent depression (inpatient)	1744	4.52 (4.51-4.52)	630	3.20 (3.19-3.20)	1114	5.90 (5.89-5.91)
1970-2016	40-45	Recurrent depression (inpatient)	5276	3.39 (3.39-3.39)	1946	2.47 (2.47-2.47)	3330	4.33 (4.33-4.33)
1970-1984	40-45	Recurrent depression (inpatient)	1625	3.75 (3.74-3.75)	491	2.26 (2.26-2.26)	1134	5.24 (5.23-5.24)
1985-1994	40-45	Recurrent depression (inpatient)	593	1.61 (1.61-1.61)	198	1.06 (1.06-1.07)	395	2.17 (2.16-2.17)
1995-2004	40-45	Recurrent depression (inpatient)	1135	3.35 (3.35-3.36)	441	2.56 (2.56-2.56)	694	4.17 (4.16-4.18)
2005-2016	40-45	Recurrent depression (inpatient)	1923	4.63 (4.62-4.63)	816	3.85 (3.85-3.86)	1107	5.43 (5.42-5.44)
1970-2016	45-50	Recurrent depression (inpatient)	5787	3.82 (3.82-3.83)	2151	2.83 (2.83-2.83)	3636	4.82 (4.82-4.83)
1970-1984	45-50	Recurrent depression (inpatient)	1889	4.50 (4.50-4.51)	598	2.88 (2.87-2.88)	1291	6.10 (6.09-6.11)
1985-1994	45-50	Recurrent depression (inpatient)	719	2.13 (2.13-2.13)	240	1.42 (1.41-1.42)	479	2.85 (2.85-2.86)
1995-2004	45-50	Recurrent depression (inpatient)	1247	3.68 (3.67-3.68)	493	2.89 (2.88-2.89)	754	4.48 (4.47-4.48)

			Both sexes		Males		Females	
**Years**	**Age**	**Mental disorder**	**Cases**	**IR (95% CI)**	**Cases**	**IR (95% CI)**	**Cases**	**IR (95% CI)**
2005-2016	45-50	Recurrent depression (inpatient)	1932	4.63 (4.63-4.64)	820	3.88 (3.88-3.89)	1112	5.40 (5.39-5.41)
1970-2016	50-55	Recurrent depression (inpatient)	6001	4.14 (4.14-4.15)	2163	3.00 (3.00-3.00)	3838	5.28 (5.27-5.28)
1970-1984	50-55	Recurrent depression (inpatient)	2150	5.09 (5.09-5.10)	637	3.07 (3.06-3.07)	1513	7.06 (7.05-7.07)
1985-1994	50-55	Recurrent depression (inpatient)	734	2.63 (2.63-2.63)	229	1.65 (1.65-1.66)	505	3.60 (3.59-3.60)
1995-2004	50-55	Recurrent depression (inpatient)	1312	3.73 (3.72-3.73)	500	2.84 (2.84-2.84)	812	4.61 (4.60-4.62)
2005-2016	50-55	Recurrent depression (inpatient)	1805	4.57 (4.56-4.57)	797	4.01 (4.01-4.02)	1008	5.13 (5.13-5.14)
1970-2016	55-60	Recurrent depression (inpatient)	5787	4.23 (4.23-4.23)	2059	3.05 (3.05-3.06)	3728	5.37 (5.36-5.37)
1970-1984	55-60	Recurrent depression (inpatient)	2185	5.24 (5.23-5.24)	634	3.12 (3.11-3.12)	1551	7.26 (7.25-7.27)
1985-1994	55-60	Recurrent depression (inpatient)	759	3.01 (3.00-3.01)	229	1.85 (1.85-1.86)	530	4.11 (4.10-4.12)
1995-2004	55-60	Recurrent depression (inpatient)	1155	3.65 (3.64-3.65)	445	2.83 (2.83-2.84)	710	4.45 (4.44-4.46)
2005-2016	55-60	Recurrent depression (inpatient)	1688	4.41 (4.41-4.42)	751	3.94 (3.94-3.95)	937	4.87 (4.87-4.88)
1970-2016	60-65	Recurrent depression (inpatient)	5082	3.98 (3.97-3.98)	1604	2.59 (2.59-2.59)	3478	5.28 (5.27-5.28)
1970-1984	60-65	Recurrent depression (inpatient)	2005	5.05 (5.05-5.06)	522	2.74 (2.74-2.75)	1483	7.18 (7.17-7.19)
1985-1994	60-65	Recurrent depression (inpatient)	826	3.38 (3.37-3.38)	222	1.90 (1.89-1.90)	604	4.73 (4.73-4.74)
1995-2004	60-65	Recurrent depression (inpatient)	900	3.55 (3.55-3.56)	298	2.42 (2.41-2.42)	602	4.63 (4.62-4.64)
2005-2016	60-65	Recurrent depression (inpatient)	1351	3.52 (3.52-3.53)	562	2.98 (2.98-2.98)	789	4.05 (4.04-4.05)
1970-2016	65-70	Recurrent depression (inpatient)	4805	4.20 (4.19-4.20)	1459	2.71 (2.70-2.71)	3346	5.52 (5.52-5.53)
1970-1984	65-70	Recurrent depression (inpatient)	1745	4.96 (4.96-4.97)	465	2.85 (2.85-2.86)	1280	6.78 (6.77-6.79)
1985-1994	65-70	Recurrent depression (inpatient)	888	3.80 (3.79-3.80)	244	2.25 (2.25-2.26)	644	5.13 (5.12-5.14)
1995-2004	65-70	Recurrent depression (inpatient)	942	4.35 (4.34-4.35)	275	2.69 (2.68-2.69)	667	5.84 (5.82-5.85)
2005-2016	65-70	Recurrent depression (inpatient)	1230	3.59 (3.59-3.59)	475	2.87 (2.86-2.87)	755	4.27 (4.26-4.27)
1970-2016	70-75	Recurrent depression (inpatient)	4365	4.61 (4.61-4.62)	1153	2.71 (2.71-2.71)	3212	6.17 (6.16-6.17)
1970-1984	70-75	Recurrent depression (inpatient)	1370	4.77 (4.77-4.78)	283	2.25 (2.25-2.26)	1087	6.73 (6.72-6.74)
1985-1994	70-75	Recurrent depression (inpatient)	838	4.12 (4.11-4.12)	191	2.13 (2.12-2.13)	647	5.68 (5.67-5.69)
1995-2004	70-75	Recurrent depression (inpatient)	1032	5.38 (5.37-5.39)	284	3.29 (3.28-3.30)	748	7.09 (7.08-7.10)
2005-2016	70-75	Recurrent depression (inpatient)	1125	4.26 (4.26-4.27)	395	3.19 (3.18-3.19)	730	5.21 (5.20-5.22)
1970-2016	75-80	Recurrent depression (inpatient)	3332	4.62 (4.61-4.62)	816	2.70 (2.70-2.70)	2516	6.00 (5.99-6.01)
1970-1984	75-80	Recurrent depression (inpatient)	820	4.02 (4.02-4.03)	169	2.04 (2.03-2.04)	651	5.38 (5.37-5.39)
1985-1994	75-80	Recurrent depression (inpatient)	662	4.08 (4.08-4.09)	134	2.04 (2.03-2.04)	528	5.48 (5.47-5.49)
1995-2004	75-80	Recurrent depression (inpatient)	934	5.83 (5.82-5.83)	226	3.39 (3.38-3.40)	708	7.55 (7.54-7.57)
2005-2016	75-80	Recurrent depression (inpatient)	916	4.69 (4.69-4.70)	287	3.30 (3.30-3.31)	629	5.81 (5.80-5.82)
1970-2016	80-85	Recurrent depression (inpatient)	2003	4.15 (4.14-4.15)	451	2.47 (2.46-2.47)	1552	5.18 (5.17-5.18)
1970-1984	80-85	Recurrent depression (inpatient)	315	2.59 (2.58-2.59)	63	1.36 (1.36-1.36)	252	3.34 (3.34-3.35)
1985-1994	80-85	Recurrent depression (inpatient)	354	3.27 (3.26-3.27)	62	1.61 (1.60-1.61)	292	4.18 (4.17-4.19)
1995-2004	80-85	Recurrent depression (inpatient)	671	5.91 (5.90-5.92)	157	3.75 (3.74-3.76)	514	7.18 (7.16-7.19)
2005-2016	80-85	Recurrent depression (inpatient)	663	4.76 (4.75-4.77)	169	3.00 (3.00-3.01)	494	5.95 (5.94-5.96)
1970-2016	85-90	Recurrent depression (inpatient)	880	3.40 (3.40-3.40)	218	2.54 (2.54-2.55)	662	3.83 (3.82-3.83)
1970-1984	85-90	Recurrent depression (inpatient)	70	1.27 (1.26-1.27)	20	1.00 (1.00-1.01)	50	1.42 (1.41-1.42)
1985-1994	85-90	Recurrent depression (inpatient)	128	2.35 (2.34-2.36)	23	1.37 (1.36-1.38)	105	2.79 (2.78-2.80)
1995-2004	85-90	Recurrent depression (inpatient)	310	4.82 (4.81-4.83)	70	3.49 (3.47-3.51)	240	5.42 (5.40-5.44)

			Both sexes		Males		Females	
Years	Age	Mental disorder	Cases	IR (95% CI)	Cases	IR (95% CI)	Cases	IR (95% CI)
2005-2016	85-90	Recurrent depression (inpatient)	372	4.39 (4.38-4.40)	105	3.61 (3.60-3.62)	267	4.79 (4.78-4.80)
1970-2016	90-95	Recurrent depression (inpatient)	189	1.99 (1.99-2.00)	35	1.33 (1.32-1.33)	154	2.25 (2.24-2.26)
1970-1984	90-95	Recurrent depression (inpatient)	0-4	NA	0-4	NA	0-4	NA
1985-1994	90-95	Recurrent depression (inpatient)	11	0.59 (0.59-0.60)	0-4	NA	7-11	0.82 (0.81-0.82)
1995-2004	90-95	Recurrent depression (inpatient)	71	2.90 (2.88-2.91)	16	2.62 (2.60-2.64)	55	2.99 (2.97-3.00)
2005-2016	90-95	Recurrent depression (inpatient)	103	2.88 (2.87-2.89)	19	1.96 (1.95-1.97)	84	3.22 (3.21-3.23)
1970-2016	95-100	Recurrent depression (inpatient)	22	1.11 (1.11-1.12)	0-4	NA	18-22	1.17 (1.17-1.18)
1970-1984	95-100	Recurrent depression (inpatient)	0-4	NA	0-4	NA	0-4	NA
1985-1994	95-100	Recurrent depression (inpatient)	0-4	NA	0-4	NA	0-4	NA
1995-2004	95-100	Recurrent depression (inpatient)	0-4	NA	0-4	NA	0-4	NA
2005-2016	95-100	Recurrent depression (inpatient)	16	1.83 (1.82-1.84)	0-4	NA	12-16	1.87 (1.85-1.88)
1970-2016	10-15	Single and recurrent depression	4246	2.68 (2.68-2.69)	1527	1.89 (1.88-1.89)	2719	3.52 (3.52-3.52)
1970-1984	10-15	Single and recurrent depression	35	0.06 (0.06-0.06)	15	0.05 (0.05-0.05)	20	0.07 (0.07-0.07)
1985-1994	10-15	Single and recurrent depression	14	0.04 (0.04-0.04)	5	0.03 (0.03-0.03)	9	0.06 (0.06-0.06)
1995-2004	10-15	Single and recurrent depression	701	2.37 (2.37-2.37)	268	1.77 (1.77-1.77)	433	3.01 (3.00-3.01)
2005-2016	10-15	Single and recurrent depression	3496	8.72 (8.71-8.73)	1239	6.03 (6.02-6.04)	2257	11.55 (11.53-11.56)
1970-2016	15-20	Single and recurrent depression	17356	10.83 (10.82-10.83)	4836	5.87 (5.87-5.88)	12520	16.06 (16.05-16.08)
1970-1984	15-20	Single and recurrent depression	813	1.42 (1.42-1.43)	271	0.92 (0.92-0.93)	542	1.95 (1.95-1.95)
1985-1994	15-20	Single and recurrent depression	172	0.49 (0.49-0.49)	59	0.33 (0.33-0.33)	113	0.66 (0.66-0.66)
1995-2004	15-20	Single and recurrent depression	3053	10.75 (10.73-10.76)	819	5.63 (5.62-5.64)	2234	16.13 (16.10-16.15)
2005-2016	15-20	Single and recurrent depression	13318	33.73 (33.69-33.76)	3687	18.10 (18.08-18.13)	9631	50.37 (50.30-50.44)
1970-2016	20-25	Single and recurrent depression	20792	12.96 (12.96-12.97)	7209	8.67 (8.67-8.68)	13583	17.58 (17.57-17.59)
1970-1984	20-25	Single and recurrent depression	2441	4.40 (4.40-4.41)	915	3.19 (3.19-3.20)	1526	5.70 (5.69-5.70)
1985-1994	20-25	Single and recurrent depression	526	1.41 (1.41-1.41)	213	1.10 (1.10-1.10)	313	1.75 (1.75-1.75)
1995-2004	20-25	Single and recurrent depression	4517	14.63 (14.61-14.64)	1434	8.97 (8.95-8.98)	3083	20.71 (20.67-20.74)
2005-2016	20-25	Single and recurrent depression	13308	36.20 (36.16-36.23)	4647	24.38 (24.35-24.42)	8661	48.91 (48.84-48.99)
1970-2016	25-30	Single and recurrent depression	19910	12.39 (12.38-12.39)	7358	8.86 (8.85-8.87)	12552	16.16 (16.15-16.17)
1970-1984	25-30	Single and recurrent depression	3777	6.68 (6.68-6.69)	1381	4.75 (4.74-4.75)	2396	8.73 (8.72-8.74)
1985-1994	25-30	Single and recurrent depression	785	2.13 (2.13-2.13)	295	1.54 (1.54-1.55)	490	2.76 (2.76-2.77)
1995-2004	25-30	Single and recurrent depression	4983	14.86 (14.84-14.87)	1829	10.52 (10.51-10.54)	3154	19.52 (19.49-19.55)
2005-2016	25-30	Single and recurrent depression	10365	30.65 (30.62-30.69)	3853	22.06 (22.02-22.09)	6512	39.84 (39.78-39.91)
1970-2016	30-35	Single and recurrent depression	20693	13.00 (13.00-13.01)	7895	9.65 (9.65-9.66)	12798	16.55 (16.53-16.56)
1970-1984	30-35	Single and recurrent depression	4659	8.62 (8.61-8.63)	1700	6.16 (6.15-6.17)	2959	11.19 (11.17-11.20)
1985-1994	30-35	Single and recurrent depression	1135	3.29 (3.28-3.29)	412	2.32 (2.31-2.32)	723	4.32 (4.31-4.32)
1995-2004	30-35	Single and recurrent depression	5311	14.81 (14.80-14.83)	2088	11.28 (11.26-11.29)	3223	18.60 (18.57-18.62)
2005-2016	30-35	Single and recurrent depression	9588	27.62 (27.59-27.65)	3695	20.66 (20.63-20.69)	5893	35.03 (34.98-35.08)
1970-2016	35-40	Single and recurrent depression	21795	13.86 (13.85-13.87)	8419	10.48 (10.47-10.49)	13376	17.39 (17.37-17.40)
1970-1984	35-40	Single and recurrent depression	5311	10.81 (10.80-10.82)	1851	7.43 (7.42-7.44)	3460	14.29 (14.27-14.31)
1985-1994	35-40	Single and recurrent depression	1430	4.11 (4.11-4.12)	513	2.89 (2.89-2.90)	917	5.38 (5.37-5.39)
1995-2004	35-40	Single and recurrent depression	5703	15.97 (15.95-15.98)	2403	13.12 (13.10-13.14)	3300	18.97 (18.94-19.00)

			Both sexes		Males		Females	
**Years**	**Age**	**Mental disorder**	**Cases**	**IR (95% CI)**	**Cases**	**IR (95% CI)**	**Cases**	**IR (95% CI)**
2005-2016	35-40	Single and recurrent depression	9351	24.83 (24.81-24.86)	3652	18.86 (18.83-18.88)	5699	31.16 (31.12-31.21)
1970-2016	40-45	Single and recurrent depression	21759	14.16 (14.15-14.17)	8764	11.24 (11.23-11.25)	12995	17.17 (17.16-17.18)
1970-1984	40-45	Single and recurrent depression	5573	12.96 (12.95-12.97)	1933	8.95 (8.94-8.97)	3640	16.99 (16.97-17.02)
1985-1994	40-45	Single and recurrent depression	1762	4.83 (4.82-4.83)	702	3.80 (3.79-3.80)	1060	5.88 (5.87-5.89)
1995-2004	40-45	Single and recurrent depression	5329	15.90 (15.88-15.91)	2296	13.44 (13.42-13.46)	3033	18.45 (18.43-18.48)
2005-2016	40-45	Single and recurrent depression	9095	22.40 (22.38-22.42)	3833	18.43 (18.40-18.45)	5262	26.57 (26.54-26.61)
1970-2016	45-50	Single and recurrent depression	21837	14.64 (14.63-14.65)	8773	11.68 (11.67-11.68)	13064	17.64 (17.63-17.66)
1970-1984	45-50	Single and recurrent depression	6216	14.95 (14.94-14.96)	2193	10.61 (10.59-10.62)	4023	19.24 (19.21-19.27)
1985-1994	45-50	Single and recurrent depression	1916	5.74 (5.73-5.75)	707	4.20 (4.19-4.21)	1209	7.30 (7.29-7.31)
1995-2004	45-50	Single and recurrent depression	5008	14.95 (14.94-14.97)	2119	12.52 (12.51-12.54)	2889	17.43 (17.40-17.45)
2005-2016	45-50	Single and recurrent depression	8697	21.35 (21.33-21.38)	3754	18.12 (18.09-18.14)	4943	24.71 (24.67-24.74)
1970-2016	50-55	Single and recurrent depression	20387	14.30 (14.29-14.30)	8141	11.43 (11.42-11.43)	12246	17.16 (17.15-17.18)
1970-1984	50-55	Single and recurrent depression	6695	16.02 (16.00-16.03)	2279	11.05 (11.03-11.06)	4416	20.87 (20.84-20.89)
1985-1994	50-55	Single and recurrent depression	1751	6.36 (6.35-6.37)	632	4.60 (4.60-4.61)	1119	8.11 (8.10-8.12)
1995-2004	50-55	Single and recurrent depression	4845	13.96 (13.94-13.97)	2030	11.66 (11.64-11.68)	2815	16.27 (16.25-16.30)
2005-2016	50-55	Single and recurrent depression	7096	18.40 (18.38-18.42)	3200	16.42 (16.40-16.45)	3896	20.42 (20.39-20.44)
1970-2016	55-60	Single and recurrent depression	17793	13.21 (13.20-13.22)	7057	10.59 (10.59-10.60)	10736	15.77 (15.76-15.78)
1970-1984	55-60	Single and recurrent depression	6237	15.11 (15.09-15.12)	2095	10.38 (10.36-10.39)	4142	19.63 (19.60-19.65)
1985-1994	55-60	Single and recurrent depression	1726	6.94 (6.93-6.95)	592	4.84 (4.84-4.85)	1134	8.96 (8.95-8.98)
1995-2004	55-60	Single and recurrent depression	4073	13.07 (13.06-13.09)	1714	11.04 (11.02-11.06)	2359	15.09 (15.07-15.12)
2005-2016	55-60	Single and recurrent depression	5757	15.41 (15.39-15.42)	2656	14.22 (14.20-14.24)	3101	16.60 (16.57-16.62)
1970-2016	60-65	Single and recurrent depression	14402	11.45 (11.44-11.46)	5387	8.81 (8.80-8.81)	9015	13.95 (13.94-13.97)
1970-1984	60-65	Single and recurrent depression	5460	13.88 (13.87-13.90)	1731	9.16 (9.15-9.17)	3729	18.26 (18.23-18.28)
1985-1994	60-65	Single and recurrent depression	1824	7.57 (7.56-7.58)	599	5.17 (5.16-5.18)	1225	9.80 (9.78-9.81)
1995-2004	60-65	Single and recurrent depression	2981	11.98 (11.97-12.00)	1168	9.60 (9.58-9.62)	1813	14.26 (14.24-14.29)
2005-2016	60-65	Single and recurrent depression	4137	11.04 (11.02-11.05)	1889	10.20 (10.18-10.21)	2248	11.86 (11.84-11.87)
1970-2016	65-70	Single and recurrent depression	12743	11.31 (11.30-11.32)	4344	8.15 (8.14-8.16)	8399	14.15 (14.14-14.16)
1970-1984	65-70	Single and recurrent depression	4441	12.73 (12.72-12.74)	1293	7.98 (7.97-8.00)	3148	16.84 (16.82-16.87)
1985-1994	65-70	Single and recurrent depression	1788	7.77 (7.76-7.78)	537	5.01 (5.00-5.02)	1251	10.17 (10.15-10.19)
1995-2004	65-70	Single and recurrent depression	2980	14.02 (14.00-14.04)	1055	10.45 (10.43-10.47)	1925	17.26 (17.22-17.29)
2005-2016	65-70	Single and recurrent depression	3534	10.55 (10.53-10.56)	1459	8.95 (8.94-8.97)	2075	12.05 (12.03-12.07)
1970-2016	70-75	Single and recurrent depression	11990	12.87 (12.86-12.88)	3799	9.03 (9.02-9.04)	8191	16.04 (16.03-16.06)
1970-1984	70-75	Single and recurrent depression	3354	11.77 (11.76-11.79)	853	6.83 (6.81-6.84)	2501	15.64 (15.61-15.66)
1985-1994	70-75	Single and recurrent depression	1664	8.29 (8.28-8.30)	433	4.87 (4.86-4.88)	1231	11.02 (11.00-11.04)
1995-2004	70-75	Single and recurrent depression	3542	18.84 (18.82-18.87)	1160	13.62 (13.60-13.65)	2382	23.17 (23.12-23.21)
2005-2016	70-75	Single and recurrent depression	3430	13.30 (13.28-13.32)	1353	11.11 (11.09-11.13)	2077	15.26 (15.24-15.29)
1970-2016	75-80	Single and recurrent depression	11233	15.83 (15.82-15.84)	3415	11.42 (11.41-11.44)	7818	19.03 (19.01-19.05)
1970-1984	75-80	Single and recurrent depression	2084	10.29 (10.27-10.30)	555	6.71 (6.70-6.73)	1529	12.75 (12.72-12.77)
1985-1994	75-80	Single and recurrent depression	1438	8.99 (8.97-9.00)	357	5.47 (5.46-5.48)	1081	11.41 (11.39-11.43)
1995-2004	75-80	Single and recurrent depression	4132	26.35 (26.31-26.40)	1256	19.13 (19.09-19.18)	2876	31.56 (31.49-31.62)

			Both sexes		Males		Females	
Years	Age	Mental disorder	Cases	IR (95% CI)	Cases	IR (95% CI)	Cases	IR (95% CI)
2005-2016	75-80	Single and recurrent depression	3579	18.80 (18.78-18.83)	1247	14.60 (14.57-14.63)	2332	22.22 (22.18-22.27)
1970-2016	80-85	Single and recurrent depression	8706	18.36 (18.34-18.38)	2568	14.20 (14.18-14.22)	6138	20.92 (20.90-20.95)
1970-1984	80-85	Single and recurrent depression	852	7.04 (7.03-7.05)	219	4.74 (4.73-4.76)	633	8.46 (8.44-8.48)
1985-1994	80-85	Single and recurrent depression	875	8.17 (8.15-8.18)	204	5.33 (5.31-5.34)	671	9.75 (9.73-9.77)
1995-2004	80-85	Single and recurrent depression	3618	32.65 (32.59-32.71)	1052	25.52 (25.45-25.60)	2566	36.87 (36.79-36.96)
2005-2016	80-85	Single and recurrent depression	3361	24.84 (24.80-24.89)	1093	19.81 (19.76-19.87)	2268	28.31 (28.25-28.37)
1970-2016	85-90	Single and recurrent depression	5056	19.93 (19.90-19.95)	1293	15.25 (15.22-15.29)	3763	22.27 (22.24-22.31)
1970-1984	85-90	Single and recurrent depression	247	4.49 (4.48-4.50)	77	3.88 (3.86-3.89)	170	4.84 (4.82-4.85)
1985-1994	85-90	Single and recurrent depression	314	5.82 (5.81-5.84)	55	3.29 (3.28-3.31)	259	6.96 (6.93-6.98)
1995-2004	85-90	Single and recurrent depression	2208	35.16 (35.08-35.25)	535	27.08 (26.97-27.20)	1673	38.87 (38.76-38.99)
2005-2016	85-90	Single and recurrent depression	2287	27.89 (27.83-27.95)	626	21.99 (21.91-22.07)	1661	31.02 (30.94-31.10)
1970-2016	90-95	Single and recurrent depression	1665	17.93 (17.89-17.96)	365	14.00 (13.94-14.05)	1300	19.46 (19.42-19.51)
1970-1984	90-95	Single and recurrent depression	24	1.50 (1.50-1.51)	0-4	NA	20-24	1.91 (1.90-1.93)
1985-1994	90-95	Single and recurrent depression	58	3.15 (3.13-3.16)	12	2.38 (2.35-2.40)	46	3.44 (3.42-3.46)
1995-2004	90-95	Single and recurrent depression	711	29.65 (29.53-29.77)	144	23.89 (23.70-24.08)	567	31.58 (31.44-31.73)
2005-2016	90-95	Single and recurrent depression	872	25.26 (25.18-25.35)	205	21.59 (21.46-21.73)	667	26.65 (26.55-26.76)
1970-2016	95-100	Single and recurrent depression	282	14.54 (14.47-14.60)	53	12.06 (11.95-12.17)	229	15.26 (15.19-15.34)
1970-1984	95-100	Single and recurrent depression	0-4	NA	0-4	NA	0-4	NA
1985-1994	95-100	Single and recurrent depression	0-4	NA	0-4	NA	0-4	NA
1995-2004	95-100	Single and recurrent depression	108	21.87 (21.68-22.07)	20	20.33 (19.93-20.74)	88	22.26 (22.04-22.48)
2005-2016	95-100	Single and recurrent depression	170	20.15 (20.01-20.29)	32	18.44 (18.17-18.72)	138	20.59 (20.44-20.75)
1970-2016	10-15	Single and recurrent depression (inpatient)	780	0.49 (0.49-0.49)	230	0.28 (0.28-0.28)	550	0.71 (0.71-0.71)
1970-1984	10-15	Single and recurrent depression (inpatient)	35	0.06 (0.06-0.06)	15	0.05 (0.05-0.05)	20	0.07 (0.07-0.07)
1985-1994	10-15	Single and recurrent depression (inpatient)	11	0.03 (0.03-0.03)	5	0.03 (0.03-0.03)	6	0.04 (0.04-0.04)
1995-2004	10-15	Single and recurrent depression (inpatient)	144	0.49 (0.49-0.49)	49	0.32 (0.32-0.32)	95	0.66 (0.66-0.66)
2005-2016	10-15	Single and recurrent depression (inpatient)	590	1.47 (1.47-1.47)	161	0.78 (0.78-0.78)	429	2.19 (2.19-2.20)
1970-2016	15-20	Single and recurrent depression (inpatient)	4709	2.93 (2.93-2.93)	1332	1.62 (1.61-1.62)	3377	4.32 (4.32-4.32)
1970-1984	15-20	Single and recurrent depression (inpatient)	813	1.42 (1.42-1.43)	271	0.92 (0.92-0.93)	542	1.95 (1.95-1.95)
1985-1994	15-20	Single and recurrent depression (inpatient)	165	0.47 (0.47-0.47)	57	0.31 (0.31-0.32)	108	0.63 (0.63-0.63)
1995-2004	15-20	Single and recurrent depression (inpatient)	786	2.76 (2.76-2.77)	204	1.40 (1.40-1.40)	582	4.19 (4.19-4.20)
2005-2016	15-20	Single and recurrent depression (inpatient)	2945	7.40 (7.39-7.41)	800	3.91 (3.90-3.92)	2145	11.09 (11.08-11.11)
1970-2016	20-25	Single and recurrent depression (inpatient)	7794	4.84 (4.84-4.84)	2778	3.33 (3.33-3.34)	5016	6.45 (6.45-6.45)
1970-1984	20-25	Single and recurrent depression (inpatient)	2441	4.40 (4.40-4.41)	915	3.19 (3.19-3.20)	1526	5.70 (5.69-5.70)
1985-1994	20-25	Single and recurrent depression (inpatient)	495	1.33 (1.33-1.33)	197	1.01 (1.01-1.02)	298	1.67 (1.66-1.67)
1995-2004	20-25	Single and recurrent depression (inpatient)	1440	4.65 (4.64-4.65)	462	2.88 (2.88-2.89)	978	6.54 (6.53-6.55)
2005-2016	20-25	Single and recurrent depression (inpatient)	3418	9.14 (9.13-9.15)	1204	6.26 (6.25-6.26)	2214	12.21 (12.19-12.23)
1970-2016	25-30	Single and recurrent depression (inpatient)	9081	5.62 (5.62-5.62)	3416	4.10 (4.10-4.10)	5665	7.24 (7.24-7.25)
1970-1984	25-30	Single and recurrent depression (inpatient)	3777	6.68 (6.68-6.69)	1381	4.75 (4.74-4.75)	2396	8.73 (8.72-8.74)
1985-1994	25-30	Single and recurrent depression (inpatient)	737	2.00 (2.00-2.00)	288	1.51 (1.50-1.51)	449	2.53 (2.53-2.53)
1995-2004	25-30	Single and recurrent depression (inpatient)	1758	5.22 (5.22-5.23)	660	3.79 (3.78-3.79)	1098	6.76 (6.75-6.77)

			Both sexes		Males		Females	
Years	Age	Mental disorder	Cases	IR (95% CI)	Cases	IR (95% CI)	Cases	IR (95% CI)
2005-2016	25-30	Single and recurrent depression (inpatient)	2809	8.14 (8.13-8.15)	1087	6.14 (6.13-6.15)	1722	10.24 (10.23-10.26)
1970-2016	30-35	Single and recurrent depression (inpatient)	10848	6.78 (6.78-6.78)	4205	5.12 (5.12-5.13)	6643	8.53 (8.52-8.53)
1970-1984	30-35	Single and recurrent depression (inpatient)	4659	8.62 (8.61-8.63)	1700	6.16 (6.15-6.17)	2959	11.19 (11.17-11.20)
1985-1994	30-35	Single and recurrent depression (inpatient)	1084	3.14 (3.14-3.14)	397	2.23 (2.23-2.23)	687	4.10 (4.10-4.11)
1995-2004	30-35	Single and recurrent depression (inpatient)	2186	6.08 (6.07-6.08)	900	4.85 (4.84-4.85)	1286	7.39 (7.38-7.40)
2005-2016	30-35	Single and recurrent depression (inpatient)	2919	8.24 (8.23-8.25)	1208	6.66 (6.66-6.67)	1711	9.90 (9.88-9.91)
1970-2016	35-40	Single and recurrent depression (inpatient)	12205	7.72 (7.72-7.72)	4707	5.84 (5.83-5.84)	7498	9.68 (9.67-9.68)
1970-1984	35-40	Single and recurrent depression (inpatient)	5311	10.81 (10.80-10.82)	1851	7.43 (7.42-7.44)	3460	14.29 (14.27-14.31)
1985-1994	35-40	Single and recurrent depression (inpatient)	1377	3.96 (3.96-3.96)	497	2.80 (2.80-2.81)	880	5.16 (5.15-5.17)
1995-2004	35-40	Single and recurrent depression (inpatient)	2508	7.00 (6.99-7.00)	1081	5.88 (5.88-5.89)	1427	8.17 (8.15-8.18)
2005-2016	35-40	Single and recurrent depression (inpatient)	3009	7.84 (7.83-7.85)	1278	6.51 (6.50-6.52)	1731	9.23 (9.22-9.25)
1970-2016	40-45	Single and recurrent depression (inpatient)	12978	8.40 (8.40-8.41)	5224	6.67 (6.67-6.68)	7754	10.18 (10.17-10.18)
1970-1984	40-45	Single and recurrent depression (inpatient)	5573	12.96 (12.95-12.97)	1933	8.95 (8.94-8.97)	3640	16.99 (16.97-17.02)
1985-1994	40-45	Single and recurrent depression (inpatient)	1678	4.60 (4.59-4.60)	671	3.63 (3.63-3.64)	1007	5.58 (5.58-5.59)
1995-2004	40-45	Single and recurrent depression (inpatient)	2491	7.40 (7.40-7.41)	1111	6.48 (6.47-6.49)	1380	8.36 (8.35-8.37)
2005-2016	40-45	Single and recurrent depression (inpatient)	3236	7.83 (7.83-7.84)	1509	7.16 (7.15-7.17)	1727	8.53 (8.52-8.54)
1970-2016	45-50	Single and recurrent depression (inpatient)	13681	9.12 (9.12-9.13)	5408	7.17 (7.16-7.17)	8273	11.10 (11.09-11.11)
1970-1984	45-50	Single and recurrent depression (inpatient)	6216	14.95 (14.94-14.96)	2193	10.61 (10.59-10.62)	4023	19.24 (19.21-19.27)
1985-1994	45-50	Single and recurrent depression (inpatient)	1823	5.46 (5.45-5.47)	677	4.02 (4.02-4.03)	1146	6.92 (6.91-6.93)
1995-2004	45-50	Single and recurrent depression (inpatient)	2392	7.12 (7.11-7.12)	1025	6.04 (6.03-6.05)	1367	8.21 (8.20-8.22)
2005-2016	45-50	Single and recurrent depression (inpatient)	3250	7.85 (7.84-7.86)	1513	7.21 (7.20-7.22)	1737	8.51 (8.50-8.52)
1970-2016	50-55	Single and recurrent depression (inpatient)	13697	9.56 (9.55-9.56)	5273	7.37 (7.37-7.38)	8424	11.74 (11.73-11.75)
1970-1984	50-55	Single and recurrent depression (inpatient)	6695	16.02 (16.00-16.03)	2279	11.05 (11.03-11.06)	4416	20.87 (20.84-20.89)
1985-1994	50-55	Single and recurrent depression (inpatient)	1688	6.13 (6.12-6.14)	609	4.44 (4.43-4.44)	1079	7.82 (7.81-7.83)
1995-2004	50-55	Single and recurrent depression (inpatient)	2480	7.12 (7.11-7.13)	1013	5.80 (5.79-5.81)	1467	8.45 (8.43-8.46)
2005-2016	50-55	Single and recurrent depression (inpatient)	2834	7.24 (7.23-7.25)	1372	6.96 (6.95-6.97)	1462	7.53 (7.51-7.54)
1970-2016	55-60	Single and recurrent depression (inpatient)	12515	9.25 (9.25-9.26)	4753	7.11 (7.10-7.12)	7762	11.35 (11.34-11.35)
1970-1984	55-60	Single and recurrent depression (inpatient)	6237	15.11 (15.09-15.12)	2095	10.38 (10.36-10.39)	4142	19.63 (19.60-19.65)
1985-1994	55-60	Single and recurrent depression (inpatient)	1673	6.73 (6.72-6.73)	573	4.69 (4.68-4.70)	1100	8.69 (8.68-8.71)
1995-2004	55-60	Single and recurrent depression (inpatient)	2107	6.74 (6.73-6.75)	887	5.70 (5.69-5.71)	1220	7.77 (7.76-7.79)
2005-2016	55-60	Single and recurrent depression (inpatient)	2498	6.60 (6.59-6.61)	1198	6.35 (6.34-6.36)	1300	6.85 (6.84-6.86)
1970-2016	60-65	Single and recurrent depression (inpatient)	10906	8.64 (8.63-8.64)	3914	6.38 (6.37-6.38)	6992	10.78 (10.77-10.79)
1970-1984	60-65	Single and recurrent depression (inpatient)	5460	13.88 (13.87-13.90)	1731	9.16 (9.15-9.17)	3729	18.26 (18.23-18.28)
1985-1994	60-65	Single and recurrent depression (inpatient)	1784	7.41 (7.40-7.42)	587	5.07 (5.06-5.08)	1197	9.57 (9.56-9.59)
1995-2004	60-65	Single and recurrent depression (inpatient)	1569	6.29 (6.28-6.30)	627	5.14 (5.13-5.15)	942	7.38 (7.37-7.40)
2005-2016	60-65	Single and recurrent depression (inpatient)	2093	5.53 (5.52-5.53)	969	5.19 (5.18-5.19)	1124	5.86 (5.85-5.87)
1970-2016	65-70	Single and recurrent depression (inpatient)	9501	8.41 (8.40-8.41)	3113	5.83 (5.82-5.83)	6388	10.72 (10.71-10.73)
1970-1984	65-70	Single and recurrent depression (inpatient)	4441	12.73 (12.72-12.74)	1293	7.98 (7.97-8.00)	3148	16.84 (16.82-16.87)
1985-1994	65-70	Single and recurrent depression (inpatient)	1726	7.50 (7.49-7.51)	520	4.85 (4.84-4.86)	1206	9.80 (9.78-9.82)
1995-2004	65-70	Single and recurrent depression (inpatient)	1528	7.17 (7.16-7.18)	533	5.27 (5.26-5.28)	995	8.89 (8.87-8.90)

			Both sexes		Males		Females	
Years	Age	Mental disorder	Cases	IR (95% CI)	Cases	IR (95% CI)	Cases	IR (95% CI)
2005-2016	65-70	Single and recurrent depression (inpatient)	1806	5.34 (5.34-5.35)	767	4.67 (4.67-4.68)	1039	5.97 (5.96-5.98)
1970-2016	70-75	Single and recurrent depression (inpatient)	8155	8.73 (8.72-8.73)	2427	5.75 (5.75-5.76)	5728	11.18 (11.17-11.19)
1970-1984	70-75	Single and recurrent depression (inpatient)	3354	11.77 (11.76-11.79)	853	6.83 (6.81-6.84)	2501	15.64 (15.61-15.66)
1985-1994	70-75	Single and recurrent depression (inpatient)	1592	7.93 (7.92-7.95)	415	4.67 (4.66-4.68)	1177	10.53 (10.51-10.55)
1995-2004	70-75	Single and recurrent depression (inpatient)	1619	8.58 (8.57-8.59)	516	6.05 (6.03-6.06)	1103	10.68 (10.66-10.70)
2005-2016	70-75	Single and recurrent depression (inpatient)	1590	6.11 (6.11-6.12)	643	5.24 (5.24-5.25)	947	6.89 (6.88-6.90)
1970-2016	75-80	Single and recurrent depression (inpatient)	6556	9.20 (9.20-9.21)	1948	6.50 (6.49-6.51)	4608	11.17 (11.16-11.18)
1970-1984	75-80	Single and recurrent depression (inpatient)	2084	10.29 (10.27-10.30)	555	6.71 (6.70-6.73)	1529	12.75 (12.72-12.77)
1985-1994	75-80	Single and recurrent depression (inpatient)	1367	8.54 (8.53-8.56)	341	5.22 (5.21-5.24)	1026	10.83 (10.81-10.85)
1995-2004	75-80	Single and recurrent depression (inpatient)	1701	10.79 (10.77-10.81)	532	8.08 (8.06-8.09)	1169	12.74 (12.71-12.76)
2005-2016	75-80	Single and recurrent depression (inpatient)	1404	7.31 (7.30-7.32)	520	6.05 (6.04-6.06)	884	8.32 (8.31-8.34)
1970-2016	80-85	Single and recurrent depression (inpatient)	3997	8.38 (8.37-8.39)	1156	6.37 (6.36-6.38)	2841	9.62 (9.61-9.63)
1970-1984	80-85	Single and recurrent depression (inpatient)	852	7.04 (7.03-7.05)	219	4.74 (4.73-4.76)	633	8.46 (8.44-8.48)
1985-1994	80-85	Single and recurrent depression (inpatient)	814	7.60 (7.58-7.61)	190	4.96 (4.94-4.98)	624	9.07 (9.05-9.09)
1995-2004	80-85	Single and recurrent depression (inpatient)	1250	11.19 (11.17-11.22)	387	9.34 (9.32-9.37)	863	12.29 (12.26-12.31)
2005-2016	80-85	Single and recurrent depression (inpatient)	1081	7.89 (7.87-7.90)	360	6.47 (6.45-6.49)	721	8.86 (8.84-8.87)
1970-2016	85-90	Single and recurrent depression (inpatient)	1778	6.95 (6.94-6.96)	486	5.70 (5.69-5.72)	1292	7.58 (7.56-7.59)
1970-1984	85-90	Single and recurrent depression (inpatient)	247	4.49 (4.48-4.50)	77	3.88 (3.86-3.89)	170	4.84 (4.82-4.85)
1985-1994	85-90	Single and recurrent depression (inpatient)	283	5.25 (5.23-5.26)	50	2.99 (2.98-3.01)	233	6.26 (6.24-6.28)
1995-2004	85-90	Single and recurrent depression (inpatient)	604	9.53 (9.50-9.55)	161	8.10 (8.06-8.13)	443	10.18 (10.15-10.21)
2005-2016	85-90	Single and recurrent depression (inpatient)	644	7.72 (7.70-7.74)	198	6.88 (6.86-6.91)	446	8.16 (8.14-8.18)
1970-2016	90-95	Single and recurrent depression (inpatient)	408	4.35 (4.34-4.36)	97	3.70 (3.68-3.71)	311	4.60 (4.59-4.61)
1970-1984	90-95	Single and recurrent depression (inpatient)	24	1.50 (1.50-1.51)	0-4	NA	20-24	1.91 (1.90-1.93)
1985-1994	90-95	Single and recurrent depression (inpatient)	56	3.04 (3.03-3.05)	12	2.38 (2.35-2.40)	44	3.29 (3.27-3.31)
1995-2004	90-95	Single and recurrent depression (inpatient)	143	5.90 (5.88-5.93)	33	5.44 (5.40-5.48)	110	6.06 (6.03-6.09)
2005-2016	90-95	Single and recurrent depression (inpatient)	185	5.25 (5.23-5.27)	48	4.99 (4.96-5.03)	137	5.35 (5.33-5.37)
1970-2016	95-100	Single and recurrent depression (inpatient)	46	2.34 (2.33-2.36)	13	2.94 (2.91-2.97)	33	2.17 (2.16-2.18)
1970-1984	95-100	Single and recurrent depression (inpatient)	0-4	NA	0-4	NA	0-4	NA
1985-1994	95-100	Single and recurrent depression (inpatient)	0-4	NA	0-4	NA	0-4	NA
1995-2004	95-100	Single and recurrent depression (inpatient)	16	3.21 (3.19-3.24)	6	6.07 (5.95-6.19)	10	2.51 (2.48-2.53)
2005-2016	95-100	Single and recurrent depression (inpatient)	26	3.02 (3.00-3.04)	6	3.41 (3.36-3.46)	20	2.92 (2.90-2.94)
1970-2016	5-10	Anxiety disorder	4574	2.93 (2.93-2.94)	2835	3.55 (3.55-3.55)	1739	2.29 (2.28-2.29)
1970-1984	5-10	Anxiety disorder	39	0.07 (0.07-0.07)	30	0.11 (0.11-0.11)	9	0.03 (0.03-0.03)
1985-1994	5-10	Anxiety disorder	61	0.22 (0.22-0.22)	40	0.28 (0.28-0.28)	21	0.15 (0.15-0.15)
1995-2004	5-10	Anxiety disorder	1182	3.59 (3.59-3.60)	716	4.24 (4.23-4.25)	466	2.91 (2.90-2.91)
2005-2016	5-10	Anxiety disorder	3292	8.38 (8.38-8.39)	2049	10.19 (10.18-10.21)	1243	6.49 (6.48-6.50)
1970-2016	10-15	Anxiety disorder	17827	11.30 (11.29-11.30)	7155	8.86 (8.85-8.86)	10672	13.86 (13.85-13.86)
1970-1984	10-15	Anxiety disorder	315	0.55 (0.55-0.55)	105	0.36 (0.36-0.36)	210	0.76 (0.75-0.76)
1985-1994	10-15	Anxiety disorder	213	0.68 (0.67-0.68)	83	0.52 (0.51-0.52)	130	0.84 (0.84-0.84)
1995-2004	10-15	Anxiety disorder	3648	12.37 (12.36-12.39)	1490	9.86 (9.84-9.87)	2158	15.02 (14.99-15.04)

			Both sexes		Males		Females	
**Years**	**Age**	**Mental disorder**	**Cases**	**IR (95% CI)**	**Cases**	**IR (95% CI)**	**Cases**	**IR (95% CI)**
2005-2016	10-15	Anxiety disorder	13651	34.30 (34.27-34.34)	5477	26.87 (26.83-26.91)	8174	42.11 (42.05-42.17)
1970-2016	15-20	Anxiety disorder	41063	25.78 (25.77-25.79)	13061	15.93 (15.92-15.94)	28002	36.22 (36.20-36.25)
1970-1984	15-20	Anxiety disorder	2926	5.13 (5.13-5.13)	1005	3.43 (3.43-3.43)	1921	6.93 (6.92-6.94)
1985-1994	15-20	Anxiety disorder	1163	3.30 (3.29-3.30)	417	2.30 (2.30-2.31)	746	4.34 (4.34-4.35)
1995-2004	15-20	Anxiety disorder	9060	32.11 (32.07-32.14)	2636	18.19 (18.16-18.22)	6424	46.79 (46.72-46.87)
2005-2016	15-20	Anxiety disorder	27914	72.06 (71.99-72.13)	9003	44.85 (44.79-44.91)	18911	101.33 (101.19-101.48)
1970-2016	20-25	Anxiety disorder	40746	25.64 (25.63-25.66)	16113	19.52 (19.51-19.54)	24633	32.26 (32.23-32.28)
1970-1984	20-25	Anxiety disorder	5373	9.72 (9.71-9.73)	2241	7.84 (7.83-7.85)	3132	11.73 (11.71-11.74)
1985-1994	20-25	Anxiety disorder	2186	5.88 (5.87-5.88)	977	5.04 (5.03-5.05)	1209	6.78 (6.77-6.79)
1995-2004	20-25	Anxiety disorder	10122	33.08 (33.05-33.12)	3617	22.75 (22.72-22.79)	6505	44.26 (44.19-44.33)
2005-2016	20-25	Anxiety disorder	23065	64.40 (64.34-64.47)	9278	49.68 (49.61-49.75)	13787	80.45 (80.32-80.57)
1970-2016	25-30	Anxiety disorder	36685	23.06 (23.05-23.07)	15501	18.82 (18.81-18.83)	21184	27.62 (27.60-27.64)
1970-1984	25-30	Anxiety disorder	6722	11.93 (11.92-11.94)	2736	9.43 (9.42-9.44)	3986	14.59 (14.57-14.60)
1985-1994	25-30	Anxiety disorder	2602	7.10 (7.09-7.11)	1103	5.80 (5.79-5.80)	1499	8.50 (8.49-8.52)
1995-2004	25-30	Anxiety disorder	10299	31.05 (31.01-31.08)	4124	23.92 (23.88-23.95)	6175	38.76 (38.70-38.82)
2005-2016	25-30	Anxiety disorder	17062	51.86 (51.81-51.92)	7538	44.11 (44.05-44.18)	9524	60.24 (60.14-60.33)
1970-2016	30-35	Anxiety disorder	35855	22.79 (22.78-22.80)	15171	18.72 (18.71-18.73)	20684	27.10 (27.08-27.12)
1970-1984	30-35	Anxiety disorder	7184	13.34 (13.33-13.35)	2760	10.03 (10.02-10.04)	4424	16.79 (16.77-16.82)
1985-1994	30-35	Anxiety disorder	2976	8.69 (8.68-8.70)	1279	7.24 (7.23-7.25)	1697	10.23 (10.22-10.25)
1995-2004	30-35	Anxiety disorder	10409	29.39 (29.36-29.42)	4463	24.34 (24.30-24.37)	5946	34.82 (34.77-34.88)
2005-2016	30-35	Anxiety disorder	15286	45.19 (45.14-45.23)	6669	38.08 (38.02-38.14)	8617	52.81 (52.73-52.89)
1970-2016	35-40	Anxiety disorder	34581	22.26 (22.25-22.27)	14529	18.27 (18.26-18.29)	20052	26.44 (26.42-26.46)
1970-1984	35-40	Anxiety disorder	6898	14.09 (14.07-14.10)	2570	10.34 (10.33-10.36)	4328	17.94 (17.92-17.96)
1985-1994	35-40	Anxiety disorder	2829	8.21 (8.20-8.22)	1135	6.45 (6.44-6.46)	1694	10.05 (10.03-10.06)
1995-2004	35-40	Anxiety disorder	10000	28.41 (28.38-28.44)	4221	23.31 (23.28-23.35)	5779	33.82 (33.77-33.87)
2005-2016	35-40	Anxiety disorder	14854	40.44 (40.40-40.48)	6603	34.82 (34.77-34.87)	8251	46.44 (46.38-46.51)
1970-2016	40-45	Anxiety disorder	32040	21.11 (21.10-21.12)	13337	17.29 (17.27-17.30)	18703	25.07 (25.05-25.09)
1970-1984	40-45	Anxiety disorder	6230	14.51 (14.50-14.52)	2127	9.87 (9.85-9.88)	4103	19.19 (19.17-19.22)
1985-1994	40-45	Anxiety disorder	2879	7.95 (7.94-7.96)	1081	5.89 (5.88-5.90)	1798	10.08 (10.06-10.09)
1995-2004	40-45	Anxiety disorder	8561	25.94 (25.91-25.97)	3636	21.54 (21.51-21.57)	4925	30.54 (30.49-30.59)
2005-2016	40-45	Anxiety disorder	14370	36.28 (36.25-36.32)	6493	31.89 (31.85-31.93)	7877	40.93 (40.87-40.99)
1970-2016	45-50	Anxiety disorder	27577	18.70 (18.69-18.70)	11494	15.44 (15.43-15.45)	16083	22.01 (22.00-22.03)
1970-1984	45-50	Anxiety disorder	5768	13.87 (13.86-13.89)	2009	9.72 (9.71-9.73)	3759	17.98 (17.95-18.00)
1985-1994	45-50	Anxiety disorder	2356	7.10 (7.10-7.11)	873	5.21 (5.21-5.22)	1483	9.03 (9.02-9.05)
1995-2004	45-50	Anxiety disorder	6853	20.75 (20.73-20.77)	2820	16.85 (16.82-16.87)	4033	24.76 (24.72-24.80)
2005-2016	45-50	Anxiety disorder	12600	31.70 (31.67-31.73)	5792	28.54 (28.50-28.58)	6808	35.01 (34.96-35.06)
1970-2016	50-55	Anxiety disorder	22039	15.59 (15.58-15.60)	8850	12.51 (12.50-12.52)	13189	18.68 (18.67-18.69)
1970-1984	50-55	Anxiety disorder	5039	12.03 (12.02-12.04)	1577	7.63 (7.62-7.64)	3462	16.32 (16.30-16.34)
1985-1994	50-55	Anxiety disorder	1746	6.36 (6.36-6.37)	618	4.51 (4.51-4.52)	1128	8.21 (8.19-8.22)
1995-2004	50-55	Anxiety disorder	5645	16.44 (16.42-16.46)	2217	12.83 (12.81-12.85)	3428	20.10 (20.07-20.13)

			Both sexes		Males		Females	
Years	Age	Mental disorder	Cases	IR (95% CI)	Cases	IR (95% CI)	Cases	IR (95% CI)
2005-2016	50-55	Anxiety disorder	9609	25.47 (25.45-25.50)	4438	23.19 (23.16-23.23)	5171	27.81 (27.77-27.85)
1970-2016	55-60	Anxiety disorder	15613	11.65 (11.64-11.66)	6131	9.24 (9.23-9.24)	9482	14.02 (14.01-14.03)
1970-1984	55-60	Anxiety disorder	3827	9.23 (9.22-9.24)	1132	5.59 (5.58-5.60)	2695	12.71 (12.69-12.73)
1985-1994	55-60	Anxiety disorder	1142	4.59 (4.58-4.60)	363	2.97 (2.96-2.97)	779	6.15 (6.14-6.16)
1995-2004	55-60	Anxiety disorder	3840	12.41 (12.40-12.43)	1450	9.38 (9.37-9.40)	2390	15.44 (15.41-15.46)
2005-2016	55-60	Anxiety disorder	6804	18.52 (18.50-18.54)	3186	17.28 (17.25-17.30)	3618	19.77 (19.74-19.80)
1970-2016	60-65	Anxiety disorder	10145	8.07 (8.07-8.08)	3797	6.21 (6.20-6.21)	6348	9.84 (9.83-9.85)
1970-1984	60-65	Anxiety disorder	2528	6.39 (6.39-6.40)	706	3.72 (3.71-3.73)	1822	8.86 (8.85-8.87)
1985-1994	60-65	Anxiety disorder	958	3.96 (3.95-3.96)	295	2.54 (2.54-2.54)	663	5.27 (5.26-5.28)
1995-2004	60-65	Anxiety disorder	2403	9.68 (9.66-9.69)	885	7.28 (7.27-7.29)	1518	11.97 (11.95-11.99)
2005-2016	60-65	Anxiety disorder	4256	11.48 (11.47-11.49)	1911	10.39 (10.37-10.40)	2345	12.55 (12.53-12.57)
1970-2016	65-70	Anxiety disorder	7581	6.71 (6.71-6.71)	2649	4.96 (4.95-4.96)	4932	8.28 (8.28-8.29)
1970-1984	65-70	Anxiety disorder	1683	4.79 (4.79-4.80)	440	2.70 (2.70-2.71)	1243	6.60 (6.59-6.61)
1985-1994	65-70	Anxiety disorder	821	3.54 (3.53-3.54)	233	2.16 (2.16-2.17)	588	4.73 (4.72-4.74)
1995-2004	65-70	Anxiety disorder	1984	9.30 (9.29-9.31)	674	6.66 (6.64-6.67)	1310	11.70 (11.67-11.72)
2005-2016	65-70	Anxiety disorder	3093	9.28 (9.27-9.29)	1302	8.01 (8.00-8.03)	1791	10.49 (10.47-10.50)
1970-2016	70-75	Anxiety disorder	6077	6.48 (6.48-6.49)	2009	4.75 (4.75-4.76)	4068	7.90 (7.90-7.91)
1970-1984	70-75	Anxiety disorder	1088	3.79 (3.79-3.80)	269	2.14 (2.14-2.15)	819	5.07 (5.07-5.08)
1985-1994	70-75	Anxiety disorder	667	3.29 (3.28-3.29)	183	2.04 (2.04-2.05)	484	4.27 (4.27-4.28)
1995-2004	70-75	Anxiety disorder	1809	9.54 (9.53-9.55)	567	6.62 (6.61-6.63)	1242	11.95 (11.92-11.97)
2005-2016	70-75	Anxiety disorder	2513	9.74 (9.73-9.75)	990	8.12 (8.10-8.13)	1523	11.19 (11.17-11.21)
1970-2016	75-80	Anxiety disorder	4664	6.51 (6.50-6.51)	1482	4.92 (4.92-4.93)	3182	7.65 (7.64-7.66)
1970-1984	75-80	Anxiety disorder	511	2.50 (2.50-2.51)	146	1.76 (1.76-1.76)	365	3.02 (3.01-3.02)
1985-1994	75-80	Anxiety disorder	465	2.87 (2.87-2.88)	118	1.79 (1.79-1.80)	347	3.61 (3.60-3.61)
1995-2004	75-80	Anxiety disorder	1555	9.77 (9.76-9.79)	445	6.71 (6.70-6.73)	1110	11.96 (11.93-11.98)
2005-2016	75-80	Anxiety disorder	2133	11.12 (11.10-11.13)	773	9.00 (8.98-9.02)	1360	12.84 (12.81-12.86)
1970-2016	80-85	Anxiety disorder	3020	6.28 (6.28-6.29)	930	5.10 (5.09-5.11)	2090	7.01 (7.00-7.02)
1970-1984	80-85	Anxiety disorder	154	1.26 (1.26-1.27)	41	0.88 (0.88-0.89)	113	1.50 (1.49-1.50)
1985-1994	80-85	Anxiety disorder	230	2.12 (2.12-2.12)	72	1.87 (1.86-1.87)	158	2.26 (2.26-2.27)
1995-2004	80-85	Anxiety disorder	1071	9.48 (9.46-9.50)	291	6.97 (6.95-6.99)	780	10.95 (10.92-10.97)
2005-2016	80-85	Anxiety disorder	1565	11.39 (11.37-11.41)	526	9.43 (9.41-9.46)	1039	12.72 (12.70-12.75)
1970-2016	85-90	Anxiety disorder	1652	6.40 (6.40-6.41)	476	5.56 (5.55-5.57)	1176	6.82 (6.81-6.83)
1970-1984	85-90	Anxiety disorder	53	0.96 (0.96-0.96)	20	1.00 (1.00-1.01)	33	0.93 (0.93-0.94)
1985-1994	85-90	Anxiety disorder	77	1.41 (1.41-1.42)	25	1.49 (1.48-1.50)	52	1.38 (1.37-1.38)
1995-2004	85-90	Anxiety disorder	548	8.54 (8.52-8.56)	139	6.94 (6.91-6.97)	409	9.26 (9.23-9.29)
2005-2016	85-90	Anxiety disorder	974	11.60 (11.58-11.63)	292	10.11 (10.07-10.14)	682	12.39 (12.36-12.42)
1970-2016	90-95	Anxiety disorder	550	5.81 (5.80-5.83)	159	6.04 (6.02-6.06)	391	5.73 (5.71-5.74)
1970-1984	90-95	Anxiety disorder	11	0.69 (0.68-0.69)	7-11	1.63 (1.62-1.65)	0-4	NA
1985-1994	90-95	Anxiety disorder	21	1.13 (1.13-1.14)	6	1.18 (1.17-1.19)	15	1.11 (1.11-1.12)
1995-2004	90-95	Anxiety disorder	149	6.08 (6.05-6.10)	32	5.24 (5.20-5.29)	117	6.35 (6.32-6.38)

			Both sexes		Males		Females	
Years	Age	Mental disorder	Cases	IR (95% CI)	Cases	IR (95% CI)	Cases	IR (95% CI)
2005-2016	90-95	Anxiety disorder	369	10.40 (10.36-10.43)	112	11.61 (11.54-11.69)	257	9.94 (9.90-9.98)
1970-2016	95-100	Anxiety disorder	126	6.38 (6.35-6.41)	26	5.86 (5.81-5.92)	100	6.53 (6.50-6.56)
1970-1984	95-100	Anxiety disorder	0-4	NA	0-4	NA	0-4	NA
1985-1994	95-100	Anxiety disorder	0-4	NA	0-4	NA	0-4	NA
1995-2004	95-100	Anxiety disorder	31	6.17 (6.11-6.22)	8	8.04 (7.89-8.20)	23	5.70 (5.65-5.76)
2005-2016	95-100	Anxiety disorder	91	10.49 (10.42-10.56)	18	10.21 (10.06-10.36)	73	10.56 (10.48-10.64)
1970-2016	5-10	Anxiety disorder (inpatient)	218	0.14 (0.14-0.14)	148	0.19 (0.19-0.19)	70	0.09 (0.09-0.09)
1970-1984	5-10	Anxiety disorder (inpatient)	39	0.07 (0.07-0.07)	30	0.11 (0.11-0.11)	9	0.03 (0.03-0.03)
1985-1994	5-10	Anxiety disorder (inpatient)	27	0.10 (0.10-0.10)	16	0.11 (0.11-0.11)	11	0.08 (0.08-0.08)
1995-2004	5-10	Anxiety disorder (inpatient)	57	0.17 (0.17-0.17)	40	0.24 (0.24-0.24)	17	0.11 (0.11-0.11)
2005-2016	5-10	Anxiety disorder (inpatient)	95	0.24 (0.24-0.24)	62	0.31 (0.31-0.31)	33	0.17 (0.17-0.17)
1970-2016	10-15	Anxiety disorder (inpatient)	2420	1.53 (1.53-1.53)	828	1.02 (1.02-1.02)	1592	2.06 (2.06-2.06)
1970-1984	10-15	Anxiety disorder (inpatient)	315	0.55 (0.55-0.55)	105	0.36 (0.36-0.36)	210	0.76 (0.75-0.76)
1985-1994	10-15	Anxiety disorder (inpatient)	151	0.48 (0.48-0.48)	58	0.36 (0.36-0.36)	93	0.60 (0.60-0.60)
1995-2004	10-15	Anxiety disorder (inpatient)	440	1.49 (1.49-1.49)	152	1.00 (1.00-1.00)	288	2.00 (2.00-2.00)
2005-2016	10-15	Anxiety disorder (inpatient)	1514	3.77 (3.77-3.78)	513	2.50 (2.49-2.50)	1001	5.12 (5.11-5.12)
1970-2016	15-20	Anxiety disorder (inpatient)	11829	7.37 (7.37-7.38)	3892	4.72 (4.72-4.73)	7937	10.17 (10.16-10.18)
1970-1984	15-20	Anxiety disorder (inpatient)	2926	5.13 (5.13-5.13)	1005	3.43 (3.43-3.43)	1921	6.93 (6.92-6.94)
1985-1994	15-20	Anxiety disorder (inpatient)	1092	3.09 (3.09-3.10)	397	2.19 (2.19-2.20)	695	4.04 (4.04-4.05)
1995-2004	15-20	Anxiety disorder (inpatient)	2032	7.15 (7.14-7.16)	623	4.28 (4.27-4.29)	1409	10.17 (10.15-10.18)
2005-2016	15-20	Anxiety disorder (inpatient)	5779	14.55 (14.54-14.57)	1867	9.14 (9.13-9.15)	3912	20.29 (20.26-20.32)
1970-2016	20-25	Anxiety disorder (inpatient)	16334	10.17 (10.17-10.18)	6882	8.28 (8.27-8.29)	9452	12.20 (12.19-12.21)
1970-1984	20-25	Anxiety disorder (inpatient)	5373	9.72 (9.71-9.73)	2241	7.84 (7.83-7.85)	3132	11.73 (11.71-11.74)
1985-1994	20-25	Anxiety disorder (inpatient)	2083	5.60 (5.59-5.60)	951	4.91 (4.90-4.91)	1132	6.35 (6.34-6.36)
1995-2004	20-25	Anxiety disorder (inpatient)	2812	9.10 (9.09-9.11)	1142	7.14 (7.13-7.15)	1670	11.21 (11.19-11.22)
2005-2016	20-25	Anxiety disorder (inpatient)	6066	16.31 (16.29-16.33)	2548	13.29 (13.27-13.31)	3518	19.52 (19.49-19.55)
1970-2016	25-30	Anxiety disorder (inpatient)	16892	10.51 (10.50-10.51)	7362	8.87 (8.87-8.88)	9530	12.25 (12.24-12.26)
1970-1984	25-30	Anxiety disorder (inpatient)	6722	11.93 (11.92-11.94)	2736	9.43 (9.42-9.44)	3986	14.59 (14.57-14.60)
1985-1994	25-30	Anxiety disorder (inpatient)	2453	6.69 (6.68-6.70)	1063	5.59 (5.58-5.59)	1390	7.89 (7.87-7.90)
1995-2004	25-30	Anxiety disorder (inpatient)	3143	9.38 (9.37-9.39)	1379	7.95 (7.93-7.96)	1764	10.93 (10.91-10.94)
2005-2016	25-30	Anxiety disorder (inpatient)	4574	13.33 (13.32-13.35)	2184	12.41 (12.39-12.43)	2390	14.30 (14.28-14.32)
1970-2016	30-35	Anxiety disorder (inpatient)	17682	11.12 (11.11-11.12)	7728	9.46 (9.46-9.47)	9954	12.86 (12.85-12.87)
1970-1984	30-35	Anxiety disorder (inpatient)	7184	13.34 (13.33-13.35)	2760	10.03 (10.02-10.04)	4424	16.79 (16.77-16.82)
1985-1994	30-35	Anxiety disorder (inpatient)	2806	8.19 (8.18-8.20)	1231	6.96 (6.95-6.98)	1575	9.50 (9.48-9.51)
1995-2004	30-35	Anxiety disorder (inpatient)	3399	9.51 (9.50-9.52)	1589	8.61 (8.59-8.62)	1810	10.48 (10.46-10.49)
2005-2016	30-35	Anxiety disorder (inpatient)	4293	12.20 (12.18-12.21)	2148	11.92 (11.91-11.94)	2145	12.48 (12.46-12.50)
1970-2016	35-40	Anxiety disorder (inpatient)	17358	11.05 (11.05-11.06)	7438	9.28 (9.27-9.29)	9920	12.90 (12.89-12.91)
1970-1984	35-40	Anxiety disorder (inpatient)	6898	14.09 (14.07-14.10)	2570	10.34 (10.33-10.36)	4328	17.94 (17.92-17.96)
1985-1994	35-40	Anxiety disorder (inpatient)	2686	7.80 (7.79-7.80)	1085	6.17 (6.16-6.18)	1601	9.49 (9.48-9.51)
1995-2004	35-40	Anxiety disorder (inpatient)	3427	9.65 (9.64-9.66)	1591	8.72 (8.71-8.74)	1836	10.62 (10.60-10.63)

			Both sexes		Males		Females	
Years	Age	Mental disorder	Cases	IR (95% CI)	Cases	IR (95% CI)	Cases	IR (95% CI)
2005-2016	35-40	Anxiety disorder (inpatient)	4347	11.41 (11.40-11.42)	2192	11.25 (11.23-11.27)	2155	11.57 (11.56-11.59)
1970-2016	40-45	Anxiety disorder (inpatient)	16398	10.69 (10.68-10.70)	6767	8.70 (8.69-8.70)	9631	12.74 (12.73-12.75)
1970-1984	40-45	Anxiety disorder (inpatient)	6230	14.51 (14.50-14.52)	2127	9.87 (9.85-9.88)	4103	19.19 (19.17-19.22)
1985-1994	40-45	Anxiety disorder (inpatient)	2757	7.62 (7.61-7.62)	1042	5.68 (5.67-5.68)	1715	9.61 (9.60-9.62)
1995-2004	40-45	Anxiety disorder (inpatient)	3076	9.24 (9.23-9.25)	1363	8.02 (8.01-8.04)	1713	10.51 (10.49-10.52)
2005-2016	40-45	Anxiety disorder (inpatient)	4335	10.58 (10.57-10.59)	2235	10.70 (10.68-10.71)	2100	10.46 (10.45-10.48)
1970-2016	45-50	Anxiety disorder (inpatient)	14568	9.78 (9.77-9.78)	6034	8.04 (8.04-8.05)	8534	11.54 (11.53-11.55)
1970-1984	45-50	Anxiety disorder (inpatient)	5768	13.87 (13.86-13.89)	2009	9.72 (9.71-9.73)	3759	17.98 (17.95-18.00)
1985-1994	45-50	Anxiety disorder (inpatient)	2261	6.82 (6.81-6.83)	847	5.06 (5.05-5.07)	1414	8.61 (8.60-8.63)
1995-2004	45-50	Anxiety disorder (inpatient)	2502	7.52 (7.51-7.53)	1081	6.42 (6.41-6.43)	1421	8.65 (8.63-8.66)
2005-2016	45-50	Anxiety disorder (inpatient)	4037	9.85 (9.84-9.86)	2097	10.08 (10.07-10.10)	1940	9.60 (9.59-9.62)
1970-2016	50-55	Anxiety disorder (inpatient)	11768	8.25 (8.25-8.26)	4525	6.35 (6.35-6.36)	7243	10.15 (10.14-10.16)
1970-1984	50-55	Anxiety disorder (inpatient)	5039	12.03 (12.02-12.04)	1577	7.63 (7.62-7.64)	3462	16.32 (16.30-16.34)
1985-1994	50-55	Anxiety disorder (inpatient)	1664	6.07 (6.06-6.07)	586	4.28 (4.27-4.29)	1078	7.84 (7.83-7.86)
1995-2004	50-55	Anxiety disorder (inpatient)	1985	5.75 (5.74-5.75)	800	4.61 (4.60-4.62)	1185	6.90 (6.89-6.91)
2005-2016	50-55	Anxiety disorder (inpatient)	3080	7.95 (7.94-7.96)	1562	7.99 (7.98-8.00)	1518	7.90 (7.89-7.91)
1970-2016	55-60	Anxiety disorder (inpatient)	8452	6.26 (6.26-6.27)	3089	4.63 (4.63-4.63)	5363	7.86 (7.86-7.87)
1970-1984	55-60	Anxiety disorder (inpatient)	3827	9.23 (9.22-9.24)	1132	5.59 (5.58-5.60)	2695	12.71 (12.69-12.73)
1985-1994	55-60	Anxiety disorder (inpatient)	1092	4.39 (4.38-4.39)	349	2.85 (2.85-2.86)	743	5.87 (5.86-5.88)
1995-2004	55-60	Anxiety disorder (inpatient)	1330	4.28 (4.27-4.28)	497	3.20 (3.20-3.21)	833	5.35 (5.34-5.35)
2005-2016	55-60	Anxiety disorder (inpatient)	2203	5.87 (5.87-5.88)	1111	5.93 (5.92-5.93)	1092	5.82 (5.81-5.83)
1970-2016	60-65	Anxiety disorder (inpatient)	5859	4.64 (4.64-4.64)	2067	3.37 (3.36-3.37)	3792	5.84 (5.84-5.85)
1970-1984	60-65	Anxiety disorder (inpatient)	2528	6.39 (6.39-6.40)	706	3.72 (3.71-3.73)	1822	8.86 (8.85-8.87)
1985-1994	60-65	Anxiety disorder (inpatient)	927	3.83 (3.83-3.84)	286	2.46 (2.46-2.47)	641	5.10 (5.09-5.11)
1995-2004	60-65	Anxiety disorder (inpatient)	866	3.47 (3.47-3.48)	342	2.81 (2.80-2.81)	524	4.11 (4.11-4.12)
2005-2016	60-65	Anxiety disorder (inpatient)	1538	4.08 (4.08-4.09)	733	3.94 (3.93-3.94)	805	4.23 (4.22-4.23)
1970-2016	65-70	Anxiety disorder (inpatient)	4304	3.79 (3.79-3.80)	1380	2.58 (2.57-2.58)	2924	4.89 (4.88-4.89)
1970-1984	65-70	Anxiety disorder (inpatient)	1682	4.79 (4.79-4.80)	440	2.70 (2.70-2.71)	1242	6.59 (6.58-6.60)
1985-1994	65-70	Anxiety disorder (inpatient)	793	3.42 (3.41-3.42)	229	2.13 (2.12-2.13)	564	4.54 (4.53-4.55)
1995-2004	65-70	Anxiety disorder (inpatient)	690	3.23 (3.22-3.23)	228	2.25 (2.24-2.25)	462	4.11 (4.10-4.12)
2005-2016	65-70	Anxiety disorder (inpatient)	1139	3.38 (3.37-3.38)	483	2.95 (2.94-2.95)	656	3.79 (3.78-3.79)
1970-2016	70-75	Anxiety disorder (inpatient)	3221	3.43 (3.42-3.43)	1014	2.39 (2.39-2.40)	2207	4.27 (4.27-4.28)
1970-1984	70-75	Anxiety disorder (inpatient)	1088	3.79 (3.79-3.80)	269	2.14 (2.14-2.15)	819	5.07 (5.07-5.08)
1985-1994	70-75	Anxiety disorder (inpatient)	636	3.14 (3.13-3.14)	171	1.91 (1.91-1.91)	465	4.11 (4.10-4.11)
1995-2004	70-75	Anxiety disorder (inpatient)	601	3.16 (3.16-3.17)	199	2.32 (2.31-2.32)	402	3.85 (3.85-3.86)
2005-2016	70-75	Anxiety disorder (inpatient)	896	3.44 (3.44-3.44)	375	3.05 (3.05-3.06)	521	3.78 (3.78-3.79)
1970-2016	75-80	Anxiety disorder (inpatient)	2129	2.96 (2.96-2.96)	677	2.25 (2.24-2.25)	1452	3.48 (3.47-3.48)
1970-1984	75-80	Anxiety disorder (inpatient)	511	2.50 (2.50-2.51)	146	1.76 (1.76-1.76)	365	3.02 (3.01-3.02)
1985-1994	75-80	Anxiety disorder (inpatient)	440	2.72 (2.71-2.72)	113	1.72 (1.71-1.72)	327	3.40 (3.39-3.41)
1995-2004	75-80	Anxiety disorder (inpatient)	462	2.90 (2.89-2.90)	137	2.06 (2.06-2.07)	325	3.49 (3.48-3.49)

			Both sexes		Males		Females	
**Years**	**Age**	**Mental disorder**	**Cases**	**IR (95% CI)**	**Cases**	**IR (95% CI)**	**Cases**	**IR (95% CI)**
2005-2016	75-80	Anxiety disorder (inpatient)	716	3.70 (3.70-3.71)	281	3.25 (3.25-3.26)	435	4.06 (4.06-4.07)
1970-2016	80-85	Anxiety disorder (inpatient)	1163	2.41 (2.41-2.41)	393	2.15 (2.15-2.15)	770	2.57 (2.57-2.58)
1970-1984	80-85	Anxiety disorder (inpatient)	154	1.26 (1.26-1.27)	41	0.88 (0.88-0.89)	113	1.50 (1.49-1.50)
1985-1994	80-85	Anxiety disorder (inpatient)	220	2.03 (2.02-2.03)	69	1.79 (1.78-1.80)	151	2.16 (2.16-2.17)
1995-2004	80-85	Anxiety disorder (inpatient)	299	2.64 (2.63-2.64)	92	2.20 (2.19-2.21)	207	2.89 (2.89-2.90)
2005-2016	80-85	Anxiety disorder (inpatient)	490	3.54 (3.53-3.54)	191	3.41 (3.40-3.42)	299	3.63 (3.62-3.63)
1970-2016	85-90	Anxiety disorder (inpatient)	521	2.01 (2.01-2.02)	175	2.04 (2.04-2.04)	346	2.00 (2.00-2.00)
1970-1984	85-90	Anxiety disorder (inpatient)	53	0.96 (0.96-0.96)	20	1.00 (1.00-1.01)	33	0.93 (0.93-0.94)
1985-1994	85-90	Anxiety disorder (inpatient)	70	1.28 (1.28-1.29)	25	1.49 (1.48-1.50)	45	1.19 (1.19-1.20)
1995-2004	85-90	Anxiety disorder (inpatient)	147	2.28 (2.28-2.29)	45	2.24 (2.23-2.25)	102	2.30 (2.29-2.31)
2005-2016	85-90	Anxiety disorder (inpatient)	251	2.97 (2.96-2.97)	85	2.93 (2.92-2.94)	166	2.99 (2.98-2.99)
1970-2016	90-95	Anxiety disorder (inpatient)	147	1.55 (1.55-1.55)	58	2.20 (2.19-2.21)	89	1.30 (1.30-1.30)
1970-1984	90-95	Anxiety disorder (inpatient)	11	0.69 (0.68-0.69)	7-11	1.63 (1.62-1.65)	0-4	NA
1985-1994	90-95	Anxiety disorder (inpatient)	19	1.02 (1.02-1.03)	6	1.18 (1.17-1.19)	13	0.96 (0.96-0.97)
1995-2004	90-95	Anxiety disorder (inpatient)	32	1.30 (1.30-1.31)	10	1.64 (1.62-1.65)	22	1.19 (1.19-1.20)
2005-2016	90-95	Anxiety disorder (inpatient)	85	2.38 (2.37-2.38)	33	3.41 (3.38-3.43)	52	1.99 (1.99-2.00)
1970-2016	95-100	Anxiety disorder (inpatient)	30	1.51 (1.51-1.52)	7	1.57 (1.56-1.59)	23	1.50 (1.49-1.50)
1970-1984	95-100	Anxiety disorder (inpatient)	0-4	NA	0-4	NA	0-4	NA
1985-1994	95-100	Anxiety disorder (inpatient)	0-4	NA	0-4	NA	0-4	NA
1995-2004	95-100	Anxiety disorder (inpatient)	5	0.99 (0.98-1.00)	0-4	NA	0-4	NA
2005-2016	95-100	Anxiety disorder (inpatient)	21	2.40 (2.39-2.42)	5	2.82 (2.78-2.86)	16	2.30 (2.28-2.31)
1970-2016	5-10	Obsessive-compulsive disorder	1168	0.75 (0.75-0.75)	634	0.79 (0.79-0.79)	534	0.70 (0.70-0.70)
1970-1984	5-10	Obsessive-compulsive disorder	0-4	NA	0-4	NA	0-4	NA
1985-1994	5-10	Obsessive-compulsive disorder	8	0.03 (0.03-0.03)	4-8	0.04 (0.04-0.04)	0-4	NA
1995-2004	5-10	Obsessive-compulsive disorder	242	0.73 (0.73-0.74)	131	0.78 (0.77-0.78)	111	0.69 (0.69-0.69)
2005-2016	5-10	Obsessive-compulsive disorder	918	2.34 (2.33-2.34)	497	2.47 (2.47-2.47)	421	2.20 (2.19-2.20)
1970-2016	10-15	Obsessive-compulsive disorder	3710	2.35 (2.35-2.35)	1759	2.17 (2.17-2.17)	1951	2.53 (2.53-2.53)
1970-1984	10-15	Obsessive-compulsive disorder	13	0.02 (0.02-0.02)	7	0.02 (0.02-0.02)	6	0.02 (0.02-0.02)
1985-1994	10-15	Obsessive-compulsive disorder	37	0.12 (0.12-0.12)	19	0.12 (0.12-0.12)	18	0.12 (0.12-0.12)
1995-2004	10-15	Obsessive-compulsive disorder	809	2.74 (2.73-2.74)	433	2.86 (2.85-2.86)	376	2.61 (2.61-2.61)
2005-2016	10-15	Obsessive-compulsive disorder	2851	7.12 (7.11-7.13)	1300	6.34 (6.33-6.34)	1551	7.94 (7.93-7.95)
1970-2016	15-20	Obsessive-compulsive disorder	3491	2.17 (2.17-2.18)	1200	1.46 (1.46-1.46)	2291	2.93 (2.93-2.93)
1970-1984	15-20	Obsessive-compulsive disorder	49	0.09 (0.09-0.09)	21	0.07 (0.07-0.07)	28	0.10 (0.10-0.10)
1985-1994	15-20	Obsessive-compulsive disorder	35	0.10 (0.10-0.10)	15	0.08 (0.08-0.08)	20	0.12 (0.12-0.12)
1995-2004	15-20	Obsessive-compulsive disorder	617	2.17 (2.17-2.17)	241	1.66 (1.65-1.66)	376	2.71 (2.70-2.71)
2005-2016	15-20	Obsessive-compulsive disorder	2790	7.03 (7.02-7.03)	923	4.52 (4.52-4.53)	1867	9.67 (9.66-9.69)
1970-2016	20-25	Obsessive-compulsive disorder	3066	1.90 (1.90-1.90)	1027	1.23 (1.23-1.23)	2039	2.62 (2.62-2.62)
1970-1984	20-25	Obsessive-compulsive disorder	117	0.21 (0.21-0.21)	45	0.16 (0.16-0.16)	72	0.27 (0.27-0.27)
1985-1994	20-25	Obsessive-compulsive disorder	39	0.10 (0.10-0.10)	17	0.09 (0.09-0.09)	22	0.12 (0.12-0.12)
1995-2004	20-25	Obsessive-compulsive disorder	516	1.66 (1.66-1.67)	212	1.32 (1.32-1.32)	304	2.03 (2.03-2.03)

			Both sexes		Males		Females	
**Years**	**Age**	**Mental disorder**	**Cases**	**IR (95% CI)**	**Cases**	**IR (95% CI)**	**Cases**	**IR (95% CI)**
2005-2016	20-25	Obsessive-compulsive disorder	2394	6.40 (6.40-6.41)	753	3.92 (3.91-3.92)	1641	9.04 (9.03-9.05)
1970-2016	25-30	Obsessive-compulsive disorder	2282	1.41 (1.41-1.41)	806	0.97 (0.97-0.97)	1476	1.88 (1.88-1.88)
1970-1984	25-30	Obsessive-compulsive disorder	119	0.21 (0.21-0.21)	44	0.15 (0.15-0.15)	75	0.27 (0.27-0.27)
1985-1994	25-30	Obsessive-compulsive disorder	54	0.15 (0.15-0.15)	22	0.11 (0.11-0.12)	32	0.18 (0.18-0.18)
1995-2004	25-30	Obsessive-compulsive disorder	449	1.33 (1.33-1.33)	190	1.09 (1.09-1.09)	259	1.59 (1.59-1.59)
2005-2016	25-30	Obsessive-compulsive disorder	1660	4.80 (4.79-4.80)	550	3.10 (3.10-3.11)	1110	6.57 (6.56-6.58)
1970-2016	30-35	Obsessive-compulsive disorder	1691	1.05 (1.05-1.05)	585	0.71 (0.71-0.71)	1106	1.41 (1.41-1.41)
1970-1984	30-35	Obsessive-compulsive disorder	125	0.23 (0.23-0.23)	48	0.17 (0.17-0.17)	77	0.29 (0.29-0.29)
1985-1994	30-35	Obsessive-compulsive disorder	30	0.09 (0.09-0.09)	15	0.08 (0.08-0.08)	15	0.09 (0.09-0.09)
1995-2004	30-35	Obsessive-compulsive disorder	342	0.95 (0.95-0.95)	154	0.83 (0.83-0.83)	188	1.08 (1.07-1.08)
2005-2016	30-35	Obsessive-compulsive disorder	1194	3.35 (3.35-3.36)	368	2.02 (2.02-2.03)	826	4.74 (4.74-4.75)
1970-2016	35-40	Obsessive-compulsive disorder	1297	0.81 (0.81-0.81)	502	0.62 (0.62-0.62)	795	1.02 (1.02-1.02)
1970-1984	35-40	Obsessive-compulsive disorder	104	0.21 (0.21-0.21)	42	0.17 (0.17-0.17)	62	0.25 (0.25-0.25)
1985-1994	35-40	Obsessive-compulsive disorder	44	0.13 (0.13-0.13)	23	0.13 (0.13-0.13)	21	0.12 (0.12-0.12)
1995-2004	35-40	Obsessive-compulsive disorder	294	0.82 (0.82-0.82)	113	0.61 (0.61-0.61)	181	1.03 (1.03-1.03)
2005-2016	35-40	Obsessive-compulsive disorder	855	2.21 (2.21-2.21)	324	1.64 (1.64-1.64)	531	2.81 (2.80-2.81)
1970-2016	40-45	Obsessive-compulsive disorder	1005	0.64 (0.64-0.64)	416	0.53 (0.53-0.53)	589	0.76 (0.76-0.76)
1970-1984	40-45	Obsessive-compulsive disorder	111	0.26 (0.26-0.26)	45	0.21 (0.21-0.21)	66	0.30 (0.30-0.30)
1985-1994	40-45	Obsessive-compulsive disorder	32	0.09 (0.09-0.09)	18	0.10 (0.10-0.10)	14	0.08 (0.08-0.08)
1995-2004	40-45	Obsessive-compulsive disorder	195	0.57 (0.57-0.58)	88	0.51 (0.51-0.51)	107	0.64 (0.64-0.64)
2005-2016	40-45	Obsessive-compulsive disorder	667	1.60 (1.60-1.60)	265	1.25 (1.25-1.25)	402	1.96 (1.96-1.97)
1970-2016	45-50	Obsessive-compulsive disorder	682	0.45 (0.45-0.45)	266	0.35 (0.35-0.35)	416	0.55 (0.55-0.55)
1970-1984	45-50	Obsessive-compulsive disorder	77	0.18 (0.18-0.18)	29	0.14 (0.14-0.14)	48	0.23 (0.23-0.23)
1985-1994	45-50	Obsessive-compulsive disorder	22	0.06 (0.06-0.07)	7	0.04 (0.04-0.04)	15	0.09 (0.09-0.09)
1995-2004	45-50	Obsessive-compulsive disorder	152	0.45 (0.45-0.45)	54	0.32 (0.32-0.32)	98	0.58 (0.58-0.58)
2005-2016	45-50	Obsessive-compulsive disorder	431	1.03 (1.03-1.03)	176	0.83 (0.83-0.83)	255	1.23 (1.23-1.23)
1970-2016	50-55	Obsessive-compulsive disorder	502	0.35 (0.34-0.35)	205	0.28 (0.28-0.28)	297	0.41 (0.41-0.41)
1970-1984	50-55	Obsessive-compulsive disorder	73	0.17 (0.17-0.17)	28	0.13 (0.13-0.13)	45	0.21 (0.21-0.21)
1985-1994	50-55	Obsessive-compulsive disorder	25	0.09 (0.09-0.09)	8	0.06 (0.06-0.06)	17	0.12 (0.12-0.12)
1995-2004	50-55	Obsessive-compulsive disorder	120	0.34 (0.34-0.34)	55	0.31 (0.31-0.31)	65	0.37 (0.37-0.37)
2005-2016	50-55	Obsessive-compulsive disorder	284	0.72 (0.71-0.72)	114	0.57 (0.57-0.57)	170	0.86 (0.86-0.86)
1970-2016	55-60	Obsessive-compulsive disorder	344	0.25 (0.25-0.25)	132	0.20 (0.19-0.20)	212	0.30 (0.30-0.30)
1970-1984	55-60	Obsessive-compulsive disorder	65	0.16 (0.16-0.16)	24	0.12 (0.12-0.12)	41	0.19 (0.19-0.19)
1985-1994	55-60	Obsessive-compulsive disorder	12	0.05 (0.05-0.05)	6	0.05 (0.05-0.05)	6	0.05 (0.05-0.05)
1995-2004	55-60	Obsessive-compulsive disorder	85	0.27 (0.27-0.27)	31	0.20 (0.20-0.20)	54	0.34 (0.34-0.34)
2005-2016	55-60	Obsessive-compulsive disorder	182	0.47 (0.47-0.47)	71	0.37 (0.37-0.37)	111	0.57 (0.57-0.57)
1970-2016	60-65	Obsessive-compulsive disorder	228	0.18 (0.18-0.18)	86	0.14 (0.14-0.14)	142	0.21 (0.21-0.21)
1970-1984	60-65	Obsessive-compulsive disorder	39	0.10 (0.10-0.10)	15	0.08 (0.08-0.08)	24	0.12 (0.12-0.12)
1985-1994	60-65	Obsessive-compulsive disorder	16	0.06 (0.06-0.07)	5	0.04 (0.04-0.04)	11	0.09 (0.09-0.09)
1995-2004	60-65	Obsessive-compulsive disorder	63	0.25 (0.25-0.25)	22	0.18 (0.18-0.18)	41	0.31 (0.31-0.31)

			Both sexes		Males		Females	
Years	Age	Mental disorder	Cases	IR (95% CI)	Cases	IR (95% CI)	Cases	IR (95% CI)
2005-2016	60-65	Obsessive-compulsive disorder	110	0.28 (0.28-0.29)	44	0.23 (0.23-0.23)	66	0.34 (0.34-0.34)
1970-2016	65-70	Obsessive-compulsive disorder	144	0.12 (0.12-0.13)	49	0.09 (0.09-0.09)	95	0.16 (0.16-0.16)
1970-1984	65-70	Obsessive-compulsive disorder	30	0.08 (0.08-0.09)	8	0.05 (0.05-0.05)	22	0.12 (0.12-0.12)
1985-1994	65-70	Obsessive-compulsive disorder	8	0.03 (0.03-0.03)	0-4	NA	4-8	0.04 (0.04-0.04)
1995-2004	65-70	Obsessive-compulsive disorder	46	0.21 (0.21-0.21)	15	0.15 (0.15-0.15)	31	0.27 (0.27-0.27)
2005-2016	65-70	Obsessive-compulsive disorder	60	0.17 (0.17-0.17)	23	0.14 (0.14-0.14)	37	0.21 (0.21-0.21)
1970-2016	70-75	Obsessive-compulsive disorder	107	0.11 (0.11-0.11)	28	0.07 (0.07-0.07)	79	0.15 (0.15-0.15)
1970-1984	70-75	Obsessive-compulsive disorder	19	0.07 (0.07-0.07)	0-4	NA	15-19	0.10 (0.10-0.10)
1985-1994	70-75	Obsessive-compulsive disorder	0-4	NA	0-4	NA	0-4	NA
1995-2004	70-75	Obsessive-compulsive disorder	33	0.17 (0.17-0.17)	7	0.08 (0.08-0.08)	26	0.24 (0.24-0.24)
2005-2016	70-75	Obsessive-compulsive disorder	51	0.19 (0.19-0.19)	15	0.12 (0.12-0.12)	36	0.25 (0.25-0.25)
1970-2016	75-80	Obsessive-compulsive disorder	68	0.09 (0.09-0.09)	18	0.06 (0.06-0.06)	50	0.12 (0.12-0.12)
1970-1984	75-80	Obsessive-compulsive disorder	6	0.03 (0.03-0.03)	0-4	NA	2-6	0.05 (0.05-0.05)
1985-1994	75-80	Obsessive-compulsive disorder	0-4	NA	0-4	NA	0-4	NA
1995-2004	75-80	Obsessive-compulsive disorder	24	0.15 (0.15-0.15)	8	0.12 (0.12-0.12)	16	0.17 (0.17-0.17)
2005-2016	75-80	Obsessive-compulsive disorder	36	0.18 (0.18-0.18)	10	0.11 (0.11-0.11)	26	0.24 (0.24-0.24)
1970-2016	80-85	Obsessive-compulsive disorder	37	0.08 (0.08-0.08)	10	0.05 (0.05-0.05)	27	0.09 (0.09-0.09)
1970-1984	80-85	Obsessive-compulsive disorder	0-4	NA	0-4	NA	0-4	NA
1985-1994	80-85	Obsessive-compulsive disorder	0-4	NA	0-4	NA	0-4	NA
1995-2004	80-85	Obsessive-compulsive disorder	16	0.14 (0.14-0.14)	6	0.14 (0.14-0.14)	10	0.14 (0.14-0.14)
2005-2016	80-85	Obsessive-compulsive disorder	18	0.13 (0.13-0.13)	0-4	NA	14-18	0.18 (0.18-0.18)
1970-2016	85-90	Obsessive-compulsive disorder	25	0.10 (0.10-0.10)	9	0.10 (0.10-0.10)	16	0.09 (0.09-0.09)
1970-1984	85-90	Obsessive-compulsive disorder	0-4	NA	0-4	NA	0-4	NA
1985-1994	85-90	Obsessive-compulsive disorder	0-4	NA	0-4	NA	0-4	NA
1995-2004	85-90	Obsessive-compulsive disorder	7	0.11 (0.11-0.11)	0-4	NA	3-7	0.11 (0.11-0.11)
2005-2016	85-90	Obsessive-compulsive disorder	17	0.20 (0.20-0.20)	6	0.21 (0.20-0.21)	11	0.19 (0.19-0.20)
1970-2016	90-95	Obsessive-compulsive disorder	5	0.05 (0.05-0.05)	0-4	NA	0-4	NA
1970-1984	90-95	Obsessive-compulsive disorder	0-4	NA	0-4	NA	0-4	NA
1985-1994	90-95	Obsessive-compulsive disorder	0-4	NA	0-4	NA	0-4	NA
1995-2004	90-95	Obsessive-compulsive disorder	0-4	NA	0-4	NA	0-4	NA
2005-2016	90-95	Obsessive-compulsive disorder	0-4	NA	0-4	NA	0-4	NA
1970-2016	95-100	Obsessive-compulsive disorder	5	0.25 (0.25-0.25)	0-4	NA	1-5	0.32 (0.32-0.32)
1970-1984	95-100	Obsessive-compulsive disorder	0-4	NA	0-4	NA	0-4	NA
1985-1994	95-100	Obsessive-compulsive disorder	0-4	NA	0-4	NA	0-4	NA
1995-2004	95-100	Obsessive-compulsive disorder	0-4	NA	0-4	NA	0-4	NA
2005-2016	95-100	Obsessive-compulsive disorder	0-4	NA	0-4	NA	0-4	NA
1970-2016	5-10	Obsessive-compulsive disorder (inpatient)	42	0.03 (0.03-0.03)	25	0.03 (0.03-0.03)	17	0.02 (0.02-0.02)
1970-1984	5-10	Obsessive-compulsive disorder (inpatient)	0-4	NA	0-4	NA	0-4	NA
1985-1994	5-10	Obsessive-compulsive disorder (inpatient)	0-4	NA	0-4	NA	0-4	NA
1995-2004	5-10	Obsessive-compulsive disorder (inpatient)	9	0.03 (0.03-0.03)	5-9	0.04 (0.04-0.04)	0-4	NA

			Both sexes		Males		Females	
**Years**	**Age**	**Mental disorder**	**Cases**	**IR (95% CI)**	**Cases**	**IR (95% CI)**	**Cases**	**IR (95% CI)**
2005-2016	5-10	Obsessive-compulsive disorder (inpatient)	29	0.07 (0.07-0.07)	15	0.07 (0.07-0.07)	14	0.07 (0.07-0.07)
1970-2016	10-15	Obsessive-compulsive disorder (inpatient)	467	0.30 (0.29-0.30)	230	0.28 (0.28-0.28)	237	0.31 (0.31-0.31)
1970-1984	10-15	Obsessive-compulsive disorder (inpatient)	13	0.02 (0.02-0.02)	7	0.02 (0.02-0.02)	6	0.02 (0.02-0.02)
1985-1994	10-15	Obsessive-compulsive disorder (inpatient)	21	0.07 (0.07-0.07)	10	0.06 (0.06-0.06)	11	0.07 (0.07-0.07)
1995-2004	10-15	Obsessive-compulsive disorder (inpatient)	110	0.37 (0.37-0.37)	63	0.42 (0.41-0.42)	47	0.33 (0.33-0.33)
2005-2016	10-15	Obsessive-compulsive disorder (inpatient)	323	0.80 (0.80-0.81)	150	0.73 (0.73-0.73)	173	0.88 (0.88-0.89)
1970-2016	15-20	Obsessive-compulsive disorder (inpatient)	586	0.36 (0.36-0.36)	208	0.25 (0.25-0.25)	378	0.48 (0.48-0.48)
1970-1984	15-20	Obsessive-compulsive disorder (inpatient)	49	0.09 (0.09-0.09)	21	0.07 (0.07-0.07)	28	0.10 (0.10-0.10)
1985-1994	15-20	Obsessive-compulsive disorder (inpatient)	22	0.06 (0.06-0.06)	9	0.05 (0.05-0.05)	13	0.08 (0.08-0.08)
1995-2004	15-20	Obsessive-compulsive disorder (inpatient)	103	0.36 (0.36-0.36)	41	0.28 (0.28-0.28)	62	0.45 (0.45-0.45)
2005-2016	15-20	Obsessive-compulsive disorder (inpatient)	412	1.03 (1.03-1.03)	137	0.67 (0.67-0.67)	275	1.42 (1.42-1.42)
1970-2016	20-25	Obsessive-compulsive disorder (inpatient)	676	0.42 (0.42-0.42)	260	0.31 (0.31-0.31)	416	0.53 (0.53-0.53)
1970-1984	20-25	Obsessive-compulsive disorder (inpatient)	117	0.21 (0.21-0.21)	45	0.16 (0.16-0.16)	72	0.27 (0.27-0.27)
1985-1994	20-25	Obsessive-compulsive disorder (inpatient)	31	0.08 (0.08-0.08)	15	0.08 (0.08-0.08)	16	0.09 (0.09-0.09)
1995-2004	20-25	Obsessive-compulsive disorder (inpatient)	116	0.37 (0.37-0.37)	50	0.31 (0.31-0.31)	66	0.44 (0.44-0.44)
2005-2016	20-25	Obsessive-compulsive disorder (inpatient)	412	1.10 (1.10-1.10)	150	0.78 (0.78-0.78)	262	1.44 (1.43-1.44)
1970-2016	25-30	Obsessive-compulsive disorder (inpatient)	533	0.33 (0.33-0.33)	213	0.26 (0.25-0.26)	320	0.41 (0.41-0.41)
1970-1984	25-30	Obsessive-compulsive disorder (inpatient)	119	0.21 (0.21-0.21)	44	0.15 (0.15-0.15)	75	0.27 (0.27-0.27)
1985-1994	25-30	Obsessive-compulsive disorder (inpatient)	45	0.12 (0.12-0.12)	19	0.10 (0.10-0.10)	26	0.15 (0.15-0.15)
1995-2004	25-30	Obsessive-compulsive disorder (inpatient)	118	0.35 (0.35-0.35)	51	0.29 (0.29-0.29)	67	0.41 (0.41-0.41)
2005-2016	25-30	Obsessive-compulsive disorder (inpatient)	251	0.72 (0.72-0.72)	99	0.56 (0.56-0.56)	152	0.90 (0.89-0.90)
1970-2016	30-35	Obsessive-compulsive disorder (inpatient)	491	0.31 (0.31-0.31)	201	0.24 (0.24-0.24)	290	0.37 (0.37-0.37)
1970-1984	30-35	Obsessive-compulsive disorder (inpatient)	125	0.23 (0.23-0.23)	48	0.17 (0.17-0.17)	77	0.29 (0.29-0.29)
1985-1994	30-35	Obsessive-compulsive disorder (inpatient)	23	0.07 (0.07-0.07)	10	0.06 (0.06-0.06)	13	0.08 (0.08-0.08)
1995-2004	30-35	Obsessive-compulsive disorder (inpatient)	111	0.31 (0.31-0.31)	47	0.25 (0.25-0.25)	64	0.37 (0.37-0.37)
2005-2016	30-35	Obsessive-compulsive disorder (inpatient)	232	0.65 (0.65-0.65)	96	0.53 (0.53-0.53)	136	0.78 (0.78-0.78)
1970-2016	35-40	Obsessive-compulsive disorder (inpatient)	421	0.26 (0.26-0.26)	192	0.24 (0.24-0.24)	229	0.29 (0.29-0.29)
1970-1984	35-40	Obsessive-compulsive disorder (inpatient)	104	0.21 (0.21-0.21)	42	0.17 (0.17-0.17)	62	0.25 (0.25-0.25)
1985-1994	35-40	Obsessive-compulsive disorder (inpatient)	34	0.10 (0.10-0.10)	16	0.09 (0.09-0.09)	18	0.10 (0.10-0.10)
1995-2004	35-40	Obsessive-compulsive disorder (inpatient)	92	0.26 (0.26-0.26)	50	0.27 (0.27-0.27)	42	0.24 (0.24-0.24)
2005-2016	35-40	Obsessive-compulsive disorder (inpatient)	191	0.49 (0.49-0.49)	84	0.43 (0.42-0.43)	107	0.56 (0.56-0.56)
1970-2016	40-45	Obsessive-compulsive disorder (inpatient)	334	0.21 (0.21-0.21)	154	0.20 (0.20-0.20)	180	0.23 (0.23-0.23)
1970-1984	40-45	Obsessive-compulsive disorder (inpatient)	111	0.26 (0.26-0.26)	45	0.21 (0.21-0.21)	66	0.30 (0.30-0.30)
1985-1994	40-45	Obsessive-compulsive disorder (inpatient)	26	0.07 (0.07-0.07)	15	0.08 (0.08-0.08)	11	0.06 (0.06-0.06)
1995-2004	40-45	Obsessive-compulsive disorder (inpatient)	73	0.22 (0.21-0.22)	31	0.18 (0.18-0.18)	42	0.25 (0.25-0.25)
2005-2016	40-45	Obsessive-compulsive disorder (inpatient)	124	0.30 (0.30-0.30)	63	0.30 (0.30-0.30)	61	0.30 (0.30-0.30)
1970-2016	45-50	Obsessive-compulsive disorder (inpatient)	250	0.16 (0.16-0.16)	103	0.14 (0.14-0.14)	147	0.19 (0.19-0.19)
1970-1984	45-50	Obsessive-compulsive disorder (inpatient)	77	0.18 (0.18-0.18)	29	0.14 (0.14-0.14)	48	0.23 (0.23-0.23)
1985-1994	45-50	Obsessive-compulsive disorder (inpatient)	21	0.06 (0.06-0.06)	6	0.04 (0.04-0.04)	15	0.09 (0.09-0.09)
1995-2004	45-50	Obsessive-compulsive disorder (inpatient)	50	0.15 (0.15-0.15)	20	0.12 (0.12-0.12)	30	0.18 (0.18-0.18)

			Both sexes		Males		Females	
Years	Age	Mental disorder	Cases	IR (95% CI)	Cases	IR (95% CI)	Cases	IR (95% CI)
2005-2016	45-50	Obsessive-compulsive disorder (inpatient)	102	0.24 (0.24-0.24)	48	0.23 (0.23-0.23)	54	0.26 (0.26-0.26)
1970-2016	50-55	Obsessive-compulsive disorder (inpatient)	214	0.15 (0.15-0.15)	88	0.12 (0.12-0.12)	126	0.17 (0.17-0.17)
1970-1984	50-55	Obsessive-compulsive disorder (inpatient)	73	0.17 (0.17-0.17)	28	0.13 (0.13-0.13)	45	0.21 (0.21-0.21)
1985-1994	50-55	Obsessive-compulsive disorder (inpatient)	19	0.07 (0.07-0.07)	0-4	NA	15-19	0.11 (0.11-0.11)
1995-2004	50-55	Obsessive-compulsive disorder (inpatient)	51	0.14 (0.14-0.14)	23	0.13 (0.13-0.13)	28	0.16 (0.16-0.16)
2005-2016	50-55	Obsessive-compulsive disorder (inpatient)	71	0.18 (0.18-0.18)	33	0.17 (0.17-0.17)	38	0.19 (0.19-0.19)
1970-2016	55-60	Obsessive-compulsive disorder (inpatient)	163	0.12 (0.12-0.12)	68	0.10 (0.10-0.10)	95	0.14 (0.14-0.14)
1970-1984	55-60	Obsessive-compulsive disorder (inpatient)	65	0.16 (0.16-0.16)	24	0.12 (0.12-0.12)	41	0.19 (0.19-0.19)
1985-1994	55-60	Obsessive-compulsive disorder (inpatient)	9	0.04 (0.04-0.04)	0-4	NA	5-9	0.04 (0.04-0.04)
1995-2004	55-60	Obsessive-compulsive disorder (inpatient)	25	0.08 (0.08-0.08)	12	0.08 (0.08-0.08)	13	0.08 (0.08-0.08)
2005-2016	55-60	Obsessive-compulsive disorder (inpatient)	64	0.17 (0.17-0.17)	28	0.15 (0.15-0.15)	36	0.19 (0.19-0.19)
1970-2016	60-65	Obsessive-compulsive disorder (inpatient)	104	0.08 (0.08-0.08)	36	0.06 (0.06-0.06)	68	0.10 (0.10-0.10)
1970-1984	60-65	Obsessive-compulsive disorder (inpatient)	39	0.10 (0.10-0.10)	15	0.08 (0.08-0.08)	24	0.12 (0.12-0.12)
1985-1994	60-65	Obsessive-compulsive disorder (inpatient)	16	0.06 (0.06-0.07)	5	0.04 (0.04-0.04)	11	0.09 (0.09-0.09)
1995-2004	60-65	Obsessive-compulsive disorder (inpatient)	22	0.09 (0.09-0.09)	6	0.05 (0.05-0.05)	16	0.12 (0.12-0.12)
2005-2016	60-65	Obsessive-compulsive disorder (inpatient)	27	0.07 (0.07-0.07)	10	0.05 (0.05-0.05)	17	0.09 (0.09-0.09)
1970-2016	65-70	Obsessive-compulsive disorder (inpatient)	74	0.06 (0.06-0.06)	22	0.04 (0.04-0.04)	52	0.09 (0.09-0.09)
1970-1984	65-70	Obsessive-compulsive disorder (inpatient)	30	0.08 (0.08-0.09)	8	0.05 (0.05-0.05)	22	0.12 (0.12-0.12)
1985-1994	65-70	Obsessive-compulsive disorder (inpatient)	7	0.03 (0.03-0.03)	0-4	NA	0-4	NA
1995-2004	65-70	Obsessive-compulsive disorder (inpatient)	19	0.09 (0.09-0.09)	6	0.06 (0.06-0.06)	13	0.11 (0.11-0.11)
2005-2016	65-70	Obsessive-compulsive disorder (inpatient)	18	0.05 (0.05-0.05)	5	0.03 (0.03-0.03)	13	0.07 (0.07-0.07)
1970-2016	70-75	Obsessive-compulsive disorder (inpatient)	52	0.05 (0.05-0.05)	14	0.03 (0.03-0.03)	38	0.07 (0.07-0.07)
1970-1984	70-75	Obsessive-compulsive disorder (inpatient)	19	0.07 (0.07-0.07)	0-4	NA	15-19	0.10 (0.10-0.10)
1985-1994	70-75	Obsessive-compulsive disorder (inpatient)	0-4	NA	0-4	NA	0-4	NA
1995-2004	70-75	Obsessive-compulsive disorder (inpatient)	18	0.09 (0.09-0.09)	5	0.06 (0.06-0.06)	13	0.12 (0.12-0.12)
2005-2016	70-75	Obsessive-compulsive disorder (inpatient)	12	0.05 (0.05-0.05)	0-4	NA	8-12	0.06 (0.06-0.06)
1970-2016	75-80	Obsessive-compulsive disorder (inpatient)	25	0.03 (0.03-0.03)	5	0.02 (0.02-0.02)	20	0.05 (0.05-0.05)
1970-1984	75-80	Obsessive-compulsive disorder (inpatient)	6	0.03 (0.03-0.03)	0-4	NA	2-6	0.05 (0.05-0.05)
1985-1994	75-80	Obsessive-compulsive disorder (inpatient)	0-4	NA	0-4	NA	0-4	NA
1995-2004	75-80	Obsessive-compulsive disorder (inpatient)	5	0.03 (0.03-0.03)	0-4	NA	0-4	NA
2005-2016	75-80	Obsessive-compulsive disorder (inpatient)	12	0.06 (0.06-0.06)	0-4	NA	8-12	0.08 (0.08-0.08)
1970-2016	80-85	Obsessive-compulsive disorder (inpatient)	10	0.02 (0.02-0.02)	0-4	NA	6-10	0.03 (0.03-0.03)
1970-1984	80-85	Obsessive-compulsive disorder (inpatient)	0-4	NA	0-4	NA	0-4	NA
1985-1994	80-85	Obsessive-compulsive disorder (inpatient)	0-4	NA	0-4	NA	0-4	NA
1995-2004	80-85	Obsessive-compulsive disorder (inpatient)	0-4	NA	0-4	NA	0-4	NA
2005-2016	80-85	Obsessive-compulsive disorder (inpatient)	6	0.04 (0.04-0.04)	0-4	NA	2-6	0.06 (0.06-0.06)
1970-2016	85-90	Obsessive-compulsive disorder (inpatient)	0-4	NA	0-4	NA	0-4	NA
1970-1984	85-90	Obsessive-compulsive disorder (inpatient)	0-4	NA	0-4	NA	0-4	NA
1985-1994	85-90	Obsessive-compulsive disorder (inpatient)	0-4	NA	0-4	NA	0-4	NA
1995-2004	85-90	Obsessive-compulsive disorder (inpatient)	0-4	NA	0-4	NA	0-4	NA

			Both sexes		Males		Females	
**Years**	**Age**	**Mental disorder**	**Cases**	**IR (95% CI)**	**Cases**	**IR (95% CI)**	**Cases**	**IR (95% CI)**
2005-2016	85-90	Obsessive-compulsive disorder (inpatient)	0-4	NA	0-4	NA	0-4	NA
1970-2016	90-95	Obsessive-compulsive disorder (inpatient)	0-4	NA	0-4	NA	0-4	NA
1970-1984	90-95	Obsessive-compulsive disorder (inpatient)	0-4	NA	0-4	NA	0-4	NA
1985-1994	90-95	Obsessive-compulsive disorder (inpatient)	0-4	NA	0-4	NA	0-4	NA
1995-2004	90-95	Obsessive-compulsive disorder (inpatient)	0-4	NA	0-4	NA	0-4	NA
2005-2016	90-95	Obsessive-compulsive disorder (inpatient)	0-4	NA	0-4	NA	0-4	NA
1970-2016	95-100	Obsessive-compulsive disorder (inpatient)	0-4	NA	0-4	NA	0-4	NA
1970-1984	95-100	Obsessive-compulsive disorder (inpatient)	0-4	NA	0-4	NA	0-4	NA
1985-1994	95-100	Obsessive-compulsive disorder (inpatient)	0-4	NA	0-4	NA	0-4	NA
1995-2004	95-100	Obsessive-compulsive disorder (inpatient)	0-4	NA	0-4	NA	0-4	NA
2005-2016	95-100	Obsessive-compulsive disorder (inpatient)	0-4	NA	0-4	NA	0-4	NA
1970-2016	1-5	Eating disorders	27	0.02 (0.02-0.02)	12	0.02 (0.02-0.02)	15	0.03 (0.03-0.03)
1970-1984	1-5	Eating disorders	0-4	NA	0-4	NA	0-4	NA
1985-1994	1-5	Eating disorders	6	0.03 (0.03-0.03)	0-4	NA	0-4	NA
1995-2004	1-5	Eating disorders	8	0.03 (0.03-0.03)	4-8	0.04 (0.04-0.04)	0-4	NA
2005-2016	1-5	Eating disorders	13	0.04 (0.04-0.04)	5	0.03 (0.03-0.03)	8	0.05 (0.05-0.05)
1970-2016	5-10	Eating disorders	142	0.09 (0.09-0.09)	53	0.07 (0.07-0.07)	89	0.12 (0.12-0.12)
1970-1984	5-10	Eating disorders	6	0.01 (0.01-0.01)	0-4	NA	0-4	NA
1985-1994	5-10	Eating disorders	0-4	NA	0-4	NA	0-4	NA
1995-2004	5-10	Eating disorders	55	0.17 (0.17-0.17)	23	0.14 (0.14-0.14)	32	0.20 (0.20-0.20)
2005-2016	5-10	Eating disorders	78	0.20 (0.20-0.20)	27	0.13 (0.13-0.13)	51	0.27 (0.27-0.27)
1970-2016	10-15	Eating disorders	3779	2.39 (2.39-2.39)	448	0.55 (0.55-0.55)	3331	4.32 (4.31-4.32)
1970-1984	10-15	Eating disorders	127	0.22 (0.22-0.22)	25	0.09 (0.09-0.09)	102	0.37 (0.37-0.37)
1985-1994	10-15	Eating disorders	155	0.49 (0.49-0.49)	19	0.12 (0.12-0.12)	136	0.88 (0.88-0.88)
1995-2004	10-15	Eating disorders	901	3.05 (3.04-3.05)	90	0.59 (0.59-0.59)	811	5.63 (5.62-5.64)
2005-2016	10-15	Eating disorders	2596	6.47 (6.47-6.48)	314	1.53 (1.53-1.53)	2282	11.68 (11.66-11.69)
1970-2016	15-20	Eating disorders	8172	5.09 (5.09-5.10)	441	0.53 (0.53-0.54)	7731	9.91 (9.91-9.92)
1970-1984	15-20	Eating disorders	263	0.46 (0.46-0.46)	23	0.08 (0.08-0.08)	240	0.86 (0.86-0.87)
1985-1994	15-20	Eating disorders	341	0.97 (0.96-0.97)	14	0.08 (0.08-0.08)	327	1.90 (1.90-1.91)
1995-2004	15-20	Eating disorders	2405	8.47 (8.46-8.48)	85	0.58 (0.58-0.58)	2320	16.79 (16.76-16.81)
2005-2016	15-20	Eating disorders	5163	13.02 (13.01-13.03)	319	1.56 (1.56-1.56)	4844	25.23 (25.20-25.27)
1970-2016	20-25	Eating disorders	5643	3.51 (3.50-3.51)	215	0.26 (0.26-0.26)	5428	7.00 (6.99-7.00)
1970-1984	20-25	Eating disorders	186	0.34 (0.33-0.34)	12	0.04 (0.04-0.04)	174	0.65 (0.65-0.65)
1985-1994	20-25	Eating disorders	256	0.69 (0.69-0.69)	6	0.03 (0.03-0.03)	250	1.40 (1.40-1.40)
1995-2004	20-25	Eating disorders	1920	6.21 (6.21-6.22)	58	0.36 (0.36-0.36)	1862	12.52 (12.50-12.54)
2005-2016	20-25	Eating disorders	3281	8.81 (8.80-8.82)	139	0.72 (0.72-0.72)	3142	17.49 (17.46-17.51)
1970-2016	25-30	Eating disorders	2585	1.60 (1.60-1.60)	107	0.13 (0.13-0.13)	2478	3.17 (3.17-3.17)
1970-1984	25-30	Eating disorders	91	0.16 (0.16-0.16)	0-4	NA	87-91	0.32 (0.32-0.32)
1985-1994	25-30	Eating disorders	129	0.35 (0.35-0.35)	7	0.04 (0.04-0.04)	122	0.69 (0.69-0.69)
1995-2004	25-30	Eating disorders	875	2.60 (2.60-2.60)	21	0.12 (0.12-0.12)	854	5.27 (5.27-5.28)

			Both sexes		Males		Females	
**Years**	**Age**	**Mental disorder**	**Cases**	**IR (95% CI)**	**Cases**	**IR (95% CI)**	**Cases**	**IR (95% CI)**
2005-2016	25-30	Eating disorders	1490	4.33 (4.32-4.33)	76	0.43 (0.43-0.43)	1414	8.48 (8.47-8.49)
1970-2016	30-35	Eating disorders	1340	0.83 (0.83-0.84)	81	0.10 (0.10-0.10)	1259	1.61 (1.61-1.61)
1970-1984	30-35	Eating disorders	54	0.10 (0.10-0.10)	0-4	NA	50-54	0.19 (0.19-0.19)
1985-1994	30-35	Eating disorders	58	0.17 (0.17-0.17)	5	0.03 (0.03-0.03)	53	0.31 (0.31-0.32)
1995-2004	30-35	Eating disorders	451	1.25 (1.25-1.25)	22	0.12 (0.12-0.12)	429	2.46 (2.46-2.46)
2005-2016	30-35	Eating disorders	777	2.19 (2.19-2.19)	51	0.28 (0.28-0.28)	726	4.21 (4.21-4.22)
1970-2016	35-40	Eating disorders	849	0.53 (0.53-0.53)	55	0.07 (0.07-0.07)	794	1.02 (1.02-1.02)
1970-1984	35-40	Eating disorders	34	0.07 (0.07-0.07)	0-4	NA	30-34	0.13 (0.13-0.13)
1985-1994	35-40	Eating disorders	34	0.10 (0.10-0.10)	0-4	NA	30-34	0.17 (0.17-0.17)
1995-2004	35-40	Eating disorders	261	0.72 (0.72-0.73)	13	0.07 (0.07-0.07)	248	1.41 (1.41-1.41)
2005-2016	35-40	Eating disorders	520	1.35 (1.35-1.35)	36	0.18 (0.18-0.18)	484	2.57 (2.57-2.58)
1970-2016	40-45	Eating disorders	449	0.29 (0.29-0.29)	28	0.04 (0.04-0.04)	421	0.55 (0.55-0.55)
1970-1984	40-45	Eating disorders	11	0.03 (0.03-0.03)	0-4	NA	7-11	0.05 (0.05-0.05)
1985-1994	40-45	Eating disorders	29	0.08 (0.08-0.08)	0-4	NA	25-29	0.14 (0.14-0.14)
1995-2004	40-45	Eating disorders	111	0.33 (0.33-0.33)	11	0.06 (0.06-0.06)	100	0.60 (0.60-0.60)
2005-2016	40-45	Eating disorders	298	0.72 (0.71-0.72)	13	0.06 (0.06-0.06)	285	1.40 (1.40-1.40)
1970-2016	45-50	Eating disorders	330	0.22 (0.22-0.22)	21	0.03 (0.03-0.03)	309	0.41 (0.41-0.41)
1970-1984	45-50	Eating disorders	16	0.04 (0.04-0.04)	0-4	NA	12-16	0.07 (0.07-0.07)
1985-1994	45-50	Eating disorders	21	0.06 (0.06-0.06)	0-4	NA	17-21	0.12 (0.12-0.12)
1995-2004	45-50	Eating disorders	86	0.25 (0.25-0.25)	5	0.03 (0.03-0.03)	81	0.48 (0.48-0.48)
2005-2016	45-50	Eating disorders	207	0.49 (0.49-0.50)	14	0.07 (0.07-0.07)	193	0.93 (0.93-0.94)
1970-2016	50-55	Eating disorders	212	0.15 (0.15-0.15)	23	0.03 (0.03-0.03)	189	0.26 (0.26-0.26)
1970-1984	50-55	Eating disorders	15	0.04 (0.04-0.04)	0-4	NA	11-15	0.06 (0.06-0.06)
1985-1994	50-55	Eating disorders	15	0.05 (0.05-0.05)	0-4	NA	11-15	0.11 (0.11-0.11)
1995-2004	50-55	Eating disorders	62	0.18 (0.18-0.18)	6	0.03 (0.03-0.03)	56	0.32 (0.32-0.32)
2005-2016	50-55	Eating disorders	120	0.30 (0.30-0.30)	15	0.08 (0.08-0.08)	105	0.53 (0.53-0.53)
1970-2016	55-60	Eating disorders	115	0.08 (0.08-0.08)	14	0.02 (0.02-0.02)	101	0.14 (0.14-0.14)
1970-1984	55-60	Eating disorders	5	0.01 (0.01-0.01)	0-4	NA	0-4	NA
1985-1994	55-60	Eating disorders	0-4	NA	0-4	NA	0-4	NA
1995-2004	55-60	Eating disorders	34	0.11 (0.11-0.11)	0-4	NA	30-34	0.19 (0.19-0.19)
2005-2016	55-60	Eating disorders	72	0.19 (0.19-0.19)	9	0.05 (0.05-0.05)	63	0.33 (0.32-0.33)
1970-2016	60-65	Eating disorders	61	0.05 (0.05-0.05)	5	0.01 (0.01-0.01)	56	0.08 (0.08-0.08)
1970-1984	60-65	Eating disorders	7	0.02 (0.02-0.02)	0-4	NA	3-7	0.02 (0.02-0.02)
1985-1994	60-65	Eating disorders	0-4	NA	0-4	NA	0-4	NA
1995-2004	60-65	Eating disorders	13	0.05 (0.05-0.05)	0-4	NA	9-13	0.10 (0.10-0.10)
2005-2016	60-65	Eating disorders	39	0.10 (0.10-0.10)	0-4	NA	35-39	0.18 (0.18-0.18)
1970-2016	65-70	Eating disorders	34	0.03 (0.03-0.03)	6	0.01 (0.01-0.01)	28	0.05 (0.05-0.05)
1970-1984	65-70	Eating disorders	0-4	NA	0-4	NA	0-4	NA
1985-1994	65-70	Eating disorders	5	0.02 (0.02-0.02)	0-4	NA	1-5	0.04 (0.04-0.04)
1995-2004	65-70	Eating disorders	7	0.03 (0.03-0.03)	0-4	NA	3-7	0.05 (0.05-0.05)

			Both sexes		Males		Females	
Years	Age	Mental disorder	Cases	IR (95% CI)	Cases	IR (95% CI)	Cases	IR (95% CI)
2005-2016	65-70	Eating disorders	21	0.06 (0.06-0.06)	5	0.03 (0.03-0.03)	16	0.09 (0.09-0.09)
1970-2016	70-75	Eating disorders	25	0.03 (0.03-0.03)	0-4	NA	21-25	0.04 (0.04-0.04)
1970-1984	70-75	Eating disorders	0-4	NA	0-4	NA	0-4	NA
1985-1994	70-75	Eating disorders	0-4	NA	0-4	NA	0-4	NA
1995-2004	70-75	Eating disorders	0-4	NA	0-4	NA	0-4	NA
2005-2016	70-75	Eating disorders	17	0.06 (0.06-0.06)	0-4	NA	13-17	0.11 (0.11-0.11)
1970-2016	75-80	Eating disorders	16	0.02 (0.02-0.02)	0-4	NA	12-16	0.03 (0.03-0.03)
1970-1984	75-80	Eating disorders	0-4	NA	0-4	NA	0-4	NA
1985-1994	75-80	Eating disorders	0-4	NA	0-4	NA	0-4	NA
1995-2004	75-80	Eating disorders	10	0.06 (0.06-0.06)	0-4	NA	6-10	0.08 (0.08-0.08)
2005-2016	75-80	Eating disorders	0-4	NA	0-4	NA	0-4	NA
1970-2016	80-85	Eating disorders	11	0.02 (0.02-0.02)	0-4	NA	7-11	0.03 (0.03-0.03)
1970-1984	80-85	Eating disorders	0-4	NA	0-4	NA	0-4	NA
1985-1994	80-85	Eating disorders	0-4	NA	0-4	NA	0-4	NA
1995-2004	80-85	Eating disorders	0-4	NA	0-4	NA	0-4	NA
2005-2016	80-85	Eating disorders	7	0.05 (0.05-0.05)	0-4	NA	3-7	0.07 (0.07-0.07)
1970-2016	85-90	Eating disorders	5	0.02 (0.02-0.02)	0-4	NA	0-4	NA
1970-1984	85-90	Eating disorders	0-4	NA	0-4	NA	0-4	NA
1985-1994	85-90	Eating disorders	0-4	NA	0-4	NA	0-4	NA
1995-2004	85-90	Eating disorders	0-4	NA	0-4	NA	0-4	NA
2005-2016	85-90	Eating disorders	0-4	NA	0-4	NA	0-4	NA
1970-2016	90-95	Eating disorders	0-4	NA	0-4	NA	0-4	NA
1970-1984	90-95	Eating disorders	0-4	NA	0-4	NA	0-4	NA
1985-1994	90-95	Eating disorders	0-4	NA	0-4	NA	0-4	NA
1995-2004	90-95	Eating disorders	0-4	NA	0-4	NA	0-4	NA
2005-2016	90-95	Eating disorders	0-4	NA	0-4	NA	0-4	NA
1970-2016	95-100	Eating disorders	0-4	NA	0-4	NA	0-4	NA
1970-1984	95-100	Eating disorders	0-4	NA	0-4	NA	0-4	NA
1985-1994	95-100	Eating disorders	0-4	NA	0-4	NA	0-4	NA
1995-2004	95-100	Eating disorders	0-4	NA	0-4	NA	0-4	NA
2005-2016	95-100	Eating disorders	0-4	NA	0-4	NA	0-4	NA
1970-2016	1-5	Eating disorders (inpatient)	6	0.00 (0.00-0.00)	0-4	NA	0-4	NA
1970-1984	1-5	Eating disorders (inpatient)	0-4	NA	0-4	NA	0-4	NA
1985-1994	1-5	Eating disorders (inpatient)	6	0.03 (0.03-0.03)	0-4	NA	0-4	NA
1995-2004	1-5	Eating disorders (inpatient)	0-4	NA	0-4	NA	0-4	NA
2005-2016	1-5	Eating disorders (inpatient)	0-4	NA	0-4	NA	0-4	NA
1970-2016	5-10	Eating disorders (inpatient)	33	0.02 (0.02-0.02)	10	0.01 (0.01-0.01)	23	0.03 (0.03-0.03)
1970-1984	5-10	Eating disorders (inpatient)	6	0.01 (0.01-0.01)	0-4	NA	0-4	NA
1985-1994	5-10	Eating disorders (inpatient)	0-4	NA	0-4	NA	0-4	NA
1995-2004	5-10	Eating disorders (inpatient)	14	0.04 (0.04-0.04)	5	0.03 (0.03-0.03)	9	0.06 (0.06-0.06)

			Both sexes		Males		Females	
**Years**	**Age**	**Mental disorder**	**Cases**	**IR (95% CI)**	**Cases**	**IR (95% CI)**	**Cases**	**IR (95% CI)**
2005-2016	5-10	Eating disorders (inpatient)	10	0.03 (0.03-0.03)	0-4	NA	6-10	0.04 (0.04-0.04)
1970-2016	10-15	Eating disorders (inpatient)	1093	0.69 (0.69-0.69)	138	0.17 (0.17-0.17)	955	1.24 (1.24-1.24)
1970-1984	10-15	Eating disorders (inpatient)	127	0.22 (0.22-0.22)	25	0.09 (0.09-0.09)	102	0.37 (0.37-0.37)
1985-1994	10-15	Eating disorders (inpatient)	139	0.44 (0.44-0.44)	19	0.12 (0.12-0.12)	120	0.78 (0.78-0.78)
1995-2004	10-15	Eating disorders (inpatient)	257	0.87 (0.87-0.87)	27	0.18 (0.18-0.18)	230	1.60 (1.59-1.60)
2005-2016	10-15	Eating disorders (inpatient)	570	1.42 (1.42-1.42)	67	0.33 (0.33-0.33)	503	2.57 (2.57-2.57)
1970-2016	15-20	Eating disorders (inpatient)	2159	1.34 (1.34-1.34)	124	0.15 (0.15-0.15)	2035	2.60 (2.60-2.60)
1970-1984	15-20	Eating disorders (inpatient)	263	0.46 (0.46-0.46)	23	0.08 (0.08-0.08)	240	0.86 (0.86-0.87)
1985-1994	15-20	Eating disorders (inpatient)	286	0.81 (0.81-0.81)	14	0.08 (0.08-0.08)	272	1.58 (1.58-1.58)
1995-2004	15-20	Eating disorders (inpatient)	573	2.01 (2.01-2.02)	25	0.17 (0.17-0.17)	548	3.95 (3.94-3.96)
2005-2016	15-20	Eating disorders (inpatient)	1037	2.60 (2.60-2.61)	62	0.30 (0.30-0.30)	975	5.04 (5.03-5.04)
1970-2016	20-25	Eating disorders (inpatient)	1543	0.96 (0.96-0.96)	64	0.08 (0.08-0.08)	1479	1.90 (1.90-1.90)
1970-1984	20-25	Eating disorders (inpatient)	186	0.34 (0.33-0.34)	12	0.04 (0.04-0.04)	174	0.65 (0.65-0.65)
1985-1994	20-25	Eating disorders (inpatient)	228	0.61 (0.61-0.61)	6	0.03 (0.03-0.03)	222	1.24 (1.24-1.24)
1995-2004	20-25	Eating disorders (inpatient)	534	1.72 (1.72-1.73)	15	0.09 (0.09-0.09)	519	3.47 (3.47-3.48)
2005-2016	20-25	Eating disorders (inpatient)	595	1.59 (1.58-1.59)	31	0.16 (0.16-0.16)	564	3.10 (3.09-3.10)
1970-2016	25-30	Eating disorders (inpatient)	741	0.46 (0.46-0.46)	35	0.04 (0.04-0.04)	706	0.90 (0.90-0.90)
1970-1984	25-30	Eating disorders (inpatient)	91	0.16 (0.16-0.16)	0-4	NA	87-91	0.32 (0.32-0.32)
1985-1994	25-30	Eating disorders (inpatient)	110	0.30 (0.30-0.30)	6	0.03 (0.03-0.03)	104	0.59 (0.58-0.59)
1995-2004	25-30	Eating disorders (inpatient)	261	0.77 (0.77-0.78)	9	0.05 (0.05-0.05)	252	1.55 (1.55-1.55)
2005-2016	25-30	Eating disorders (inpatient)	279	0.80 (0.80-0.80)	17	0.10 (0.10-0.10)	262	1.55 (1.55-1.55)
1970-2016	30-35	Eating disorders (inpatient)	417	0.26 (0.26-0.26)	23	0.03 (0.03-0.03)	394	0.50 (0.50-0.50)
1970-1984	30-35	Eating disorders (inpatient)	54	0.10 (0.10-0.10)	0-4	NA	50-54	0.19 (0.19-0.19)
1985-1994	30-35	Eating disorders (inpatient)	55	0.16 (0.16-0.16)	5	0.03 (0.03-0.03)	50	0.30 (0.30-0.30)
1995-2004	30-35	Eating disorders (inpatient)	161	0.45 (0.45-0.45)	8	0.04 (0.04-0.04)	153	0.88 (0.88-0.88)
2005-2016	30-35	Eating disorders (inpatient)	147	0.41 (0.41-0.41)	7	0.04 (0.04-0.04)	140	0.80 (0.80-0.80)
1970-2016	35-40	Eating disorders (inpatient)	281	0.18 (0.18-0.18)	19	0.02 (0.02-0.02)	262	0.34 (0.33-0.34)
1970-1984	35-40	Eating disorders (inpatient)	34	0.07 (0.07-0.07)	0-4	NA	30-34	0.13 (0.13-0.13)
1985-1994	35-40	Eating disorders (inpatient)	33	0.09 (0.09-0.09)	0-4	NA	29-33	0.17 (0.17-0.17)
1995-2004	35-40	Eating disorders (inpatient)	102	0.28 (0.28-0.28)	0-4	NA	98-102	0.56 (0.56-0.56)
2005-2016	35-40	Eating disorders (inpatient)	112	0.29 (0.29-0.29)	10	0.05 (0.05-0.05)	102	0.54 (0.54-0.54)
1970-2016	40-45	Eating disorders (inpatient)	131	0.08 (0.08-0.08)	9	0.01 (0.01-0.01)	122	0.16 (0.16-0.16)
1970-1984	40-45	Eating disorders (inpatient)	11	0.03 (0.03-0.03)	0-4	NA	7-11	0.05 (0.05-0.05)
1985-1994	40-45	Eating disorders (inpatient)	27	0.07 (0.07-0.07)	0-4	NA	23-27	0.13 (0.13-0.13)
1995-2004	40-45	Eating disorders (inpatient)	41	0.12 (0.12-0.12)	0-4	NA	37-41	0.23 (0.23-0.23)
2005-2016	40-45	Eating disorders (inpatient)	52	0.12 (0.12-0.12)	0-4	NA	48-52	0.24 (0.24-0.24)
1970-2016	45-50	Eating disorders (inpatient)	109	0.07 (0.07-0.07)	6	0.01 (0.01-0.01)	103	0.14 (0.14-0.14)
1970-1984	45-50	Eating disorders (inpatient)	16	0.04 (0.04-0.04)	0-4	NA	12-16	0.07 (0.07-0.07)
1985-1994	45-50	Eating disorders (inpatient)	17	0.05 (0.05-0.05)	0-4	NA	13-17	0.09 (0.09-0.09)
1995-2004	45-50	Eating disorders (inpatient)	29	0.09 (0.09-0.09)	0-4	NA	25-29	0.16 (0.16-0.16)

			Both sexes		Males		Females	
Years	Age	Mental disorder	Cases	IR (95% CI)	Cases	IR (95% CI)	Cases	IR (95% CI)
2005-2016	45-50	Eating disorders (inpatient)	47	0.11 (0.11-0.11)	0-4	NA	43-47	0.22 (0.22-0.22)
1970-2016	50-55	Eating disorders (inpatient)	98	0.07 (0.07-0.07)	10	0.01 (0.01-0.01)	88	0.12 (0.12-0.12)
1970-1984	50-55	Eating disorders (inpatient)	15	0.04 (0.04-0.04)	0-4	NA	11-15	0.06 (0.06-0.06)
1985-1994	50-55	Eating disorders (inpatient)	14	0.05 (0.05-0.05)	0-4	NA	10-14	0.10 (0.10-0.10)
1995-2004	50-55	Eating disorders (inpatient)	33	0.09 (0.09-0.09)	0-4	NA	29-33	0.17 (0.17-0.17)
2005-2016	50-55	Eating disorders (inpatient)	36	0.09 (0.09-0.09)	5	0.03 (0.03-0.03)	31	0.16 (0.16-0.16)
1970-2016	55-60	Eating disorders (inpatient)	44	0.03 (0.03-0.03)	0-4	NA	40-44	0.06 (0.06-0.06)
1970-1984	55-60	Eating disorders (inpatient)	5	0.01 (0.01-0.01)	0-4	NA	0-4	NA
1985-1994	55-60	Eating disorders (inpatient)	0-4	NA	0-4	NA	0-4	NA
1995-2004	55-60	Eating disorders (inpatient)	13	0.04 (0.04-0.04)	0-4	NA	9-13	0.08 (0.08-0.08)
2005-2016	55-60	Eating disorders (inpatient)	22	0.06 (0.06-0.06)	0-4	NA	18-22	0.10 (0.10-0.10)
1970-2016	60-65	Eating disorders (inpatient)	22	0.02 (0.02-0.02)	0-4	NA	18-22	0.03 (0.03-0.03)
1970-1984	60-65	Eating disorders (inpatient)	7	0.02 (0.02-0.02)	0-4	NA	3-7	0.02 (0.02-0.02)
1985-1994	60-65	Eating disorders (inpatient)	0-4	NA	0-4	NA	0-4	NA
1995-2004	60-65	Eating disorders (inpatient)	0-4	NA	0-4	NA	0-4	NA
2005-2016	60-65	Eating disorders (inpatient)	11	0.03 (0.03-0.03)	0-4	NA	7-11	0.05 (0.05-0.05)
1970-2016	65-70	Eating disorders (inpatient)	15	0.01 (0.01-0.01)	0-4	NA	11-15	0.02 (0.02-0.02)
1970-1984	65-70	Eating disorders (inpatient)	0-4	NA	0-4	NA	0-4	NA
1985-1994	65-70	Eating disorders (inpatient)	5	0.02 (0.02-0.02)	0-4	NA	1-5	0.04 (0.04-0.04)
1995-2004	65-70	Eating disorders (inpatient)	0-4	NA	0-4	NA	0-4	NA
2005-2016	65-70	Eating disorders (inpatient)	8	0.02 (0.02-0.02)	0-4	NA	4-8	0.04 (0.04-0.04)
1970-2016	70-75	Eating disorders (inpatient)	13	0.01 (0.01-0.01)	0-4	NA	9-13	0.02 (0.02-0.02)
1970-1984	70-75	Eating disorders (inpatient)	0-4	NA	0-4	NA	0-4	NA
1985-1994	70-75	Eating disorders (inpatient)	0-4	NA	0-4	NA	0-4	NA
1995-2004	70-75	Eating disorders (inpatient)	0-4	NA	0-4	NA	0-4	NA
2005-2016	70-75	Eating disorders (inpatient)	8	0.03 (0.03-0.03)	0-4	NA	4-8	0.04 (0.04-0.04)
1970-2016	75-80	Eating disorders (inpatient)	7	0.01 (0.01-0.01)	0-4	NA	3-7	0.01 (0.01-0.01)
1970-1984	75-80	Eating disorders (inpatient)	0-4	NA	0-4	NA	0-4	NA
1985-1994	75-80	Eating disorders (inpatient)	0-4	NA	0-4	NA	0-4	NA
1995-2004	75-80	Eating disorders (inpatient)	0-4	NA	0-4	NA	0-4	NA
2005-2016	75-80	Eating disorders (inpatient)	0-4	NA	0-4	NA	0-4	NA
1970-2016	80-85	Eating disorders (inpatient)	6	0.01 (0.01-0.01)	0-4	NA	2-6	0.02 (0.02-0.02)
1970-1984	80-85	Eating disorders (inpatient)	0-4	NA	0-4	NA	0-4	NA
1985-1994	80-85	Eating disorders (inpatient)	0-4	NA	0-4	NA	0-4	NA
1995-2004	80-85	Eating disorders (inpatient)	0-4	NA	0-4	NA	0-4	NA
2005-2016	80-85	Eating disorders (inpatient)	0-4	NA	0-4	NA	0-4	NA
1970-2016	85-90	Eating disorders (inpatient)	0-4	NA	0-4	NA	0-4	NA
1970-1984	85-90	Eating disorders (inpatient)	0-4	NA	0-4	NA	0-4	NA
1985-1994	85-90	Eating disorders (inpatient)	0-4	NA	0-4	NA	0-4	NA
1995-2004	85-90	Eating disorders (inpatient)	0-4	NA	0-4	NA	0-4	NA

			Both sexes		Males		Females	
Years	Age	Mental disorder	Cases	IR (95% CI)	Cases	IR (95% CI)	Cases	IR (95% CI)
2005-2016	85-90	Eating disorders (inpatient)	0-4	NA	0-4	NA	0-4	NA
1970-2016	90-95	Eating disorders (inpatient)	0-4	NA	0-4	NA	0-4	NA
1970-1984	90-95	Eating disorders (inpatient)	0-4	NA	0-4	NA	0-4	NA
1985-1994	90-95	Eating disorders (inpatient)	0-4	NA	0-4	NA	0-4	NA
1995-2004	90-95	Eating disorders (inpatient)	0-4	NA	0-4	NA	0-4	NA
2005-2016	90-95	Eating disorders (inpatient)	0-4	NA	0-4	NA	0-4	NA
1970-2016	95-100	Eating disorders (inpatient)	0-4	NA	0-4	NA	0-4	NA
1970-1984	95-100	Eating disorders (inpatient)	0-4	NA	0-4	NA	0-4	NA
1985-1994	95-100	Eating disorders (inpatient)	0-4	NA	0-4	NA	0-4	NA
1995-2004	95-100	Eating disorders (inpatient)	0-4	NA	0-4	NA	0-4	NA
2005-2016	95-100	Eating disorders (inpatient)	0-4	NA	0-4	NA	0-4	NA
1970-2016	1-5	Anorexia nervosa	0-4	NA	0-4	NA	0-4	NA
1970-1984	1-5	Anorexia nervosa	0-4	NA	0-4	NA	0-4	NA
1985-1994	1-5	Anorexia nervosa	0-4	NA	0-4	NA	0-4	NA
1995-2004	1-5	Anorexia nervosa	0-4	NA	0-4	NA	0-4	NA
2005-2016	1-5	Anorexia nervosa	0-4	NA	0-4	NA	0-4	NA
1970-2016	5-10	Anorexia nervosa	25	0.02 (0.02-0.02)	7	0.01 (0.01-0.01)	18	0.02 (0.02-0.02)
1970-1984	5-10	Anorexia nervosa	0-4	NA	0-4	NA	0-4	NA
1985-1994	5-10	Anorexia nervosa	0-4	NA	0-4	NA	0-4	NA
1995-2004	5-10	Anorexia nervosa	11	0.03 (0.03-0.03)	0-4	NA	7-11	0.04 (0.04-0.04)
2005-2016	5-10	Anorexia nervosa	9	0.02 (0.02-0.02)	0-4	NA	5-9	0.03 (0.03-0.03)
1970-2016	10-15	Anorexia nervosa	1916	1.21 (1.21-1.21)	203	0.25 (0.25-0.25)	1713	2.22 (2.22-2.22)
1970-1984	10-15	Anorexia nervosa	120	0.21 (0.21-0.21)	21	0.07 (0.07-0.07)	99	0.36 (0.36-0.36)
1985-1994	10-15	Anorexia nervosa	128	0.41 (0.41-0.41)	15	0.09 (0.09-0.09)	113	0.73 (0.73-0.73)
1995-2004	10-15	Anorexia nervosa	461	1.56 (1.56-1.56)	42	0.28 (0.28-0.28)	419	2.91 (2.90-2.91)
2005-2016	10-15	Anorexia nervosa	1207	3.01 (3.01-3.01)	125	0.61 (0.61-0.61)	1082	5.53 (5.52-5.54)
1970-2016	15-20	Anorexia nervosa	2929	1.82 (1.82-1.82)	133	0.16 (0.16-0.16)	2796	3.58 (3.58-3.58)
1970-1984	15-20	Anorexia nervosa	244	0.43 (0.43-0.43)	19	0.06 (0.06-0.06)	225	0.81 (0.81-0.81)
1985-1994	15-20	Anorexia nervosa	231	0.65 (0.65-0.65)	11	0.06 (0.06-0.06)	220	1.28 (1.28-1.28)
1995-2004	15-20	Anorexia nervosa	780	2.74 (2.74-2.75)	21	0.14 (0.14-0.14)	759	5.47 (5.47-5.48)
2005-2016	15-20	Anorexia nervosa	1674	4.21 (4.20-4.21)	82	0.40 (0.40-0.40)	1592	8.24 (8.23-8.25)
1970-2016	20-25	Anorexia nervosa	1512	0.94 (0.94-0.94)	58	0.07 (0.07-0.07)	1454	1.87 (1.87-1.87)
1970-1984	20-25	Anorexia nervosa	159	0.29 (0.29-0.29)	10	0.03 (0.03-0.03)	149	0.56 (0.55-0.56)
1985-1994	20-25	Anorexia nervosa	138	0.37 (0.37-0.37)	5	0.03 (0.03-0.03)	133	0.74 (0.74-0.74)
1995-2004	20-25	Anorexia nervosa	418	1.35 (1.35-1.35)	13	0.08 (0.08-0.08)	405	2.71 (2.71-2.71)
2005-2016	20-25	Anorexia nervosa	797	2.13 (2.13-2.13)	30	0.16 (0.16-0.16)	767	4.22 (4.22-4.23)
1970-2016	25-30	Anorexia nervosa	624	0.39 (0.38-0.39)	30	0.04 (0.04-0.04)	594	0.76 (0.76-0.76)
1970-1984	25-30	Anorexia nervosa	77	0.14 (0.14-0.14)	0-4	NA	73-77	0.27 (0.27-0.27)
1985-1994	25-30	Anorexia nervosa	61	0.17 (0.17-0.17)	5	0.03 (0.03-0.03)	56	0.32 (0.31-0.32)
1995-2004	25-30	Anorexia nervosa	173	0.51 (0.51-0.51)	0-4	NA	169-173	1.05 (1.04-1.05)

			Both sexes		Males		Females	
**Years**	**Age**	**Mental disorder**	**Cases**	**IR (95% CI)**	**Cases**	**IR (95% CI)**	**Cases**	**IR (95% CI)**
2005-2016	25-30	Anorexia nervosa	313	0.90 (0.90-0.90)	20	0.11 (0.11-0.11)	293	1.73 (1.73-1.74)
1970-2016	30-35	Anorexia nervosa	307	0.19 (0.19-0.19)	11	0.01 (0.01-0.01)	296	0.38 (0.38-0.38)
1970-1984	30-35	Anorexia nervosa	46	0.08 (0.08-0.08)	0-4	NA	42-46	0.17 (0.17-0.17)
1985-1994	30-35	Anorexia nervosa	29	0.08 (0.08-0.08)	0-4	NA	25-29	0.16 (0.16-0.16)
1995-2004	30-35	Anorexia nervosa	89	0.25 (0.25-0.25)	0-4	NA	85-89	0.49 (0.49-0.49)
2005-2016	30-35	Anorexia nervosa	143	0.40 (0.40-0.40)	5	0.03 (0.03-0.03)	138	0.79 (0.79-0.79)
1970-2016	35-40	Anorexia nervosa	184	0.12 (0.12-0.12)	12	0.01 (0.01-0.01)	172	0.22 (0.22-0.22)
1970-1984	35-40	Anorexia nervosa	28	0.06 (0.06-0.06)	0-4	NA	24-28	0.11 (0.11-0.11)
1985-1994	35-40	Anorexia nervosa	18	0.05 (0.05-0.05)	0-4	NA	14-18	0.09 (0.09-0.09)
1995-2004	35-40	Anorexia nervosa	41	0.11 (0.11-0.11)	0-4	NA	37-41	0.22 (0.22-0.22)
2005-2016	35-40	Anorexia nervosa	97	0.25 (0.25-0.25)	0-4	NA	93-97	0.49 (0.49-0.49)
1970-2016	40-45	Anorexia nervosa	91	0.06 (0.06-0.06)	0-4	NA	87-91	0.12 (0.12-0.12)
1970-1984	40-45	Anorexia nervosa	6	0.01 (0.01-0.01)	0-4	NA	2-6	0.03 (0.03-0.03)
1985-1994	40-45	Anorexia nervosa	16	0.04 (0.04-0.04)	0-4	NA	12-16	0.08 (0.08-0.08)
1995-2004	40-45	Anorexia nervosa	23	0.07 (0.07-0.07)	0-4	NA	19-23	0.14 (0.14-0.14)
2005-2016	40-45	Anorexia nervosa	46	0.11 (0.11-0.11)	0-4	NA	42-46	0.22 (0.22-0.22)
1970-2016	45-50	Anorexia nervosa	85	0.06 (0.06-0.06)	0-4	NA	81-85	0.11 (0.11-0.11)
1970-1984	45-50	Anorexia nervosa	11	0.03 (0.03-0.03)	0-4	NA	7-11	0.05 (0.05-0.05)
1985-1994	45-50	Anorexia nervosa	16	0.05 (0.05-0.05)	0-4	NA	12-16	0.09 (0.09-0.09)
1995-2004	45-50	Anorexia nervosa	20	0.06 (0.06-0.06)	0-4	NA	16-20	0.11 (0.11-0.11)
2005-2016	45-50	Anorexia nervosa	38	0.09 (0.09-0.09)	0-4	NA	34-38	0.18 (0.18-0.18)
1970-2016	50-55	Anorexia nervosa	61	0.04 (0.04-0.04)	0-4	NA	57-61	0.08 (0.08-0.08)
1970-1984	50-55	Anorexia nervosa	6	0.01 (0.01-0.01)	0-4	NA	2-6	0.03 (0.03-0.03)
1985-1994	50-55	Anorexia nervosa	10	0.04 (0.04-0.04)	0-4	NA	6-10	0.07 (0.07-0.07)
1995-2004	50-55	Anorexia nervosa	11	0.03 (0.03-0.03)	0-4	NA	7-11	0.06 (0.06-0.06)
2005-2016	50-55	Anorexia nervosa	34	0.09 (0.09-0.09)	0-4	NA	30-34	0.17 (0.17-0.17)
1970-2016	55-60	Anorexia nervosa	27	0.02 (0.02-0.02)	0-4	NA	23-27	0.04 (0.04-0.04)
1970-1984	55-60	Anorexia nervosa	0-4	NA	0-4	NA	0-4	NA
1985-1994	55-60	Anorexia nervosa	0-4	NA	0-4	NA	0-4	NA
1995-2004	55-60	Anorexia nervosa	8	0.03 (0.03-0.03)	0-4	NA	4-8	0.05 (0.05-0.05)
2005-2016	55-60	Anorexia nervosa	14	0.04 (0.04-0.04)	0-4	NA	10-14	0.07 (0.07-0.07)
1970-2016	60-65	Anorexia nervosa	13	0.01 (0.01-0.01)	0-4	NA	9-13	0.02 (0.02-0.02)
1970-1984	60-65	Anorexia nervosa	0-4	NA	0-4	NA	0-4	NA
1985-1994	60-65	Anorexia nervosa	0-4	NA	0-4	NA	0-4	NA
1995-2004	60-65	Anorexia nervosa	0-4	NA	0-4	NA	0-4	NA
2005-2016	60-65	Anorexia nervosa	9	0.02 (0.02-0.02)	0-4	NA	5-9	0.04 (0.04-0.04)
1970-2016	65-70	Anorexia nervosa	7	0.01 (0.01-0.01)	0-4	NA	3-7	0.01 (0.01-0.01)
1970-1984	65-70	Anorexia nervosa	0-4	NA	0-4	NA	0-4	NA
1985-1994	65-70	Anorexia nervosa	0-4	NA	0-4	NA	0-4	NA
1995-2004	65-70	Anorexia nervosa	0-4	NA	0-4	NA	0-4	NA

			Both sexes		Males		Females	
Years	Age	Mental disorder	Cases	IR (95% CI)	Cases	IR (95% CI)	Cases	IR (95% CI)
2005-2016	65-70	Anorexia nervosa	0-4	NA	0-4	NA	0-4	NA
1970-2016	70-75	Anorexia nervosa	0-4	NA	0-4	NA	0-4	NA
1970-1984	70-75	Anorexia nervosa	0-4	NA	0-4	NA	0-4	NA
1985-1994	70-75	Anorexia nervosa	0-4	NA	0-4	NA	0-4	NA
1995-2004	70-75	Anorexia nervosa	0-4	NA	0-4	NA	0-4	NA
2005-2016	70-75	Anorexia nervosa	0-4	NA	0-4	NA	0-4	NA
1970-2016	75-80	Anorexia nervosa	0-4	NA	0-4	NA	0-4	NA
1970-1984	75-80	Anorexia nervosa	0-4	NA	0-4	NA	0-4	NA
1985-1994	75-80	Anorexia nervosa	0-4	NA	0-4	NA	0-4	NA
1995-2004	75-80	Anorexia nervosa	0-4	NA	0-4	NA	0-4	NA
2005-2016	75-80	Anorexia nervosa	0-4	NA	0-4	NA	0-4	NA
1970-2016	80-85	Anorexia nervosa	0-4	NA	0-4	NA	0-4	NA
1970-1984	80-85	Anorexia nervosa	0-4	NA	0-4	NA	0-4	NA
1985-1994	80-85	Anorexia nervosa	0-4	NA	0-4	NA	0-4	NA
1995-2004	80-85	Anorexia nervosa	0-4	NA	0-4	NA	0-4	NA
2005-2016	80-85	Anorexia nervosa	0-4	NA	0-4	NA	0-4	NA
1970-2016	85-90	Anorexia nervosa	0-4	NA	0-4	NA	0-4	NA
1970-1984	85-90	Anorexia nervosa	0-4	NA	0-4	NA	0-4	NA
1985-1994	85-90	Anorexia nervosa	0-4	NA	0-4	NA	0-4	NA
1995-2004	85-90	Anorexia nervosa	0-4	NA	0-4	NA	0-4	NA
2005-2016	85-90	Anorexia nervosa	0-4	NA	0-4	NA	0-4	NA
1970-2016	90-95	Anorexia nervosa	0-4	NA	0-4	NA	0-4	NA
1970-1984	90-95	Anorexia nervosa	0-4	NA	0-4	NA	0-4	NA
1985-1994	90-95	Anorexia nervosa	0-4	NA	0-4	NA	0-4	NA
1995-2004	90-95	Anorexia nervosa	0-4	NA	0-4	NA	0-4	NA
2005-2016	90-95	Anorexia nervosa	0-4	NA	0-4	NA	0-4	NA
1970-2016	95-100	Anorexia nervosa	0-4	NA	0-4	NA	0-4	NA
1970-1984	95-100	Anorexia nervosa	0-4	NA	0-4	NA	0-4	NA
1985-1994	95-100	Anorexia nervosa	0-4	NA	0-4	NA	0-4	NA
1995-2004	95-100	Anorexia nervosa	0-4	NA	0-4	NA	0-4	NA
2005-2016	95-100	Anorexia nervosa	0-4	NA	0-4	NA	0-4	NA
1970-2016	1-5	Anorexia nervosa (inpatient)	0-4	NA	0-4	NA	0-4	NA
1970-1984	1-5	Anorexia nervosa (inpatient)	0-4	NA	0-4	NA	0-4	NA
1985-1994	1-5	Anorexia nervosa (inpatient)	0-4	NA	0-4	NA	0-4	NA
1995-2004	1-5	Anorexia nervosa (inpatient)	0-4	NA	0-4	NA	0-4	NA
2005-2016	1-5	Anorexia nervosa (inpatient)	0-4	NA	0-4	NA	0-4	NA
1970-2016	5-10	Anorexia nervosa (inpatient)	9	0.01 (0.01-0.01)	0-4	NA	5-9	0.01 (0.01-0.01)
1970-1984	5-10	Anorexia nervosa (inpatient)	0-4	NA	0-4	NA	0-4	NA
1985-1994	5-10	Anorexia nervosa (inpatient)	0-4	NA	0-4	NA	0-4	NA
1995-2004	5-10	Anorexia nervosa (inpatient)	0-4	NA	0-4	NA	0-4	NA

			Both sexes		Males		Females	
Years	Age	Mental disorder	Cases	IR (95% CI)	Cases	IR (95% CI)	Cases	IR (95% CI)
2005-2016	5-10	Anorexia nervosa (inpatient)	0-4	NA	0-4	NA	0-4	NA
1970-2016	10-15	Anorexia nervosa (inpatient)	813	0.51 (0.51-0.51)	92	0.11 (0.11-0.11)	721	0.93 (0.93-0.93)
1970-1984	10-15	Anorexia nervosa (inpatient)	120	0.21 (0.21-0.21)	21	0.07 (0.07-0.07)	99	0.36 (0.36-0.36)
1985-1994	10-15	Anorexia nervosa (inpatient)	119	0.38 (0.38-0.38)	15	0.09 (0.09-0.09)	104	0.67 (0.67-0.67)
1995-2004	10-15	Anorexia nervosa (inpatient)	183	0.62 (0.62-0.62)	18	0.12 (0.12-0.12)	165	1.14 (1.14-1.15)
2005-2016	10-15	Anorexia nervosa (inpatient)	391	0.97 (0.97-0.98)	38	0.18 (0.18-0.19)	353	1.80 (1.80-1.81)
1970-2016	15-20	Anorexia nervosa (inpatient)	1274	0.79 (0.79-0.79)	65	0.08 (0.08-0.08)	1209	1.55 (1.54-1.55)
1970-1984	15-20	Anorexia nervosa (inpatient)	244	0.43 (0.43-0.43)	19	0.06 (0.06-0.06)	225	0.81 (0.81-0.81)
1985-1994	15-20	Anorexia nervosa (inpatient)	214	0.61 (0.61-0.61)	11	0.06 (0.06-0.06)	203	1.18 (1.18-1.18)
1995-2004	15-20	Anorexia nervosa (inpatient)	289	1.02 (1.01-1.02)	11	0.08 (0.08-0.08)	278	2.00 (2.00-2.01)
2005-2016	15-20	Anorexia nervosa (inpatient)	527	1.32 (1.32-1.32)	24	0.12 (0.12-0.12)	503	2.60 (2.59-2.60)
1970-2016	20-25	Anorexia nervosa (inpatient)	685	0.42 (0.42-0.42)	30	0.04 (0.04-0.04)	655	0.84 (0.84-0.84)
1970-1984	20-25	Anorexia nervosa (inpatient)	159	0.29 (0.29-0.29)	10	0.03 (0.03-0.03)	149	0.56 (0.55-0.56)
1985-1994	20-25	Anorexia nervosa (inpatient)	135	0.36 (0.36-0.36)	5	0.03 (0.03-0.03)	130	0.73 (0.73-0.73)
1995-2004	20-25	Anorexia nervosa (inpatient)	133	0.43 (0.43-0.43)	0-4	NA	129-133	0.86 (0.86-0.86)
2005-2016	20-25	Anorexia nervosa (inpatient)	258	0.69 (0.69-0.69)	11	0.06 (0.06-0.06)	247	1.35 (1.35-1.36)
1970-2016	25-30	Anorexia nervosa (inpatient)	313	0.19 (0.19-0.19)	13	0.02 (0.02-0.02)	300	0.38 (0.38-0.38)
1970-1984	25-30	Anorexia nervosa (inpatient)	77	0.14 (0.14-0.14)	0-4	NA	73-77	0.27 (0.27-0.27)
1985-1994	25-30	Anorexia nervosa (inpatient)	59	0.16 (0.16-0.16)	5	0.03 (0.03-0.03)	54	0.30 (0.30-0.30)
1995-2004	25-30	Anorexia nervosa (inpatient)	58	0.17 (0.17-0.17)	0-4	NA	54-58	0.36 (0.36-0.36)
2005-2016	25-30	Anorexia nervosa (inpatient)	119	0.34 (0.34-0.34)	6	0.03 (0.03-0.03)	113	0.67 (0.67-0.67)
1970-2016	30-35	Anorexia nervosa (inpatient)	160	0.10 (0.10-0.10)	5	0.01 (0.01-0.01)	155	0.20 (0.20-0.20)
1970-1984	30-35	Anorexia nervosa (inpatient)	46	0.08 (0.08-0.08)	0-4	NA	42-46	0.17 (0.17-0.17)
1985-1994	30-35	Anorexia nervosa (inpatient)	28	0.08 (0.08-0.08)	0-4	NA	24-28	0.15 (0.15-0.15)
1995-2004	30-35	Anorexia nervosa (inpatient)	37	0.10 (0.10-0.10)	0-4	NA	33-37	0.21 (0.21-0.21)
2005-2016	30-35	Anorexia nervosa (inpatient)	49	0.14 (0.14-0.14)	0-4	NA	45-49	0.27 (0.27-0.28)
1970-2016	35-40	Anorexia nervosa (inpatient)	103	0.06 (0.06-0.06)	8	0.01 (0.01-0.01)	95	0.12 (0.12-0.12)
1970-1984	35-40	Anorexia nervosa (inpatient)	28	0.06 (0.06-0.06)	0-4	NA	24-28	0.11 (0.11-0.11)
1985-1994	35-40	Anorexia nervosa (inpatient)	18	0.05 (0.05-0.05)	0-4	NA	14-18	0.09 (0.09-0.09)
1995-2004	35-40	Anorexia nervosa (inpatient)	17	0.05 (0.05-0.05)	0-4	NA	13-17	0.10 (0.10-0.10)
2005-2016	35-40	Anorexia nervosa (inpatient)	40	0.10 (0.10-0.10)	0-4	NA	36-40	0.19 (0.19-0.19)
1970-2016	40-45	Anorexia nervosa (inpatient)	53	0.03 (0.03-0.03)	0-4	NA	49-53	0.07 (0.07-0.07)
1970-1984	40-45	Anorexia nervosa (inpatient)	6	0.01 (0.01-0.01)	0-4	NA	2-6	0.03 (0.03-0.03)
1985-1994	40-45	Anorexia nervosa (inpatient)	16	0.04 (0.04-0.04)	0-4	NA	12-16	0.08 (0.08-0.08)
1995-2004	40-45	Anorexia nervosa (inpatient)	14	0.04 (0.04-0.04)	0-4	NA	10-14	0.08 (0.08-0.08)
2005-2016	40-45	Anorexia nervosa (inpatient)	17	0.04 (0.04-0.04)	0-4	NA	13-17	0.08 (0.08-0.08)
1970-2016	45-50	Anorexia nervosa (inpatient)	44	0.03 (0.03-0.03)	0-4	NA	40-44	0.05 (0.05-0.05)
1970-1984	45-50	Anorexia nervosa (inpatient)	11	0.03 (0.03-0.03)	0-4	NA	7-11	0.05 (0.05-0.05)
1985-1994	45-50	Anorexia nervosa (inpatient)	13	0.04 (0.04-0.04)	0-4	NA	9-13	0.08 (0.08-0.08)
1995-2004	45-50	Anorexia nervosa (inpatient)	7	0.02 (0.02-0.02)	0-4	NA	3-7	0.03 (0.03-0.03)

			Both sexes		Males		Females	
Years	Age	Mental disorder	Cases	IR (95% CI)	Cases	IR (95% CI)	Cases	IR (95% CI)
2005-2016	45-50	Anorexia nervosa (inpatient)	13	0.03 (0.03-0.03)	0-4	NA	9-13	0.06 (0.06-0.06)
1970-2016	50-55	Anorexia nervosa (inpatient)	34	0.02 (0.02-0.02)	0-4	NA	30-34	0.05 (0.05-0.05)
1970-1984	50-55	Anorexia nervosa (inpatient)	6	0.01 (0.01-0.01)	0-4	NA	2-6	0.03 (0.03-0.03)
1985-1994	50-55	Anorexia nervosa (inpatient)	10	0.04 (0.04-0.04)	0-4	NA	6-10	0.07 (0.07-0.07)
1995-2004	50-55	Anorexia nervosa (inpatient)	6	0.02 (0.02-0.02)	0-4	NA	2-6	0.03 (0.03-0.03)
2005-2016	50-55	Anorexia nervosa (inpatient)	12	0.03 (0.03-0.03)	0-4	NA	8-12	0.06 (0.06-0.06)
1970-2016	55-60	Anorexia nervosa (inpatient)	16	0.01 (0.01-0.01)	0-4	NA	12-16	0.02 (0.02-0.02)
1970-1984	55-60	Anorexia nervosa (inpatient)	0-4	NA	0-4	NA	0-4	NA
1985-1994	55-60	Anorexia nervosa (inpatient)	0-4	NA	0-4	NA	0-4	NA
1995-2004	55-60	Anorexia nervosa (inpatient)	0-4	NA	0-4	NA	0-4	NA
2005-2016	55-60	Anorexia nervosa (inpatient)	7	0.02 (0.02-0.02)	0-4	NA	3-7	0.04 (0.04-0.04)
1970-2016	60-65	Anorexia nervosa (inpatient)	5	0.00 (0.00-0.00)	0-4	NA	1-5	0.01 (0.01-0.01)
1970-1984	60-65	Anorexia nervosa (inpatient)	0-4	NA	0-4	NA	0-4	NA
1985-1994	60-65	Anorexia nervosa (inpatient)	0-4	NA	0-4	NA	0-4	NA
1995-2004	60-65	Anorexia nervosa (inpatient)	0-4	NA	0-4	NA	0-4	NA
2005-2016	60-65	Anorexia nervosa (inpatient)	0-4	NA	0-4	NA	0-4	NA
1970-2016	65-70	Anorexia nervosa (inpatient)	5	0.00 (0.00-0.00)	0-4	NA	1-5	0.01 (0.01-0.01)
1970-1984	65-70	Anorexia nervosa (inpatient)	0-4	NA	0-4	NA	0-4	NA
1985-1994	65-70	Anorexia nervosa (inpatient)	0-4	NA	0-4	NA	0-4	NA
1995-2004	65-70	Anorexia nervosa (inpatient)	0-4	NA	0-4	NA	0-4	NA
2005-2016	65-70	Anorexia nervosa (inpatient)	0-4	NA	0-4	NA	0-4	NA
1970-2016	70-75	Anorexia nervosa (inpatient)	0-4	NA	0-4	NA	0-4	NA
1970-1984	70-75	Anorexia nervosa (inpatient)	0-4	NA	0-4	NA	0-4	NA
1985-1994	70-75	Anorexia nervosa (inpatient)	0-4	NA	0-4	NA	0-4	NA
1995-2004	70-75	Anorexia nervosa (inpatient)	0-4	NA	0-4	NA	0-4	NA
2005-2016	70-75	Anorexia nervosa (inpatient)	0-4	NA	0-4	NA	0-4	NA
1970-2016	75-80	Anorexia nervosa (inpatient)	0-4	NA	0-4	NA	0-4	NA
1970-1984	75-80	Anorexia nervosa (inpatient)	0-4	NA	0-4	NA	0-4	NA
1985-1994	75-80	Anorexia nervosa (inpatient)	0-4	NA	0-4	NA	0-4	NA
1995-2004	75-80	Anorexia nervosa (inpatient)	0-4	NA	0-4	NA	0-4	NA
2005-2016	75-80	Anorexia nervosa (inpatient)	0-4	NA	0-4	NA	0-4	NA
1970-2016	80-85	Anorexia nervosa (inpatient)	0-4	NA	0-4	NA	0-4	NA
1970-1984	80-85	Anorexia nervosa (inpatient)	0-4	NA	0-4	NA	0-4	NA
1985-1994	80-85	Anorexia nervosa (inpatient)	0-4	NA	0-4	NA	0-4	NA
1995-2004	80-85	Anorexia nervosa (inpatient)	0-4	NA	0-4	NA	0-4	NA
2005-2016	80-85	Anorexia nervosa (inpatient)	0-4	NA	0-4	NA	0-4	NA
1970-2016	85-90	Anorexia nervosa (inpatient)	0-4	NA	0-4	NA	0-4	NA
1970-1984	85-90	Anorexia nervosa (inpatient)	0-4	NA	0-4	NA	0-4	NA
1985-1994	85-90	Anorexia nervosa (inpatient)	0-4	NA	0-4	NA	0-4	NA
1995-2004	85-90	Anorexia nervosa (inpatient)	0-4	NA	0-4	NA	0-4	NA

			Both sexes		Males		Females	
**Years**	**Age**	**Mental disorder**	**Cases**	**IR (95% CI)**	**Cases**	**IR (95% CI)**	**Cases**	**IR (95% CI)**
2005-2016	85-90	Anorexia nervosa (inpatient)	0-4	NA	0-4	NA	0-4	NA
1970-2016	90-95	Anorexia nervosa (inpatient)	0-4	NA	0-4	NA	0-4	NA
1970-1984	90-95	Anorexia nervosa (inpatient)	0-4	NA	0-4	NA	0-4	NA
1985-1994	90-95	Anorexia nervosa (inpatient)	0-4	NA	0-4	NA	0-4	NA
1995-2004	90-95	Anorexia nervosa (inpatient)	0-4	NA	0-4	NA	0-4	NA
2005-2016	90-95	Anorexia nervosa (inpatient)	0-4	NA	0-4	NA	0-4	NA
1970-2016	95-100	Anorexia nervosa (inpatient)	0-4	NA	0-4	NA	0-4	NA
1970-1984	95-100	Anorexia nervosa (inpatient)	0-4	NA	0-4	NA	0-4	NA
1985-1994	95-100	Anorexia nervosa (inpatient)	0-4	NA	0-4	NA	0-4	NA
1995-2004	95-100	Anorexia nervosa (inpatient)	0-4	NA	0-4	NA	0-4	NA
2005-2016	95-100	Anorexia nervosa (inpatient)	0-4	NA	0-4	NA	0-4	NA
1970-2016	10-15	Personality disorders	1,477	0.93 (0.93-0.93)	460	0.57 (0.57-0.57)	1,017	1.32 (1.32-1.32)
1970-1984	10-15	Personality disorders	299	0.52 (0.52-0.53)	164	0.56 (0.56-0.56)	135	0.49 (0.48-0.49)
1985-1994	10-15	Personality disorders	107	0.34 (0.34-0.34)	54	0.34 (0.33-0.34)	53	0.34 (0.34-0.34)
1995-2004	10-15	Personality disorders	504	1.70 (1.70-1.71)	147	0.97 (0.97-0.97)	357	2.48 (2.47-2.48)
2005-2016	10-15	Personality disorders	567	1.41 (1.41-1.41)	95	0.46 (0.46-0.46)	472	2.41 (2.41-2.42)
1970-2016	15-20	Personality disorders	16,651	10.38 (10.38-10.39)	5,480	6.65 (6.65-6.66)	11,171	14.32 (14.31-14.33)
1970-1984	15-20	Personality disorders	3,981	6.98 (6.98-6.99)	2,053	7.01 (7.00-7.02)	1,928	6.95 (6.94-6.96)
1985-1994	15-20	Personality disorders	1,071	3.03 (3.03-3.04)	496	2.74 (2.74-2.74)	575	3.35 (3.34-3.35)
1995-2004	15-20	Personality disorders	4,188	14.76 (14.74-14.78)	1,267	8.71 (8.70-8.73)	2,921	21.12 (21.08-21.16)
2005-2016	15-20	Personality disorders	7,411	18.66 (18.64-18.68)	1,664	8.14 (8.13-8.15)	5,747	29.83 (29.79-29.87)
1970-2016	20-25	Personality disorders	25,974	16.22 (16.21-16.23)	10,087	12.16 (12.15-12.17)	15,887	20.59 (20.57-20.60)
1970-1984	20-25	Personality disorders	7,630	13.83 (13.82-13.84)	4,090	14.36 (14.34-14.38)	3,540	13.27 (13.25-13.28)
1985-1994	20-25	Personality disorders	2,333	6.27 (6.26-6.28)	1,111	5.74 (5.73-5.74)	1,222	6.85 (6.84-6.86)
1995-2004	20-25	Personality disorders	5,917	19.24 (19.22-19.26)	2,104	13.20 (13.18-13.22)	3,813	25.74 (25.70-25.78)
2005-2016	20-25	Personality disorders	10,094	27.29 (27.26-27.32)	2,782	14.53 (14.51-14.55)	7,312	41.00 (40.94-41.06)
1970-2016	25-30	Personality disorders	21,874	13.66 (13.66-13.67)	9,472	11.46 (11.45-11.46)	12,402	16.02 (16.01-16.03)
1970-1984	25-30	Personality disorders	8,203	14.61 (14.60-14.62)	4,096	14.19 (14.18-14.21)	4,107	15.05 (15.03-15.07)
1985-1994	25-30	Personality disorders	2,601	7.10 (7.10-7.11)	1,143	6.02 (6.01-6.03)	1,458	8.27 (8.26-8.28)
1995-2004	25-30	Personality disorders	4,939	14.80 (14.78-14.82)	2,092	12.09 (12.07-12.11)	2,847	17.72 (17.69-17.74)
2005-2016	25-30	Personality disorders	6,131	18.05 (18.03-18.07)	2,141	12.21 (12.19-12.23)	3,990	24.28 (24.24-24.32)
1970-2016	30-35	Personality disorders	19,829	12.52 (12.51-12.53)	8,780	10.80 (10.79-10.81)	11,049	14.34 (14.33-14.35)
1970-1984	30-35	Personality disorders	8,375	15.60 (15.58-15.61)	4,023	14.70 (14.68-14.71)	4,352	16.53 (16.52-16.55)
1985-1994	30-35	Personality disorders	2,646	7.75 (7.74-7.76)	1,132	6.44 (6.43-6.45)	1,514	9.14 (9.13-9.15)
1995-2004	30-35	Personality disorders	4,316	12.10 (12.09-12.12)	1,959	10.64 (10.62-10.65)	2,357	13.67 (13.65-13.69)
2005-2016	30-35	Personality disorders	4,492	12.88 (12.87-12.90)	1,666	9.29 (9.28-9.31)	2,826	16.68 (16.66-16.71)
1970-2016	35-40	Personality disorders	17,960	11.48 (11.47-11.48)	7,634	9.56 (9.56-9.57)	10,326	13.46 (13.46-13.47)
1970-1984	35-40	Personality disorders	8,086	16.55 (16.54-16.57)	3,627	14.67 (14.65-14.68)	4,459	18.49 (18.47-18.51)
1985-1994	35-40	Personality disorders	2,585	7.54 (7.53-7.54)	1,075	6.15 (6.15-6.16)	1,510	8.97 (8.95-8.98)
1995-2004	35-40	Personality disorders	3,723	10.50 (10.49-10.51)	1,586	8.72 (8.71-8.73)	2,137	12.37 (12.36-12.39)

			Both sexes		Males		Females	
**Years**	**Age**	**Mental disorder**	**Cases**	**IR (95% CI)**	**Cases**	**IR (95% CI)**	**Cases**	**IR (95% CI)**
2005-2016	35-40	Personality disorders	3,566	9.41 (9.40-9.42)	1,346	6.93 (6.92-6.94)	2,220	12.02 (12.01-12.04)
1970-2016	40-45	Personality disorders	15,022	9.82 (9.81-9.82)	6,308	8.14 (8.13-8.14)	8,714	11.54 (11.53-11.55)
1970-1984	40-45	Personality disorders	6,955	16.23 (16.22-16.25)	3,067	14.29 (14.27-14.31)	3,888	18.18 (18.16-18.21)
1985-1994	40-45	Personality disorders	2,482	6.88 (6.87-6.89)	918	5.03 (5.02-5.04)	1,564	8.77 (8.76-8.78)
1995-2004	40-45	Personality disorders	2,710	8.16 (8.15-8.17)	1,144	6.76 (6.75-6.77)	1,566	9.62 (9.60-9.63)
2005-2016	40-45	Personality disorders	2,875	7.03 (7.02-7.04)	1,179	5.64 (5.64-5.65)	1,696	8.48 (8.47-8.49)
1970-2016	45-50	Personality disorders	12,572	8.45 (8.45-8.46)	5,165	6.91 (6.90-6.91)	7,407	10.01 (10.01-10.02)
1970-1984	45-50	Personality disorders	6,247	15.05 (15.03-15.06)	2,724	13.24 (13.22-13.25)	3,523	16.83 (16.81-16.85)
1985-1994	45-50	Personality disorders	2,092	6.33 (6.32-6.33)	745	4.47 (4.47-4.48)	1,347	8.21 (8.20-8.22)
1995-2004	45-50	Personality disorders	2,133	6.43 (6.42-6.44)	854	5.10 (5.09-5.11)	1,279	7.79 (7.77-7.80)
2005-2016	45-50	Personality disorders	2,100	5.12 (5.12-5.13)	842	4.05 (4.04-4.05)	1,258	6.23 (6.22-6.24)
1970-2016	50-55	Personality disorders	9,351	6.56 (6.56-6.57)	3,538	4.98 (4.98-4.98)	5,813	8.14 (8.13-8.15)
1970-1984	50-55	Personality disorders	5,207	12.45 (12.43-12.46)	2,087	10.14 (10.12-10.15)	3,120	14.68 (14.66-14.70)
1985-1994	50-55	Personality disorders	1,370	5.01 (5.00-5.01)	403	2.96 (2.95-2.96)	967	7.04 (7.03-7.05)
1995-2004	50-55	Personality disorders	1,451	4.21 (4.21-4.21)	540	3.13 (3.12-3.13)	911	5.30 (5.29-5.31)
2005-2016	50-55	Personality disorders	1,323	3.41 (3.41-3.41)	508	2.60 (2.59-2.60)	815	4.24 (4.23-4.24)
1970-2016	55-60	Personality disorders	6,273	4.65 (4.65-4.65)	2,238	3.36 (3.36-3.36)	4,035	5.91 (5.90-5.91)
1970-1984	55-60	Personality disorders	3,638	8.78 (8.77-8.79)	1,342	6.64 (6.63-6.65)	2,296	10.81 (10.79-10.82)
1985-1994	55-60	Personality disorders	965	3.88 (3.88-3.89)	266	2.19 (2.18-2.19)	699	5.52 (5.51-5.53)
1995-2004	55-60	Personality disorders	882	2.84 (2.84-2.84)	312	2.02 (2.01-2.02)	570	3.65 (3.65-3.66)
2005-2016	55-60	Personality disorders	788	2.10 (2.10-2.10)	318	1.70 (1.70-1.70)	470	2.50 (2.50-2.50)
1970-2016	60-65	Personality disorders	3,891	3.08 (3.08-3.08)	1,178	1.92 (1.92-1.92)	2,713	4.17 (4.17-4.17)
1970-1984	60-65	Personality disorders	2,130	5.38 (5.38-5.39)	648	3.42 (3.41-3.42)	1,482	7.19 (7.18-7.20)
1985-1994	60-65	Personality disorders	782	3.23 (3.23-3.24)	177	1.53 (1.53-1.53)	605	4.80 (4.79-4.81)
1995-2004	60-65	Personality disorders	546	2.19 (2.19-2.19)	182	1.50 (1.49-1.50)	364	2.85 (2.85-2.86)
2005-2016	60-65	Personality disorders	433	1.15 (1.15-1.15)	171	0.92 (0.92-0.92)	262	1.37 (1.37-1.37)
1970-2016	65-70	Personality disorders	2,574	2.27 (2.27-2.27)	668	1.25 (1.25-1.25)	1,906	3.18 (3.18-3.18)
1970-1984	65-70	Personality disorders	1,252	3.56 (3.56-3.57)	314	1.93 (1.93-1.93)	938	4.97 (4.96-4.98)
1985-1994	65-70	Personality disorders	615	2.65 (2.65-2.65)	139	1.29 (1.29-1.29)	476	3.82 (3.81-3.83)
1995-2004	65-70	Personality disorders	430	2.01 (2.01-2.01)	114	1.13 (1.12-1.13)	316	2.80 (2.80-2.81)
2005-2016	65-70	Personality disorders	277	0.82 (0.82-0.82)	101	0.62 (0.62-0.62)	176	1.01 (1.01-1.01)
1970-2016	70-75	Personality disorders	1,669	1.77 (1.77-1.77)	342	0.81 (0.81-0.81)	1,327	2.56 (2.56-2.56)
1970-1984	70-75	Personality disorders	711	2.48 (2.47-2.48)	132	1.05 (1.05-1.05)	579	3.58 (3.58-3.59)
1985-1994	70-75	Personality disorders	414	2.04 (2.04-2.04)	73	0.82 (0.81-0.82)	341	3.00 (3.00-3.01)
1995-2004	70-75	Personality disorders	359	1.89 (1.88-1.89)	84	0.98 (0.98-0.98)	275	2.63 (2.62-2.63)
2005-2016	70-75	Personality disorders	185	0.71 (0.71-0.71)	53	0.43 (0.43-0.43)	132	0.96 (0.95-0.96)
1970-2016	75-80	Personality disorders	1,002	1.39 (1.39-1.39)	219	0.73 (0.73-0.73)	783	1.87 (1.87-1.87)
1970-1984	75-80	Personality disorders	287	1.41 (1.40-1.41)	57	0.69 (0.69-0.69)	230	1.90 (1.89-1.90)
1985-1994	75-80	Personality disorders	274	1.69 (1.69-1.69)	52	0.79 (0.79-0.79)	222	2.30 (2.30-2.31)
1995-2004	75-80	Personality disorders	296	1.85 (1.85-1.86)	64	0.96 (0.96-0.97)	232	2.48 (2.48-2.49)

			Both sexes		Males		Females	
**Years**	**Age**	**Mental disorder**	**Cases**	**IR (95% CI)**	**Cases**	**IR (95% CI)**	**Cases**	**IR (95% CI)**
2005-2016	75-80	Personality disorders	145	0.75 (0.75-0.75)	46	0.53 (0.53-0.53)	99	0.92 (0.92-0.92)
1970-2016	80-85	Personality disorders	486	1.01 (1.01-1.01)	88	0.48 (0.48-0.48)	398	1.33 (1.32-1.33)
1970-1984	80-85	Personality disorders	75	0.62 (0.61-0.62)	10	0.22 (0.22-0.22)	65	0.86 (0.86-0.86)
1985-1994	80-85	Personality disorders	99	0.91 (0.91-0.91)	14	0.36 (0.36-0.36)	85	1.21 (1.21-1.22)
1995-2004	80-85	Personality disorders	211	1.86 (1.86-1.86)	43	1.03 (1.03-1.03)	168	2.34 (2.34-2.35)
2005-2016	80-85	Personality disorders	101	0.73 (0.73-0.73)	21	0.37 (0.37-0.38)	80	0.97 (0.96-0.97)
1970-2016	85-90	Personality disorders	219	0.84 (0.84-0.85)	44	0.51 (0.51-0.51)	175	1.01 (1.01-1.01)
1970-1984	85-90	Personality disorders	30	0.54 (0.54-0.54)	6	0.30 (0.30-0.30)	24	0.68 (0.68-0.68)
1985-1994	85-90	Personality disorders	28	0.51 (0.51-0.51)	5	0.30 (0.30-0.30)	23	0.61 (0.61-0.61)
1995-2004	85-90	Personality disorders	96	1.49 (1.48-1.49)	22	1.10 (1.09-1.10)	74	1.66 (1.66-1.67)
2005-2016	85-90	Personality disorders	65	0.77 (0.76-0.77)	11	0.38 (0.38-0.38)	54	0.97 (0.97-0.97)
1970-2016	90-95	Personality disorders	60	0.63 (0.63-0.63)	12	0.45 (0.45-0.46)	48	0.70 (0.70-0.70)
1970-1984	90-95	Personality disorders	0-4	NA	0-4	NA	0-4	NA
1985-1994	90-95	Personality disorders	0-4	NA	0-4	NA	0-4	NA
1995-2004	90-95	Personality disorders	30	1.22 (1.21-1.22)	7	1.14 (1.14-1.15)	23	1.24 (1.24-1.25)
2005-2016	90-95	Personality disorders	24	0.67 (0.67-0.67)	0-4	NA	20-24	0.80 (0.80-0.81)
1970-2016	95-100	Personality disorders	5	0.25 (0.25-0.25)	0-4	NA	0-4	NA
1970-1984	95-100	Personality disorders	0-4	NA	0-4	NA	0-4	NA
1985-1994	95-100	Personality disorders	0-4	NA	0-4	NA	0-4	NA
1995-2004	95-100	Personality disorders	0-4	NA	0-4	NA	0-4	NA
2005-2016	95-100	Personality disorders	5	0.57 (0.57-0.57)	0-4	NA	0-4	NA
1970-2016	10-15	Personality disorders (inpatient)	664	0.42 (0.42-0.42)	264	0.33 (0.33-0.33)	400	0.52 (0.52-0.52)
1970-1984	10-15	Personality disorders (inpatient)	299	0.52 (0.52-0.53)	164	0.56 (0.56-0.56)	135	0.49 (0.48-0.49)
1985-1994	10-15	Personality disorders (inpatient)	84	0.27 (0.27-0.27)	41	0.25 (0.25-0.25)	43	0.28 (0.28-0.28)
1995-2004	10-15	Personality disorders (inpatient)	170	0.57 (0.57-0.58)	48	0.32 (0.32-0.32)	122	0.85 (0.85-0.85)
2005-2016	10-15	Personality disorders (inpatient)	111	0.28 (0.28-0.28)	11	0.05 (0.05-0.05)	100	0.51 (0.51-0.51)
1970-2016	15-20	Personality disorders (inpatient)	8,428	5.25 (5.25-5.25)	3,309	4.01 (4.01-4.02)	5,119	6.55 (6.54-6.55)
1970-1984	15-20	Personality disorders (inpatient)	3,981	6.98 (6.98-6.99)	2,053	7.01 (7.00-7.02)	1,928	6.95 (6.94-6.96)
1985-1994	15-20	Personality disorders (inpatient)	995	2.82 (2.82-2.82)	472	2.61 (2.60-2.61)	523	3.04 (3.04-3.05)
1995-2004	15-20	Personality disorders (inpatient)	1,471	5.17 (5.17-5.18)	390	2.68 (2.67-2.68)	1,081	7.79 (7.78-7.81)
2005-2016	15-20	Personality disorders (inpatient)	1,981	4.97 (4.97-4.98)	394	1.92 (1.92-1.93)	1,587	8.19 (8.18-8.21)
1970-2016	20-25	Personality disorders (inpatient)	15,152	9.43 (9.43-9.44)	6,784	8.16 (8.16-8.17)	8,368	10.79 (10.78-10.80)
1970-1984	20-25	Personality disorders (inpatient)	7,630	13.83 (13.82-13.84)	4,090	14.36 (14.34-14.38)	3,540	13.27 (13.25-13.28)
1985-1994	20-25	Personality disorders (inpatient)	2,185	5.87 (5.87-5.88)	1,072	5.54 (5.53-5.54)	1,113	6.24 (6.23-6.25)
1995-2004	20-25	Personality disorders (inpatient)	2,398	7.76 (7.75-7.77)	869	5.43 (5.43-5.44)	1,529	10.26 (10.24-10.27)
2005-2016	20-25	Personality disorders (inpatient)	2,939	7.86 (7.85-7.87)	753	3.91 (3.90-3.91)	2,186	12.06 (12.04-12.07)
1970-2016	25-30	Personality disorders (inpatient)	14,594	9.08 (9.07-9.08)	6,759	8.15 (8.15-8.16)	7,835	10.06 (10.05-10.07)
1970-1984	25-30	Personality disorders (inpatient)	8,203	14.61 (14.60-14.62)	4,096	14.19 (14.18-14.21)	4,107	15.05 (15.03-15.07)
1985-1994	25-30	Personality disorders (inpatient)	2,457	6.71 (6.70-6.72)	1,106	5.82 (5.82-5.83)	1,351	7.66 (7.65-7.67)
1995-2004	25-30	Personality disorders (inpatient)	2,091	6.24 (6.23-6.24)	895	5.15 (5.15-5.16)	1,196	7.40 (7.38-7.41)

			Both sexes		Males		Females	
Years	Age	Mental disorder	Cases	IR (95% CI)	Cases	IR (95% CI)	Cases	IR (95% CI)
2005-2016	25-30	Personality disorders (inpatient)	1,843	5.34 (5.34-5.35)	662	3.74 (3.73-3.75)	1,181	7.03 (7.02-7.04)
1970-2016	30-35	Personality disorders (inpatient)	14,286	8.98 (8.98-8.99)	6,578	8.07 (8.06-8.07)	7,708	9.95 (9.94-9.95)
1970-1984	30-35	Personality disorders (inpatient)	8,375	15.60 (15.58-15.61)	4,023	14.70 (14.68-14.71)	4,352	16.53 (16.52-16.55)
1985-1994	30-35	Personality disorders (inpatient)	2,511	7.35 (7.34-7.36)	1,090	6.20 (6.19-6.21)	1,421	8.58 (8.56-8.59)
1995-2004	30-35	Personality disorders (inpatient)	1,937	5.41 (5.40-5.42)	882	4.77 (4.77-4.78)	1,055	6.09 (6.08-6.10)
2005-2016	30-35	Personality disorders (inpatient)	1,463	4.13 (4.13-4.14)	583	3.22 (3.21-3.22)	880	5.09 (5.09-5.10)
1970-2016	35-40	Personality disorders (inpatient)	13,467	8.57 (8.57-8.58)	5,929	7.41 (7.40-7.41)	7,538	9.78 (9.78-9.79)
1970-1984	35-40	Personality disorders (inpatient)	8,086	16.55 (16.54-16.57)	3,627	14.67 (14.65-14.68)	4,459	18.49 (18.47-18.51)
1985-1994	35-40	Personality disorders (inpatient)	2,468	7.19 (7.19-7.20)	1,043	5.97 (5.96-5.98)	1,425	8.46 (8.45-8.47)
1995-2004	35-40	Personality disorders (inpatient)	1,673	4.70 (4.70-4.71)	725	3.97 (3.97-3.98)	948	5.47 (5.46-5.47)
2005-2016	35-40	Personality disorders (inpatient)	1,240	3.23 (3.23-3.23)	534	2.72 (2.72-2.73)	706	3.76 (3.76-3.77)
1970-2016	40-45	Personality disorders (inpatient)	11,529	7.51 (7.51-7.51)	4,938	6.35 (6.35-6.36)	6,591	8.69 (8.69-8.70)
1970-1984	40-45	Personality disorders (inpatient)	6,955	16.23 (16.22-16.25)	3,067	14.29 (14.27-14.31)	3,888	18.18 (18.16-18.21)
1985-1994	40-45	Personality disorders (inpatient)	2,385	6.61 (6.60-6.62)	887	4.86 (4.85-4.87)	1,498	8.40 (8.39-8.41)
1995-2004	40-45	Personality disorders (inpatient)	1,209	3.63 (3.63-3.63)	523	3.08 (3.08-3.09)	686	4.20 (4.19-4.20)
2005-2016	40-45	Personality disorders (inpatient)	980	2.37 (2.37-2.38)	461	2.19 (2.19-2.19)	519	2.56 (2.56-2.57)
1970-2016	45-50	Personality disorders (inpatient)	10,000	6.70 (6.70-6.71)	4,199	5.60 (5.60-5.61)	5,801	7.82 (7.81-7.82)
1970-1984	45-50	Personality disorders (inpatient)	6,247	15.05 (15.03-15.06)	2,724	13.24 (13.22-13.25)	3,523	16.83 (16.81-16.85)
1985-1994	45-50	Personality disorders (inpatient)	2,017	6.10 (6.09-6.11)	730	4.38 (4.38-4.39)	1,287	7.84 (7.83-7.85)
1995-2004	45-50	Personality disorders (inpatient)	964	2.90 (2.90-2.90)	391	2.33 (2.33-2.33)	573	3.48 (3.47-3.48)
2005-2016	45-50	Personality disorders (inpatient)	772	1.87 (1.87-1.87)	354	1.69 (1.69-1.69)	418	2.05 (2.05-2.05)
1970-2016	50-55	Personality disorders (inpatient)	7,683	5.38 (5.38-5.38)	2,932	4.12 (4.12-4.12)	4,751	6.63 (6.63-6.64)
1970-1984	50-55	Personality disorders (inpatient)	5,207	12.45 (12.43-12.46)	2,087	10.14 (10.12-10.15)	3,120	14.68 (14.66-14.70)
1985-1994	50-55	Personality disorders (inpatient)	1,316	4.81 (4.80-4.81)	384	2.82 (2.81-2.82)	932	6.78 (6.77-6.79)
1995-2004	50-55	Personality disorders (inpatient)	668	1.93 (1.93-1.94)	256	1.48 (1.48-1.48)	412	2.39 (2.39-2.39)
2005-2016	50-55	Personality disorders (inpatient)	492	1.26 (1.26-1.26)	205	1.04 (1.04-1.04)	287	1.48 (1.48-1.48)
1970-2016	55-60	Personality disorders (inpatient)	5,294	3.92 (3.92-3.92)	1,891	2.84 (2.84-2.84)	3,403	4.97 (4.97-4.97)
1970-1984	55-60	Personality disorders (inpatient)	3,638	8.78 (8.77-8.79)	1,342	6.64 (6.63-6.65)	2,296	10.81 (10.79-10.82)
1985-1994	55-60	Personality disorders (inpatient)	941	3.79 (3.78-3.79)	262	2.15 (2.15-2.16)	679	5.36 (5.35-5.37)
1995-2004	55-60	Personality disorders (inpatient)	404	1.30 (1.30-1.30)	144	0.93 (0.93-0.93)	260	1.66 (1.66-1.67)
2005-2016	55-60	Personality disorders (inpatient)	311	0.82 (0.82-0.83)	143	0.76 (0.76-0.76)	168	0.89 (0.89-0.89)
1970-2016	60-65	Personality disorders (inpatient)	3,343	2.64 (2.64-2.64)	986	1.61 (1.61-1.61)	2,357	3.62 (3.62-3.62)
1970-1984	60-65	Personality disorders (inpatient)	2,130	5.38 (5.38-5.39)	648	3.42 (3.41-3.42)	1,482	7.19 (7.18-7.20)
1985-1994	60-65	Personality disorders (inpatient)	761	3.15 (3.14-3.15)	174	1.50 (1.50-1.51)	587	4.66 (4.65-4.67)
1995-2004	60-65	Personality disorders (inpatient)	254	1.02 (1.02-1.02)	83	0.68 (0.68-0.68)	171	1.34 (1.34-1.34)
2005-2016	60-65	Personality disorders (inpatient)	198	0.52 (0.52-0.52)	81	0.43 (0.43-0.44)	117	0.61 (0.61-0.61)
1970-2016	65-70	Personality disorders (inpatient)	2,193	1.93 (1.93-1.93)	547	1.02 (1.02-1.02)	1,646	2.74 (2.74-2.74)
1970-1984	65-70	Personality disorders (inpatient)	1,252	3.56 (3.56-3.57)	314	1.93 (1.93-1.93)	938	4.97 (4.96-4.98)
1985-1994	65-70	Personality disorders (inpatient)	593	2.55 (2.55-2.56)	132	1.23 (1.22-1.23)	461	3.70 (3.69-3.71)
1995-2004	65-70	Personality disorders (inpatient)	216	1.01 (1.01-1.01)	52	0.51 (0.51-0.51)	164	1.45 (1.45-1.46)

			Both sexes		Males		Females	
**Years**	**Age**	**Mental disorder**	**Cases**	**IR (95% CI)**	**Cases**	**IR (95% CI)**	**Cases**	**IR (95% CI)**
2005-2016	65-70	Personality disorders (inpatient)	132	0.39 (0.39-0.39)	49	0.30 (0.30-0.30)	83	0.48 (0.48-0.48)
1970-2016	70-75	Personality disorders (inpatient)	1,318	1.40 (1.40-1.40)	256	0.60 (0.60-0.60)	1,062	2.05 (2.05-2.05)
1970-1984	70-75	Personality disorders (inpatient)	711	2.48 (2.47-2.48)	132	1.05 (1.05-1.05)	579	3.58 (3.58-3.59)
1985-1994	70-75	Personality disorders (inpatient)	402	1.98 (1.98-1.98)	71	0.79 (0.79-0.79)	331	2.92 (2.91-2.92)
1995-2004	70-75	Personality disorders (inpatient)	121	0.64 (0.63-0.64)	26	0.30 (0.30-0.30)	95	0.91 (0.91-0.91)
2005-2016	70-75	Personality disorders (inpatient)	84	0.32 (0.32-0.32)	27	0.22 (0.22-0.22)	57	0.41 (0.41-0.41)
1970-2016	75-80	Personality disorders (inpatient)	701	0.97 (0.97-0.97)	157	0.52 (0.52-0.52)	544	1.30 (1.30-1.30)
1970-1984	75-80	Personality disorders (inpatient)	287	1.41 (1.40-1.41)	57	0.69 (0.69-0.69)	230	1.90 (1.89-1.90)
1985-1994	75-80	Personality disorders (inpatient)	267	1.65 (1.64-1.65)	51	0.78 (0.77-0.78)	216	2.24 (2.23-2.24)
1995-2004	75-80	Personality disorders (inpatient)	99	0.62 (0.62-0.62)	29	0.44 (0.44-0.44)	70	0.75 (0.75-0.75)
2005-2016	75-80	Personality disorders (inpatient)	48	0.25 (0.25-0.25)	20	0.23 (0.23-0.23)	28	0.26 (0.26-0.26)
1970-2016	80-85	Personality disorders (inpatient)	272	0.56 (0.56-0.56)	47	0.26 (0.26-0.26)	225	0.75 (0.75-0.75)
1970-1984	80-85	Personality disorders (inpatient)	75	0.62 (0.61-0.62)	10	0.22 (0.22-0.22)	65	0.86 (0.86-0.86)
1985-1994	80-85	Personality disorders (inpatient)	93	0.86 (0.85-0.86)	14	0.36 (0.36-0.36)	79	1.13 (1.12-1.13)
1995-2004	80-85	Personality disorders (inpatient)	63	0.55 (0.55-0.56)	14	0.33 (0.33-0.34)	49	0.68 (0.68-0.68)
2005-2016	80-85	Personality disorders (inpatient)	41	0.30 (0.29-0.30)	9	0.16 (0.16-0.16)	32	0.39 (0.39-0.39)
1970-2016	85-90	Personality disorders (inpatient)	91	0.35 (0.35-0.35)	18	0.21 (0.21-0.21)	73	0.42 (0.42-0.42)
1970-1984	85-90	Personality disorders (inpatient)	30	0.54 (0.54-0.54)	6	0.30 (0.30-0.30)	24	0.68 (0.68-0.68)
1985-1994	85-90	Personality disorders (inpatient)	27	0.49 (0.49-0.50)	5	0.30 (0.30-0.30)	22	0.58 (0.58-0.58)
1995-2004	85-90	Personality disorders (inpatient)	26	0.40 (0.40-0.40)	5	0.25 (0.25-0.25)	21	0.47 (0.47-0.47)
2005-2016	85-90	Personality disorders (inpatient)	8	0.09 (0.09-0.09)	0-4	NA	4-8	0.11 (0.11-0.11)
1970-2016	90-95	Personality disorders (inpatient)	10	0.11 (0.10-0.11)	0-4	NA	6-10	0.12 (0.12-0.12)
1970-1984	90-95	Personality disorders (inpatient)	0-4	NA	0-4	NA	0-4	NA
1985-1994	90-95	Personality disorders (inpatient)	0-4	NA	0-4	NA	0-4	NA
1995-2004	90-95	Personality disorders (inpatient)	0-4	NA	0-4	NA	0-4	NA
2005-2016	90-95	Personality disorders (inpatient)	0-4	NA	0-4	NA	0-4	NA
1970-2016	95-100	Personality disorders (inpatient)	0-4	NA	0-4	NA	0-4	NA
1970-1984	95-100	Personality disorders (inpatient)	0-4	NA	0-4	NA	0-4	NA
1985-1994	95-100	Personality disorders (inpatient)	0-4	NA	0-4	NA	0-4	NA
1995-2004	95-100	Personality disorders (inpatient)	0-4	NA	0-4	NA	0-4	NA
2005-2016	95-100	Personality disorders (inpatient)	0-4	NA	0-4	NA	0-4	NA
1970-2016	10-15	Personality disorders (borderline-type)	410	0.26 (0.26-0.26)	51	0.06 (0.06-0.06)	359	0.46 (0.46-0.47)
1970-1984	10-15	Personality disorders (borderline-type)	24	0.04 (0.04-0.04)	16	0.05 (0.05-0.05)	8	0.03 (0.03-0.03)
1985-1994	10-15	Personality disorders (borderline-type)	37	0.12 (0.12-0.12)	13	0.08 (0.08-0.08)	24	0.16 (0.16-0.16)
1995-2004	10-15	Personality disorders (borderline-type)	170	0.57 (0.57-0.58)	16	0.11 (0.11-0.11)	154	1.07 (1.07-1.07)
2005-2016	10-15	Personality disorders (borderline-type)	179	0.45 (0.45-0.45)	6	0.03 (0.03-0.03)	173	0.88 (0.88-0.89)
1970-2016	15-20	Personality disorders (borderline-type)	4,187	2.61 (2.60-2.61)	431	0.52 (0.52-0.52)	3,756	4.80 (4.80-4.81)
1970-1984	15-20	Personality disorders (borderline-type)	143	0.25 (0.25-0.25)	84	0.29 (0.29-0.29)	59	0.21 (0.21-0.21)
1985-1994	15-20	Personality disorders (borderline-type)	231	0.65 (0.65-0.65)	79	0.44 (0.44-0.44)	152	0.88 (0.88-0.89)
1995-2004	15-20	Personality disorders (borderline-type)	1,171	4.12 (4.11-4.12)	96	0.66 (0.66-0.66)	1,075	7.75 (7.74-7.76)

			Both sexes		Males		Females	
**Years**	**Age**	**Mental disorder**	**Cases**	**IR (95% CI)**	**Cases**	**IR (95% CI)**	**Cases**	**IR (95% CI)**
2005-2016	15-20	Personality disorders (borderline-type)	2,642	6.63 (6.63-6.64)	172	0.84 (0.84-0.84)	2,470	12.77 (12.75-12.78)
1970-2016	20-25	Personality disorders (borderline-type)	5,782	3.59 (3.59-3.59)	824	0.99 (0.99-0.99)	4,958	6.38 (6.37-6.38)
1970-1984	20-25	Personality disorders (borderline-type)	235	0.42 (0.42-0.42)	125	0.44 (0.44-0.44)	110	0.41 (0.41-0.41)
1985-1994	20-25	Personality disorders (borderline-type)	388	1.04 (1.04-1.04)	158	0.81 (0.81-0.81)	230	1.29 (1.28-1.29)
1995-2004	20-25	Personality disorders (borderline-type)	1,438	4.65 (4.64-4.65)	226	1.41 (1.41-1.41)	1,212	8.12 (8.11-8.13)
2005-2016	20-25	Personality disorders (borderline-type)	3,721	9.95 (9.94-9.96)	315	1.63 (1.63-1.64)	3,406	18.83 (18.80-18.86)
1970-2016	25-30	Personality disorders (borderline-type)	3,807	2.35 (2.35-2.35)	769	0.92 (0.92-0.92)	3,038	3.88 (3.88-3.88)
1970-1984	25-30	Personality disorders (borderline-type)	221	0.39 (0.39-0.39)	112	0.38 (0.38-0.38)	109	0.40 (0.39-0.40)
1985-1994	25-30	Personality disorders (borderline-type)	480	1.30 (1.30-1.30)	175	0.91 (0.91-0.92)	305	1.72 (1.71-1.72)
1995-2004	25-30	Personality disorders (borderline-type)	1,107	3.29 (3.29-3.29)	233	1.34 (1.34-1.34)	874	5.39 (5.38-5.40)
2005-2016	25-30	Personality disorders (borderline-type)	1,999	5.79 (5.78-5.79)	249	1.40 (1.40-1.40)	1,750	10.43 (10.42-10.45)
1970-2016	30-35	Personality disorders (borderline-type)	2,778	1.73 (1.73-1.73)	628	0.76 (0.76-0.76)	2,150	2.75 (2.75-2.75)
1970-1984	30-35	Personality disorders (borderline-type)	188	0.35 (0.35-0.35)	83	0.30 (0.30-0.30)	105	0.39 (0.39-0.39)
1985-1994	30-35	Personality disorders (borderline-type)	451	1.30 (1.30-1.30)	170	0.95 (0.95-0.95)	281	1.67 (1.67-1.67)
1995-2004	30-35	Personality disorders (borderline-type)	860	2.39 (2.39-2.39)	200	1.08 (1.07-1.08)	660	3.79 (3.78-3.79)
2005-2016	30-35	Personality disorders (borderline-type)	1,279	3.60 (3.60-3.61)	175	0.96 (0.96-0.96)	1,104	6.38 (6.37-6.39)
1970-2016	35-40	Personality disorders (borderline-type)	2,058	1.29 (1.29-1.29)	477	0.59 (0.59-0.59)	1,581	2.03 (2.02-2.03)
1970-1984	35-40	Personality disorders (borderline-type)	159	0.32 (0.32-0.32)	74	0.30 (0.30-0.30)	85	0.35 (0.35-0.35)
1985-1994	35-40	Personality disorders (borderline-type)	317	0.91 (0.90-0.91)	111	0.62 (0.62-0.62)	206	1.20 (1.20-1.20)
1995-2004	35-40	Personality disorders (borderline-type)	737	2.05 (2.05-2.05)	159	0.86 (0.86-0.86)	578	3.30 (3.29-3.30)
2005-2016	35-40	Personality disorders (borderline-type)	845	2.19 (2.19-2.19)	133	0.67 (0.67-0.67)	712	3.78 (3.78-3.79)
1970-2016	40-45	Personality disorders (borderline-type)	1,369	0.88 (0.88-0.88)	295	0.37 (0.37-0.37)	1,074	1.39 (1.39-1.40)
1970-1984	40-45	Personality disorders (borderline-type)	98	0.23 (0.23-0.23)	34	0.16 (0.16-0.16)	64	0.29 (0.29-0.30)
1985-1994	40-45	Personality disorders (borderline-type)	256	0.69 (0.69-0.69)	72	0.39 (0.39-0.39)	184	1.01 (1.00-1.01)
1995-2004	40-45	Personality disorders (borderline-type)	459	1.36 (1.35-1.36)	101	0.59 (0.59-0.59)	358	2.15 (2.15-2.15)
2005-2016	40-45	Personality disorders (borderline-type)	556	1.34 (1.34-1.34)	88	0.41 (0.41-0.42)	468	2.30 (2.29-2.30)
1970-2016	45-50	Personality disorders (borderline-type)	829	0.55 (0.55-0.55)	200	0.26 (0.26-0.26)	629	0.83 (0.83-0.83)
1970-1984	45-50	Personality disorders (borderline-type)	95	0.23 (0.23-0.23)	46	0.22 (0.22-0.22)	49	0.23 (0.23-0.23)
1985-1994	45-50	Personality disorders (borderline-type)	153	0.45 (0.45-0.45)	35	0.21 (0.21-0.21)	118	0.70 (0.70-0.70)
1995-2004	45-50	Personality disorders (borderline-type)	295	0.87 (0.87-0.87)	72	0.42 (0.42-0.42)	223	1.32 (1.32-1.32)
2005-2016	45-50	Personality disorders (borderline-type)	286	0.68 (0.68-0.69)	47	0.22 (0.22-0.22)	239	1.16 (1.16-1.16)
1970-2016	50-55	Personality disorders (borderline-type)	506	0.35 (0.35-0.35)	107	0.15 (0.15-0.15)	399	0.55 (0.55-0.55)
1970-1984	50-55	Personality disorders (borderline-type)	84	0.20 (0.20-0.20)	29	0.14 (0.14-0.14)	55	0.26 (0.26-0.26)
1985-1994	50-55	Personality disorders (borderline-type)	77	0.27 (0.27-0.28)	20	0.14 (0.14-0.14)	57	0.40 (0.40-0.40)
1995-2004	50-55	Personality disorders (borderline-type)	166	0.47 (0.47-0.47)	28	0.16 (0.16-0.16)	138	0.78 (0.78-0.78)
2005-2016	50-55	Personality disorders (borderline-type)	179	0.45 (0.45-0.45)	30	0.15 (0.15-0.15)	149	0.76 (0.75-0.76)
1970-2016	55-60	Personality disorders (borderline-type)	230	0.17 (0.17-0.17)	53	0.08 (0.08-0.08)	177	0.25 (0.25-0.25)
1970-1984	55-60	Personality disorders (borderline-type)	49	0.12 (0.12-0.12)	17	0.08 (0.08-0.08)	32	0.15 (0.15-0.15)
1985-1994	55-60	Personality disorders (borderline-type)	33	0.13 (0.13-0.13)	10	0.08 (0.08-0.08)	23	0.18 (0.18-0.18)
1995-2004	55-60	Personality disorders (borderline-type)	70	0.22 (0.22-0.22)	11	0.07 (0.07-0.07)	59	0.37 (0.37-0.37)

			Both sexes		Males		Females	
**Years**	**Age**	**Mental disorder**	**Cases**	**IR (95% CI)**	**Cases**	**IR (95% CI)**	**Cases**	**IR (95% CI)**
2005-2016	55-60	Personality disorders (borderline-type)	78	0.20 (0.20-0.20)	15	0.08 (0.08-0.08)	63	0.33 (0.33-0.33)
1970-2016	60-65	Personality disorders (borderline-type)	118	0.09 (0.09-0.09)	21	0.03 (0.03-0.03)	97	0.15 (0.15-0.15)
1970-1984	60-65	Personality disorders (borderline-type)	34	0.09 (0.09-0.09)	8	0.04 (0.04-0.04)	26	0.13 (0.12-0.13)
1985-1994	60-65	Personality disorders (borderline-type)	19	0.08 (0.08-0.08)	0-4	NA	15-19	0.13 (0.13-0.13)
1995-2004	60-65	Personality disorders (borderline-type)	28	0.11 (0.11-0.11)	0-4	NA	24-28	0.18 (0.18-0.18)
2005-2016	60-65	Personality disorders (borderline-type)	37	0.10 (0.10-0.10)	7	0.04 (0.04-0.04)	30	0.15 (0.15-0.15)
1970-2016	65-70	Personality disorders (borderline-type)	59	0.05 (0.05-0.05)	9	0.02 (0.02-0.02)	50	0.08 (0.08-0.08)
1970-1984	65-70	Personality disorders (borderline-type)	25	0.07 (0.07-0.07)	5	0.03 (0.03-0.03)	20	0.11 (0.11-0.11)
1985-1994	65-70	Personality disorders (borderline-type)	10	0.04 (0.04-0.04)	0-4	NA	6-10	0.07 (0.07-0.07)
1995-2004	65-70	Personality disorders (borderline-type)	10	0.05 (0.05-0.05)	0-4	NA	6-10	0.08 (0.08-0.08)
2005-2016	65-70	Personality disorders (borderline-type)	14	0.04 (0.04-0.04)	0-4	NA	10-14	0.07 (0.07-0.07)
1970-2016	70-75	Personality disorders (borderline-type)	46	0.05 (0.05-0.05)	9	0.02 (0.02-0.02)	37	0.07 (0.07-0.07)
1970-1984	70-75	Personality disorders (borderline-type)	25	0.09 (0.09-0.09)	5	0.04 (0.04-0.04)	20	0.12 (0.12-0.12)
1985-1994	70-75	Personality disorders (borderline-type)	10	0.05 (0.05-0.05)	0-4	NA	6-10	0.07 (0.07-0.07)
1995-2004	70-75	Personality disorders (borderline-type)	5	0.03 (0.03-0.03)	0-4	NA	0-4	NA
2005-2016	70-75	Personality disorders (borderline-type)	6	0.02 (0.02-0.02)	0-4	NA	2-6	0.04 (0.04-0.04)
1970-2016	75-80	Personality disorders (borderline-type)	24	0.03 (0.03-0.03)	6	0.02 (0.02-0.02)	18	0.04 (0.04-0.04)
1970-1984	75-80	Personality disorders (borderline-type)	9	0.04 (0.04-0.04)	0-4	NA	5-9	0.05 (0.05-0.05)
1985-1994	75-80	Personality disorders (borderline-type)	7	0.04 (0.04-0.04)	0-4	NA	0-4	NA
1995-2004	75-80	Personality disorders (borderline-type)	0-4	NA	0-4	NA	0-4	NA
2005-2016	75-80	Personality disorders (borderline-type)	5	0.03 (0.03-0.03)	0-4	NA	1-5	0.05 (0.05-0.05)
1970-2016	80-85	Personality disorders (borderline-type)	16	0.03 (0.03-0.03)	5	0.03 (0.03-0.03)	11	0.04 (0.04-0.04)
1970-1984	80-85	Personality disorders (borderline-type)	10	0.08 (0.08-0.08)	0-4	NA	6-10	0.09 (0.09-0.09)
1985-1994	80-85	Personality disorders (borderline-type)	0-4	NA	0-4	NA	0-4	NA
1995-2004	80-85	Personality disorders (borderline-type)	0-4	NA	0-4	NA	0-4	NA
2005-2016	80-85	Personality disorders (borderline-type)	0-4	NA	0-4	NA	0-4	NA
1970-2016	85-90	Personality disorders (borderline-type)	6	0.02 (0.02-0.02)	0-4	NA	2-6	0.03 (0.03-0.03)
1970-1984	85-90	Personality disorders (borderline-type)	6	0.11 (0.11-0.11)	0-4	NA	2-6	0.17 (0.17-0.17)
1985-1994	85-90	Personality disorders (borderline-type)	0-4	NA	0-4	NA	0-4	NA
1995-2004	85-90	Personality disorders (borderline-type)	0-4	NA	0-4	NA	0-4	NA
2005-2016	85-90	Personality disorders (borderline-type)	0-4	NA	0-4	NA	0-4	NA
1970-2016	90-95	Personality disorders (borderline-type)	0-4	NA	0-4	NA	0-4	NA
1970-1984	90-95	Personality disorders (borderline-type)	0-4	NA	0-4	NA	0-4	NA
1985-1994	90-95	Personality disorders (borderline-type)	0-4	NA	0-4	NA	0-4	NA
1995-2004	90-95	Personality disorders (borderline-type)	0-4	NA	0-4	NA	0-4	NA
2005-2016	90-95	Personality disorders (borderline-type)	0-4	NA	0-4	NA	0-4	NA
1970-2016	95-100	Personality disorders (borderline-type)	0-4	NA	0-4	NA	0-4	NA
1970-1984	95-100	Personality disorders (borderline-type)	0-4	NA	0-4	NA	0-4	NA
1985-1994	95-100	Personality disorders (borderline-type)	0-4	NA	0-4	NA	0-4	NA
1995-2004	95-100	Personality disorders (borderline-type)	0-4	NA	0-4	NA	0-4	NA

			Both sexes		Males		Females	
**Years**	**Age**	**Mental disorder**	**Cases**	**IR (95% CI)**	**Cases**	**IR (95% CI)**	**Cases**	**IR (95% CI)**
2005-2016	95-100	Personality disorders (borderline-type)	0-4	NA	0-4	NA	0-4	NA
1970-2016	10-15	Personality disorders (borderline-type) (inpatient)	169	0.11 (0.11-0.11)	32	0.04 (0.04-0.04)	137	0.18 (0.18-0.18)
1970-1984	10-15	Personality disorders (borderline-type) (inpatient)	24	0.04 (0.04-0.04)	16	0.05 (0.05-0.05)	8	0.03 (0.03-0.03)
1985-1994	10-15	Personality disorders (borderline-type) (inpatient)	30	0.10 (0.09-0.10)	9	0.06 (0.06-0.06)	21	0.14 (0.14-0.14)
1995-2004	10-15	Personality disorders (borderline-type) (inpatient)	69	0.23 (0.23-0.23)	6	0.04 (0.04-0.04)	63	0.44 (0.44-0.44)
2005-2016	10-15	Personality disorders (borderline-type) (inpatient)	46	0.11 (0.11-0.11)	0-4	NA	42-46	0.23 (0.23-0.23)
1970-2016	15-20	Personality disorders (borderline-type) (inpatient)	1674	1.04 (1.04-1.04)	252	0.31 (0.31-0.31)	1422	1.82 (1.82-1.82)
1970-1984	15-20	Personality disorders (borderline-type) (inpatient)	143	0.25 (0.25-0.25)	84	0.29 (0.29-0.29)	59	0.21 (0.21-0.21)
1985-1994	15-20	Personality disorders (borderline-type) (inpatient)	210	0.59 (0.59-0.60)	76	0.42 (0.42-0.42)	134	0.78 (0.78-0.78)
1995-2004	15-20	Personality disorders (borderline-type) (inpatient)	532	1.87 (1.87-1.87)	39	0.27 (0.27-0.27)	493	3.55 (3.55-3.56)
2005-2016	15-20	Personality disorders (borderline-type) (inpatient)	789	1.98 (1.98-1.98)	53	0.26 (0.26-0.26)	736	3.80 (3.79-3.80)
1970-2016	20-25	Personality disorders (borderline-type) (inpatient)	2549	1.58 (1.58-1.58)	483	0.58 (0.58-0.58)	2066	2.65 (2.65-2.65)
1970-1984	20-25	Personality disorders (borderline-type) (inpatient)	235	0.42 (0.42-0.42)	125	0.44 (0.44-0.44)	110	0.41 (0.41-0.41)
1985-1994	20-25	Personality disorders (borderline-type) (inpatient)	353	0.95 (0.95-0.95)	151	0.78 (0.78-0.78)	202	1.13 (1.13-1.13)
1995-2004	20-25	Personality disorders (borderline-type) (inpatient)	723	2.33 (2.33-2.34)	107	0.67 (0.67-0.67)	616	4.12 (4.11-4.13)
2005-2016	20-25	Personality disorders (borderline-type) (inpatient)	1238	3.30 (3.30-3.30)	100	0.52 (0.52-0.52)	1138	6.25 (6.24-6.26)
1970-2016	25-30	Personality disorders (borderline-type) (inpatient)	1903	1.17 (1.17-1.18)	475	0.57 (0.57-0.57)	1428	1.82 (1.82-1.82)
1970-1984	25-30	Personality disorders (borderline-type) (inpatient)	221	0.39 (0.39-0.39)	112	0.38 (0.38-0.38)	109	0.40 (0.39-0.40)
1985-1994	25-30	Personality disorders (borderline-type) (inpatient)	438	1.19 (1.19-1.19)	166	0.87 (0.87-0.87)	272	1.53 (1.53-1.53)
1995-2004	25-30	Personality disorders (borderline-type) (inpatient)	576	1.71 (1.71-1.71)	117	0.67 (0.67-0.67)	459	2.83 (2.82-2.83)
2005-2016	25-30	Personality disorders (borderline-type) (inpatient)	668	1.93 (1.92-1.93)	80	0.45 (0.45-0.45)	588	3.48 (3.47-3.48)
1970-2016	30-35	Personality disorders (borderline-type) (inpatient)	1519	0.95 (0.94-0.95)	419	0.51 (0.51-0.51)	1100	1.40 (1.40-1.41)
1970-1984	30-35	Personality disorders (borderline-type) (inpatient)	188	0.35 (0.35-0.35)	83	0.30 (0.30-0.30)	105	0.39 (0.39-0.39)
1985-1994	30-35	Personality disorders (borderline-type) (inpatient)	401	1.16 (1.16-1.16)	162	0.91 (0.91-0.91)	239	1.42 (1.42-1.42)
1995-2004	30-35	Personality disorders (borderline-type) (inpatient)	490	1.36 (1.36-1.36)	114	0.61 (0.61-0.61)	376	2.15 (2.15-2.16)
2005-2016	30-35	Personality disorders (borderline-type) (inpatient)	440	1.24 (1.23-1.24)	60	0.33 (0.33-0.33)	380	2.18 (2.18-2.19)
1970-2016	35-40	Personality disorders (borderline-type) (inpatient)	1186	0.75 (0.74-0.75)	323	0.40 (0.40-0.40)	863	1.10 (1.10-1.11)
1970-1984	35-40	Personality disorders (borderline-type) (inpatient)	159	0.32 (0.32-0.32)	74	0.30 (0.30-0.30)	85	0.35 (0.35-0.35)
1985-1994	35-40	Personality disorders (borderline-type) (inpatient)	276	0.79 (0.79-0.79)	104	0.58 (0.58-0.58)	172	1.00 (1.00-1.00)
1995-2004	35-40	Personality disorders (borderline-type) (inpatient)	431	1.20 (1.20-1.20)	85	0.46 (0.46-0.46)	346	1.97 (1.97-1.97)
2005-2016	35-40	Personality disorders (borderline-type) (inpatient)	320	0.83 (0.83-0.83)	60	0.30 (0.30-0.30)	260	1.38 (1.37-1.38)
1970-2016	40-45	Personality disorders (borderline-type) (inpatient)	802	0.51 (0.51-0.51)	188	0.24 (0.24-0.24)	614	0.80 (0.80-0.80)
1970-1984	40-45	Personality disorders (borderline-type) (inpatient)	98	0.23 (0.23-0.23)	34	0.16 (0.16-0.16)	64	0.29 (0.29-0.30)
1985-1994	40-45	Personality disorders (borderline-type) (inpatient)	213	0.58 (0.58-0.58)	59	0.32 (0.32-0.32)	154	0.84 (0.84-0.84)
1995-2004	40-45	Personality disorders (borderline-type) (inpatient)	264	0.78 (0.78-0.78)	61	0.35 (0.35-0.35)	203	1.22 (1.22-1.22)
2005-2016	40-45	Personality disorders (borderline-type) (inpatient)	227	0.54 (0.54-0.55)	34	0.16 (0.16-0.16)	193	0.94 (0.94-0.95)
1970-2016	45-50	Personality disorders (borderline-type) (inpatient)	535	0.35 (0.35-0.35)	137	0.18 (0.18-0.18)	398	0.53 (0.53-0.53)
1970-1984	45-50	Personality disorders (borderline-type) (inpatient)	95	0.23 (0.23-0.23)	46	0.22 (0.22-0.22)	49	0.23 (0.23-0.23)
1985-1994	45-50	Personality disorders (borderline-type) (inpatient)	132	0.39 (0.39-0.39)	31	0.18 (0.18-0.18)	101	0.60 (0.60-0.60)
1995-2004	45-50	Personality disorders (borderline-type) (inpatient)	178	0.52 (0.52-0.52)	44	0.26 (0.26-0.26)	134	0.79 (0.79-0.79)

			Both sexes		Males		Females	
**Years**	**Age**	**Mental disorder**	**Cases**	**IR (95% CI)**	**Cases**	**IR (95% CI)**	**Cases**	**IR (95% CI)**
2005-2016	45-50	Personality disorders (borderline-type) (inpatient)	130	0.31 (0.31-0.31)	16	0.08 (0.08-0.08)	114	0.55 (0.55-0.55)
1970-2016	50-55	Personality disorders (borderline-type) (inpatient)	305	0.21 (0.21-0.21)	75	0.10 (0.10-0.10)	230	0.31 (0.31-0.31)
1970-1984	50-55	Personality disorders (borderline-type) (inpatient)	84	0.20 (0.20-0.20)	29	0.14 (0.14-0.14)	55	0.26 (0.26-0.26)
1985-1994	50-55	Personality disorders (borderline-type) (inpatient)	61	0.22 (0.22-0.22)	17	0.12 (0.12-0.12)	44	0.31 (0.31-0.31)
1995-2004	50-55	Personality disorders (borderline-type) (inpatient)	96	0.27 (0.27-0.27)	18	0.10 (0.10-0.10)	78	0.44 (0.44-0.44)
2005-2016	50-55	Personality disorders (borderline-type) (inpatient)	64	0.16 (0.16-0.16)	11	0.06 (0.06-0.06)	53	0.27 (0.27-0.27)
1970-2016	55-60	Personality disorders (borderline-type) (inpatient)	150	0.11 (0.11-0.11)	40	0.06 (0.06-0.06)	110	0.16 (0.16-0.16)
1970-1984	55-60	Personality disorders (borderline-type) (inpatient)	49	0.12 (0.12-0.12)	17	0.08 (0.08-0.08)	32	0.15 (0.15-0.15)
1985-1994	55-60	Personality disorders (borderline-type) (inpatient)	32	0.13 (0.13-0.13)	10	0.08 (0.08-0.08)	22	0.17 (0.17-0.17)
1995-2004	55-60	Personality disorders (borderline-type) (inpatient)	34	0.11 (0.11-0.11)	5	0.03 (0.03-0.03)	29	0.18 (0.18-0.18)
2005-2016	55-60	Personality disorders (borderline-type) (inpatient)	35	0.09 (0.09-0.09)	8	0.04 (0.04-0.04)	27	0.14 (0.14-0.14)
1970-2016	60-65	Personality disorders (borderline-type) (inpatient)	87	0.07 (0.07-0.07)	19	0.03 (0.03-0.03)	68	0.10 (0.10-0.10)
1970-1984	60-65	Personality disorders (borderline-type) (inpatient)	34	0.09 (0.09-0.09)	8	0.04 (0.04-0.04)	26	0.13 (0.12-0.13)
1985-1994	60-65	Personality disorders (borderline-type) (inpatient)	15	0.06 (0.06-0.06)	0-4	NA	11-15	0.10 (0.10-0.10)
1995-2004	60-65	Personality disorders (borderline-type) (inpatient)	15	0.06 (0.06-0.06)	0-4	NA	11-15	0.11 (0.11-0.11)
2005-2016	60-65	Personality disorders (borderline-type) (inpatient)	23	0.06 (0.06-0.06)	8	0.04 (0.04-0.04)	15	0.08 (0.08-0.08)
1970-2016	65-70	Personality disorders (borderline-type) (inpatient)	43	0.04 (0.04-0.04)	9	0.02 (0.02-0.02)	34	0.06 (0.06-0.06)
1970-1984	65-70	Personality disorders (borderline-type) (inpatient)	25	0.07 (0.07-0.07)	5	0.03 (0.03-0.03)	20	0.11 (0.11-0.11)
1985-1994	65-70	Personality disorders (borderline-type) (inpatient)	9	0.04 (0.04-0.04)	0-4	NA	5-9	0.06 (0.06-0.06)
1995-2004	65-70	Personality disorders (borderline-type) (inpatient)	0-4	NA	0-4	NA	0-4	NA
2005-2016	65-70	Personality disorders (borderline-type) (inpatient)	5	0.01 (0.01-0.01)	0-4	NA	0-4	NA
1970-2016	70-75	Personality disorders (borderline-type) (inpatient)	46	0.05 (0.05-0.05)	7	0.02 (0.02-0.02)	39	0.07 (0.07-0.07)
1970-1984	70-75	Personality disorders (borderline-type) (inpatient)	25	0.09 (0.09-0.09)	5	0.04 (0.04-0.04)	20	0.12 (0.12-0.12)
1985-1994	70-75	Personality disorders (borderline-type) (inpatient)	10	0.05 (0.05-0.05)	0-4	NA	6-10	0.07 (0.07-0.07)
1995-2004	70-75	Personality disorders (borderline-type) (inpatient)	0-4	NA	0-4	NA	0-4	NA
2005-2016	70-75	Personality disorders (borderline-type) (inpatient)	9	0.03 (0.03-0.03)	0-4	NA	5-9	0.06 (0.06-0.06)
1970-2016	75-80	Personality disorders (borderline-type) (inpatient)	19	0.03 (0.03-0.03)	6	0.02 (0.02-0.02)	13	0.03 (0.03-0.03)
1970-1984	75-80	Personality disorders (borderline-type) (inpatient)	9	0.04 (0.04-0.04)	0-4	NA	5-9	0.05 (0.05-0.05)
1985-1994	75-80	Personality disorders (borderline-type) (inpatient)	7	0.04 (0.04-0.04)	0-4	NA	0-4	NA
1995-2004	75-80	Personality disorders (borderline-type) (inpatient)	0-4	NA	0-4	NA	0-4	NA
2005-2016	75-80	Personality disorders (borderline-type) (inpatient)	0-4	NA	0-4	NA	0-4	NA
1970-2016	80-85	Personality disorders (borderline-type) (inpatient)	12	0.02 (0.02-0.02)	0-4	NA	8-12	0.03 (0.03-0.03)
1970-1984	80-85	Personality disorders (borderline-type) (inpatient)	10	0.08 (0.08-0.08)	0-4	NA	6-10	0.09 (0.09-0.09)
1985-1994	80-85	Personality disorders (borderline-type) (inpatient)	0-4	NA	0-4	NA	0-4	NA
1995-2004	80-85	Personality disorders (borderline-type) (inpatient)	0-4	NA	0-4	NA	0-4	NA
2005-2016	80-85	Personality disorders (borderline-type) (inpatient)	0-4	NA	0-4	NA	0-4	NA
1970-2016	85-90	Personality disorders (borderline-type) (inpatient)	6	0.02 (0.02-0.02)	0-4	NA	2-6	0.03 (0.03-0.03)
1970-1984	85-90	Personality disorders (borderline-type) (inpatient)	6	0.11 (0.11-0.11)	0-4	NA	2-6	0.17 (0.17-0.17)
1985-1994	85-90	Personality disorders (borderline-type) (inpatient)	0-4	NA	0-4	NA	0-4	NA
1995-2004	85-90	Personality disorders (borderline-type) (inpatient)	0-4	NA	0-4	NA	0-4	NA

			Both sexes		Males		Females	
Years	Age	Mental disorder	Cases	IR (95% CI)	Cases	IR (95% CI)	Cases	IR (95% CI)
2005-2016	85-90	Personality disorders (borderline-type) (inpatient)	0-4	NA	0-4	NA	0-4	NA
1970-2016	90-95	Personality disorders (borderline-type) (inpatient)	0-4	NA	0-4	NA	0-4	NA
1970-1984	90-95	Personality disorders (borderline-type) (inpatient)	0-4	NA	0-4	NA	0-4	NA
1985-1994	90-95	Personality disorders (borderline-type) (inpatient)	0-4	NA	0-4	NA	0-4	NA
1995-2004	90-95	Personality disorders (borderline-type) (inpatient)	0-4	NA	0-4	NA	0-4	NA
2005-2016	90-95	Personality disorders (borderline-type) (inpatient)	0-4	NA	0-4	NA	0-4	NA
1970-2016	95-100	Personality disorders (borderline-type) (inpatient)	0-4	NA	0-4	NA	0-4	NA
1970-1984	95-100	Personality disorders (borderline-type) (inpatient)	0-4	NA	0-4	NA	0-4	NA
1985-1994	95-100	Personality disorders (borderline-type) (inpatient)	0-4	NA	0-4	NA	0-4	NA
1995-2004	95-100	Personality disorders (borderline-type) (inpatient)	0-4	NA	0-4	NA	0-4	NA
2005-2016	95-100	Personality disorders (borderline-type) (inpatient)	0-4	NA	0-4	NA	0-4	NA
1970-2016	10-15	Antisocial personality disorder	109	0.07 (0.07-0.07)	74	0.09 (0.09-0.09)	35	0.05 (0.05-0.05)
1970-1984	10-15	Antisocial personality disorder	51	0.09 (0.09-0.09)	30	0.10 (0.10-0.10)	21	0.08 (0.08-0.08)
1985-1994	10-15	Antisocial personality disorder	0-4	NA	0-4	NA	0-4	NA
1995-2004	10-15	Antisocial personality disorder	34	0.11 (0.11-0.12)	26	0.17 (0.17-0.17)	8	0.06 (0.06-0.06)
2005-2016	10-15	Antisocial personality disorder	20	0.05 (0.05-0.05)	14	0.07 (0.07-0.07)	6	0.03 (0.03-0.03)
1970-2016	15-20	Antisocial personality disorder	1543	0.96 (0.96-0.96)	1084	1.31 (1.31-1.32)	459	0.59 (0.59-0.59)
1970-1984	15-20	Antisocial personality disorder	864	1.51 (1.51-1.51)	564	1.92 (1.92-1.93)	300	1.08 (1.08-1.08)
1985-1994	15-20	Antisocial personality disorder	83	0.24 (0.23-0.24)	60	0.33 (0.33-0.33)	23	0.13 (0.13-0.13)
1995-2004	15-20	Antisocial personality disorder	272	0.96 (0.95-0.96)	210	1.44 (1.44-1.44)	62	0.45 (0.45-0.45)
2005-2016	15-20	Antisocial personality disorder	324	0.81 (0.81-0.81)	250	1.22 (1.22-1.22)	74	0.38 (0.38-0.38)
1970-2016	20-25	Antisocial personality disorder	2799	1.74 (1.73-1.74)	2135	2.56 (2.56-2.56)	664	0.85 (0.85-0.85)
1970-1984	20-25	Antisocial personality disorder	1748	3.15 (3.15-3.16)	1255	4.39 (4.38-4.39)	493	1.84 (1.84-1.84)
1985-1994	20-25	Antisocial personality disorder	205	0.55 (0.55-0.55)	159	0.82 (0.82-0.82)	46	0.26 (0.26-0.26)
1995-2004	20-25	Antisocial personality disorder	410	1.32 (1.32-1.32)	352	2.20 (2.19-2.20)	58	0.39 (0.39-0.39)
2005-2016	20-25	Antisocial personality disorder	436	1.16 (1.16-1.16)	369	1.91 (1.91-1.92)	67	0.37 (0.37-0.37)
1970-2016	25-30	Antisocial personality disorder	2842	1.76 (1.75-1.76)	2122	2.55 (2.54-2.55)	720	0.92 (0.92-0.92)
1970-1984	25-30	Antisocial personality disorder	1886	3.33 (3.33-3.34)	1310	4.51 (4.50-4.51)	576	2.09 (2.09-2.09)
1985-1994	25-30	Antisocial personality disorder	246	0.67 (0.67-0.67)	196	1.03 (1.02-1.03)	50	0.28 (0.28-0.28)
1995-2004	25-30	Antisocial personality disorder	351	1.04 (1.04-1.04)	310	1.78 (1.78-1.78)	41	0.25 (0.25-0.25)
2005-2016	25-30	Antisocial personality disorder	359	1.03 (1.03-1.03)	306	1.72 (1.72-1.73)	53	0.31 (0.31-0.31)
1970-2016	30-35	Antisocial personality disorder	2770	1.73 (1.72-1.73)	2080	2.53 (2.53-2.53)	690	0.88 (0.88-0.88)
1970-1984	30-35	Antisocial personality disorder	1850	3.41 (3.41-3.42)	1297	4.70 (4.69-4.71)	553	2.08 (2.08-2.08)
1985-1994	30-35	Antisocial personality disorder	246	0.71 (0.71-0.71)	179	1.01 (1.00-1.01)	67	0.40 (0.40-0.40)
1995-2004	30-35	Antisocial personality disorder	360	1.00 (1.00-1.00)	325	1.75 (1.75-1.75)	35	0.20 (0.20-0.20)
2005-2016	30-35	Antisocial personality disorder	314	0.88 (0.88-0.88)	279	1.53 (1.53-1.54)	35	0.20 (0.20-0.20)
1970-2016	35-40	Antisocial personality disorder	2382	1.50 (1.50-1.50)	1719	2.13 (2.13-2.13)	663	0.85 (0.85-0.85)
1970-1984	35-40	Antisocial personality disorder	1666	3.37 (3.37-3.38)	1137	4.56 (4.55-4.56)	529	2.17 (2.16-2.17)
1985-1994	35-40	Antisocial personality disorder	196	0.56 (0.56-0.56)	135	0.76 (0.76-0.76)	61	0.35 (0.35-0.36)
1995-2004	35-40	Antisocial personality disorder	327	0.91 (0.91-0.91)	276	1.50 (1.50-1.50)	51	0.29 (0.29-0.29)

			Both sexes		Males		Females	
Years	Age	Mental disorder	Cases	IR (95% CI)	Cases	IR (95% CI)	Cases	IR (95% CI)
2005-2016	35-40	Antisocial personality disorder	193	0.50 (0.50-0.50)	171	0.87 (0.87-0.87)	22	0.12 (0.12-0.12)
1970-2016	40-45	Antisocial personality disorder	1875	1.20 (1.20-1.21)	1371	1.75 (1.74-1.75)	504	0.65 (0.65-0.65)
1970-1984	40-45	Antisocial personality disorder	1353	3.12 (3.12-3.13)	951	4.39 (4.39-4.40)	402	1.85 (1.85-1.86)
1985-1994	40-45	Antisocial personality disorder	152	0.41 (0.41-0.41)	104	0.56 (0.56-0.56)	48	0.26 (0.26-0.26)
1995-2004	40-45	Antisocial personality disorder	203	0.60 (0.60-0.60)	164	0.95 (0.95-0.96)	39	0.23 (0.23-0.23)
2005-2016	40-45	Antisocial personality disorder	167	0.40 (0.40-0.40)	152	0.72 (0.72-0.72)	15	0.07 (0.07-0.07)
1970-2016	45-50	Antisocial personality disorder	1393	0.92 (0.92-0.92)	975	1.29 (1.28-1.29)	418	0.55 (0.55-0.55)
1970-1984	45-50	Antisocial personality disorder	1100	2.62 (2.62-2.62)	744	3.59 (3.58-3.59)	356	1.68 (1.68-1.68)
1985-1994	45-50	Antisocial personality disorder	99	0.29 (0.29-0.29)	61	0.36 (0.36-0.36)	38	0.23 (0.23-0.23)
1995-2004	45-50	Antisocial personality disorder	97	0.29 (0.29-0.29)	82	0.48 (0.48-0.48)	15	0.09 (0.09-0.09)
2005-2016	45-50	Antisocial personality disorder	97	0.23 (0.23-0.23)	88	0.42 (0.42-0.42)	9	0.04 (0.04-0.04)
1970-2016	50-55	Antisocial personality disorder	864	0.60 (0.60-0.60)	594	0.82 (0.82-0.83)	270	0.37 (0.37-0.37)
1970-1984	50-55	Antisocial personality disorder	711	1.68 (1.68-1.68)	471	2.27 (2.27-2.27)	240	1.12 (1.11-1.12)
1985-1994	50-55	Antisocial personality disorder	41	0.15 (0.15-0.15)	30	0.22 (0.22-0.22)	11	0.08 (0.08-0.08)
1995-2004	50-55	Antisocial personality disorder	64	0.18 (0.18-0.18)	55	0.31 (0.31-0.31)	9	0.05 (0.05-0.05)
2005-2016	50-55	Antisocial personality disorder	48	0.12 (0.12-0.12)	38	0.19 (0.19-0.19)	10	0.05 (0.05-0.05)
1970-2016	55-60	Antisocial personality disorder	473	0.34 (0.34-0.34)	326	0.48 (0.48-0.48)	147	0.21 (0.21-0.21)
1970-1984	55-60	Antisocial personality disorder	405	0.97 (0.97-0.97)	269	1.32 (1.32-1.33)	136	0.63 (0.63-0.63)
1985-1994	55-60	Antisocial personality disorder	23	0.09 (0.09-0.09)	16	0.13 (0.13-0.13)	7	0.05 (0.05-0.05)
1995-2004	55-60	Antisocial personality disorder	21	0.07 (0.07-0.07)	17-21	0.13 (0.13-0.13)	0-4	NA
2005-2016	55-60	Antisocial personality disorder	24	0.06 (0.06-0.06)	20-24	0.11 (0.11-0.11)	0-4	NA
1970-2016	60-65	Antisocial personality disorder	243	0.19 (0.19-0.19)	134	0.22 (0.22-0.22)	109	0.16 (0.16-0.16)
1970-1984	60-65	Antisocial personality disorder	201	0.51 (0.50-0.51)	103	0.54 (0.54-0.54)	98	0.47 (0.47-0.47)
1985-1994	60-65	Antisocial personality disorder	16	0.07 (0.07-0.07)	11	0.09 (0.09-0.09)	5	0.04 (0.04-0.04)
1995-2004	60-65	Antisocial personality disorder	15	0.06 (0.06-0.06)	11-15	0.10 (0.10-0.10)	0-4	NA
2005-2016	60-65	Antisocial personality disorder	11	0.03 (0.03-0.03)	7-11	0.04 (0.04-0.04)	0-4	NA
1970-2016	65-70	Antisocial personality disorder	106	0.09 (0.09-0.09)	61	0.11 (0.11-0.11)	45	0.07 (0.07-0.07)
1970-1984	65-70	Antisocial personality disorder	90	0.26 (0.25-0.26)	50	0.31 (0.31-0.31)	40	0.21 (0.21-0.21)
1985-1994	65-70	Antisocial personality disorder	0-4	NA	0-4	NA	0-4	NA
1995-2004	65-70	Antisocial personality disorder	7	0.03 (0.03-0.03)	3-7	0.06 (0.06-0.06)	0-4	NA
2005-2016	65-70	Antisocial personality disorder	7	0.02 (0.02-0.02)	0-4	NA	0-4	NA
1970-2016	70-75	Antisocial personality disorder	59	0.06 (0.06-0.06)	29	0.07 (0.07-0.07)	30	0.06 (0.06-0.06)
1970-1984	70-75	Antisocial personality disorder	34	0.12 (0.12-0.12)	18	0.14 (0.14-0.14)	16	0.10 (0.10-0.10)
1985-1994	70-75	Antisocial personality disorder	5	0.02 (0.02-0.02)	0-4	NA	0-4	NA
1995-2004	70-75	Antisocial personality disorder	13	0.07 (0.07-0.07)	6	0.07 (0.07-0.07)	7	0.07 (0.07-0.07)
2005-2016	70-75	Antisocial personality disorder	7	0.03 (0.03-0.03)	0-4	NA	0-4	NA
1970-2016	75-80	Antisocial personality disorder	39	0.05 (0.05-0.05)	22	0.07 (0.07-0.07)	17	0.04 (0.04-0.04)
1970-1984	75-80	Antisocial personality disorder	21	0.10 (0.10-0.10)	9	0.11 (0.11-0.11)	12	0.10 (0.10-0.10)
1985-1994	75-80	Antisocial personality disorder	6	0.04 (0.04-0.04)	0-4	NA	0-4	NA
1995-2004	75-80	Antisocial personality disorder	5	0.03 (0.03-0.03)	0-4	NA	0-4	NA

			Both sexes		Males		Females	
Years	Age	Mental disorder	Cases	IR (95% CI)	Cases	IR (95% CI)	Cases	IR (95% CI)
2005-2016	75-80	Antisocial personality disorder	7	0.04 (0.04-0.04)	3-7	0.06 (0.06-0.06)	0-4	NA
1970-2016	80-85	Antisocial personality disorder	12	0.02 (0.02-0.02)	8-12	0.04 (0.04-0.04)	0-4	NA
1970-1984	80-85	Antisocial personality disorder	0-4	NA	0-4	NA	0-4	NA
1985-1994	80-85	Antisocial personality disorder	0-4	NA	0-4	NA	0-4	NA
1995-2004	80-85	Antisocial personality disorder	5	0.04 (0.04-0.04)	0-4	NA	0-4	NA
2005-2016	80-85	Antisocial personality disorder	0-4	NA	0-4	NA	0-4	NA
1970-2016	85-90	Antisocial personality disorder	9	0.03 (0.03-0.03)	5-9	0.06 (0.06-0.06)	0-4	NA
1970-1984	85-90	Antisocial personality disorder	0-4	NA	0-4	NA	0-4	NA
1985-1994	85-90	Antisocial personality disorder	0-4	NA	0-4	NA	0-4	NA
1995-2004	85-90	Antisocial personality disorder	5	0.08 (0.08-0.08)	0-4	NA	0-4	NA
2005-2016	85-90	Antisocial personality disorder	0-4	NA	0-4	NA	0-4	NA
1970-2016	90-95	Antisocial personality disorder	0-4	NA	0-4	NA	0-4	NA
1970-1984	90-95	Antisocial personality disorder	0-4	NA	0-4	NA	0-4	NA
1985-1994	90-95	Antisocial personality disorder	0-4	NA	0-4	NA	0-4	NA
1995-2004	90-95	Antisocial personality disorder	0-4	NA	0-4	NA	0-4	NA
2005-2016	90-95	Antisocial personality disorder	0-4	NA	0-4	NA	0-4	NA
1970-2016	95-100	Antisocial personality disorder	0-4	NA	0-4	NA	0-4	NA
1970-1984	95-100	Antisocial personality disorder	0-4	NA	0-4	NA	0-4	NA
1985-1994	95-100	Antisocial personality disorder	0-4	NA	0-4	NA	0-4	NA
1995-2004	95-100	Antisocial personality disorder	0-4	NA	0-4	NA	0-4	NA
2005-2016	95-100	Antisocial personality disorder	0-4	NA	0-4	NA	0-4	NA
1970-2016	10-15	Antisocial personality disorder (inpatient)	68	0.04 (0.04-0.04)	42	0.05 (0.05-0.05)	26	0.03 (0.03-0.03)
1970-1984	10-15	Antisocial personality disorder (inpatient)	51	0.09 (0.09-0.09)	30	0.10 (0.10-0.10)	21	0.08 (0.08-0.08)
1985-1994	10-15	Antisocial personality disorder (inpatient)	0-4	NA	0-4	NA	0-4	NA
1995-2004	10-15	Antisocial personality disorder (inpatient)	12	0.04 (0.04-0.04)	8-12	0.05 (0.05-0.05)	0-4	NA
2005-2016	10-15	Antisocial personality disorder (inpatient)	0-4	NA	0-4	NA	0-4	NA
1970-2016	15-20	Antisocial personality disorder (inpatient)	1110	0.69 (0.69-0.69)	760	0.92 (0.92-0.92)	350	0.45 (0.45-0.45)
1970-1984	15-20	Antisocial personality disorder (inpatient)	864	1.51 (1.51-1.51)	564	1.92 (1.92-1.93)	300	1.08 (1.08-1.08)
1985-1994	15-20	Antisocial personality disorder (inpatient)	79	0.22 (0.22-0.22)	57	0.31 (0.31-0.32)	22	0.13 (0.13-0.13)
1995-2004	15-20	Antisocial personality disorder (inpatient)	92	0.32 (0.32-0.32)	72	0.49 (0.49-0.49)	20	0.14 (0.14-0.14)
2005-2016	15-20	Antisocial personality disorder (inpatient)	75	0.19 (0.19-0.19)	67	0.33 (0.33-0.33)	8	0.04 (0.04-0.04)
1970-2016	20-25	Antisocial personality disorder (inpatient)	2341	1.45 (1.45-1.45)	1750	2.10 (2.10-2.10)	591	0.76 (0.76-0.76)
1970-1984	20-25	Antisocial personality disorder (inpatient)	1748	3.15 (3.15-3.16)	1255	4.39 (4.38-4.39)	493	1.84 (1.84-1.84)
1985-1994	20-25	Antisocial personality disorder (inpatient)	203	0.54 (0.54-0.54)	158	0.81 (0.81-0.81)	45	0.25 (0.25-0.25)
1995-2004	20-25	Antisocial personality disorder (inpatient)	201	0.65 (0.65-0.65)	172	1.07 (1.07-1.07)	29	0.19 (0.19-0.19)
2005-2016	20-25	Antisocial personality disorder (inpatient)	189	0.50 (0.50-0.50)	165	0.85 (0.85-0.86)	24	0.13 (0.13-0.13)
1970-2016	25-30	Antisocial personality disorder (inpatient)	2448	1.51 (1.51-1.51)	1792	2.15 (2.15-2.15)	656	0.83 (0.83-0.84)
1970-1984	25-30	Antisocial personality disorder (inpatient)	1886	3.33 (3.33-3.34)	1310	4.51 (4.50-4.51)	576	2.09 (2.09-2.09)
1985-1994	25-30	Antisocial personality disorder (inpatient)	243	0.66 (0.66-0.66)	193	1.01 (1.01-1.01)	50	0.28 (0.28-0.28)
1995-2004	25-30	Antisocial personality disorder (inpatient)	169	0.50 (0.50-0.50)	153	0.88 (0.88-0.88)	16	0.10 (0.10-0.10)

			Both sexes		Males		Females	
Years	Age	Mental disorder	Cases	IR (95% CI)	Cases	IR (95% CI)	Cases	IR (95% CI)
2005-2016	25-30	Antisocial personality disorder (inpatient)	150	0.43 (0.43-0.43)	136	0.77 (0.76-0.77)	14	0.08 (0.08-0.08)
1970-2016	30-35	Antisocial personality disorder (inpatient)	2405	1.50 (1.50-1.50)	1753	2.13 (2.13-2.13)	652	0.83 (0.83-0.83)
1970-1984	30-35	Antisocial personality disorder (inpatient)	1850	3.41 (3.41-3.42)	1297	4.70 (4.69-4.71)	553	2.08 (2.08-2.08)
1985-1994	30-35	Antisocial personality disorder (inpatient)	243	0.70 (0.70-0.70)	177	0.99 (0.99-1.00)	66	0.39 (0.39-0.39)
1995-2004	30-35	Antisocial personality disorder (inpatient)	177	0.49 (0.49-0.49)	157	0.84 (0.84-0.85)	20	0.11 (0.11-0.11)
2005-2016	30-35	Antisocial personality disorder (inpatient)	135	0.38 (0.38-0.38)	122	0.67 (0.67-0.67)	13	0.07 (0.07-0.07)
1970-2016	35-40	Antisocial personality disorder (inpatient)	2116	1.33 (1.33-1.33)	1494	1.85 (1.85-1.85)	622	0.80 (0.80-0.80)
1970-1984	35-40	Antisocial personality disorder (inpatient)	1666	3.37 (3.37-3.38)	1137	4.56 (4.55-4.56)	529	2.17 (2.16-2.17)
1985-1994	35-40	Antisocial personality disorder (inpatient)	193	0.55 (0.55-0.55)	133	0.75 (0.75-0.75)	60	0.35 (0.35-0.35)
1995-2004	35-40	Antisocial personality disorder (inpatient)	150	0.42 (0.42-0.42)	127	0.69 (0.69-0.69)	23	0.13 (0.13-0.13)
2005-2016	35-40	Antisocial personality disorder (inpatient)	107	0.28 (0.28-0.28)	97	0.49 (0.49-0.49)	10	0.05 (0.05-0.05)
1970-2016	40-45	Antisocial personality disorder (inpatient)	1701	1.09 (1.09-1.09)	1226	1.56 (1.56-1.56)	475	0.62 (0.62-0.62)
1970-1984	40-45	Antisocial personality disorder (inpatient)	1353	3.12 (3.12-3.13)	951	4.39 (4.39-4.40)	402	1.85 (1.85-1.86)
1985-1994	40-45	Antisocial personality disorder (inpatient)	151	0.41 (0.41-0.41)	103	0.56 (0.56-0.56)	48	0.26 (0.26-0.26)
1995-2004	40-45	Antisocial personality disorder (inpatient)	107	0.32 (0.32-0.32)	91	0.53 (0.53-0.53)	16	0.10 (0.10-0.10)
2005-2016	40-45	Antisocial personality disorder (inpatient)	90	0.22 (0.22-0.22)	81	0.38 (0.38-0.38)	9	0.04 (0.04-0.04)
1970-2016	45-50	Antisocial personality disorder (inpatient)	1290	0.85 (0.85-0.85)	887	1.17 (1.17-1.17)	403	0.53 (0.53-0.53)
1970-1984	45-50	Antisocial personality disorder (inpatient)	1100	2.62 (2.62-2.62)	744	3.59 (3.58-3.59)	356	1.68 (1.68-1.68)
1985-1994	45-50	Antisocial personality disorder (inpatient)	98	0.29 (0.29-0.29)	60	0.35 (0.35-0.36)	38	0.23 (0.23-0.23)
1995-2004	45-50	Antisocial personality disorder (inpatient)	40	0.12 (0.12-0.12)	34	0.20 (0.20-0.20)	6	0.04 (0.04-0.04)
2005-2016	45-50	Antisocial personality disorder (inpatient)	52	0.12 (0.12-0.12)	48-52	0.23 (0.23-0.23)	0-4	NA
1970-2016	50-55	Antisocial personality disorder (inpatient)	807	0.56 (0.56-0.56)	550	0.76 (0.76-0.76)	257	0.35 (0.35-0.35)
1970-1984	50-55	Antisocial personality disorder (inpatient)	711	1.68 (1.68-1.68)	471	2.27 (2.27-2.27)	240	1.12 (1.11-1.12)
1985-1994	50-55	Antisocial personality disorder (inpatient)	38	0.14 (0.14-0.14)	27	0.20 (0.19-0.20)	11	0.08 (0.08-0.08)
1995-2004	50-55	Antisocial personality disorder (inpatient)	32	0.09 (0.09-0.09)	28-32	0.16 (0.16-0.17)	0-4	NA
2005-2016	50-55	Antisocial personality disorder (inpatient)	26	0.07 (0.07-0.07)	22-26	0.12 (0.12-0.12)	0-4	NA
1970-2016	55-60	Antisocial personality disorder (inpatient)	449	0.33 (0.33-0.33)	305	0.45 (0.45-0.45)	144	0.21 (0.21-0.21)
1970-1984	55-60	Antisocial personality disorder (inpatient)	405	0.97 (0.97-0.97)	269	1.32 (1.32-1.33)	136	0.63 (0.63-0.63)
1985-1994	55-60	Antisocial personality disorder (inpatient)	22	0.09 (0.09-0.09)	15	0.12 (0.12-0.12)	7	0.05 (0.05-0.05)
1995-2004	55-60	Antisocial personality disorder (inpatient)	12	0.04 (0.04-0.04)	8-12	0.08 (0.08-0.08)	0-4	NA
2005-2016	55-60	Antisocial personality disorder (inpatient)	10	0.03 (0.03-0.03)	6-10	0.05 (0.05-0.05)	0-4	NA
1970-2016	60-65	Antisocial personality disorder (inpatient)	228	0.18 (0.18-0.18)	123	0.20 (0.20-0.20)	105	0.16 (0.16-0.16)
1970-1984	60-65	Antisocial personality disorder (inpatient)	201	0.51 (0.50-0.51)	103	0.54 (0.54-0.54)	98	0.47 (0.47-0.47)
1985-1994	60-65	Antisocial personality disorder (inpatient)	16	0.07 (0.07-0.07)	11	0.09 (0.09-0.09)	5	0.04 (0.04-0.04)
1995-2004	60-65	Antisocial personality disorder (inpatient)	7	0.03 (0.03-0.03)	3-7	0.04 (0.04-0.04)	0-4	NA
2005-2016	60-65	Antisocial personality disorder (inpatient)	0-4	NA	0-4	NA	0-4	NA
1970-2016	65-70	Antisocial personality disorder (inpatient)	94	0.08 (0.08-0.08)	53	0.10 (0.10-0.10)	41	0.07 (0.07-0.07)
1970-1984	65-70	Antisocial personality disorder (inpatient)	90	0.26 (0.25-0.26)	50	0.31 (0.31-0.31)	40	0.21 (0.21-0.21)
1985-1994	65-70	Antisocial personality disorder (inpatient)	0-4	NA	0-4	NA	0-4	NA
1995-2004	65-70	Antisocial personality disorder (inpatient)	0-4	NA	0-4	NA	0-4	NA

			Both sexes		Males		Females	
Years	Age	Mental disorder	Cases	IR (95% CI)	Cases	IR (95% CI)	Cases	IR (95% CI)
2005-2016	65-70	Antisocial personality disorder (inpatient)	0-4	NA	0-4	NA	0-4	NA
1970-2016	70-75	Antisocial personality disorder (inpatient)	41	0.04 (0.04-0.04)	18	0.04 (0.04-0.04)	23	0.04 (0.04-0.04)
1970-1984	70-75	Antisocial personality disorder (inpatient)	34	0.12 (0.12-0.12)	18	0.14 (0.14-0.14)	16	0.10 (0.10-0.10)
1985-1994	70-75	Antisocial personality disorder (inpatient)	0-4	NA	0-4	NA	0-4	NA
1995-2004	70-75	Antisocial personality disorder (inpatient)	0-4	NA	0-4	NA	0-4	NA
2005-2016	70-75	Antisocial personality disorder (inpatient)	0-4	NA	0-4	NA	0-4	NA
1970-2016	75-80	Antisocial personality disorder (inpatient)	31	0.04 (0.04-0.04)	16	0.05 (0.05-0.05)	15	0.04 (0.04-0.04)
1970-1984	75-80	Antisocial personality disorder (inpatient)	21	0.10 (0.10-0.10)	9	0.11 (0.11-0.11)	12	0.10 (0.10-0.10)
1985-1994	75-80	Antisocial personality disorder (inpatient)	6	0.04 (0.04-0.04)	0-4	NA	0-4	NA
1995-2004	75-80	Antisocial personality disorder (inpatient)	0-4	NA	0-4	NA	0-4	NA
2005-2016	75-80	Antisocial personality disorder (inpatient)	0-4	NA	0-4	NA	0-4	NA
1970-2016	80-85	Antisocial personality disorder (inpatient)	5	0.01 (0.01-0.01)	0-4	NA	0-4	NA
1970-1984	80-85	Antisocial personality disorder (inpatient)	0-4	NA	0-4	NA	0-4	NA
1985-1994	80-85	Antisocial personality disorder (inpatient)	0-4	NA	0-4	NA	0-4	NA
1995-2004	80-85	Antisocial personality disorder (inpatient)	0-4	NA	0-4	NA	0-4	NA
2005-2016	80-85	Antisocial personality disorder (inpatient)	0-4	NA	0-4	NA	0-4	NA
1970-2016	85-90	Antisocial personality disorder (inpatient)	0-4	NA	0-4	NA	0-4	NA
1970-1984	85-90	Antisocial personality disorder (inpatient)	0-4	NA	0-4	NA	0-4	NA
1985-1994	85-90	Antisocial personality disorder (inpatient)	0-4	NA	0-4	NA	0-4	NA
1995-2004	85-90	Antisocial personality disorder (inpatient)	0-4	NA	0-4	NA	0-4	NA
2005-2016	85-90	Antisocial personality disorder (inpatient)	0-4	NA	0-4	NA	0-4	NA
1970-2016	90-95	Antisocial personality disorder (inpatient)	0-4	NA	0-4	NA	0-4	NA
1970-1984	90-95	Antisocial personality disorder (inpatient)	0-4	NA	0-4	NA	0-4	NA
1985-1994	90-95	Antisocial personality disorder (inpatient)	0-4	NA	0-4	NA	0-4	NA
1995-2004	90-95	Antisocial personality disorder (inpatient)	0-4	NA	0-4	NA	0-4	NA
2005-2016	90-95	Antisocial personality disorder (inpatient)	0-4	NA	0-4	NA	0-4	NA
1970-2016	95-100	Antisocial personality disorder (inpatient)	0-4	NA	0-4	NA	0-4	NA
1970-1984	95-100	Antisocial personality disorder (inpatient)	0-4	NA	0-4	NA	0-4	NA
1985-1994	95-100	Antisocial personality disorder (inpatient)	0-4	NA	0-4	NA	0-4	NA
1995-2004	95-100	Antisocial personality disorder (inpatient)	0-4	NA	0-4	NA	0-4	NA
2005-2016	95-100	Antisocial personality disorder (inpatient)	0-4	NA	0-4	NA	0-4	NA
1970-2016	1-5	Intellectual disability	1968	1.62 (1.62-1.62)	1449	2.33 (2.33-2.34)	519	0.88 (0.88-0.88)
1970-1984	1-5	Intellectual disability	67	0.16 (0.16-0.16)	40	0.19 (0.19-0.19)	27	0.14 (0.14-0.14)
1985-1994	1-5	Intellectual disability	69	0.30 (0.30-0.30)	43	0.37 (0.36-0.37)	26	0.23 (0.23-0.23)
1995-2004	1-5	Intellectual disability	713	2.63 (2.63-2.63)	504	3.63 (3.62-3.63)	209	1.58 (1.58-1.58)
2005-2016	1-5	Intellectual disability	1119	3.68 (3.68-3.68)	862	5.53 (5.52-5.54)	257	1.73 (1.73-1.74)
1970-2016	5-10	Intellectual disability	3836	2.46 (2.46-2.46)	2817	3.53 (3.53-3.54)	1019	1.34 (1.34-1.34)
1970-1984	5-10	Intellectual disability	119	0.21 (0.21-0.21)	87	0.31 (0.30-0.31)	32	0.12 (0.12-0.12)
1985-1994	5-10	Intellectual disability	95	0.34 (0.34-0.34)	56	0.39 (0.39-0.39)	39	0.28 (0.28-0.29)
1995-2004	5-10	Intellectual disability	1099	3.34 (3.34-3.35)	810	4.80 (4.80-4.81)	289	1.80 (1.80-1.81)

			Both sexes		Males		Females	
**Years**	**Age**	**Mental disorder**	**Cases**	**IR (95% CI)**	**Cases**	**IR (95% CI)**	**Cases**	**IR (95% CI)**
2005-2016	5-10	Intellectual disability	2523	6.44 (6.43-6.44)	1864	9.30 (9.29-9.31)	659	3.44 (3.44-3.45)
1970-2016	10-15	Intellectual disability	3547	2.25 (2.24-2.25)	2303	2.85 (2.85-2.85)	1244	1.61 (1.61-1.61)
1970-1984	10-15	Intellectual disability	64	0.11 (0.11-0.11)	39	0.13 (0.13-0.13)	25	0.09 (0.09-0.09)
1985-1994	10-15	Intellectual disability	64	0.20 (0.20-0.20)	36	0.22 (0.22-0.22)	28	0.18 (0.18-0.18)
1995-2004	10-15	Intellectual disability	807	2.73 (2.73-2.74)	521	3.44 (3.44-3.45)	286	1.99 (1.98-1.99)
2005-2016	10-15	Intellectual disability	2612	6.54 (6.54-6.55)	1707	8.36 (8.35-8.38)	905	4.64 (4.63-4.64)
1970-2016	15-20	Intellectual disability	3335	2.08 (2.08-2.08)	1841	2.24 (2.24-2.24)	1494	1.91 (1.91-1.91)
1970-1984	15-20	Intellectual disability	133	0.23 (0.23-0.23)	74	0.25 (0.25-0.25)	59	0.21 (0.21-0.21)
1985-1994	15-20	Intellectual disability	85	0.24 (0.24-0.24)	47	0.26 (0.26-0.26)	38	0.22 (0.22-0.22)
1995-2004	15-20	Intellectual disability	598	2.10 (2.10-2.11)	362	2.49 (2.48-2.49)	236	1.70 (1.70-1.70)
2005-2016	15-20	Intellectual disability	2519	6.36 (6.35-6.36)	1358	6.69 (6.68-6.70)	1161	6.01 (6.00-6.02)
1970-2016	20-25	Intellectual disability	2068	1.28 (1.28-1.28)	1125	1.35 (1.35-1.35)	943	1.21 (1.21-1.21)
1970-1984	20-25	Intellectual disability	195	0.35 (0.35-0.35)	116	0.40 (0.40-0.41)	79	0.29 (0.29-0.29)
1985-1994	20-25	Intellectual disability	110	0.29 (0.29-0.30)	64	0.33 (0.33-0.33)	46	0.26 (0.26-0.26)
1995-2004	20-25	Intellectual disability	461	1.49 (1.49-1.49)	260	1.62 (1.62-1.63)	201	1.34 (1.34-1.34)
2005-2016	20-25	Intellectual disability	1302	3.48 (3.48-3.49)	685	3.57 (3.57-3.58)	617	3.39 (3.39-3.40)
1970-2016	25-30	Intellectual disability	1562	0.96 (0.96-0.96)	849	1.02 (1.02-1.02)	713	0.91 (0.91-0.91)
1970-1984	25-30	Intellectual disability	188	0.33 (0.33-0.33)	105	0.36 (0.36-0.36)	83	0.30 (0.30-0.30)
1985-1994	25-30	Intellectual disability	126	0.34 (0.34-0.34)	74	0.39 (0.39-0.39)	52	0.29 (0.29-0.29)
1995-2004	25-30	Intellectual disability	505	1.50 (1.50-1.50)	283	1.62 (1.62-1.63)	222	1.36 (1.36-1.37)
2005-2016	25-30	Intellectual disability	743	2.15 (2.14-2.15)	387	2.19 (2.18-2.19)	356	2.10 (2.10-2.11)
1970-2016	30-35	Intellectual disability	1385	0.86 (0.86-0.86)	763	0.93 (0.93-0.93)	622	0.79 (0.79-0.79)
1970-1984	30-35	Intellectual disability	185	0.34 (0.34-0.34)	111	0.40 (0.40-0.40)	74	0.28 (0.28-0.28)
1985-1994	30-35	Intellectual disability	148	0.43 (0.43-0.43)	90	0.50 (0.50-0.51)	58	0.34 (0.34-0.35)
1995-2004	30-35	Intellectual disability	524	1.45 (1.45-1.45)	280	1.51 (1.50-1.51)	244	1.40 (1.39-1.40)
2005-2016	30-35	Intellectual disability	528	1.48 (1.48-1.48)	282	1.55 (1.55-1.55)	246	1.41 (1.41-1.41)
1970-2016	35-40	Intellectual disability	1415	0.89 (0.89-0.89)	754	0.93 (0.93-0.93)	661	0.85 (0.84-0.85)
1970-1984	35-40	Intellectual disability	185	0.37 (0.37-0.37)	93	0.37 (0.37-0.37)	92	0.38 (0.38-0.38)
1985-1994	35-40	Intellectual disability	138	0.39 (0.39-0.39)	70	0.39 (0.39-0.39)	68	0.39 (0.39-0.40)
1995-2004	35-40	Intellectual disability	497	1.38 (1.38-1.38)	263	1.43 (1.43-1.43)	234	1.33 (1.33-1.33)
2005-2016	35-40	Intellectual disability	595	1.54 (1.54-1.54)	328	1.66 (1.66-1.67)	267	1.41 (1.41-1.41)
1970-2016	40-45	Intellectual disability	1303	0.84 (0.84-0.84)	719	0.91 (0.91-0.91)	584	0.76 (0.76-0.76)
1970-1984	40-45	Intellectual disability	165	0.38 (0.38-0.38)	74	0.34 (0.34-0.34)	91	0.42 (0.42-0.42)
1985-1994	40-45	Intellectual disability	126	0.34 (0.34-0.34)	60	0.32 (0.32-0.32)	66	0.36 (0.36-0.36)
1995-2004	40-45	Intellectual disability	452	1.33 (1.33-1.33)	257	1.49 (1.49-1.49)	195	1.17 (1.17-1.17)
2005-2016	40-45	Intellectual disability	560	1.34 (1.34-1.35)	328	1.55 (1.55-1.55)	232	1.13 (1.13-1.14)
1970-2016	45-50	Intellectual disability	1212	0.80 (0.80-0.80)	605	0.80 (0.79-0.80)	607	0.80 (0.80-0.80)
1970-1984	45-50	Intellectual disability	131	0.31 (0.31-0.31)	55	0.26 (0.26-0.26)	76	0.36 (0.36-0.36)
1985-1994	45-50	Intellectual disability	128	0.38 (0.38-0.38)	48	0.28 (0.28-0.28)	80	0.47 (0.47-0.47)
1995-2004	45-50	Intellectual disability	416	1.22 (1.22-1.23)	217	1.27 (1.27-1.27)	199	1.18 (1.18-1.18)

			Both sexes		Males		Females	
**Years**	**Age**	**Mental disorder**	**Cases**	**IR (95% CI)**	**Cases**	**IR (95% CI)**	**Cases**	**IR (95% CI)**
2005-2016	45-50	Intellectual disability	537	1.28 (1.28-1.29)	285	1.35 (1.35-1.35)	252	1.22 (1.22-1.22)
1970-2016	50-55	Intellectual disability	1088	0.75 (0.75-0.75)	568	0.79 (0.79-0.79)	520	0.71 (0.71-0.71)
1970-1984	50-55	Intellectual disability	115	0.27 (0.27-0.27)	52	0.25 (0.25-0.25)	63	0.29 (0.29-0.29)
1985-1994	50-55	Intellectual disability	96	0.34 (0.34-0.34)	51	0.37 (0.37-0.37)	45	0.32 (0.32-0.32)
1995-2004	50-55	Intellectual disability	375	1.06 (1.06-1.06)	213	1.21 (1.21-1.21)	162	0.91 (0.91-0.92)
2005-2016	50-55	Intellectual disability	502	1.27 (1.26-1.27)	252	1.27 (1.26-1.27)	250	1.27 (1.26-1.27)
1970-2016	55-60	Intellectual disability	871	0.63 (0.63-0.63)	456	0.67 (0.67-0.68)	415	0.59 (0.59-0.59)
1970-1984	55-60	Intellectual disability	90	0.22 (0.21-0.22)	39	0.19 (0.19-0.19)	51	0.24 (0.24-0.24)
1985-1994	55-60	Intellectual disability	66	0.26 (0.26-0.26)	26	0.21 (0.21-0.21)	40	0.31 (0.31-0.31)
1995-2004	55-60	Intellectual disability	325	1.02 (1.02-1.02)	158	1.00 (1.00-1.00)	167	1.04 (1.04-1.04)
2005-2016	55-60	Intellectual disability	390	1.01 (1.01-1.02)	233	1.22 (1.22-1.22)	157	0.81 (0.81-0.81)
1970-2016	60-65	Intellectual disability	611	0.48 (0.48-0.48)	320	0.52 (0.51-0.52)	291	0.44 (0.44-0.44)
1970-1984	60-65	Intellectual disability	51	0.13 (0.13-0.13)	23	0.12 (0.12-0.12)	28	0.13 (0.13-0.13)
1985-1994	60-65	Intellectual disability	55	0.22 (0.22-0.22)	34	0.29 (0.29-0.29)	21	0.16 (0.16-0.16)
1995-2004	60-65	Intellectual disability	230	0.90 (0.90-0.90)	109	0.88 (0.88-0.88)	121	0.92 (0.92-0.92)
2005-2016	60-65	Intellectual disability	275	0.71 (0.71-0.71)	154	0.81 (0.81-0.81)	121	0.62 (0.62-0.62)
1970-2016	65-70	Intellectual disability	473	0.41 (0.41-0.41)	216	0.40 (0.40-0.40)	257	0.42 (0.42-0.42)
1970-1984	65-70	Intellectual disability	45	0.13 (0.13-0.13)	15	0.09 (0.09-0.09)	30	0.16 (0.16-0.16)
1985-1994	65-70	Intellectual disability	53	0.22 (0.22-0.23)	26	0.24 (0.24-0.24)	27	0.21 (0.21-0.21)
1995-2004	65-70	Intellectual disability	170	0.78 (0.78-0.78)	71	0.69 (0.69-0.69)	99	0.86 (0.86-0.86)
2005-2016	65-70	Intellectual disability	205	0.59 (0.59-0.59)	104	0.62 (0.62-0.63)	101	0.57 (0.56-0.57)
1970-2016	70-75	Intellectual disability	329	0.35 (0.35-0.35)	151	0.35 (0.35-0.35)	178	0.34 (0.34-0.34)
1970-1984	70-75	Intellectual disability	21	0.07 (0.07-0.07)	10	0.08 (0.08-0.08)	11	0.07 (0.07-0.07)
1985-1994	70-75	Intellectual disability	33	0.16 (0.16-0.16)	18	0.20 (0.20-0.20)	15	0.13 (0.13-0.13)
1995-2004	70-75	Intellectual disability	139	0.72 (0.72-0.72)	54	0.62 (0.62-0.62)	85	0.80 (0.79-0.80)
2005-2016	70-75	Intellectual disability	136	0.51 (0.51-0.51)	69	0.55 (0.55-0.56)	67	0.47 (0.47-0.47)
1970-2016	75-80	Intellectual disability	203	0.28 (0.28-0.28)	86	0.28 (0.28-0.28)	117	0.28 (0.28-0.28)
1970-1984	75-80	Intellectual disability	11	0.05 (0.05-0.05)	0-4	NA	7-11	0.07 (0.07-0.07)
1985-1994	75-80	Intellectual disability	12	0.07 (0.07-0.07)	0-4	NA	8-12	0.08 (0.08-0.08)
1995-2004	75-80	Intellectual disability	101	0.62 (0.62-0.62)	41	0.61 (0.61-0.61)	60	0.63 (0.63-0.63)
2005-2016	75-80	Intellectual disability	79	0.40 (0.40-0.40)	38	0.44 (0.43-0.44)	41	0.37 (0.37-0.37)
1970-2016	80-85	Intellectual disability	99	0.20 (0.20-0.20)	34	0.19 (0.18-0.19)	65	0.21 (0.21-0.21)
1970-1984	80-85	Intellectual disability	0-4	NA	0-4	NA	0-4	NA
1985-1994	80-85	Intellectual disability	5	0.05 (0.05-0.05)	0-4	NA	0-4	NA
1995-2004	80-85	Intellectual disability	50	0.44 (0.44-0.44)	16	0.38 (0.38-0.38)	34	0.47 (0.47-0.47)
2005-2016	80-85	Intellectual disability	43	0.31 (0.30-0.31)	16	0.28 (0.28-0.28)	27	0.32 (0.32-0.32)
1970-2016	85-90	Intellectual disability	46	0.18 (0.18-0.18)	12	0.14 (0.14-0.14)	34	0.19 (0.19-0.19)
1970-1984	85-90	Intellectual disability	0-4	NA	0-4	NA	0-4	NA
1985-1994	85-90	Intellectual disability	0-4	NA	0-4	NA	0-4	NA
1995-2004	85-90	Intellectual disability	21	0.32 (0.32-0.32)	6	0.30 (0.30-0.30)	15	0.33 (0.33-0.34)

			Both sexes		Males		Females	
Years	Age	Mental disorder	Cases	IR (95% CI)	Cases	IR (95% CI)	Cases	IR (95% CI)
2005-2016	85-90	Intellectual disability	24	0.28 (0.28-0.28)	6	0.21 (0.20-0.21)	18	0.32 (0.32-0.32)
1970-2016	90-95	Intellectual disability	6	0.06 (0.06-0.06)	0-4	NA	0-4	NA
1970-1984	90-95	Intellectual disability	0-4	NA	0-4	NA	0-4	NA
1985-1994	90-95	Intellectual disability	0-4	NA	0-4	NA	0-4	NA
1995-2004	90-95	Intellectual disability	0-4	NA	0-4	NA	0-4	NA
2005-2016	90-95	Intellectual disability	0-4	NA	0-4	NA	0-4	NA
1970-2016	95-100	Intellectual disability	0-4	NA	0-4	NA	0-4	NA
1970-1984	95-100	Intellectual disability	0-4	NA	0-4	NA	0-4	NA
1985-1994	95-100	Intellectual disability	0-4	NA	0-4	NA	0-4	NA
1995-2004	95-100	Intellectual disability	0-4	NA	0-4	NA	0-4	NA
2005-2016	95-100	Intellectual disability	0-4	NA	0-4	NA	0-4	NA
1970-2016	1-5	Intellectual disability (inpatient)	217	0.18 (0.18-0.18)	153	0.25 (0.25-0.25)	64	0.11 (0.11-0.11)
1970-1984	1-5	Intellectual disability (inpatient)	67	0.16 (0.16-0.16)	40	0.19 (0.19-0.19)	27	0.14 (0.14-0.14)
1985-1994	1-5	Intellectual disability (inpatient)	58	0.25 (0.25-0.25)	38	0.32 (0.32-0.32)	20	0.18 (0.18-0.18)
1995-2004	1-5	Intellectual disability (inpatient)	83	0.31 (0.31-0.31)	67	0.48 (0.48-0.48)	16	0.12 (0.12-0.12)
2005-2016	1-5	Intellectual disability (inpatient)	9	0.03 (0.03-0.03)	5-9	0.05 (0.05-0.05)	0-4	NA
1970-2016	5-10	Intellectual disability (inpatient)	331	0.21 (0.21-0.21)	236	0.30 (0.30-0.30)	95	0.12 (0.12-0.12)
1970-1984	5-10	Intellectual disability (inpatient)	119	0.21 (0.21-0.21)	87	0.31 (0.30-0.31)	32	0.12 (0.12-0.12)
1985-1994	5-10	Intellectual disability (inpatient)	68	0.24 (0.24-0.24)	38	0.27 (0.27-0.27)	30	0.22 (0.22-0.22)
1995-2004	5-10	Intellectual disability (inpatient)	118	0.36 (0.36-0.36)	90	0.53 (0.53-0.53)	28	0.17 (0.17-0.17)
2005-2016	5-10	Intellectual disability (inpatient)	26	0.07 (0.07-0.07)	21	0.10 (0.10-0.10)	5	0.03 (0.03-0.03)
1970-2016	10-15	Intellectual disability (inpatient)	382	0.24 (0.24-0.24)	214	0.26 (0.26-0.26)	168	0.22 (0.22-0.22)
1970-1984	10-15	Intellectual disability (inpatient)	64	0.11 (0.11-0.11)	39	0.13 (0.13-0.13)	25	0.09 (0.09-0.09)
1985-1994	10-15	Intellectual disability (inpatient)	39	0.12 (0.12-0.12)	17	0.11 (0.11-0.11)	22	0.14 (0.14-0.14)
1995-2004	10-15	Intellectual disability (inpatient)	98	0.33 (0.33-0.33)	56	0.37 (0.37-0.37)	42	0.29 (0.29-0.29)
2005-2016	10-15	Intellectual disability (inpatient)	181	0.45 (0.45-0.45)	102	0.50 (0.50-0.50)	79	0.40 (0.40-0.40)
1970-2016	15-20	Intellectual disability (inpatient)	865	0.54 (0.54-0.54)	437	0.53 (0.53-0.53)	428	0.55 (0.55-0.55)
1970-1984	15-20	Intellectual disability (inpatient)	133	0.23 (0.23-0.23)	74	0.25 (0.25-0.25)	59	0.21 (0.21-0.21)
1985-1994	15-20	Intellectual disability (inpatient)	57	0.16 (0.16-0.16)	25	0.14 (0.14-0.14)	32	0.19 (0.19-0.19)
1995-2004	15-20	Intellectual disability (inpatient)	145	0.51 (0.51-0.51)	79	0.54 (0.54-0.54)	66	0.48 (0.47-0.48)
2005-2016	15-20	Intellectual disability (inpatient)	530	1.33 (1.33-1.33)	259	1.27 (1.26-1.27)	271	1.40 (1.40-1.40)
1970-2016	20-25	Intellectual disability (inpatient)	847	0.52 (0.52-0.53)	492	0.59 (0.59-0.59)	355	0.46 (0.45-0.46)
1970-1984	20-25	Intellectual disability (inpatient)	195	0.35 (0.35-0.35)	116	0.40 (0.40-0.41)	79	0.29 (0.29-0.29)
1985-1994	20-25	Intellectual disability (inpatient)	76	0.20 (0.20-0.20)	46	0.24 (0.24-0.24)	30	0.17 (0.17-0.17)
1995-2004	20-25	Intellectual disability (inpatient)	142	0.46 (0.46-0.46)	84	0.52 (0.52-0.52)	58	0.39 (0.39-0.39)
2005-2016	20-25	Intellectual disability (inpatient)	434	1.16 (1.16-1.16)	246	1.28 (1.27-1.28)	188	1.03 (1.03-1.03)
1970-2016	25-30	Intellectual disability (inpatient)	676	0.42 (0.42-0.42)	381	0.46 (0.46-0.46)	295	0.38 (0.38-0.38)
1970-1984	25-30	Intellectual disability (inpatient)	188	0.33 (0.33-0.33)	105	0.36 (0.36-0.36)	83	0.30 (0.30-0.30)
1985-1994	25-30	Intellectual disability (inpatient)	84	0.23 (0.23-0.23)	49	0.26 (0.26-0.26)	35	0.20 (0.20-0.20)
1995-2004	25-30	Intellectual disability (inpatient)	169	0.50 (0.50-0.50)	94	0.54 (0.54-0.54)	75	0.46 (0.46-0.46)

			Both sexes		Males		Females	
**Years**	**Age**	**Mental disorder**	**Cases**	**IR (95% CI)**	**Cases**	**IR (95% CI)**	**Cases**	**IR (95% CI)**
2005-2016	25-30	Intellectual disability (inpatient)	235	0.68 (0.68-0.68)	133	0.75 (0.75-0.75)	102	0.60 (0.60-0.60)
1970-2016	30-35	Intellectual disability (inpatient)	647	0.40 (0.40-0.40)	363	0.44 (0.44-0.44)	284	0.36 (0.36-0.36)
1970-1984	30-35	Intellectual disability (inpatient)	185	0.34 (0.34-0.34)	111	0.40 (0.40-0.40)	74	0.28 (0.28-0.28)
1985-1994	30-35	Intellectual disability (inpatient)	91	0.26 (0.26-0.26)	57	0.32 (0.32-0.32)	34	0.20 (0.20-0.20)
1995-2004	30-35	Intellectual disability (inpatient)	200	0.55 (0.55-0.55)	101	0.54 (0.54-0.54)	99	0.57 (0.57-0.57)
2005-2016	30-35	Intellectual disability (inpatient)	171	0.48 (0.48-0.48)	94	0.52 (0.52-0.52)	77	0.44 (0.44-0.44)
1970-2016	35-40	Intellectual disability (inpatient)	584	0.37 (0.37-0.37)	295	0.36 (0.36-0.36)	289	0.37 (0.37-0.37)
1970-1984	35-40	Intellectual disability (inpatient)	185	0.37 (0.37-0.37)	93	0.37 (0.37-0.37)	92	0.38 (0.38-0.38)
1985-1994	35-40	Intellectual disability (inpatient)	71	0.20 (0.20-0.20)	33	0.19 (0.19-0.19)	38	0.22 (0.22-0.22)
1995-2004	35-40	Intellectual disability (inpatient)	149	0.41 (0.41-0.41)	79	0.43 (0.43-0.43)	70	0.40 (0.40-0.40)
2005-2016	35-40	Intellectual disability (inpatient)	179	0.46 (0.46-0.46)	90	0.46 (0.46-0.46)	89	0.47 (0.47-0.47)
1970-2016	40-45	Intellectual disability (inpatient)	544	0.35 (0.35-0.35)	276	0.35 (0.35-0.35)	268	0.35 (0.35-0.35)
1970-1984	40-45	Intellectual disability (inpatient)	165	0.38 (0.38-0.38)	74	0.34 (0.34-0.34)	91	0.42 (0.42-0.42)
1985-1994	40-45	Intellectual disability (inpatient)	63	0.17 (0.17-0.17)	31	0.17 (0.17-0.17)	32	0.17 (0.17-0.18)
1995-2004	40-45	Intellectual disability (inpatient)	133	0.39 (0.39-0.39)	75	0.44 (0.43-0.44)	58	0.35 (0.35-0.35)
2005-2016	40-45	Intellectual disability (inpatient)	183	0.44 (0.44-0.44)	96	0.45 (0.45-0.45)	87	0.42 (0.42-0.43)
1970-2016	45-50	Intellectual disability (inpatient)	468	0.31 (0.31-0.31)	220	0.29 (0.29-0.29)	248	0.33 (0.33-0.33)
1970-1984	45-50	Intellectual disability (inpatient)	131	0.31 (0.31-0.31)	55	0.26 (0.26-0.26)	76	0.36 (0.36-0.36)
1985-1994	45-50	Intellectual disability (inpatient)	81	0.24 (0.24-0.24)	30	0.18 (0.18-0.18)	51	0.30 (0.30-0.30)
1995-2004	45-50	Intellectual disability (inpatient)	132	0.39 (0.39-0.39)	69	0.40 (0.40-0.40)	63	0.37 (0.37-0.37)
2005-2016	45-50	Intellectual disability (inpatient)	124	0.30 (0.30-0.30)	66	0.31 (0.31-0.31)	58	0.28 (0.28-0.28)
1970-2016	50-55	Intellectual disability (inpatient)	397	0.27 (0.27-0.27)	197	0.27 (0.27-0.27)	200	0.27 (0.27-0.27)
1970-1984	50-55	Intellectual disability (inpatient)	115	0.27 (0.27-0.27)	52	0.25 (0.25-0.25)	63	0.29 (0.29-0.29)
1985-1994	50-55	Intellectual disability (inpatient)	61	0.22 (0.22-0.22)	30	0.22 (0.22-0.22)	31	0.22 (0.22-0.22)
1995-2004	50-55	Intellectual disability (inpatient)	114	0.32 (0.32-0.32)	61	0.35 (0.35-0.35)	53	0.30 (0.30-0.30)
2005-2016	50-55	Intellectual disability (inpatient)	107	0.27 (0.27-0.27)	54	0.27 (0.27-0.27)	53	0.27 (0.27-0.27)
1970-2016	55-60	Intellectual disability (inpatient)	305	0.22 (0.22-0.22)	144	0.21 (0.21-0.21)	161	0.23 (0.23-0.23)
1970-1984	55-60	Intellectual disability (inpatient)	90	0.22 (0.21-0.22)	39	0.19 (0.19-0.19)	51	0.24 (0.24-0.24)
1985-1994	55-60	Intellectual disability (inpatient)	37	0.15 (0.15-0.15)	11	0.09 (0.09-0.09)	26	0.20 (0.20-0.20)
1995-2004	55-60	Intellectual disability (inpatient)	94	0.30 (0.29-0.30)	46	0.29 (0.29-0.29)	48	0.30 (0.30-0.30)
2005-2016	55-60	Intellectual disability (inpatient)	84	0.22 (0.22-0.22)	48	0.25 (0.25-0.25)	36	0.19 (0.19-0.19)
1970-2016	60-65	Intellectual disability (inpatient)	234	0.18 (0.18-0.18)	112	0.18 (0.18-0.18)	122	0.18 (0.18-0.18)
1970-1984	60-65	Intellectual disability (inpatient)	51	0.13 (0.13-0.13)	23	0.12 (0.12-0.12)	28	0.13 (0.13-0.13)
1985-1994	60-65	Intellectual disability (inpatient)	39	0.16 (0.16-0.16)	22	0.19 (0.19-0.19)	17	0.13 (0.13-0.13)
1995-2004	60-65	Intellectual disability (inpatient)	77	0.30 (0.30-0.30)	33	0.27 (0.27-0.27)	44	0.34 (0.33-0.34)
2005-2016	60-65	Intellectual disability (inpatient)	67	0.17 (0.17-0.17)	34	0.18 (0.18-0.18)	33	0.17 (0.17-0.17)
1970-2016	65-70	Intellectual disability (inpatient)	165	0.14 (0.14-0.14)	76	0.14 (0.14-0.14)	89	0.15 (0.15-0.15)
1970-1984	65-70	Intellectual disability (inpatient)	45	0.13 (0.13-0.13)	15	0.09 (0.09-0.09)	30	0.16 (0.16-0.16)
1985-1994	65-70	Intellectual disability (inpatient)	37	0.16 (0.16-0.16)	21	0.19 (0.19-0.19)	16	0.13 (0.13-0.13)
1995-2004	65-70	Intellectual disability (inpatient)	43	0.20 (0.20-0.20)	19	0.18 (0.18-0.19)	24	0.21 (0.21-0.21)

			Both sexes		Males		Females	
Years	Age	Mental disorder	Cases	IR (95% CI)	Cases	IR (95% CI)	Cases	IR (95% CI)
2005-2016	65-70	Intellectual disability (inpatient)	40	0.12 (0.12-0.12)	21	0.13 (0.13-0.13)	19	0.11 (0.11-0.11)
1970-2016	70-75	Intellectual disability (inpatient)	100	0.10 (0.10-0.10)	50	0.12 (0.12-0.12)	50	0.10 (0.09-0.10)
1970-1984	70-75	Intellectual disability (inpatient)	21	0.07 (0.07-0.07)	10	0.08 (0.08-0.08)	11	0.07 (0.07-0.07)
1985-1994	70-75	Intellectual disability (inpatient)	20	0.10 (0.10-0.10)	11	0.12 (0.12-0.12)	9	0.08 (0.08-0.08)
1995-2004	70-75	Intellectual disability (inpatient)	25	0.13 (0.13-0.13)	9	0.10 (0.10-0.10)	16	0.15 (0.15-0.15)
2005-2016	70-75	Intellectual disability (inpatient)	34	0.13 (0.13-0.13)	20	0.16 (0.16-0.16)	14	0.10 (0.10-0.10)
1970-2016	75-80	Intellectual disability (inpatient)	61	0.08 (0.08-0.08)	24	0.08 (0.08-0.08)	37	0.09 (0.09-0.09)
1970-1984	75-80	Intellectual disability (inpatient)	11	0.05 (0.05-0.05)	0-4	NA	7-11	0.07 (0.07-0.07)
1985-1994	75-80	Intellectual disability (inpatient)	8	0.05 (0.05-0.05)	0-4	NA	4-8	0.05 (0.05-0.05)
1995-2004	75-80	Intellectual disability (inpatient)	25	0.15 (0.15-0.15)	10	0.15 (0.15-0.15)	15	0.16 (0.16-0.16)
2005-2016	75-80	Intellectual disability (inpatient)	17	0.09 (0.09-0.09)	8	0.09 (0.09-0.09)	9	0.08 (0.08-0.08)
1970-2016	80-85	Intellectual disability (inpatient)	23	0.05 (0.05-0.05)	10	0.05 (0.05-0.05)	13	0.04 (0.04-0.04)
1970-1984	80-85	Intellectual disability (inpatient)	0-4	NA	0-4	NA	0-4	NA
1985-1994	80-85	Intellectual disability (inpatient)	0-4	NA	0-4	NA	0-4	NA
1995-2004	80-85	Intellectual disability (inpatient)	13	0.11 (0.11-0.11)	5	0.12 (0.12-0.12)	8	0.11 (0.11-0.11)
2005-2016	80-85	Intellectual disability (inpatient)	6	0.04 (0.04-0.04)	0-4	NA	0-4	NA
1970-2016	85-90	Intellectual disability (inpatient)	7	0.03 (0.03-0.03)	0-4	NA	3-7	0.03 (0.03-0.03)
1970-1984	85-90	Intellectual disability (inpatient)	0-4	NA	0-4	NA	0-4	NA
1985-1994	85-90	Intellectual disability (inpatient)	0-4	NA	0-4	NA	0-4	NA
1995-2004	85-90	Intellectual disability (inpatient)	0-4	NA	0-4	NA	0-4	NA
2005-2016	85-90	Intellectual disability (inpatient)	5	0.06 (0.06-0.06)	0-4	NA	0-4	NA
1970-2016	90-95	Intellectual disability (inpatient)	0-4	NA	0-4	NA	0-4	NA
1970-1984	90-95	Intellectual disability (inpatient)	0-4	NA	0-4	NA	0-4	NA
1985-1994	90-95	Intellectual disability (inpatient)	0-4	NA	0-4	NA	0-4	NA
1995-2004	90-95	Intellectual disability (inpatient)	0-4	NA	0-4	NA	0-4	NA
2005-2016	90-95	Intellectual disability (inpatient)	0-4	NA	0-4	NA	0-4	NA
1970-2016	95-100	Intellectual disability (inpatient)	0-4	NA	0-4	NA	0-4	NA
1970-1984	95-100	Intellectual disability (inpatient)	0-4	NA	0-4	NA	0-4	NA
1985-1994	95-100	Intellectual disability (inpatient)	0-4	NA	0-4	NA	0-4	NA
1995-2004	95-100	Intellectual disability (inpatient)	0-4	NA	0-4	NA	0-4	NA
2005-2016	95-100	Intellectual disability (inpatient)	0-4	NA	0-4	NA	0-4	NA
1970-2016	1-5	Developmental disorders	4503	3.72 (3.71-3.72)	3566	5.75 (5.74-5.75)	937	1.58 (1.58-1.59)
1970-1984	1-5	Developmental disorders	68	0.17 (0.17-0.17)	42	0.20 (0.20-0.20)	26	0.13 (0.13-0.13)
1985-1994	1-5	Developmental disorders	117	0.51 (0.51-0.51)	87	0.74 (0.74-0.74)	30	0.27 (0.27-0.27)
1995-2004	1-5	Developmental disorders	1269	4.68 (4.68-4.69)	1007	7.25 (7.24-7.26)	262	1.98 (1.98-1.99)
2005-2016	1-5	Developmental disorders	3049	10.03 (10.02-10.04)	2430	15.61 (15.59-15.64)	619	4.18 (4.17-4.18)
1970-2016	5-10	Developmental disorders	10413	6.69 (6.69-6.70)	8638	10.86 (10.85-10.87)	1775	2.33 (2.33-2.34)
1970-1984	5-10	Developmental disorders	139	0.25 (0.25-0.25)	107	0.38 (0.37-0.38)	32	0.12 (0.12-0.12)
1985-1994	5-10	Developmental disorders	165	0.59 (0.59-0.59)	142	0.99 (0.99-0.99)	23	0.17 (0.17-0.17)
1995-2004	5-10	Developmental disorders	2326	7.08 (7.07-7.09)	2014	11.97 (11.95-11.99)	312	1.95 (1.94-1.95)

			Both sexes		Males		Females	
Years	Age	Mental disorder	Cases	IR (95% CI)	Cases	IR (95% CI)	Cases	IR (95% CI)
2005-2016	5-10	Developmental disorders	7783	19.95 (19.93-19.97)	6375	32.05 (32.00-32.09)	1408	7.36 (7.35-7.37)
1970-2016	10-15	Developmental disorders	9511	6.03 (6.03-6.04)	6827	8.48 (8.47-8.49)	2684	3.48 (3.48-3.48)
1970-1984	10-15	Developmental disorders	156	0.27 (0.27-0.27)	122	0.42 (0.42-0.42)	34	0.12 (0.12-0.12)
1985-1994	10-15	Developmental disorders	197	0.62 (0.62-0.63)	155	0.96 (0.96-0.96)	42	0.27 (0.27-0.27)
1995-2004	10-15	Developmental disorders	1368	4.64 (4.63-4.64)	1116	7.40 (7.38-7.41)	252	1.75 (1.75-1.75)
2005-2016	10-15	Developmental disorders	7790	19.66 (19.64-19.68)	5434	26.96 (26.92-27.00)	2356	12.10 (12.08-12.11)
1970-2016	15-20	Developmental disorders	5493	3.43 (3.43-3.43)	3492	4.26 (4.25-4.26)	2001	2.56 (2.56-2.56)
1970-1984	15-20	Developmental disorders	81	0.14 (0.14-0.14)	47	0.16 (0.16-0.16)	34	0.12 (0.12-0.12)
1985-1994	15-20	Developmental disorders	76	0.22 (0.22-0.22)	53	0.29 (0.29-0.29)	23	0.13 (0.13-0.13)
1995-2004	15-20	Developmental disorders	523	1.84 (1.84-1.84)	391	2.69 (2.69-2.70)	132	0.95 (0.95-0.95)
2005-2016	15-20	Developmental disorders	4813	12.23 (12.22-12.24)	3001	14.95 (14.93-14.97)	1812	9.40 (9.39-9.42)
1970-2016	20-25	Developmental disorders	1716	1.07 (1.06-1.07)	1156	1.39 (1.39-1.39)	560	0.72 (0.72-0.72)
1970-1984	20-25	Developmental disorders	30	0.05 (0.05-0.05)	19	0.07 (0.07-0.07)	11	0.04 (0.04-0.04)
1985-1994	20-25	Developmental disorders	34	0.09 (0.09-0.09)	23	0.12 (0.12-0.12)	11	0.06 (0.06-0.06)
1995-2004	20-25	Developmental disorders	170	0.55 (0.55-0.55)	131	0.82 (0.82-0.82)	39	0.26 (0.26-0.26)
2005-2016	20-25	Developmental disorders	1482	3.98 (3.97-3.98)	983	5.16 (5.15-5.16)	499	2.74 (2.74-2.75)
1970-2016	25-30	Developmental disorders	950	0.59 (0.59-0.59)	636	0.76 (0.76-0.76)	314	0.40 (0.40-0.40)
1970-1984	25-30	Developmental disorders	15	0.03 (0.03-0.03)	5	0.02 (0.02-0.02)	10	0.04 (0.04-0.04)
1985-1994	25-30	Developmental disorders	24	0.07 (0.06-0.07)	14	0.07 (0.07-0.07)	10	0.06 (0.06-0.06)
1995-2004	25-30	Developmental disorders	113	0.34 (0.33-0.34)	86	0.49 (0.49-0.49)	27	0.17 (0.17-0.17)
2005-2016	25-30	Developmental disorders	798	2.30 (2.30-2.31)	531	3.01 (3.00-3.01)	267	1.57 (1.57-1.58)
1970-2016	30-35	Developmental disorders	600	0.37 (0.37-0.37)	373	0.45 (0.45-0.45)	227	0.29 (0.29-0.29)
1970-1984	30-35	Developmental disorders	12	0.02 (0.02-0.02)	7	0.03 (0.03-0.03)	5	0.02 (0.02-0.02)
1985-1994	30-35	Developmental disorders	19	0.05 (0.05-0.05)	9	0.05 (0.05-0.05)	10	0.06 (0.06-0.06)
1995-2004	30-35	Developmental disorders	74	0.21 (0.20-0.21)	51	0.27 (0.27-0.27)	23	0.13 (0.13-0.13)
2005-2016	30-35	Developmental disorders	495	1.39 (1.39-1.39)	306	1.68 (1.68-1.69)	189	1.08 (1.08-1.08)
1970-2016	35-40	Developmental disorders	496	0.31 (0.31-0.31)	332	0.41 (0.41-0.41)	164	0.21 (0.21-0.21)
1970-1984	35-40	Developmental disorders	8	0.02 (0.02-0.02)	4-8	0.02 (0.02-0.02)	0-4	NA
1985-1994	35-40	Developmental disorders	26	0.07 (0.07-0.07)	14	0.08 (0.08-0.08)	12	0.07 (0.07-0.07)
1995-2004	35-40	Developmental disorders	77	0.21 (0.21-0.21)	58	0.31 (0.31-0.32)	19	0.11 (0.11-0.11)
2005-2016	35-40	Developmental disorders	385	0.99 (0.99-1.00)	255	1.29 (1.29-1.29)	130	0.69 (0.68-0.69)
1970-2016	40-45	Developmental disorders	344	0.22 (0.22-0.22)	215	0.27 (0.27-0.27)	129	0.17 (0.17-0.17)
1970-1984	40-45	Developmental disorders	0-4	NA	0-4	NA	0-4	NA
1985-1994	40-45	Developmental disorders	17	0.05 (0.05-0.05)	9	0.05 (0.05-0.05)	8	0.04 (0.04-0.04)
1995-2004	40-45	Developmental disorders	45	0.13 (0.13-0.13)	31	0.18 (0.18-0.18)	14	0.08 (0.08-0.08)
2005-2016	40-45	Developmental disorders	279	0.67 (0.67-0.67)	175	0.82 (0.82-0.83)	104	0.51 (0.51-0.51)
1970-2016	45-50	Developmental disorders	256	0.17 (0.17-0.17)	170	0.22 (0.22-0.22)	86	0.11 (0.11-0.11)
1970-1984	45-50	Developmental disorders	6	0.01 (0.01-0.01)	0-4	NA	2-6	0.02 (0.02-0.02)
1985-1994	45-50	Developmental disorders	10	0.03 (0.03-0.03)	5	0.03 (0.03-0.03)	5	0.03 (0.03-0.03)
1995-2004	45-50	Developmental disorders	36	0.11 (0.11-0.11)	24	0.14 (0.14-0.14)	12	0.07 (0.07-0.07)

			Both sexes		Males		Females	
**Years**	**Age**	**Mental disorder**	**Cases**	**IR (95% CI)**	**Cases**	**IR (95% CI)**	**Cases**	**IR (95% CI)**
2005-2016	45-50	Developmental disorders	204	0.49 (0.49-0.49)	140	0.66 (0.66-0.66)	64	0.31 (0.31-0.31)
1970-2016	50-55	Developmental disorders	156	0.11 (0.11-0.11)	113	0.16 (0.16-0.16)	43	0.06 (0.06-0.06)
1970-1984	50-55	Developmental disorders	0-4	NA	0-4	NA	0-4	NA
1985-1994	50-55	Developmental disorders	5	0.02 (0.02-0.02)	0-4	NA	0-4	NA
1995-2004	50-55	Developmental disorders	25	0.07 (0.07-0.07)	19	0.11 (0.11-0.11)	6	0.03 (0.03-0.03)
2005-2016	50-55	Developmental disorders	124	0.31 (0.31-0.31)	91	0.46 (0.46-0.46)	33	0.17 (0.17-0.17)
1970-2016	55-60	Developmental disorders	104	0.08 (0.08-0.08)	73	0.11 (0.11-0.11)	31	0.04 (0.04-0.04)
1970-1984	55-60	Developmental disorders	0-4	NA	0-4	NA	0-4	NA
1985-1994	55-60	Developmental disorders	0-4	NA	0-4	NA	0-4	NA
1995-2004	55-60	Developmental disorders	28	0.09 (0.09-0.09)	20	0.13 (0.13-0.13)	8	0.05 (0.05-0.05)
2005-2016	55-60	Developmental disorders	70	0.18 (0.18-0.18)	50	0.26 (0.26-0.26)	20	0.10 (0.10-0.10)
1970-2016	60-65	Developmental disorders	52	0.04 (0.04-0.04)	35	0.06 (0.06-0.06)	17	0.03 (0.03-0.03)
1970-1984	60-65	Developmental disorders	0-4	NA	0-4	NA	0-4	NA
1985-1994	60-65	Developmental disorders	0-4	NA	0-4	NA	0-4	NA
1995-2004	60-65	Developmental disorders	13	0.05 (0.05-0.05)	8	0.06 (0.06-0.06)	5	0.04 (0.04-0.04)
2005-2016	60-65	Developmental disorders	36	0.09 (0.09-0.09)	26	0.14 (0.14-0.14)	10	0.05 (0.05-0.05)
1970-2016	65-70	Developmental disorders	34	0.03 (0.03-0.03)	18	0.03 (0.03-0.03)	16	0.03 (0.03-0.03)
1970-1984	65-70	Developmental disorders	0-4	NA	0-4	NA	0-4	NA
1985-1994	65-70	Developmental disorders	0-4	NA	0-4	NA	0-4	NA
1995-2004	65-70	Developmental disorders	8	0.04 (0.04-0.04)	0-4	NA	0-4	NA
2005-2016	65-70	Developmental disorders	25	0.07 (0.07-0.07)	14	0.08 (0.08-0.08)	11	0.06 (0.06-0.06)
1970-2016	70-75	Developmental disorders	18	0.02 (0.02-0.02)	13	0.03 (0.03-0.03)	5	0.01 (0.01-0.01)
1970-1984	70-75	Developmental disorders	0-4	NA	0-4	NA	0-4	NA
1985-1994	70-75	Developmental disorders	0-4	NA	0-4	NA	0-4	NA
1995-2004	70-75	Developmental disorders	5	0.03 (0.03-0.03)	0-4	NA	0-4	NA
2005-2016	70-75	Developmental disorders	12	0.05 (0.04-0.05)	8-12	0.08 (0.08-0.08)	0-4	NA
1970-2016	75-80	Developmental disorders	12	0.02 (0.02-0.02)	8-12	0.03 (0.03-0.03)	0-4	NA
1970-1984	75-80	Developmental disorders	0-4	NA	0-4	NA	0-4	NA
1985-1994	75-80	Developmental disorders	0-4	NA	0-4	NA	0-4	NA
1995-2004	75-80	Developmental disorders	0-4	NA	0-4	NA	0-4	NA
2005-2016	75-80	Developmental disorders	11	0.06 (0.06-0.06)	7-11	0.10 (0.10-0.10)	0-4	NA
1970-2016	80-85	Developmental disorders	7	0.01 (0.01-0.01)	3-7	0.03 (0.03-0.03)	0-4	NA
1970-1984	80-85	Developmental disorders	0-4	NA	0-4	NA	0-4	NA
1985-1994	80-85	Developmental disorders	0-4	NA	0-4	NA	0-4	NA
1995-2004	80-85	Developmental disorders	0-4	NA	0-4	NA	0-4	NA
2005-2016	80-85	Developmental disorders	5	0.04 (0.04-0.04)	0-4	NA	0-4	NA
1970-2016	85-90	Developmental disorders	0-4	NA	0-4	NA	0-4	NA
1970-1984	85-90	Developmental disorders	0-4	NA	0-4	NA	0-4	NA
1985-1994	85-90	Developmental disorders	0-4	NA	0-4	NA	0-4	NA
1995-2004	85-90	Developmental disorders	0-4	NA	0-4	NA	0-4	NA

			Both sexes		Males		Females	
**Years**	**Age**	**Mental disorder**	**Cases**	**IR (95% CI)**	**Cases**	**IR (95% CI)**	**Cases**	**IR (95% CI)**
2005-2016	85-90	Developmental disorders	0-4	NA	0-4	NA	0-4	NA
1970-2016	90-95	Developmental disorders	0-4	NA	0-4	NA	0-4	NA
1970-1984	90-95	Developmental disorders	0-4	NA	0-4	NA	0-4	NA
1985-1994	90-95	Developmental disorders	0-4	NA	0-4	NA	0-4	NA
1995-2004	90-95	Developmental disorders	0-4	NA	0-4	NA	0-4	NA
2005-2016	90-95	Developmental disorders	0-4	NA	0-4	NA	0-4	NA
1970-2016	95-100	Developmental disorders	0-4	NA	0-4	NA	0-4	NA
1970-1984	95-100	Developmental disorders	0-4	NA	0-4	NA	0-4	NA
1985-1994	95-100	Developmental disorders	0-4	NA	0-4	NA	0-4	NA
1995-2004	95-100	Developmental disorders	0-4	NA	0-4	NA	0-4	NA
2005-2016	95-100	Developmental disorders	0-4	NA	0-4	NA	0-4	NA
1970-2016	1-5	Developmental disorders (inpatient)	374	0.31 (0.31-0.31)	280	0.45 (0.45-0.45)	94	0.16 (0.16-0.16)
1970-1984	1-5	Developmental disorders (inpatient)	67	0.16 (0.16-0.16)	41	0.20 (0.20-0.20)	26	0.13 (0.13-0.13)
1985-1994	1-5	Developmental disorders (inpatient)	87	0.38 (0.38-0.38)	62	0.53 (0.53-0.53)	25	0.22 (0.22-0.22)
1995-2004	1-5	Developmental disorders (inpatient)	179	0.66 (0.66-0.66)	144	1.04 (1.03-1.04)	35	0.26 (0.26-0.27)
2005-2016	1-5	Developmental disorders (inpatient)	41	0.13 (0.13-0.13)	33	0.21 (0.21-0.21)	8	0.05 (0.05-0.05)
1970-2016	5-10	Developmental disorders (inpatient)	809	0.52 (0.52-0.52)	672	0.84 (0.84-0.84)	137	0.18 (0.18-0.18)
1970-1984	5-10	Developmental disorders (inpatient)	139	0.25 (0.25-0.25)	107	0.38 (0.37-0.38)	32	0.12 (0.12-0.12)
1985-1994	5-10	Developmental disorders (inpatient)	136	0.49 (0.49-0.49)	119	0.83 (0.83-0.83)	17	0.12 (0.12-0.12)
1995-2004	5-10	Developmental disorders (inpatient)	353	1.07 (1.07-1.07)	307	1.82 (1.82-1.82)	46	0.29 (0.29-0.29)
2005-2016	5-10	Developmental disorders (inpatient)	181	0.46 (0.46-0.46)	139	0.69 (0.69-0.69)	42	0.22 (0.22-0.22)
1970-2016	10-15	Developmental disorders (inpatient)	1386	0.88 (0.88-0.88)	926	1.14 (1.14-1.14)	460	0.60 (0.60-0.60)
1970-1984	10-15	Developmental disorders (inpatient)	156	0.27 (0.27-0.27)	122	0.42 (0.42-0.42)	34	0.12 (0.12-0.12)
1985-1994	10-15	Developmental disorders (inpatient)	168	0.53 (0.53-0.53)	131	0.81 (0.81-0.81)	37	0.24 (0.24-0.24)
1995-2004	10-15	Developmental disorders (inpatient)	241	0.82 (0.81-0.82)	190	1.25 (1.25-1.26)	51	0.35 (0.35-0.35)
2005-2016	10-15	Developmental disorders (inpatient)	821	2.05 (2.05-2.05)	483	2.35 (2.35-2.35)	338	1.73 (1.73-1.73)
1970-2016	15-20	Developmental disorders (inpatient)	1206	0.75 (0.75-0.75)	693	0.84 (0.84-0.84)	513	0.66 (0.66-0.66)
1970-1984	15-20	Developmental disorders (inpatient)	81	0.14 (0.14-0.14)	47	0.16 (0.16-0.16)	34	0.12 (0.12-0.12)
1985-1994	15-20	Developmental disorders (inpatient)	60	0.17 (0.17-0.17)	39	0.22 (0.22-0.22)	21	0.12 (0.12-0.12)
1995-2004	15-20	Developmental disorders (inpatient)	138	0.49 (0.48-0.49)	103	0.71 (0.71-0.71)	35	0.25 (0.25-0.25)
2005-2016	15-20	Developmental disorders (inpatient)	927	2.33 (2.33-2.33)	504	2.47 (2.46-2.47)	423	2.18 (2.18-2.19)
1970-2016	20-25	Developmental disorders (inpatient)	510	0.32 (0.32-0.32)	361	0.43 (0.43-0.43)	149	0.19 (0.19-0.19)
1970-1984	20-25	Developmental disorders (inpatient)	30	0.05 (0.05-0.05)	19	0.07 (0.07-0.07)	11	0.04 (0.04-0.04)
1985-1994	20-25	Developmental disorders (inpatient)	18	0.05 (0.05-0.05)	11	0.06 (0.06-0.06)	7	0.04 (0.04-0.04)
1995-2004	20-25	Developmental disorders (inpatient)	66	0.21 (0.21-0.21)	57	0.36 (0.36-0.36)	9	0.06 (0.06-0.06)
2005-2016	20-25	Developmental disorders (inpatient)	396	1.06 (1.05-1.06)	274	1.42 (1.42-1.43)	122	0.67 (0.67-0.67)
1970-2016	25-30	Developmental disorders (inpatient)	235	0.14 (0.14-0.15)	153	0.18 (0.18-0.18)	82	0.10 (0.10-0.10)
1970-1984	25-30	Developmental disorders (inpatient)	15	0.03 (0.03-0.03)	5	0.02 (0.02-0.02)	10	0.04 (0.04-0.04)
1985-1994	25-30	Developmental disorders (inpatient)	10	0.03 (0.03-0.03)	5	0.03 (0.03-0.03)	5	0.03 (0.03-0.03)
1995-2004	25-30	Developmental disorders (inpatient)	39	0.12 (0.12-0.12)	31	0.18 (0.18-0.18)	8	0.05 (0.05-0.05)

			Both sexes		Males		Females	
Years	Age	Mental disorder	Cases	IR (95% CI)	Cases	IR (95% CI)	Cases	IR (95% CI)
2005-2016	25-30	Developmental disorders (inpatient)	171	0.49 (0.49-0.49)	112	0.63 (0.63-0.63)	59	0.35 (0.35-0.35)
1970-2016	30-35	Developmental disorders (inpatient)	153	0.10 (0.10-0.10)	105	0.13 (0.13-0.13)	48	0.06 (0.06-0.06)
1970-1984	30-35	Developmental disorders (inpatient)	12	0.02 (0.02-0.02)	7	0.03 (0.03-0.03)	5	0.02 (0.02-0.02)
1985-1994	30-35	Developmental disorders (inpatient)	6	0.02 (0.02-0.02)	0-4	NA	2-6	0.03 (0.03-0.03)
1995-2004	30-35	Developmental disorders (inpatient)	19	0.05 (0.05-0.05)	15-19	0.08 (0.08-0.08)	0-4	NA
2005-2016	30-35	Developmental disorders (inpatient)	116	0.32 (0.32-0.33)	82	0.45 (0.45-0.45)	34	0.19 (0.19-0.19)
1970-2016	35-40	Developmental disorders (inpatient)	117	0.07 (0.07-0.07)	82	0.10 (0.10-0.10)	35	0.04 (0.04-0.04)
1970-1984	35-40	Developmental disorders (inpatient)	8	0.02 (0.02-0.02)	4-8	0.02 (0.02-0.02)	0-4	NA
1985-1994	35-40	Developmental disorders (inpatient)	8	0.02 (0.02-0.02)	4-8	0.03 (0.03-0.03)	0-4	NA
1995-2004	35-40	Developmental disorders (inpatient)	19	0.05 (0.05-0.05)	14	0.08 (0.08-0.08)	5	0.03 (0.03-0.03)
2005-2016	35-40	Developmental disorders (inpatient)	82	0.21 (0.21-0.21)	58	0.29 (0.29-0.29)	24	0.13 (0.13-0.13)
1970-2016	40-45	Developmental disorders (inpatient)	96	0.06 (0.06-0.06)	60	0.08 (0.08-0.08)	36	0.05 (0.05-0.05)
1970-1984	40-45	Developmental disorders (inpatient)	0-4	NA	0-4	NA	0-4	NA
1985-1994	40-45	Developmental disorders (inpatient)	0-4	NA	0-4	NA	0-4	NA
1995-2004	40-45	Developmental disorders (inpatient)	17	0.05 (0.05-0.05)	11	0.06 (0.06-0.06)	6	0.04 (0.04-0.04)
2005-2016	40-45	Developmental disorders (inpatient)	72	0.17 (0.17-0.17)	47	0.22 (0.22-0.22)	25	0.12 (0.12-0.12)
1970-2016	45-50	Developmental disorders (inpatient)	64	0.04 (0.04-0.04)	45	0.06 (0.06-0.06)	19	0.03 (0.03-0.03)
1970-1984	45-50	Developmental disorders (inpatient)	6	0.01 (0.01-0.01)	0-4	NA	2-6	0.02 (0.02-0.02)
1985-1994	45-50	Developmental disorders (inpatient)	0-4	NA	0-4	NA	0-4	NA
1995-2004	45-50	Developmental disorders (inpatient)	7	0.02 (0.02-0.02)	3-7	0.04 (0.03-0.04)	0-4	NA
2005-2016	45-50	Developmental disorders (inpatient)	48	0.11 (0.11-0.11)	36	0.17 (0.17-0.17)	12	0.06 (0.06-0.06)
1970-2016	50-55	Developmental disorders (inpatient)	32	0.02 (0.02-0.02)	25	0.03 (0.03-0.03)	7	0.01 (0.01-0.01)
1970-1984	50-55	Developmental disorders (inpatient)	0-4	NA	0-4	NA	0-4	NA
1985-1994	50-55	Developmental disorders (inpatient)	0-4	NA	0-4	NA	0-4	NA
1995-2004	50-55	Developmental disorders (inpatient)	0-4	NA	0-4	NA	0-4	NA
2005-2016	50-55	Developmental disorders (inpatient)	27	0.07 (0.07-0.07)	22	0.11 (0.11-0.11)	5	0.03 (0.03-0.03)
1970-2016	55-60	Developmental disorders (inpatient)	31	0.02 (0.02-0.02)	22	0.03 (0.03-0.03)	9	0.01 (0.01-0.01)
1970-1984	55-60	Developmental disorders (inpatient)	0-4	NA	0-4	NA	0-4	NA
1985-1994	55-60	Developmental disorders (inpatient)	0-4	NA	0-4	NA	0-4	NA
1995-2004	55-60	Developmental disorders (inpatient)	7	0.02 (0.02-0.02)	3-7	0.03 (0.03-0.03)	0-4	NA
2005-2016	55-60	Developmental disorders (inpatient)	20	0.05 (0.05-0.05)	16-20	0.08 (0.08-0.08)	0-4	NA
1970-2016	60-65	Developmental disorders (inpatient)	14	0.01 (0.01-0.01)	7	0.01 (0.01-0.01)	7	0.01 (0.01-0.01)
1970-1984	60-65	Developmental disorders (inpatient)	0-4	NA	0-4	NA	0-4	NA
1985-1994	60-65	Developmental disorders (inpatient)	0-4	NA	0-4	NA	0-4	NA
1995-2004	60-65	Developmental disorders (inpatient)	0-4	NA	0-4	NA	0-4	NA
2005-2016	60-65	Developmental disorders (inpatient)	7	0.02 (0.02-0.02)	0-4	NA	0-4	NA
1970-2016	65-70	Developmental disorders (inpatient)	14	0.01 (0.01-0.01)	8	0.01 (0.01-0.01)	6	0.01 (0.01-0.01)
1970-1984	65-70	Developmental disorders (inpatient)	0-4	NA	0-4	NA	0-4	NA
1985-1994	65-70	Developmental disorders (inpatient)	0-4	NA	0-4	NA	0-4	NA
1995-2004	65-70	Developmental disorders (inpatient)	0-4	NA	0-4	NA	0-4	NA

			Both sexes		Males		Females	
**Years**	**Age**	**Mental disorder**	**Cases**	**IR (95% CI)**	**Cases**	**IR (95% CI)**	**Cases**	**IR (95% CI)**
2005-2016	65-70	Developmental disorders (inpatient)	9	0.03 (0.03-0.03)	5-9	0.04 (0.04-0.04)	0-4	NA
1970-2016	70-75	Developmental disorders (inpatient)	7	0.01 (0.01-0.01)	3-7	0.01 (0.01-0.01)	0-4	NA
1970-1984	70-75	Developmental disorders (inpatient)	0-4	NA	0-4	NA	0-4	NA
1985-1994	70-75	Developmental disorders (inpatient)	0-4	NA	0-4	NA	0-4	NA
1995-2004	70-75	Developmental disorders (inpatient)	0-4	NA	0-4	NA	0-4	NA
2005-2016	70-75	Developmental disorders (inpatient)	5	0.02 (0.02-0.02)	0-4	NA	0-4	NA
1970-2016	75-80	Developmental disorders (inpatient)	0-4	NA	0-4	NA	0-4	NA
1970-1984	75-80	Developmental disorders (inpatient)	0-4	NA	0-4	NA	0-4	NA
1985-1994	75-80	Developmental disorders (inpatient)	0-4	NA	0-4	NA	0-4	NA
1995-2004	75-80	Developmental disorders (inpatient)	0-4	NA	0-4	NA	0-4	NA
2005-2016	75-80	Developmental disorders (inpatient)	0-4	NA	0-4	NA	0-4	NA
1970-2016	80-85	Developmental disorders (inpatient)	0-4	NA	0-4	NA	0-4	NA
1970-1984	80-85	Developmental disorders (inpatient)	0-4	NA	0-4	NA	0-4	NA
1985-1994	80-85	Developmental disorders (inpatient)	0-4	NA	0-4	NA	0-4	NA
1995-2004	80-85	Developmental disorders (inpatient)	0-4	NA	0-4	NA	0-4	NA
2005-2016	80-85	Developmental disorders (inpatient)	0-4	NA	0-4	NA	0-4	NA
1970-2016	85-90	Developmental disorders (inpatient)	0-4	NA	0-4	NA	0-4	NA
1970-1984	85-90	Developmental disorders (inpatient)	0-4	NA	0-4	NA	0-4	NA
1985-1994	85-90	Developmental disorders (inpatient)	0-4	NA	0-4	NA	0-4	NA
1995-2004	85-90	Developmental disorders (inpatient)	0-4	NA	0-4	NA	0-4	NA
2005-2016	85-90	Developmental disorders (inpatient)	0-4	NA	0-4	NA	0-4	NA
1970-2016	90-95	Developmental disorders (inpatient)	0-4	NA	0-4	NA	0-4	NA
1970-1984	90-95	Developmental disorders (inpatient)	0-4	NA	0-4	NA	0-4	NA
1985-1994	90-95	Developmental disorders (inpatient)	0-4	NA	0-4	NA	0-4	NA
1995-2004	90-95	Developmental disorders (inpatient)	0-4	NA	0-4	NA	0-4	NA
2005-2016	90-95	Developmental disorders (inpatient)	0-4	NA	0-4	NA	0-4	NA
1970-2016	95-100	Developmental disorders (inpatient)	0-4	NA	0-4	NA	0-4	NA
1970-1984	95-100	Developmental disorders (inpatient)	0-4	NA	0-4	NA	0-4	NA
1985-1994	95-100	Developmental disorders (inpatient)	0-4	NA	0-4	NA	0-4	NA
1995-2004	95-100	Developmental disorders (inpatient)	0-4	NA	0-4	NA	0-4	NA
2005-2016	95-100	Developmental disorders (inpatient)	0-4	NA	0-4	NA	0-4	NA
1970-2016	1-5	Childhood autism	2771	2.29 (2.29-2.29)	2242	3.61 (3.61-3.61)	529	0.89 (0.89-0.90)
1970-1984	1-5	Childhood autism	44	0.11 (0.11-0.11)	29	0.14 (0.14-0.14)	15	0.08 (0.08-0.08)
1985-1994	1-5	Childhood autism	81	0.35 (0.35-0.35)	62	0.53 (0.53-0.53)	19	0.17 (0.17-0.17)
1995-2004	1-5	Childhood autism	705	2.60 (2.60-2.60)	572	4.12 (4.11-4.12)	133	1.01 (1.00-1.01)
2005-2016	1-5	Childhood autism	1941	6.38 (6.38-6.39)	1579	10.14 (10.12-10.15)	362	2.44 (2.44-2.45)
1970-2016	5-10	Childhood autism	3471	2.23 (2.23-2.23)	2839	3.56 (3.56-3.56)	632	0.83 (0.83-0.83)
1970-1984	5-10	Childhood autism	41	0.07 (0.07-0.07)	27	0.09 (0.09-0.09)	14	0.05 (0.05-0.05)
1985-1994	5-10	Childhood autism	40	0.14 (0.14-0.14)	32	0.22 (0.22-0.22)	8	0.06 (0.06-0.06)
1995-2004	5-10	Childhood autism	516	1.57 (1.57-1.57)	428	2.54 (2.53-2.54)	88	0.55 (0.55-0.55)

			Both sexes		Males		Females	
**Years**	**Age**	**Mental disorder**	**Cases**	**IR (95% CI)**	**Cases**	**IR (95% CI)**	**Cases**	**IR (95% CI)**
2005-2016	5-10	Childhood autism	2874	7.34 (7.33-7.34)	2352	11.75 (11.73-11.77)	522	2.73 (2.72-2.73)
1970-2016	10-15	Childhood autism	1969	1.25 (1.25-1.25)	1501	1.86 (1.86-1.86)	468	0.61 (0.61-0.61)
1970-1984	10-15	Childhood autism	24	0.04 (0.04-0.04)	18	0.06 (0.06-0.06)	6	0.02 (0.02-0.02)
1985-1994	10-15	Childhood autism	11	0.03 (0.03-0.03)	7-11	0.06 (0.06-0.06)	0-4	NA
1995-2004	10-15	Childhood autism	161	0.54 (0.54-0.55)	118	0.78 (0.78-0.78)	43	0.30 (0.30-0.30)
2005-2016	10-15	Childhood autism	1773	4.44 (4.43-4.44)	1356	6.64 (6.63-6.65)	417	2.13 (2.13-2.14)
1970-2016	15-20	Childhood autism	1076	0.67 (0.67-0.67)	788	0.96 (0.96-0.96)	288	0.37 (0.37-0.37)
1970-1984	15-20	Childhood autism	10	0.02 (0.02-0.02)	6-10	0.03 (0.03-0.03)	0-4	NA
1985-1994	15-20	Childhood autism	16	0.05 (0.05-0.05)	11	0.06 (0.06-0.06)	5	0.03 (0.03-0.03)
1995-2004	15-20	Childhood autism	89	0.31 (0.31-0.31)	67	0.46 (0.46-0.46)	22	0.16 (0.16-0.16)
2005-2016	15-20	Childhood autism	961	2.42 (2.41-2.42)	702	3.44 (3.44-3.45)	259	1.34 (1.33-1.34)
1970-2016	20-25	Childhood autism	240	0.15 (0.15-0.15)	176	0.21 (0.21-0.21)	64	0.08 (0.08-0.08)
1970-1984	20-25	Childhood autism	7	0.01 (0.01-0.01)	0-4	NA	0-4	NA
1985-1994	20-25	Childhood autism	17	0.05 (0.05-0.05)	12	0.06 (0.06-0.06)	5	0.03 (0.03-0.03)
1995-2004	20-25	Childhood autism	44	0.14 (0.14-0.14)	34	0.21 (0.21-0.21)	10	0.07 (0.07-0.07)
2005-2016	20-25	Childhood autism	172	0.46 (0.46-0.46)	126	0.65 (0.65-0.66)	46	0.25 (0.25-0.25)
1970-2016	25-30	Childhood autism	122	0.08 (0.08-0.08)	86	0.10 (0.10-0.10)	36	0.05 (0.05-0.05)
1970-1984	25-30	Childhood autism	0-4	NA	0-4	NA	0-4	NA
1985-1994	25-30	Childhood autism	10	0.03 (0.03-0.03)	6-10	0.03 (0.03-0.03)	0-4	NA
1995-2004	25-30	Childhood autism	25	0.07 (0.07-0.07)	16	0.09 (0.09-0.09)	9	0.06 (0.06-0.06)
2005-2016	25-30	Childhood autism	85	0.24 (0.24-0.24)	63	0.35 (0.35-0.36)	22	0.13 (0.13-0.13)
1970-2016	30-35	Childhood autism	76	0.05 (0.05-0.05)	44	0.05 (0.05-0.05)	32	0.04 (0.04-0.04)
1970-1984	30-35	Childhood autism	0-4	NA	0-4	NA	0-4	NA
1985-1994	30-35	Childhood autism	9	0.03 (0.03-0.03)	5-9	0.03 (0.03-0.03)	0-4	NA
1995-2004	30-35	Childhood autism	17	0.05 (0.05-0.05)	8	0.04 (0.04-0.04)	9	0.05 (0.05-0.05)
2005-2016	30-35	Childhood autism	48	0.13 (0.13-0.13)	30	0.16 (0.16-0.16)	18	0.10 (0.10-0.10)
1970-2016	35-40	Childhood autism	73	0.05 (0.05-0.05)	54	0.07 (0.07-0.07)	19	0.02 (0.02-0.02)
1970-1984	35-40	Childhood autism	0-4	NA	0-4	NA	0-4	NA
1985-1994	35-40	Childhood autism	13	0.04 (0.04-0.04)	8	0.04 (0.04-0.04)	5	0.03 (0.03-0.03)
1995-2004	35-40	Childhood autism	14	0.04 (0.04-0.04)	10-14	0.06 (0.06-0.06)	0-4	NA
2005-2016	35-40	Childhood autism	44	0.11 (0.11-0.11)	33	0.17 (0.17-0.17)	11	0.06 (0.06-0.06)
1970-2016	40-45	Childhood autism	58	0.04 (0.04-0.04)	42	0.05 (0.05-0.05)	16	0.02 (0.02-0.02)
1970-1984	40-45	Childhood autism	0-4	NA	0-4	NA	0-4	NA
1985-1994	40-45	Childhood autism	5	0.01 (0.01-0.01)	1-5	0.03 (0.03-0.03)	0-4	NA
1995-2004	40-45	Childhood autism	6	0.02 (0.02-0.02)	0-4	NA	0-4	NA
2005-2016	40-45	Childhood autism	47	0.11 (0.11-0.11)	33	0.16 (0.16-0.16)	14	0.07 (0.07-0.07)
1970-2016	45-50	Childhood autism	43	0.03 (0.03-0.03)	28	0.04 (0.04-0.04)	15	0.02 (0.02-0.02)
1970-1984	45-50	Childhood autism	0-4	NA	0-4	NA	0-4	NA
1985-1994	45-50	Childhood autism	0-4	NA	0-4	NA	0-4	NA
1995-2004	45-50	Childhood autism	5	0.01 (0.01-0.01)	0-4	NA	0-4	NA

			Both sexes		Males		Females	
**Years**	**Age**	**Mental disorder**	**Cases**	**IR (95% CI)**	**Cases**	**IR (95% CI)**	**Cases**	**IR (95% CI)**
2005-2016	45-50	Childhood autism	33	0.08 (0.08-0.08)	24	0.11 (0.11-0.11)	9	0.04 (0.04-0.04)
1970-2016	50-55	Childhood autism	38	0.03 (0.03-0.03)	27	0.04 (0.04-0.04)	11	0.02 (0.02-0.02)
1970-1984	50-55	Childhood autism	0-4	NA	0-4	NA	0-4	NA
1985-1994	50-55	Childhood autism	0-4	NA	0-4	NA	0-4	NA
1995-2004	50-55	Childhood autism	8	0.02 (0.02-0.02)	4-8	0.04 (0.04-0.04)	0-4	NA
2005-2016	50-55	Childhood autism	28	0.07 (0.07-0.07)	20	0.10 (0.10-0.10)	8	0.04 (0.04-0.04)
1970-2016	55-60	Childhood autism	26	0.02 (0.02-0.02)	20	0.03 (0.03-0.03)	6	0.01 (0.01-0.01)
1970-1984	55-60	Childhood autism	0-4	NA	0-4	NA	0-4	NA
1985-1994	55-60	Childhood autism	0-4	NA	0-4	NA	0-4	NA
1995-2004	55-60	Childhood autism	6	0.02 (0.02-0.02)	0-4	NA	0-4	NA
2005-2016	55-60	Childhood autism	19	0.05 (0.05-0.05)	15-19	0.08 (0.08-0.08)	0-4	NA
1970-2016	60-65	Childhood autism	22	0.02 (0.02-0.02)	15	0.02 (0.02-0.02)	7	0.01 (0.01-0.01)
1970-1984	60-65	Childhood autism	0-4	NA	0-4	NA	0-4	NA
1985-1994	60-65	Childhood autism	0-4	NA	0-4	NA	0-4	NA
1995-2004	60-65	Childhood autism	0-4	NA	0-4	NA	0-4	NA
2005-2016	60-65	Childhood autism	18	0.05 (0.05-0.05)	12	0.06 (0.06-0.06)	6	0.03 (0.03-0.03)
1970-2016	65-70	Childhood autism	9	0.01 (0.01-0.01)	5-9	0.01 (0.01-0.01)	0-4	NA
1970-1984	65-70	Childhood autism	0-4	NA	0-4	NA	0-4	NA
1985-1994	65-70	Childhood autism	0-4	NA	0-4	NA	0-4	NA
1995-2004	65-70	Childhood autism	0-4	NA	0-4	NA	0-4	NA
2005-2016	65-70	Childhood autism	6	0.02 (0.02-0.02)	0-4	NA	0-4	NA
1970-2016	70-75	Childhood autism	7	0.01 (0.01-0.01)	3-7	0.01 (0.01-0.01)	0-4	NA
1970-1984	70-75	Childhood autism	0-4	NA	0-4	NA	0-4	NA
1985-1994	70-75	Childhood autism	0-4	NA	0-4	NA	0-4	NA
1995-2004	70-75	Childhood autism	0-4	NA	0-4	NA	0-4	NA
2005-2016	70-75	Childhood autism	5	0.02 (0.02-0.02)	0-4	NA	0-4	NA
1970-2016	75-80	Childhood autism	0-4	NA	0-4	NA	0-4	NA
1970-1984	75-80	Childhood autism	0-4	NA	0-4	NA	0-4	NA
1985-1994	75-80	Childhood autism	0-4	NA	0-4	NA	0-4	NA
1995-2004	75-80	Childhood autism	0-4	NA	0-4	NA	0-4	NA
2005-2016	75-80	Childhood autism	0-4	NA	0-4	NA	0-4	NA
1970-2016	80-85	Childhood autism	0-4	NA	0-4	NA	0-4	NA
1970-1984	80-85	Childhood autism	0-4	NA	0-4	NA	0-4	NA
1985-1994	80-85	Childhood autism	0-4	NA	0-4	NA	0-4	NA
1995-2004	80-85	Childhood autism	0-4	NA	0-4	NA	0-4	NA
2005-2016	80-85	Childhood autism	0-4	NA	0-4	NA	0-4	NA
1970-2016	85-90	Childhood autism	0-4	NA	0-4	NA	0-4	NA
1970-1984	85-90	Childhood autism	0-4	NA	0-4	NA	0-4	NA
1985-1994	85-90	Childhood autism	0-4	NA	0-4	NA	0-4	NA
1995-2004	85-90	Childhood autism	0-4	NA	0-4	NA	0-4	NA

			Both sexes		Males		Females	
Years	Age	Mental disorder	Cases	IR (95% CI)	Cases	IR (95% CI)	Cases	IR (95% CI)
2005-2016	85-90	Childhood autism	0-4	NA	0-4	NA	0-4	NA
1970-2016	90-95	Childhood autism	0-4	NA	0-4	NA	0-4	NA
1970-1984	90-95	Childhood autism	0-4	NA	0-4	NA	0-4	NA
1985-1994	90-95	Childhood autism	0-4	NA	0-4	NA	0-4	NA
1995-2004	90-95	Childhood autism	0-4	NA	0-4	NA	0-4	NA
2005-2016	90-95	Childhood autism	0-4	NA	0-4	NA	0-4	NA
1970-2016	95-100	Childhood autism	0-4	NA	0-4	NA	0-4	NA
1970-1984	95-100	Childhood autism	0-4	NA	0-4	NA	0-4	NA
1985-1994	95-100	Childhood autism	0-4	NA	0-4	NA	0-4	NA
1995-2004	95-100	Childhood autism	0-4	NA	0-4	NA	0-4	NA
2005-2016	95-100	Childhood autism	0-4	NA	0-4	NA	0-4	NA
1970-2016	1-5	Childhood autism (inpatient)	233	0.19 (0.19-0.19)	175	0.28 (0.28-0.28)	58	0.10 (0.10-0.10)
1970-1984	1-5	Childhood autism (inpatient)	43	0.11 (0.11-0.11)	28	0.13 (0.13-0.13)	15	0.08 (0.08-0.08)
1985-1994	1-5	Childhood autism (inpatient)	60	0.26 (0.26-0.26)	44	0.37 (0.37-0.37)	16	0.14 (0.14-0.14)
1995-2004	1-5	Childhood autism (inpatient)	102	0.38 (0.38-0.38)	81	0.58 (0.58-0.58)	21	0.16 (0.16-0.16)
2005-2016	1-5	Childhood autism (inpatient)	28	0.09 (0.09-0.09)	22	0.14 (0.14-0.14)	6	0.04 (0.04-0.04)
1970-2016	5-10	Childhood autism (inpatient)	219	0.14 (0.14-0.14)	174	0.22 (0.22-0.22)	45	0.06 (0.06-0.06)
1970-1984	5-10	Childhood autism (inpatient)	41	0.07 (0.07-0.07)	27	0.09 (0.09-0.09)	14	0.05 (0.05-0.05)
1985-1994	5-10	Childhood autism (inpatient)	28	0.10 (0.10-0.10)	24-28	0.17 (0.17-0.17)	0-4	NA
1995-2004	5-10	Childhood autism (inpatient)	84	0.26 (0.25-0.26)	70	0.41 (0.41-0.42)	14	0.09 (0.09-0.09)
2005-2016	5-10	Childhood autism (inpatient)	66	0.17 (0.17-0.17)	52	0.26 (0.26-0.26)	14	0.07 (0.07-0.07)
1970-2016	10-15	Childhood autism (inpatient)	210	0.13 (0.13-0.13)	149	0.18 (0.18-0.18)	61	0.08 (0.08-0.08)
1970-1984	10-15	Childhood autism (inpatient)	24	0.04 (0.04-0.04)	18	0.06 (0.06-0.06)	6	0.02 (0.02-0.02)
1985-1994	10-15	Childhood autism (inpatient)	5	0.02 (0.02-0.02)	1-5	0.03 (0.03-0.03)	0-4	NA
1995-2004	10-15	Childhood autism (inpatient)	18	0.06 (0.06-0.06)	14-18	0.09 (0.09-0.09)	0-4	NA
2005-2016	10-15	Childhood autism (inpatient)	163	0.41 (0.41-0.41)	112	0.54 (0.54-0.55)	51	0.26 (0.26-0.26)
1970-2016	15-20	Childhood autism (inpatient)	166	0.10 (0.10-0.10)	112	0.14 (0.14-0.14)	54	0.07 (0.07-0.07)
1970-1984	15-20	Childhood autism (inpatient)	10	0.02 (0.02-0.02)	6-10	0.03 (0.03-0.03)	0-4	NA
1985-1994	15-20	Childhood autism (inpatient)	12	0.03 (0.03-0.03)	7	0.04 (0.04-0.04)	5	0.03 (0.03-0.03)
1995-2004	15-20	Childhood autism (inpatient)	15	0.05 (0.05-0.05)	11-15	0.08 (0.08-0.08)	0-4	NA
2005-2016	15-20	Childhood autism (inpatient)	129	0.32 (0.32-0.32)	86	0.42 (0.42-0.42)	43	0.22 (0.22-0.22)
1970-2016	20-25	Childhood autism (inpatient)	72	0.04 (0.04-0.04)	52	0.06 (0.06-0.06)	20	0.03 (0.03-0.03)
1970-1984	20-25	Childhood autism (inpatient)	7	0.01 (0.01-0.01)	0-4	NA	0-4	NA
1985-1994	20-25	Childhood autism (inpatient)	7	0.02 (0.02-0.02)	0-4	NA	0-4	NA
1995-2004	20-25	Childhood autism (inpatient)	10	0.03 (0.03-0.03)	6-10	0.06 (0.06-0.06)	0-4	NA
2005-2016	20-25	Childhood autism (inpatient)	48	0.13 (0.13-0.13)	35	0.18 (0.18-0.18)	13	0.07 (0.07-0.07)
1970-2016	25-30	Childhood autism (inpatient)	35	0.02 (0.02-0.02)	25	0.03 (0.03-0.03)	10	0.01 (0.01-0.01)
1970-1984	25-30	Childhood autism (inpatient)	0-4	NA	0-4	NA	0-4	NA
1985-1994	25-30	Childhood autism (inpatient)	0-4	NA	0-4	NA	0-4	NA
1995-2004	25-30	Childhood autism (inpatient)	9	0.03 (0.03-0.03)	5-9	0.03 (0.03-0.03)	0-4	NA

			Both sexes		Males		Females	
**Years**	**Age**	**Mental disorder**	**Cases**	**IR (95% CI)**	**Cases**	**IR (95% CI)**	**Cases**	**IR (95% CI)**
2005-2016	25-30	Childhood autism (inpatient)	20	0.06 (0.06-0.06)	16-20	0.09 (0.09-0.09)	0-4	NA
1970-2016	30-35	Childhood autism (inpatient)	24	0.01 (0.01-0.01)	17	0.02 (0.02-0.02)	7	0.01 (0.01-0.01)
1970-1984	30-35	Childhood autism (inpatient)	0-4	NA	0-4	NA	0-4	NA
1985-1994	30-35	Childhood autism (inpatient)	5	0.01 (0.01-0.01)	0-4	NA	0-4	NA
1995-2004	30-35	Childhood autism (inpatient)	6	0.02 (0.02-0.02)	2-6	0.03 (0.03-0.03)	0-4	NA
2005-2016	30-35	Childhood autism (inpatient)	11	0.03 (0.03-0.03)	7-11	0.04 (0.04-0.04)	0-4	NA
1970-2016	35-40	Childhood autism (inpatient)	19	0.01 (0.01-0.01)	12	0.01 (0.01-0.01)	7	0.01 (0.01-0.01)
1970-1984	35-40	Childhood autism (inpatient)	0-4	NA	0-4	NA	0-4	NA
1985-1994	35-40	Childhood autism (inpatient)	0-4	NA	0-4	NA	0-4	NA
1995-2004	35-40	Childhood autism (inpatient)	0-4	NA	0-4	NA	0-4	NA
2005-2016	35-40	Childhood autism (inpatient)	10	0.03 (0.03-0.03)	6-10	0.04 (0.04-0.04)	0-4	NA
1970-2016	40-45	Childhood autism (inpatient)	9	0.01 (0.01-0.01)	0-4	NA	5-9	0.01 (0.01-0.01)
1970-1984	40-45	Childhood autism (inpatient)	0-4	NA	0-4	NA	0-4	NA
1985-1994	40-45	Childhood autism (inpatient)	0-4	NA	0-4	NA	0-4	NA
1995-2004	40-45	Childhood autism (inpatient)	0-4	NA	0-4	NA	0-4	NA
2005-2016	40-45	Childhood autism (inpatient)	6	0.01 (0.01-0.01)	0-4	NA	0-4	NA
1970-2016	45-50	Childhood autism (inpatient)	8	0.01 (0.01-0.01)	4-8	0.01 (0.01-0.01)	0-4	NA
1970-1984	45-50	Childhood autism (inpatient)	0-4	NA	0-4	NA	0-4	NA
1985-1994	45-50	Childhood autism (inpatient)	0-4	NA	0-4	NA	0-4	NA
1995-2004	45-50	Childhood autism (inpatient)	0-4	NA	0-4	NA	0-4	NA
2005-2016	45-50	Childhood autism (inpatient)	0-4	NA	0-4	NA	0-4	NA
1970-2016	50-55	Childhood autism (inpatient)	5	0.00 (0.00-0.00)	0-4	NA	0-4	NA
1970-1984	50-55	Childhood autism (inpatient)	0-4	NA	0-4	NA	0-4	NA
1985-1994	50-55	Childhood autism (inpatient)	0-4	NA	0-4	NA	0-4	NA
1995-2004	50-55	Childhood autism (inpatient)	0-4	NA	0-4	NA	0-4	NA
2005-2016	50-55	Childhood autism (inpatient)	5	0.01 (0.01-0.01)	0-4	NA	0-4	NA
1970-2016	55-60	Childhood autism (inpatient)	0-4	NA	0-4	NA	0-4	NA
1970-1984	55-60	Childhood autism (inpatient)	0-4	NA	0-4	NA	0-4	NA
1985-1994	55-60	Childhood autism (inpatient)	0-4	NA	0-4	NA	0-4	NA
1995-2004	55-60	Childhood autism (inpatient)	0-4	NA	0-4	NA	0-4	NA
2005-2016	55-60	Childhood autism (inpatient)	0-4	NA	0-4	NA	0-4	NA
1970-2016	60-65	Childhood autism (inpatient)	0-4	NA	0-4	NA	0-4	NA
1970-1984	60-65	Childhood autism (inpatient)	0-4	NA	0-4	NA	0-4	NA
1985-1994	60-65	Childhood autism (inpatient)	0-4	NA	0-4	NA	0-4	NA
1995-2004	60-65	Childhood autism (inpatient)	0-4	NA	0-4	NA	0-4	NA
2005-2016	60-65	Childhood autism (inpatient)	0-4	NA	0-4	NA	0-4	NA
1970-2016	65-70	Childhood autism (inpatient)	0-4	NA	0-4	NA	0-4	NA
1970-1984	65-70	Childhood autism (inpatient)	0-4	NA	0-4	NA	0-4	NA
1985-1994	65-70	Childhood autism (inpatient)	0-4	NA	0-4	NA	0-4	NA
1995-2004	65-70	Childhood autism (inpatient)	0-4	NA	0-4	NA	0-4	NA

			Both sexes		Males		Females	
**Years**	**Age**	**Mental disorder**	**Cases**	**IR (95% CI)**	**Cases**	**IR (95% CI)**	**Cases**	**IR (95% CI)**
2005-2016	65-70	Childhood autism (inpatient)	0-4	NA	0-4	NA	0-4	NA
1970-2016	70-75	Childhood autism (inpatient)	0-4	NA	0-4	NA	0-4	NA
1970-1984	70-75	Childhood autism (inpatient)	0-4	NA	0-4	NA	0-4	NA
1985-1994	70-75	Childhood autism (inpatient)	0-4	NA	0-4	NA	0-4	NA
1995-2004	70-75	Childhood autism (inpatient)	0-4	NA	0-4	NA	0-4	NA
2005-2016	70-75	Childhood autism (inpatient)	0-4	NA	0-4	NA	0-4	NA
1970-2016	75-80	Childhood autism (inpatient)	0-4	NA	0-4	NA	0-4	NA
1970-1984	75-80	Childhood autism (inpatient)	0-4	NA	0-4	NA	0-4	NA
1985-1994	75-80	Childhood autism (inpatient)	0-4	NA	0-4	NA	0-4	NA
1995-2004	75-80	Childhood autism (inpatient)	0-4	NA	0-4	NA	0-4	NA
2005-2016	75-80	Childhood autism (inpatient)	0-4	NA	0-4	NA	0-4	NA
1970-2016	80-85	Childhood autism (inpatient)	0-4	NA	0-4	NA	0-4	NA
1970-1984	80-85	Childhood autism (inpatient)	0-4	NA	0-4	NA	0-4	NA
1985-1994	80-85	Childhood autism (inpatient)	0-4	NA	0-4	NA	0-4	NA
1995-2004	80-85	Childhood autism (inpatient)	0-4	NA	0-4	NA	0-4	NA
2005-2016	80-85	Childhood autism (inpatient)	0-4	NA	0-4	NA	0-4	NA
1970-2016	85-90	Childhood autism (inpatient)	0-4	NA	0-4	NA	0-4	NA
1970-1984	85-90	Childhood autism (inpatient)	0-4	NA	0-4	NA	0-4	NA
1985-1994	85-90	Childhood autism (inpatient)	0-4	NA	0-4	NA	0-4	NA
1995-2004	85-90	Childhood autism (inpatient)	0-4	NA	0-4	NA	0-4	NA
2005-2016	85-90	Childhood autism (inpatient)	0-4	NA	0-4	NA	0-4	NA
1970-2016	90-95	Childhood autism (inpatient)	0-4	NA	0-4	NA	0-4	NA
1970-1984	90-95	Childhood autism (inpatient)	0-4	NA	0-4	NA	0-4	NA
1985-1994	90-95	Childhood autism (inpatient)	0-4	NA	0-4	NA	0-4	NA
1995-2004	90-95	Childhood autism (inpatient)	0-4	NA	0-4	NA	0-4	NA
2005-2016	90-95	Childhood autism (inpatient)	0-4	NA	0-4	NA	0-4	NA
1970-2016	95-100	Childhood autism (inpatient)	0-4	NA	0-4	NA	0-4	NA
1970-1984	95-100	Childhood autism (inpatient)	0-4	NA	0-4	NA	0-4	NA
1985-1994	95-100	Childhood autism (inpatient)	0-4	NA	0-4	NA	0-4	NA
1995-2004	95-100	Childhood autism (inpatient)	0-4	NA	0-4	NA	0-4	NA
2005-2016	95-100	Childhood autism (inpatient)	0-4	NA	0-4	NA	0-4	NA
1970-2016	1-5	Behavioral disorders	4073	3.36 (3.36-3.36)	2826	4.55 (4.55-4.56)	1247	2.11 (2.11-2.11)
1970-1984	1-5	Behavioral disorders	162	0.40 (0.40-0.40)	110	0.53 (0.53-0.53)	52	0.26 (0.26-0.26)
1985-1994	1-5	Behavioral disorders	282	1.23 (1.23-1.23)	184	1.56 (1.56-1.57)	98	0.88 (0.87-0.88)
1995-2004	1-5	Behavioral disorders	1249	4.61 (4.60-4.61)	853	6.14 (6.13-6.15)	396	3.00 (2.99-3.00)
2005-2016	1-5	Behavioral disorders	2380	7.83 (7.82-7.84)	1679	10.78 (10.76-10.80)	701	4.73 (4.72-4.74)
1970-2016	5-10	Behavioral disorders	27576	17.76 (17.75-17.77)	21257	26.80 (26.78-26.82)	6319	8.32 (8.32-8.33)
1970-1984	5-10	Behavioral disorders	1363	2.45 (2.45-2.45)	1044	3.66 (3.66-3.67)	319	1.17 (1.17-1.17)
1985-1994	5-10	Behavioral disorders	1254	4.48 (4.48-4.49)	954	6.68 (6.67-6.69)	300	2.19 (2.19-2.20)
1995-2004	5-10	Behavioral disorders	6632	20.25 (20.22-20.27)	5223	31.15 (31.11-31.20)	1409	8.81 (8.80-8.83)

			Both sexes		Males		Females	
**Years**	**Age**	**Mental disorder**	**Cases**	**IR (95% CI)**	**Cases**	**IR (95% CI)**	**Cases**	**IR (95% CI)**
2005-2016	5-10	Behavioral disorders	18327	47.18 (47.14-47.23)	14036	70.98 (70.88-71.08)	4291	22.50 (22.47-22.54)
1970-2016	10-15	Behavioral disorders	24938	15.92 (15.91-15.93)	16355	20.50 (20.48-20.51)	8583	11.17 (11.16-11.18)
1970-1984	10-15	Behavioral disorders	1457	2.56 (2.56-2.56)	957	3.29 (3.28-3.29)	500	1.80 (1.80-1.80)
1985-1994	10-15	Behavioral disorders	1213	3.86 (3.85-3.86)	773	4.82 (4.81-4.82)	440	2.85 (2.85-2.86)
1995-2004	10-15	Behavioral disorders	5313	18.16 (18.14-18.19)	3608	24.19 (24.15-24.23)	1705	11.90 (11.88-11.92)
2005-2016	10-15	Behavioral disorders	16955	43.45 (43.41-43.50)	11017	55.86 (55.79-55.94)	5938	30.77 (30.73-30.81)
1970-2016	15-20	Behavioral disorders	15187	9.56 (9.55-9.56)	7437	9.15 (9.15-9.16)	7750	9.98 (9.97-9.99)
1970-1984	15-20	Behavioral disorders	584	1.02 (1.02-1.03)	305	1.04 (1.04-1.04)	279	1.01 (1.00-1.01)
1985-1994	15-20	Behavioral disorders	359	1.02 (1.02-1.02)	144	0.80 (0.80-0.80)	215	1.25 (1.25-1.25)
1995-2004	15-20	Behavioral disorders	1835	6.51 (6.50-6.52)	950	6.60 (6.59-6.61)	885	6.41 (6.40-6.42)
2005-2016	15-20	Behavioral disorders	12409	32.22 (32.19-32.25)	6038	30.86 (30.82-30.90)	6371	33.62 (33.58-33.67)
1970-2016	20-25	Behavioral disorders	5421	3.39 (3.38-3.39)	3160	3.83 (3.83-3.83)	2261	2.92 (2.91-2.92)
1970-1984	20-25	Behavioral disorders	177	0.32 (0.32-0.32)	107	0.37 (0.37-0.37)	70	0.26 (0.26-0.26)
1985-1994	20-25	Behavioral disorders	104	0.28 (0.28-0.28)	30	0.16 (0.15-0.16)	74	0.41 (0.41-0.41)
1995-2004	20-25	Behavioral disorders	210	0.68 (0.68-0.68)	146	0.92 (0.91-0.92)	64	0.43 (0.43-0.43)
2005-2016	20-25	Behavioral disorders	4930	13.50 (13.48-13.51)	2877	15.45 (15.43-15.47)	2053	11.46 (11.45-11.48)
1970-2016	25-30	Behavioral disorders	3703	2.30 (2.29-2.30)	2188	2.64 (2.63-2.64)	1515	1.93 (1.93-1.94)
1970-1984	25-30	Behavioral disorders	140	0.25 (0.25-0.25)	70	0.24 (0.24-0.24)	70	0.25 (0.25-0.25)
1985-1994	25-30	Behavioral disorders	84	0.23 (0.23-0.23)	41	0.21 (0.21-0.22)	43	0.24 (0.24-0.24)
1995-2004	25-30	Behavioral disorders	124	0.37 (0.37-0.37)	79	0.45 (0.45-0.46)	45	0.28 (0.28-0.28)
2005-2016	25-30	Behavioral disorders	3355	9.82 (9.81-9.83)	1998	11.49 (11.47-11.50)	1357	8.09 (8.08-8.10)
1970-2016	30-35	Behavioral disorders	2933	1.83 (1.83-1.83)	1649	2.01 (2.01-2.01)	1284	1.64 (1.64-1.64)
1970-1984	30-35	Behavioral disorders	179	0.33 (0.33-0.33)	95	0.34 (0.34-0.34)	84	0.32 (0.31-0.32)
1985-1994	30-35	Behavioral disorders	85	0.25 (0.25-0.25)	32	0.18 (0.18-0.18)	53	0.32 (0.31-0.32)
1995-2004	30-35	Behavioral disorders	105	0.29 (0.29-0.29)	71	0.38 (0.38-0.38)	34	0.19 (0.19-0.20)
2005-2016	30-35	Behavioral disorders	2564	7.25 (7.24-7.25)	1451	8.05 (8.04-8.06)	1113	6.41 (6.40-6.42)
1970-2016	35-40	Behavioral disorders	2339	1.47 (1.47-1.47)	1259	1.56 (1.56-1.56)	1080	1.38 (1.38-1.38)
1970-1984	35-40	Behavioral disorders	171	0.35 (0.34-0.35)	74	0.30 (0.30-0.30)	97	0.40 (0.40-0.40)
1985-1994	35-40	Behavioral disorders	65	0.19 (0.19-0.19)	22	0.12 (0.12-0.12)	43	0.25 (0.25-0.25)
1995-2004	35-40	Behavioral disorders	63	0.18 (0.18-0.18)	41	0.22 (0.22-0.22)	22	0.13 (0.13-0.13)
2005-2016	35-40	Behavioral disorders	2040	5.30 (5.29-5.30)	1122	5.72 (5.71-5.73)	918	4.86 (4.85-4.87)
1970-2016	40-45	Behavioral disorders	1644	1.06 (1.05-1.06)	878	1.12 (1.11-1.12)	766	0.99 (0.99-0.99)
1970-1984	40-45	Behavioral disorders	121	0.28 (0.28-0.28)	46	0.21 (0.21-0.21)	75	0.35 (0.34-0.35)
1985-1994	40-45	Behavioral disorders	72	0.20 (0.19-0.20)	30	0.16 (0.16-0.16)	42	0.23 (0.23-0.23)
1995-2004	40-45	Behavioral disorders	56	0.17 (0.17-0.17)	38	0.22 (0.22-0.22)	18	0.11 (0.11-0.11)
2005-2016	40-45	Behavioral disorders	1395	3.36 (3.35-3.36)	764	3.62 (3.61-3.62)	631	3.09 (3.08-3.09)
1970-2016	45-50	Behavioral disorders	1069	0.70 (0.70-0.70)	578	0.76 (0.76-0.76)	491	0.65 (0.65-0.65)
1970-1984	45-50	Behavioral disorders	115	0.27 (0.27-0.27)	43	0.21 (0.21-0.21)	72	0.34 (0.34-0.34)
1985-1994	45-50	Behavioral disorders	62	0.18 (0.18-0.18)	22	0.13 (0.13-0.13)	40	0.24 (0.24-0.24)
1995-2004	45-50	Behavioral disorders	30	0.09 (0.09-0.09)	17	0.10 (0.10-0.10)	13	0.08 (0.08-0.08)

			Both sexes		Males		Females	
Years	Age	Mental disorder	Cases	IR (95% CI)	Cases	IR (95% CI)	Cases	IR (95% CI)
2005-2016	45-50	Behavioral disorders	862	2.06 (2.06-2.07)	496	2.35 (2.35-2.35)	366	1.77 (1.77-1.77)
1970-2016	50-55	Behavioral disorders	530	0.36 (0.36-0.36)	279	0.39 (0.39-0.39)	251	0.34 (0.34-0.34)
1970-1984	50-55	Behavioral disorders	86	0.20 (0.20-0.20)	34	0.16 (0.16-0.16)	52	0.24 (0.24-0.24)
1985-1994	50-55	Behavioral disorders	29	0.10 (0.10-0.10)	15	0.11 (0.11-0.11)	14	0.10 (0.10-0.10)
1995-2004	50-55	Behavioral disorders	29	0.08 (0.08-0.08)	15	0.09 (0.08-0.09)	14	0.08 (0.08-0.08)
2005-2016	50-55	Behavioral disorders	386	0.97 (0.97-0.97)	215	1.08 (1.08-1.08)	171	0.87 (0.86-0.87)
1970-2016	55-60	Behavioral disorders	314	0.23 (0.23-0.23)	150	0.22 (0.22-0.22)	164	0.23 (0.23-0.23)
1970-1984	55-60	Behavioral disorders	71	0.17 (0.17-0.17)	23	0.11 (0.11-0.11)	48	0.22 (0.22-0.22)
1985-1994	55-60	Behavioral disorders	33	0.13 (0.13-0.13)	12	0.10 (0.10-0.10)	21	0.16 (0.16-0.16)
1995-2004	55-60	Behavioral disorders	17	0.05 (0.05-0.05)	6	0.04 (0.04-0.04)	11	0.07 (0.07-0.07)
2005-2016	55-60	Behavioral disorders	193	0.50 (0.50-0.50)	109	0.57 (0.57-0.57)	84	0.43 (0.43-0.43)
1970-2016	60-65	Behavioral disorders	168	0.13 (0.13-0.13)	62	0.10 (0.10-0.10)	106	0.16 (0.16-0.16)
1970-1984	60-65	Behavioral disorders	59	0.15 (0.15-0.15)	19	0.10 (0.10-0.10)	40	0.19 (0.19-0.19)
1985-1994	60-65	Behavioral disorders	33	0.13 (0.13-0.13)	8	0.07 (0.07-0.07)	25	0.19 (0.19-0.19)
1995-2004	60-65	Behavioral disorders	17	0.07 (0.07-0.07)	8	0.06 (0.06-0.06)	9	0.07 (0.07-0.07)
2005-2016	60-65	Behavioral disorders	59	0.15 (0.15-0.15)	27	0.14 (0.14-0.14)	32	0.16 (0.16-0.16)
1970-2016	65-70	Behavioral disorders	112	0.10 (0.10-0.10)	52	0.10 (0.10-0.10)	60	0.10 (0.10-0.10)
1970-1984	65-70	Behavioral disorders	40	0.11 (0.11-0.11)	17	0.10 (0.10-0.10)	23	0.12 (0.12-0.12)
1985-1994	65-70	Behavioral disorders	27	0.11 (0.11-0.11)	10	0.09 (0.09-0.09)	17	0.13 (0.13-0.13)
1995-2004	65-70	Behavioral disorders	10	0.05 (0.05-0.05)	0-4	NA	6-10	0.06 (0.06-0.06)
2005-2016	65-70	Behavioral disorders	35	0.10 (0.10-0.10)	22	0.13 (0.13-0.13)	13	0.07 (0.07-0.07)
1970-2016	70-75	Behavioral disorders	90	0.09 (0.09-0.09)	29	0.07 (0.07-0.07)	61	0.12 (0.12-0.12)
1970-1984	70-75	Behavioral disorders	34	0.12 (0.12-0.12)	11	0.09 (0.09-0.09)	23	0.14 (0.14-0.14)
1985-1994	70-75	Behavioral disorders	16	0.08 (0.08-0.08)	8	0.09 (0.09-0.09)	8	0.07 (0.07-0.07)
1995-2004	70-75	Behavioral disorders	17	0.09 (0.09-0.09)	0-4	NA	13-17	0.16 (0.16-0.16)
2005-2016	70-75	Behavioral disorders	23	0.09 (0.09-0.09)	10	0.08 (0.08-0.08)	13	0.09 (0.09-0.09)
1970-2016	75-80	Behavioral disorders	49	0.07 (0.07-0.07)	15	0.05 (0.05-0.05)	34	0.08 (0.08-0.08)
1970-1984	75-80	Behavioral disorders	17	0.08 (0.08-0.08)	0-4	NA	13-17	0.12 (0.12-0.12)
1985-1994	75-80	Behavioral disorders	6	0.04 (0.04-0.04)	0-4	NA	2-6	0.05 (0.05-0.05)
1995-2004	75-80	Behavioral disorders	9	0.06 (0.06-0.06)	0-4	NA	5-9	0.05 (0.05-0.05)
2005-2016	75-80	Behavioral disorders	17	0.09 (0.09-0.09)	8	0.09 (0.09-0.09)	9	0.08 (0.08-0.08)
1970-2016	80-85	Behavioral disorders	48	0.10 (0.10-0.10)	18	0.10 (0.10-0.10)	30	0.10 (0.10-0.10)
1970-1984	80-85	Behavioral disorders	8	0.07 (0.07-0.07)	0-4	NA	4-8	0.08 (0.08-0.08)
1985-1994	80-85	Behavioral disorders	6	0.05 (0.05-0.06)	0-4	NA	0-4	NA
1995-2004	80-85	Behavioral disorders	12	0.10 (0.10-0.10)	0-4	NA	8-12	0.11 (0.11-0.11)
2005-2016	80-85	Behavioral disorders	22	0.16 (0.16-0.16)	10	0.18 (0.18-0.18)	12	0.14 (0.14-0.14)
1970-2016	85-90	Behavioral disorders	31	0.12 (0.12-0.12)	6	0.07 (0.07-0.07)	25	0.14 (0.14-0.14)
1970-1984	85-90	Behavioral disorders	6	0.11 (0.11-0.11)	0-4	NA	0-4	NA
1985-1994	85-90	Behavioral disorders	0-4	NA	0-4	NA	0-4	NA
1995-2004	85-90	Behavioral disorders	9	0.14 (0.14-0.14)	0-4	NA	5-9	0.13 (0.13-0.13)

			Both sexes		Males		Females	
Years	Age	Mental disorder	Cases	IR (95% CI)	Cases	IR (95% CI)	Cases	IR (95% CI)
2005-2016	85-90	Behavioral disorders	13	0.15 (0.15-0.15)	0-4	NA	9-13	0.21 (0.21-0.21)
1970-2016	90-95	Behavioral disorders	12	0.13 (0.13-0.13)	0-4	NA	8-12	0.12 (0.12-0.12)
1970-1984	90-95	Behavioral disorders	0-4	NA	0-4	NA	0-4	NA
1985-1994	90-95	Behavioral disorders	0-4	NA	0-4	NA	0-4	NA
1995-2004	90-95	Behavioral disorders	0-4	NA	0-4	NA	0-4	NA
2005-2016	90-95	Behavioral disorders	6	0.17 (0.17-0.17)	0-4	NA	0-4	NA
1970-2016	95-100	Behavioral disorders	0-4	NA	0-4	NA	0-4	NA
1970-1984	95-100	Behavioral disorders	0-4	NA	0-4	NA	0-4	NA
1985-1994	95-100	Behavioral disorders	0-4	NA	0-4	NA	0-4	NA
1995-2004	95-100	Behavioral disorders	0-4	NA	0-4	NA	0-4	NA
2005-2016	95-100	Behavioral disorders	0-4	NA	0-4	NA	0-4	NA
1970-2016	1-5	Behavioral disorders (inpatient)	612	0.50 (0.50-0.51)	421	0.68 (0.68-0.68)	191	0.32 (0.32-0.32)
1970-1984	1-5	Behavioral disorders (inpatient)	162	0.40 (0.40-0.40)	110	0.53 (0.53-0.53)	52	0.26 (0.26-0.26)
1985-1994	1-5	Behavioral disorders (inpatient)	243	1.06 (1.06-1.06)	162	1.38 (1.37-1.38)	81	0.72 (0.72-0.72)
1995-2004	1-5	Behavioral disorders (inpatient)	188	0.69 (0.69-0.69)	134	0.96 (0.96-0.97)	54	0.41 (0.41-0.41)
2005-2016	1-5	Behavioral disorders (inpatient)	19	0.06 (0.06-0.06)	15-19	0.10 (0.10-0.10)	0-4	NA
1970-2016	5-10	Behavioral disorders (inpatient)	3776	2.42 (2.42-2.42)	2897	3.63 (3.63-3.63)	879	1.16 (1.15-1.16)
1970-1984	5-10	Behavioral disorders (inpatient)	1363	2.45 (2.45-2.45)	1044	3.66 (3.66-3.67)	319	1.17 (1.17-1.17)
1985-1994	5-10	Behavioral disorders (inpatient)	1082	3.87 (3.86-3.87)	833	5.83 (5.82-5.84)	249	1.82 (1.82-1.82)
1995-2004	5-10	Behavioral disorders (inpatient)	952	2.89 (2.89-2.90)	738	4.37 (4.37-4.38)	214	1.34 (1.33-1.34)
2005-2016	5-10	Behavioral disorders (inpatient)	379	0.96 (0.96-0.97)	282	1.40 (1.40-1.40)	97	0.51 (0.51-0.51)
1970-2016	10-15	Behavioral disorders (inpatient)	4667	2.96 (2.95-2.96)	2807	3.48 (3.47-3.48)	1860	2.41 (2.41-2.41)
1970-1984	10-15	Behavioral disorders (inpatient)	1457	2.56 (2.56-2.56)	957	3.29 (3.28-3.29)	500	1.80 (1.80-1.80)
1985-1994	10-15	Behavioral disorders (inpatient)	1022	3.25 (3.24-3.25)	644	4.01 (4.01-4.02)	378	2.45 (2.45-2.46)
1995-2004	10-15	Behavioral disorders (inpatient)	858	2.91 (2.91-2.91)	546	3.62 (3.61-3.62)	312	2.17 (2.16-2.17)
2005-2016	10-15	Behavioral disorders (inpatient)	1330	3.32 (3.32-3.32)	660	3.22 (3.21-3.22)	670	3.43 (3.42-3.43)
1970-2016	15-20	Behavioral disorders (inpatient)	3364	2.10 (2.10-2.10)	1543	1.88 (1.88-1.88)	1821	2.33 (2.33-2.33)
1970-1984	15-20	Behavioral disorders (inpatient)	584	1.02 (1.02-1.03)	305	1.04 (1.04-1.04)	279	1.01 (1.00-1.01)
1985-1994	15-20	Behavioral disorders (inpatient)	331	0.94 (0.94-0.94)	130	0.72 (0.72-0.72)	201	1.17 (1.17-1.17)
1995-2004	15-20	Behavioral disorders (inpatient)	415	1.46 (1.46-1.47)	189	1.30 (1.30-1.31)	226	1.63 (1.63-1.63)
2005-2016	15-20	Behavioral disorders (inpatient)	2034	5.12 (5.12-5.13)	919	4.51 (4.50-4.51)	1115	5.77 (5.76-5.78)
1970-2016	20-25	Behavioral disorders (inpatient)	1495	0.93 (0.93-0.93)	908	1.09 (1.09-1.09)	587	0.75 (0.75-0.75)
1970-1984	20-25	Behavioral disorders (inpatient)	177	0.32 (0.32-0.32)	107	0.37 (0.37-0.37)	70	0.26 (0.26-0.26)
1985-1994	20-25	Behavioral disorders (inpatient)	99	0.27 (0.27-0.27)	28	0.14 (0.14-0.14)	71	0.40 (0.40-0.40)
1995-2004	20-25	Behavioral disorders (inpatient)	58	0.19 (0.19-0.19)	40	0.25 (0.25-0.25)	18	0.12 (0.12-0.12)
2005-2016	20-25	Behavioral disorders (inpatient)	1161	3.11 (3.10-3.11)	733	3.82 (3.82-3.83)	428	2.35 (2.35-2.36)
1970-2016	25-30	Behavioral disorders (inpatient)	918	0.57 (0.57-0.57)	569	0.68 (0.68-0.68)	349	0.44 (0.44-0.44)
1970-1984	25-30	Behavioral disorders (inpatient)	140	0.25 (0.25-0.25)	70	0.24 (0.24-0.24)	70	0.25 (0.25-0.25)
1985-1994	25-30	Behavioral disorders (inpatient)	79	0.21 (0.21-0.21)	38	0.20 (0.20-0.20)	41	0.23 (0.23-0.23)
1995-2004	25-30	Behavioral disorders (inpatient)	33	0.10 (0.10-0.10)	22	0.13 (0.13-0.13)	11	0.07 (0.07-0.07)

			Both sexes		Males		Females	
Years	Age	Mental disorder	Cases	IR (95% CI)	Cases	IR (95% CI)	Cases	IR (95% CI)
2005-2016	25-30	Behavioral disorders (inpatient)	666	1.93 (1.92-1.93)	439	2.49 (2.48-2.49)	227	1.34 (1.34-1.34)
1970-2016	30-35	Behavioral disorders (inpatient)	865	0.54 (0.54-0.54)	504	0.61 (0.61-0.61)	361	0.46 (0.46-0.46)
1970-1984	30-35	Behavioral disorders (inpatient)	179	0.33 (0.33-0.33)	95	0.34 (0.34-0.34)	84	0.32 (0.31-0.32)
1985-1994	30-35	Behavioral disorders (inpatient)	81	0.23 (0.23-0.23)	29	0.16 (0.16-0.16)	52	0.31 (0.31-0.31)
1995-2004	30-35	Behavioral disorders (inpatient)	29	0.08 (0.08-0.08)	25-29	0.13 (0.13-0.13)	0-4	NA
2005-2016	30-35	Behavioral disorders (inpatient)	576	1.62 (1.62-1.62)	355	1.96 (1.96-1.96)	221	1.27 (1.27-1.27)
1970-2016	35-40	Behavioral disorders (inpatient)	696	0.44 (0.44-0.44)	389	0.48 (0.48-0.48)	307	0.39 (0.39-0.39)
1970-1984	35-40	Behavioral disorders (inpatient)	171	0.35 (0.34-0.35)	74	0.30 (0.30-0.30)	97	0.40 (0.40-0.40)
1985-1994	35-40	Behavioral disorders (inpatient)	63	0.18 (0.18-0.18)	22	0.12 (0.12-0.12)	41	0.24 (0.24-0.24)
1995-2004	35-40	Behavioral disorders (inpatient)	19	0.05 (0.05-0.05)	10	0.05 (0.05-0.05)	9	0.05 (0.05-0.05)
2005-2016	35-40	Behavioral disorders (inpatient)	443	1.15 (1.15-1.15)	283	1.44 (1.44-1.44)	160	0.85 (0.84-0.85)
1970-2016	40-45	Behavioral disorders (inpatient)	576	0.37 (0.37-0.37)	319	0.41 (0.40-0.41)	257	0.33 (0.33-0.33)
1970-1984	40-45	Behavioral disorders (inpatient)	121	0.28 (0.28-0.28)	46	0.21 (0.21-0.21)	75	0.35 (0.34-0.35)
1985-1994	40-45	Behavioral disorders (inpatient)	64	0.17 (0.17-0.17)	27	0.15 (0.14-0.15)	37	0.20 (0.20-0.20)
1995-2004	40-45	Behavioral disorders (inpatient)	14	0.04 (0.04-0.04)	10-14	0.06 (0.06-0.06)	0-4	NA
2005-2016	40-45	Behavioral disorders (inpatient)	377	0.91 (0.90-0.91)	235	1.11 (1.11-1.11)	142	0.69 (0.69-0.69)
1970-2016	45-50	Behavioral disorders (inpatient)	389	0.26 (0.26-0.26)	187	0.25 (0.25-0.25)	202	0.27 (0.27-0.27)
1970-1984	45-50	Behavioral disorders (inpatient)	115	0.27 (0.27-0.27)	43	0.21 (0.21-0.21)	72	0.34 (0.34-0.34)
1985-1994	45-50	Behavioral disorders (inpatient)	59	0.17 (0.17-0.17)	21	0.12 (0.12-0.12)	38	0.23 (0.22-0.23)
1995-2004	45-50	Behavioral disorders (inpatient)	10	0.03 (0.03-0.03)	5	0.03 (0.03-0.03)	5	0.03 (0.03-0.03)
2005-2016	45-50	Behavioral disorders (inpatient)	205	0.49 (0.49-0.49)	118	0.56 (0.56-0.56)	87	0.42 (0.42-0.42)
1970-2016	50-55	Behavioral disorders (inpatient)	219	0.15 (0.15-0.15)	112	0.15 (0.15-0.16)	107	0.15 (0.15-0.15)
1970-1984	50-55	Behavioral disorders (inpatient)	86	0.20 (0.20-0.20)	34	0.16 (0.16-0.16)	52	0.24 (0.24-0.24)
1985-1994	50-55	Behavioral disorders (inpatient)	29	0.10 (0.10-0.10)	15	0.11 (0.11-0.11)	14	0.10 (0.10-0.10)
1995-2004	50-55	Behavioral disorders (inpatient)	12	0.03 (0.03-0.03)	6	0.03 (0.03-0.03)	6	0.03 (0.03-0.03)
2005-2016	50-55	Behavioral disorders (inpatient)	92	0.23 (0.23-0.23)	57	0.29 (0.29-0.29)	35	0.18 (0.18-0.18)
1970-2016	55-60	Behavioral disorders (inpatient)	160	0.12 (0.12-0.12)	70	0.10 (0.10-0.10)	90	0.13 (0.13-0.13)
1970-1984	55-60	Behavioral disorders (inpatient)	71	0.17 (0.17-0.17)	23	0.11 (0.11-0.11)	48	0.22 (0.22-0.22)
1985-1994	55-60	Behavioral disorders (inpatient)	33	0.13 (0.13-0.13)	12	0.10 (0.10-0.10)	21	0.16 (0.16-0.16)
1995-2004	55-60	Behavioral disorders (inpatient)	0-4	NA	0-4	NA	0-4	NA
2005-2016	55-60	Behavioral disorders (inpatient)	52	0.14 (0.13-0.14)	34	0.18 (0.18-0.18)	18	0.09 (0.09-0.09)
1970-2016	60-65	Behavioral disorders (inpatient)	109	0.08 (0.08-0.08)	38	0.06 (0.06-0.06)	71	0.11 (0.11-0.11)
1970-1984	60-65	Behavioral disorders (inpatient)	59	0.15 (0.15-0.15)	19	0.10 (0.10-0.10)	40	0.19 (0.19-0.19)
1985-1994	60-65	Behavioral disorders (inpatient)	33	0.13 (0.13-0.13)	8	0.07 (0.07-0.07)	25	0.19 (0.19-0.19)
1995-2004	60-65	Behavioral disorders (inpatient)	0-4	NA	0-4	NA	0-4	NA
2005-2016	60-65	Behavioral disorders (inpatient)	14	0.04 (0.04-0.04)	10-14	0.05 (0.05-0.05)	0-4	NA
1970-2016	65-70	Behavioral disorders (inpatient)	79	0.07 (0.07-0.07)	33	0.06 (0.06-0.06)	46	0.08 (0.08-0.08)
1970-1984	65-70	Behavioral disorders (inpatient)	40	0.11 (0.11-0.11)	17	0.10 (0.10-0.10)	23	0.12 (0.12-0.12)
1985-1994	65-70	Behavioral disorders (inpatient)	27	0.11 (0.11-0.11)	10	0.09 (0.09-0.09)	17	0.13 (0.13-0.13)
1995-2004	65-70	Behavioral disorders (inpatient)	0-4	NA	0-4	NA	0-4	NA

			Both sexes		Males		Females	
Years	Age	Mental disorder	Cases	IR (95% CI)	Cases	IR (95% CI)	Cases	IR (95% CI)
2005-2016	65-70	Behavioral disorders (inpatient)	8	0.02 (0.02-0.02)	4-8	0.04 (0.04-0.04)	0-4	NA
1970-2016	70-75	Behavioral disorders (inpatient)	59	0.06 (0.06-0.06)	20	0.05 (0.05-0.05)	39	0.07 (0.07-0.07)
1970-1984	70-75	Behavioral disorders (inpatient)	34	0.12 (0.12-0.12)	11	0.09 (0.09-0.09)	23	0.14 (0.14-0.14)
1985-1994	70-75	Behavioral disorders (inpatient)	16	0.08 (0.08-0.08)	8	0.09 (0.09-0.09)	8	0.07 (0.07-0.07)
1995-2004	70-75	Behavioral disorders (inpatient)	6	0.03 (0.03-0.03)	0-4	NA	2-6	0.06 (0.06-0.06)
2005-2016	70-75	Behavioral disorders (inpatient)	0-4	NA	0-4	NA	0-4	NA
1970-2016	75-80	Behavioral disorders (inpatient)	27	0.04 (0.04-0.04)	0-4	NA	23-27	0.06 (0.06-0.06)
1970-1984	75-80	Behavioral disorders (inpatient)	17	0.08 (0.08-0.08)	0-4	NA	13-17	0.12 (0.12-0.12)
1985-1994	75-80	Behavioral disorders (inpatient)	6	0.04 (0.04-0.04)	0-4	NA	2-6	0.05 (0.05-0.05)
1995-2004	75-80	Behavioral disorders (inpatient)	0-4	NA	0-4	NA	0-4	NA
2005-2016	75-80	Behavioral disorders (inpatient)	0-4	NA	0-4	NA	0-4	NA
1970-2016	80-85	Behavioral disorders (inpatient)	20	0.04 (0.04-0.04)	7	0.04 (0.04-0.04)	13	0.04 (0.04-0.04)
1970-1984	80-85	Behavioral disorders (inpatient)	8	0.07 (0.07-0.07)	0-4	NA	4-8	0.08 (0.08-0.08)
1985-1994	80-85	Behavioral disorders (inpatient)	6	0.05 (0.05-0.06)	0-4	NA	0-4	NA
1995-2004	80-85	Behavioral disorders (inpatient)	5	0.04 (0.04-0.04)	0-4	NA	0-4	NA
2005-2016	80-85	Behavioral disorders (inpatient)	0-4	NA	0-4	NA	0-4	NA
1970-2016	85-90	Behavioral disorders (inpatient)	13	0.05 (0.05-0.05)	5	0.06 (0.06-0.06)	8	0.05 (0.05-0.05)
1970-1984	85-90	Behavioral disorders (inpatient)	6	0.11 (0.11-0.11)	0-4	NA	0-4	NA
1985-1994	85-90	Behavioral disorders (inpatient)	0-4	NA	0-4	NA	0-4	NA
1995-2004	85-90	Behavioral disorders (inpatient)	0-4	NA	0-4	NA	0-4	NA
2005-2016	85-90	Behavioral disorders (inpatient)	0-4	NA	0-4	NA	0-4	NA
1970-2016	90-95	Behavioral disorders (inpatient)	0-4	NA	0-4	NA	0-4	NA
1970-1984	90-95	Behavioral disorders (inpatient)	0-4	NA	0-4	NA	0-4	NA
1985-1994	90-95	Behavioral disorders (inpatient)	0-4	NA	0-4	NA	0-4	NA
1995-2004	90-95	Behavioral disorders (inpatient)	0-4	NA	0-4	NA	0-4	NA
2005-2016	90-95	Behavioral disorders (inpatient)	0-4	NA	0-4	NA	0-4	NA
1970-2016	95-100	Behavioral disorders (inpatient)	0-4	NA	0-4	NA	0-4	NA
1970-1984	95-100	Behavioral disorders (inpatient)	0-4	NA	0-4	NA	0-4	NA
1985-1994	95-100	Behavioral disorders (inpatient)	0-4	NA	0-4	NA	0-4	NA
1995-2004	95-100	Behavioral disorders (inpatient)	0-4	NA	0-4	NA	0-4	NA
2005-2016	95-100	Behavioral disorders (inpatient)	0-4	NA	0-4	NA	0-4	NA
1970-2016	1-5	ADHD	1570	1.30 (1.29-1.30)	1229	1.98 (1.98-1.98)	341	0.58 (0.58-0.58)
1970-1984	1-5	ADHD	29	0.07 (0.07-0.07)	17	0.08 (0.08-0.08)	12	0.06 (0.06-0.06)
1985-1994	1-5	ADHD	44	0.19 (0.19-0.19)	36	0.31 (0.31-0.31)	8	0.07 (0.07-0.07)
1995-2004	1-5	ADHD	336	1.24 (1.24-1.24)	285	2.05 (2.05-2.05)	51	0.39 (0.39-0.39)
2005-2016	1-5	ADHD	1161	3.82 (3.81-3.82)	891	5.72 (5.71-5.73)	270	1.82 (1.82-1.82)
1970-2016	5-10	ADHD	15550	9.99 (9.99-10.00)	12438	15.63 (15.62-15.64)	3112	4.09 (4.09-4.10)
1970-1984	5-10	ADHD	363	0.65 (0.65-0.65)	299	1.05 (1.05-1.05)	64	0.24 (0.24-0.24)
1985-1994	5-10	ADHD	282	1.01 (1.01-1.01)	232	1.62 (1.62-1.62)	50	0.37 (0.36-0.37)
1995-2004	5-10	ADHD	2443	7.43 (7.42-7.44)	2120	12.58 (12.56-12.60)	323	2.02 (2.01-2.02)

			Both sexes		Males		Females	
Years	Age	Mental disorder	Cases	IR (95% CI)	Cases	IR (95% CI)	Cases	IR (95% CI)
2005-2016	5-10	ADHD	12462	31.94 (31.91-31.97)	9787	49.18 (49.11-49.25)	2675	13.99 (13.97-14.01)
1970-2016	10-15	ADHD	10972	6.97 (6.96-6.97)	8149	10.14 (10.13-10.14)	2823	3.66 (3.66-3.66)
1970-1984	10-15	ADHD	331	0.58 (0.58-0.58)	259	0.89 (0.89-0.89)	72	0.26 (0.26-0.26)
1985-1994	10-15	ADHD	203	0.64 (0.64-0.64)	147	0.91 (0.91-0.91)	56	0.36 (0.36-0.36)
1995-2004	10-15	ADHD	1147	3.89 (3.88-3.89)	972	6.44 (6.43-6.45)	175	1.21 (1.21-1.22)
2005-2016	10-15	ADHD	9291	23.52 (23.50-23.55)	6771	33.77 (33.72-33.81)	2520	12.96 (12.94-12.98)
1970-2016	15-20	ADHD	8247	5.15 (5.15-5.16)	4699	5.73 (5.73-5.74)	3548	4.54 (4.54-4.55)
1970-1984	15-20	ADHD	34	0.06 (0.06-0.06)	23	0.08 (0.08-0.08)	11	0.04 (0.04-0.04)
1985-1994	15-20	ADHD	34	0.10 (0.10-0.10)	22	0.12 (0.12-0.12)	12	0.07 (0.07-0.07)
1995-2004	15-20	ADHD	374	1.32 (1.31-1.32)	295	2.03 (2.03-2.03)	79	0.57 (0.57-0.57)
2005-2016	15-20	ADHD	7805	19.89 (19.87-19.91)	4359	21.79 (21.76-21.82)	3446	17.92 (17.89-17.94)
1970-2016	20-25	ADHD	4995	3.10 (3.10-3.11)	2996	3.61 (3.60-3.61)	1999	2.57 (2.57-2.57)
1970-1984	20-25	ADHD	0-4	NA	0-4	NA	0-4	NA
1985-1994	20-25	ADHD	0-4	NA	0-4	NA	0-4	NA
1995-2004	20-25	ADHD	80	0.26 (0.26-0.26)	72	0.45 (0.45-0.45)	8	0.05 (0.05-0.05)
2005-2016	20-25	ADHD	4910	13.23 (13.22-13.25)	2922	15.40 (15.38-15.42)	1988	10.97 (10.95-10.98)
1970-2016	25-30	ADHD	3340	2.06 (2.06-2.06)	2020	2.42 (2.42-2.43)	1320	1.68 (1.68-1.68)
1970-1984	25-30	ADHD	0-4	NA	0-4	NA	0-4	NA
1985-1994	25-30	ADHD	0-4	NA	0-4	NA	0-4	NA
1995-2004	25-30	ADHD	48	0.14 (0.14-0.14)	37	0.21 (0.21-0.21)	11	0.07 (0.07-0.07)
2005-2016	25-30	ADHD	3289	9.53 (9.52-9.54)	1982	11.26 (11.25-11.28)	1307	7.73 (7.72-7.74)
1970-2016	30-35	ADHD	2493	1.55 (1.55-1.55)	1429	1.74 (1.74-1.74)	1064	1.36 (1.36-1.36)
1970-1984	30-35	ADHD	0-4	NA	0-4	NA	0-4	NA
1985-1994	30-35	ADHD	0-4	NA	0-4	NA	0-4	NA
1995-2004	30-35	ADHD	25	0.07 (0.07-0.07)	17	0.09 (0.09-0.09)	8	0.05 (0.05-0.05)
2005-2016	30-35	ADHD	2465	6.93 (6.92-6.94)	1411	7.78 (7.77-7.79)	1054	6.05 (6.04-6.05)
1970-2016	35-40	ADHD	1940	1.22 (1.22-1.22)	1062	1.31 (1.31-1.31)	878	1.12 (1.12-1.12)
1970-1984	35-40	ADHD	0-4	NA	0-4	NA	0-4	NA
1985-1994	35-40	ADHD	0-4	NA	0-4	NA	0-4	NA
1995-2004	35-40	ADHD	17	0.05 (0.05-0.05)	11	0.06 (0.06-0.06)	6	0.03 (0.03-0.03)
2005-2016	35-40	ADHD	1921	4.97 (4.97-4.98)	1051	5.34 (5.33-5.34)	870	4.59 (4.59-4.60)
1970-2016	40-45	ADHD	1328	0.85 (0.85-0.85)	740	0.94 (0.94-0.94)	588	0.76 (0.76-0.76)
1970-1984	40-45	ADHD	0-4	NA	0-4	NA	0-4	NA
1985-1994	40-45	ADHD	0-4	NA	0-4	NA	0-4	NA
1995-2004	40-45	ADHD	12	0.04 (0.04-0.04)	8-12	0.06 (0.06-0.06)	0-4	NA
2005-2016	40-45	ADHD	1313	3.15 (3.15-3.15)	730	3.44 (3.44-3.45)	583	2.85 (2.84-2.85)
1970-2016	45-50	ADHD	790	0.52 (0.52-0.52)	456	0.60 (0.60-0.60)	334	0.44 (0.44-0.44)
1970-1984	45-50	ADHD	0-4	NA	0-4	NA	0-4	NA
1985-1994	45-50	ADHD	0-4	NA	0-4	NA	0-4	NA
1995-2004	45-50	ADHD	5	0.01 (0.01-0.01)	0-4	NA	0-4	NA

			Both sexes		Males		Females	
**Years**	**Age**	**Mental disorder**	**Cases**	**IR (95% CI)**	**Cases**	**IR (95% CI)**	**Cases**	**IR (95% CI)**
2005-2016	45-50	ADHD	784	1.87 (1.87-1.87)	453	2.14 (2.14-2.14)	331	1.60 (1.60-1.60)
1970-2016	50-55	ADHD	332	0.23 (0.23-0.23)	187	0.26 (0.26-0.26)	145	0.20 (0.20-0.20)
1970-1984	50-55	ADHD	0-4	NA	0-4	NA	0-4	NA
1985-1994	50-55	ADHD	0-4	NA	0-4	NA	0-4	NA
1995-2004	50-55	ADHD	0-4	NA	0-4	NA	0-4	NA
2005-2016	50-55	ADHD	330	0.83 (0.83-0.83)	187	0.94 (0.94-0.94)	143	0.72 (0.72-0.72)
1970-2016	55-60	ADHD	164	0.12 (0.12-0.12)	91	0.13 (0.13-0.13)	73	0.10 (0.10-0.10)
1970-1984	55-60	ADHD	0-4	NA	0-4	NA	0-4	NA
1985-1994	55-60	ADHD	0-4	NA	0-4	NA	0-4	NA
1995-2004	55-60	ADHD	0-4	NA	0-4	NA	0-4	NA
2005-2016	55-60	ADHD	163	0.42 (0.42-0.42)	91	0.48 (0.47-0.48)	72	0.37 (0.37-0.37)
1970-2016	60-65	ADHD	45	0.03 (0.03-0.03)	16	0.03 (0.03-0.03)	29	0.04 (0.04-0.04)
1970-1984	60-65	ADHD	0-4	NA	0-4	NA	0-4	NA
1985-1994	60-65	ADHD	0-4	NA	0-4	NA	0-4	NA
1995-2004	60-65	ADHD	0-4	NA	0-4	NA	0-4	NA
2005-2016	60-65	ADHD	43	0.11 (0.11-0.11)	15	0.08 (0.08-0.08)	28	0.14 (0.14-0.14)
1970-2016	65-70	ADHD	9	0.01 (0.01-0.01)	5-9	0.01 (0.01-0.01)	0-4	NA
1970-1984	65-70	ADHD	0-4	NA	0-4	NA	0-4	NA
1985-1994	65-70	ADHD	0-4	NA	0-4	NA	0-4	NA
1995-2004	65-70	ADHD	0-4	NA	0-4	NA	0-4	NA
2005-2016	65-70	ADHD	9	0.03 (0.03-0.03)	5-9	0.03 (0.03-0.03)	0-4	NA
1970-2016	70-75	ADHD	6	0.01 (0.01-0.01)	0-4	NA	0-4	NA
1970-1984	70-75	ADHD	0-4	NA	0-4	NA	0-4	NA
1985-1994	70-75	ADHD	0-4	NA	0-4	NA	0-4	NA
1995-2004	70-75	ADHD	0-4	NA	0-4	NA	0-4	NA
2005-2016	70-75	ADHD	0-4	NA	0-4	NA	0-4	NA
1970-2016	75-80	ADHD	0-4	NA	0-4	NA	0-4	NA
1970-1984	75-80	ADHD	0-4	NA	0-4	NA	0-4	NA
1985-1994	75-80	ADHD	0-4	NA	0-4	NA	0-4	NA
1995-2004	75-80	ADHD	0-4	NA	0-4	NA	0-4	NA
2005-2016	75-80	ADHD	0-4	NA	0-4	NA	0-4	NA
1970-2016	80-85	ADHD	0-4	NA	0-4	NA	0-4	NA
1970-1984	80-85	ADHD	0-4	NA	0-4	NA	0-4	NA
1985-1994	80-85	ADHD	0-4	NA	0-4	NA	0-4	NA
1995-2004	80-85	ADHD	0-4	NA	0-4	NA	0-4	NA
2005-2016	80-85	ADHD	0-4	NA	0-4	NA	0-4	NA
1970-2016	85-90	ADHD	0-4	NA	0-4	NA	0-4	NA
1970-1984	85-90	ADHD	0-4	NA	0-4	NA	0-4	NA
1985-1994	85-90	ADHD	0-4	NA	0-4	NA	0-4	NA
1995-2004	85-90	ADHD	0-4	NA	0-4	NA	0-4	NA

			Both sexes		Males		Females	
Years	Age	Mental disorder	Cases	IR (95% CI)	Cases	IR (95% CI)	Cases	IR (95% CI)
2005-2016	85-90	ADHD	0-4	NA	0-4	NA	0-4	NA
1970-2016	90-95	ADHD	0-4	NA	0-4	NA	0-4	NA
1970-1984	90-95	ADHD	0-4	NA	0-4	NA	0-4	NA
1985-1994	90-95	ADHD	0-4	NA	0-4	NA	0-4	NA
1995-2004	90-95	ADHD	0-4	NA	0-4	NA	0-4	NA
2005-2016	90-95	ADHD	0-4	NA	0-4	NA	0-4	NA
1970-2016	95-100	ADHD	0-4	NA	0-4	NA	0-4	NA
1970-1984	95-100	ADHD	0-4	NA	0-4	NA	0-4	NA
1985-1994	95-100	ADHD	0-4	NA	0-4	NA	0-4	NA
1995-2004	95-100	ADHD	0-4	NA	0-4	NA	0-4	NA
2005-2016	95-100	ADHD	0-4	NA	0-4	NA	0-4	NA
1970-2016	1-5	ADHD (inpatient)	134	0.11 (0.11-0.11)	103	0.17 (0.17-0.17)	31	0.05 (0.05-0.05)
1970-1984	1-5	ADHD (inpatient)	29	0.07 (0.07-0.07)	17	0.08 (0.08-0.08)	12	0.06 (0.06-0.06)
1985-1994	1-5	ADHD (inpatient)	39	0.17 (0.17-0.17)	32	0.27 (0.27-0.27)	7	0.06 (0.06-0.06)
1995-2004	1-5	ADHD (inpatient)	55	0.20 (0.20-0.20)	46	0.33 (0.33-0.33)	9	0.07 (0.07-0.07)
2005-2016	1-5	ADHD (inpatient)	11	0.04 (0.04-0.04)	7-11	0.05 (0.05-0.05)	0-4	NA
1970-2016	5-10	ADHD (inpatient)	1172	0.75 (0.75-0.75)	957	1.20 (1.20-1.20)	215	0.28 (0.28-0.28)
1970-1984	5-10	ADHD (inpatient)	363	0.65 (0.65-0.65)	299	1.05 (1.05-1.05)	64	0.24 (0.24-0.24)
1985-1994	5-10	ADHD (inpatient)	253	0.90 (0.90-0.90)	206	1.44 (1.44-1.44)	47	0.34 (0.34-0.34)
1995-2004	5-10	ADHD (inpatient)	336	1.02 (1.02-1.02)	286	1.69 (1.69-1.70)	50	0.31 (0.31-0.31)
2005-2016	5-10	ADHD (inpatient)	220	0.56 (0.56-0.56)	166	0.82 (0.82-0.83)	54	0.28 (0.28-0.28)
1970-2016	10-15	ADHD (inpatient)	1255	0.79 (0.79-0.79)	865	1.07 (1.07-1.07)	390	0.51 (0.50-0.51)
1970-1984	10-15	ADHD (inpatient)	331	0.58 (0.58-0.58)	259	0.89 (0.89-0.89)	72	0.26 (0.26-0.26)
1985-1994	10-15	ADHD (inpatient)	190	0.60 (0.60-0.60)	135	0.84 (0.84-0.84)	55	0.36 (0.36-0.36)
1995-2004	10-15	ADHD (inpatient)	165	0.56 (0.56-0.56)	134	0.88 (0.88-0.89)	31	0.22 (0.21-0.22)
2005-2016	10-15	ADHD (inpatient)	569	1.42 (1.42-1.42)	337	1.64 (1.64-1.64)	232	1.19 (1.18-1.19)
1970-2016	15-20	ADHD (inpatient)	1270	0.79 (0.79-0.79)	697	0.85 (0.85-0.85)	573	0.73 (0.73-0.73)
1970-1984	15-20	ADHD (inpatient)	34	0.06 (0.06-0.06)	23	0.08 (0.08-0.08)	11	0.04 (0.04-0.04)
1985-1994	15-20	ADHD (inpatient)	32	0.09 (0.09-0.09)	21	0.12 (0.12-0.12)	11	0.06 (0.06-0.06)
1995-2004	15-20	ADHD (inpatient)	77	0.27 (0.27-0.27)	56	0.38 (0.38-0.39)	21	0.15 (0.15-0.15)
2005-2016	15-20	ADHD (inpatient)	1127	2.83 (2.83-2.83)	597	2.92 (2.92-2.92)	530	2.73 (2.73-2.74)
1970-2016	20-25	ADHD (inpatient)	1103	0.68 (0.68-0.68)	715	0.86 (0.86-0.86)	388	0.50 (0.50-0.50)
1970-1984	20-25	ADHD (inpatient)	0-4	NA	0-4	NA	0-4	NA
1985-1994	20-25	ADHD (inpatient)	0-4	NA	0-4	NA	0-4	NA
1995-2004	20-25	ADHD (inpatient)	25	0.08 (0.08-0.08)	21-25	0.14 (0.14-0.14)	0-4	NA
2005-2016	20-25	ADHD (inpatient)	1075	2.87 (2.86-2.87)	690	3.59 (3.58-3.59)	385	2.11 (2.11-2.11)
1970-2016	25-30	ADHD (inpatient)	643	0.40 (0.40-0.40)	434	0.52 (0.52-0.52)	209	0.27 (0.27-0.27)
1970-1984	25-30	ADHD (inpatient)	0-4	NA	0-4	NA	0-4	NA
1985-1994	25-30	ADHD (inpatient)	0-4	NA	0-4	NA	0-4	NA
1995-2004	25-30	ADHD (inpatient)	12	0.04 (0.04-0.04)	8-12	0.05 (0.05-0.05)	0-4	NA

			Both sexes		Males		Females	
**Years**	**Age**	**Mental disorder**	**Cases**	**IR (95% CI)**	**Cases**	**IR (95% CI)**	**Cases**	**IR (95% CI)**
2005-2016	25-30	ADHD (inpatient)	629	1.81 (1.81-1.81)	425	2.40 (2.39-2.40)	204	1.20 (1.20-1.20)
1970-2016	30-35	ADHD (inpatient)	562	0.35 (0.35-0.35)	352	0.43 (0.43-0.43)	210	0.27 (0.27-0.27)
1970-1984	30-35	ADHD (inpatient)	0-4	NA	0-4	NA	0-4	NA
1985-1994	30-35	ADHD (inpatient)	0-4	NA	0-4	NA	0-4	NA
1995-2004	30-35	ADHD (inpatient)	6	0.02 (0.02-0.02)	2-6	0.03 (0.03-0.03)	0-4	NA
2005-2016	30-35	ADHD (inpatient)	553	1.55 (1.55-1.55)	345	1.90 (1.89-1.90)	208	1.19 (1.19-1.19)
1970-2016	35-40	ADHD (inpatient)	438	0.27 (0.27-0.28)	278	0.34 (0.34-0.34)	160	0.20 (0.20-0.20)
1970-1984	35-40	ADHD (inpatient)	0-4	NA	0-4	NA	0-4	NA
1985-1994	35-40	ADHD (inpatient)	0-4	NA	0-4	NA	0-4	NA
1995-2004	35-40	ADHD (inpatient)	0-4	NA	0-4	NA	0-4	NA
2005-2016	35-40	ADHD (inpatient)	434	1.12 (1.12-1.12)	276	1.40 (1.40-1.40)	158	0.83 (0.83-0.83)
1970-2016	40-45	ADHD (inpatient)	367	0.24 (0.24-0.24)	229	0.29 (0.29-0.29)	138	0.18 (0.18-0.18)
1970-1984	40-45	ADHD (inpatient)	0-4	NA	0-4	NA	0-4	NA
1985-1994	40-45	ADHD (inpatient)	0-4	NA	0-4	NA	0-4	NA
1995-2004	40-45	ADHD (inpatient)	0-4	NA	0-4	NA	0-4	NA
2005-2016	40-45	ADHD (inpatient)	364	0.87 (0.87-0.87)	228	1.07 (1.07-1.08)	136	0.66 (0.66-0.66)
1970-2016	45-50	ADHD (inpatient)	190	0.13 (0.13-0.13)	109	0.14 (0.14-0.14)	81	0.11 (0.11-0.11)
1970-1984	45-50	ADHD (inpatient)	0-4	NA	0-4	NA	0-4	NA
1985-1994	45-50	ADHD (inpatient)	0-4	NA	0-4	NA	0-4	NA
1995-2004	45-50	ADHD (inpatient)	0-4	NA	0-4	NA	0-4	NA
2005-2016	45-50	ADHD (inpatient)	188	0.45 (0.45-0.45)	108	0.51 (0.51-0.51)	80	0.39 (0.39-0.39)
1970-2016	50-55	ADHD (inpatient)	82	0.06 (0.06-0.06)	52	0.07 (0.07-0.07)	30	0.04 (0.04-0.04)
1970-1984	50-55	ADHD (inpatient)	0-4	NA	0-4	NA	0-4	NA
1985-1994	50-55	ADHD (inpatient)	0-4	NA	0-4	NA	0-4	NA
1995-2004	50-55	ADHD (inpatient)	0-4	NA	0-4	NA	0-4	NA
2005-2016	50-55	ADHD (inpatient)	81	0.20 (0.20-0.20)	52	0.26 (0.26-0.26)	29	0.15 (0.15-0.15)
1970-2016	55-60	ADHD (inpatient)	44	0.03 (0.03-0.03)	28	0.04 (0.04-0.04)	16	0.02 (0.02-0.02)
1970-1984	55-60	ADHD (inpatient)	0-4	NA	0-4	NA	0-4	NA
1985-1994	55-60	ADHD (inpatient)	0-4	NA	0-4	NA	0-4	NA
1995-2004	55-60	ADHD (inpatient)	0-4	NA	0-4	NA	0-4	NA
2005-2016	55-60	ADHD (inpatient)	43	0.11 (0.11-0.11)	28	0.15 (0.15-0.15)	15	0.08 (0.08-0.08)
1970-2016	60-65	ADHD (inpatient)	7	0.01 (0.01-0.01)	0-4	NA	0-4	NA
1970-1984	60-65	ADHD (inpatient)	0-4	NA	0-4	NA	0-4	NA
1985-1994	60-65	ADHD (inpatient)	0-4	NA	0-4	NA	0-4	NA
1995-2004	60-65	ADHD (inpatient)	0-4	NA	0-4	NA	0-4	NA
2005-2016	60-65	ADHD (inpatient)	7	0.02 (0.02-0.02)	0-4	NA	0-4	NA
1970-2016	65-70	ADHD (inpatient)	0-4	NA	0-4	NA	0-4	NA
1970-1984	65-70	ADHD (inpatient)	0-4	NA	0-4	NA	0-4	NA
1985-1994	65-70	ADHD (inpatient)	0-4	NA	0-4	NA	0-4	NA
1995-2004	65-70	ADHD (inpatient)	0-4	NA	0-4	NA	0-4	NA

			Both sexes		Males		Females	
Years	Age	Mental disorder	Cases	IR (95% CI)	Cases	IR (95% CI)	Cases	IR (95% CI)
2005-2016	65-70	ADHD (inpatient)	0-4	NA	0-4	NA	0-4	NA
1970-2016	70-75	ADHD (inpatient)	0-4	NA	0-4	NA	0-4	NA
1970-1984	70-75	ADHD (inpatient)	0-4	NA	0-4	NA	0-4	NA
1985-1994	70-75	ADHD (inpatient)	0-4	NA	0-4	NA	0-4	NA
1995-2004	70-75	ADHD (inpatient)	0-4	NA	0-4	NA	0-4	NA
2005-2016	70-75	ADHD (inpatient)	0-4	NA	0-4	NA	0-4	NA
1970-2016	75-80	ADHD (inpatient)	0-4	NA	0-4	NA	0-4	NA
1970-1984	75-80	ADHD (inpatient)	0-4	NA	0-4	NA	0-4	NA
1985-1994	75-80	ADHD (inpatient)	0-4	NA	0-4	NA	0-4	NA
1995-2004	75-80	ADHD (inpatient)	0-4	NA	0-4	NA	0-4	NA
2005-2016	75-80	ADHD (inpatient)	0-4	NA	0-4	NA	0-4	NA
1970-2016	80-85	ADHD (inpatient)	0-4	NA	0-4	NA	0-4	NA
1970-1984	80-85	ADHD (inpatient)	0-4	NA	0-4	NA	0-4	NA
1985-1994	80-85	ADHD (inpatient)	0-4	NA	0-4	NA	0-4	NA
1995-2004	80-85	ADHD (inpatient)	0-4	NA	0-4	NA	0-4	NA
2005-2016	80-85	ADHD (inpatient)	0-4	NA	0-4	NA	0-4	NA
1970-2016	85-90	ADHD (inpatient)	0-4	NA	0-4	NA	0-4	NA
1970-1984	85-90	ADHD (inpatient)	0-4	NA	0-4	NA	0-4	NA
1985-1994	85-90	ADHD (inpatient)	0-4	NA	0-4	NA	0-4	NA
1995-2004	85-90	ADHD (inpatient)	0-4	NA	0-4	NA	0-4	NA
2005-2016	85-90	ADHD (inpatient)	0-4	NA	0-4	NA	0-4	NA
1970-2016	90-95	ADHD (inpatient)	0-4	NA	0-4	NA	0-4	NA
1970-1984	90-95	ADHD (inpatient)	0-4	NA	0-4	NA	0-4	NA
1985-1994	90-95	ADHD (inpatient)	0-4	NA	0-4	NA	0-4	NA
1995-2004	90-95	ADHD (inpatient)	0-4	NA	0-4	NA	0-4	NA
2005-2016	90-95	ADHD (inpatient)	0-4	NA	0-4	NA	0-4	NA
1970-2016	95-100	ADHD (inpatient)	0-4	NA	0-4	NA	0-4	NA
1970-1984	95-100	ADHD (inpatient)	0-4	NA	0-4	NA	0-4	NA
1985-1994	95-100	ADHD (inpatient)	0-4	NA	0-4	NA	0-4	NA
1995-2004	95-100	ADHD (inpatient)	0-4	NA	0-4	NA	0-4	NA
2005-2016	95-100	ADHD (inpatient)	0-4	NA	0-4	NA	0-4	NA

eTable 5A.Case numbers and incidence rates for mental disorders in Denmark, inpatient contacts only (total study period 1970-2016)

Mental disorder	All cases	IR, all	Cases, males	IR, males	Cases, females	IR, females
Any mental disorder (inpatient)	465,886	20.67 (20.67-20.68)	216,249	19.36 (19.35-19.36)	249,637	21.97 (21.97-21.98)
Organic disorders (inpatient)	93,301	7.51 (7.51-7.51)	43,564	7.35 (7.35-7.35)	49,737	7.66 (7.66-7.66)
Dementia in alzheimer (inpatient)	36,764	2.95 (2.95-2.95)	15,549	2.61 (2.61-2.62)	21,215	3.25 (3.25-3.25)
Vascular dementia (inpatient)	15,772	1.26 (1.26-1.26)	8,027	1.35 (1.35-1.35)	7,745	1.19 (1.19-1.19)
Substance use disorder (inpatient)	138,562	6.82 (6.82-6.82)	86,939	8.73 (8.73-8.74)	51,623	4.99 (4.98-4.99)
Alcohol use disorder (inpatient)	104,678	5.14 (5.14-5.14)	69,421	6.96 (6.95-6.96)	35,257	3.40 (3.39-3.40)
Cannabis use disorder (inpatient)	15,754	0.77 (0.77-0.77)	12,012	1.19 (1.19-1.19)	3,742	0.36 (0.36-0.36)
Schizophrenia spectrum disorder (inpatient)	92,727	4.55 (4.55-4.55)	43,626	4.36 (4.36-4.36)	49,101	4.74 (4.74-4.74)
Schizophrenia (inpatient)	37,916	1.85 (1.85-1.85)	21,643	2.15 (2.15-2.16)	16,273	1.56 (1.56-1.56)
Schizoaffective disorders (inpatient)	8,552	0.42 (0.42-0.42)	3,210	0.32 (0.32-0.32)	5,342	0.51 (0.51-0.51)
Mood disorders (inpatient)	161,716	7.98 (7.98-7.98)	61,033	6.10 (6.10-6.10)	100,683	9.80 (9.80-9.80)
Bipolar disorder (inpatient)	28,589	1.40 (1.40-1.40)	11,580	1.15 (1.15-1.15)	17,009	1.63 (1.63-1.63)
Recurrent depression (inpatient)	57,749	2.82 (2.82-2.82)	18,845	1.87 (1.87-1.87)	38,904	3.74 (3.74-3.74)
Single and recurrent depression (inpatient)	139,635	6.87 (6.87-6.88)	50,480	5.04 (5.04-5.04)	89,155	8.66 (8.66-8.66)
Anxiety disorder (inpatient)	151,293	6.93 (6.93-6.93)	60,464	5.60 (5.60-5.60)	90,829	8.23 (8.23-8.23)
Obsessive-compulsive disorder (inpatient)	4,446	0.20 (0.20-0.20)	1,823	0.17 (0.17-0.17)	2,623	0.23 (0.23-0.23)
Eating disorders (inpatient)	6,720	0.29 (0.29-0.29)	453	0.04 (0.04-0.04)	6,267	0.53 (0.53-0.53)
Anorexia nervosa (inpatient)	3,519	0.15 (0.15-0.15)	224	0.02 (0.02-0.02)	3,295	0.28 (0.28-0.28)
Personality disorders (inpatient)	109,026	5.37 (5.37-5.37)	45,596	4.56 (4.56-4.57)	63,430	6.16 (6.16-6.16)
Personality disorders (borderline-type) (inpatient)	11,007	0.54 (0.54-0.54)	2,468	0.24 (0.24-0.24)	8,539	0.82 (0.82-0.82)
Antisocial personality disorder (inpatient)	15,138	0.74 (0.74-0.74)	10,774	1.07 (1.07-1.07)	4,364	0.42 (0.42-0.42)
Intellectual disability (inpatient)	6,853	0.29 (0.29-0.29)	3,681	0.32 (0.32-0.32)	3,172	0.27 (0.27-0.27)
Developmental disorders (inpatient)	5,054	0.22 (0.22-0.22)	3,448	0.30 (0.30-0.30)	1,606	0.14 (0.14-0.14)
Childhood autism (inpatient)	1,009	0.04 (0.04-0.04)	737	0.06 (0.06-0.06)	272	0.02 (0.02-0.02)
Behavioral disorders (inpatient)	18,048	0.78 (0.78-0.78)	10,834	0.94 (0.94-0.94)	7,214	0.61 (0.61-0.61)
ADHD (inpatient)	7,270	0.31 (0.31-0.31)	4,824	0.42 (0.42-0.42)	2,446	0.21 (0.21-0.21)

eTable 5B.Case numbers and incidence rates for mental disorders in Denmark by time period, inpatient contacts only, both sexes

	Number of all cases, inpatient contacts only				Incidence rate in all, inpatient contacts only			
**Mental disorder**	**1970-1984**	**1985-1994**	**1995-2004**	**2005-2016**	**1970-1984**	**1985-1994**	**1995-2004**	**2005-2016**
Any mental disorder (inpatient)	209,124	80,298	79,144	97,320	28.88 (28.87-28.88)	17.00 (16.99-17.00)	16.64 (16.64-16.65)	16.74 (16.74-16.75)
Organic disorders (inpatient)	47,575	17,846	13,967	13,913	13.18 (13.17-13.18)	6.84 (6.83-6.84)	5.10 (5.10-5.11)	4.02 (4.02-4.02)
Dementia in alzheimer (inpatient)	22,069	9,028	2,792	2,875	6.09 (6.09-6.09)	3.44 (3.44-3.44)	1.02 (1.02-1.02)	0.83 (0.83-0.83)
Vascular dementia (inpatient)	9,492	2,662	2,356	1,262	2.62 (2.61-2.62)	1.01 (1.01-1.01)	0.86 (0.86-0.86)	0.36 (0.36-0.36)
Substance use disorder (inpatient)	61,079	25,388	22,356	29,739	9.55 (9.55-9.55)	5.85 (5.85-5.86)	5.21 (5.21-5.22)	5.62 (5.62-5.62)
Alcohol use disorder (inpatient)	47,026	20,705	16,579	20,368	7.34 (7.34-7.34)	4.76 (4.76-4.76)	3.86 (3.85-3.86)	3.84 (3.84-3.84)
Cannabis use disorder (inpatient)	2,784	1,961	3,390	7,619	0.43 (0.43-0.43)	0.45 (0.45-0.45)	0.78 (0.78-0.78)	1.43 (1.42-1.43)
Schizophrenia spectrum disorder (inpatient)	36,461	20,839	16,029	19,398	5.69 (5.69-5.69)	4.79 (4.79-4.79)	3.73 (3.73-3.73)	3.66 (3.66-3.66)
Schizophrenia (inpatient)	12,837	6,536	8,752	9,791	2.00 (2.00-2.00)	1.49 (1.49-1.49)	2.02 (2.02-2.03)	1.84 (1.84-1.84)
Schizoaffective disorders (inpatient)	3,434	1,874	1,845	1,399	0.53 (0.53-0.53)	0.43 (0.43-0.43)	0.43 (0.43-0.43)	0.26 (0.26-0.26)
Mood disorders (inpatient)	67,657	21,946	30,377	41,736	10.60 (10.59-10.60)	5.07 (5.06-5.07)	7.09 (7.09-7.10)	7.91 (7.91-7.92)
Bipolar disorder (inpatient)	9,872	4,860	5,660	8,197	1.53 (1.53-1.54)	1.11 (1.11-1.11)	1.31 (1.31-1.31)	1.53 (1.53-1.54)
Recurrent depression (inpatient)	17,779	7,477	12,733	19,760	2.77 (2.77-2.77)	1.71 (1.71-1.71)	2.94 (2.94-2.95)	3.71 (3.71-3.71)
Single and recurrent depression (inpatient)	58,221	18,355	26,722	36,337	9.11 (9.11-9.11)	4.23 (4.23-4.23)	6.23 (6.22-6.23)	6.87 (6.87-6.88)
Anxiety disorder (inpatient)	56,350	22,179	27,305	45,459	8.10 (8.10-8.11)	4.81 (4.80-4.81)	5.93 (5.92-5.93)	8.04 (8.03-8.04)
Obsessive-compulsive disorder (inpatient)	949	284	925	2,288	0.14 (0.14-0.14)	0.06 (0.06-0.06)	0.20 (0.20-0.20)	0.40 (0.40-0.40)
Eating disorders (inpatient)	819	932	2,029	2,940	0.11 (0.11-0.11)	0.19 (0.19-0.19)	0.41 (0.41-0.41)	0.49 (0.49-0.49)
Anorexia nervosa (inpatient)	708	619	751	1,441	0.10 (0.10-0.10)	0.13 (0.13-0.13)	0.15 (0.15-0.15)	0.24 (0.24-0.24)
Personality disorders (inpatient)	63,108	19,506	13,764	12,648	9.88 (9.87-9.88)	4.51 (4.50-4.51)	3.21 (3.21-3.22)	2.39 (2.39-2.39)
Personality disorders (borderline-type) (inpatient)	1,406	2,188	3,415	3,998	0.22 (0.22-0.22)	0.50 (0.50-0.50)	0.79 (0.79-0.79)	0.75 (0.75-0.75)
Antisocial personality disorder (inpatient)	11,984	1,304	1,007	843	1.86 (1.86-1.86)	0.30 (0.30-0.30)	0.23 (0.23-0.23)	0.16 (0.16-0.16)
Intellectual disability (inpatient)	1,766	893	1,762	2,432	0.24 (0.24-0.24)	0.18 (0.18-0.18)	0.36 (0.36-0.36)	0.40 (0.40-0.40)
Developmental disorders (inpatient)	527	502	1,097	2,928	0.07 (0.07-0.07)	0.10 (0.10-0.10)	0.22 (0.22-0.22)	0.48 (0.48-0.48)
Childhood autism (inpatient)	133	128	253	495	0.02 (0.02-0.02)	0.03 (0.03-0.03)	0.05 (0.05-0.05)	0.08 (0.08-0.08)
Behavioral disorders (inpatient)	4,792	3,275	2,615	7,366	0.65 (0.65-0.65)	0.67 (0.67-0.67)	0.53 (0.53-0.53)	1.22 (1.22-1.22)
ADHD (inpatient)	769	517	681	5,303	0.10 (0.10-0.10)	0.11 (0.11-0.11)	0.14 (0.14-0.14)	0.88 (0.88-0.88)

eTable 5C.Case numbers and incidence rates for mental disorders in Denmark by time period, inpatient contacts only, males only

	Number of male cases, inpatient contacts only				Incidence rate in males, inpatient contacts only			
**Mental disorder**	**1970-1984**	**1985-1994**	**1995-2004**	**2005-2016**	**1970-1984**	**1985-1994**	**1995-2004**	**2005-2016**
Any mental disorder (inpatient)	93,808	37,185	37,533	47,723	26.15 (26.14-26.16)	15.93 (15.93-15.94)	15.92 (15.91-15.92)	16.49 (16.49-16.50)
Organic disorders (inpatient)	22,061	8,007	6,557	6,939	12.87 (12.87-12.88)	6.49 (6.49-6.50)	5.02 (5.02-5.02)	4.15 (4.15-4.15)
Dementia in alzheimer (inpatient)	9,331	3,777	1,172	1,269	5.42 (5.42-5.43)	3.05 (3.05-3.05)	0.89 (0.89-0.89)	0.76 (0.76-0.76)
Vascular dementia (inpatient)	4,825	1,370	1,197	635	2.80 (2.80-2.80)	1.10 (1.10-1.11)	0.91 (0.91-0.91)	0.38 (0.38-0.38)
Substance use disorder (inpatient)	37,762	15,447	14,209	19,521	12.04 (12.03-12.04)	7.30 (7.29-7.30)	6.77 (6.77-6.77)	7.50 (7.50-7.50)
Alcohol use disorder (inpatient)	32,353	13,163	10,559	13,346	10.30 (10.30-10.30)	6.21 (6.20-6.21)	5.02 (5.02-5.02)	5.11 (5.11-5.11)
Cannabis use disorder (inpatient)	2,129	1,584	2,682	5,617	0.67 (0.67-0.67)	0.74 (0.74-0.74)	1.26 (1.26-1.26)	2.13 (2.13-2.13)
Schizophrenia spectrum disorder (inpatient)	16,006	9,162	8,276	10,182	5.08 (5.08-5.08)	4.30 (4.30-4.30)	3.92 (3.92-3.92)	3.89 (3.89-3.89)
Schizophrenia (inpatient)	7,196	3,884	5,067	5,496	2.28 (2.28-2.28)	1.82 (1.81-1.82)	2.39 (2.39-2.39)	2.09 (2.09-2.09)
Schizoaffective disorders (inpatient)	1,265	673	722	550	0.40 (0.40-0.40)	0.31 (0.31-0.31)	0.34 (0.34-0.34)	0.21 (0.21-0.21)
Mood disorders (inpatient)	23,941	7,777	11,869	17,446	7.61 (7.61-7.61)	3.65 (3.65-3.65)	5.62 (5.62-5.62)	6.68 (6.67-6.68)
Bipolar disorder (inpatient)	3,868	1,825	2,306	3,581	1.22 (1.22-1.22)	0.85 (0.85-0.85)	1.08 (1.08-1.08)	1.36 (1.36-1.36)
Recurrent depression (inpatient)	5,102	2,089	4,217	7,437	1.61 (1.61-1.61)	0.97 (0.97-0.97)	1.98 (1.98-1.98)	2.82 (2.82-2.82)
Single and recurrent depression (inpatient)	19,366	6,086	10,187	14,841	6.15 (6.15-6.15)	2.85 (2.85-2.85)	4.82 (4.81-4.82)	5.67 (5.66-5.67)
Anxiety disorder (inpatient)	19,923	8,524	11,312	20,705	5.80 (5.80-5.80)	3.75 (3.75-3.75)	4.97 (4.96-4.97)	7.37 (7.37-7.37)
Obsessive-compulsive disorder (inpatient)	360	122	414	927	0.10 (0.10-0.10)	0.05 (0.05-0.05)	0.18 (0.18-0.18)	0.33 (0.33-0.33)
Eating disorders (inpatient)	79	60	102	212	0.02 (0.02-0.02)	0.02 (0.02-0.02)	0.04 (0.04-0.04)	0.07 (0.07-0.07)
Anorexia nervosa (inpatient)	57	42	39	86	0.02 (0.02-0.02)	0.02 (0.02-0.02)	0.02 (0.02-0.02)	0.03 (0.03-0.03)
Personality disorders (inpatient)	28,440	7,536	5,332	4,288	9.06 (9.05-9.06)	3.55 (3.55-3.55)	2.53 (2.53-2.53)	1.64 (1.64-1.64)
Personality disorders (borderline-type) (inpatient)	644	793	598	433	0.20 (0.20-0.20)	0.37 (0.37-0.37)	0.28 (0.28-0.28)	0.16 (0.16-0.16)
Antisocial personality disorder (inpatient)	8,211	944	864	755	2.60 (2.60-2.60)	0.44 (0.44-0.44)	0.41 (0.41-0.41)	0.29 (0.29-0.29)
Intellectual disability (inpatient)	936	463	977	1,305	0.26 (0.26-0.26)	0.19 (0.19-0.19)	0.40 (0.40-0.40)	0.44 (0.44-0.44)
Developmental disorders (inpatient)	356	377	891	1,824	0.10 (0.10-0.10)	0.16 (0.16-0.16)	0.37 (0.37-0.37)	0.61 (0.61-0.61)
Childhood autism (inpatient)	89	94	201	353	0.02 (0.02-0.02)	0.04 (0.04-0.04)	0.08 (0.08-0.08)	0.12 (0.12-0.12)
Behavioral disorders (inpatient)	2,963	1,990	1,732	4,149	0.81 (0.81-0.81)	0.83 (0.83-0.83)	0.71 (0.71-0.71)	1.39 (1.39-1.39)
ADHD (inpatient)	600	395	564	3,265	0.16 (0.16-0.16)	0.16 (0.16-0.16)	0.23 (0.23-0.23)	1.09 (1.09-1.09)

eTable 5D.Case numbers and incidence rates for mental disorders in Denmark by time period, inpatient contacts only, females only

	Number of female cases, inpatient contacts only				Incidence rate in females, inpatient contacts only			
**Mental disorder**	**1970-1984**	**1985-1994**	**1995-2004**	**2005-2016**	**1970-1984**	**1985-1994**	**1995-2004**	**2005-2016**
Any mental disorder (inpatient)	115,316	43,113	41,611	49,597	31.55 (31.54-31.56)	18.04 (18.03-18.05)	17.36 (17.35-17.36)	16.99 (16.98-17.00)
Organic disorders (inpatient)	25,514	9,839	7,410	6,974	13.45 (13.45-13.46)	7.14 (7.14-7.15)	5.18 (5.18-5.18)	3.90 (3.90-3.90)
Dementia in alzheimer (inpatient)	12,738	5,251	1,620	1,606	6.69 (6.69-6.69)	3.79 (3.79-3.79)	1.13 (1.13-1.13)	0.89 (0.89-0.90)
Vascular dementia (inpatient)	4,667	1,292	1,159	627	2.45 (2.45-2.45)	0.93 (0.93-0.93)	0.81 (0.80-0.81)	0.35 (0.35-0.35)
Substance use disorder (inpatient)	23,317	9,941	8,147	10,218	7.15 (7.15-7.16)	4.48 (4.48-4.48)	3.72 (3.72-3.72)	3.80 (3.80-3.80)
Alcohol use disorder (inpatient)	14,673	7,542	6,020	7,022	4.49 (4.49-4.49)	3.39 (3.39-3.39)	2.74 (2.74-2.74)	2.61 (2.60-2.61)
Cannabis use disorder (inpatient)	655	377	708	2,002	0.20 (0.20-0.20)	0.17 (0.17-0.17)	0.32 (0.32-0.32)	0.74 (0.74-0.74)
Schizophrenia spectrum disorder (inpatient)	20,455	11,677	7,753	9,216	6.28 (6.28-6.28)	5.26 (5.26-5.26)	3.54 (3.54-3.55)	3.43 (3.43-3.43)
Schizophrenia (inpatient)	5,641	2,652	3,685	4,295	1.73 (1.73-1.73)	1.19 (1.19-1.19)	1.67 (1.67-1.67)	1.59 (1.59-1.59)
Schizoaffective disorders (inpatient)	2,169	1,201	1,123	849	0.66 (0.66-0.66)	0.54 (0.54-0.54)	0.51 (0.51-0.51)	0.31 (0.31-0.31)
Mood disorders (inpatient)	43,716	14,169	18,508	24,290	13.50 (13.49-13.50)	6.43 (6.43-6.44)	8.52 (8.52-8.53)	9.13 (9.13-9.13)
Bipolar disorder (inpatient)	6,004	3,035	3,354	4,616	1.84 (1.83-1.84)	1.36 (1.36-1.36)	1.52 (1.52-1.52)	1.71 (1.71-1.71)
Recurrent depression (inpatient)	12,677	5,388	8,516	12,323	3.88 (3.88-3.88)	2.42 (2.42-2.42)	3.87 (3.87-3.88)	4.57 (4.57-4.58)
Single and recurrent depression (inpatient)	38,855	12,269	16,535	21,496	11.98 (11.97-11.98)	5.56 (5.56-5.56)	7.60 (7.59-7.60)	8.06 (8.06-8.06)
Anxiety disorder (inpatient)	36,427	13,655	15,993	24,754	10.35 (10.35-10.36)	5.83 (5.83-5.83)	6.86 (6.86-6.87)	8.69 (8.69-8.70)
Obsessive-compulsive disorder (inpatient)	589	162	511	1,361	0.17 (0.17-0.17)	0.07 (0.07-0.07)	0.22 (0.22-0.22)	0.47 (0.47-0.47)
Eating disorders (inpatient)	740	872	1,927	2,728	0.20 (0.20-0.20)	0.35 (0.35-0.35)	0.77 (0.77-0.77)	0.89 (0.89-0.90)
Anorexia nervosa (inpatient)	651	577	712	1,355	0.17 (0.17-0.17)	0.23 (0.23-0.23)	0.28 (0.28-0.28)	0.44 (0.44-0.44)
Personality disorders (inpatient)	34,668	11,970	8,432	8,360	10.67 (10.67-10.68)	5.43 (5.42-5.43)	3.88 (3.88-3.88)	3.13 (3.13-3.13)
Personality disorders (borderline-type) (inpatient)	762	1,395	2,817	3,565	0.23 (0.23-0.23)	0.62 (0.62-0.62)	1.28 (1.28-1.28)	1.32 (1.32-1.32)
Antisocial personality disorder (inpatient)	3,773	360	143	88	1.15 (1.15-1.15)	0.16 (0.16-0.16)	0.06 (0.06-0.06)	0.03 (0.03-0.03)
Intellectual disability (inpatient)	830	430	785	1,127	0.22 (0.22-0.22)	0.17 (0.17-0.17)	0.31 (0.31-0.31)	0.37 (0.37-0.37)
Developmental disorders (inpatient)	171	125	206	1,104	0.05 (0.05-0.05)	0.05 (0.05-0.05)	0.08 (0.08-0.08)	0.36 (0.36-0.36)
Childhood autism (inpatient)	44	34	52	142	0.01 (0.01-0.01)	0.01 (0.01-0.01)	0.02 (0.02-0.02)	0.05 (0.05-0.05)
Behavioral disorders (inpatient)	1,829	1,285	883	3,217	0.49 (0.49-0.49)	0.52 (0.52-0.52)	0.35 (0.35-0.35)	1.06 (1.05-1.06)
ADHD (inpatient)	169	122	117	2,038	0.05 (0.05-0.05)	0.05 (0.05-0.05)	0.05 (0.05-0.05)	0.67 (0.67-0.67)

eTable 6. Effects of shift in disease classification in 1994 (to ICD-10) and the inclusion of outpatient contacts in the registers in 1995 on the change in incidence of each mental disorder, estimated as hazard ratios (HRs) with 95% confidence intervals by Cox regression.

Mental disorder	All			Males			Females	
	ICD-10 shift, HR (95% CI)	Outpatient, HR (95% CI)		ICD-10 shift, HR (95% CI)	Outpatient, HR (95% CI)		ICD-10 shift, HR (95% CI)	Outpatient, HR (95% CI)
Any mental disorder	1.3 (1.2-1.4)	2.1 (2.0-2.1)		1.3 (1.2-1.4)	2.1 (2.0-2.2)		1.3 (1.2-1.4)	2.0 (1.9-2.1)
Organic disorders	1.2 (1.1-1.4)	1.8 (1.6-1.9)		1.2 (1.0-1.4)	1.8 (1.5-2.0)		1.2 (1.1-1.4)	1.8 (1.7-2.0)
Alzheimer’s disease	0.6 (0.5-0.7)	2.2 (1.9-2.6)		0.5 (0.4-0.7)	2.1 (1.7-2.7)		0.6 (0.5-0.8)	2.3 (1.9-2.7)
Vascular dementia	1.6 (1.2-2.1)	2.3 (1.9-2.7)		1.7 (1.2-2.3)	2.1 (1.6-2.8)		1.5 (1.2-2.0)	2.5 (2.0-3.0)
Substance use disorder	1.4 (1.3-1.6)	2.1 (1.9-2.3)		1.4 (1.3-1.7)	2.2 (1.9-2.4)		1.4 (1.3-1.6)	2.0 (1.8-2.2)
Alcohol use disorder	1.4 (1.3-1.6)	2.2 (2.0-2.4)		1.4 (1.2-1.6)	2.2 (1.9-2.5)		1.5 (1.4-1.8)	2.2 (2.0-2.5)
Cannabis use disorder	1.6 (1.2-2.2)	1.8 (1.4-2.3)		1.6 (1.0-2.7)	1.7 (1.1-2.6)		1.4 (0.9-2.1)	2.4 (1.7-3.3)
Schizophrenia spectrum disorder	0.8 (0.7-0.9)	1.4 (1.3-1.6)		0.9 (0.8-1.0)	1.5 (1.3-1.8)		0.7 (0.6-0.8)	1.4 (1.2-1.5)
Schizophrenia	1.0 (0.8-1.1)	1.4 (1.2-1.6)		0.9 (0.8-1.2)	1.4 (1.2-1.7)		1.0 (0.8-1.2)	1.4 (1.2-1.7)
Schizoaffective disorders	0.9 (0.7-1.2)	1.3 (1.0-1.7)		1.1 (0.7-1.6)	1.2 (0.8-1.7)		0.8 (0.6-1.1)	1.3 (1.0-1.8)
Mood disorders	1.2 (1.1-1.3)	1.7 (1.6-1.8)		1.2 (1.0-1.3)	1.8 (1.6-2.0)		1.2 (1.1-1.3)	1.7 (1.5-1.8)
Bipolar disorder	1.2 (1.0-1.5)	1.3 (1.1-1.5)		1.1 (0.9-1.4)	1.5 (1.2-1.9)		1.3 (1.1-1.6)	1.2 (1.0-1.4)
Recurrent depression	1.3 (1.1-1.4)	1.7 (1.5-1.9)		1.3 (1.1-1.6)	1.9 (1.6-2.3)		1.2 (1.1-1.4)	1.6 (1.5-1.9)
Single and recurrent depression	1.1 (1.0-1.2)	1.8 (1.6-1.9)		1.1 (1.0-1.3)	1.9 (1.7-2.2)		1.1 (1.0-1.2)	1.7 (1.6-1.9)
Anxiety disorder	1.5 (1.3-1.6)	2.4 (2.2-2.6)		1.5 (1.3-1.7)	2.6 (2.4-2.9)		1.4 (1.3-1.6)	2.3 (2.1-2.5)
Obsessive-compulsive disorder	1.6 (0.9-2.6)	2.0 (1.4-3.0)		2.0 (1.0-4.0)	1.5 (0.9-2.7)		1.2 (0.7-2.2)	2.6 (1.7-4.0)
Eating disorders	1.5 (1.1-2.2)	2.4 (1.9-3.1)		0.9 (0.4-1.8)	3.2 (1.7-6.0)		1.6 (1.1-2.2)	2.4 (1.9-3.0)
Anorexia nervosa	0.8 (0.5-1.5)	1.9 (1.2-3.0)		0.9 (0.4-2.5)	1.4 (0.6-3.3)		0.8 (0.5-1.5)	2.0 (1.3-3.0)
Personality disorders	1.0 (0.9-1.2)	2.1 (1.9-2.3)		0.9 (0.7-1.0)	2.0 (1.8-2.3)		1.2 (1.1-1.3)	2.2 (2.0-2.4)
Personality disorders (borderline-type)	1.7 (1.4-2.1)	1.5 (1.2-1.7)		1.1 (0.8-1.4)	0.7 (0.6-0.9)		2.2 (1.8-2.7)	1.7 (1.4-2.1)
Antisocial personality disorder	1.5 (1.0-2.4)	1.8 (1.2-2.7)		1.9 (0.9-3.9)	1.8 (0.9-3.8)		0.8 (0.4-1.4)	1.8 (1.0-3.1)
Intellectual disability	4.3 (3.2-5.8)	1.2 (1.0-1.5)		4.3 (3.0-6.2)	1.4 (1.1-1.8)		4.2 (3.1-5.8)	1.1 (0.9-1.3)
Developmental disorders	1.5 (1.0-2.4)	1.8 (1.4-2.3)		1.6 (0.9-3.0)	1.7 (1.1-2.8)		1.3 (0.8-2.2)	1.8 (1.3-2.5)
Childhood autism	1.0 (0.5-2.1)	1.1 (0.7-1.8)		1.2 (0.4-3.2)	1.2 (0.5-2.6)		0.8 (0.3-1.7)	1.0 (0.6-1.9)
Behavioral disorders	1.2 (1.0-1.5)	2.5 (2.1-2.9)		1.4 (1.0-1.9)	2.3 (1.8-3.0)		1.0 (0.8-1.3)	2.8 (2.3-3.4)
ADHD	1.6 (0.9-3.0)	5.0 (3.5-7.3)		1.9 (0.6-5.6)	3.8 (1.5-10.0)		0.7 (0.3-1.6)	15.7 (7.8-31.4)
